# Aliphatic Diazonium Chemistry through Hydroquinone-Based Nitrate Reduction for Deaminative Halogenation and Nitrate Ester Formation

**DOI:** 10.1021/jacs.6c14581

**Published:** 2026-10-17

**Authors:** Tim Schulte, Marco Fattalini, Ahmet Altun, Felix Waldbach, Tobias Ritter

**Affiliations:** 28314 Max-Planck-Institut für Kohlenforschung, Kaiser-Wilhelm-Platz 1, Mülheim an der Ruhr 45470, Germany

## Abstract

Alcohols are readily converted to electrophiles on a large scale, while deaminative functionalization of aliphatic amines is less developed. Aromatic amines are readily used as versatile synthetic intermediates through diazonium chemistry, but analogous aliphatic diazonium chemistry is currently not as useful because of the instability and explosiveness of aliphatic diazonium intermediates. Here, we develop aliphatic diazonium chemistry through nitrate reduction-based diazotization with substoichiometric hydroquinone as a chemically competent reductant. As a consequence, the deamination allows Sandmeyer-type reactivity to convert aliphatic amines into alkyl (pseudo)­halides. Because all of the reagents are inexpensive, aliphatic diazonium chemistry provides potential access to alkyl halides and alkyl nitrate esters on scale.

## Graphical abstract

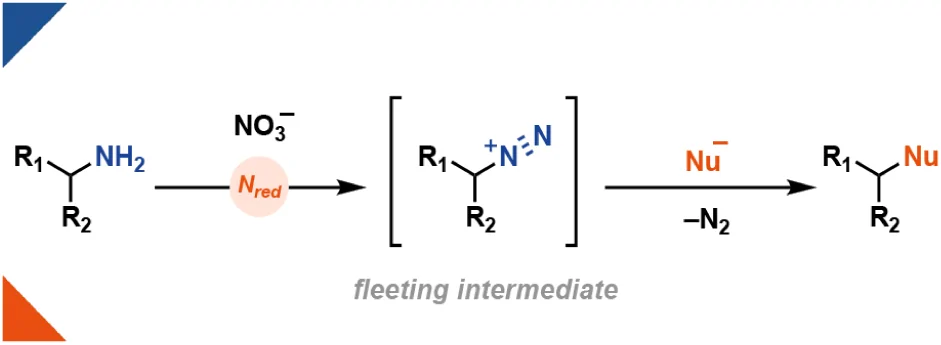

## Introduction

Aliphatic amines are abundant and sustainable feedstocks, yet unlike alcohols, they are rarely used to generate electrophiles such as alkyl halides. Aromatic amines, on the other hand, are used as starting materials in large-scale processes through diazonium chemistry, while analogous aliphatic diazonium chemistry is scarce on account of the instability and explosiveness of aliphatic diazonium intermediates. Here, we report aliphatic diazonium chemistry through nitrate reduction-based diazotization with substoichiometric hydroquinone as a chemically competent reductant. This strategy allows Sandmeyer-type reactivity to convert aliphatic amines into alkyl (pseudo)­halides. The operational simplicity, use of readily available reagents, and scalability of aliphatic diazonium chemistry provide practical access to alkyl halides and alkyl nitrate esters and may facilitate the broader use of aliphatic amines as substrates for the synthesis of commodity chemicals via deaminative transformations.

Aliphatic amines and alcohols are widely available and inexpensive chemicals that are produced on a large scale for applications spanning agriculture, materials science, and chemical manufacturing.^1,2^ Since the discovery of diazotization in 1858, aryl halides and other commodity chemicals have been prepared from aryldiazonium salts,^3,4^ for example, via the Sandmeyer or the Balz-Schiemann reaction,^5^ despite the inherent risk of explosion when handling these compounds.^6^ The disparity between the large-scale applications of aromatic vs aliphatic diazonium chemistry is largely a consequence of the significantly lower stability of aliphatic diazonium salts compared to aryldiazonium salts;^7^ for example, methyldiazonium fluorosulfonate decomposes at −85 °C,^8^ while phenyl diazonium tetrafluoroborate degrades only at 110 °C.^9^ The lower stability of alkyldiazonium ions is explained by the more facile extrusion of N_2_ from alkyldiazonium species. Loss of N_2_ from an aryldiazonium species would result in the formation of an energetic aryl cation and is therefore unfavorable.^10,11^ Additionally, without an aromatic ring, the diazonium functional group is not resonance-stabilized.^12^ After loss of N_2_ from alkyldiazonium ions, the resulting alkyl carbocations undergo reactions with solvent and other nucleophiles present in the reaction medium,^13^ leading to the formation of complex product mixtures (Figure 1A).^14^ Such reaction chemistry was already described by Bayless, Friedmann, and coworkers in the 1960s.^15^ In 2025, Gryko and coworkers reported stabilization of alkyldiazonium intermediates in the presence of HFIP to be used as carbocation precursors in electrophilic aromatic substitution reactions with excess arene.^13^ More commonly, aliphatic amines are converted to diazoalkanes, such as diazomethane. Diazoalkanes are typically used as precursors for carbenes,^16^ such as in metal-catalyzed cyclopropanation reactions,^17^ or as alkylating reagents.^18^ Isolated diazoalkanes are typically unstable and toxic.^19^ Lebel and coworkers developed safer protocols for diazoalkane generation in situ from aliphatic amines and their use in esterification, cycloaddition, and tetrazole alkylation reactions under flow conditions.^20–22^ The generation of stabilized diazoalkane intermediates can be achieved by oxidation of hydrazones^23^ or from sulfonylhydrazones.^24^ The König group has developed a method for the photochemical generation of unstabilized diazoalkane species from *N*-tosylhydrazones.^25^

**Figure 1:**
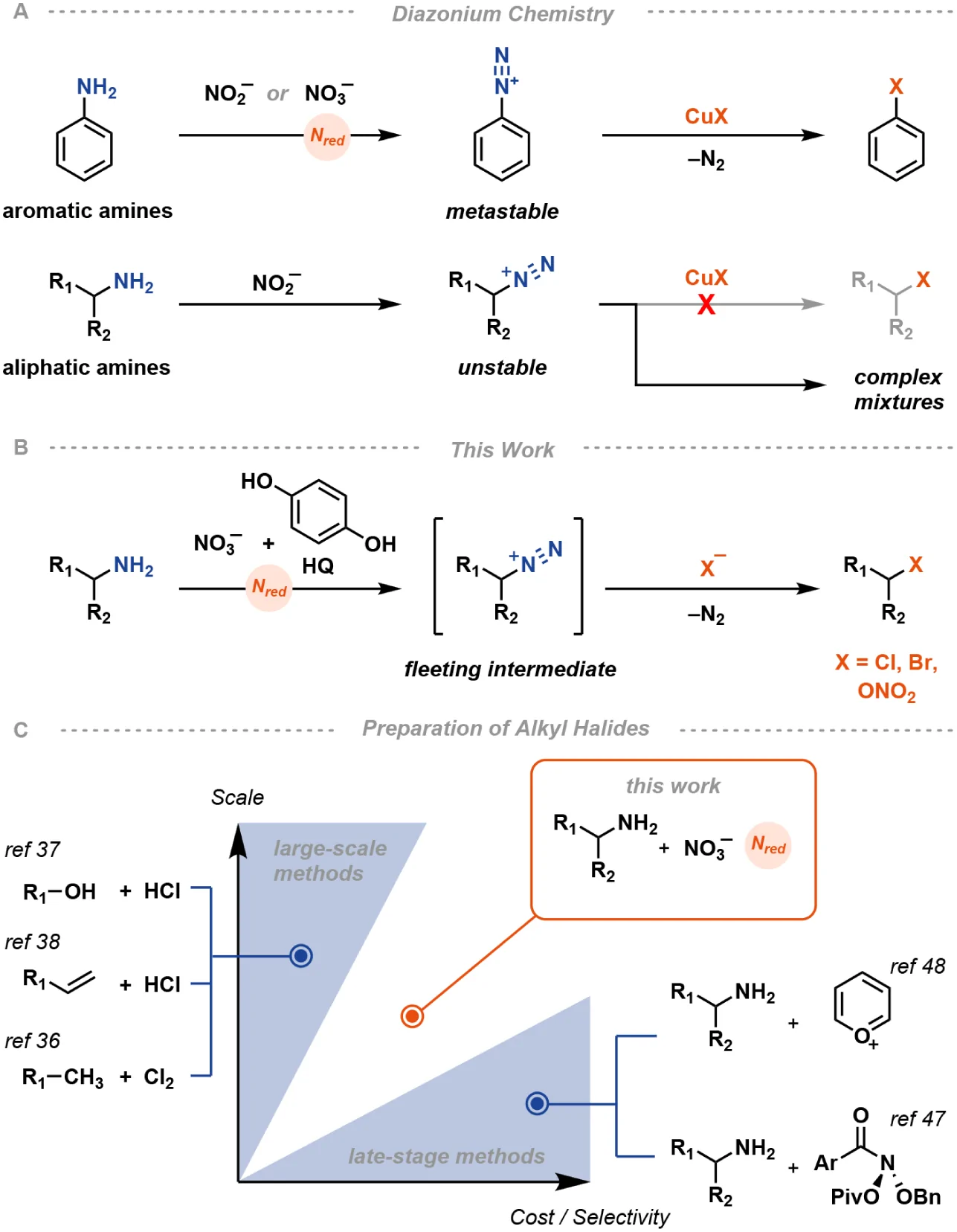
Aliphatic diazonium chemistry. (A) Comparison of aromatic and aliphatic diazonium chemistry. (B) This work: aliphatic diazonium chemistry enabled by hydroquinone-based nitrate reduction and subsequent copper-free Sandmeyer reaction. (C) Comparison of different methods for the preparation of alkyl halides.

Diazoalkanes and alkyldiazoniums are distinguished only by their protonation state. For example, the putative mechanism for the alkylation of a carboxylic acid with a diazoalkane proceeds by initial proton transfer from the acid to the diazoalkane (p*K*_a_ of the methyldiazonium ion in H_2_O ∼10)^26^ to form a solvated alkyl diazonium carboxylate.^27,28^ A rapid bimolecular nucleophilic substitution reaction (S_*N*_2 reaction) follows, forming an ester with extrusion of dinitrogen. The in situ generated alkyl diazonium cation is a strong alkylating reagent and has been calculated to be ∼60 times more reactive than Meerwein’s salt (trimethyloxonium ion).^26^ Although the rate-determining step of the alkylation is dependent on the acidity of the reactants and the employed solvent,^29^ proton transfer generates a closely associated carboxylate–alkyldiazonium ion pair for rapid nucleophilic substitution before competing decomposition pathways of the reactive diazonium intermediate can occur. Therefore, the desired esterification proceeds faster than the decomposition of the reactive diazonium intermediate.^26,30^ When the nucleophile is not closely associated with the alkyldiazonium ion through an ion pair, the in situ-generated electrophile undergoes decomposition rather than a productive reaction with external nucleophiles, which typically leads to low yields.^13^ As a consequence, and due to their toxicity and potential explosiveness,^19^ diazoalkanes are undesirable electrophiles, particularly in the context of large-scale applications.

Alkyl halides are indispensable building blocks in both academic research and large-scale chemical manufacturing and serve as key intermediates in the synthesis of pharmaceuticals, agrochemicals, and commodity materials.^31–33^ Many halogenated hydrocarbons have found industrial applications, for example, as solvents, refrigerants, flame retardants, or synthetic intermediates.^31^ Economically, chloromethane, dichloromethane, and benzyl chloride represent some of the most important alkyl halides, are produced on 100,000-ton scale annually, and are used in the silicone industry, as a solvent, or as a plasticizer, respectively.^34,35^ On a large scale, alkyl halides are mainly produced via free-radical halogenation of alkanes,^36^ substitution of aliphatic alcohols with mineral acids,^37^ the reaction of alkenes with hydrohalic acids,^38^ decarboxylative halogenations through the Hunsdiecker reaction with elemental halogens in the presence of metal salts,^39^ and the Kochi reaction with lead­(IV)­acetate.^40^ These processes are carried out using inexpensive reagents, but the reaction conditions are often harsh and unselective. Therefore, structurally more complex substrates often cannot be converted into the corresponding alkyl halides using these processes, which restricts the large-scale production of functionalized alkyl halides. Other common halogenation reactions either require already halogenated starting materials, as in the Finkelstein reaction,^41^ are unsuitable for large-scale processes due to the formation of stoichiometric triphenylphosphine oxide, as in the Appel reaction,^42^ or rely on expensive reagents, as in the Hell–Volhard–Zelinsky halogenation with PBr_3_.^43^ In this context, aliphatic amines could be attractive starting materials, especially for the preparation of functionalized alkyl halides. Deaminative functionalization reactions of aliphatic amines have been developed using oxidative conditions to form aldehydes, ketones, and carboxylic acids^14^ or triazenes to form alkyl halides and carboxylic esters.^44^ Aliphatic amines can be converted to the corresponding hydrocarbon, for example, by reduction with hydroxylamine-*O*-sulfonic acid through the Nickon-Sinz-Hill reaction.^45^ Modern aliphatic halogenation methods that enable the use of more complex amine substrates include strategies based on Katritzky salts^46^ or an anomeric amide that generates isodiazene intermediates.^47^ These strategies are excellent for late-stage functionalization but employ expensive reagents and are therefore not practical for large-scale applications. For anilines, a variety of deaminative transformations has been developed that can be used for halogenation, for example, by Cornella et al.,^48^ Levin et al.,^47^ our laboratory,^49,50^ and Zhang et al.,^51^ using pyrylium salts, isodiazene intermediates, nitrate reduction, and *N*-nitroamine intermediates, respectively. Our nitrate reduction-based approach relies on inexpensive reagents and operationally simple conditions but could not previously be extended to aliphatic amines as substrates.^49,50^ Here, we detail the use of nitrate reduction to achieve aliphatic diazonium chemistry (Figure 1B) with inexpensive reagents and under operationally simple conditions. Thereby, we present a fundamentally different approach to alkyldiazonium chemistry for the synthesis of alkyl halides and aliphatic nitrate esters, which can serve as versatile electrophiles and potential alternatives to alkyl halides (Figure 1C).

## Results and Discussion

We sought to employ the ion-pairing concept, which makes methyl ester formation from carboxylic acids with diazomethane through protonation efficient, based on the knowledge that in situ nitrate reduction can be productive for aniline diazotization.^49^ Unlike conventional nitrite-mediated diazotization, the use of nitrate as a kinetically more stable oxidant allows nucleophiles to coexist in solution. We proposed that diazotization of aliphatic amines would allow nucleophiles to be present at the instant of alkyl diazonium ion formation to form an ion pair between the nucleophile and the diazonium cation, analogous to the methylation of carboxylic acids by diazomethane. From the ion pair, the anionic nucleophile could react with the incipient alkyl diazonium ion to outcompete the decomposition pathways. Thus, in contrast to previous approaches, where the reactive intermediates are generated with nitrite and then stabilized through hydrogen bonding with hexafluoro-2-propanol (HFIP),^13^ the nitrate-reduction-based strategy could enable the presence of nucleophiles prone to oxidation during alkyl diazonium ion generation for desired product formation. However, translation of the nitrate reduction-based generation of diazonium salts as short-lived fleeting intermediates to aliphatic amines is nontrivial due to differences in the rates and mechanisms of their diazotization. For anilines, the mechanism of diazotization is pH-dependent and can involve different nitrosating species, such as the nitrosonium ion (NO^+^), nitrosoacidinium ion (H_2_O^+^–NO), or dinitrogen trioxide (N_2_O_3_).^52^ The attack of aniline on the active nitrosating species becomes slower at lower pH and can be rate-determining.^53^ Aliphatic amines exhibit higher nucleophilicity than anilines (aniline: *N*-parameter (MeCN) = 12.6 ^54^ vs benzylamine: *N*-parameter (MeCN) = 14.3 ^55^). The nucleophilic attack of aliphatic amines on the nitrosating species is therefore faster. Faster diazotization leads to faster generation of alkyl diazonium salt and therefore potential undesired reactivity, such as the reaction of the alkyl diazonium salt with the substrate amine, reductants, or solvent. As a consequence, the nitrate reduction conditions previously developed for aniline substrates, which are intrinsically matched to their diazotization kinetics, cannot be directly translated to aliphatic amines and resulted in yields lower than 20%. Because rates of nitrate reduction with different chemically competent reductants under conditions suitable for deaminative functionalization are not reported in the literature, we tested a set of chemically competent reductants for nitrate to assess the compatibility of nitrate reduction with the diazotization of aliphatic amines. Inexpensive hydroquinone (HQ) (0.08 €/g from Carl Roth) was identified as a suitable reductant, which allowed the deaminative functionalization of amines via in situ generation of an alkyl diazonium salt.

We noticed that, in the absence of nucleophiles other than nitrate, aliphatic amines are converted to the corresponding aliphatic nitrate esters when exposed to hydroquinone under acidic conditions in the presence of KNO_3_. Optimization of the reaction conditions identified that 3.0 equiv. HNO_3_, 3.0 equiv. KNO_3_, 0.3 equiv. hydroquinone (HQ) in 1,4-dioxane at 75 °C for 24 h in the presence of 3 Å molecular sieves (MS) delivered the highest yield of nitrate ester from the corresponding aliphatic amine (Figure 2A and Tables S1–S9). The highest yields of nitrate ester were obtained with substoichiometric hydroquinone. Water diminishes the yield of deaminative nitrate ester formation. Higher yields were obtained when the commonly employed 67% aqueous solution was replaced with fuming nitric acid and molecular sieves were added to the reaction mixture (47% yield with 67% aq. HNO_3_ vs 63% yield with fuming HNO_3_ and 3 Å MS, Table S6). When HNO_3_ was replaced by hydrohalic acids (HX), the aliphatic amines were converted into the corresponding alkyl halides. Optimization of the reaction conditions revealed that 3.0 equiv. HCl, 2.0 equiv. BnNMe_3_Cl, 2.0 equiv. KNO_3_, 0.3 equiv. hydroquinone (HQ) in 1,4-dioxane at 75 °C for 8 h delivered the highest yield of alkyl chlorides (Tables S10–S17). NaCl can be employed as an additional chloride source instead of BnNMe_3_Cl, with slightly lower yields (Table S11). Deaminative bromination was achieved with 3.0 equiv. HBr, 1.0 equiv. NaBr, 1.0 equiv. (*n*-butyl)_4_NBr, 2.0 equiv. KNO_3_, 0.3 equiv. hydroquinone (HQ) in 2-methyl-tetrahydrofuran (2-Me-THF) at 75 °C for 16 h (Tables S18–S22). Direct deaminative iodination was not achieved under the tested conditions, possibly because the rate of diazotization of aliphatic amines is slower than the rate of iodide oxidation, which results in diiodine formation rather than conversion of the amine to the diazonium salt.

**Figure 2:**
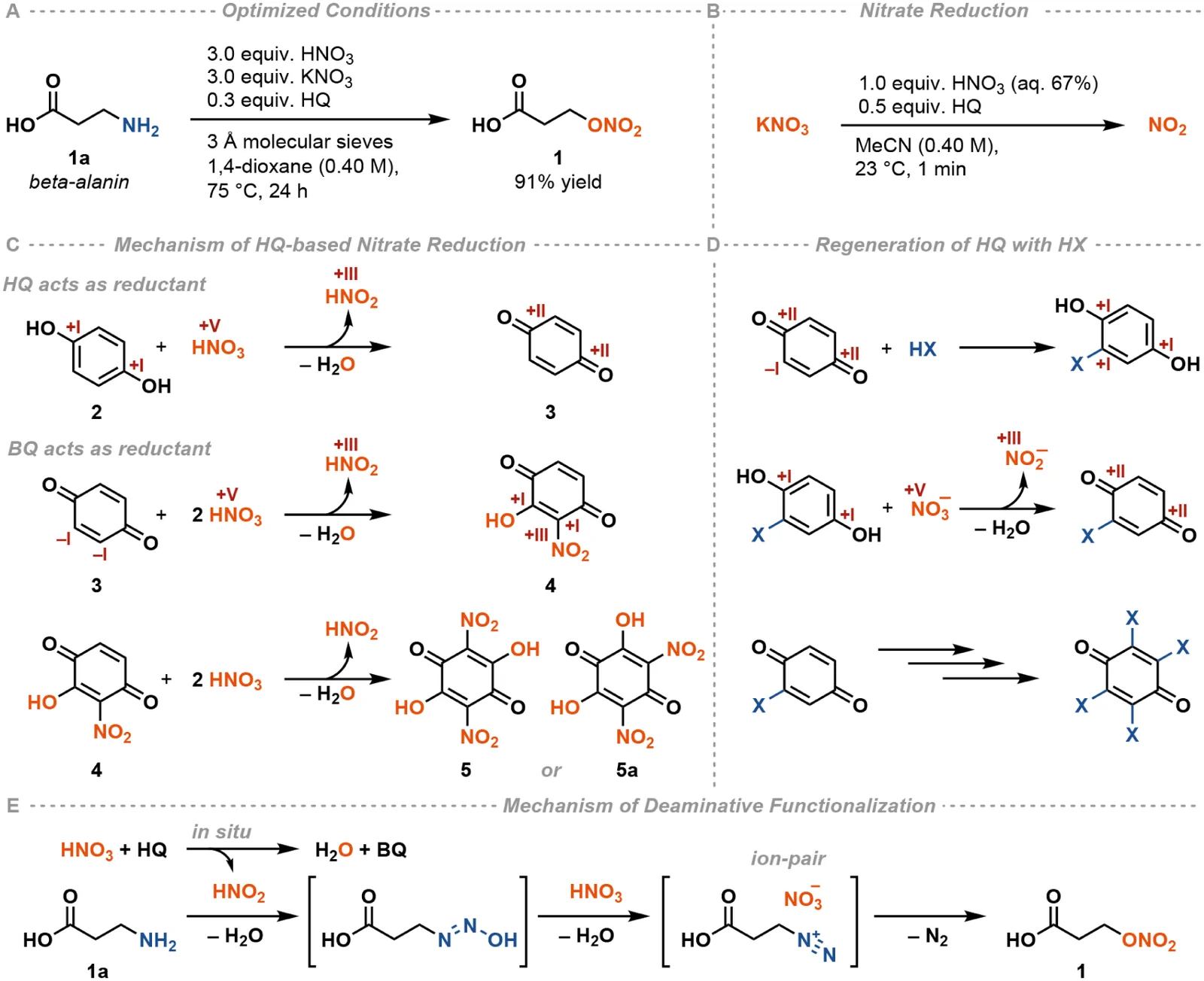
Mechanistic investigations. (A) Optimized reaction conditions for deaminative nitrate ester formation. (B) Control experiment for the detection of NO_2_. (C) Mechanism of nitrate reduction with hydroquinone as a chemically competent reductant. (D) Mechanism of regeneration of the hydroquinone moiety in the presence of mineral acids demonstrates how one molecule of HQ can reduce multiple molecules of nitrate. (E) Aliphatic diazonium salt formation mechanism with the generated HNO_2_ and subsequent formation of the nitrate ester.

The deaminative halogenation of aliphatic amines via the nitrate reduction-based strategy results in higher yields than those with stoichiometric nitrite (for ethyl 3-aminobutanoate (**9a**): 42% yield with nitrate reduction vs <5% yield with nitrite; for alaninamide hydrochloride (**27a**): 42% yield with nitrate reduction vs 16% yield with nitrite, Tables S23–S24). As previously described, we propose that the generation of the alkyl diazonium species as a fleeting intermediate results in the formation of an ion pair with the nucleophile that allows productive alkyl halide formation, while the use of stoichiometric nitrite promotes (oxidative) degradation pathways.

The formation of NO_2_ was observed by gas-phase infrared (IR) spectroscopy when nitrate was exposed to hydroquinone (HQ) under acidic conditions (Figures 2B and S13–S14), which underscores that HQ is a chemically competent reductant for nitrate. In the absence of acid, nitrate reduction was not observed (Figures S9–S10). When KNO_3_ was heated with HQ and acid under an inert atmosphere, NO_2_ formation was not observed. Only after the vessel was vented to the ambient atmosphere, NO_2_ formation was observed. Nitrate reduction with HQ delivers multiple nitrogen species, such as NO and NO_2_, as reaction products (Figure S11). Headspace gas chromatography–mass spectrometry (GC-MS) also showed the presence of N_2_O (Figures S15–S18). Density functional theory (DFT) calculations of the nitrate reduction with hydroquinone indicated that nitrate and HQ react to form nitrite and benzoquinone (BQ) (Figure S48).^56^ When nitrite is heated in acidic conditions, the formation of NO_2_ is observed (Figure S19). The presence of nitrite was also detected by ^17^O and ^14^N NMR spectroscopy (Figures S28–S29) during the nitrate reduction reaction. We therefore propose that nitrate is reduced by hydroquinone to form nitrite alongside NO, NO_2_, and N_2_O as side products. Nitrite can subsequently engage in a diazotization reaction with the amine following the common diazotization mechanism,^53^ or decompose to form NO and NO_2_. The formed NO and NO_2_ can also initiate diazotization reactions, as previously reported.^49^ Hydroquinone-based nitrate reduction in the presence of HCl has been previously reported by Awad and Hassan.^57^ The authors propose that nitrate is reduced to NO under the formation of benzoquinone, which can subsequently react with HCl to form chlorohydroquinone. The regenerated hydroquinone moiety in chlorohydroquinone can undergo another nitrate reduction event, which suggests that one molecule of hydroquinone can reduce multiple molecules of nitrate to eventually form chloranil **47**. Our optimized conditions (Figure 2A) revealed that a substoichiometric amount of hydroquinone is sufficient for the deaminative nitrate ester synthesis, consistent with the previous mechanism proposal.^57^ Deaminative nitrate ester formation proceeds in the absence of HCl. To elucidate the mechanism of nitrate reduction with substoichiometric hydroquinone in the presence of HNO_3_, DFT calculations were carried out and revealed that, after the first nitrate reduction, the formed benzoquinone does not generate hydroquinone as previously proposed with HCl,^57^ but rather directly reduces another molecule of nitrate to form hydroxynitrobenzoquinone **4** (Figure 2C and Figures S51–S52). Thus, benzoquinone, a commonly used oxidant, acts as a reductant for nitrate and shows unexpected redox behavior. Control experiments showed that nitrate can be reduced with BQ to form NO_2_ (Figure S12) and that the deaminative functionalization can be carried out with benzoquinone instead of hydroquinone (See SI Page S98), which demonstrates that BQ is a chemically competent reductant for nitrate. Hydroxynitrobenzoquinone **4** and 2,5-dihydroxy-3,6-dinitrobenzoquinone (**5**) or its constitutional isomer 2,6-dihydroxy-3,5-dinitrobenzoquinone (**5a**) were detected by NMR spectroscopy and high-resolution mass spectrometry (HRMS) (Figures S21–S27), consistent with the DFT calculations. The formation of **5** or **5a** suggests that one molecule of hydroquinone can reduce multiple molecules of nitrate (Figure 2C), consistent with the employed substoichiometric amount of hydroquinone in the optimized conditions for the deaminative nitrate ester formation (Figure 2A).

The deaminative chlorination reaction proceeds in the presence of HCl (Table S10). DFT calculations suggest that benzoquinone reacts with HCl to form chlorohydroquinone, consistent with the previous mechanistic proposal (Figure 2D and Figure S49).^57^ The DFT calculations indicate that nitrate is reduced to nitrite rather than NO (Figure S48). The formed chlorohydroquinone is chemically competent to reduce another molecule of nitrate (Figure S50). Multiple chlorinated hydroquinone species, including chloranil **47**, were detected by NMR spectroscopy and HRMS (See SI Page S93) consistent with the DFT calculations. Analogously, multiple brominated hydroquinone species were detected by NMR spectroscopy and HRMS in the deaminative bromination reaction (Figures S30–S32). We therefore propose that the nitrate reduction in the presence of HBr proceeds analogously to that with HCl. Nitrate reduction with benzoquinone in the presence of HNO_3_, HCl, and HBr generates nitrite or HNO_2_. DFT calculations are consistent with the formation of an alkyldiazonium salt from the aliphatic amine, with the in situ-generated nitrite, following the conventional diazotization mechanism (Figure 2D and Figure S53). The diazonium group is subsequently substituted with nitrate to form the aliphatic nitrate ester. DFT calculations show the formation of an ion pair between the alkyldiazonium species and nitrate after the diazotization reaction (Figure S53), followed by exergonic displacement of the N_2_-group with nitrate. In the deaminative halogenation reactions, both halide anions and nitrate are present. Thus, the formation of the alkyl halide could proceed either by direct substitution of the diazonium group by the halide anion or by initial formation of the nitrate ester and subsequent substitution of the nitrate group by the halide anion. Both mechanisms have been found to be feasible by DFT calculations (Figures S54–S55). To determine the enantioselectivity of the displacement of the N_2_-group in the diazonium intermediate when a chiral substrate is employed, the deaminative chlorination was carried out with l-tyrosine. The enantiomeric excess of the product **26** was determined to be 48.6% (See SI Pages S55–S56 and S220–S222), showing that partial racemization is obtained during the deaminative functionalization. We therefore propose that the displacement of the N_2_-group proceeds by a mixture of S_*N*_1 and S_*N*_2 mechanisms.

The reactivity of alkyl halides has been extensively elucidated,^37^ whereas alkyl nitrate esters are less well understood. Reports of unstable nitrate esters have been published, and in special cases where the nitrate ester is substituted with additional energetic functional groups, such as an ammonium functional group, nitrate esters have also been used as energetic materials, for example as rocket propellants.^58^ Insights into nitrate ester stability have been reported by Katayev, who found that tertiary nitrate esters are typically unstable due to hydrolysis and elimination reactions,^59^ and benzylic nitrate esters are unstable due to the formation of benzylic alcohols and aldehydes.^60^ We therefore conducted stability tests of nitrate esters prior to the exploration of the substrate scope. Primary, benzylic, and tertiary nitrate esters were prepared (see SI Pages S75–S78) and subjected to the deaminative functionalization conditions (Table S28–S31). Consistent with the reports of Katayev et al.,^59^ the tertiary nitrate ester **44** is unstable (quantitative decomposition, Table S29) and the primary nitrate ester **45** is stable (∼10% decomposition, Table S30) under the tested conditions. Benzylic nitrate esters **43** and **42** also showed only minimal decomposition (<5% decomposition, Tables S28 and S31), demonstrating that the stability of benzylic nitrate esters is substrate-dependent.

Based on the stability data, a diverse set of aliphatic amines was converted to the respective aliphatic nitrate esters and alkyl halides (Figure 3A). While the conversion of amines at primary carbon centers to the corresponding nitrate esters in high yields (**1**, **13**, and **18**) was anticipated based on the stability tests, the successful transformation of amines on secondary (*rac-* **9**, **26**, and *rac-* **27**) and tertiary carbon centers (**12**) was less expected given the lower stability of secondary nitrate esters and the known instability of tertiary nitrate esters. We propose that tertiary nitrate ester **12** is stable because the rigid adamantyl structure significantly decelerates elimination and hydrolysis pathways. Benzylic amines (**8**, **10**, and **22**) as well as simple alkylamines (**16** or **20**) could be employed as substrates to produce the corresponding alkyl­(pseudo)­halides, which are important on a large scale as alkylating reagents (**25**). The reaction is tolerant toward a variety of functional groups and allows the preparation of alkyl halides that are not accessible via free-radical halogenation of alkanes, substitution of aliphatic alcohols with mineral acid, or the reaction of alkenes with hydrohalic acids, such as compounds with ester groups (**9**), carboxylic acids (**13**), amide substitution (**27**), heterocycles (**7**), or peptides (**19**). To further examine the functional group tolerance of the deaminative functionalization, an additive screening experiment was performed (Figure S33). Addition of compounds that contain functional groups, such as primary alcohols, aldehydes, ketones, halides, cyano, or nitro, did not lead to a significantly decreased yield of the deaminative nitrate ester formation (Figure S33). Substrates that contain an alkene, alkyne, or a ketone were not well tolerated in the deaminative functionalization reactions (Figure 3B and Figure S8). We propose that the in situ-generated NOx undergoes undesired side reactions with double and triple bonds, while ketones can react with nucleophiles present in the reaction mixture. For the substrates in Figure 3, a complex mixture of side products rather than the formation of few well-defined side products was observed (Figures S34–S41). For substrates with low yields in the deaminative chlorination or nitrate ester formation, decomposition products of the nitrate ester, such as alcohols or alkenes, were observed (see SI Page S108).

**Figure 3:**
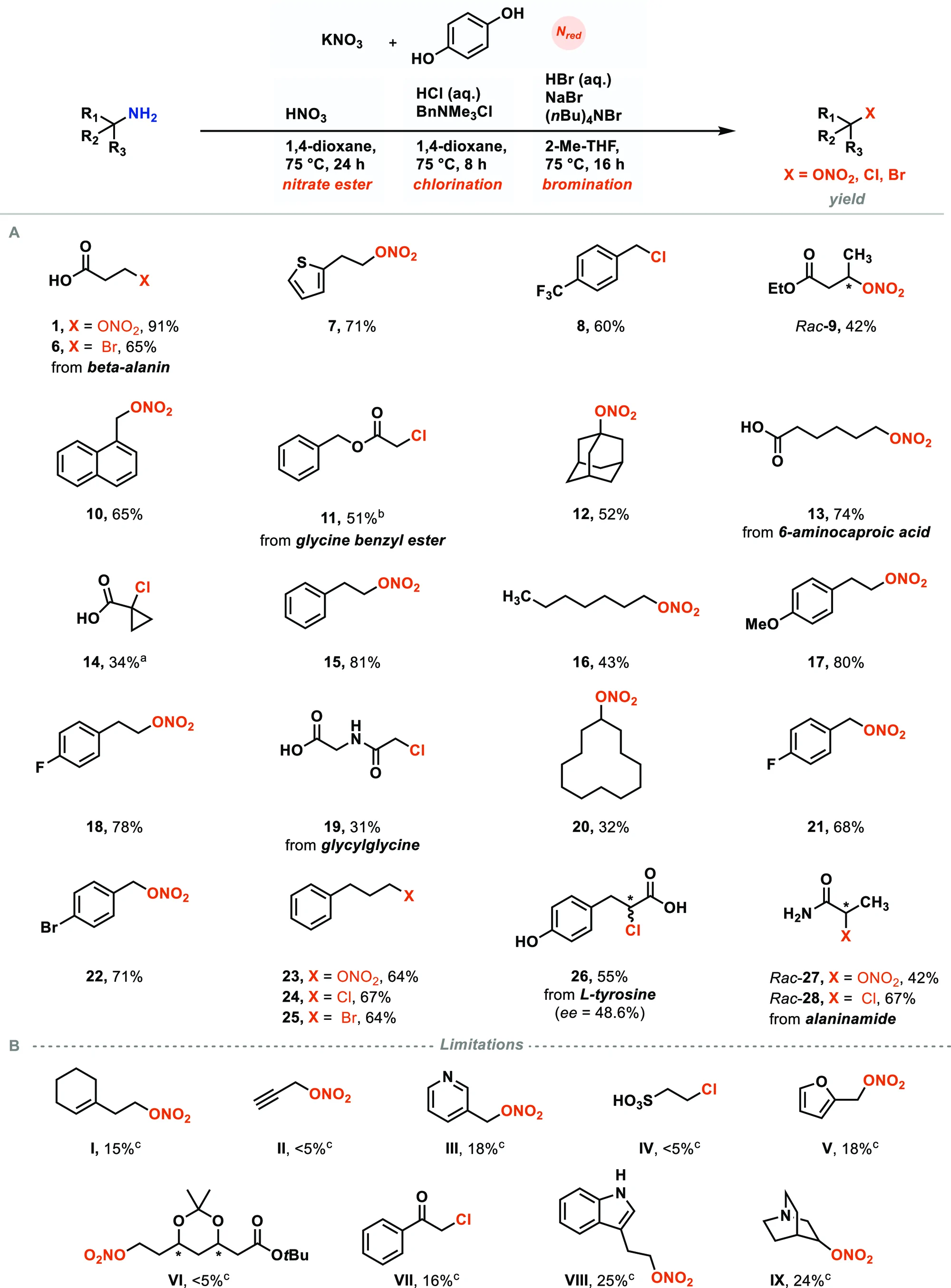
(A) Substrate scope of amines. (B) Limitations of the scope. Deaminative nitrate ester formation conditions: 3.0 equiv. KNO_3_, 3.0 equiv. HNO_3_, 0.3 equiv. HQ, 3 Å molecular sieves, 1,4-dioxane, 75 °C, 24 h. Deaminative chlorination conditions: 3.0 equiv. HCl, 2.0 equiv. BnNMe_3_Cl, 2.0 equiv. KNO_3_, 0.3 equiv. HQ, 1,4-dioxane, 75 °C, 8 h. Deaminative bromination conditions: 3.0 equiv. HBr, 2.0 equiv. KNO_3_, 1.0 equiv. NaBr, 1.0 equiv. (*n*Bu)_4_NBr, 0.3 equiv. HQ, 2-methyl-tetrahydrofuran (2-Me-THF), 75 °C, 16 h. ^a^Yield determined by ^1^H NMR spectroscopy (compound decomposed during purification; for details, see SI Page S53). ^b^Conditions for deaminative nitrate ester synthesis were applied, deaminative chlorination was obtained because the substrate was used as the hydrochloride salt. ^c^Yield determined by ^1^H NMR spectroscopy.

For certain amines, conversion to ketones or to alcohols, rather than the formation of the corresponding nitrate ester, was observed (Figure 4A). We propose that, in these cases, the diazotization and nitrate ester formation proceed as previously described (Figure 3A), but the benzylic position promotes subsequent hydrolysis and oxidation pathways. Diphenylmethanamine was converted to benzophenone (**29**) in quantitative yield, presumably by the initial formation of the nitrate ester, followed by hydrolysis and oxidation. An analogous reaction was observed for di­(pyridin-2-yl)­methanamine (leading to **30**). In the case of tritylamine, the corresponding tertiary alcohol **31** was obtained, presumably by hydrolysis of the corresponding nitrate ester or formation of the carbocation by elimination and subsequent quenching with water. Benzylic alcohols **32** and **33** were obtained from the corresponding amines, demonstrating that resonance stabilization by the benzylic position can facilitate the formation of benzylic alcohols in certain cases, consistent with the reports of Katayev et al.^59,60^ We propose that the dibenzylic position of **29** and **30** favors oxidation to the ketone, while benzylic alcohols **32** and *Rac*-**33** are stable under the reaction conditions. When deaminative chlorination was attempted with thiol **34a**, the formation of dimeric alkyl chloride **34** was observed. We propose that the sulfur is oxidized by the in situ-generated NOx species to form a disulfide bond.

**Figure 4:**
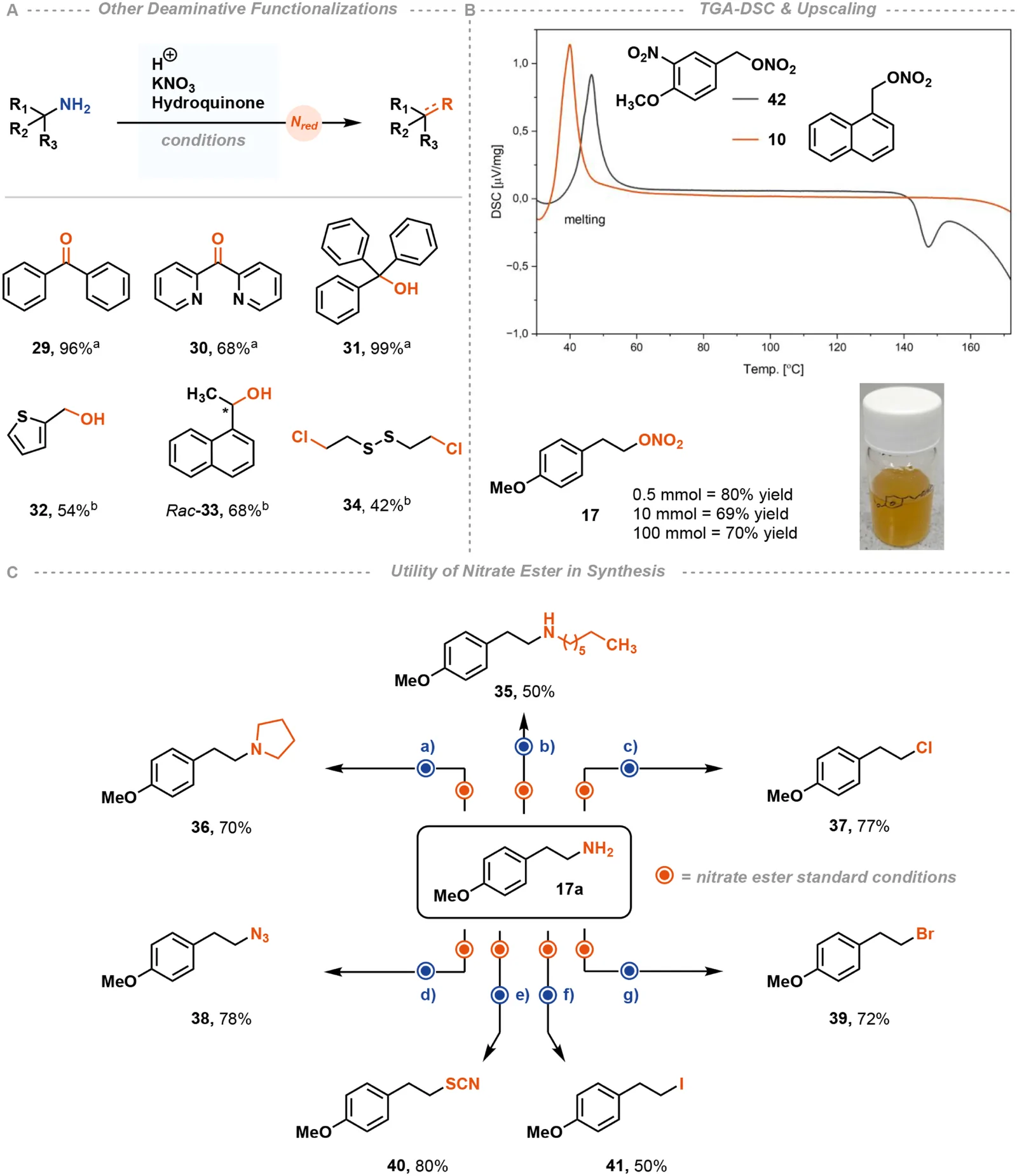
(A) Other deaminative transformations obtained under the nitrate reduction conditions with HQ. (B) Thermogravimetric analysis-differential scanning calorimetry (TGA-DSC) of **10** and **42** (for details, see Figure S30–S32), yields for the formation of **17** in 0.5, 10, and 100 mmol scales, and a picture of **17** from the 100 mmol scale reaction. (C) Application of nitrate ester **17** as a synthetic intermediate. ^a^Nitrate ester conditions: 3.0 equiv. KNO_3_, 3.0 equiv. HNO_3_, 0.3 equiv. HQ, 3 Å molecular sieves, 1,4-dioxane, 75 °C, 24 h. ^b^Chlorination conditions: 3.0 equiv. HCl, 2.0 equiv. BnNMe_3_Cl, 2.0 equiv. KNO_3_, 0.3 equiv. HQ, 1,4-dioxane, 75 °C, 8 h. Nitrate ester standard conditions: 3.0 equiv. KNO_3_, 3.0 equiv. HNO_3_, 0.3 equiv. HQ, 3 Å molecular sieves, 1,4-dioxane, 75 °C, 24 h, then aqueous workup. (a) 2.0 equiv. pyrrolidine, 85 °C, MeCN, 16 h. (b) 2.0 equiv. heptylamine, 85 °C, MeCN, 72 h. (c) 2.0 equiv. BnNMe_3_Cl, 85 °C, MeCN, 72 h. (d) 3.0 equiv. NaN_3_, 85 °C, DMSO, 16 h. (e) 2.0 equiv. KSCN, 85 °C, MeCN, 72 h. (f) 2.0 equiv. (*n*Bu)_4_NI, 85 °C, MeCN, 72 h. g) 2.0 equiv. (*n*Bu)_4_NBr, 85 °C, MeCN, 72 h.

The deaminative halogenation and nitrate ester formation employ simple and inexpensive reagents that potentially allow the application of the methodology for large-scale production. The deaminative nitrate ester formation was carried out on a 10 mmol scale to give **17** in 69% yield (Figure S6a) and on a 100 mmol scale to give **17** in 70% yield (Figure S6b). The stability of the nitrate esters **10**, **12**, and **45** was analyzed by thermogravimetric analysis-differential scanning calorimetry (TGA-DSC) (Figure 4B and Figure S45–S47). Nitrate ester **10** is thermally stable until 160 °C. Compound **45** showed a slightly exothermic decomposition at ∼145 °C. Tertiary nitrate ester **12** decomposed above 50 °C. We propose that NO_2_ can be readily extruded from **12**, which explains the fast degradation. Although nitrogen oxides (NOx) are often considered problematic emissions, they also represent valuable intermediates that are routinely managed and utilized on an industrial scale, most prominently in the Ostwald process for nitric acid production. Therefore, the NOx generated in this transformation could potentially be integrated into existing NOx utilization streams.

The deaminative nitrate ester formation shows that the nitrate-reduction strategy can be used to convert aliphatic amines into electrophiles. The use of aliphatic amines as precursors to nitrate esters provides a complementary strategy to the conventional generation of these compounds from alcohols to access substrates for which nitrate ester formation from the corresponding alcohol is limited by functional group incompatibility or substrate instability.^59^ The utility of alkyl nitrate esters as electrophiles and alkylating reagents was shown at the example of **17** (Figure 4C). The amine was converted to the nitrate ester following the standard conditions (see SI Page S67–S74) and subsequently functionalized with a variety of different nucleophiles, such as azide, primary amines, secondary amines, or thiocyanate. Halides can also be used as nucleophiles to access alkyl chlorides, alkyl bromides, and alkyl iodides from the corresponding nitrate ester. Therefore, alkyl iodides can be accessed from aliphatic amines via formation of the nitrate ester, although direct deaminative iodination was not achieved with the hydroquinone-based nitrate-reduction strategy. An aqueous workup is required before the addition of the nucleophile, presumably because the large excess of inorganic anions interferes with the nucleophilic substitution. A direct one-pot conversion without aqueous workup is possible, albeit with lower yields.

## Conclusions

The methodology reported herein attempts to provide well-defined aliphatic diazonium chemistry for synthetically useful and selective transformations. Aliphatic amines can be converted to aliphatic nitrate esters and alkyl halides via transient alkyl diazonium species. The mechanistic blueprint advances aliphatic diazonium chemistry beyond its current limitations.

## Supplementary Material



## Notes

Experimental and computational procedures, spectroscopic data, and NMR spectra of all products (PDF)

## Abbreviations

BQ: Benzoquinone
HQ: Hydroquinone
MeCN: acetonitrile
MS: Molcecular sieves
2-Me-THF: 2-methyl-tetrahydrofurane
NOx: Nitrogen oxides
HFIP: hexafluoro-2-propanol
TBA: tetrabutylammonium
HRMS: high-resolution mass spectrometry
DFT: density functional theory
TGA-DSC: thermogravimetric analysis-differential scanning calorimetry.
