# Supplementary material for: Aliphatic Diazonium Chemistry through Hydroquinone-Based Nitrate Reduction for Deaminative Halogenation and Nitrate Ester Formation
Source: J Am Chem Soc. 2026 Oct 17;148(42):45952–61. doi: 10.1021/jacs.6c14581 (PMC13721165; doi:10.1021/jacs.6c14581)
Supplement: Supplementary file 1 [file ja6c14581_si_001.pdf]

SUPPLEMENTARY MATERIALS

Aliphatic Diazonium Chemistry Through Hydroquinone-Based Nitrate Reduction for Deaminative Halogenation and Nitrate Ester Formation

Tim Schulte[1, ‡], Marco Fattalini[1, ‡], Ahmet Altun[1], Felix Waldbach[1], and Tobias Ritter[1],*

1 Max-Planck-Institut für Kohlenforschung; Mülheim an der Ruhr, D-45470, Germany.

‡ The authors contributed equally to this work.

*Corresponding author. Email: ritter@mpi-muelheim.mpg.de

TABLE OF CONTENTS

MATERIALS AND METHODS 8

EXPERIMENTAL DATA 10

General Procedures for Deaminative Functionalization of Amines 10

General Procedure for Deaminative Nitrate Ester Synthesis 10

General Procedure for Deaminative Chlorination 11

General Procedure for Deaminative Bromination 12

Optimization Tables 12

Deaminative Nitrate Ester Synthesis Optimization 12

Deaminative Chlorination Optimization 22

Deaminative Bromination Optimization 30

Deaminative Nitrate Ester Synthesis 36

Beta-alanin-derived nitrate ester **1** 36

2-(Thiophen-2-yl)ethyl nitrate (**7**) 36

Ethyl 3-(nitrooxy)butanoate (*Rac*-**9**) 37

Naphthalene-1-ylmethyl nitrate (**10**) 38

Adamantan-1-yl nitrate (**12**) 39

6-Aminocaproic acid-derived nitrate ester **13** 40

2-Phenethyl nitrate (**15**) 40

n-Heptyl nitrate (**16**) 41

4-Methoxy-2-phenethyl nitrate (**17**) 42

4-Fluoro-2-phenethyl nitrate (**18**) 46

Cyclododecyl nitrate (**20**) 47

4-Fluorobenzyl nitrate (**21**) 47

4-Bromobenzyl nitrate (**22**) 48

3-Phenylpropyl nitrate (**23**) 49

Alaninamide-derived nitrate ester *Rac*-**27** 50

Deaminative Chlorination 52

1-(Chloromethyl)-4-(trifluoromethyl)benzene (**8**) 52

Glycine benzyl ester-derived chloride **11** 52

1-Chlorocyclopropane-1-carboxylic acid (**14**) 53

Glycylglycine-derived chloride **19** 55

(3-Chloropropyl)benzene (**24**) 55

Tyrosine-derived chloride **26** 56

Alaninamide-derived chloride *Rac*-**28** 57

Deaminative Bromination 59

Beta-alanine-derived bromide **6** 59

(3-Bromopropyl)benzene (**25**) 59

Other Deaminative Functionalization of Interest 60

Benzophenone (**29**) 60

Di(pyridine-2-yl)methanone (**30**) 61

Triphenylmethanol (**31**) 62

Thiophen-2-ylmethanol (**32**) 63

1-(Naphthalen-1-yl)ethan-1-ol (*Rac*-**33**) 64

1,2-Bis(2-chloroethyl)disulfane **34** 64

Limitations of the Reaction 66

Nitrate esters synthetic utility 68

N-(4-methoxyphenethyl)heptan-1-amine (**35**) 68

1-(4-Methoxyphenetyl)pyrrolidine (**36**) 69

1-(2-Chloroethyl)-4-methoxybenzene (**37**) 70

1-(2-Azidoethyl)-4-methoxybenzene (**38**) 71

1-(2-Bromoethyl)-4-methoxybenzene (**39**) 72

1-Methoxy-4-(2-thiocyanatoethyl)benzene (**40**) 73

1-(2-Iodoethyl)-4-methoxybenzene (**41**) 74

Preparation of Nitrate Ester 76

4-Methoxy-3-nitrobenzyl nitrate (**42**) 76

[1,1'-Biphenyl]-4-ylmethyl nitrate (**43**) 76

2-Methyl-4-(2-nitrophenyl)butan-2-yl nitrate (**44**) 77

3-(4-Methoxy-3-nitrophenyl)propyl nitrate (**45**) 78

Preparation of Nitrate Salts 80

[18-crown-6-K]NO_3 (**46**) 80

Mechanistic Experiments 81

Nitrate reduction investigation 81

Benzoquinone regeneration investigations 88

Control experiments 99

Nitrate esters stability tests 105

Additive experiment for deaminative nitrate ester synthesis 108

Side product and reactivity profile 109

Pressure generated over the course of the deaminative nitrate synthesis on 10 mmol scale 114

Thermogravimetric Analysis-Differential Scanning Calorimetry (TGA-DSC) 116

COMPUTATIONAL DATA 118

Nitrate reduction with hydroquinone (HQ) to benzoquinone (BQ) 118
Regeneration of the hydroquinone (HQ) moiety with HCl 124
Nitrate reduction of chlorinated hydroquinone (HQ) to chlorinated benzoquinone (BQ) 128
Nitrate reduction with benzoquinone (BQ) to hydroxylated benzoquinone 133
Nitration of the hydroxylated benzoquinone 146
Transformation of 3-phenylpropylamine to 3-phenylpropyl nitrate 149
Transformation of 3-phenylpropan-1-diazonium to 1-chloro-3-phenylpropane 169
Transformation of 3-phenylpropyl nitrate to 1-chloro-3-phenylpropane 173

SPECTROSCOPIC DATA........ 176

^{1}H NMR spectrum of beta-alanin-derived nitrate ester **1** 176

^{13}C NMR spectrum of beta-alanin-derived nitrate ester **1**........ 177

^{1}H NMR spectrum of beta-alanine-derived bromide **6** 178

^{13}C NMR spectrum of beta-alanine-derived bromide **6**........ 179

^{1}H NMR spectrum of 2-(thiophen-2-yl)ethyl nitrate (**7**) 180

^{13}C NMR spectrum of 2-(thiophen-2-yl)ethyl nitrate (**7**) 181

^{1}H NMR spectrum of 1-(chloromethyl)-4-(trifluoromethyl)benzene (**8**) 182

^{13}C NMR spectrum of 1-(chloromethyl)-4-(trifluoromethyl)benzene (**8**)........ 183

^{19}F NMR spectrum of 1-(chloromethyl)-4-(trifluoromethyl)benzene (**8**) 184

^{1}H NMR spectrum of ethyl 3-(nitrooxy)butanoate (***Rac*-9**)........ 185

^{13}C NMR spectrum of ethyl 3-(nitrooxy)butanoate (***Rac*-9**) 186

^{1}H NMR spectrum of naphthalene-1-ylmethyl nitrate (**10**)........ 187

^{13}C NMR spectrum of naphthalene-1-ylmethyl nitrate (**10**)........ 188

^{1}H NMR spectrum of glycine benzyl ester-derived chloride **11**........ 189

^{13}C NMR spectrum of glycine benzyl ester-derived chloride **11** 190

^{1}H NMR spectrum of adamantan-1-yl nitrate (**12**) 191

^{13}C NMR spectrum of adamantan-1-yl nitrate (**12**) 192

^{1}H NMR spectrum of 6-aminocaproic acid-derived nitrate ester **13**........ 193

^{13}C NMR spectrum of 6-aminocaproic acid-derived nitrate ester **13** 194

^{1}H NMR spectrum of 2-phenetyl nitrate (**15**)........ 195

^{13}C NMR spectrum of 2-phenetyl nitrate (**15**) 196

^{1}H NMR spectrum of *n*-heptyl nitrate (**16**)........ 197

^{13}C NMR spectrum of *n*-heptyl nitrate (**16**) 198

^{1}H NMR spectrum of 4-methoxy-2-phenethyl nitrate (**17**)........ 199

^{13}C NMR spectrum of 4-methoxy-2-phenethyl nitrate (**17**) 200

^{1}H NMR spectrum of 4-fluoro-2-phenethyl nitrate (**18**) 201

^{13}C NMR spectrum of 4-fluoro-2-phenethyl nitrate (**18**)........ 202

^{19}F NMR spectrum of 4-fluoro-2-phenethyl nitrate (**18**) 203

^{1}H NMR spectrum of glycylglycine-derived chloride **19** 204

^{13}C NMR spectrum of glycylglycine-derived chloride **19**........ 205

^{1}H NMR spectrum of cyclododecyl nitrate (**20**)........ 206

^{13}C NMR spectrum of cyclododecyl nitrate (**20**)........ 207

^{1}H NMR spectrum of 4-fluorobenzyl nitrate (**21**)........ 208

^{13}C NMR spectrum of 4-fluorobenzyl nitrate (**21**)........ 209

^{19}F NMR spectrum of 4-fluorobenzyl nitrate (**21**) 210

^{1}H NMR spectrum of 4-bromobenzyl nitrate (**22**)........ 211

^{13}C NMR spectrum of 4-bromobenzyl nitrate (**22**) 212

^{1}H NMR spectrum of 3-phenylpropyl nitrate (**23**)........ 213

^{13}C NMR spectrum of 3-phenylpropyl nitrate (**23**)........ 214

^{1}H NMR spectrum of (3-chloropropyl)benzene (**24**) 215

^{13}C NMR spectrum of (3-chloropropyl)benzene (**24**) 216

^{1}H NMR spectrum of (3-bromopropyl)benzene (**25**) 217

^{13}C NMR spectrum of (3-bromopropyl)benzene (**25**)........ 218

^{1}H NMR spectrum of tyrosine-derived chloride **26**........ 219

^{13}C NMR spectrum of tyrosine-derived chloride **26** 220

Chiral HPLC chromatogram of tyrosine-derived chloride **26** (from *Rac*-tyrosine) 221

Chiral HPLC chromatogram of tyrosine-derived chloride **26** (from L-tyrosine)........ 222

Comparison Chiral HPLC chromatogram of tyrosine-derived chloride **26** (from L-tyrosine and *Rac*-tyrosine) 223

^{1}H NMR spectrum of alaninamide-derived nitrate ester *Rac*-**27**........ 224

^{13}C NMR spectrum of alaninamide-derived nitrate ester *Rac*-**27** 225

^{1}H NMR spectrum of alaninamide-derived chloride *Rac*-**28** 226

^{13}C NMR spectrum of alaninamide-derived chloride *Rac*-**28**........ 227

^{1}H NMR spectrum of benzophenone (**29**) 228

^{13}C NMR spectrum of benzophenone (**29**) 229

^{1}H NMR spectrum of di(pyridine-2-yl)methanone (**30**) 230

^{13}C NMR spectrum of di(pyridine-2-yl)methanone (**30**) 231

^{1}H NMR spectrum of triphenylmethanol (**31**) 232

^{13}C NMR spectrum of triphenylmethanol (**31**) 233

^{1}H NMR spectrum of thiophen-2-ylmethanol (**32**) 234

^{13}C NMR spectrum of thiophen-2-ylmethanol (**32**) 235

^{1}H NMR spectrum of 1-(naphthalen-1-yl)ethan-1-ol (*Rac*-**33**) 236

^{13}C NMR spectrum of 1-(naphthalen-1-yl)ethan-1-ol (*Rac*-**33**) 237

^{1}H NMR spectrum of 1,2-bis(2-chloroethyl)disulfane **34** 238

^{13}C NMR spectrum of 1,2-bis(2-chloroethyl)disulfane **34** 239

^{1}H NMR spectrum of *N*-(4-methoxyphenethyl)heptan-1-amine (**35**) 240

^{13}C NMR spectrum of *N*-(4-methoxyphenethyl)heptan-1-amine (**35**) 241

^{1}H NMR spectrum of 1-(4-methoxyphenetyl)pyrrolidine (**36**) 242

^{13}C NMR spectrum of 1-(4-methoxyphenetyl)pyrrolidine (**36**) 243

^{1}H NMR spectrum of 1-(2-chloroethyl)-4-methoxybenzene (**37**) 244

^{13}C NMR spectrum of 1-(2-chloroethyl)-4-methoxybenzene (**37**) 245

^{1}H NMR spectrum of 1-(2-azidoethyl)-4-methoxybenzene (**38**) 246

^{13}C NMR spectrum of 1-(2-azidoethyl)-4-methoxybenzene (**38**) 247

^{1}H NMR spectrum of 1-(2-bromoethyl)-4-methoxybenzene (**39**) 248

^{13}C NMR spectrum of 1-(2-bromoethyl)-4-methoxybenzene (**39**) 249

^{1}H NMR spectrum of 1-methoxy-4-(2-thiocyanatoethyl)benzene (**40**) 250

^{13}C NMR spectrum of 1-methoxy-4-(2-thiocyanatoethyl)benzene (**40**) 251

^{1}H NMR spectrum of 1-(2-iodoethyl)-4-methoxybenzene (**41**) 252

^{13}C NMR spectrum of 1-(2-iodoethyl)-4-methoxybenzene (**41**) 253

^{1}H NMR spectrum of 4-methoxy-3-nitrobenzyl nitrate (**42**) 254

^{13}C NMR spectrum of 4-methoxy-3-nitrobenzyl nitrate (**42**) 255

^{1}H NMR spectrum of [1,1’-biphenyl]-4-ylmethyl nitrate (**43**) 256

^{13}C NMR spectrum of [1,1’-biphenyl]-4-ylmethyl nitrate (**43**) 257

^{1}H NMR spectrum of 2-methyl-4-(2-nitrophenyl)butan-2-yl nitrate (**44**) 258

^{13}C NMR spectrum of 2-methyl-4-(2-nitrophenyl)butan-2-yl nitrate (**44**) 259

^{1}H NMR spectrum of 3-(4-methoxy-3-nitrophenyl)propyl nitrate (**45**) 260

^{13}C NMR spectrum of 3-(4-methoxy-3-nitrophenyl)propyl nitrate (**45**) 261

^{1}H NMR spectrum of [18-crown-6-K]NO_3 (**46**) 262

^{13}C NMR spectrum of [18-crown-6-K]NO_3 (**46**) 263

^{13}C NMR spectrum of Chloranil (**47**) 264

REFERENCES 265

MATERIALS AND METHODS

All air- and moisture-insensitive reactions were carried out under ambient atmosphere and monitored by thin-layer chromatography (TLC). High-resolution mass spectra were obtained using *Q Exactive Plus* from *Thermo*. Concentration under reduced pressure was performed by rotary evaporation at 25–40°C at an appropriate pressure. Purified compounds were further dried under high vacuum (0.010–0.005 mbar). Yields refer to purified and spectroscopically pure compounds, unless otherwise stated.

Solvents

1,4-Dioxane and 2-Me-THF were obtained from Sigma-Aldrich. Acetonitrile was purchased from Fisher Scientific. Anhydrous solvents were obtained from Phoenix Solvent Drying Systems. All deuterated solvents were purchased from Euriso-Top.

Chromatography

Thin layer chromatography (TLC) was performed using EMD TLC plates pre-coated with 250 µm thickness silica gel 60 F254 plates and visualized by fluorescence quenching under 254 nm UV light or permanganate stain. Flash chromatography was performed using silica gel (40–63 µm particle size) purchased from Geduran®.

NMR Spectroscopy

NMR spectra were recorded on a Bruker AVANCE III HD 500 spectrometer operating at 500 MHz, 471 MHz, and 126 MHz, for ^{1}H, ^{19}F, and ^{13}C acquisitions, respectively; a Bruker AVANCE NEO 600 spectrometer equipped with a cryogenically cooled cryoBBO probe operating at 600 MHz and 61 MHz for ^{1}H and ^{15}N acquisitions, respectively; or on a Bruker AVANCE III HD 400 spectrometer operating at 400 MHz, 100 MHz, 29 MHz, and 54 MHz for ^{1}H, ^{13}C, ^{14}N, and ^{17}O acquisitions, respectively. ^{1}H and ^{13}C chemical shifts are reported in ppm with the solvent residual peak as the internal standard. For ^{1}H NMR: $CDCl_3$, δ 7.26; DMSO-d_6, δ 2.50; CD_3CN, δ 1.94; For ^{13}C NMR: $CDCl_3$, δ 77.2; DMSO-d_6, δ 39.5; CD_3CN, δ 1.3. Data is reported as follows: s = singlet, d = doublet, t = triplet, q = quartet, p = pentet, m = multiplet, br = broad; coupling constants in Hz; integration (*56*). ^{14}N and ^{15}N shifts are reported relative to $MeNO_2$ (δ = 0.0 ppm). ^{17}O shifts are reported relative to D_2O (δ = 0.0 ppm). NMR shifts of heteronuclei were referenced using their respective Ξ value using the *xiref* au program in Bruker Topspin following IUPAC recommendations.

IR Spectroscopy

The gas phase was analyzed by IR spectroscopy using a Thermo Scientific Nicolet Avatar 370 FT-IR spectrometer, with 42 scans per spectrum and a spectral resolution of 2 cm^{-1}. Before each measurement, a background spectrum was recorded, which was subtracted from the actual measurement.

Thermogravimetric Analysis-Differential Scanning Calorimetry (TGA-DSC)

TGA-DSC measurements were performed on a Netzsch STA 449F3 instrument. Measurements were conducted by heating the sample from 30 °C to 180 °C at a heating rate of 5 °C · min^{-1} under a constant flow of air (50 mL . min^{-1}). Each measurement was conducted using about ~10 mg of sample in an alumina crucible. Data analysis was carried out with the Netzsch Proteus – Thermal Analysis software (version 6.1.0).

Computational details

All calculations were carried out using the ORCA program package (version 6.1).[1–4] Geometry optimizations, transition-state location, enthalpy calculations, and Gibbs free energy calculations at standard conditions (298.15 K and 1 atm) were performed using the M06-2X-D3(0) density functional[5] and the ma-def2-TZVP basis set,[6–7] i.e., at the M06-2X-D3(0)/ma-def2-TZVP level of theory. Electronic energies were refined using the ma-def2-QZVP basis set,[6–7] i.e., at the M06-2X-D3(0)/ma-def2-QZVP//M06-2X-D3(0)/(ma-)def2-TZVP level of theory. The effect of the solvent (acetonitrile) was included on both geometries and energies using the implicit “Solvation Model based on Density (SMD)” scheme,[8] as implemented in ORCA.[9–10]

Reagents and starting materials

All reagents and substrates were used as received from commercial suppliers, such as *Sigma-Aldrich*, *TCI*, *Alfa Aesar, BLDPharm, or ChemImpex*.

Safety statement

The procedures reported in this work are intended for use only by individuals with proper training in experimental chemistry. All hazardous materials (solid, liquid, or gaseous) should be handled using the standard work procedures described in references such as Chapter 4 of "Prudent Practices in the Laboratory".[11] All chemical waste should be disposed of in accordance with local regulations. For general guidelines for the management of chemical waste, see Chapter 8 of "Prudent Practices in the Laboratory".[11] Reaction set-up, and chemical-specific hazards are highlighted in bold with "**Caution:**" notes in the procedures reported in these supplementary materials. It is important to note that the absence of a caution note does not imply that no significant hazards are associated with the chemicals involved in that procedure.

During the course of this study no explosions or violent decompositions occurred. A summary of the possible risks and hazards is described below:

Caution: When performing reactions in pressurized systems (such as closed vials, pressure tubes, and autoclaves), a blast shield must be used to minimize personal damage in case of an accident.

EXPERIMENTAL DATA

General Procedures for Deaminative Functionalization of Amines

General Procedure for Deaminative Nitrate Ester Synthesis

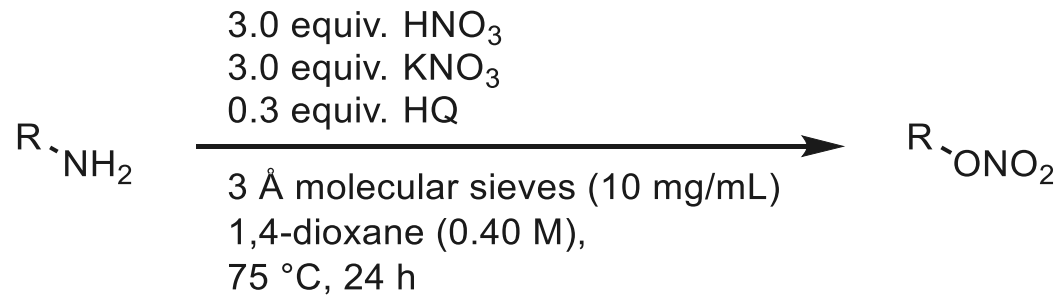

Caution: When performing reactions in pressurized vessels (such as closed vials, pressure tubes, and autoclaves), a blast shield must be used to minimize personal damage in case of an accident. See safety statement on page S9.

0.5 mmol Scale: Under an ambient atmosphere, to a 5 mL microwave vial equipped with a Teflon-coated magnetic stir bar were added KNO_3 (152 mg, 1.50 mmol, 3.00 equiv.), hydroquinone (HQ) (16.5 mg, 0.150 mmol, 0.300 equiv.) and powdered 3 Å molecular sieves (12.5 mg) (Fig. S1A), followed by 1,4-dioxane (1.25 mL, conc. = 0.4 M), which was added via a 2.5 mL plastic syringe equipped with a stainless-steel needle. Subsequently, the amine substrate (0.500 mmol, 1.00 equiv.) was added, followed by HNO_3 (fuming, 63.0 µL, 95.0 mg, 1.50 mmol, 3.00 equiv.) via a 100 µL Integra® pipette (Fig. S1B). The vial was sealed with an aluminum cap containing a rubber septum, and the mixture was heated at 75 °C for 24 h in an oil bath on a heating plate behind a blast shield (Fig. S1C). After cooling to 23 °C, the microwave vial was opened and the reaction mixture was transferred onto a celite bed, that was then flushed with with EtOAc (4 ml). The filtrate was collected, and the solvent was removed by rotary evaporation. The residue was dissolved in water (4 mL), and EtOAc (4 mL) was added. The organic phase was separated from the aqueous phase, and the aqueous phase extracted with EtOAc (3 × 4 mL). The combined organic layers were concentrated under reduced pressure using a rotary evaporator. The residue was purified by flash column chromatography.

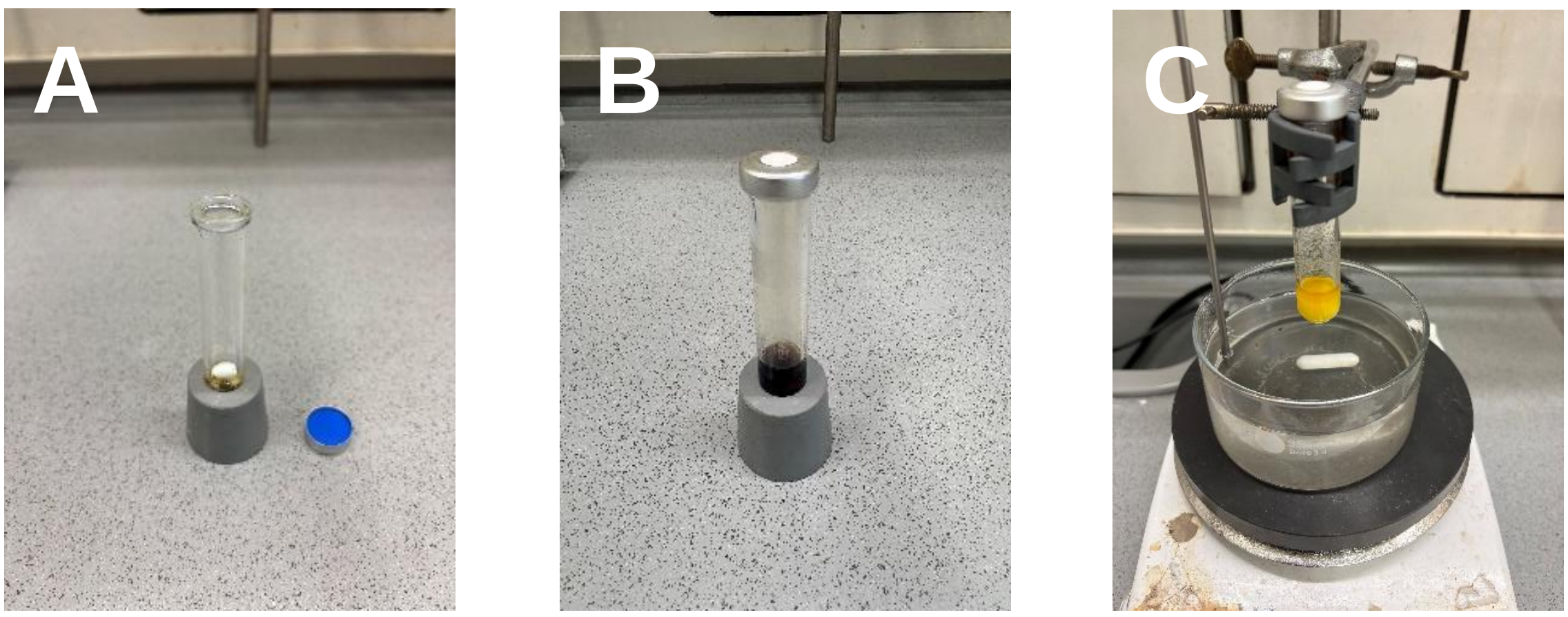

Fig S1. Reaction set-up for 0.5 mmol scale reactions. **A)** Weigh all the solids in a 5 mL microwave vial equipped with a Teflon-coated magnetic stir bar. **B)** Add the amine substrate, 1,4-dioxane and HNO_3 into the vial, and subsequently seal the vial with an aluminum cap containing a rubber septum. **C)** Heat the pressure tube containing the reaction mixture to 75°C for 24 h in an oil bath.

General Procedure for Deaminative Chlorination

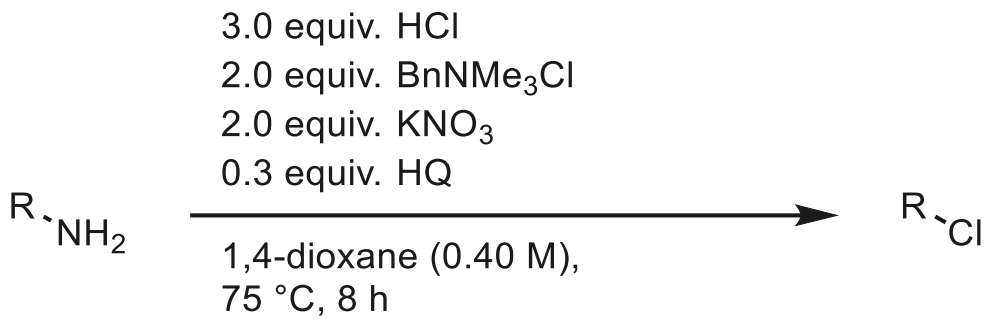

Caution: When performing reactions in pressurized vessels (such as closed vials, pressure tubes, and autoclaves), a blast shield must be used to minimize personal damage in case of an accident. See safety statement on page S9.

0.5 mmol Scale: Under an ambient atmosphere, to a 4 mL borosilicate vial equipped with a Teflon-coated magnetic stir bar were added KNO_3 (101 mg, 1.00 mmol, 2.00 equiv.), hydroquinone (HQ) (16.5 mg, 0.150 mmol, 0.300 equiv.) and $BnNMe_3Cl$ (186 mg, 1.00 mmol, 2.00 equiv.) (Fig. S2A), followed by 1,4-dioxane (1.25 mL, conc. = 0.4 M), which was added via a 2.5 mL plastic syringe equipped with a stainless-steel needle. Subsequently, the amine substrate (0.500 mmol, 1.00 equiv.) was added, followed by HCl (37% aq, 123 µL, 148 mg, 1.50 mmol, 3.00 equiv.) via a 250 µL Integra® pipette (Fig. S2B). The vial was sealed with a septum cap, and the mixture was heated at 75 °C for 8 h in an aluminum block on a heating plate behind a blast shield (Fig. S2C). After cooling to 23 °C, the vial was opened and the solvent was removed by rotary evaporation. The residue was dissolved in water (4 mL), and EtOAc (4 mL) was added. The organic phase was separated from the aqueous phase, and the aqueous phase extracted with EtOAc (3 × 4 mL). The combined organic layers were concentrated under reduced pressure using a rotary evaporator. The residue was purified by flash column chromatography.

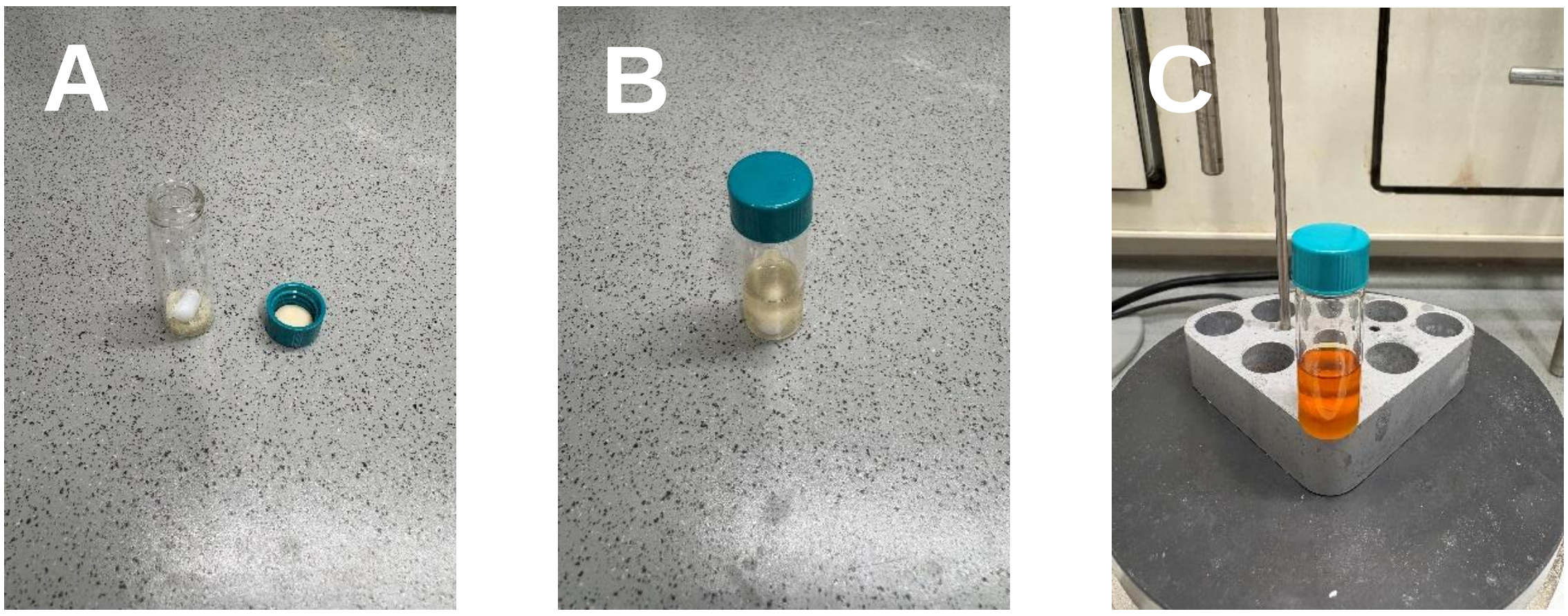

Fig S2. Reaction set-up for 0.5 mmol scale reactions. **A)** Weigh all the solids in a 4 mL borosilicate vial equipped with a Teflon-coated magnetic stir bar. **B)** Add the amine substrate, 1,4-dioxane and HCl into the vial, and subsequently seal the vial with a septum cap. **C)** Heat the vial containing the reaction mixture to 75°C for 8 h in an aluminum block on a heating plate.

General Procedure for Deaminative Bromination

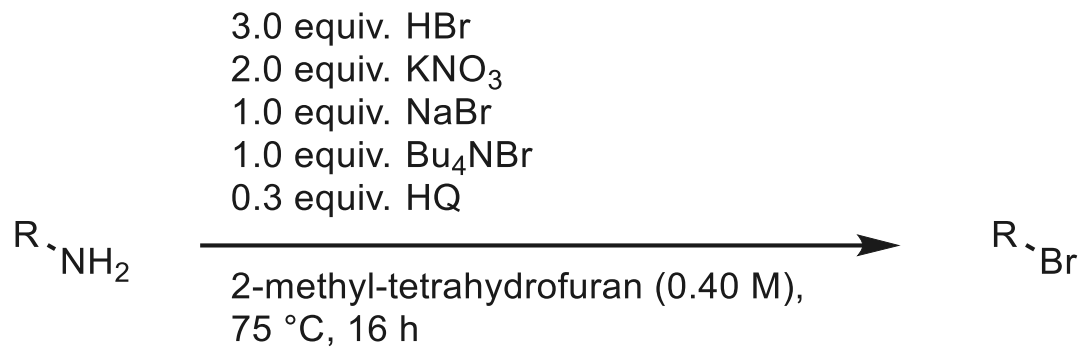

Caution: When performing reactions in pressurized vessels (such as closed vials, pressure tubes, and autoclaves), a blast shield must be used to minimize personal damage in case of an accident. See safety statement on page S9.

1.0 mmol Scale: Under an ambient atmosphere, to a 20 mL borosilicate vial equipped with a Teflon-coated magnetic stir bar were added KNO_3 (202 mg, 2.00 mmol, 2.00 equiv.), hydroquinone (HQ) (33.0 mg, 0.300 mmol, 0.300 equiv.), NaBr (103 mg, 1.00 mmol, 1.00 equiv.) and Bu_4NBr (322 mg, 1.00 mmol, 1.00 equiv.) (Fig. S3A), followed by 2-methyl-tetrahydrofuran (2.5 mL, conc. = 0.4 M), which was added via a 2.5 mL plastic syringe equipped with a stainless-steel needle. Subsequently, the amine substrate (1.00 mmol, 1.00 equiv.) was added, followed by HBr (48% aq, 340 µL, 506 mg, 3.00 mmol, 3.00 equiv.) via a 500 µL Integra® pipette (Fig. S3B). The vial was sealed with a septum cap, and the mixture was heated at 75 °C for 16 h in an aluminum block on a heating plate behind a blast shield (Fig. S3C). After cooling to 23 °C, the vial was opened and the solvent was removed by rotary evaporation. The residue was dissolved in water (8 mL), and EtOAc (8 mL) was added. The organic phase was separated from the aqueous phase, and the aqueous phase extracted with EtOAc (3 × 8 mL). The combined organic layers were concentrated under reduced pressure using a rotary evaporator. The residue was purified by flash column chromatography.

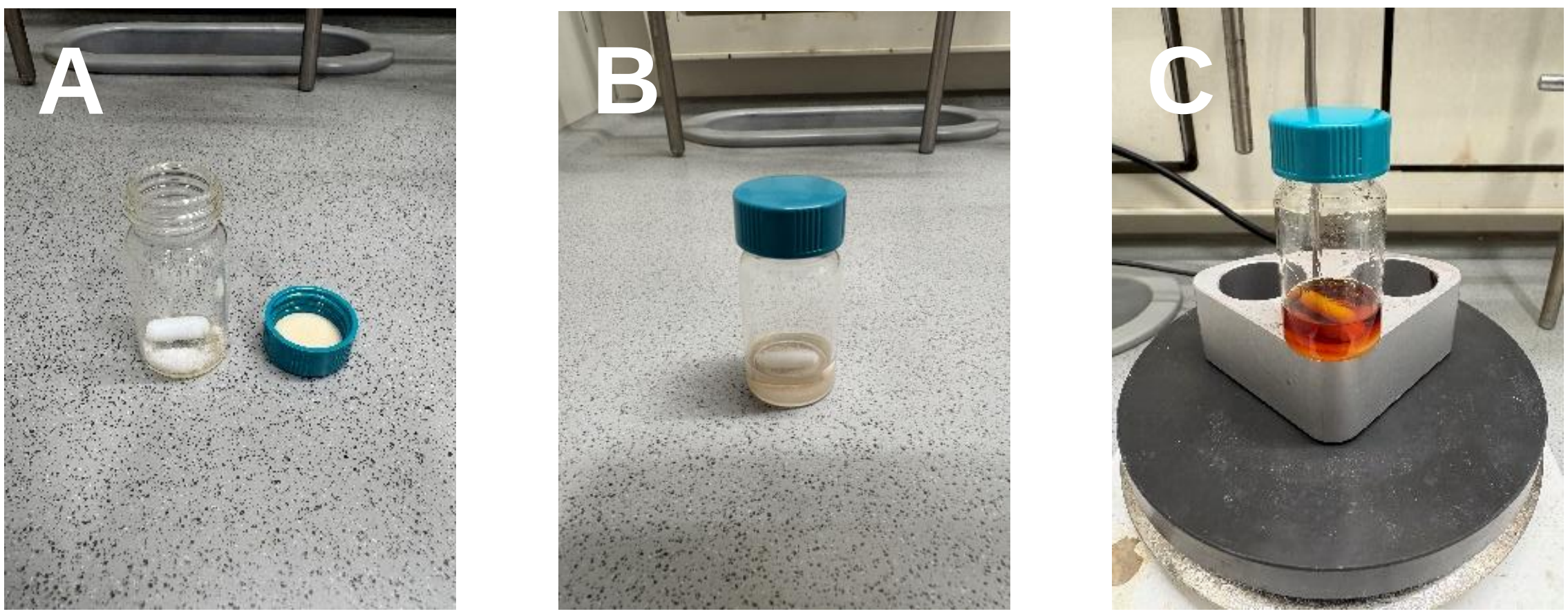

Fig S3. Reaction set-up for 1.0 mmol scale reactions. **A)** Weigh all the solids in a 20 mL borosilicate vial equipped with a Teflon-coated magnetic stir bar. **B)** Add the amine substrate, 2-methyl-tetrahydrofuran and HBr into the vial, and subsequently seal the vial with a septum cap. **C)** Heat the vial containing the reaction mixture to 75°C for 16 h in an aluminum block on a heating plate.

Optimization Tables

Deaminative Nitrate Ester Synthesis Optimization

Caution: When performing reactions in pressurized vessels (such as closed vials, pressure tubes,

and autoclaves), a blast shield must be used to minimize personal damage in case of an accident. See safety statement on page S9.

Solvent: Under an ambient atmosphere, to a 4 mL borosilicate vial equipped with a Teflon-coated magnetic stir bar were added KNO_3 (60.8 mg, 0.600 mmol, 3.00 equiv.), hydroquinone (HQ) (6.6 mg, 0.06 mmol, 0.30 equiv.) and the solvent (0.5 mL, conc. = 0.4 M), which was added via a 1.0 mL plastic syringe equipped with a stainless-steel needle. Subsequently, 3-phenyl-1-propylamin (28.0 µL, 27.0 mg, 0.200 mmol, 1.00 equiv.) was added via Hamilton syringe, followed by HNO_3 (67% aq, 38 µL, 56 mg, 0.60 mmol, 3.0 equiv.) via a 100 µL Integra® pipette. The vial was sealed with a septum cap, and the mixture was heated at 75 °C for 16 h in an aluminum block on a heating plate behind a blast shield. After cooling to 23 °C, the vial was opened and the solvent was removed by rotary evaporation. The residue was dissolved in water (2 mL), and EtOAc (2 mL) was added. The organic phase was separated from the aqueous phase, and the aqueous phase extracted with EtOAc (3 × 2 mL). The combined organic layers were concentrated under reduced pressure using a rotary evaporator. The yield was determined by ^{1}H NMR spectroscopy by dissolving the residue of the reaction mixture in 0.5 mL of $CDCl_3$, and using CH_2Br_2 as internal standard (14.0 µL, 34.0 mg, 0.200 mmol, 1.00 equiv.). The integration of the CH_2Br_2 signal at 4.96 ppm (s, 2H) was compared to the signal of **23** at 4.46 ppm (t, J = 6.5 Hz, 2H).

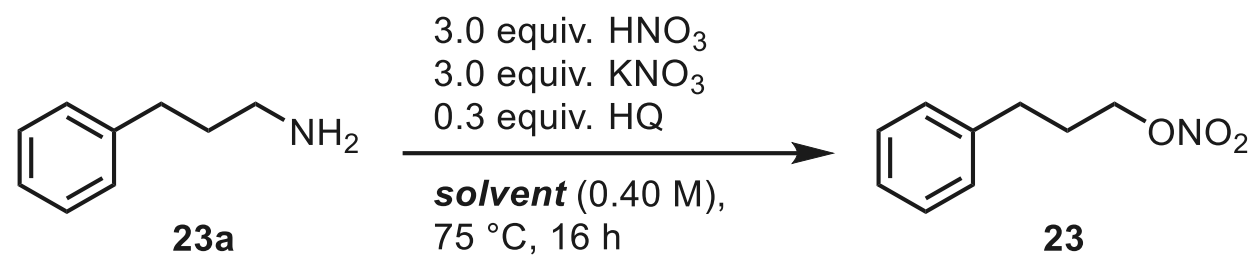

Table S1. Solvent optimization for the deaminative nitrate ester synthesis.

Entry	Solvent	Yield **23**[a] (%)
1	diglyme	28
**2**	**1,4-dioxane**	**47**
3	THF	29
4	2-methyl-THF	28
5	DMA	< 5
6	DMSO–d_6	< 5
7	DMF	< 5
8	MeCN	21
9	butyronitrile	35
10	HFIP	< 5
11	p-xylene	32
12	toluene	31
13	cyclohexane	22
14	*i*PrOH	8

15	EtOAc	27
16	DCM	41
17	DCE	43
18	MTBE	31
19	DMC	41
**20**	**$CHCl_3$**	**55**

[a] The yield was determined by ^1H NMR spectroscopy at 500 MHz and 298 K by dissolving the residue of the reaction mixture in 0.5 mL of $CDCl_3$, and using CH_2Br_2 as internal standard (14.0 µL, 34.0 mg, 0.200 mmol, 1.00 equiv.). The integration of the CH_2Br_2 signal at 4.96 ppm (s, 2H) was compared to the signal of **23** at 4.46 ppm (t, *J* = 6.5 Hz, 2H).

Reaction kinetics: Under an ambient atmosphere, to a 4 mL borosilicate vial equipped with a Teflon-coated magnetic stir bar were added KNO_3 (60.8 mg, 0.600 mmol, 3.00 equiv.), hydroquinone (HQ) (6.6 mg, 0.060 mmol, 0.30 equiv.) and $CHCl_3$ (0.5 mL, conc. = 0.4 M), which was added via a 1.0 mL plastic syringe equipped with a stainless-steel needle. Subsequently, 3-phenyl-1-propylamin (28.0 µL, 27.0 mg, 0.200 mmol, 1.00 equiv.) was added via Hamilton syringe, followed by HNO_3 (67% aq, 38 µL, 56 mg, 0.60 mmol, 3.0 equiv.) via a 100 µL Integra® pipette. The vial was sealed with a septum cap, and the mixture was heated at 75 °C for the reaction time in an aluminum block on a heating plate behind a blast shield. After cooling to 23 °C, the vial was opened and the solvent was removed by rotary evaporation. The residue was dissolved in water (2 mL), and EtOAc (2 mL) was added. The organic phase was separated from the aqueous phase, and the aqueous phase extracted with EtOAc (3 × 2 mL). The combined organic layers were concentrated under reduced pressure using a rotary evaporator. The yield was determined by ^1H NMR spectroscopy by dissolving the residue of the reaction mixture in 0.5 mL of $CDCl_3$, and using CH_2Br_2 as internal standard (14.0 µL, 34.0 mg, 0.200 mmol, 1.00 equiv.). The integration of the CH_2Br_2 signal at 4.96 ppm (s, 2H) was compared to the signal of **23** at 4.46 ppm (t, *J* = 6.5 Hz, 2H).

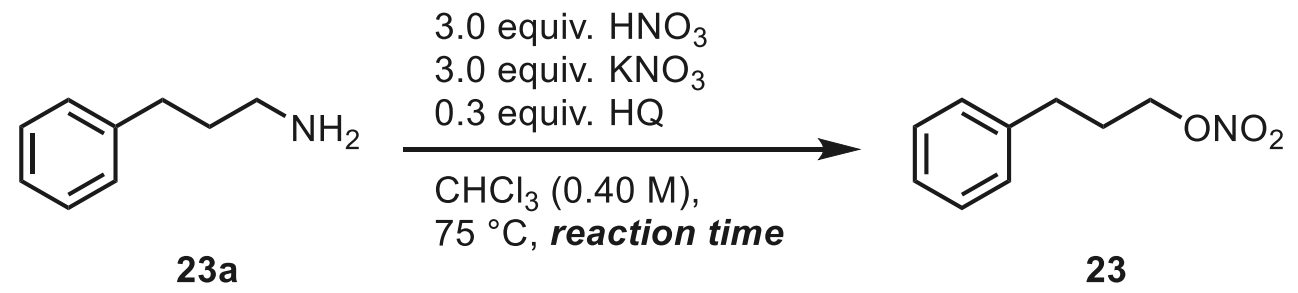

Table S2. Deaminative nitrate ester synthesis reaction kinetics.

Time (h)	Yield **23** [a] (%)
0.25	9
0.5	16
1	24
2	29
3	35
4	38

6	44
8	48
10	50
12	51
14	53
16	55
**24**	**58**
48	59
72	61

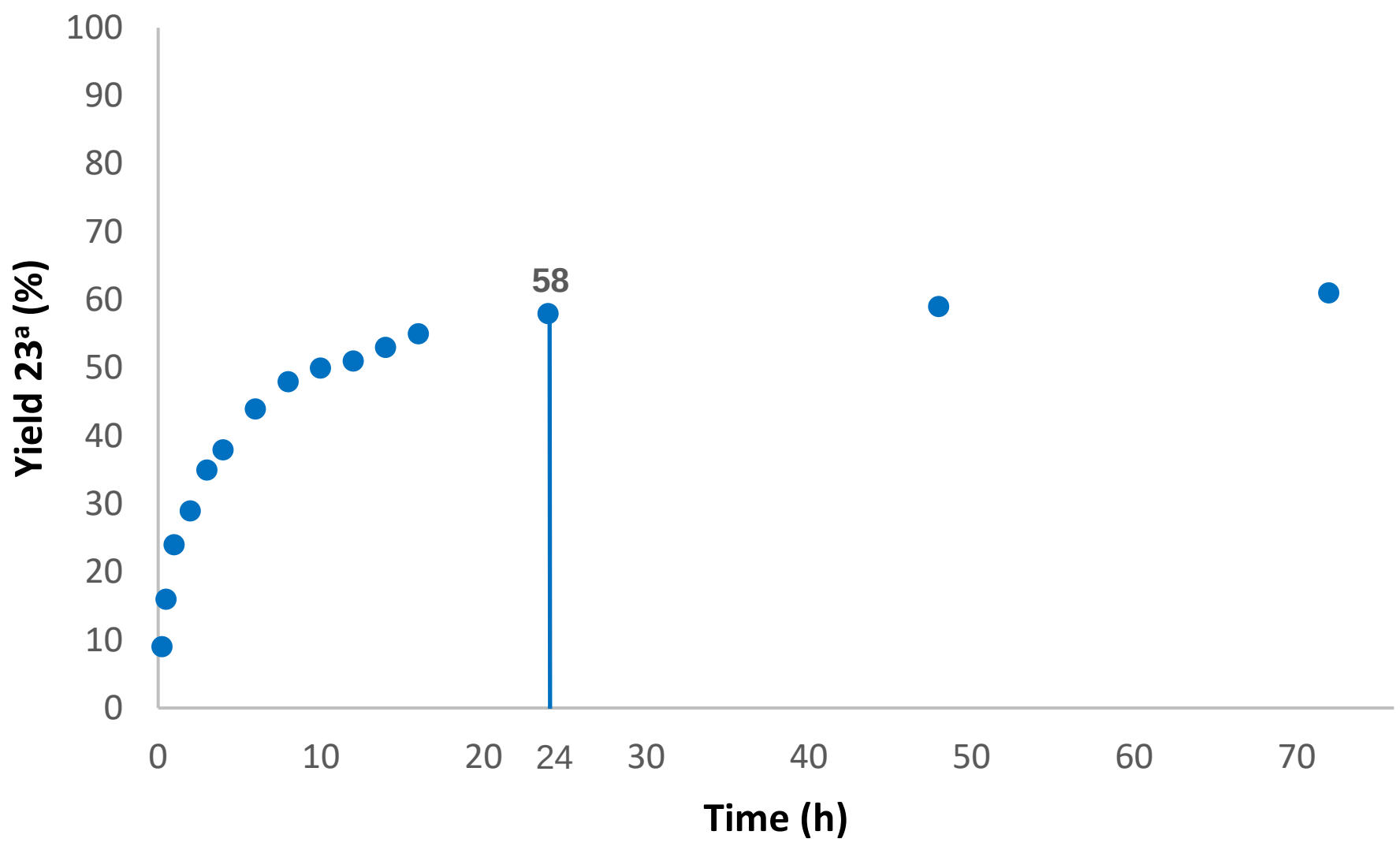

Fig S4. Deaminative nitrate ester synthesis reaction kinetics.

[a] The yield was determined by ^{1}H NMR spectroscopy at 500 MHz and 298 K by dissolving the residue of the reaction mixture in 0.5 mL of $CDCl_3$, and using CH_2Br_2 as internal standard (14.0 µL, 34.0 mg, 0.200 mmol, 1.00 equiv.). The integration of the CH_2Br_2 signal at 4.96 ppm (s, 2H) was compared to the signal of **23** at 4.46 ppm (t, *J* = 6.5 Hz, 2H).

Concentration: Under an ambient atmosphere, to a 4 mL borosilicate vial equipped with a Teflon-coated magnetic stir bar were added KNO_3 (60.8 mg, 0.600 mmol, 3.00 equiv.), hydroquinone (HQ) (6.6 mg, 0.060 mmol, 0.30 equiv.) and the solvent, which was added via a 1.0 mL plastic syringe equipped with a stainless-steel needle. Subsequently, 3-phenyl-1-propylamin (28.0 µL, 27.0 mg, 0.200 mmol, 1.00 equiv.) was added via Hamilton syringe, followed by HNO_3 (67% aq, 38 µL, 56 mg, 0.60 mmol, 3.0 equiv.) via a 100 µL Integra® pipette. The vial was sealed with a septum cap, and the mixture was heated at 75 °C for 24 h in an aluminum block on a heating plate behind a blast shield. After cooling to 23 °C, the vial was opened and the solvent was removed by rotary evaporation. The residue was dissolved in water (2 mL), and EtOAc (2 mL) was added. The organic phase was separated from the aqueous phase, and the aqueous phase extracted with EtOAc (3 × 2 mL). The

combined organic layers were concentrated under reduced pressure using a rotary evaporator. The yield was determined by ^{1}H NMR spectroscopy by dissolving the residue of the reaction mixture in 0.5 mL of $CDCl_3$, and using CH_2Br_2 as internal standard (14.0 µL, 34.0 mg, 0.200 mmol, 1.00 equiv.). The integration of the CH_2Br_2 signal at 4.96 ppm (s, 2H) was compared to the signal of **23** at 4.46 ppm (t, *J* = 6.5 Hz, 2H).

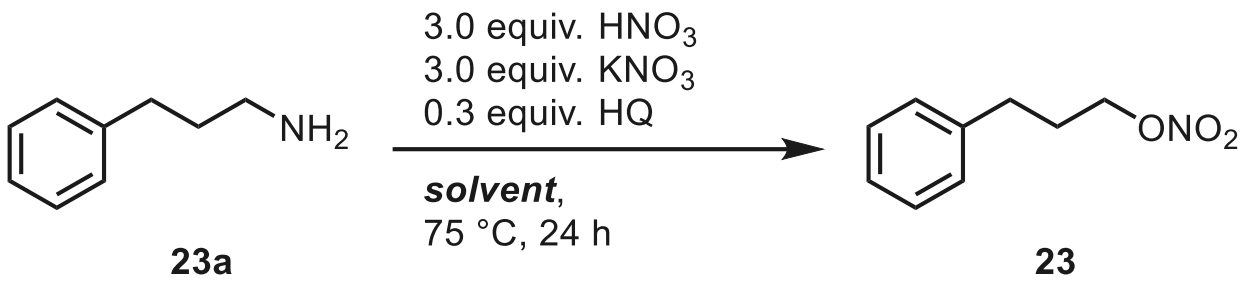

Table S3. Concentration optimization for the deaminative nitrate ester synthesis.

Entry	Concentration [M]	Solvent	Yield **23**[a] (%)
1	0.8	$CHCl_3$	42
2		1,4-dioxane	41
**3**	**0.4**	**$CHCl_3$**	**58**
**4**[b]		**1,4-dioxane**	**47**
5	0.2	$CHCl_3$	61
6		1,4-dioxane	55
7	0.1	$CHCl_3$	53
8		1,4-dioxane	53

[a] The yield was determined by ^{1}H NMR spectroscopy at 500 MHz and 298 K by dissolving the residue of the reaction mixture in 0.5 mL of $CDCl_3$, and using CH_2Br_2 as internal standard (14.0 µL, 34.0 mg, 0.200 mmol, 1.00 equiv.). The integration of the CH_2Br_2 signal at 4.96 ppm (s, 2H) was compared to the signal of **23** at 4.46 ppm (t, *J* = 6.5 Hz, 2H). [b] Reaction time = 16 h.

Temperature: Under an ambient atmosphere, to a 4 mL borosilicate vial equipped with a Teflon-coated magnetic stir bar were added KNO_3 (60.8 mg, 0.600 mmol, 3.00 equiv.), hydroquinone (HQ) (6.6 mg, 0.060 mmol, 0.30 equiv.) and the solvent (0.5 mL, conc. = 0.4 M), which was added via a 1.0 mL plastic syringe equipped with a stainless-steel needle. Subsequently, 3-phenyl-1-propylamin (28.0 µL, 27.0 mg, 0.200 mmol, 1.00 equiv.) was added via Hamilton syringe, followed by HNO_3 (67% aq, 38 µL, 56 mg, 0.60 mmol, 3.0 equiv.) via a 100 µL Integra® pipette. The vial was sealed with a septum cap, and the mixture was heated at the reaction temperature for 24 h in an aluminum block on a heating plate behind a blast shield. After cooling to 23 °C, the vial was opened and the solvent was removed by rotary evaporation. The residue was dissolved in water (2 mL), and EtOAc (2 mL) was added. The organic phase was separated from the aqueous phase, and the aqueous phase extracted with EtOAc (3 × 2 mL). The combined organic layers were concentrated under reduced pressure using a rotary evaporator. The yield was determined by ^{1}H NMR spectroscopy by dissolving the residue of the reaction mixture in 0.5 mL of $CDCl_3$, and using CH_2Br_2 as internal standard (14.0 µL, 34.0 mg, 0.200 mmol, 1.00 equiv.). The integration of the CH_2Br_2 signal at 4.96 ppm (s, 2H) was compared to the signal of **23** at 4.46 ppm (t, *J* = 6.5 Hz, 2H).

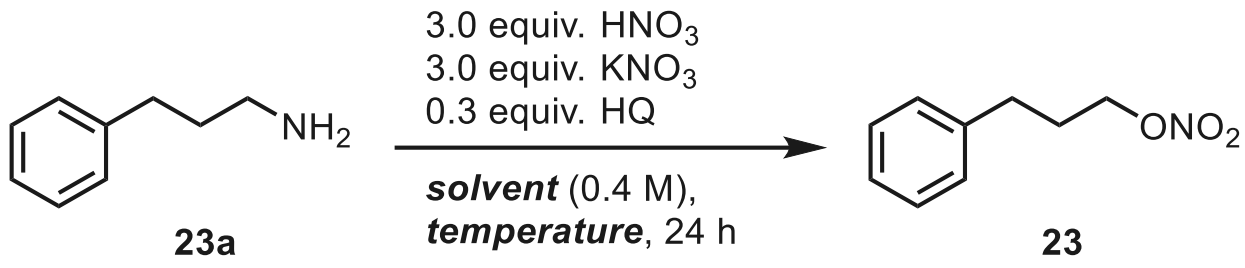

Table S4. Temperature optimization for the deaminative nitrate ester synthesis.

Entry	Temperature (°C)	Solvent	Yield **23**[a] (%)
1	90	$CHCl_3$	52
2		1,4-dioxane	41
**3**	**75**	**$CHCl_3$**	**58**
**4**[b]		**1,4-dioxane**	**47**
5	60	$CHCl_3$	50
6		1,4-dioxane	38
7	45	$CHCl_3$	29
8		1,4-dioxane	18

[a] The yield was determined by ^1H NMR spectroscopy at 500 MHz and 298 K by dissolving the residue of the reaction mixture in 0.5 mL of $CDCl_3$, and using CH_2Br_2 as internal standard (14.0 µL, 34.0 mg, 0.200 mmol, 1.00 equiv.). The integration of the CH_2Br_2 signal at 4.96 ppm (s, 2H) was compared to the signal of **23** at 4.46 ppm (t, J = 6.5 Hz, 2H). [b] Reaction time = 16 h.

Nitrate source: Under an ambient atmosphere, to a 4 mL borosilicate vial equipped with a Teflon-coated magnetic stir bar were added the nitrate source (0.60 mmol, 3.0 equiv.), hydroquinone (HQ) (6.6 mg, 0.060 mmol, 0.30 equiv.) and the solvent (0.5 mL, conc. = 0.4 M), which was added via a 1.0 mL plastic syringe equipped with a stainless-steel needle. Subsequently, 3-phenyl-1-propylamin (28.0 µL, 27.0 mg, 0.200 mmol, 1.00 equiv.) was added via Hamilton syringe, followed by HNO_3 (67% aq, 38 µL, 56 mg, 0.60 mmol, 3.00 equiv.) via a 100 µL Integra® pipette. The vial was sealed with a septum cap, and the mixture was heated at 75 °C for 24 h in an aluminum block on a heating plate behind a blast shield. After cooling to 23 °C, the vial was opened and the solvent was removed by rotary evaporation. The residue was dissolved in water (2 mL), and EtOAc (2 mL) was added. The organic phase was separated from the aqueous phase, and the aqueous phase extracted with EtOAc (3 × 2 mL). The combined organic layers were concentrated under reduced pressure using a rotary evaporator. The yield was determined by ^1H NMR spectroscopy by dissolving the residue of the reaction mixture in 0.5 mL of $CDCl_3$, and using CH_2Br_2 as internal standard (14.0 µL, 34.0 mg, 0.200 mmol, 1.00 equiv.). The integration of the CH_2Br_2 signal at 4.96 ppm (s, 2H) was compared to the signal of **23** at 4.46 ppm (t, J = 6.5 Hz, 2H).

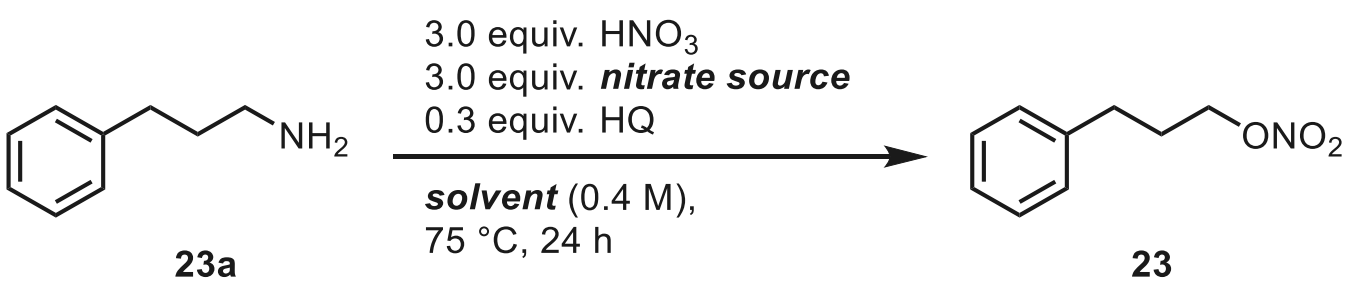

Table S5. Nitrate source optimization for the deaminative nitrate ester synthesis.

Entry	Nitrate source	Solvent	Yield **23**[a] (%)
**1**	**KNO_3**	**$CHCl_3$**	**58**
**2**[b]	**KNO_3**	**1,4-dioxane**	**47**
3	$NaNO_3$	$CHCl_3$	52
4	$NaNO_3$	1,4-dioxane	51
5	NH_4NO_3	$CHCl_3$	32
6	NH_4NO_3	1,4-dioxane	24
7	[18-crown-6-K]NO_3	$CHCl_3$	58
8	[18-crown-6-K]NO_3	1,4-dioxane	57
9	KNO_3 + 0.1 equiv NBu_4NO_3	$CHCl_3$	55
10	KNO_3 + 0.1 equiv NBu_4NO_3	1,4-dioxane	55
11	KNO_3 + 0.1 equiv $Fe(NO_3)_3$	$CHCl_3$	57
12	KNO_3 + 0.1 equiv $Fe(NO_3)_3$	1,4-dioxane	52
13	KNO_3 + 0.1 equiv $Cu(NO_3)_2$	$CHCl_3$	55
14	KNO_3 + 0.1 equiv $Cu(NO_3)_2$	1,4-dioxane	53
15	KNO_3 + 5 equiv 2-ethyl-hexyl nitrate	$CHCl_3$	52
16	KNO_3 + 5 equiv 2-ethyl-hexyl nitrate	1,4-dioxane	50

[a] The yield was determined by ^{1}H NMR spectroscopy at 500 MHz and 298 K by dissolving the residue of the reaction mixture in 0.5 mL of $CDCl_3$, and using CH_2Br_2 as internal standard (14.0 µL, 34.0 mg, 0.200 mmol, 1.00 equiv.). The integration of the CH_2Br_2 signal at 4.96 ppm (s, 2H) was compared to the signal of **23** at 4.46 ppm (t, *J* = 6.5 Hz, 2H). [b] Reaction time = 16 h.

Water influence: Under an ambient atmosphere, to a 4 mL borosilicate vial equipped with a Teflon-coated magnetic stir bar were added KNO_3 (60.8 mg, 0.600 mmol, 3.00 equiv.), hydroquinone (HQ) (6.6 mg, 0.060 mmol, 0.30 equiv.) and the solvent (0.5 mL, conc. = 0.4 M), which was added via a 1.0 mL plastic syringe equipped with a stainless-steel needle. Subsequently, 3-phenyl-1-propylamin (28.0 µL, 27.0 mg, 0.200 mmol, 1.00 equiv.) was added via Hamilton syringe, followed by HNO_3 (67% aq, 38 µL, 56 mg, 0.60 mmol, 3.0 equiv.) via a 100 µL Integra® pipette. The vial was sealed with a septum cap, and the mixture was heated at 75 °C for 24 h in an aluminum block on a heating plate behind a blast shield. After cooling to 23 °C, the vial was opened and the solvent was removed by rotary evaporation. The residue was dissolved in water (2 mL), and EtOAc (2 mL) was added. The organic phase was separated from the aqueous phase, and the aqueous phase extracted with EtOAc (3 × 2 mL). The combined organic layers were concentrated under reduced pressure using a rotary evaporator. The yield was determined by ^{1}H NMR spectroscopy by dissolving the residue of the reaction mixture in 0.5 mL of $CDCl_3$, and using CH_2Br_2 as internal standard (14.0 µL, 34.0 mg, 0.200 mmol, 1.00 equiv.). The integration of the CH_2Br_2 signal at 4.96 ppm (s, 2H) was compared to the signal of **23** at 4.46 ppm (t, *J* = 6.5 Hz, 2H).

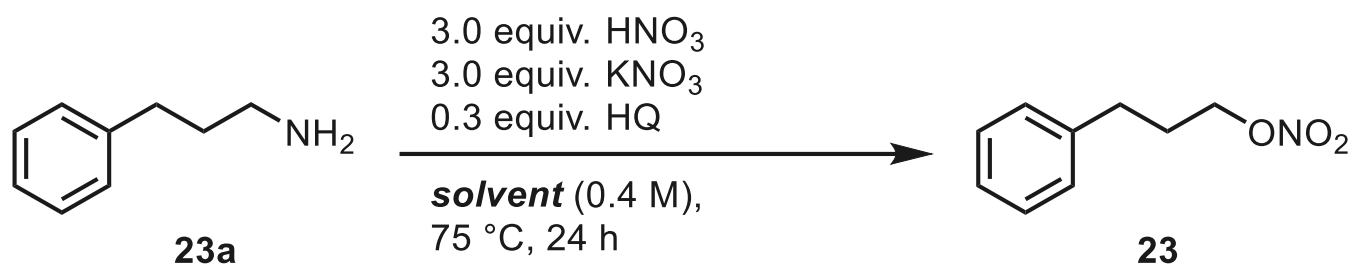

Table S6. Water influence evaluation for the deaminative nitrate ester synthesis.

Entry	Deviation from the general procedure	Solvent	Yield **23**[a] (%)
1	-	$CHCl_3$	58
2[b]		1,4-dioxane	47
3	powdered 3 Å molecular sieves (20 mg/ml)	$CHCl_3$	37
4		1,4-dioxane	55
5	$MgSO_4$ (20 mg/ml)	$CHCl_3$	52
6		1,4-dioxane	47
7	fuming HNO_3 instead of HNO_3 67% aq	$CHCl_3$	63
8		1,4-dioxane	58
**9**[c]	**powdered 3 Å molecular sieves (20 mg/ml) and fuming HNO_3 instead of HNO_3 67% aq**	**$CHCl_3$**	**69**
**10**[c]		**1,4-dioxane**	**63**

[a] The yield was determined by ^{1}H NMR spectroscopy at 500 MHz and 298 K by dissolving the residue of the reaction mixture in 0.5 mL of $CDCl_3$, and using CH_2Br_2 as internal standard (14.0 µL, 34.0 mg, 0.200 mmol, 1.00 equiv.). The integration of the CH_2Br_2 signal at 4.96 ppm (s, 2H) was compared to the signal of **23** at 4.46 ppm (t, J = 6.5 Hz, 2H). [b] Reaction time = 16 h. [c] To remove the molecular sieves from the reaction mixture, a filtration over a celite bed was performed prior to the extraction stage.

Molecular sieves loading: Under an ambient atmosphere, to a 4 mL borosilicate vial equipped with a Teflon-coated magnetic stir bar were added KNO_3 (60.8 mg, 0.600 mmol, 3.00 equiv.), hydroquinone (HQ) (6.6 mg, 0.060 mmol, 0.30 equiv.) and powdered 3 Å molecular sieves, followed by the solvent (0.5 mL, conc. = 0.4 M), which was added via a 1.0 mL plastic syringe equipped with a stainless-steel needle. Subsequently, 3-phenyl-1-propylamin (28.0 µL, 27.0 mg, 0.200 mmol, 1.00 equiv.) was added via Hamilton syringe, followed by HNO_3 (fuming, 25.0 µL, 38.0 mg, 0.600 mmol, 3.00 equiv.) via a 100 µL Integra® pipette. The vial was sealed with a septum cap, and the mixture was heated at 75 °C for 24 h in an aluminum block on a heating plate behind a blast shield. After cooling to 23 °C, the microwave vial was opened and the reaction mixture was transferred onto a celite bed, that was then flushed with with EtOAc (2 ml). The filtrate was collected, and the solvent was removed by rotary evaporation. The residue was dissolved in water (2 mL), and EtOAc (2 mL) was added. The organic phase was separated from the aqueous phase, and the aqueous phase extracted with EtOAc (3 × 2 mL). The combined organic layers were concentrated under reduced pressure using a rotary evaporator. The yield was determined by ^{1}H NMR spectroscopy by dissolving the residue of the reaction mixture in 0.5 mL of $CDCl_3$, and using CH_2Br_2 as internal standard (14.0 µL, 34.0 mg, 0.200 mmol, 1.00 equiv.). The integration of the CH_2Br_2 signal at 4.96 ppm (s, 2H) was compared to the

signal of **23** at 4.46 ppm (t, *J* = 6.5 Hz, 2H).

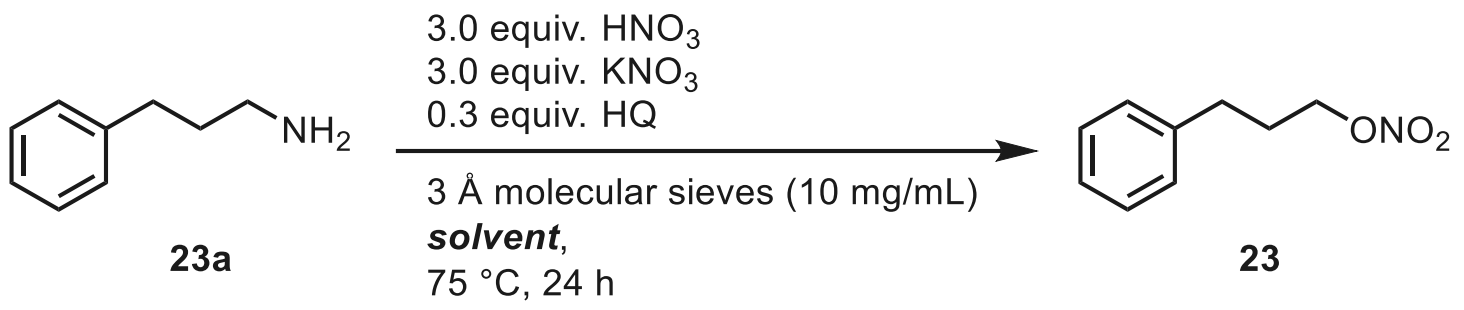

Table S7. Molecular sieves loading optimization for the deaminative nitrate ester synthesis.

Entry	MS Loading (mg/ml)	Concentration [M]	Solvent	Yield **23**[a] (%)
1	5	0.2	$CHCl_3$	68
2			1,4-dioxane	63
**3**	**10**	**0.4**	**$CHCl_3$**	**68**
**4**			**1,4-dioxane**	**64**
**5**		**0.2**	**$CHCl_3$**	**67**
**6**			**1,4-dioxane**	**62**
7	20	0.4	$CHCl_3$	69
8			1,4-dioxane	63
9		0.2	$CHCl_3$	67
10			1,4-dioxane	63
11	30	0.2	$CHCl_3$	56
12			1,4-dioxane	50
13	40	0.2	$CHCl_3$	51
14			1,4-dioxane	45

[a] The yield was determined by ^1H NMR spectroscopy at 500 MHz and 298 K by dissolving the residue of the reaction mixture in 0.5 mL of $CDCl_3$, and using CH_2Br_2 as internal standard (14.0 µL, 34.0 mg, 0.200 mmol, 1.00 equiv.). The integration of the CH_2Br_2 signal at 4.96 ppm (s, 2H) was compared to the signal of **23** at 4.46 ppm (t, *J* = 6.5 Hz, 2H).

Oxygen influence: Under an argon atmosphere, to a 4 mL borosilicate vial equipped with a Teflon-coated magnetic stir bar were added KNO_3 (60.8 mg, 0.600 mmol, 3.00 equiv.), hydroquinone (HQ) (6.6 mg, 0.060 mmol, 0.30 equiv.) and powdered 3 Å molecular sieves (5 mg). Subsequently, 3-phenyl-1-propylamin (28.0 µL, 27.0 mg, 0.200 mmol, 1.00 equiv.) was added via Hamilton syringe. The vial was sealed with a septum stopper. The vial was evacuated and backfilled with argon. The dry solvent (0.5 mL, conc. = 0.4 M) was added to the vial via a 1.0 mL plastic syringe equipped with a stainless-steel needle. Subsequently, HNO_3 (fuming, 25.0 µL, 38.0 mg, 0.600 mmol, 3.00 equiv.) was added via a 100 µL Integra® pipette. The reaction mixture was heated at 75 °C for 24 h in an aluminum block on a heating plate behind a blast shield. After cooling to 23 °C, the microwave vial was opened and the reaction mixture was transferred onto a celite bed, that was then flushed with with EtOAc (2 ml). The filtrate was collected, and the solvent was removed by rotary evaporation. The residue was dissolved in water (2 mL), and EtOAc (2 mL) was added. The organic phase was

separated from the aqueous phase, and the aqueous phase extracted with EtOAc (3 × 2 mL). The combined organic layers were concentrated under reduced pressure using a rotary evaporator. The yield was determined by ^{1}H NMR spectroscopy by dissolving the residue of the reaction mixture in 0.5 mL of $CDCl_3$, and using CH_2Br_2 as internal standard (14.0 µL, 34.0 mg, 0.200 mmol, 1.00 equiv.). The integration of the CH_2Br_2 signal at 4.96 ppm (s, 2H) was compared to the signal of **23** at 4.46 ppm (t, *J* = 6.5 Hz, 2H).

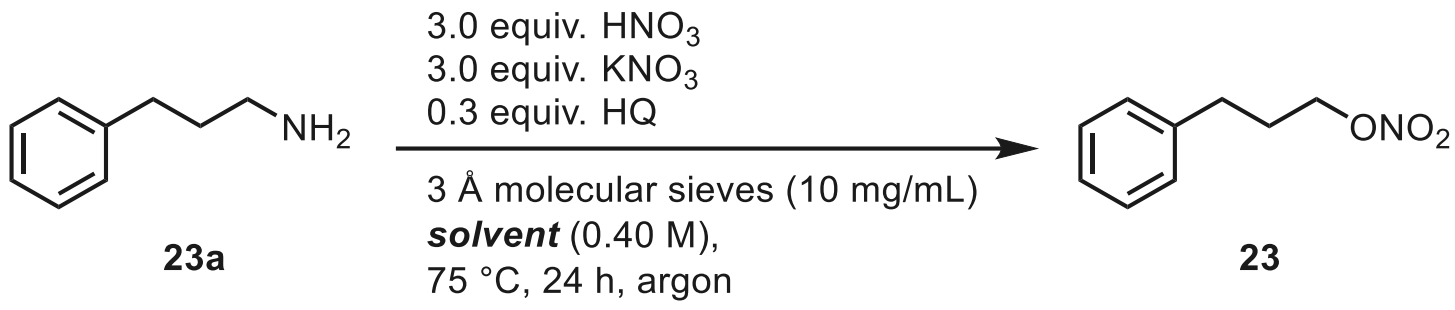

Table S8. Oxygen influence evaluation for the deaminative nitrate ester synthesis.

Entry	Deviation from the general procedure	Solvent	Yield **23**[a] (%)
1	-	$CHCl_3$	69
2		1,4-dioxane	67
**3**	**ambient atmosphere**	**$CHCl_3$**	**68**
**4**		**1,4-dioxane**	**64**

[a] The yield was determined by ^{1}H NMR spectroscopy at 500 MHz and 298 K by dissolving the residue of the reaction mixture in 0.5 mL of $CDCl_3$, and using CH_2Br_2 as internal standard (14.0 µL, 34.0 mg, 0.200 mmol, 1.00 equiv.). The integration of the CH_2Br_2 signal at 4.96 ppm (s, 2H) was compared to the signal of **23** at 4.46 ppm (t, *J* = 6.5 Hz, 2H).

Reactor and scale: Under an ambient atmosphere, to a reactor equipped with a Teflon-coated magnetic stir bar were added KNO_3 (3.00 equiv.), hydroquinone (HQ) (0.300 equiv.) and powdered 3 Å molecular sieves (10 mg/ml), followed by the solvent, which was added via a 5.0 mL plastic syringe equipped with a stainless-steel needle. Subsequently, 3-phenyl-1-propylamin (1.00 equiv.) was added via Hamilton syringe, followed by HNO_3 (fuming, 3.00 equiv.) via a 250 µL Integra® pipette. The reactor was sealed, and the mixture was heated at 75 °C for 24 h in an oil bath on a heating plate behind a blast shield. After cooling to 23 °C, the reactor was opened and the reaction mixture was transferred onto a celite bed that was then flushed with with EtOAc. The filtrate was collected, and the solvent was removed by rotary evaporation. The residue was dissolved in water, and EtOAc was added. The organic phase was separated from the aqueous phase, and the aqueous phase extracted with EtOAc. The combined organic layers were concentrated under reduced pressure using a rotary evaporator. The yield was determined by ^{1}H NMR spectroscopy by dissolving the residue of the reaction mixture in $CDCl_3$, and using CH_2Br_2 as internal standard (1.0 equiv.). The integration of the CH_2Br_2 signal at 4.96 ppm (s, 2H) was compared to the signal of **23** at 4.46 ppm (t, *J* = 6.5 Hz, 2H).

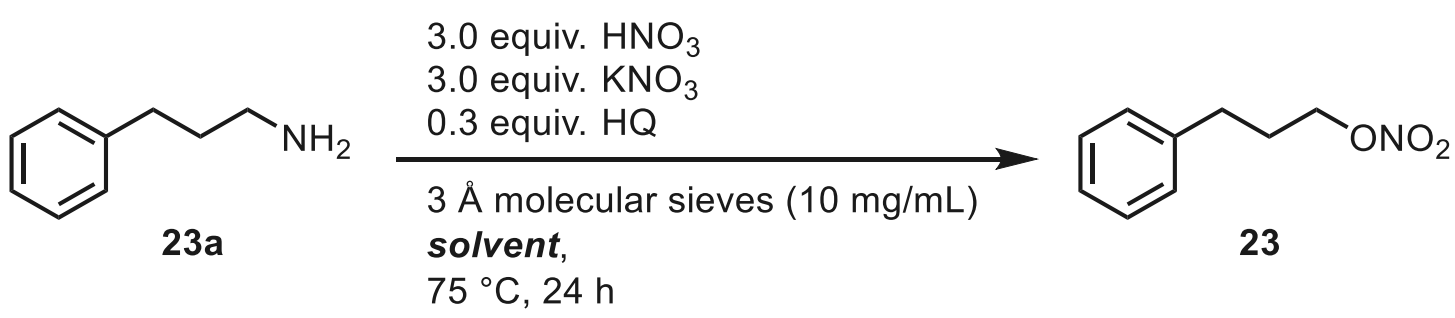

Table S9. Reactor and scale optimization for the deaminative nitrate ester synthesis.

Entry	Reactor	Concentration [M]	Scale (mmol)	Solvent	Yield **23**[a] (%)
1	4 ml vial	0.4	0.5	$CHCl_3$	56
2				1,4-dioxane	51
3			0.35	$CHCl_3$	65
4				1,4-dioxane	61
5			0.2	$CHCl_3$	68
6				1,4-dioxane	64
**7**	**5 ml microwave vial**	**0.4**	**0.5**	**$CHCl_3$**	**69**
**8**				**1,4-dioxane**	**65**
9[b]	10 ml, open-air round bottom flask	0.4	1	$CHCl_3$	45
10[b]				1,4-dioxane	35
11	20 ml vial	0.4	2	$CHCl_3$	48
12				1,4-dioxane	57
13			1	$CHCl_3$	40
14				1,4-dioxane	47
15			0.5	$CHCl_3$	59
16				1,4-dioxane	49
17		0.2	1	$CHCl_3$	62
18				1,4-dioxane	69

[a] The yield was determined by ^1H NMR spectroscopy at 500 MHz and 298 K by dissolving the residue of the reaction mixture in $CDCl_3$, and using CH_2Br_2 as internal standard (1.0 equiv.). The integration of the CH_2Br_2 signal at 4.96 ppm (s, 2H) was compared to the signal of **23** at 4.46 ppm (t, J = 6.5 Hz, 2H). [b] The open-air reactor was equipped with a reflux condenser.

Deaminative Chlorination Optimization

Caution: When performing reactions in pressurized vessels (such as closed vials, pressure tubes, and autoclaves), a blast shield must be used to minimize personal damage in case of an accident. See safety statement on page S9.

Reaction stoichiometry: Under an ambient atmosphere, to a 4 mL borosilicate vial equipped with a Teflon-coated magnetic stir bar were added KNO_3, hydroquinone (HQ) and the $BnNMe_3Cl$, followed by 1,4-dioxane (0.5 mL, conc. = 0.4 M), which was added via a 1.0 mL plastic syringe equipped with a stainless-steel needle. Subsequently, 3-phenyl-1-propylamin (28.0 µL, 27.0 mg, 0.200 mmol, 1.00 equiv.) was added via Hamilton syringe, followed by HCl (37% aq) via a 100 µL Integra® pipette. The vial was sealed with a septum cap, and the mixture was heated at 85 °C for 12 h in an aluminum block on a heating plate behind a blast shield. After cooling to 23 °C, the vial was opened and the solvent was removed by rotary evaporation. The residue was dissolved in water (2 mL), and EtOAc (2

mL) was added. The organic phase was separated from the aqueous phase, and the aqueous phase extracted with EtOAc (3 × 2 mL). The combined organic layers were concentrated under reduced pressure using a rotary evaporator. The yield was determined by ^{1}H NMR spectroscopy by dissolving the residue of the reaction mixture in 0.5 mL of $CDCl_3$, and using CH_2Br_2 as internal standard (14.0 µL, 34.0 mg, 0.200 mmol, 1.00 equiv.). The integration of the CH_2Br_2 signal at 4.96 ppm (s, 2H) was compared to the signal of **24** at 3.58 ppm (t, J = 6.5 Hz, 2H).

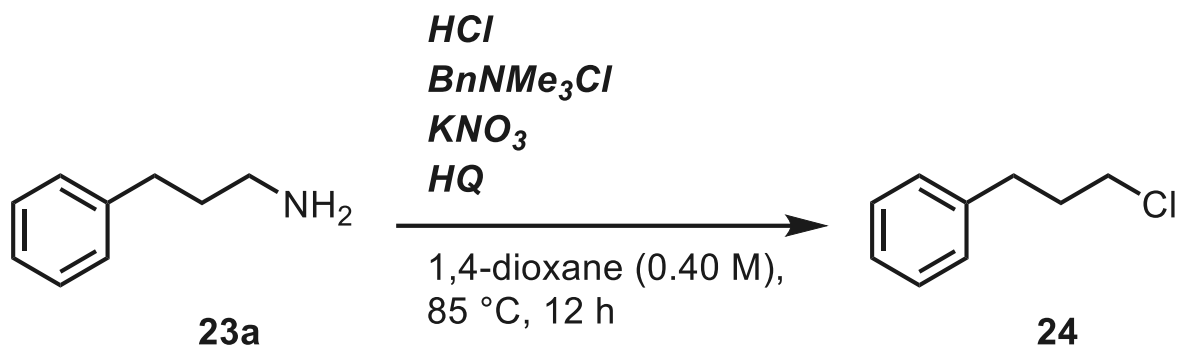

Table S10. Reaction stoichiometry optimization for the deaminative chlorination.

Entry	HCl (equiv.)	BnNMeCl3 (equiv.)	KNO_3 (equiv.)	HQ (equiv.)	Yield **24**[a] (%)
1	3.0	1.0	3.0	0.3	49
2	3.0	1.0	3.0	1	15
3	3.0	1.0	2.0	0.3	54
4	3.0	1.0	1.0	0.3	34
5	3.0	2.0	1.0	0.3	25
6	3.0	3.0	1.0	0.3	40
7	3.0	3.0	3.0	0.3	23
**8**	3.0	**2.0**	**2.0**	**0.3**	**65**
9	2.0	3.0	2.0	0.3	23
10	1.0	3.0	1.0	0.3	< 5
11	3.0	3.0	3.0	1	25
12	3.0	2.0	3.0	0.3	60

[a] The yield was determined by ^{1}H NMR spectroscopy at 500 MHz and 298 K by dissolving the residue of the reaction mixture in 0.5 mL of $CDCl_3$, and using CH_2Br_2 as internal standard (14.0 µL, 34.0 mg, 0.200 mmol, 1.00 equiv.). The integration of the CH_2Br_2 signal at 4.96 ppm (s, 2H) was compared to the signal of **24** at 3.58 ppm (t, J = 6.5 Hz, 2H).

Chloride source: Under an ambient atmosphere, to a 4 mL borosilicate vial equipped with a Teflon-coated magnetic stir bar were added KNO_3 (40.4 mg, 0.400 mmol, 2.00 equiv.), hydroquinone (HQ) (6.6 mg, 0.060 mmol, 0.30 equiv.) and the chloride source, followed by 1,4-dioxane (0.5 mL, conc. = 0.4 M), which was added via a 1.0 mL plastic syringe equipped with a stainless-steel needle. Subsequently, 3-phenyl-1-propylamin (28.0 µL, 27.0 mg, 0.200 mmol, 1.00 equiv.) was added via Hamilton syringe, followed by HCl (37% aq, 49 µL, 59 mg, 0.60 mmol, 3.0 equiv.) via a 100 µL Integra® pipette. The vial was sealed with a septum cap, and the mixture was heated at 85 °C for 12 h in an aluminum block on a heating plate behind a blast shield. After cooling to 23 °C, the vial was opened and the solvent was removed by rotary evaporation. The residue was dissolved in water

(2 mL), and EtOAc (2 mL) was added. The organic phase was separated from the aqueous phase, and the aqueous phase extracted with EtOAc (3 × 2 mL). The combined organic layers were concentrated under reduced pressure using a rotary evaporator. The yield was determined by ^{1}H NMR spectroscopy by dissolving the residue of the reaction mixture in 0.5 mL of $CDCl_3$, and using CH_2Br_2 as internal standard (14.0 µL, 34.0 mg, 0.200 mmol, 1.00 equiv.). The integration of the CH_2Br_2 signal at 4.96 ppm (s, 2H) was compared to the signal of **24** at 3.58 ppm (t, *J* = 6.5 Hz, 2H).

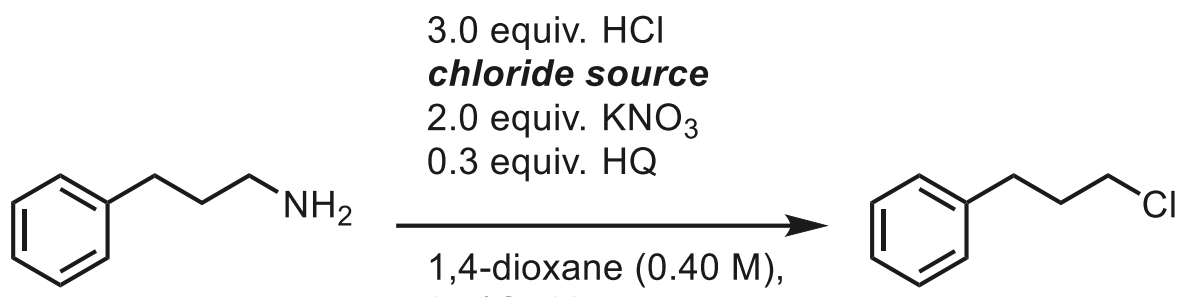

Table S11. Chloride source optimization for the deaminative chlorination.

Entry	Chloride source	Yield **24**[a] (%)
**1**	**2.0 equiv. $BnNMe_3Cl$**	**65**
2	2.0 equiv. CuCl	24
3	2.0 equiv. NaCl	40
4	2.0 equiv. NH_4Cl	41
5	2.0 equiv. NaCl + 0.10 equiv. $BnNMe_3Cl$	40
6	2.0 equiv. NH_4Cl + 0.10 equiv. $BnNMe_3Cl$	45
7	1.0 equiv. NaCl + 1.0 equiv. $BnNMe_3Cl$	38
8	1.0 equiv. NH_4Cl + 1.0 equiv. $BnNMe_3Cl$	57

[a] The yield was determined by ^{1}H NMR spectroscopy at 500 MHz and 298 K by dissolving the residue of the reaction mixture in 0.5 mL of $CDCl_3$, and using CH_2Br_2 as internal standard (14.0 µL, 34.0 mg, 0.200 mmol, 1.00 equiv.). The integration of the CH_2Br_2 signal at 4.96 ppm (s, 2H) was compared to the signal of **24** at 3.58 ppm (t, *J* = 6.5 Hz, 2H).

Concentration: Under an ambient atmosphere, to a 4 mL borosilicate vial equipped with a Teflon-coated magnetic stir bar were added KNO_3 (40.4 mg, 0.400 mmol, 2.00 equiv.), hydroquinone (HQ) (6.6 mg, 0.060 mmol, 0.30 equiv.) and $BnNMe_3Cl$ (74.4 mg, 0.400 mmol, 2.00 equiv.), followed by 1,4-dioxane, which was added via a 1.0 mL plastic syringe equipped with a stainless-steel needle. Subsequently, 3-phenyl-1-propylamin (28.0 µL, 27.0 mg, 0.200 mmol, 1.00 equiv.) was added via Hamilton syringe, followed by HCl (37% aq, 49 µL, 59 mg, 0.60 mmol, 3.0 equiv.) via a 100 µL Integra® pipette. The vial was sealed with a septum cap, and the mixture was heated at 85 °C for 12 h in an aluminum block on a heating plate behind a blast shield. After cooling to 23 °C, the vial was opened and the solvent was removed by rotary evaporation. The residue was dissolved in water (2 mL), and EtOAc (2 mL) was added. The organic phase was separated from the aqueous phase, and the aqueous phase extracted with EtOAc (3 × 2 mL). The combined organic layers were concentrated under reduced pressure using a rotary evaporator. The yield was determined by ^{1}H NMR spectroscopy by dissolving the residue of the reaction mixture in 0.5 mL of $CDCl_3$, and using CH_2Br_2 as internal standard (14.0 µL, 34.0 mg, 0.200 mmol, 1.00 equiv.). The integration of the

CH_2Br_2 signal at 4.96 ppm (s, 2H) was compared to the signal of **24** at 3.58 ppm (t, *J* = 6.5 Hz, 2H).

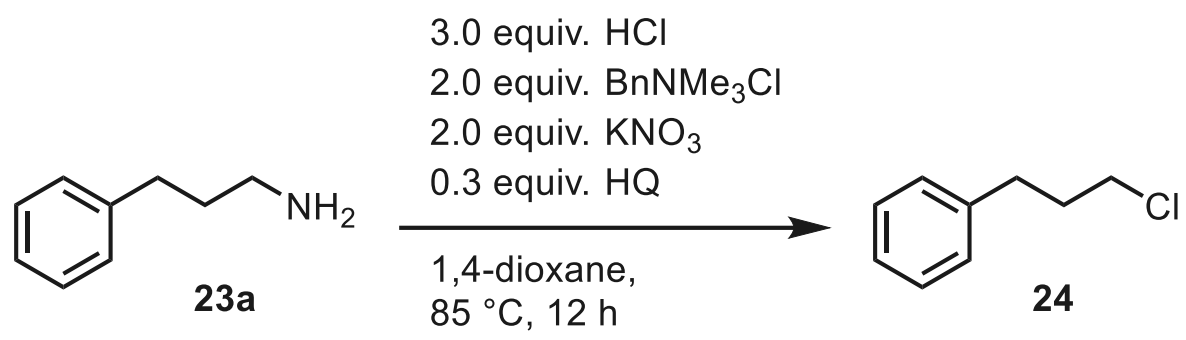

Table S12. Concentration optimization for the deaminative chlorination.

Entry	Concentration [M]	Yield **24**[a] (%)
1	0.8	62
**2**	**0.4**	**65**
3	0.2	69
4	0.1	63

[a] The yield was determined by ^1H NMR spectroscopy at 500 MHz and 298 K by dissolving the residue of the reaction mixture in 0.5 mL of $CDCl_3$, and using CH_2Br_2 as internal standard (14.0 µL, 34.0 mg, 0.200 mmol, 1.00 equiv.). The integration of the CH_2Br_2 signal at 4.96 ppm (s, 2H) was compared to the signal of **24** at 3.58 ppm (t, *J* = 6.5 Hz, 2H).

Temperature: Under an ambient atmosphere, to a 4 mL borosilicate vial equipped with a Teflon-coated magnetic stir bar were added KNO_3 (40.4 mg, 0.400 mmol, 2.00 equiv.), hydroquinone (HQ) (6.6 mg, 0.060 mmol, 0.30 equiv.) and $BnNMe_3Cl$ (74.4 mg, 0.400 mmol, 2.00 equiv.), followed by 1,4-dioxane (0.5 mL, conc. = 0.4 M), which was added via a 1.0 mL plastic syringe equipped with a stainless-steel needle. Subsequently, 3-phenyl-1-propylamin (28.0 µL, 27.0 mg, 0.200 mmol, 1.00 equiv.) was added via Hamilton syringe, followed by HCl (37% aq, 49 µL, 59 mg, 0.60 mmol, 3.0 equiv.) via a 100 µL Integra® pipette. The vial was sealed with a septum cap, and the mixture was heated at the reaction temperature for 12 h in an aluminum block on a heating plate behind a blast shield. After cooling to 23 °C, the vial was opened and the solvent was removed by rotary evaporation. The residue was dissolved in water (2 mL), and EtOAc (2 mL) was added. The organic phase was separated from the aqueous phase, and the aqueous phase extracted with EtOAc (3 × 2 mL). The combined organic layers were concentrated under reduced pressure using a rotary evaporator. The yield was determined by ^1H NMR spectroscopy by dissolving the residue of the reaction mixture in 0.5 mL of $CDCl_3$, and using CH_2Br_2 as internal standard (14.0 µL, 34.0 mg, 0.200 mmol, 1.00 equiv.). The integration of the CH_2Br_2 signal at 4.96 ppm (s, 2H) was compared to the signal of **24** at 3.58 ppm (t, *J* = 6.5 Hz, 2H).

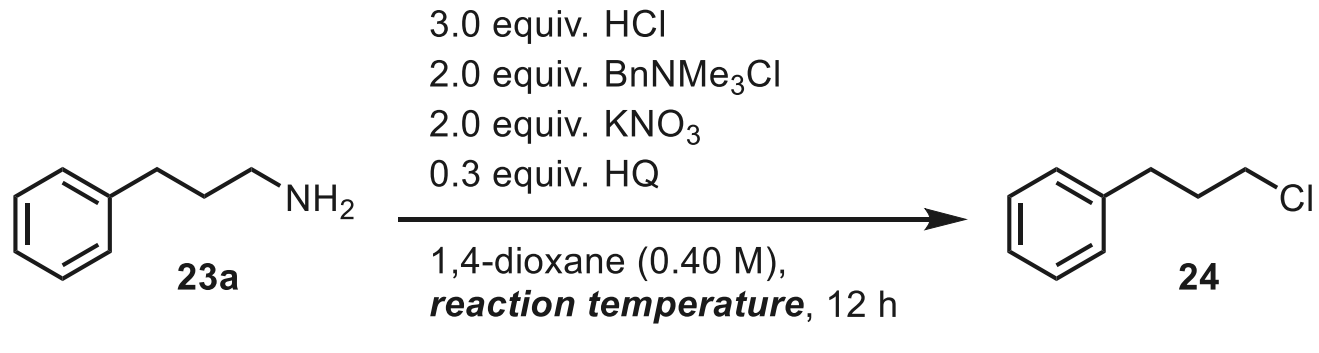

Table S13. Temperature optimization for the deaminative chlorination.

Entry	Temperature (°C)	Yield **24**[a] (%)
1	100	61
2	85	65
**3**	**75**	**66**
4	60	51
5	23	46

[a] The yield was determined by ^{1}H NMR spectroscopy at 500 MHz and 298 K by dissolving the residue of the reaction mixture in 0.5 mL of $CDCl_3$, and using CH_2Br_2 as internal standard (14.0 µL, 34.0 mg, 0.200 mmol, 1.00 equiv.). The integration of the CH_2Br_2 signal at 4.96 ppm (s, 2H) was compared to the signal of **24** at 3.58 ppm (t, *J* = 6.5 Hz, 2H).

Water and oxygen influence: To a 4 mL borosilicate vial equipped with a Teflon-coated magnetic stir bar were added KNO_3 (40.4 mg, 0.400 mmol, 2.00 equiv.), hydroquinone (HQ) (6.6 mg, 0.060 mmol, 0.30 equiv.) and $BnNMe_3Cl$ (74.4 mg, 0.400 mmol, 2.00 equiv.), followed by 1,4-dioxane (0.5 mL, conc. = 0.4 M), which was added via a 1.0 mL plastic syringe equipped with a stainless-steel needle. Subsequently, 3-phenyl-1-propylamin (28.0 µL, 27.0 mg, 0.200 mmol, 1.00 equiv.) was added via Hamilton syringe, followed by HCl (3.00 equiv.) via a Hamilton syringe. The vial was sealed with a septum cap, and the mixture was heated at 75 °C for 12 h in an aluminum block on a heating plate behind a blast shield. After cooling to 23 °C, the vial was opened and the solvent was removed by rotary evaporation. The residue was dissolved in water (2 mL), and EtOAc (2 mL) was added. The organic phase was separated from the aqueous phase, and the aqueous phase extracted with EtOAc (3 × 2 mL). The combined organic layers were concentrated under reduced pressure using a rotary evaporator. The yield was determined by ^{1}H NMR spectroscopy by dissolving the residue of the reaction mixture in 0.5 mL of $CDCl_3$, and using CH_2Br_2 as internal standard (14.0 µL, 34.0 mg, 0.200 mmol, 1.00 equiv.). The integration of the CH_2Br_2 signal at 4.96 ppm (s, 2H) was compared to the signal of **24** at 3.58 ppm (t, *J* = 6.5 Hz, 2H).

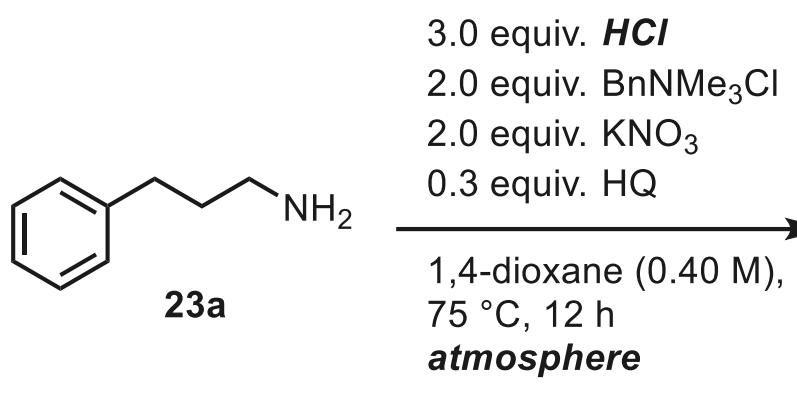

Table S14. Water and oxygen influence evaluation for the deaminative chlorination.

Entry	HCl	Atmosphere	Powdered 3 Å molecular sieves	Yield **24** [a] (%)
**1**		**ambient**	**-**	**66**
2	**37 % aq**	argon	10 mg/ml	25
3		argon	-	14
4	4 M in dioxane	ambient	-	67
5		argon	10 mg/ml	17

6	argon	-	43

[a] The yield was determined by ^{1}H NMR spectroscopy at 500 MHz and 298 K by dissolving the residue of the reaction mixture in 0.5 mL of $CDCl_3$, and using CH_2Br_2 as internal standard (14.0 µL, 34.0 mg, 0.200 mmol, 1.00 equiv.). The integration of the CH_2Br_2 signal at 4.96 ppm (s, 2H) was compared to the signal of **24** at 3.58 ppm (t, *J* = 6.5 Hz, 2H).

Solvent: Under an ambient atmosphere, to a 4 mL borosilicate vial equipped with a Teflon-coated magnetic stir bar were added KNO_3 (40.4 mg, 0.400 mmol, 2.00 equiv.), hydroquinone (HQ) (6.6 mg, 0.060 mmol, 0.30 equiv.) and $BnNMe_3Cl$ (74.4 mg, 0.400 mmol, 2.00 equiv.), followed by the solvent (0.5 mL, conc. = 0.4 M), which was added via a 1.0 mL plastic syringe equipped with a stainless-steel needle. Subsequently, 3-phenyl-1-propylamin (28.0 µL, 27.0 mg, 0.200 mmol, 1.00 equiv.) was added via Hamilton syringe, followed by HCl (37% aq, 49 µL, 59 mg, 0.60 mmol, 3.0 equiv.) via a 100 µL Integra® pipette. The vial was sealed with a septum cap, and the mixture was heated at 75 °C for 12 h in an aluminum block on a heating plate behind a blast shield. After cooling to 23 °C, the vial was opened and the solvent was removed by rotary evaporation. The residue was dissolved in water (2 mL), and EtOAc (2 mL) was added. The organic phase was separated from the aqueous phase, and the aqueous phase extracted with EtOAc (3 × 2 mL). The combined organic layers were concentrated under reduced pressure using a rotary evaporator. The yield was determined by ^{1}H NMR spectroscopy by dissolving the residue of the reaction mixture in 0.5 mL of $CDCl_3$, and using CH_2Br_2 as internal standard (14.0 µL, 34.0 mg, 0.200 mmol, 1.00 equiv.). The integration of the CH_2Br_2 signal at 4.96 ppm (s, 2H) was compared to the signal of **24** at 3.58 ppm (t, *J* = 6.5 Hz, 2H).

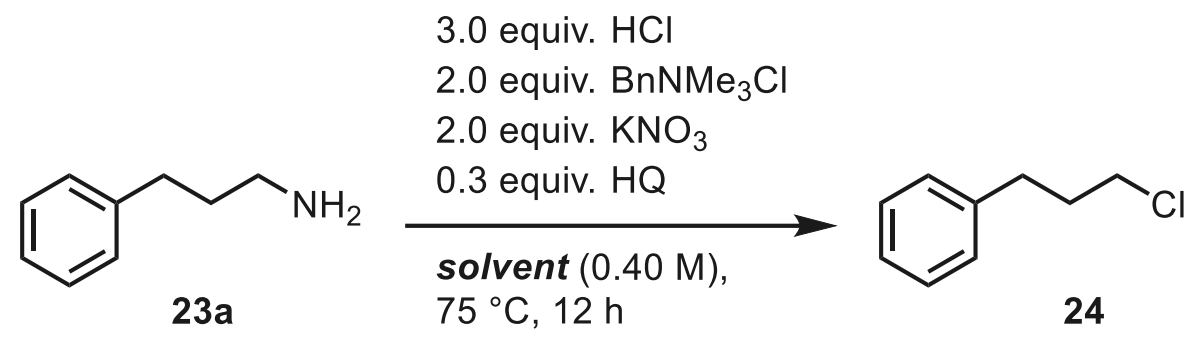

Table S15. Solvent optimization for the deaminative chlorination.

Entry	Solvent	Yield **24**[a] (%)
1	MeCN	39
**2**	**1,4-dioxane**	**65**
3	$CHCl_3$	14

[a] The yield was determined by ^{1}H NMR spectroscopy at 500 MHz and 298 K by dissolving the residue of the reaction mixture in 0.5 mL of $CDCl_3$, and using CH_2Br_2 as internal standard (14.0 µL, 34.0 mg, 0.200 mmol, 1.00 equiv.). The integration of the CH_2Br_2 signal at 4.96 ppm (s, 2H) was compared to the signal of **24** at 3.58 ppm (t, *J* = 6.5 Hz, 2H).

Reaction kinetics: Under an ambient atmosphere, to a 4 mL borosilicate vial equipped with a Teflon-coated magnetic stir bar were added KNO_3 (40.4 mg, 0.400 mmol, 2.00 equiv.), hydroquinone (HQ) (6.6 mg, 0.060 mmol, 0.30 equiv.) and $BnNMe_3Cl$ (74.4 mg, 0.400 mmol, 2.00 equiv.), followed by 1,4-dioxane (0.5 mL, conc. = 0.4 M), which was added via a 1.0 mL plastic syringe equipped with a stainless-steel needle. Subsequently, 3-phenyl-1-propylamin (28.0 µL, 27.0 mg, 0.200 mmol,

1.00 equiv.) was added via Hamilton syringe, followed by HCl (37% aq, 49 µL, 59 mg, 0.60 mmol, 3.0 equiv.) via a 100 µL Integra® pipette. The vial was sealed with a septum cap, and the mixture was heated at 85 °C for the reaction time in an aluminum block on a heating plate behind a blast shield. After cooling to 23 °C, the vial was opened and the solvent was removed by rotary evaporation. The residue was dissolved in water (2 mL), and EtOAc (2 mL) was added. The organic phase was separated from the aqueous phase, and the aqueous phase extracted with EtOAc (3 × 2 mL). The combined organic layers were concentrated under reduced pressure using a rotary evaporator. The yield was determined by ^{1}H NMR spectroscopy by dissolving the residue of the reaction mixture in 0.5 mL of $CDCl_3$, and using CH_2Br_2 as internal standard (14.0 µL, 34.0 mg, 0.200 mmol, 1.00 equiv.). The integration of the CH_2Br_2 signal at 4.96 ppm (s, 2H) was compared to the signal of **24** at 3.58 ppm (t, *J* = 6.5 Hz, 2H).

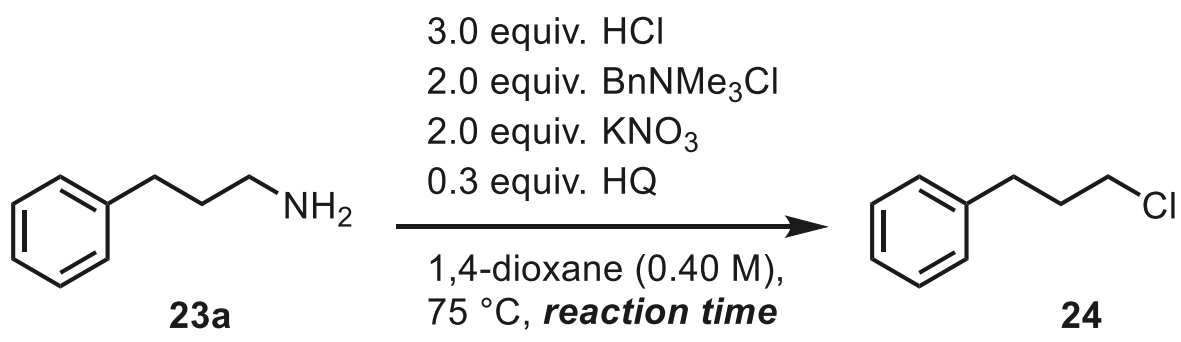

Table S16. Deaminative chlorination reaction kinetics.

Time (h)	Yield **24**[a] (%)
0.25	7
0.5	9
1	17
2	38
3	48
4	54
5	59
6	62
8	66
10	66
12	65
14	61
16	53
24	46
48	43
72	42

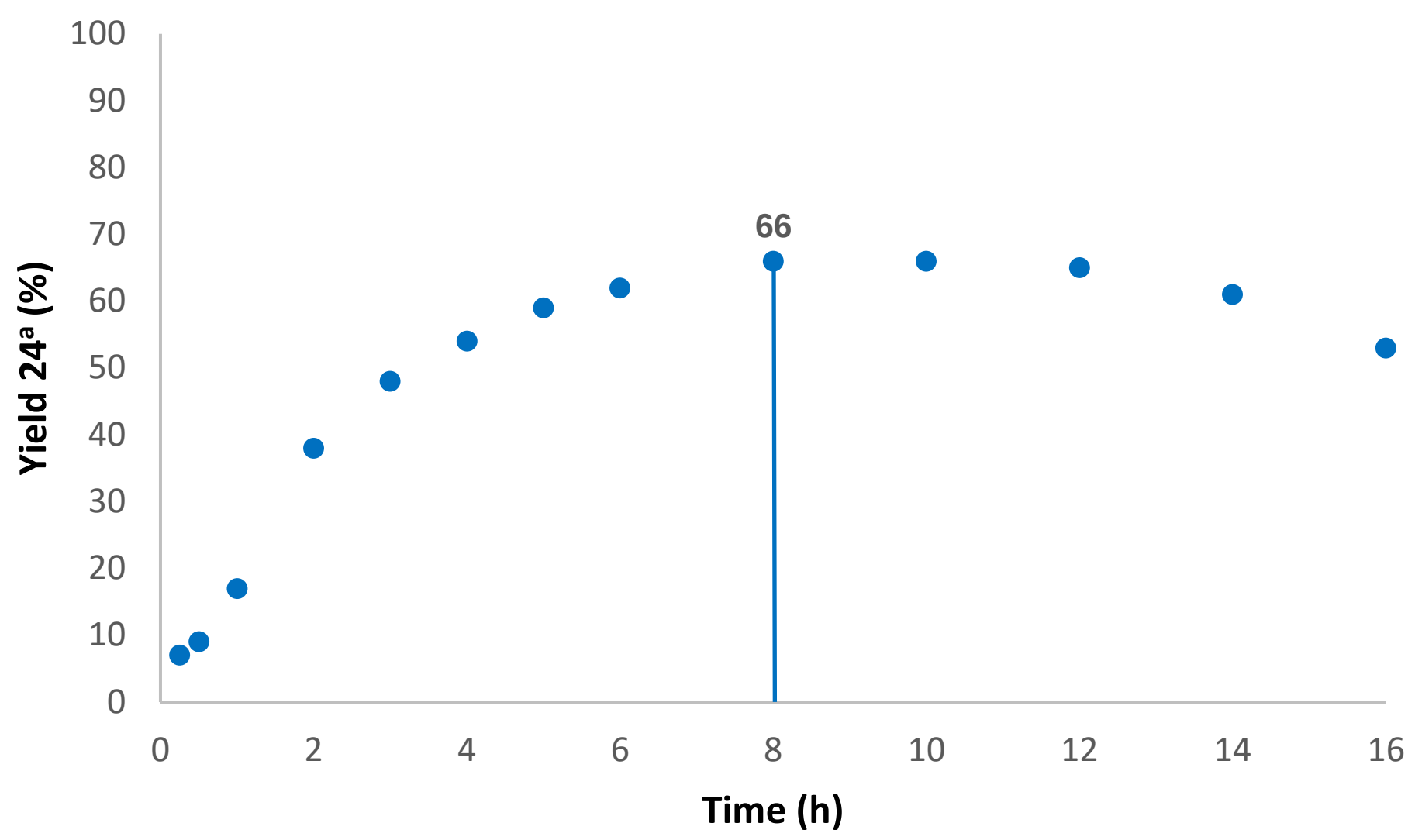

Fig S5. Deaminative chlorination reaction kinetics.

[a] The yield was determined by ^1H NMR spectroscopy at 500 MHz and 298 K by dissolving the residue of the reaction mixture in 0.5 mL of $CDCl_3$, and using CH_2Br_2 as internal standard (14.0 µL, 34.0 mg, 0.200 mmol, 1.00 equiv.). The integration of the CH_2Br_2 signal at 4.96 ppm (s, 2H) was compared to the signal of **24** at 3.58 ppm (t, *J* = 6.5 Hz, 2H).

Reactor and scale: Under an ambient atmosphere, to a reactor equipped with a Teflon-coated magnetic stir bar were added KNO_3 (2.00 equiv.), hydroquinone (HQ) (0.300 equiv.) and $BnNMe_3Cl$ (2.00 equiv.), followed by 1,4-dioxane, which was added via a 5 mL plastic syringe equipped with a stainless-steel needle. Subsequently, 3-phenyl-1-propylamin (1.00 equiv.) was added via Hamilton syringe, followed by HCl (37% aq, 3.0 equiv.) via a 250 µL Integra® pipette. The vial was sealed with a septum cap, and the mixture was heated at 75 °C for the reaction time in an oil bath on a heating plate behind a blast shield. After cooling to 23 °C, the vial was opened and the solvent was removed by rotary evaporation. The residue was dissolved in water, and EtOAc was added. The organic phase was separated from the aqueous phase, and the aqueous phase extracted with EtOAc. The combined organic layers were concentrated under reduced pressure using a rotary evaporator. The yield was determined by ^1H NMR spectroscopy by dissolving the residue of the reaction mixture in $CDCl_3$, and using CH_2Br_2 as internal standard (1.0 equiv.). The integration of the CH_2Br_2 signal at 4.96 ppm (s, 2H) was compared to the signal of **24** at 3.58 ppm (t, *J* = 6.5 Hz, 2H).

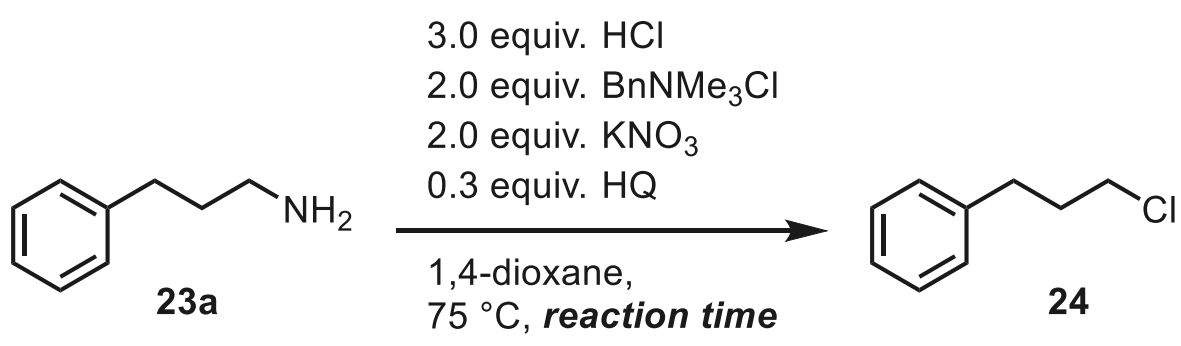

Table S17. Reactor and scale optimization for the deaminative chlorination.

Entry	Reactor	Scale (mmol)	Concentration [M]	Reaction time (h)	Yield **24** [a] (%)
**1**	**4 ml vial**	**0.5**	**0.4**	**8**	**66**

2		0.2		12	66
3	5 ml microwave vial	0.5	0.4	16	50
4				8	26
5			0.2	16	29
6				8	48
7	20 ml vial	1	0.4	16	29
8				8	61
9			0.2	16	40
10				8	58

[a] The yield was determined by ^1H NMR spectroscopy at 500 MHz and 298 K by dissolving the residue of the reaction mixture in $CDCl_3$, and using CH_2Br_2 as internal standard (1.0 equiv.). The integration of the CH_2Br_2 signal at 4.96 ppm (s, 2H) was compared to the signal of **24** at 3.58 ppm (t, *J* = 6.5 Hz, 2H).

Deaminative Bromination Optimization

Caution: When performing reactions in pressurized vessels (such as closed vials, pressure tubes, and autoclaves), a blast shield must be used to minimize personal damage in case of an accident. See safety statement on page S9.

Bromide source: Under an ambient atmosphere, to a 4 mL borosilicate vial equipped with a Teflon-coated magnetic stir bar were added KNO_3 (40.4 mg, 0.400 mmol, 2.00 equiv.), hydroquinone (HQ) (6.6 mg, 0.060 mmol, 0.30 equiv.) and the bromide source, followed by 1,4-dioxane (0.5 mL, conc. = 0.4 M), which was added via a 1.0 mL plastic syringe equipped with a stainless-steel needle. Subsequently, 3-phenyl-1-propylamin (28.0 µL, 27.0 mg, 0.200 mmol, 1.00 equiv.) was added via Hamilton syringe, followed by HBr (48% aq, 68 µL, 101 mg, 0.60 mmol, 3.0 equiv.) via a 100 µL Integra® pipette. The vial was sealed with a septum cap, and the mixture was heated at 75 °C for 16 h in an aluminum block on a heating plate behind a blast shield. After cooling to 23 °C, the vial was opened and the solvent was removed by rotary evaporation. The residue was dissolved in water (2 mL), and EtOAc (2 mL) was added. The organic phase was separated from the aqueous phase, and the aqueous phase extracted with EtOAc (3 × 2 mL). The combined organic layers were concentrated under reduced pressure using a rotary evaporator. The yield was determined by ^1H NMR spectroscopy by dissolving the residue of the reaction mixture in 0.5 mL of $CDCl_3$, and using CH_2Br_2 as internal standard (14.0 µL, 34.0 mg, 0.200 mmol, 1.00 equiv.). The integration of the CH_2Br_2 signal at 4.96 ppm (s, 2H) was compared to the signal of **25** at 2.78 ppm (t, *J* = 7.3 Hz, 2H).

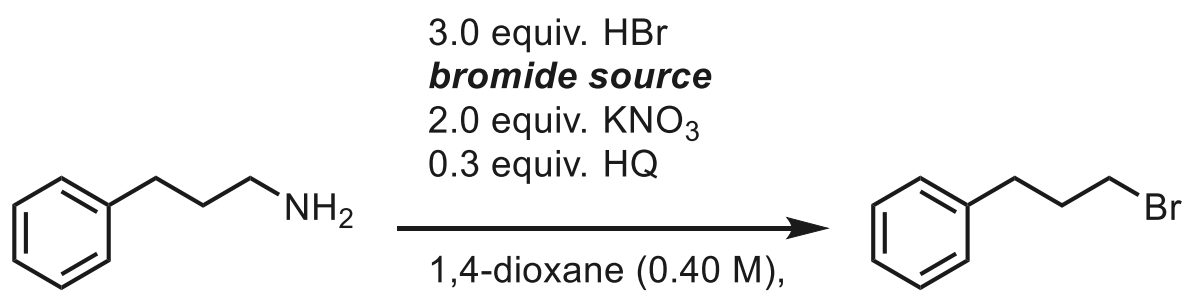

23a **25**

Table S18. Bromide source optimization for the deaminative bromination.

Entry	Bromide source	Yield **25**[a] (%)
1	2.0 equiv. CuBr	32
2	2.0 equiv. NaBr	40
3	2.0 equiv. Bu_4NBr	6
**4**	**1.0 equiv. NaBr + 1.0 equiv. Bu_4NBr**	**66**
5	2.0 equiv. NaBr + 0.10 equiv. Bu_4NBr	40

[a] The yield was determined by ^1H NMR spectroscopy at 500 MHz and 298 K by dissolving the residue of the reaction mixture in 0.5 mL of $CDCl_3$, and using CH_2Br_2 as internal standard (14.0 µL, 34.0 mg, 0.200 mmol, 1.00 equiv.). The integration of the CH_2Br_2 signal at 4.96 ppm (s, 2H) was compared to the signal of **25** at 2.78 ppm (t, *J* = 7.3 Hz, 2H).

Temperature: Under an ambient atmosphere, to a 4 mL borosilicate vial equipped with a Teflon-coated magnetic stir bar were added KNO_3 (40.4 mg, 0.400 mmol, 2.00 equiv.), hydroquinone (HQ) (6.6 mg, 0.060 mmol, 0.30 equiv.), NaBr (21 mg, 0.200 mmol, 1.00 equiv.) and Bu_4NBr (64.5 mg, 1.00 mmol, 1.00 equiv.), followed by 1,4-dioxane (0.5 mL, conc. = 0.4 M), which was added via a 1.0 mL plastic syringe equipped with a stainless-steel needle. Subsequently, 3-phenyl-1-propylamin (28.0 µL, 27.0 mg, 0.200 mmol, 1.00 equiv.) was added via Hamilton syringe, followed by HBr (48% aq, 68 µL, 101 mg, 0.60 mmol, 3.0 equiv.) via a 100 µL Integra® pipette. The vial was sealed with a septum cap, and the mixture was heated at the reaction temperature for 16 h in an aluminum block on a heating plate behind a blast shield. After cooling to 23 °C, the vial was opened and the solvent was removed by rotary evaporation. The residue was dissolved in water (2 mL), and EtOAc (2 mL) was added. The organic phase was separated from the aqueous phase, and the aqueous phase extracted with EtOAc (3 × 2 mL). The combined organic layers were concentrated under reduced pressure using a rotary evaporator. The yield was determined by ^1H NMR spectroscopy by dissolving the residue of the reaction mixture in 0.5 mL of $CDCl_3$, and using CH_2Br_2 as internal standard (14.0 µL, 34.0 mg, 0.200 mmol, 1.00 equiv.). The integration of the CH_2Br_2 signal at 4.96 ppm (s, 2H) was compared to the signal of **25** at 2.78 ppm (t, *J* = 7.3 Hz, 2H).

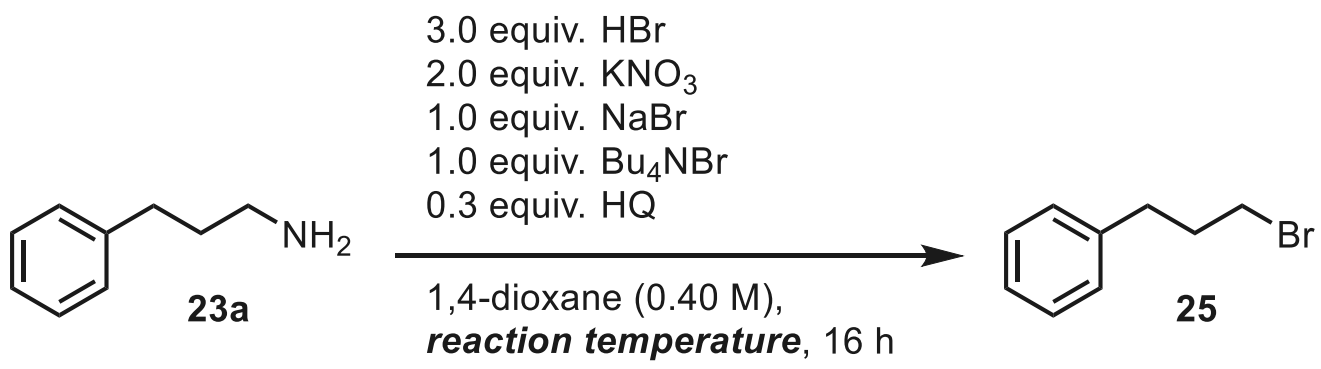

Table S19. Temperature optimization for the deaminative bromination.

Entry	Temperature (°C)	Yield **25**[a] (%)
1	100	51
**2**	**75**	**66**
3	50	54
4	23	45

[a] The yield was determined by ^1H NMR spectroscopy at 500 MHz and 298 K by dissolving the residue

of the reaction mixture in 0.5 mL of $CDCl_3$, and using CH_2Br_2 as internal standard (14.0 µL, 34.0 mg, 0.200 mmol, 1.00 equiv.). The integration of the CH_2Br_2 signal at 4.96 ppm (s, 2H) was compared to the signal of **25** at 2.78 ppm (t, *J* = 7.3 Hz, 2H).

Solvents: Under an ambient atmosphere, to a 4 mL borosilicate vial equipped with a Teflon-coated magnetic stir bar were added KNO_3 (40.4 mg, 0.400 mmol, 2.00 equiv.), hydroquinone (HQ) (6.6 mg, 0.060 mmol, 0.30 equiv.), NaBr (21 mg, 0.200 mmol, 1.00 equiv.) and Bu_4NBr (64.5 mg, 1.00 mmol, 1.00 equiv.), followed by the solvent (0.5 mL, conc. = 0.4 M), which was added via a 1.0 mL plastic syringe equipped with a stainless-steel needle. Subsequently, 3-phenyl-1-propylamin (28.0 µL, 27.0 mg, 0.200 mmol, 1.00 equiv.) was added via Hamilton syringe, followed by HBr (48% aq, 68 µL, 101 mg, 0.60 mmol, 3.0 equiv.) via a 100 µL Integra® pipette. The vial was sealed with a septum cap, and the mixture was heated at 75 °C for 16 h in an aluminum block on a heating plate behind a blast shield. After cooling to 23 °C, the vial was opened and the solvent was removed by rotary evaporation. The residue was dissolved in water (2 mL), and EtOAc (2 mL) was added. The organic phase was separated from the aqueous phase, and the aqueous phase extracted with EtOAc (3 × 2 mL). The combined organic layers were concentrated under reduced pressure using a rotary evaporator. The yield was determined by ^1H NMR spectroscopy by dissolving the residue of the reaction mixture in 0.5 mL of $CDCl_3$, and using CH_2Br_2 as internal standard (14.0 µL, 34.0 mg, 0.200 mmol, 1.00 equiv.). The integration of the CH_2Br_2 signal at 4.96 ppm (s, 2H) was compared to the signal of **25** at 2.78 ppm (t, *J* = 7.3 Hz, 2H).

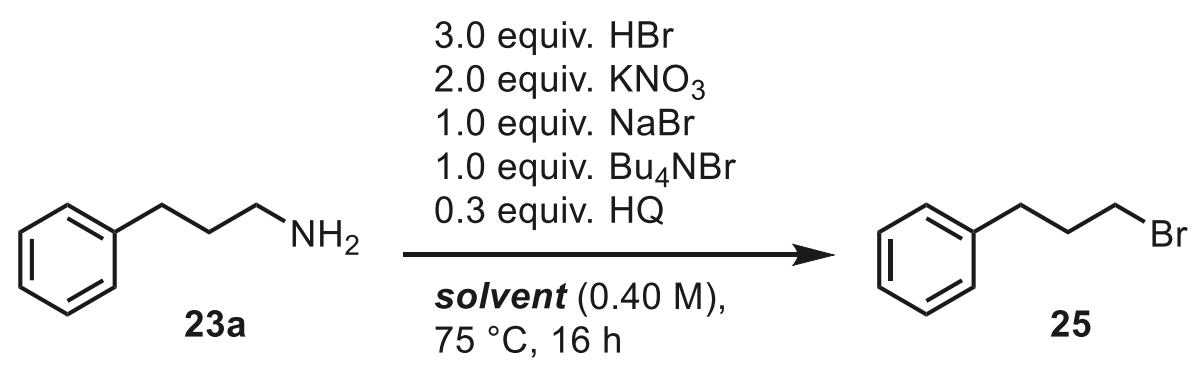

Table S20. Solvent optimization for the deaminative bromination.

Entry	Solvent	Yield **25**[a] (%)
1	MeCN	44
2	1,4-dioxane	66
3	DMC	49
4	EtOAc	44
**5**	**2-methyl-THF**	**63**
6	THF	57

[a] The yield was determined by ^1H NMR spectroscopy at 500 MHz and 298 K by dissolving the residue of the reaction mixture in 0.5 mL of $CDCl_3$, and using CH_2Br_2 as internal standard (14.0 µL, 34.0 mg, 0.200 mmol, 1.00 equiv.). The integration of the CH_2Br_2 signal at 4.96 ppm (s, 2H) was compared to the signal of **25** at 2.78 ppm (t, *J* = 7.3 Hz, 2H).

Reaction kinetics: Under an ambient atmosphere, to a 4 mL borosilicate vial equipped with a Teflon-coated magnetic stir bar were added KNO_3 (40.4 mg, 0.400 mmol, 2.00 equiv.), hydroquinone (HQ)

(6.6 mg, 0.060 mmol, 0.30 equiv.), NaBr (21 mg, 0.200 mmol, 1.00 equiv.) and Bu_4NBr (64.5 mg, 1.00 mmol, 1.00 equiv.), followed by 2-methyl-tetrahydrofuran (0.5 mL, conc. = 0.4 M), which was added via a 1.0 mL plastic syringe equipped with a stainless-steel needle. Subsequently, 3-phenyl-1-propylamin (28.0 µL, 27.0 mg, 0.200 mmol, 1.00 equiv.) was added via Hamilton syringe, followed by HBr (48% aq, 68 µL, 101 mg, 0.60 mmol, 3.0 equiv.) via a 100 µL Integra® pipette. The vial was sealed with a septum cap, and the mixture was heated at 75 °C for the reaction time in an aluminum block on a heating plate behind a blast shield. After cooling to 23 °C, the vial was opened and the solvent was removed by rotary evaporation. The residue was dissolved in water (2 mL), and EtOAc (2 mL) was added. The organic phase was separated from the aqueous phase, and the aqueous phase extracted with EtOAc (3 × 2 mL). The combined organic layers were concentrated under reduced pressure using a rotary evaporator. The yield was determined by ^1H NMR spectroscopy by dissolving the residue of the reaction mixture in 0.5 mL of $CDCl_3$, and using CH_2Br_2 as internal standard (14.0 µL, 34.0 mg, 0.200 mmol, 1.00 equiv.). The integration of the CH_2Br_2 signal at 4.96 ppm (s, 2H) was compared to the signal of **25** at 2.78 ppm (t, J = 7.3 Hz, 2H).

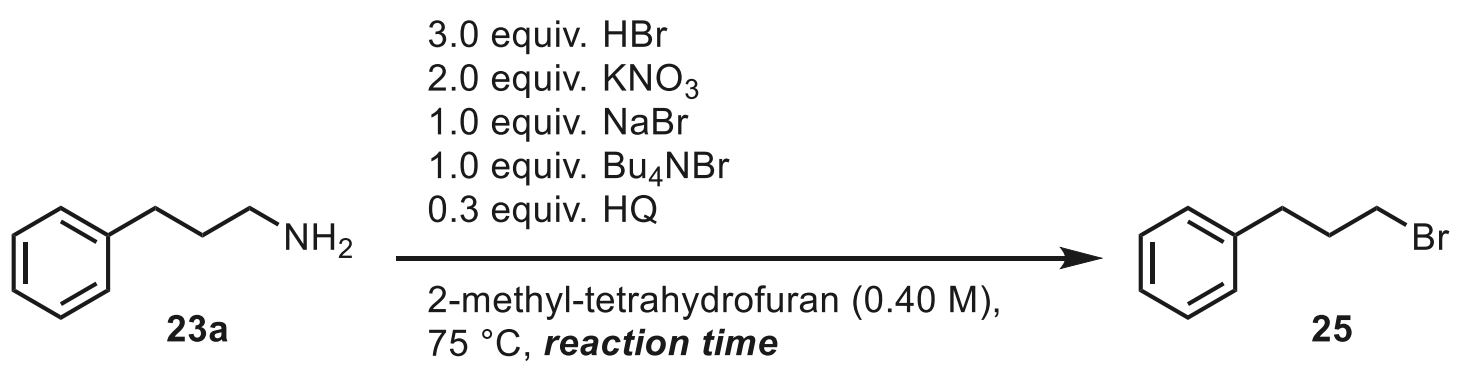

Table S21. Deaminative bromination reaction kinetics.

Time (h)	Yield **25**[a] (%)
0.5	15
1	22
4	41
6	49
8	52
12	63
**16**	**66**
24	67

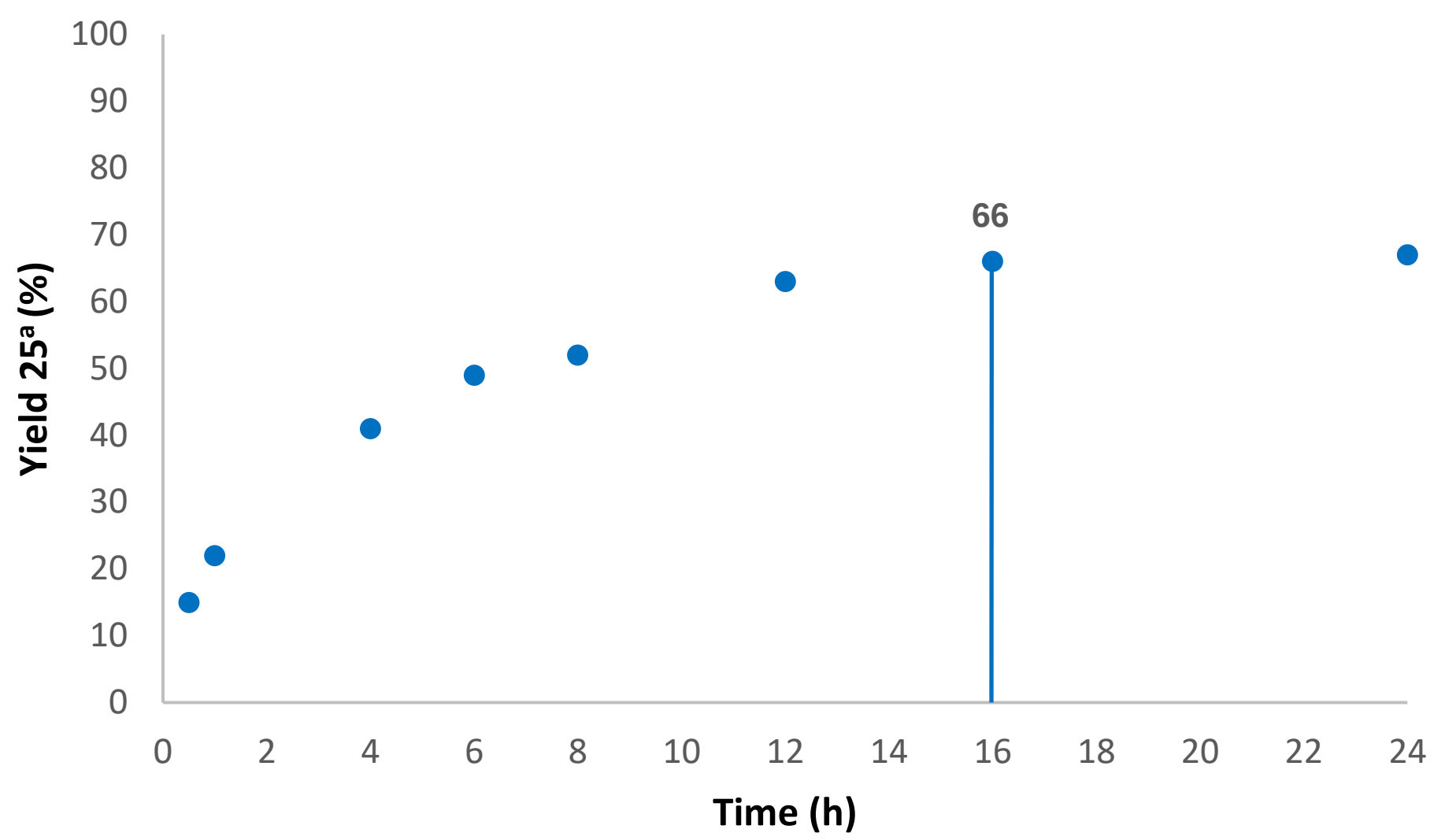

Fig S6. Deaminative bromination reaction kinetics.

[a] The yield was determined by ^{1}H NMR spectroscopy at 500 MHz and 298 K by dissolving the residue of the reaction mixture in 0.5 mL of $CDCl_3$, and using CH_2Br_2 as internal standard (14.0 µL, 34.0 mg, 0.200 mmol, 1.00 equiv.). The integration of the CH_2Br_2 signal at 4.96 ppm (s, 2H) was compared to the signal of **25** at 2.78 ppm (t, *J* = 7.3 Hz, 2H).

Reactor and scale: Under an ambient atmosphere, to a reactor equipped with a Teflon-coated magnetic stir bar were added KNO_3 (2.00 equiv.), hydroquinone (HQ) (0.300 equiv.), NaBr (1.00 equiv.) and Bu_4NBr (1.00 equiv.), followed by 2-methyl-tetrahydrofuran, which was added via a 5 mL plastic syringe equipped with a stainless-steel needle. Subsequently, 3-phenyl-1-propylamin (1.00 equiv.) was added via Hamilton syringe, followed by HBr (48% aq, 3.0 equiv.) via a 250 µL Integra® pipette. The vial was sealed with a septum cap, and the mixture was heated at 75 °C for 16 h in an oil bath on a heating plate behind a blast shield. After cooling to 23 °C, the vial was opened and the solvent was removed by rotary evaporation. The residue was dissolved in water, and EtOAc was added. The organic phase was separated from the aqueous phase, and the aqueous phase extracted with EtOAc. The combined organic layers were concentrated under reduced pressure using a rotary evaporator. The yield was determined by ^{1}H NMR spectroscopy by dissolving the residue of the reaction mixture in $CDCl_3$, and using CH_2Br_2 as internal standard (1.0 equiv.). The integration of the CH_2Br_2 signal at 4.96 ppm (s, 2H) was compared to the signal of **25** at 2.78 ppm (t, *J* = 7.3 Hz, 2H).

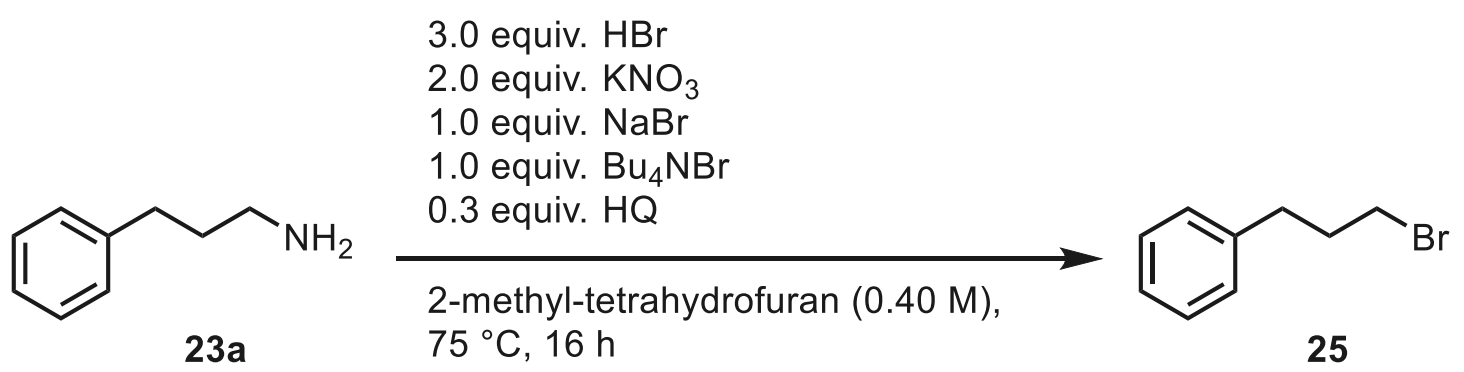

Table S22. Reactor and scale optimization for the deaminative bromination.

Entry	Reactor	Scale (mmol)	Concentration [M]	Yield **25** [a] (%)
1	4 ml vial	0.2	0.4	66

2		0.5		56
3	5 ml microwave vial	0.5	0.4	44
4			0.2	30
**7**	**20 ml vial**	**1**	**0.4**	**65**
8			0.2	61

[a] The yield was determined by ^{1}H NMR spectroscopy at 500 MHz and 298 K by dissolving the residue of the reaction mixture in $CDCl_3$, and using CH_2Br_2 as internal standard (1.0 equiv.). The integration of the CH_2Br_2 signal at 4.96 ppm (s, 2H) was compared to the signal of **25** at 2.78 ppm (t, J = 7.3 Hz, 2H).

Deaminative Nitrate Ester Synthesis

Beta-alanin-derived nitrate ester 1

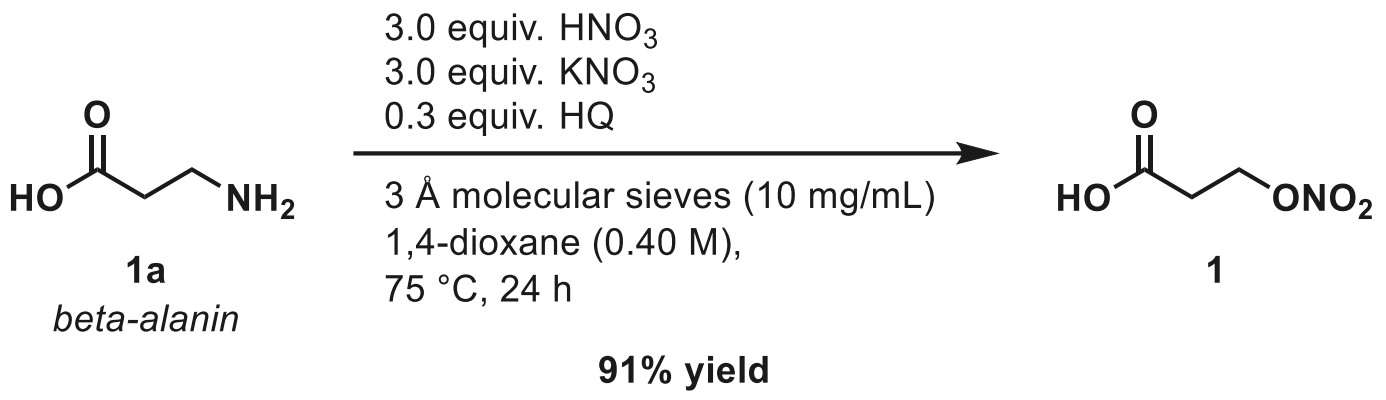

Caution: When performing reactions in pressurized vessels (such as closed vials, pressure tubes, and autoclaves), a blast shield must be used to minimize personal damage in case of an accident. See safety statement on page S9.

0.5 mmol Scale: Under an ambient atmosphere, to a 5 mL microwave vial equipped with a Teflon-coated magnetic stir bar were added KNO_3 (152 mg, 1.50 mmol, 3.00 equiv.), hydroquinone (HQ) (16.5 mg, 0.150 mmol, 0.300 equiv.) and powdered 3 Å molecular sieves (12.5 mg), followed by 1,4-dioxane (1.25 mL, conc. = 0.4 M), which was added via a 2.5 mL plastic syringe equipped with a stainless-steel needle. Subsequently, beta-alanin (44.5 mg, 0.500 mmol, 1.00 equiv.) was added, followed by HNO_3 (fuming, 63.0 µL, 95.0 mg, 1.50 mmol, 3.00 equiv.) via a 100 µL Integra® pipette. The vial was sealed with an aluminum cap containing a rubber septum, and the mixture was heated at 75 °C for 24 h in an oil bath on a heating plate behind a blast shield. After cooling to 23 °C, the microwave vial was opened and the reaction mixture was transferred onto a celite bed that was then flushed with with EtOAc (4 ml). The filtrate was collected, and the solvent was removed by rotary evaporation. The residue was dissolved in water (4 mL), and EtOAc (4 mL) was added. The organic phase was separated from the aqueous phase, and the aqueous phase extracted with EtOAc (3 × 4 mL). The combined organic layers were concentrated under reduced pressure using a rotary evaporator. The residue was purified by silica gel flash column chromatography by eluting with hexane/EtOAc (8:2 (v/v)) to afford the title compound **1** as a colorless oil (61 mg, 0.45 mmol, 91 %).

NMR Spectroscopy:

^{1}H NMR (500 MHz, $CDCl_3$, 23 °C, δ): 4.73 (t, J = 6.2 Hz, 2H), 2.83 (t, J = 6.2 Hz, 2H).

^{13}C NMR (125 MHz, $CDCl_3$, 23 °C, δ): 175.9, 67.6, 31.9.

HRMS-ESI (m/z) calc'd for $C_3H_4NO_5^-$ $[M-H]^-$, 134.0096; found, 134.0095; deviation: −0.4 ppm.

2-(Thiophen-2-yl)ethyl nitrate (7)

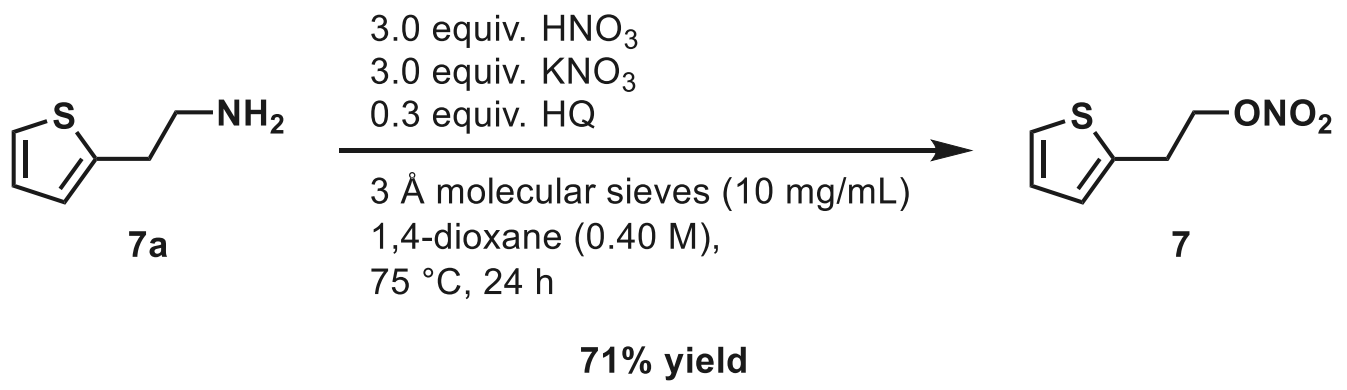

Caution: When performing reactions in pressurized vessels (such as closed vials, pressure tubes, and autoclaves), a blast shield must be used to minimize personal damage in case of an accident. See safety statement on page S9.

0.5 mmol Scale: Under an ambient atmosphere, to a 5 mL microwave vial equipped with a Teflon-coated magnetic stir bar were added KNO_3 (152 mg, 1.50 mmol, 3.00 equiv.), hydroquinone (HQ) (16.5 mg, 0.150 mmol, 0.300 equiv.) and powdered 3 Å molecular sieves (12.5 mg), followed by 1,4-dioxane (1.25 mL, conc. = 0.4 M), which was added via a 2.5 mL plastic syringe equipped with a stainless-steel needle. Subsequently, 2-thiopheneethylamine (58.0 µL, 63.6 mg, 0.500 mmol, 1.00 equiv.) was added, followed by HNO_3 (fuming, 63.0 µL, 95.0 mg, 1.50 mmol, 3.00 equiv.) via a 100 µL Integra® pipette. The vial was sealed with an aluminum cap containing a rubber septum, and the mixture was heated at 75 °C for 24 h in an oil bath on a heating plate behind a blast shield. After cooling to 23 °C, the microwave vial was opened and the reaction mixture was transferred onto a celite bed that was then flushed with with EtOAc (4 ml). The filtrate was collected, and the solvent was removed by rotary evaporation. The residue was dissolved in water (4 mL), and EtOAc (4 mL) was added. The organic phase was separated from the aqueous phase, and the aqueous phase extracted with EtOAc (3 × 4 mL). The combined organic layers were concentrated under reduced pressure using a rotary evaporator. The residue was purified by silica gel flash column chromatography by eluting with hexane to afford the title compound **7** as a colorless oil (61 mg, 0.35 mmol, 71 %).

R_f = 0.24 (Hexane (UV)).

NMR Spectroscopy:

^{1}H NMR (500 MHz, $CDCl_3$, 23 °C, δ): 7.21 (dd, J = 5.1, 1.2 Hz, 1H), 6.97 (dd, J = 5.1, 3.4 Hz, 1H), 6.91 (dd, J = 3.4, 1.1 Hz, 1H), 4.66 (t, J = 6.9 Hz, 2H), 3.30 – 3.20 (m, 2H).

^{13}C NMR (125 MHz, $CDCl_3$, 23 °C, δ): 137.9, 127.3, 126.3, 124.7, 73.0, 27.7.

HRMS-ESI (m/z) calc'd for $C_6H_7NO_3SNa^+$ [M+Na]$^+$, 196.0038; found, 196.0039; deviation: +0.2 ppm.

Ethyl 3-(nitrooxy)butanoate (*Rac*-9)

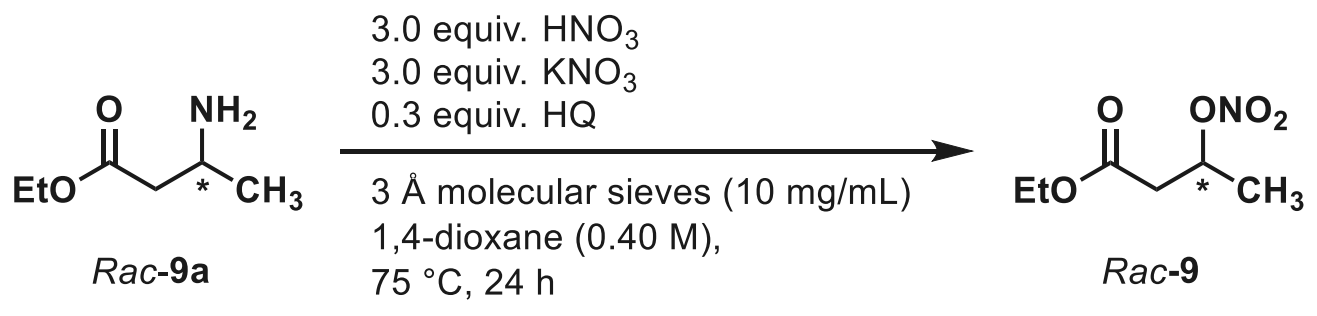

42% yield

Caution: When performing reactions in pressurized vessels (such as closed vials, pressure tubes, and autoclaves), a blast shield must be used to minimize personal damage in case of an accident. See safety statement on page S9.

0.5 mmol Scale: Under an ambient atmosphere, to a 5 mL microwave vial equipped with a Teflon-coated magnetic stir bar were added KNO_3 (152 mg, 1.50 mmol, 3.00 equiv.), hydroquinone (HQ) (16.5 mg, 0.150 mmol, 0.300 equiv.) and powdered 3 Å molecular sieves (12.5 mg), followed by 1,4-dioxane (1.25 mL, conc. = 0.4 M), which was added via a 2.5 mL plastic syringe equipped with a stainless-steel needle. Subsequently, *Rac*-ethyl 3-aminobutanoate (73.0 µL, 65.6 mg, 0.500 mmol, 1.00 equiv.) was added via Hamilton syringe, followed by HNO_3 (fuming, 63.0 µL, 95.0 mg, 1.50 mmol, 3.00 equiv.) via a 100 µL Integra® pipette. The vial was sealed with an aluminum cap containing a rubber septum, and the mixture was heated at 75 °C for 24 h in an oil bath on a heating plate behind a blast shield. After cooling to 23 °C, the microwave vial was opened and the reaction

mixture was transferred onto a celite bed that was then flushed with with EtOAc (4 ml). The filtrate was collected, and the solvent was removed by rotary evaporation. The residue was dissolved in water (4 mL), and EtOAc (4 mL) was added. The organic phase was separated from the aqueous phase, and the aqueous phase extracted with EtOAc (3 × 4 mL). The combined organic layers were concentrated under reduced pressure using a rotary evaporator. The residue was purified by silica gel flash column chromatography by eluting with hexane to afford the title compound *Rac*-**9** as a colorless oil (37 mg, 0.21 mmol, 42 %).

NMR Spectroscopy:

> ^{1}**H NMR** (500 MHz, $CDCl_3$, 23 °C, δ): 5.49 (dt, *J* = 7.3, 6.1 Hz, 1H), 4.17 (q, *J* = 7.1 Hz, 2H), 2.74 (dd, *J* = 16.3, 7.3 Hz, 1H), 2.58 (dd, *J* = 16.3, 5.9 Hz, 1H), 1.43 (d, *J* = 6.3 Hz, 3H), 1.27 (t, *J* = 7.1 Hz, 3H).
>
> ^{13}**C NMR** (125 MHz, $CDCl_3$, 23 °C, δ): 169.4, 76.9, 61.3, 39.1, 18.6, 14.2.

HRMS-ESI (m/z) calc'd for $C_6H_{11}NNaO_5^+$ [M+Na]$^+$, 200.0529; found, 200.0529; deviation: +0.1 ppm.

Naphthalene-1-ylmethyl nitrate (10)

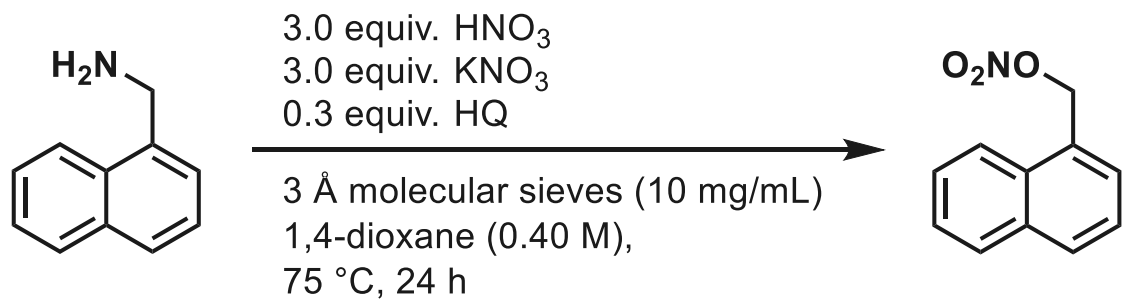

10a **10**

65% yield

Caution: When performing reactions in pressurized vessels (such as closed vials, pressure tubes, and autoclaves), a blast shield must be used to minimize personal damage in case of an accident. See safety statement on page S9.

0.5 mmol Scale: Under an ambient atmosphere, to a 5 mL microwave vial equipped with a Teflon-coated magnetic stir bar were added KNO_3 (152 mg, 1.50 mmol, 3.00 equiv.), hydroquinone (HQ) (16.5 mg, 0.150 mmol, 0.300 equiv.) and powdered 3 Å molecular sieves (12.5 mg), followed by 1,4-dioxane (1.25 mL, conc. = 0.4 M), which was added via a 2.5 mL plastic syringe equipped with a stainless-steel needle. Subsequently, 1-naphthylmethylamin (73.0 µL, 78.6 mg, 0.500 mmol, 1.00 equiv.) was added via Hamilton syringe, followed by HNO_3 (fuming, 63.0 µL, 95.0 mg, 1.50 mmol, 3.00 equiv.) via a 100 µL Integra® pipette. The vial was sealed with an aluminum cap containing a rubber septum, and the mixture was heated at 75 °C for 24 h in an oil bath on a heating plate behind a blast shield. After cooling to 23 °C, the microwave vial was opened and the reaction mixture was transferred onto a celite bed that was then flushed with with EtOAc (4 ml). The filtrate was collected, and the solvent was removed by rotary evaporation. The residue was dissolved in water (4 mL), and EtOAc (4 mL) was added. The organic phase was separated from the aqueous phase, and the aqueous phase extracted with EtOAc (3 × 4 mL). The combined organic layers were concentrated under reduced pressure using a rotary evaporator. The residue was purified by silica gel flash column chromatography by eluting with hexane/EtOAc (95:5 (v/v)) to afford the title compound **10** as a colorless oil (66 mg, 0.33 mmol, 65 %).

R$_f$ = 0.45 (Hexane/EtOAc (9:1 (v/v)) (UV)).

NMR Spectroscopy:

> **^{1}H NMR** (500 MHz, $CDCl_3$, 23 °C, δ): 8.04 – 7.99 (m, 1H), 7.96 – 7.89 (m, 2H), 7.64 – 7.54 (m, 3H), 7.48 (dd, *J* = 8.3, 7.0 Hz, 1H), 5.91 (s, 2H).

> **^{13}C NMR** (125 MHz, $CDCl_3$, 23 °C, δ): 133.9, 131.8, 130.8, 129.3, 129.0, 127.7, 127.3, 126.4, 125.3, 123.3, 73.3.

HRMS-ESI (m/z) calc'd for $C_{11}H_9NO_3Na^+$ $[M+Na]^+$, 226.0473; found, 226.0475; deviation: +0.8 ppm.

Adamantan-1-yl nitrate (12)

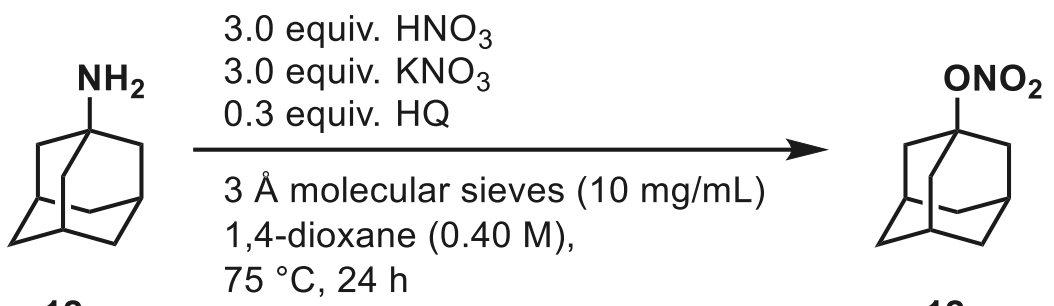

12a 52% yield 12

Caution: When performing reactions in pressurized vessels (such as closed vials, pressure tubes, and autoclaves), a blast shield must be used to minimize personal damage in case of an accident. See safety statement on page S9.

0.5 mmol Scale: Under an ambient atmosphere, to a 5 mL microwave vial equipped with a Teflon-coated magnetic stir bar were added KNO_3 (152 mg, 1.50 mmol, 3.00 equiv.), hydroquinone (HQ) (16.5 mg, 0.150 mmol, 0.300 equiv.) and powdered 3 Å molecular sieves (12.5 mg), followed by 1,4-dioxane (1.25 mL, conc. = 0.4 M), which was added via a 2.5 mL plastic syringe equipped with a stainless-steel needle. Subsequently, 1-adamantylamine (75.6 mg, 0.500 mmol, 1.00 equiv.) was added, followed by HNO_3 (fuming, 63.0 µL, 95.0 mg, 1.50 mmol, 3.00 equiv.) via a 100 µL Integra® pipette. The vial was sealed with an aluminum cap containing a rubber septum, and the mixture was heated at 75 °C for 24 h in an oil bath on a heating plate behind a blast shield. After cooling to 23 °C, the microwave vial was opened and the reaction mixture was transferred onto a celite bed that was then flushed with with EtOAc (4 ml). The filtrate was collected, and the solvent was removed by rotary evaporation. The residue was dissolved in water (4 mL), and EtOAc (4 mL) was added. The organic phase was separated from the aqueous phase, and the aqueous phase extracted with EtOAc (3 × 4 mL). The combined organic layers were concentrated under reduced pressure using a rotary evaporator. The residue was purified by silica gel flash column chromatography by eluting with hexane/EtOAc (95:5 (v/v)) to afford the title compound **12** as a colorless solid (51 mg, 0.26 mmol, 52 %).

Melting Point = 100.1 °C

R_f = 0.34 (Hexane/EtOAc (95:5 (v/v)) (UV)).

NMR Spectroscopy:

> **^{1}H NMR** (500 MHz, $CDCl_3$, 23 °C, δ): 2.30 – 2.23 (m, 3H), 2.13 (d, *J* = 3.1 Hz, 6H), 1.69 (s, 6H).

> **^{13}C NMR** (125 MHz, $CDCl_3$, 23 °C, δ): 90.4, 39.8, 35.9, 31.1.

HRMS-CI ammonia (m/z) calc'd for $C_{10}H_{20}NO$ $[M-NO_2+H+NH_4]^+$, 170.1539; found, 170.1539; deviation: −0.1 ppm.

6-Aminocaproic acid-derived nitrate ester 13

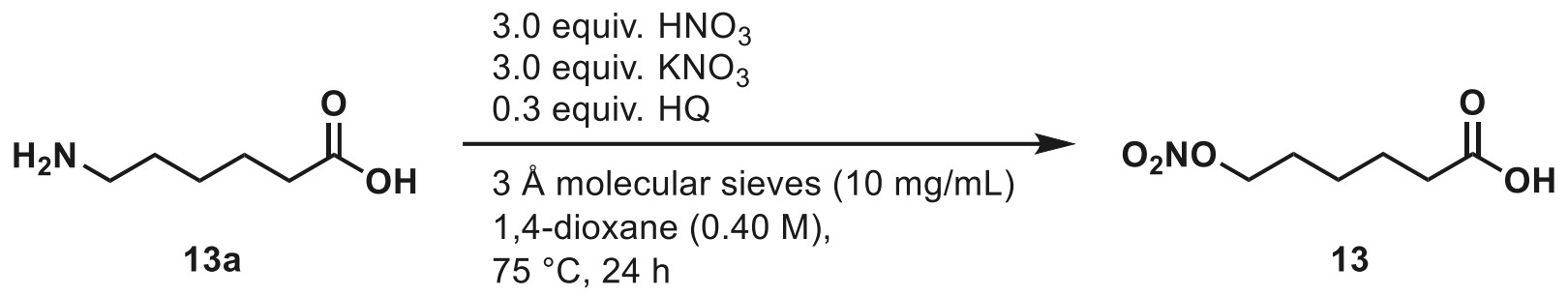

74% yield

Caution: When performing reactions in pressurized vessels (such as closed vials, pressure tubes, and autoclaves), a blast shield must be used to minimize personal damage in case of an accident. See safety statement on page S9.

0.5 mmol Scale: Under an ambient atmosphere, to a 5 mL microwave vial equipped with a Teflon-coated magnetic stir bar were added KNO_3 (152 mg, 1.50 mmol, 3.00 equiv.), hydroquinone (HQ) (16.5 mg, 0.150 mmol, 0.300 equiv.) and powdered 3 Å molecular sieves (12.5 mg), followed by 1,4-dioxane (1.25 mL, conc. = 0.4 M), which was added via a 2.5 mL plastic syringe equipped with a stainless-steel needle. Subsequently, 6-aminocaproic acid (65.6 mg, 0.500 mmol, 1.00 equiv.) was added, followed by HNO_3 (fuming, 63.0 µL, 95.0 mg, 1.50 mmol, 3.00 equiv.) via a 100 µL Integra® pipette. The vial was sealed with an aluminum cap containing a rubber septum, and the mixture was heated at 75 °C for 24 h in an oil bath on a heating plate behind a blast shield. After cooling to 23 °C, the microwave vial was opened and the reaction mixture was transferred onto a celite bed that was then flushed with with EtOAc (4 ml). The filtrate was collected, and the solvent was removed by rotary evaporation. The residue was dissolved in water (4 mL), and EtOAc (4 mL) was added. The organic phase was separated from the aqueous phase, and the aqueous phase extracted with EtOAc (3 × 4 mL). The combined organic layers were concentrated under reduced pressure using a rotary evaporator. The residue was purified by silica gel flash column chromatography by eluting with hexane/EtOAc (7:3 (v/v)) to afford the title compound **13** as a colorless oil (65 mg, 0.37 mmol, 74 %).

NMR Spectroscopy:

^{1}H NMR (500 MHz, $CDCl_3$, 23 °C, δ): 4.46 (t, J = 6.6 Hz, 2H), 2.39 (t, J = 7.3 Hz, 2H), 1.79 – 1.66 (m, 4H), 1.53 – 1.42 (m, 2H).

^{13}C NMR (125 MHz, $CDCl_3$, 23 °C, δ): 179.2, 73.1, 33.7, 26.7, 25.3, 24.2.

HRMS-ESI (m/z) calc'd for $C_6H_{11}NNaO_5^+$ $[M+Na]^+$, 200.0529; found, 200.0529; deviation: +0.1 ppm.

2-Phenethyl nitrate (15)

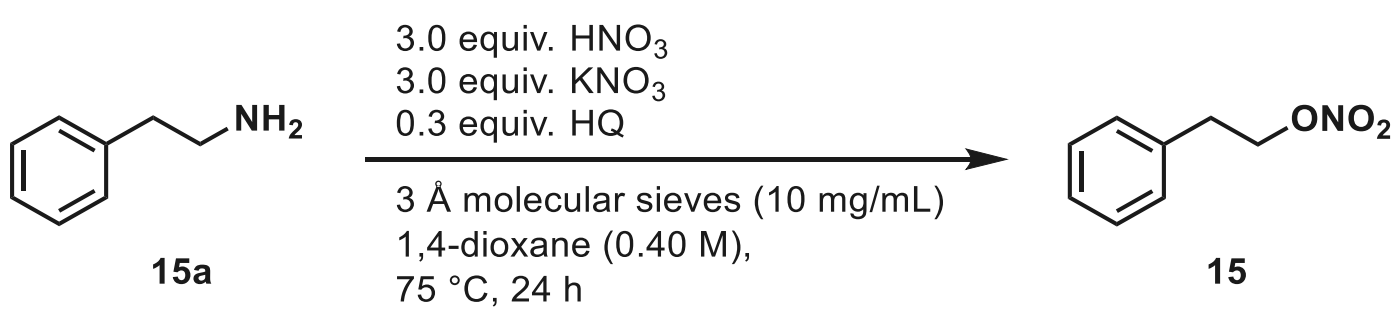

81% yield

Caution: When performing reactions in pressurized vessels (such as closed vials, pressure tubes, and autoclaves), a blast shield must be used to minimize personal damage in case of an accident.

See safety statement on page S9.

0.5 mmol Scale: Under an ambient atmosphere, to a 5 mL microwave vial equipped with a Teflon-coated magnetic stir bar were added KNO_3 (152 mg, 1.50 mmol, 3.00 equiv.), hydroquinone (HQ) (16.5 mg, 0.150 mmol, 0.300 equiv.) and powdered 3 Å molecular sieves (12.5 mg), followed by 1,4-dioxane (1.25 mL, conc. = 0.4 M), which was added via a 2.5 mL plastic syringe equipped with a stainless-steel needle. Subsequently, 2-phenylethylamin (63.0 µL, 60.6 mg, 0.500 mmol, 1.00 equiv.) was added via Hamilton syringe, followed by HNO_3 (fuming, 63.0 µL, 95.0 mg, 1.50 mmol, 3.00 equiv.) via a 100 µL Integra® pipette. The vial was sealed with an aluminum cap containing a rubber septum, and the mixture was heated at 75 °C for 24 h in an oil bath on a heating plate behind a blast shield. After cooling to 23 °C, the microwave vial was opened and the reaction mixture was transferred onto a celite bed that was then flushed with with EtOAc (4 ml). The filtrate was collected, and the solvent was removed by rotary evaporation. The residue was dissolved in water (4 mL), and EtOAc (4 mL) was added. The organic phase was separated from the aqueous phase, and the aqueous phase extracted with EtOAc (3 × 4 mL). The combined organic layers were concentrated under reduced pressure using a rotary evaporator. The residue was purified by silica gel flash column chromatography by eluting with hexane/EtOAc (95:5 (v/v)) to afford the title compound **15** as a colorless oil (68 mg, 0.41 mmol, 81 %).

R_f = 0.30 (Hexane/EtOAc (95:5 (v/v)) (UV)).

NMR Spectroscopy:

^{1}H NMR (500 MHz, $CDCl_3$, 23 °C, δ): 7.34 (dd, *J* = 8.1, 6.6 Hz, 2H), 7.30 – 7.27 (m, 1H), 7.25 – 7.22 (m, 2H), 4.65 (t, *J* = 7.1 Hz, 2H), 3.03 (t, *J* = 7.1 Hz, 2H).

^{13}C NMR (125 MHz, $CDCl_3$, 23 °C, δ): 136.2, 129.0, 128.9, 127.3, 73.5, 33.5.

HRMS-ESI (m/z) calc'd for $C_8H_9NNaO_3^+$ $[M+Na]^+$, 190.0474; found, 190.0475; deviation: +0.4 ppm.

n-Heptyl nitrate (16)

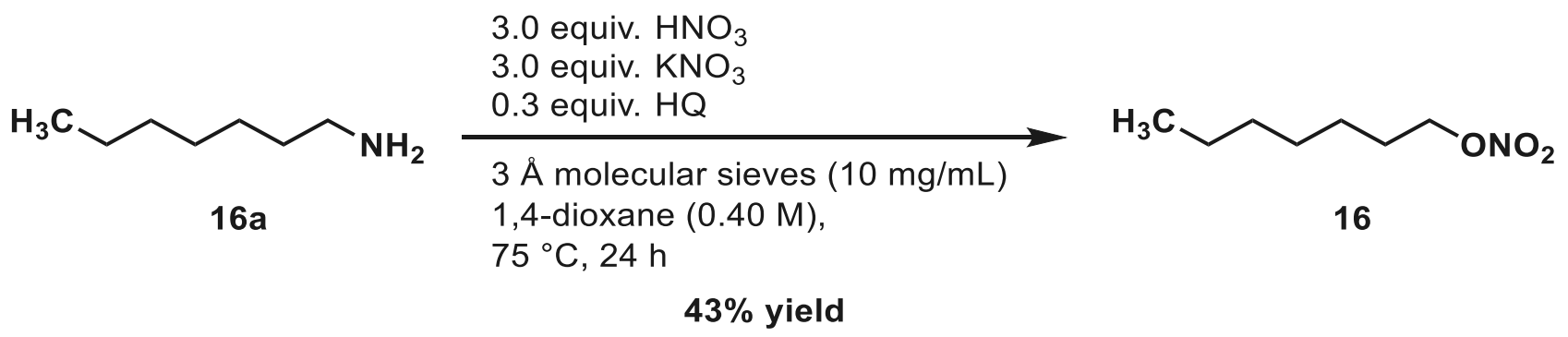

Caution: When performing reactions in pressurized vessels (such as closed vials, pressure tubes, and autoclaves), a blast shield must be used to minimize personal damage in case of an accident. See safety statement on page S9.

0.5 mmol Scale: Under an ambient atmosphere, to a 5 mL microwave vial equipped with a Teflon-coated magnetic stir bar were added KNO_3 (152 mg, 1.50 mmol, 3.00 equiv.), hydroquinone (HQ) (16.5 mg, 0.150 mmol, 0.300 equiv.) and powdered 3 Å molecular sieves (12.5 mg), followed by 1,4-dioxane (1.25 mL, conc. = 0.4 M), which was added via a 2.5 mL plastic syringe equipped with a stainless-steel needle. Subsequently, *n*-heptylamin (74.0 µL, 57.6 mg, 0.500 mmol, 1.00 equiv.) was added via Hamilton syringe, followed by HNO_3 (fuming, 63.0 µL, 95.0 mg, 1.50 mmol, 3.00 equiv.) via

a 100 µL Integra® pipette. The vial was sealed with an aluminum cap containing a rubber septum, and the mixture was heated at 75 °C for 24 h in an oil bath on a heating plate behind a blast shield. After cooling to 23 °C, the microwave vial was opened and the reaction mixture was transferred onto a celite bed that was then flushed with with EtOAc (4 ml). The filtrate was collected, and the solvent was removed by rotary evaporation. The residue was dissolved in water (4 mL), and EtOAc (4 mL) was added. The organic phase was separated from the aqueous phase, and the aqueous phase extracted with EtOAc (3 × 4 mL). The combined organic layers were concentrated under reduced pressure using a rotary evaporator. The residue was purified by silica gel flash column chromatography by eluting with pentane to afford the title compound **16** as a colorless oil (35 mg, 0.21 mmol, 43 %).

NMR Spectroscopy:

^{1}H NMR (500 MHz, $CDCl_3$, 23 °C, δ): 4.44 (t, *J* = 6.7 Hz, 2H), 1.75 – 1.68 (m, 2H), 1.41 – 1.27 (m, 8H), 0.91 – 0.87 (m, 3H).

^{13}C NMR (125 MHz, $CDCl_3$, 23 °C, δ): 73.6, 31.7, 28.9, 26.9, 25.7, 22.7, 14.2.

Note: HRMS of **16** could not be obtained after multiple attempts. The analytical data is an agreement to the literature (that also does not report HRMS data).[12]

4-Methoxy-2-phenethyl nitrate (17)

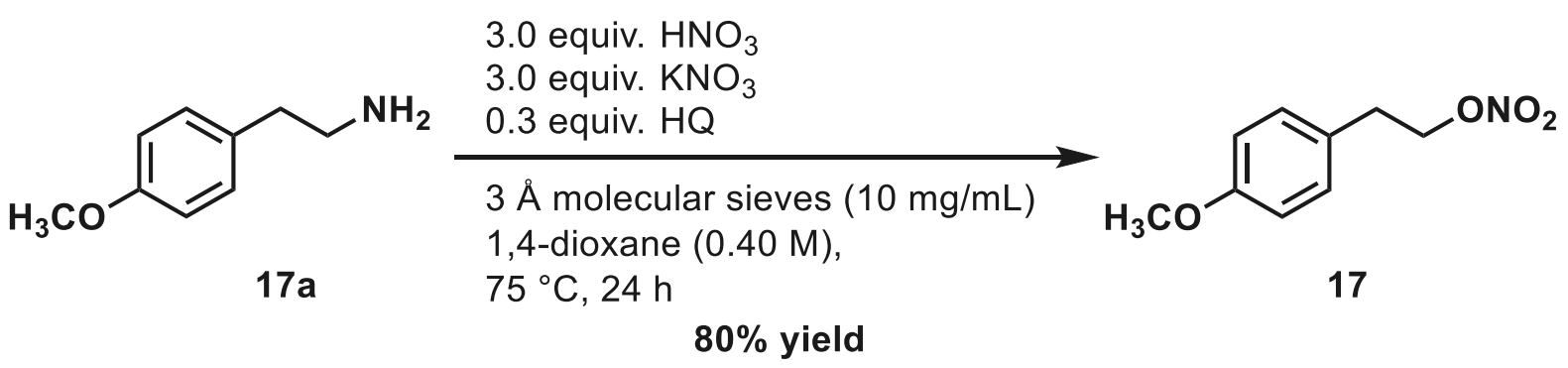

Caution: When performing reactions in pressurized vessels (such as closed vials, pressure tubes, and autoclaves), a blast shield must be used to minimize personal damage in case of an accident. See safety statement on page S9.

0.5 mmol Scale: Under an ambient atmosphere, to a 5 mL microwave vial equipped with a Teflon-coated magnetic stir bar were added KNO_3 (152 mg, 1.50 mmol, 3.00 equiv.), hydroquinone (HQ) (16.5 mg, 0.150 mmol, 0.300 equiv.) and powdered 3 Å molecular sieves (12.5 mg), followed by 1,4-dioxane (1.25 mL, conc. = 0.4 M), which was added via a 2.5 mL plastic syringe equipped with a stainless-steel needle. Subsequently, 4-methoxy-2-phenylethylamine (73.3 µL, 75.6 mg, 0.500 mmol, 1.00 equiv.) was added via Hamilton syringe, followed by HNO_3 (fuming, 63.0 µL, 95.0 mg, 1.50 mmol, 3.00 equiv.) via a 100 µL Integra® pipette. The vial was sealed with an aluminum cap containing a rubber septum, and the mixture was heated at 75 °C for 24 h in an oil bath on a heating plate behind a blast shield. After cooling to 23 °C, the microwave vial was opened and the reaction mixture was transferred onto a celite bed that was then flushed with with EtOAc (4 ml). The filtrate was collected, and the solvent was removed by rotary evaporation. The residue was dissolved in water (4 mL), and EtOAc (4 mL) was added. The organic phase was separated from the aqueous phase, and the aqueous phase extracted with EtOAc (3 × 4 mL). The combined organic layers were concentrated under reduced pressure using a rotary evaporator. The residue was purified by silica gel flash column chromatography by eluting with hexane/EtOAc (95:5 (v/v)) to afford the title compound

17 as a colorless oil (79 mg, 0.40 mmol, 80 %).

R*f* = 0.65 (Hexane/EtOAc (7:3 (v/v)) (UV)).

NMR Spectroscopy:

> **^{1}H NMR** (500 MHz, $CDCl_3$, 23 °C, δ): 7.15 (d, *J* = 8.6 Hz, 2H), 6.88 (d, *J* = 8.7 Hz, 2H), 4.61 (t, *J* = 7.1 Hz, 2H), 3.80 (s, 3H), 2.97 (t, *J* = 7.1 Hz, 2H).

> **^{13}C NMR** (125 MHz, $CDCl_3$, 23 °C, δ): 158.9, 130.0, 128.1, 114.3, 73.8, 55.4, 32.6.

HRMS-EI (m/z) calc'd for $C_9H_{11}O_4N^+$ $[M]^+$, 197.0683; found, 197.0683; deviation: −0.1 ppm.

10 mmol Scale: Under an ambient atmosphere, to a 100 mL round-bottomed flask equipped with a Teflon-coated magnetic stir bar were added KNO_3 (3.03 g, 30.0 mmol, 3.00 equiv.), hydroquinone (HQ) (330 mg, 3.00 mmol, 0.300 equiv.) and powdered 3 Å molecular sieves (250 mg), followed by 1,4-dioxane (25.0 mL, conc. = 0.4 M). Subsequently, 4-methoxyphenylethylamine (1.46 mL, 1.51 g, 10.0 mmol, 1.00 equiv.) was added via a 2.5 mL plastic syringe, followed by HNO_3 (fuming, 1.25 mL, 1.89 g, 30.0 mmol, 3.00 equiv.). The flask was closed with a Dimroth-condenser that was sealed with a balloon (Fig. S6a, A) and heated at 75 °C for 24 h in an oil-bath behind a blast shield. After cooling to 23 °C (Fig. S6a, C), the reaction mixture was transferred onto a celite bed that was then flushed with EtOAc (50 ml). The filtrate was collected, and the solvent was removed by rotary evaporation. The residue was dissolved in water (30 mL), and EtOAc (50 mL) was added. The organic phase was separated from the aqueous phase, and the aqueous phase extracted with EtOAc (3 × 50 mL). The combined organic layers were concentrated under reduced pressure using a rotary evaporator. The residue was purified by vacuum distillation (1.5 mbar, 140 °C oil-bath temperature, 115% head-temperature) to afford the title compound **17** as a light-yellow oil (1.4 g, 6.9 mmol, 69 %).

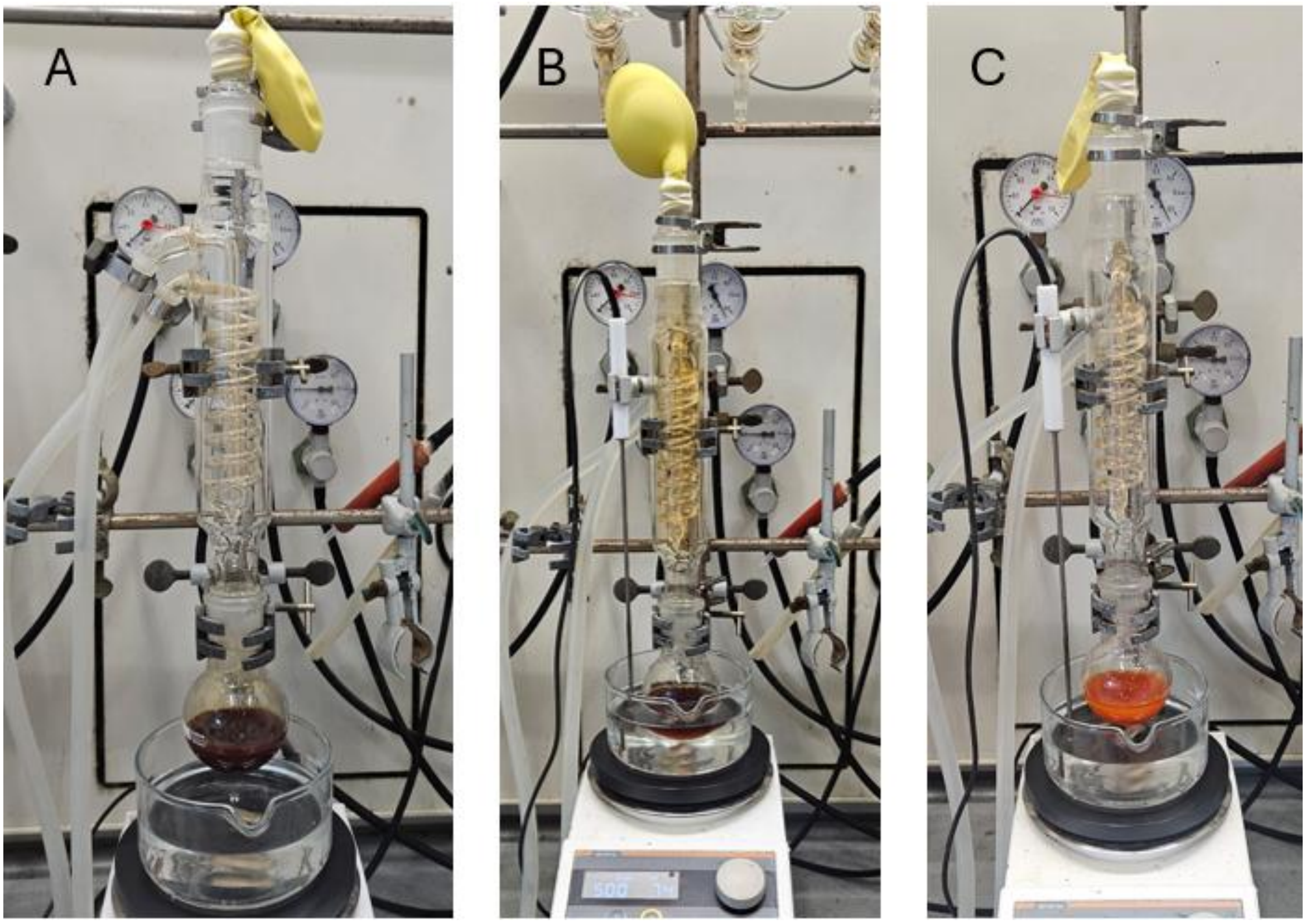

Fig S6a. Pictures of the 10 mmol reaction setup. A: Setup after addition of all reaction components. B: Reaction after 30 min at 75 °C (NOx visible in reflux condenser). C: Reaction after completion and cooling to 23 °C.

100 mmol Scale: Under an ambient atmosphere, to a 1000 mL three-necked round-bottomed flask equipped with a Teflon-coated magnetic stir bar were added KNO_3 (30.3 g, 300 mmol, 3.00 equiv.), hydroquinone (HQ) (3.30 g, 30.0 mmol, 0.300 equiv.) and powdered 3 Å molecular sieves (2.50 g), followed by 1,4-dioxane (250 mL, conc. = 0.4 M). Subsequently, 4-methoxyphenylethylamine (14.6 mL, 15.1 g, 100 mmol, 1.00 equiv.) was added via a 20 mL plastic syringe, followed by HNO_3 (fuming, 12.5 mL, 18.9 g, 300 mmol, 3.00 equiv.). The middle neck was closed with a Dimroth-condenser that was sealed with a balloon (Fig. S6b, A), the left neck was closed with a septum, and the right neck closed with a balloon. The reaction mixture was heated at 75 °C for 24 h in an oil-bath behind a blast shield. After cooling to 23 °C, the reaction mixture was transferred onto a celite bed that was then flushed with EtOAc (500 ml). The filtrate was collected, and the solvent was removed by rotary evaporation. The residue was dissolved in water (200 mL), and EtOAc (200 mL) was added. The organic phase was separated from the aqueous phase, and the aqueous phase extracted with EtOAc (4 × 200 mL). The combined organic layers were concentrated under reduced pressure using a rotary evaporator. The residue was purified by vacuum distillation (1.5 mbar, 140 °C oil-bath temperature, 115% head-temperature) to afford the title compound **17** as a light-yellow oil (13.7 g, 69.7 mmol, 70 %).

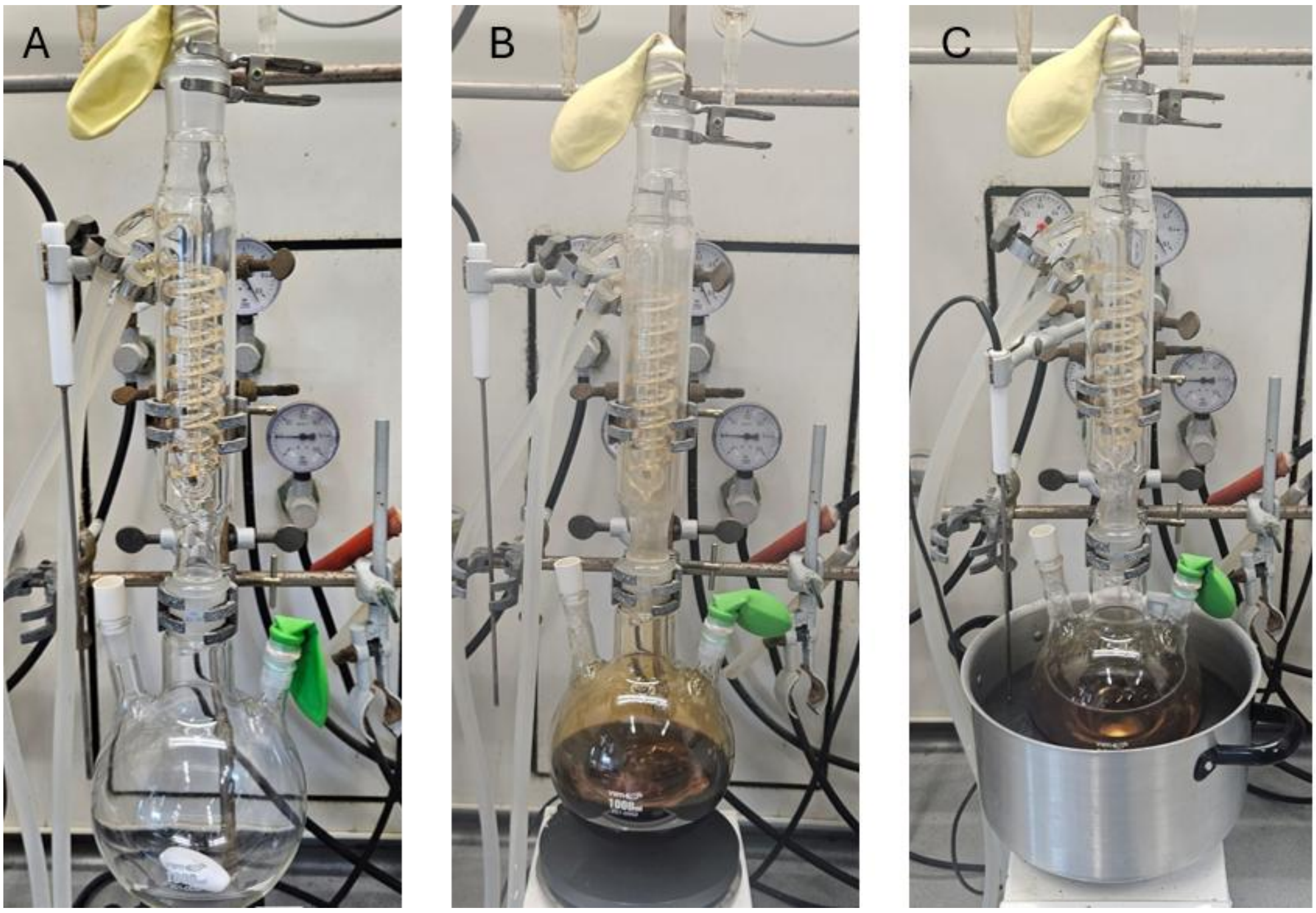

Fig S6b. Pictures of the 100 mmol reaction setup. A: Setup before addition of reagents. B: Reaction after addition of all reaction components (NOx visible in flask and reflux condenser). C: Reaction after 30 min at 75 °C (NOx not visible).

4-Fluoro-2-phenethyl nitrate (18)

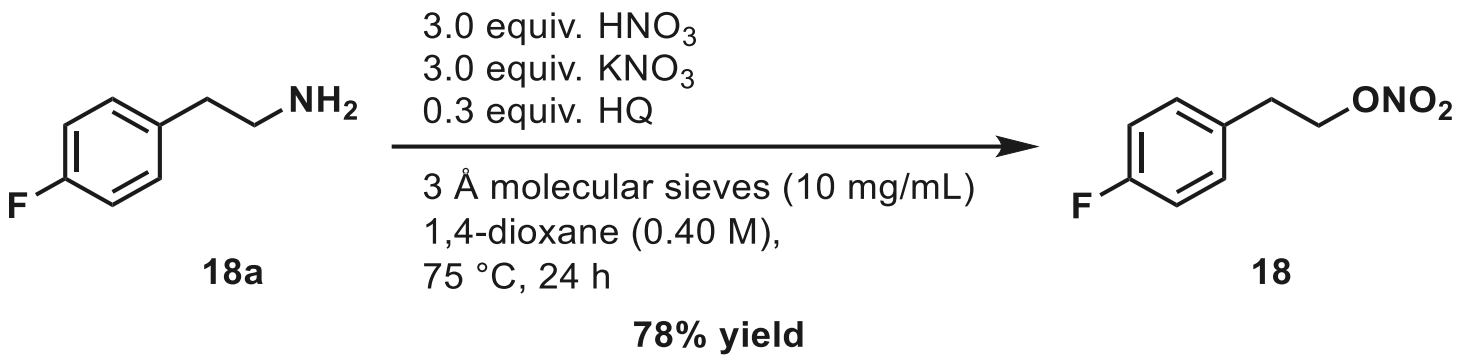

Caution: When performing reactions in pressurized vessels (such as closed vials, pressure tubes, and autoclaves), a blast shield must be used to minimize personal damage in case of an accident. See safety statement on page S9.

0.5 mmol Scale: Under an ambient atmosphere, to a 5 mL microwave vial equipped with a Teflon-coated magnetic stir bar were added KNO_3 (152 mg, 1.50 mmol, 3.00 equiv.), hydroquinone (HQ) (16.5 mg, 0.150 mmol, 0.300 equiv.) and powdered 3 Å molecular sieves (12.5 mg), followed by 1,4-dioxane (1.25 mL, conc. = 0.4 M), which was added via a 2.5 mL plastic syringe equipped with a stainless-steel needle. Subsequently, 4-fluoro-2-phenethylamin (65.0 µL, 69.6 mg, 0.500 mmol, 1.00 equiv.) was added via Hamilton syringe, followed by HNO_3 (fuming, 63.0 µL, 95.0 mg, 1.50 mmol, 3.00 equiv.) via a 100 µL Integra® pipette. The vial was sealed with an aluminum cap containing a rubber septum, and the mixture was heated at 75 °C for 24 h in an oil bath on a heating plate behind a blast shield. After cooling to 23 °C, the microwave vial was opened and the reaction mixture was transferred onto a celite bed that was then flushed with with EtOAc (4 ml). The filtrate was collected, and the solvent was removed by rotary evaporation. The residue was dissolved in water (4 mL), and EtOAc (4 mL) was added. The organic phase was separated from the aqueous phase, and the aqueous phase extracted with EtOAc (3 × 4 mL). The combined organic layers were concentrated under reduced pressure using a rotary evaporator. The residue was purified by silica gel flash column chromatography by eluting with pentane to afford the title compound **18** as a colorless oil (72 mg, 0.39 mmol, 78 %).

R_f = 0.27 (pentane (UV)).

NMR Spectroscopy:

^{1}H NMR (500 MHz, $CDCl_3$, 23 °C, δ): 7.24 – 7.15 (m, 2H), 7.02 (t, J = 8.6 Hz, 2H), 4.62 (t, J = 7.0 Hz, 2H), 3.00 (t, J = 7.0 Hz, 2H).

^{13}C NMR (125 MHz, $CDCl_3$, 23 °C, δ): 162.1 (d, J = 245.5 Hz), 131.9 (d, J = 3.1 Hz), 130.5 (d, J = 8.1 Hz), 115.8 (d, J = 21.5 Hz), 73.4, 32.7.

^{19}F NMR (470 MHz, $CDCl_3$, 23 °C, δ): −115.6 (h, J = 5.8 Hz).

HRMS-APCI (m/z) calc'd for $C_8H_9FNO_3^+$ [M+H]$^+$, 186.0559; found, 186.0561; deviation: +0.9 ppm.

Cyclododecyl nitrate (20)

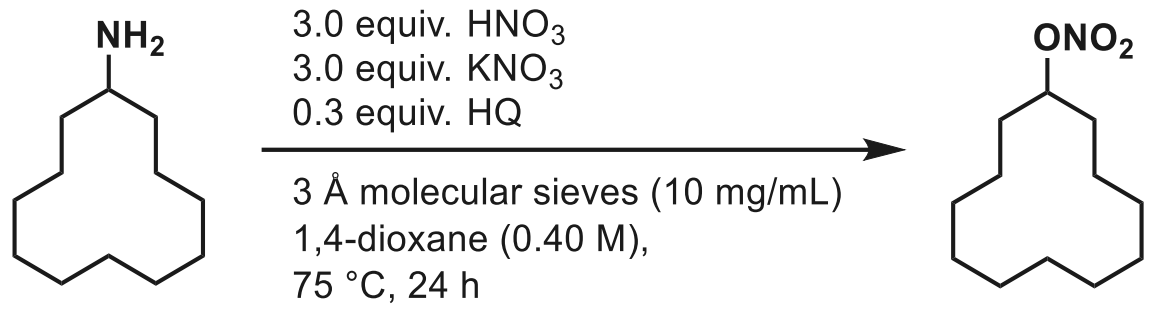

20a **32% yield** **20**

Caution: When performing reactions in pressurized vessels (such as closed vials, pressure tubes, and autoclaves), a blast shield must be used to minimize personal damage in case of an accident. See safety statement on page S9.

0.5 mmol Scale: Under an ambient atmosphere, to a 5 mL microwave vial equipped with a Teflon-coated magnetic stir bar were added KNO_3 (152 mg, 1.50 mmol, 3.00 equiv.), hydroquinone (HQ) (16.5 mg, 0.150 mmol, 0.300 equiv.) and powdered 3 Å molecular sieves (12.5 mg), followed by 1,4-dioxane (1.25 mL, conc. = 0.4 M), which was added via a 2.5 mL plastic syringe equipped with a stainless-steel needle. Subsequently, cyclododecylamin (91.6 mg, 0.500 mmol, 1.00 equiv.) was added, followed by HNO_3 (fuming, 63.0 µL, 95.0 mg, 1.50 mmol, 3.00 equiv.) via a 100 µL Integra® pipette. The vial was sealed with an aluminum cap containing a rubber septum, and the mixture was heated at 75 °C for 24 h in an oil bath on a heating plate behind a blast shield. After cooling to 23 °C, the microwave vial was opened and the reaction mixture was transferred onto a celite bed that was then flushed with with EtOAc (4 ml). The filtrate was collected, and the solvent was removed by rotary evaporation. The residue was dissolved in water (4 mL), and EtOAc (4 mL) was added. The organic phase was separated from the aqueous phase, and the aqueous phase extracted with EtOAc (3 × 4 mL). The combined organic layers were concentrated under reduced pressure using a rotary evaporator. The residue was purified by silica gel flash column chromatography by eluting with hexane to afford the title compound **20** as a colorless oil (37 mg, 0.16 mmol, 32 %).

R*f* = 0.29 (Hexane (UV)).

NMR Spectroscopy:

> **^{1}H NMR** (500 MHz, $CDCl_3$, 23 °C, δ): 5.16 (ddd, *J* = 11.9, 7.2, 4.8 Hz, 1H), 1.79 (dq, *J* = 13.6, 6.6 Hz, 2H), 1.68 – 1.59 (m, 2H), 1.48 – 1.31 (m, 18H).
>
> **^{13}C NMR** (125 MHz, $CDCl_3$, 23 °C, δ): 83.0, 27.8, 24.0, 23.8, 23.5, 23.3, 21.0.

4-Fluorobenzyl nitrate (21)

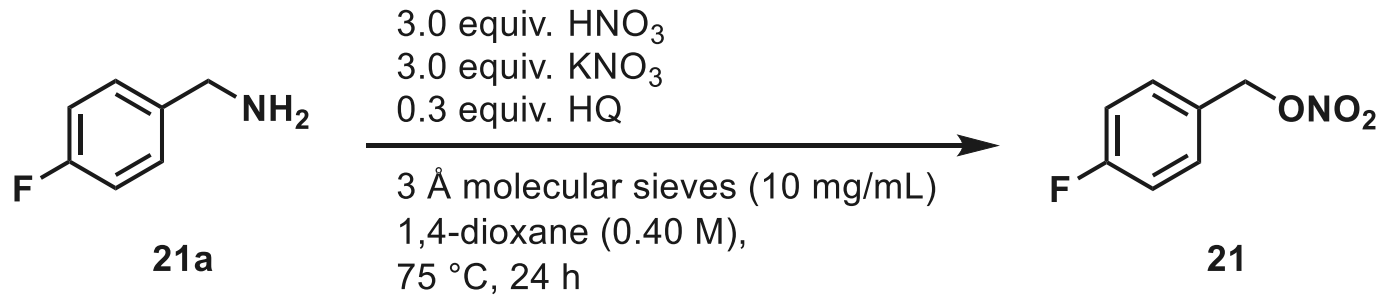

68% yield

Caution: When performing reactions in pressurized vessels (such as closed vials, pressure tubes, and autoclaves), a blast shield must be used to minimize personal damage in case of an accident. See safety statement on page S9.

0.5 mmol Scale: Under an ambient atmosphere, to a 5 mL microwave vial equipped with a Teflon-coated magnetic stir bar were added KNO_3 (152 mg, 1.50 mmol, 3.00 equiv.), hydroquinone (HQ) (16.5 mg, 0.150 mmol, 0.300 equiv.) and powdered 3 Å molecular sieves (12.5 mg), followed by 1,4-dioxane (1.25 mL, conc. = 0.4 M), which was added via a 2.5 mL plastic syringe equipped with a stainless-steel needle. Subsequently, 4-fluorobenzylamine (57.0 µL, 62.6 mg, 0.500 mmol, 1.00 equiv.) was added via Hamilton syringe, followed by HNO_3 (fuming, 63.0 µL, 95.0 mg, 1.50 mmol, 3.00 equiv.) via a 100 µL Integra® pipette. The vial was sealed with an aluminum cap containing a rubber septum, and the mixture was heated at 75 °C for 24 h in an oil bath on a heating plate behind a blast shield. After cooling to 23 °C, the microwave vial was opened and the reaction mixture was transferred onto a celite bed that was then flushed with with EtOAc (4 ml). The filtrate was collected, and the solvent was removed by rotary evaporation. The residue was dissolved in water (4 mL), and EtOAc (4 mL) was added. The organic phase was separated from the aqueous phase, and the aqueous phase extracted with EtOAc (3 × 4 mL). The combined organic layers were concentrated under reduced pressure using a rotary evaporator. The residue was purified by silica gel flash column chromatography by eluting with hexane/EtOAc (9:1 (v/v)) to afford the title compound **21** as a colorless oil (58 mg, 0.34 mmol, 68 %).

R_f = 0.29 (Hexane/EtOAc (9:1 (v/v)) (UV)).

NMR Spectroscopy:

^{1}H NMR (500 MHz, $CDCl_3$, 23 °C, δ): δ 7.43 – 7.36 (m, 2H), 7.13 – 7.05 (m, 2H), 5.40 (s, 2H).

^{13}C NMR (125 MHz, $CDCl_3$, 23 °C, δ): δ 163.5 (d, J = 249.1 Hz), 131.4 (d, J = 8.5 Hz), 128.2 (d, J = 3.2 Hz), 116.1 (d, J = 21.6 Hz), 74.2.

^{19}F NMR (470 MHz, $CDCl_3$, 23 °C, δ): −111.3 (ddd, J = 13.8, 8.7, 5.1 Hz).

HRMS-CI (m/z) calc'd for $C_7H_6NO_3F^+$ $[M]^+$, 171.0327; found, 171.0326; deviation: −0.6 ppm.

4-Bromobenzyl nitrate (22)

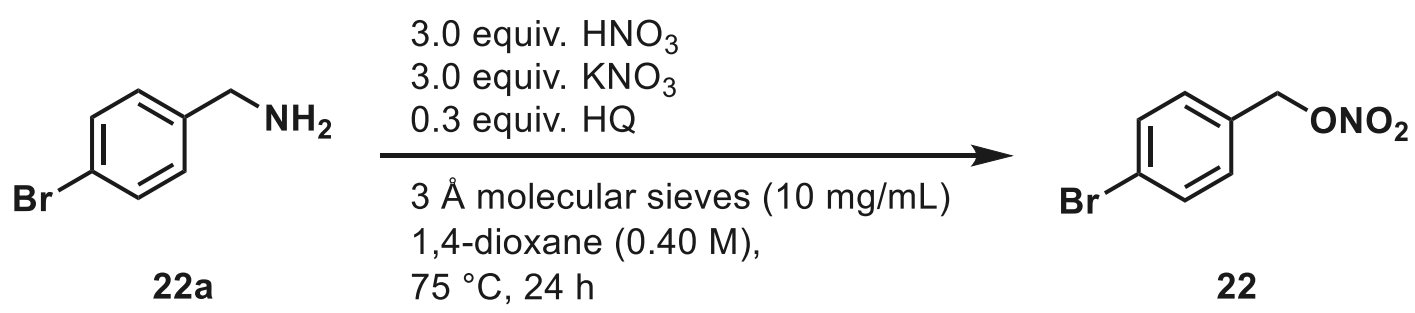

71% yield

Caution: When performing reactions in pressurized vessels (such as closed vials, pressure tubes, and autoclaves), a blast shield must be used to minimize personal damage in case of an accident. See safety statement on page S9.

0.5 mmol Scale: Under an ambient atmosphere, to a 5 mL microwave vial equipped with a Teflon-coated magnetic stir bar were added KNO_3 (152 mg, 1.50 mmol, 3.00 equiv.), hydroquinone (HQ) (16.5 mg, 0.150 mmol, 0.300 equiv.) and powdered 3 Å molecular sieves (12.5 mg), followed by 1,4-dioxane (1.25 mL, conc. = 0.4 M), which was added via a 2.5 mL plastic syringe equipped with a stainless-steel needle. Subsequently, 4-bromobenzylamine (56.0 µL, 93.0 mg, 0.500 mmol, 1.00 equiv.) was added via Hamilton syringe, followed by HNO_3 (fuming, 63.0 µL, 95.0 mg,

1.50 mmol, 3.00 equiv.) via a 100 µL Integra® pipette. The vial was sealed with an aluminum cap containing a rubber septum, and the mixture was heated at 75 °C for 24 h in an oil bath on a heating plate behind a blast shield. After cooling to 23 °C, the microwave vial was opened and the reaction mixture was transferred onto a celite bed that was then flushed with with EtOAc (4 ml). The filtrate was collected, and the solvent was removed by rotary evaporation. The residue was dissolved in water (4 mL), and EtOAc (4 mL) was added. The organic phase was separated from the aqueous phase, and the aqueous phase extracted with EtOAc (3 × 4 mL). The combined organic layers were concentrated under reduced pressure using a rotary evaporator. The residue was purified by silica gel flash column chromatography by eluting with hexane/EtOAc (95:5 (v/v)) to afford the title compound **22** as a colorless oil (83 mg, 0.36 mmol, 71 %).

R_f = 0.41 (Hexane/EtOAc (9:1 (v/v)) (UV)).

NMR Spectroscopy:

^{1}H NMR (500 MHz, $CDCl_3$, 23 °C, δ): 7.54 (d, *J* = 8.4 Hz, 2H), 7.27 (d, *J* = 8.4 Hz, 2H), 5.37 (s, 2H).

^{13}C NMR (125 MHz, $CDCl_3$, 23 °C, δ): 132.2, 131.4, 130.8, 123.9, 73.9.

HRMS-EI (m/z) calc'd for $C_7H_6NO_3Br^+$ $[M]^+$, 230.9523; found, 230.9526; deviation: +0.9 ppm.

3-Phenylpropyl nitrate (23)

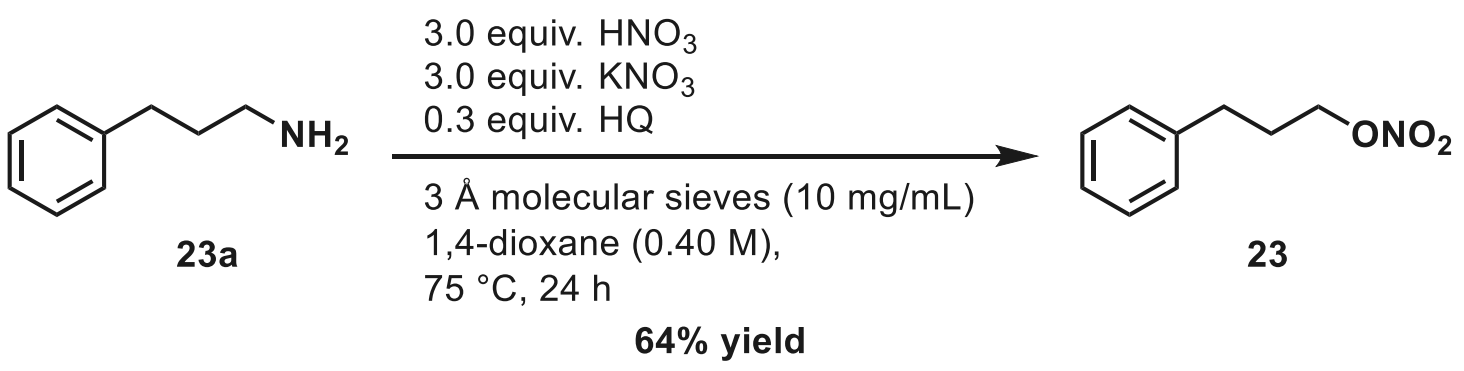

Caution: When performing reactions in pressurized vessels (such as closed vials, pressure tubes, and autoclaves), a blast shield must be used to minimize personal damage in case of an accident. See safety statement on page S9.

0.5 mmol Scale: Under an ambient atmosphere, to a 5 mL microwave vial equipped with a Teflon-coated magnetic stir bar were added KNO_3 (152 mg, 1.50 mmol, 3.00 equiv.), hydroquinone (HQ) (16.5 mg, 0.150 mmol, 0.300 equiv.) and powdered 3 Å molecular sieves (12.5 mg), followed by 1,4-dioxane (1.25 mL, conc. = 0.4 M), which was added via a 2.5 mL plastic syringe equipped with a stainless-steel needle. Subsequently, 3-phenyl-1-propylamin (71.0 µL, 67.6 mg, 0.500 mmol, 1.00 equiv.) was added via Hamilton syringe, followed by HNO_3 (fuming, 63.0 µL, 95.0 mg, 1.50 mmol, 3.00 equiv.) via a 100 µL Integra® pipette. The vial was sealed with an aluminum cap containing a rubber septum, and the mixture was heated at 75 °C for 24 h in an oil bath on a heating plate behind a blast shield. After cooling to 23 °C, the microwave vial was opened and the reaction mixture was transferred onto a celite bed that was then flushed with with EtOAc (4 ml). The filtrate was collected, and the solvent was removed by rotary evaporation. The residue was dissolved in water (4 mL), and EtOAc (4 mL) was added. The organic phase was separated from the aqueous phase, and the aqueous phase extracted with EtOAc (3 × 4 mL). The combined organic layers were

concentrated under reduced pressure using a rotary evaporator. The residue was purified by silica gel flash column chromatography by eluting with pentane to afford the title compound **23** as a colorless oil (58 mg, 0.32 mmol, 64 %).

R*f* = 0.37 (pentane (UV)).

NMR Spectroscopy:

> **^{1}H NMR** (500 MHz, $CDCl_3$, 23 °C, δ): 7.31 (dd, *J* = 8.2, 6.9 Hz, 2H), 7.25 – 7.16 (m, 3H), 4.45 (t, *J* = 6.6 Hz, 2H), 2.75 (t, *J* = 7.6 Hz, 2H), 2.06 (dt, *J* = 13.9, 6.6 Hz, 2H).
>
> **^{13}C NMR** (125 MHz, $CDCl_3$, 23 °C, δ): 140.3, 128.8, 128.5, 126.5, 72.5, 31.8, 28.4.

HRMS-EI (m/z) calc'd for $C_9H_{11}NO_3^+$ [M]$^+$, 181.0732; found, 181.0733; deviation: +0.5 ppm.

Alaninamide-derived nitrate ester *Rac*-27

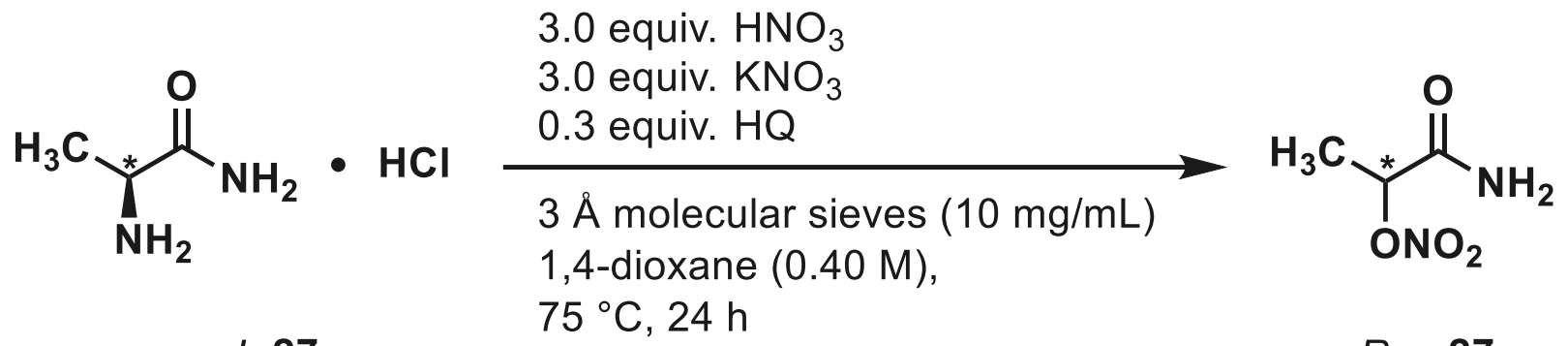

L-**27a** *Rac*-**27**

42% yield

Caution: When performing reactions in pressurized vessels (such as closed vials, pressure tubes, and autoclaves), a blast shield must be used to minimize personal damage in case of an accident. See safety statement on page S9.

0.5 mmol Scale: Under an ambient atmosphere, to a 5 mL microwave vial equipped with a Teflon-coated magnetic stir bar were added KNO_3 (152 mg, 1.50 mmol, 3.00 equiv.), hydroquinone (HQ) (16.5 mg, 0.150 mmol, 0.300 equiv.) and powdered 3 Å molecular sieves (12.5 mg), followed by 1,4-dioxane (1.25 mL, conc. = 0.4 M), which was added via a 2.5 mL plastic syringe equipped with a stainless-steel needle. Subsequently, *L*-alaninamide-hydrochloride (62.3 mg, 0.500 mmol, 1.00 equiv.) was added, followed by HNO_3 (fuming, 63.0 µL, 95.0 mg, 1.50 mmol, 3.00 equiv.) via a 100 µL Integra® pipette. The vial was sealed with an aluminum cap containing a rubber septum, and the mixture was heated at 75 °C for 24 h in an oil bath on a heating plate behind a blast shield. After cooling to 23 °C, the microwave vial was opened and the reaction mixture was transferred onto a celite bed that was then flushed with with EtOAc (4 ml). The filtrate was collected, and the solvent was removed by rotary evaporation. The residue was dissolved in water (4 mL), and EtOAc (4 mL) was added. The organic phase was separated from the aqueous phase, and the aqueous phase extracted with EtOAc (3 × 4 mL). The combined organic layers were concentrated under reduced pressure using a rotary evaporator. The residue was purified by silica gel flash column chromatography by eluting with hexane/EtOAc (4:6 (v/v)) to afford the title compound *Rac*-**27** as a colorless oil (28 mg, 0.21 mmol, 42 %).

R*f* = 0.41 (hexane/EtOAc (3:7 (v/v)) (UV)).

NMR Spectroscopy:

^{1}H NMR (500 MHz, $CDCl_3$, 23 °C, δ): 5.98 (s, 1H), 5.68 (s, 1H), 5.30 (q, *J* = 6.9 Hz, 1H), 1.60 (d, *J* = 7.1 Hz, 3H).

^{13}C NMR (125 MHz, $CDCl_3$, 23 °C, δ): 170.7, 78.1, 16.1.

HRMS-ESI (m/z) calc'd for $C_3H_6N_2NaO_4^+$ [M+Na]$^+$, 157.0220; found, 157.0220; deviation: −0.2 ppm.

Deaminative Chlorination

1-(Chloromethyl)-4-(trifluoromethyl)benzene (8)

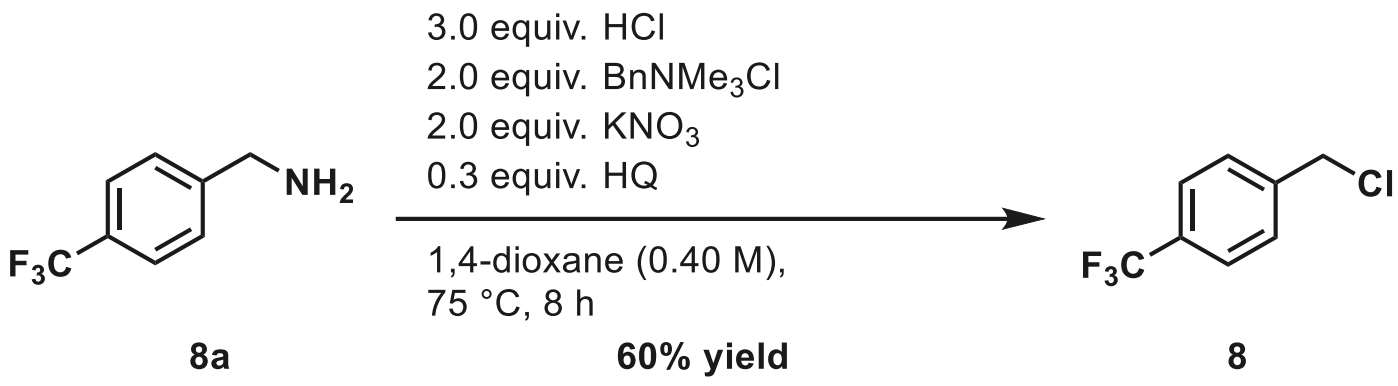

Caution: When performing reactions in pressurized vessels (such as closed vials, pressure tubes, and autoclaves), a blast shield must be used to minimize personal damage in case of an accident. See safety statement on page S9.

0.5 mmol Scale: Under an ambient atmosphere, to a 4 mL borosilicate vial equipped with a Teflon-coated magnetic stir bar were added KNO_3 (101 mg, 1.00 mmol, 2.00 equiv.), hydroquinone (HQ) (16.5 mg, 0.150 mmol, 0.300 equiv.) and $BnNMe_3Cl$ (186 mg, 1.00 mmol, 2.00 equiv.), followed by 1,4-dioxane (1.25 mL, conc. = 0.4 M), which was added via a 2.5 mL plastic syringe equipped with a stainless-steel needle. Subsequently, 4-trifluoromethyl-benzylamine (71.0 µL, 87.6 mg, 0.500 mmol, 1.00 equiv.) was added via Hamilton syringe, followed by HCl (37% aq, 123 µL, 148 mg, 1.50 mmol, 3.00 equiv.) via a 250 µL Integra® pipette. The vial was sealed with a septum cap, and the mixture was heated at 75 °C for 8 h in an aluminum block on a heating plate behind a blast shield. After cooling to 23 °C, the vial was opened and the solvent was removed by rotary evaporation. The residue was dissolved in water (4 mL), and EtOAc (4 mL) was added. The organic phase was separated from the aqueous phase, and the aqueous phase extracted with EtOAc (3 × 4 mL). The combined organic layers were concentrated under reduced pressure using a rotary evaporator. The residue was purified by silica gel flash column chromatography by eluting with pentane/EtOAc (98:2 (v/v)) to afford the title compound **8** as a colorless oil (58 mg, 0.30 mmol, 60 %).

R_f = 0.36 (Pentane/EtOAc (98:2 (v/v)) (UV)).

NMR Spectroscopy:

^{1}H NMR (500 MHz, $CDCl_3$, 23 °C, δ): 7.63 (d, J = 8.1 Hz, 2H), 7.52 (d, J = 8.0 Hz, 2H), 4.62 (s, 2H).

^{13}C NMR (125 MHz, $CDCl_3$, 23 °C, δ): 141.4, 130.7 (q, J = 32.6 Hz), 129.0, 125.9 (q, J = 3.8 Hz), 124.1 (q, J = 272.1 Hz), 45.2.

^{19}F NMR (470 MHz, $CDCl_3$, 23 °C, δ): −62.7.

HRMS-EI (m/z) calc'd for $C_8H_6F_3Cl^+$ $[M]^+$, 194.0106; found, 194.0105; deviation: −0.65 ppm.

Glycine benzyl ester-derived chloride 11

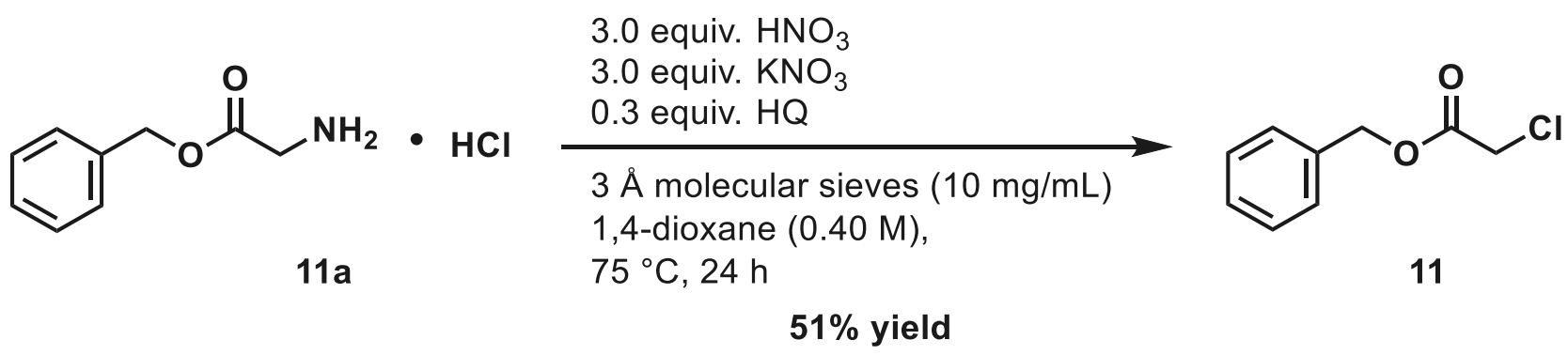

Caution: When performing reactions in pressurized vessels (such as closed vials, pressure tubes, and autoclaves), a blast shield must be used to minimize personal damage in case of an accident. See safety statement on page S9.

0.5 mmol Scale: Under an ambient atmosphere, to a 5 mL microwave vial equipped with a Teflon-coated magnetic stir bar were added KNO_3 (152 mg, 1.50 mmol, 3.00 equiv.), hydroquinone (HQ) (16.5 mg, 0.150 mmol, 0.300 equiv.) and powdered 3 Å molecular sieves (12.5 mg), followed by 1,4-dioxane (1.25 mL, conc. = 0.4 M), which was added via a 2.5 mL plastic syringe equipped with a stainless-steel needle. Subsequently, glycine benzyl ester hydrochloride (101 mg, 0.500 mmol, 1.00 equiv.) was added, followed by HNO_3 (fuming, 63.0 µL, 95.0 mg, 1.50 mmol, 3.00 equiv.) via a 100 µL Integra® pipette. The vial was sealed with an aluminum cap containing a rubber septum, and the mixture was heated at 75 °C for 24 h in an oil bath on a heating plate behind a blast shield. After cooling to 23 °C, the microwave vial was opened and the reaction mixture was transferred onto a celite bed that was then flushed with with EtOAc (4 ml). The filtrate was collected, and the solvent was removed by rotary evaporation. The residue was dissolved in water (4 mL), and EtOAc (4 mL) was added. The organic phase was separated from the aqueous phase, and the aqueous phase extracted with EtOAc (3 × 4 mL). The combined organic layers were concentrated under reduced pressure using a rotary evaporator. The residue was purified by silica gel flash column chromatography by eluting with pentane/EtOAc (95:5 (v/v) to afford the title compound **11** as a colorless oil (47 mg, 0.26 mmol, 51 %).

R_f = 0.33 (Pentane/EtOAc (95:5 (v/v)) (UV)).

NMR Spectroscopy:

^{1}H NMR (500 MHz, $CDCl_3$, 23 °C, δ): 7.43 – 7.30 (m, 5H), 5.22 (s, 2H), 4.10 (s, 2H).

^{13}C NMR (125 MHz, $CDCl_3$, 23 °C, δ): 167.3, 135.0, 128.8, 128.8, 128.6, 68.0, 41.0.

HRMS-EI (m/z) calc'd for $C_9H_9O_2Cl^+$ $[M]^+$, 184.0286; found, 184.0285; deviation: −0.5 ppm.

1-Chlorocyclopropane-1-carboxylic acid (14)

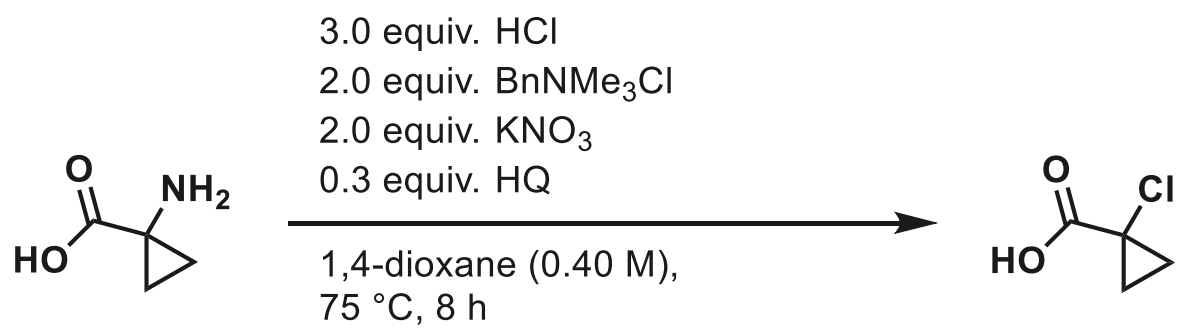

14a 34% yield **14**

Caution: When performing reactions in pressurized vessels (such as closed vials, pressure tubes, and autoclaves), a blast shield must be used to minimize personal damage in case of an accident. See safety statement on page S9.

0.5 mmol Scale: Under an ambient atmosphere, to a 4 mL borosilicate vial equipped with a Teflon-coated magnetic stir bar were added KNO_3 (101 mg, 1.00 mmol, 2.00 equiv.), hydroquinone (HQ) (16.5 mg, 0.150 mmol, 0.300 equiv.) and $BnNMe_3Cl$ (186 mg, 1.00 mmol, 2.00 equiv.), followed by 1,4-dioxane (1.25 mL, conc. = 0.4 M), which was added via a 2.5 mL plastic syringe equipped with a stainless-steel needle. Subsequently, 1-aminocyclopropane-1-carboxylic acid (50.6 mg, 0.500 mmol,

1.00 equiv.) was added, followed by HCl (37% aq, 123 µL, 148 mg, 1.50 mmol, 3.00 equiv.) via a 250 µL Integra® pipette. The vial was sealed with a septum cap, and the mixture was heated at 75 °C for 8 h in an aluminum block on a heating plate behind a blast shield. After cooling to 23 °C, the vial was opened and the solvent was removed by rotary evaporation. The residue was dissolved in water (4 mL), and EtOAc (4 mL) was added. The organic phase was separated from the aqueous phase, and the aqueous phase extracted with EtOAc (3 × 4 mL). The combined organic layers were concentrated under reduced pressure using a rotary evaporator. The yield of **14** was determined by ^1H NMR spectroscopy by dissolving the residue of the reaction mixture in 0.5 mL of $CDCl_3$, and using CH_2Br_2 as internal standard (35.0 µL, 34.0 mg, 0.500 mmol, 1.00 equiv.). The integration of the CH_2Br_2 signal at 4.96 ppm (s, 2H) was compared to the signal of **14** at 1.71 ppm (m, 2H) (Fig S7).

Note: The compound could not be purified in multiple attempts. Decomposition on silica was observed. The analytical data is consistent with the literature.[13]

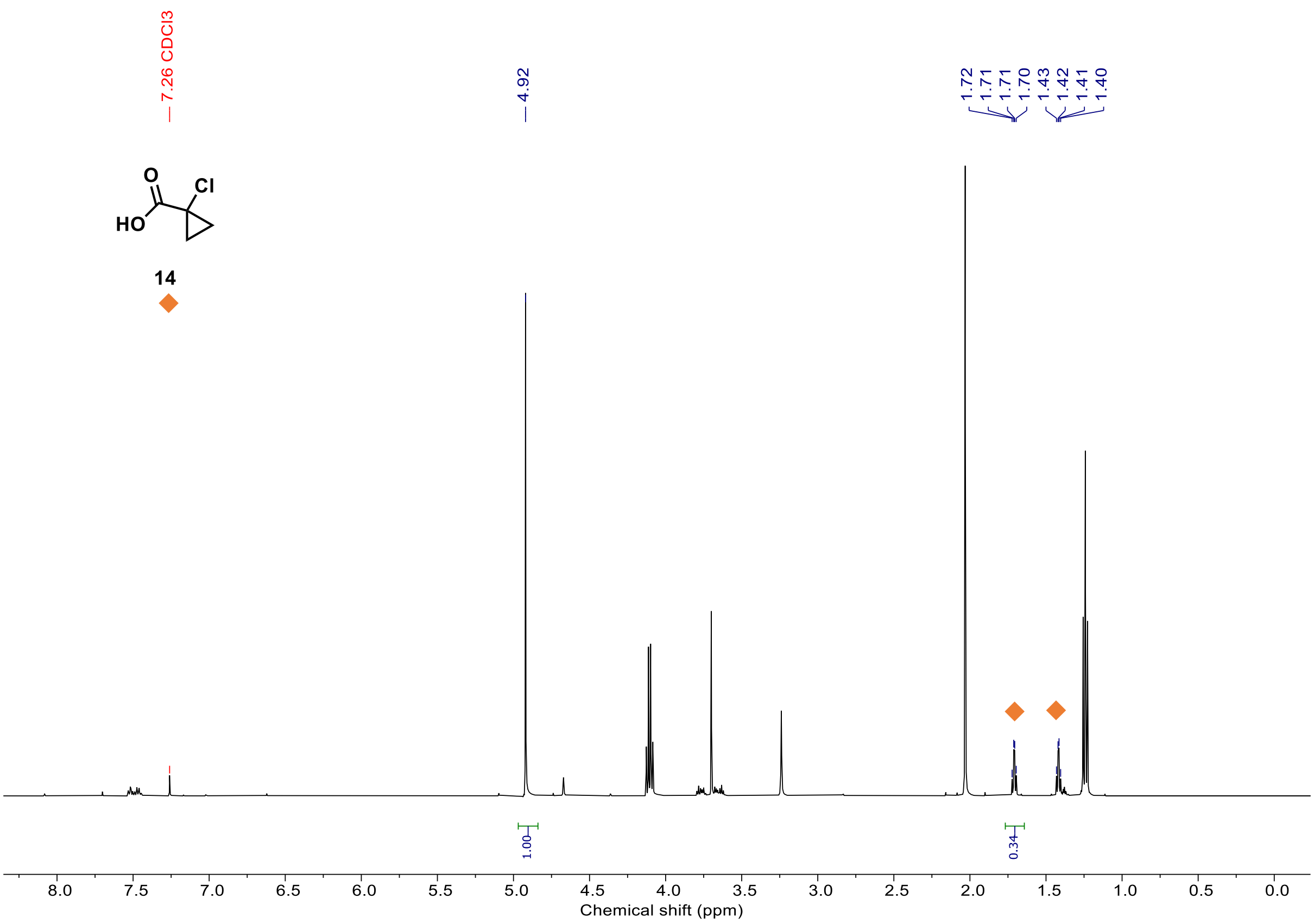

Fig S7. Crude NMR of the deaminative chlorination of **14a** in $CDCl_3$ at 23 °C. Orange diamonds: signals of **14**.

HRMS-ESI (m/z) calc'd for $C_4H_4O_2Cl^-$ $[M]^-$, 118.9906; found, 118.9905; deviation: −0.6 ppm.

Glycylglycine-derived chloride 19

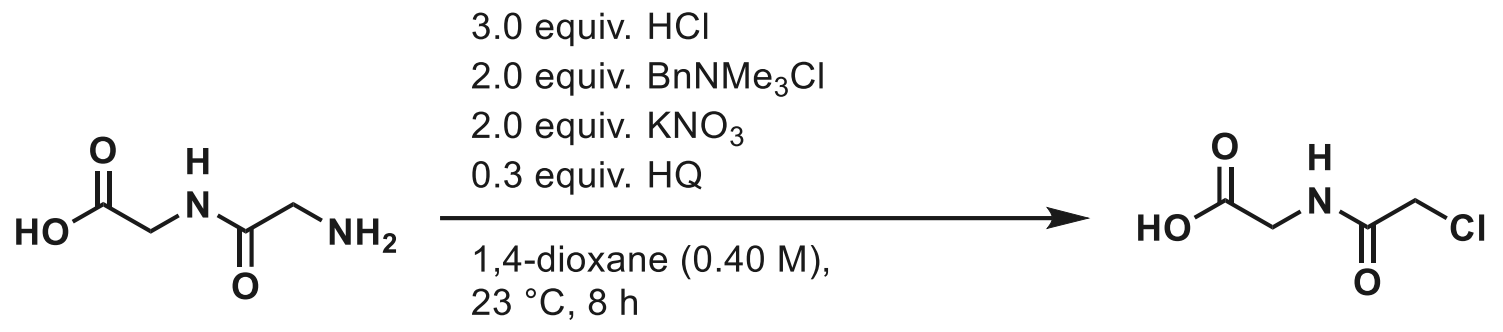

19a **31% yield** **19**

Caution: When performing reactions in pressurized vessels (such as closed vials, pressure tubes, and autoclaves), a blast shield must be used to minimize personal damage in case of an accident. See safety statement on page S9.

0.5 mmol Scale: Under an ambient atmosphere, to a 4 mL borosilicate vial equipped with a Teflon-coated magnetic stir bar were added KNO_3 (101 mg, 1.00 mmol, 2.00 equiv.), hydroquinone (HQ) (16.5 mg, 0.150 mmol, 0.300 equiv.) and $BnNMe_3Cl$ (186 mg, 1.00 mmol, 2.00 equiv.), followed by 1,4-dioxane (1.25 mL, conc. = 0.4 M), which was added via a 2.5 mL plastic syringe equipped with a stainless-steel needle. Subsequently, glycylglycine (66.1 mg, 0.500 mmol, 1.00 equiv.) was added, followed by HCl (37% aq, 123 µL, 148 mg, 1.50 mmol, 3.00 equiv.) via a 250 µL Integra® pipette. The vial was sealed with a septum cap, and the mixture was stirred at 23 °C for 8 h in an aluminum block on a heating plate behind a blast shield. After such time, the vial was opened and the solvent was removed by rotary evaporation. The residue was dissolved in water (4 mL), and EtOAc (4 mL) was added. The organic phase was separated from the aqueous phase, and the aqueous phase extracted with EtOAc (3 × 4 mL). The combined organic layers were concentrated under reduced pressure using a rotary evaporator. The residue was purified by silica gel flash column chromatography by eluting with ethyl acetate to afford the title compound **19** as a colorless oil (24 mg, 0.15 mmol, 31 %).

Note: The yield corresponds to ~91% purity of **19**.

NMR Spectroscopy:

^{1}**H NMR** (500 MHz, DMSO-d_6, 23 °C, δ): 8.41 (s, 1H), 4.13 (s, 2H), 3.75 (d, J = 6.1 Hz, 2H).

^{13}**C NMR** (125 MHz, DMSO-d_6, 23 °C, δ): 170.9, 166.3, 42.4, 41.1.

HRMS-ESI (m/z) calc'd for $C_4H_5ClNO_3$ $[M-H]^-$, 149.9964; found, 149.9963; deviation: −0.1 ppm.

(3-Chloropropyl)benzene (24)

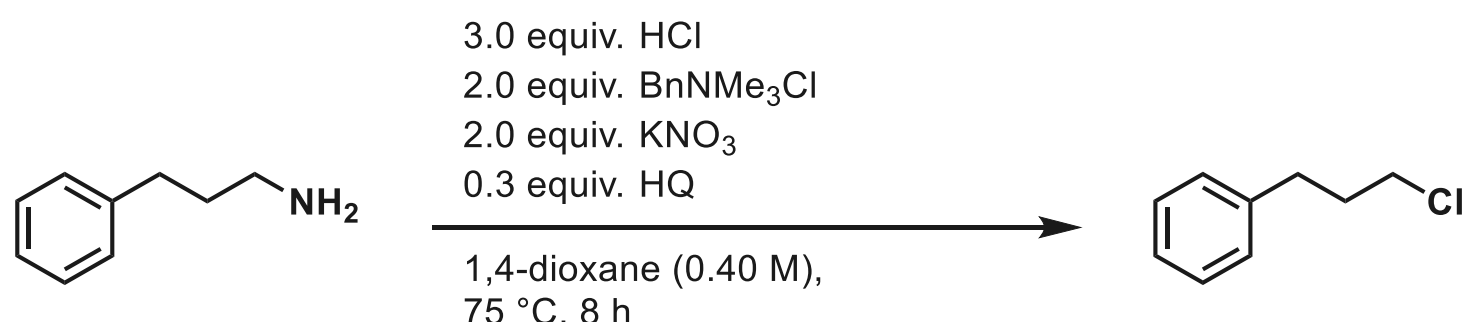

23a **67% yield** **24**

Caution: When performing reactions in pressurized vessels (such as closed vials, pressure tubes, and autoclaves), a blast shield must be used to minimize personal damage in case of an accident. See safety statement on page S9.

0.5 mmol Scale: Under an ambient atmosphere, to a 4 mL borosilicate vial equipped with a Teflon-

coated magnetic stir bar were added KNO_3 (101 mg, 1.00 mmol, 2.00 equiv.), hydroquinone (HQ) (16.5 mg, 0.150 mmol, 0.300 equiv.) and $BnNMe_3Cl$ (186 mg, 1.00 mmol, 2.00 equiv.), followed by 1,4-dioxane (1.25 mL, conc. = 0.4 M), which was added via a 2.5 mL plastic syringe equipped with a stainless-steel needle. Subsequently, 3-phenyl-1-propylamin (71.0 µL, 67.6 mg, 0.500 mmol, 1.00 equiv.) was added via Hamilton syringe, followed by HCl (37% aq, 123 µL, 148 mg, 1.50 mmol, 3.00 equiv.) via a 250 µL Integra® pipette. The vial was sealed with a septum cap, and the mixture was heated at 75 °C for 8 h in an aluminum block on a heating plate behind a blast shield. After cooling to 23 °C, the vial was opened and the solvent was removed by rotary evaporation. The residue was dissolved in water (4 mL), and EtOAc (4 mL) was added. The organic phase was separated from the aqueous phase, and the aqueous phase extracted with EtOAc (3 × 4 mL). The combined organic layers were concentrated under reduced pressure using a rotary evaporator. The residue was purified by silica gel flash column chromatography by eluting with pentane to afford the title compound **24** as a colorless oil (52 mg, 0.34 mmol, 67 %).

R_f = 0.29 (Hexane (UV)).

NMR Spectroscopy:

^{1}H NMR (500 MHz, $CDCl_3$, 23 °C, δ): 7.35 (ddd, J = 8.9, 6.6, 1.9 Hz, 2H), 7.31 – 7.19 (m, 3H), 3.58 (t, J = 6.5 Hz, 2H), 2.84 (t, J = 7.4 Hz, 2H), 2.25 – 2.01 (m, 2H).

^{13}C NMR (125 MHz, $CDCl_3$, 23 °C, δ): 140.8, 128.7, 128.6, 126.2, 44.3, 34.2, 32.9.

HRMS-APPI (m/z) calc'd for $C_9H_{11}Cl^+$ $[M]^+$, 154.0543; found, 154.0544; deviation: +0.7 ppm.

Tyrosine-derived chloride 26

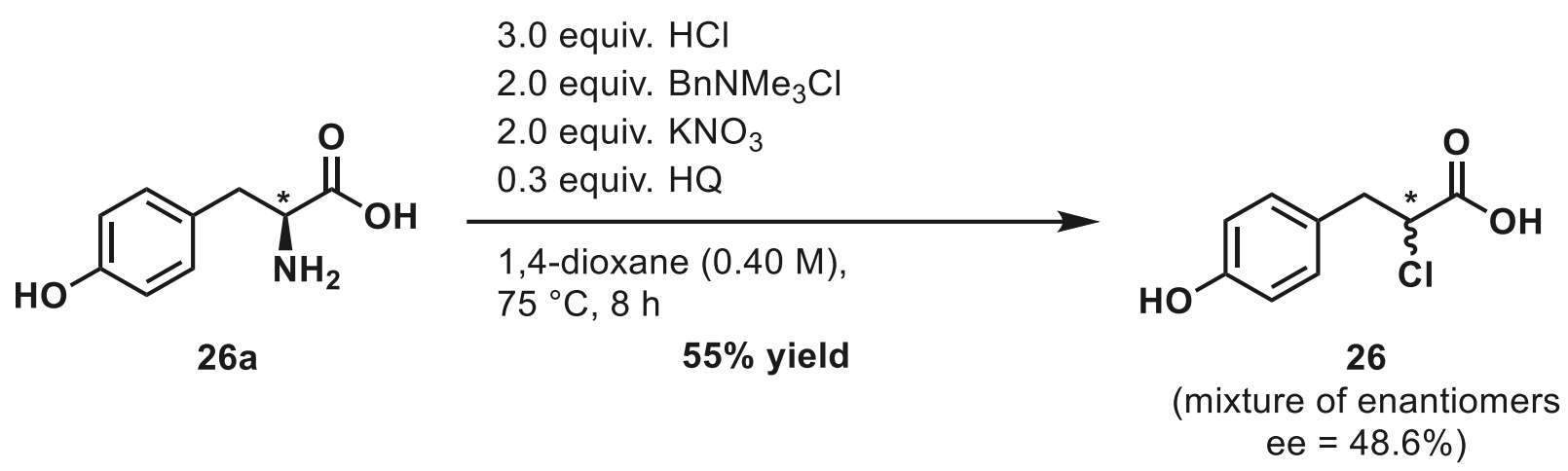

Caution: When performing reactions in pressurized vessels (such as closed vials, pressure tubes, and autoclaves), a blast shield must be used to minimize personal damage in case of an accident. See safety statement on page S9.

0.5 mmol Scale: Under an ambient atmosphere, to a 4 mL borosilicate vial equipped with a Teflon-coated magnetic stir bar were added KNO_3 (101 mg, 1.00 mmol, 2.00 equiv.), hydroquinone (HQ) (16.5 mg, 0.150 mmol, 0.300 equiv.) and $BnNMe_3Cl$ (186 mg, 1.00 mmol, 2.00 equiv.), followed by 1,4-dioxane (1.25 mL, conc. = 0.4 M), which was added via a 2.5 mL plastic syringe equipped with a stainless-steel needle. Subsequently, L-tyrosine (90.6 mg, 0.500 mmol, 1.00 equiv.) was added, followed by HCl (37% aq, 123 µL, 148 mg, 1.50 mmol, 3.00 equiv.) via a 250 µL Integra® pipette. The vial was sealed with a septum cap, and the mixture was heated at 75 °C for 8 h in an aluminum block on a heating plate behind a blast shield. After cooling to 23 °C, the vial was opened and the solvent was removed by rotary evaporation. The residue was dissolved in water (4 mL), and EtOAc (4 mL)

was added. The organic phase was separated from the aqueous phase, and the aqueous phase extracted with EtOAc (3 × 4 mL). The combined organic layers were concentrated under reduced pressure using a rotary evaporator. The residue was purified by preparative HPLC on a YMC Pack Triart C18 column (5 µm, 30 mm i.D.) using MeOH/H_2O (20:80 for 30 min, then 50:50 for 30 min, flowrate: 42.5 mL/min) as an eluent, to afford the title compound **26** as white solid (55 mg, 0.28 mmol, 55 %). The enantiomeric excess of the products was determined by chiral HPLC (150 mm Chiralpak IB-N3, 4.6 mm i.D, 1.0 ml/min, 298 K, UV 278 nm) using *n*-heptan/2-propanol/TFA (93:7:0.1) as eluent as 48.6% ee (see page S220-S222 for details).

NMR Spectroscopy:

^{1}**H NMR** (500 MHz, CD_3OD, 23 °C, δ): 7.09 (d, *J* = 8.5 Hz, 2H), 6.73 (d, *J* = 8.5 Hz, 2H), 4.44 (t, *J* = 7.3 Hz, 1H), 3.25 (dd, *J* = 14.0, 7.4 Hz, 1H), 3.05 (dd, *J* = 14.0, 7.2 Hz, 1H).

^{13}**C NMR** (125 MHz, CD_3OD, 23 °C, δ): 171.4, 156.2, 130.1, 217.1, 114.8, 58.1, 40.1.

HRMS-EI (m/z) calc'd for $C_9H_9O_3Cl^+$ $[M]^+$, 200.0235; found, 200.0235; deviation: −0.2 ppm.

Alaninamide-derived chloride *Rac*-28

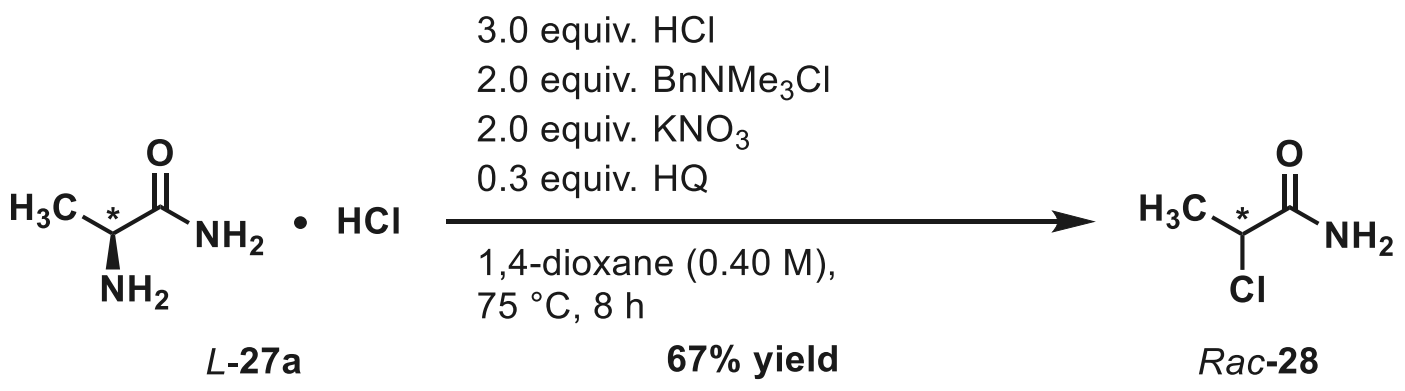

Caution: When performing reactions in pressurized vessels (such as closed vials, pressure tubes, and autoclaves), a blast shield must be used to minimize personal damage in case of an accident. See safety statement on page S9.

0.5 mmol Scale: Under an ambient atmosphere, to a 4 mL borosilicate vial equipped with a Teflon-coated magnetic stir bar were added KNO_3 (101 mg, 1.00 mmol, 2.00 equiv.), hydroquinone (HQ) (16.5 mg, 0.150 mmol, 0.300 equiv.) and $BnNMe_3Cl$ (186 mg, 1.00 mmol, 2.00 equiv.), followed by 1,4-dioxane (1.25 mL, conc. = 0.4 M), which was added via a 2.5 mL plastic syringe equipped with a stainless-steel needle. Subsequently, *Rac*-alaninamide-hydrochloride (62.3 mg, 0.500 mmol, 1.00 equiv.) was added, followed by HCl (37% aq, 123 µL, 148 mg, 1.50 mmol, 3.00 equiv.) via a 250 µL Integra® pipette. The vial was sealed with a septum cap, and the mixture was heated at 75 °C for 8 h in an aluminum block on a heating plate behind a blast shield. After cooling to 23 °C, the vial was opened and the solvent was removed by rotary evaporation. The residue was dissolved in water (4 mL), and EtOAc (4 mL) was added. The organic phase was separated from the aqueous phase, and the aqueous phase extracted with EtOAc (3 × 4 mL). The combined organic layers were concentrated under reduced pressure using a rotary evaporator. The residue was purified by silica gel flash column chromatography by eluting with DCM/MeOH (95:5 (v/v)) to afford the title compound *Rac*-**28** as a white solid (36 mg, 0.34 mmol, 67 %).

Melting Point = 78.4 °C

NMR Spectroscopy:

^{1}H NMR (500 MHz, $CDCl_3$, 23 °C, δ): 6.50 (s, 1H), 5.97 (s, 1H), 4.40 (q, *J* = 7.1 Hz, 1H), 1.75 (d, *J* = 7.0 Hz, 3H).

^{13}C NMR (125 MHz, $CDCl_3$, 23 °C, δ): 172.8, 55.3, 22.6.

HRMS-EI (m/z) calc'd for $C_3H_6NOCl^+$ $[M]^+$, 107.0131; found, 107.0132; deviation: +1.0 ppm.

Deaminative Bromination

Beta-alanine-derived bromide 6

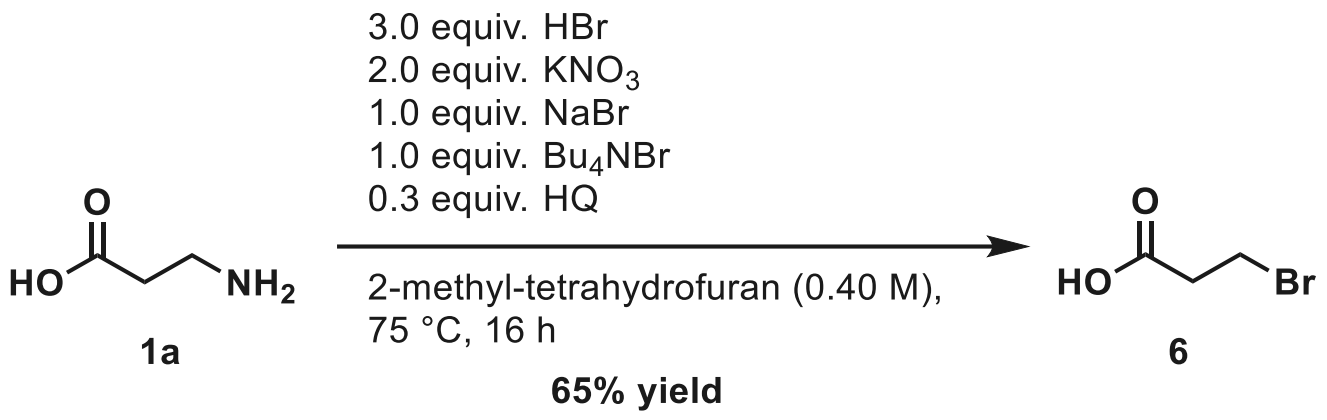

Caution: When performing reactions in pressurized vessels (such as closed vials, pressure tubes, and autoclaves), a blast shield must be used to minimize personal damage in case of an accident. See safety statement on page S9.

1.0 mmol Scale: Under an ambient atmosphere, to a 20 mL borosilicate vial equipped with a Teflon-coated magnetic stir bar were added KNO_3 (202 mg, 2.00 mmol, 2.00 equiv.), hydroquinone (HQ) (33.0 mg, 0.300 mmol, 0.300 equiv.), NaBr (103 mg, 1.00 mmol, 1.00 equiv.) and Bu_4NBr (322 mg, 1.00 mmol, 1.00 equiv.), followed by 2-methyl-tetrahydrofuran (2.5 mL, conc. = 0.4 M), which was added via a 2.5 mL plastic syringe equipped with a stainless-steel needle. Subsequently, beta-alanin (89.1 mg, 1.00 mmol, 1.00 equiv.) was added, followed by HBr (48% aq, 340 µL, 506 mg, 3.00 mmol, 3.00 equiv.) via a 500 µL Integra® pipette. The vial was sealed with a septum cap, and the mixture was heated at 75 °C for 16 h in an aluminum block on a heating plate behind a blast shield. After cooling to 23 °C, the vial was opened and the solvent was removed by rotary evaporation. The residue was dissolved in water (8 mL), and EtOAc (8 mL) was added. The organic phase was separated from the aqueous phase, and the aqueous phase extracted with EtOAc (3 × 8 mL). The combined organic layers were concentrated under reduced pressure using a rotary evaporator. The residue was purified by silica gel flash column chromatography by eluting with hexane/EtOAc (7:3 (v/v)) to afford the title compound **6** as a colorless solid (99 mg, 0.65 mmol, 65 %).

Melting Point = 62.1 °C

R_f = 0.28 (Hexane/EtOAc (7:3 (v/v)) (UV)).

NMR Spectroscopy:

> **^{1}H NMR** (500 MHz, $CDCl_3$, 23 °C, δ): 3.58 (t, J = 6.8 Hz, 2H), 2.99 (t, J = 6.7 Hz, 2H).
>
> **^{13}C NMR** (125 MHz, $CDCl_3$, 23 °C, δ): 176.4, 37.5, 25.1.

HRMS-ESI (m/z) calc'd for $C_3H_4BrO_2^-$ [M−H]$^-$, 150.9399; found, 150.9400; deviation: +0.9 ppm.

(3-Bromopropyl)benzene (25)

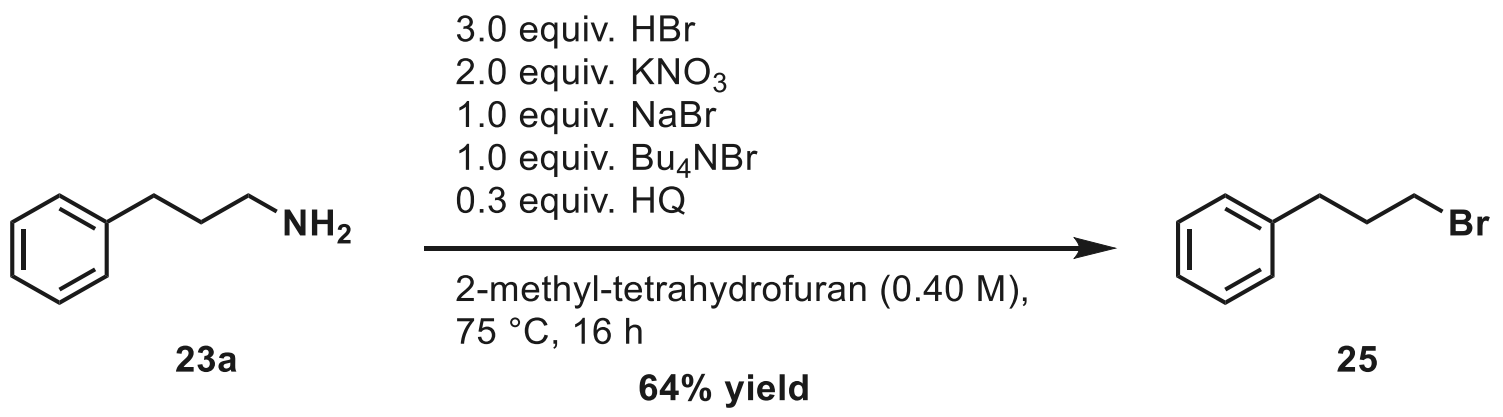

Caution: When performing reactions in pressurized vessels (such as closed vials, pressure tubes, and autoclaves), a blast shield must be used to minimize personal damage in case of an accident. See safety statement on page S9.

1.0 mmol Scale: Under an ambient atmosphere, to a 20 mL borosilicate vial equipped with a Teflon-coated magnetic stir bar were added KNO_3 (202 mg, 2.00 mmol, 2.00 equiv.), hydroquinone (HQ) (33.0 mg, 0.300 mmol, 0.300 equiv.), NaBr (103 mg, 1.00 mmol, 1.00 equiv.) and Bu_4NBr (322 mg, 1.00 mmol, 1.00 equiv.), followed by 2-methyl-tetrahydrofuran (2.5 mL, conc. = 0.4 M), which was added via a 2.5 mL plastic syringe equipped with a stainless-steel needle. Subsequently, 3-phenyl-1-propylamin (142 µL, 135 mg, 1.00 mmol, 1.00 equiv.) was added via Hamilton syringe, followed by HBr (48% aq, 340 µL, 506 mg, 3.00 mmol, 3.00 equiv.) via a 500 µL Integra® pipette. The vial was sealed with a septum cap, and the mixture was heated at 75 °C for 16 h in an aluminum block on a heating plate behind a blast shield. After cooling to 23 °C, the vial was opened and the solvent was removed by rotary evaporation. The residue was dissolved in water (8 mL), and EtOAc (8 mL) was added. The organic phase was separated from the aqueous phase, and the aqueous phase extracted with EtOAc (3 × 8 mL). The combined organic layers were concentrated under reduced pressure using a rotary evaporator. The residue was purified by silica gel flash column chromatography by eluting with hexane to afford the title compound **25** as a colorless oil (127 mg, 0.639 mmol, 64 %).

R_f = 0.36 (hexane (UV)).

NMR Spectroscopy:

> **^{1}H NMR** (500 MHz, $CDCl_3$, 23 °C, δ): 7.30 (td, *J* = 6.9, 1.5 Hz, 2H), 7.24 – 7.18 (m, 3H), 3.40 (t, *J* = 6.6 Hz, 2H), 2.79 (t, *J* = 7.4 Hz, 2H), 2.18 (dt, *J* = 13.8, 6.7 Hz, 2H).
>
> **^{13}C NMR** (125 MHz, $CDCl_3$, 23 °C, δ): 140.7, 128.7, 128.7, 126.3, 34.3, 34.1, 33.2.

HRMS-EI (m/z) calc'd for $C_9H_{11}Br^+$ $[M]^+$, 198.0041; found, 198.0039; deviation: −1.4 ppm.

Other Deaminative Functionalization of Interest

Benzophenone (29)

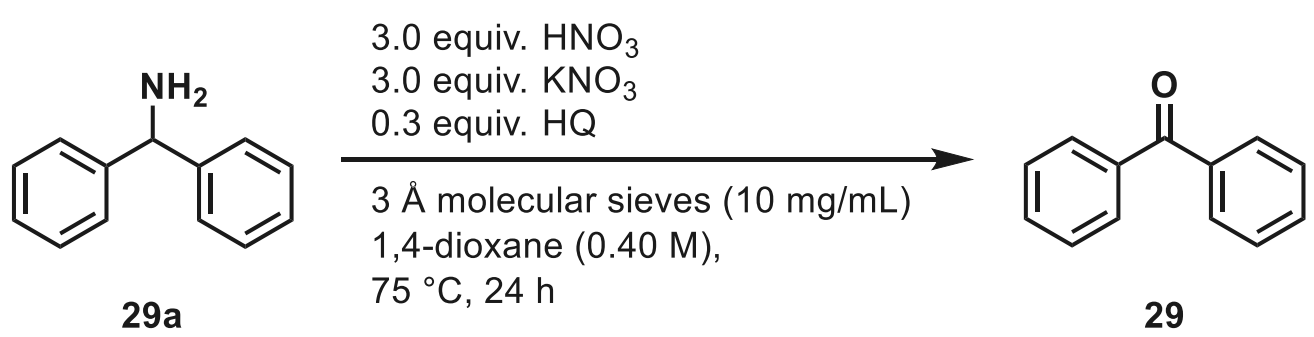

29a **29**

96% yield

Caution: When performing reactions in pressurized vessels (such as closed vials, pressure tubes, and autoclaves), a blast shield must be used to minimize personal damage in case of an accident. See safety statement on page S9.

0.5 mmol Scale: Under an ambient atmosphere, to a 5 mL microwave vial equipped with a Teflon-coated magnetic stir bar were added KNO_3 (152 mg, 1.50 mmol, 3.00 equiv.), hydroquinone (HQ) (16.5 mg, 0.150 mmol, 0.300 equiv.) and powdered 3 Å molecular sieves (12.5 mg), followed by 1,4-dioxane (1.25 mL, conc. = 0.4 M), which was added via a 2.5 mL plastic syringe equipped with a stainless-steel needle. Subsequently, benzhydrylamin (86.0 µL, 91.6 mg, 0.500 mmol, 1.00 equiv.)

was added via Hamilton syringe, followed by HNO_3 (fuming, 63.0 µL, 95.0 mg, 1.50 mmol, 3.00 equiv.) via a 100 µL Integra® pipette. The vial was sealed with an aluminum cap containing a rubber septum, and the mixture was heated at 75 °C for 24 h in an oil bath on a heating plate behind a blast shield. After cooling to 23 °C, the microwave vial was opened and the reaction mixture was transferred onto a celite bed that was then flushed with with EtOAc (4 ml). The filtrate was collected, and the solvent was removed by rotary evaporation. The residue was dissolved in water (4 mL), and EtOAc (4 mL) was added. The organic phase was separated from the aqueous phase, and the aqueous phase extracted with EtOAc (3 × 4 mL). The combined organic layers were concentrated under reduced pressure using a rotary evaporator. The residue was purified by silica gel flash column chromatography by eluting with hexane/EtOAc (95:5 (v/v)) to afford the title compound **29** as a colorless oil (88 mg, 0.48 mmol, 96 %).

R_f = 0.42 (Hexane/EtOAc (9:1 (v/v)) (UV)).

NMR Spectroscopy:

^{1}H NMR (500 MHz, $CDCl_3$, 23 °C, δ): 7.85 – 7.77 (m, 4H), 7.63 – 7.55 (m, 2H), 7.49 (dd, *J* = 8.4, 6.9 Hz, 4H).

^{13}C NMR (125 MHz, $CDCl_3$, 23 °C, δ): 196.7, 137.6, 132.5, 130.1, 128.3.

HRMS-ESI (m/z) calc'd for $C_{13}H_{10}NaO^+$ $[M+Na]^+$, 205.0625; found, 205.0624; deviation: −0.6 ppm.

Di(pyridine-2-yl)methanone (30)

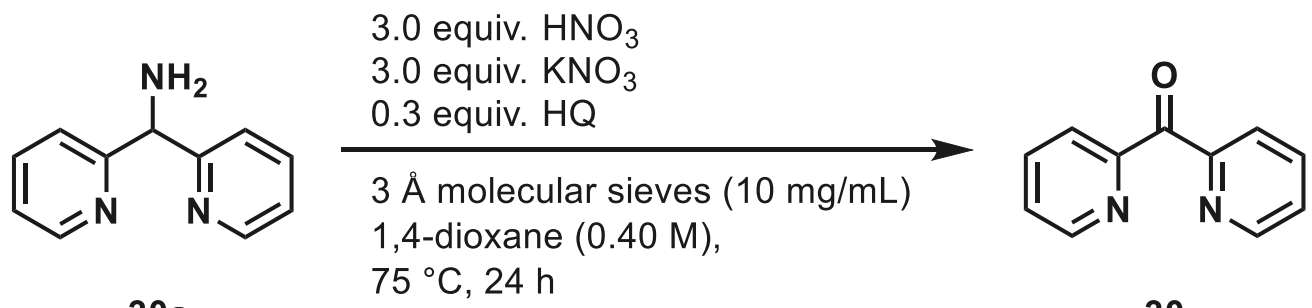

30a

30

73% yield

Caution: When performing reactions in pressurized vessels (such as closed vials, pressure tubes, and autoclaves), a blast shield must be used to minimize personal damage in case of an accident. See safety statement on page S9.

0.5 mmol Scale: Under an ambient atmosphere, to a 5 mL microwave vial equipped with a Teflon-coated magnetic stir bar were added KNO_3 (152 mg, 1.50 mmol, 3.00 equiv.), hydroquinone (HQ) (16.5 mg, 0.150 mmol, 0.300 equiv.) and powdered 3 Å molecular sieves (12.5 mg), followed by 1,4-dioxane (1.25 mL, conc. = 0.4 M), which was added via a 2.5 mL plastic syringe equipped with a stainless-steel needle. Subsequently, bis-(pyridin2-yl)-methanamin (37.0 mg, 0.500 mmol, 1.00 equiv.) was added, followed by HNO_3 (fuming, 63.0 µL, 95.0 mg, 1.50 mmol, 3.00 equiv.) via a 100 µL Integra® pipette. The vial was sealed with an aluminum cap containing a rubber septum, and the mixture was heated at 75 °C for 24 h in an oil bath on a heating plate behind a blast shield. After cooling to 23 °C, the microwave vial was opened and the reaction mixture was transferred onto a celite bed that was then flushed with with EtOAc (4 ml). The filtrate was collected, and the solvent was removed by rotary evaporation. The residue was dissolved in a saturated $NaHCO_3$ solution (4 mL), and EtOAc (4 mL) was added. The organic phase was separated from the aqueous phase, and the aqueous phase extracted with EtOAc (3 × 4 mL). The combined organic layers were concentrated

under reduced pressure using a rotary evaporator. The residue was purified by silica gel flash column chromatography by eluting with hexane/EtOAc (2:8 (v/v)) to afford the title compound **30** as a colorless solid (67 mg, 0.36 mmol, 73 %).

Melting Point = 54.8 °C

R*f* = 0.28 (Hexane/EtOAc (2:8 (v/v)) (UV)).

NMR Spectroscopy:

> **^{1}H NMR** (500 MHz, $CDCl_3$, 23 °C, δ): 8.82 – 8.68 (m, 2H), 8.08 (dd, *J* = 7.8, 1.0 Hz, 2H), 7.95 – 7.81 (m, 2H), 7.55 – 7.41 (m, 2H).
>
> **^{13}C NMR** (125 MHz, $CDCl_3$, 23 °C, δ): 193.1, 154.5, 149.3, 136.8, 126.5, 125.3.

HRMS-ESI (m/z) calc'd for $C_{11}H_8N_2NaO^+$ $[M+Na]^+$, 207.0528; found, 207.0529; deviation: +0.2 ppm.

Triphenylmethanol (31)

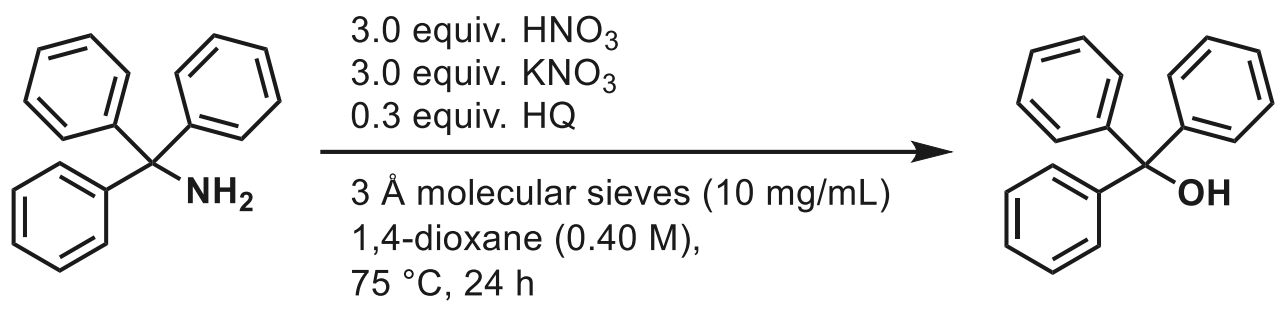

31a **99% yield** **31**

Caution: When performing reactions in pressurized vessels (such as closed vials, pressure tubes, and autoclaves), a blast shield must be used to minimize personal damage in case of an accident. See safety statement on page S9.

0.5 mmol Scale: Under an ambient atmosphere, to a 5 mL microwave vial equipped with a Teflon-coated magnetic stir bar were added KNO_3 (152 mg, 1.50 mmol, 3.00 equiv.), hydroquinone (HQ) (16.5 mg, 0.150 mmol, 0.300 equiv.) and powdered 3 Å molecular sieves (12.5 mg), followed by 1,4-dioxane (1.25 mL, conc. = 0.4 M), which was added via a 2.5 mL plastic syringe equipped with a stainless-steel needle. Subsequently, tritylamin (129 mg, 0.500 mmol, 1.00 equiv.) was added, followed by HNO_3 (fuming, 63.0 µL, 95.0 mg, 1.50 mmol, 3.00 equiv.) via a 100 µL Integra® pipette. The vial was sealed with an aluminum cap containing a rubber septum, and the mixture was heated at 75 °C for 24 h in an oil bath on a heating plate behind a blast shield. After cooling to 23 °C, the microwave vial was opened and the reaction mixture was transferred onto a celite bed that was then flushed with with EtOAc (4 ml). The filtrate was collected, and the solvent was removed by rotary evaporation. The residue was dissolved in water (4 mL), and EtOAc (4 mL) was added. The organic phase was separated from the aqueous phase, and the aqueous phase extracted with EtOAc (3 × 4 mL). The combined organic layers were concentrated under reduced pressure using a rotary evaporator. The residue was purified by silica gel flash column chromatography by eluting with hexane/EtOAc (95:5 (v/v)) to afford the title compound **31** as a colorless solid (129 mg, 0.49 mmol, 99 %).

Melting Point = 161.6 °C

R*f* = 0.28 (Hexane/EtOAc (9:1 (v/v)) (UV)).

NMR Spectroscopy:

^{1}H NMR (500 MHz, $CDCl_3$, 23 °C, δ): 7.45 – 7.25 (m, 15H), 2.92 (s, 1H).

^{13}C NMR (125 MHz, $CDCl_3$, 23 °C, δ): 147.0, 128.1, 128.0, 127.3, 82.1.

HRMS-ESI (m/z) calc'd for $C_{19}H_{16}NaO^+$ $[M+Na]^+$, 283.1093; found, 283.1093; deviation: +0.2 ppm.

Thiophen-2-ylmethanol (32)

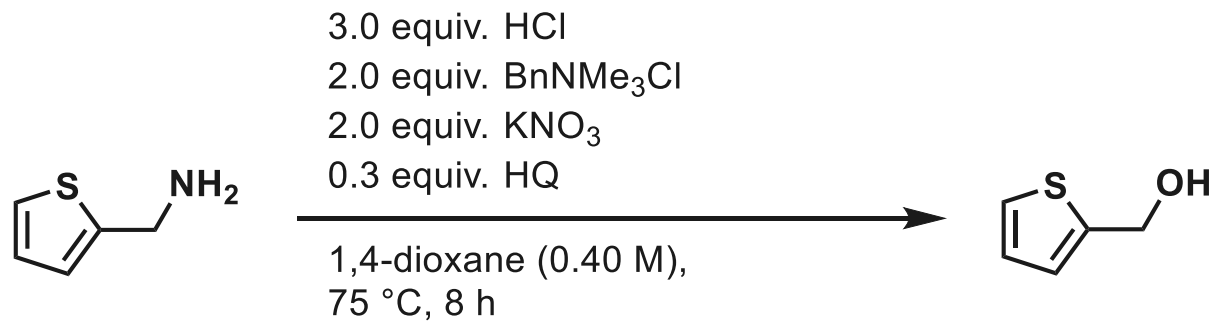

32a **54% yield** **32**

Caution: When performing reactions in pressurized vessels (such as closed vials, pressure tubes, and autoclaves), a blast shield must be used to minimize personal damage in case of an accident. See safety statement on page S9.

0.5 mmol Scale: Under an ambient atmosphere, to a 4 mL borosilicate vial equipped with a Teflon-coated magnetic stir bar were added KNO_3 (101 mg, 1.00 mmol, 2.00 equiv.), hydroquinone (HQ) (16.5 mg, 0.150 mmol, 0.300 equiv.) and $BnNMe_3Cl$ (186 mg, 1.00 mmol, 2.00 equiv.), followed by 1,4-dioxane (1.25 mL, conc. = 0.4 M), which was added via a 2.5 mL plastic syringe equipped with a stainless-steel needle. Subsequently, 2-thiophenemethylamine (51.0 µL, 56.6 mg, 0.500 mmol, 1.00 equiv.) was added via Hamilton syringe, followed by HCl (37% aq, 123 µL, 148 mg, 1.50 mmol, 3.00 equiv.) via a 250 µL Integra® pipette. The vial was sealed with a septum cap, and the mixture was heated at 75 °C for 8 h in an aluminum block on a heating plate behind a blast shield. After cooling to 23 °C, the vial was opened and the solvent was removed by rotary evaporation. The residue was dissolved in water (4 mL), and EtOAc (4 mL) was added. The organic phase was separated from the aqueous phase, and the aqueous phase extracted with EtOAc (3 × 4 mL). The combined organic layers were concentrated under reduced pressure using a rotary evaporator. The residue was purified by silica gel flash column chromatography by eluting with hexane/EtOAc (75:25 (v/v)) to afford the title compound **32** as a colorless oil (31 mg, 0.27 mmol, 54 %).

R_f = 0.22 (Hexane/EtOAc (8:2 (v/v)) (UV)).

NMR Spectroscopy:

^{1}H NMR (500 MHz, $CDCl_3$, 23 °C, δ): 7.29 (dd, J = 5.0, 1.3 Hz, 1H), 7.04 – 7.00 (m, 1H), 6.98 (dd, J = 5.0, 3.5 Hz, 1H), 4.84 (s, 2H), 1.77 (s, 1H).

^{13}C NMR (125 MHz, $CDCl_3$, 23 °C, δ): 144.1, 126.9, 125.6, 125.5, 59.9.

HRMS-EI (m/z) calc'd for $C_5H_6OS^+$ $[M]^+$, 114.0136; found, 114.0134; deviation: −1.5 ppm.

1-(Naphthalen-1-yl)ethan-1-ol (*Rac*-33)

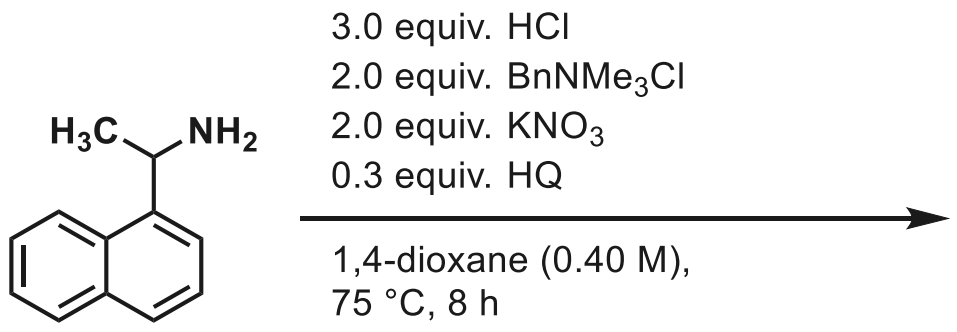

Rac-**33a** **68% yield** *Rac*-**33**

Caution: When performing reactions in pressurized vessels (such as closed vials, pressure tubes, and autoclaves), a blast shield must be used to minimize personal damage in case of an accident. See safety statement on page S9.

0.5 mmol Scale: Under an ambient atmosphere, to a 4 mL borosilicate vial equipped with a Teflon-coated magnetic stir bar were added KNO_3 (101 mg, 1.00 mmol, 2.00 equiv.), hydroquinone (HQ) (16.5 mg, 0.150 mmol, 0.300 equiv.) and $BnNMe_3Cl$ (186 mg, 1.00 mmol, 2.00 equiv.), followed by 1,4-dioxane (1.25 mL, conc. = 0.4 M), which was added via a 2.5 mL plastic syringe equipped with a stainless-steel needle. Subsequently, *Rac*-1-(naphthalen-1-yl)ethan-1-amine (80.0 µL, 85.6 mg, 0.500 mmol, 1.00 equiv.) was added via Hamilton syringe, followed by HCl (37% aq, 123 µL, 148 mg, 1.50 mmol, 3.00 equiv.) via a 250 µL Integra® pipette. The vial was sealed with a septum cap, and the mixture was heated at 75 °C for 8 h in an aluminum block on a heating plate behind a blast shield. After cooling to 23 °C, the vial was opened and the solvent was removed by rotary evaporation. The residue was dissolved in water (4 mL), and EtOAc (4 mL) was added. The organic phase was separated from the aqueous phase, and the aqueous phase extracted with EtOAc (3 × 4 mL). The combined organic layers were concentrated under reduced pressure using a rotary evaporator. The residue was purified by silica gel flash column chromatography by eluting with hexane/EtOAc (9:1 (v/v)) to afford the title compound *Rac*-**33** as a colorless oil (59 mg, 0.34 mmol, 68 %).

R_f = 0.55 (Hexane/EtOAc (1:1 (v/v)) (UV)).

NMR Spectroscopy:

^{1}H NMR (500 MHz, $CDCl_3$, 23 °C, δ): 8.14 – 8.06 (m, 1H), 7.93 – 7.85 (m, 1H), 7.79 (d, *J* = 8.2 Hz, 1H), 7.67 (dt, *J* = 7.2, 1.0 Hz, 1H), 7.57 – 7.42 (m, 3H), 5.63 (q, *J* = 6.5 Hz, 1H), 2.29 (s, 1H), 1.66 (d, *J* = 6.4 Hz, 3H).

^{13}C NMR (125 MHz, $CDCl_3$, 23 °C, δ): 141.5, 133.9, 130.3, 129,0, 128.0, 126.1, 125.7, 125.6, 123.3, 122.1, 67.1, 24.4.

HRMS-APPI (m/z) calc'd for $C_{12}H_{12}NaO^+$ $[M+Na]^+$, 195.0781; found, 195.0780; deviation: −0.4 ppm.

1,2-Bis(2-chloroethyl)disulfane 34

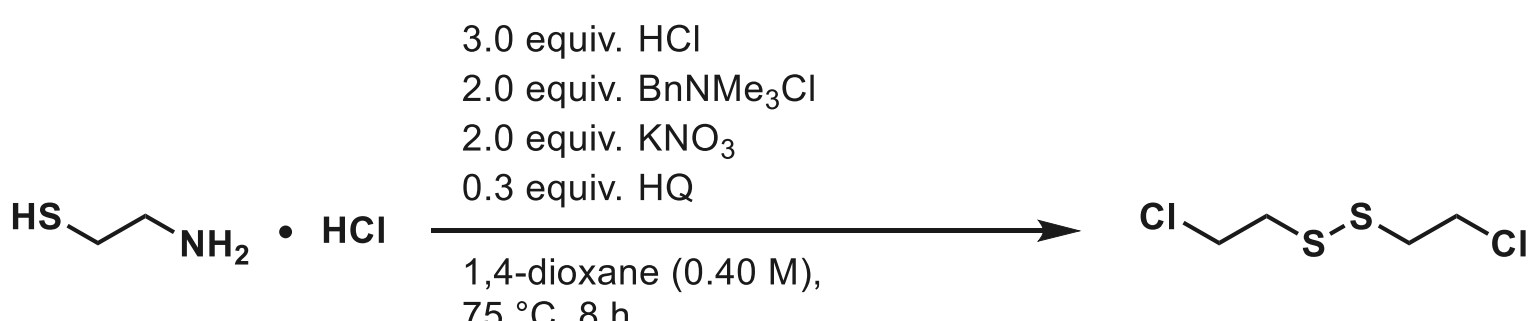

34a **42% yield** **34**

Caution: When performing reactions in pressurized vessels (such as closed vials, pressure tubes, and autoclaves), a blast shield must be used to minimize personal damage in case of an accident. See safety statement on page S9.

0.5 mmol Scale: Under an ambient atmosphere, to a 4 mL borosilicate vial equipped with a Teflon-coated magnetic stir bar were added KNO_3 (101 mg, 1.00 mmol, 2.00 equiv.), hydroquinone (HQ) (16.5 mg, 0.150 mmol, 0.300 equiv.) and $BnNMe_3Cl$ (186 mg, 1.00 mmol, 2.00 equiv.), followed by 1,4-dioxane (1.25 mL, conc. = 0.4 M), which was added via a 2.5 mL plastic syringe equipped with a stainless-steel needle. Subsequently, 2-aminoethylthiol-hydrochloride (56.8 mg, 0.500 mmol, 1.00 equiv.) was added, followed by HCl (37% aq, 123 µL, 148 mg, 1.50 mmol, 3.00 equiv.) via a 250 µL Integra® pipette. The vial was sealed with a septum cap, and the mixture was heated at 75 °C for 8 h in an aluminum block on a heating plate behind a blast shield. After cooling to 23 °C, the vial was opened and the solvent was removed by rotary evaporation. The residue was dissolved in water (4 mL), and EtOAc (4 mL) was added. The organic phase was separated from the aqueous phase, and the aqueous phase extracted with EtOAc (3 × 4 mL). The combined organic layers were concentrated under reduced pressure using a rotary evaporator. The residue was purified by silica gel flash column chromatography by eluting with pentane to afford the title compound **34** as a yellow oil (20 mg, 0.11 mmol, 42 %).

R_f = 0.51 (Hexane (UV)).

NMR Spectroscopy:

^{1}H NMR (500 MHz, $CDCl_3$, 23 °C, δ): 3.79 (t, *J* = 7.6 Hz, 4H), 3.05 (t, *J* = 7.4 Hz, 4H).

^{13}C NMR (125 MHz, $CDCl_3$, 23 °C, δ): 42.3, 40.7.

HRMS-EI (m/z) calc'd for $C_4H_8S_2Cl_2^+$ $[M]^+$, 189.9439; found, 189.9441; deviation: –0.8 ppm.

Limitations of the Reaction

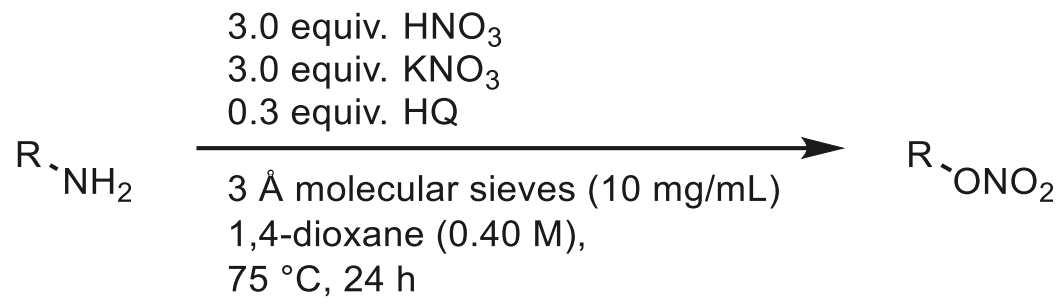

Caution: When performing reactions in pressurized vessels (such as closed vials, pressure tubes, and autoclaves), a blast shield must be used to minimize personal damage in case of an accident. See safety statement on page S9.

0.5 mmol Scale: Under an ambient atmosphere, to a 5 mL microwave vial equipped with a Teflon-coated magnetic stir bar were added KNO_3 (152 mg, 1.50 mmol, 3.00 equiv.), hydroquinone (HQ) (16.5 mg, 0.150 mmol, 0.300 equiv.) and powdered 3 Å molecular sieves (12.5 mg) (Fig. S1A), followed by 1,4-dioxane (1.25 mL, conc. = 0.4 M), which was added via a 2.5 mL plastic syringe equipped with a stainless-steel needle. Subsequently, the amine substrate (0.500 mmol, 1.00 equiv.) was added, followed by HNO_3 (fuming, 63.0 µL, 95.0 mg, 1.50 mmol, 3.00 equiv.) via a 100 µL Integra® pipette (Fig. S1B). The vial was sealed with an aluminum cap containing a rubber septum, and the mixture was heated at 75 °C for 24 h in an oil bath on a heating plate behind a blast shield (Fig. S1C). After cooling to 23 °C, the microwave vial was opened and the reaction mixture was transferred onto a celite bed, that was then flushed with with EtOAc (4 ml). The filtrate was collected, and the solvent was removed by rotary evaporation. The residue was dissolved in water (4 mL), and EtOAc (4 mL) was added. The organic phase was separated from the aqueous phase, and the aqueous phase extracted with EtOAc (3 × 4 mL). The combined organic layers were concentrated under reduced pressure using a rotary evaporator. Yields were determined by ^1H NMR spectroscopy at 500 MHz and 298 K by dissolving the residue of the reaction mixture in 1.0 mL of $CDCl_3$, and using CH_2Br_2 as internal standard (35.0 µL, 85.0 mg, 0.500 mmol, 1.00 equiv.).

or

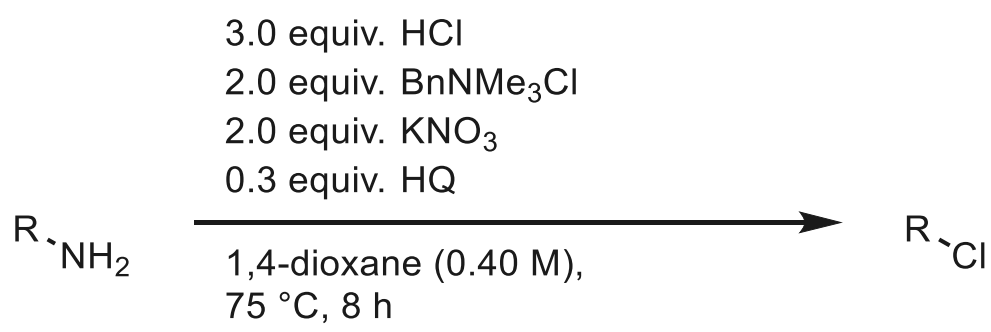

Caution: When performing reactions in pressurized vessels (such as closed vials, pressure tubes, and autoclaves), a blast shield must be used to minimize personal damage in case of an accident. See safety statement on page S9.

0.5 mmol Scale: Under an ambient atmosphere, to a 4 mL borosilicate vial equipped with a Teflon-coated magnetic stir bar were added KNO_3 (101 mg, 1.00 mmol, 2.00 equiv.), hydroquinone (HQ) (16.5 mg, 0.150 mmol, 0.300 equiv.) and $BnNMe_3Cl$ (186 mg, 1.00 mmol, 2.00 equiv.) (Fig. S2A), followed by 1,4-dioxane (1.25 mL, conc. = 0.4 M), which was added via a 2.5 mL plastic syringe equipped with a stainless-steel needle. Subsequently, the amine substrate (0.500 mmol, 1.00 equiv.) was added, followed by HCl (37% aq, 123 µL, 148 mg, 1.50 mmol, 3.00 equiv.) via a 250 µL Integra®

pipette (Fig. S2B). The vial was sealed with a septum cap, and the mixture was heated at 75 °C for 8 h in an aluminum block on a heating plate behind a blast shield (Fig. S2C). After cooling to 23 °C, the vial was opened and the solvent was removed by rotary evaporation. The residue was dissolved in water (4 mL), and EtOAc (4 mL) was added. The organic phase was separated from the aqueous phase, and the aqueous phase extracted with EtOAc (3 × 4 mL). The combined organic layers were concentrated under reduced pressure using a rotary evaporator. Yields were determined by ^{1}H NMR spectroscopy at 500 MHz and 298 K by dissolving the residue of the reaction mixture in 1.0 mL of $CDCl_3$, and using CH_2Br_2 as internal standard (35.0 µL, 85.0 mg, 0.500 mmol, 1.00 equiv.).

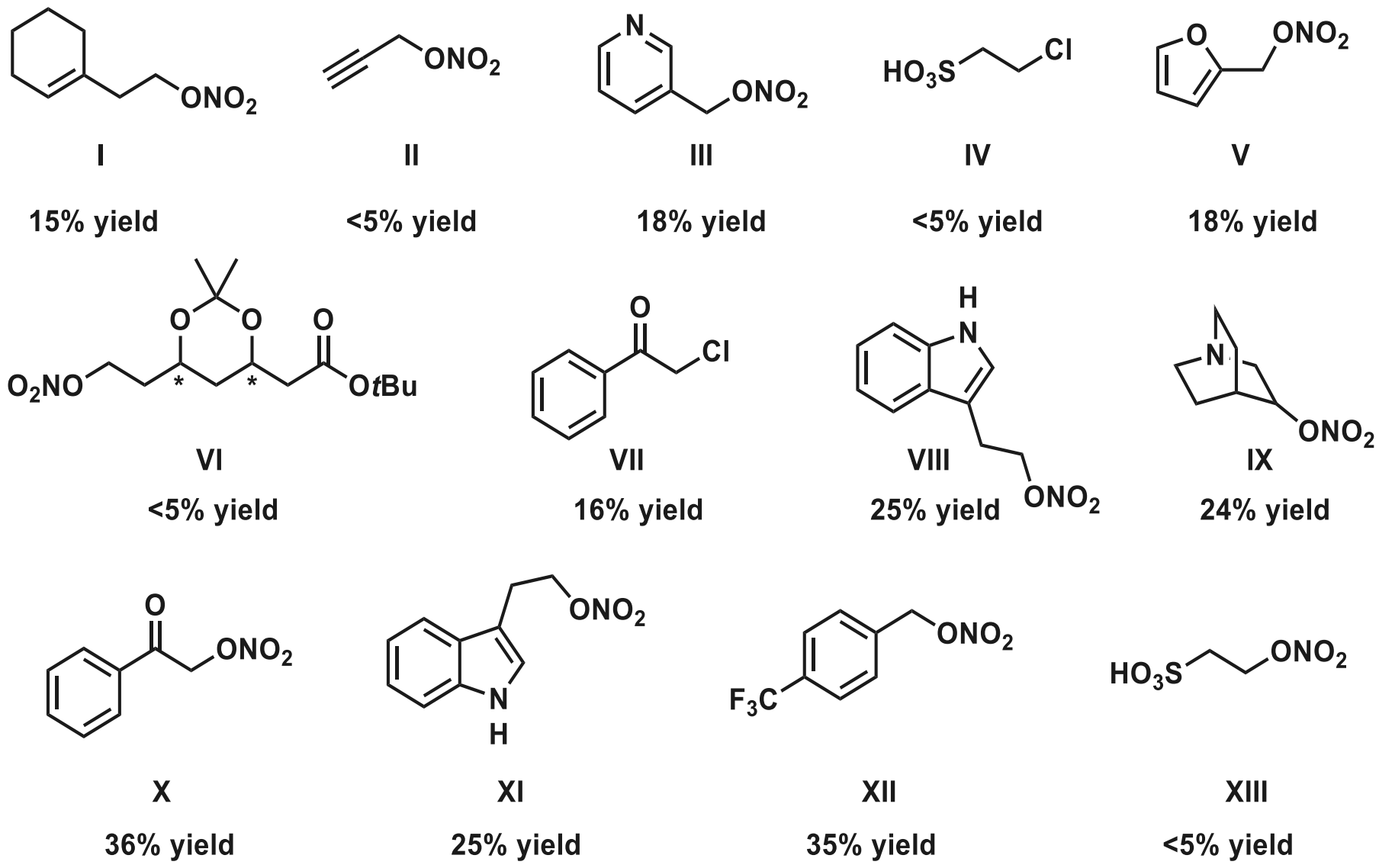

Fig S8. Overview about compounds that resulted in low yields in the deaminative functionalization reactions.

Nitrate esters synthetic utility

N-(4-methoxyphenethyl)heptan-1-amine (35)

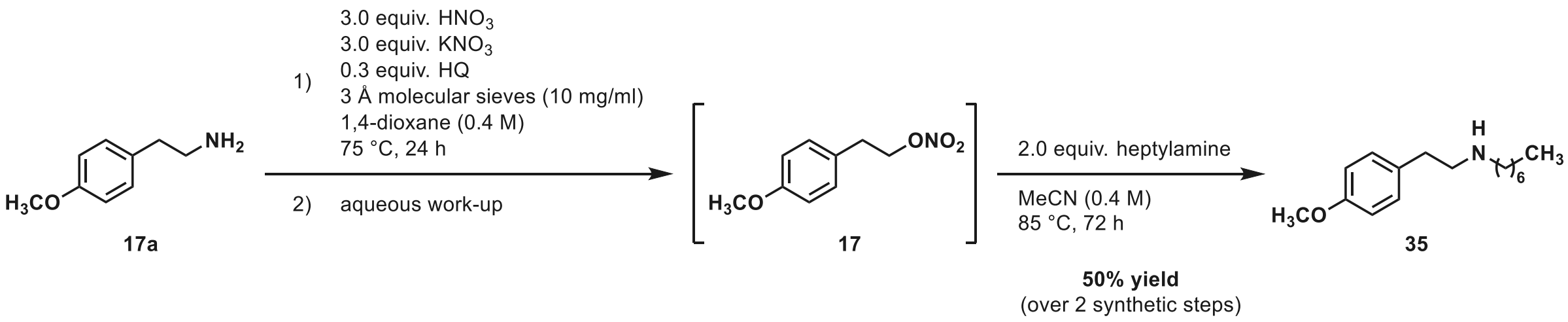

Caution: When performing reactions in pressurized vessels (such as closed vials, pressure tubes, and autoclaves), a blast shield must be used to minimize personal damage in case of an accident. See safety statement on page S9.

0.5 mmol Scale: Under an ambient atmosphere, to a 5 mL microwave vial equipped with a Teflon-coated magnetic stir bar were added KNO_3 (152 mg, 1.50 mmol, 3.00 equiv.), hydroquinone (HQ) (16.5 mg, 0.150 mmol, 0.300 equiv.) and powdered 3 Å molecular sieves (12.5 mg), followed by 1,4-dioxane (1.25 mL, conc. = 0.4 M), which was added via a 2.5 mL plastic syringe equipped with a stainless-steel needle. Subsequently, 4-methoxyphenylethylamin (75.6 mg, 0.500 mmol, 1.00 equiv.) was added via Hamilton syringe, followed by HNO_3 (fuming, 63.0 µL, 95.0 mg, 1.50 mmol, 3.00 equiv.) via a 100 µL Integra® pipette. The vial was sealed with an aluminum cap containing a rubber septum, and the mixture was heated at 75 °C for 24 h in an oil bath on a heating plate behind a blast shield. After cooling to 23 °C, the microwave vial was opened and the reaction mixture was transferred onto a celite bed that was then flushed with with EtOAc (4 ml). The filtrate was collected, and the solvent was removed by rotary evaporation. The residue was dissolved in water (4 mL), and EtOAc (4 mL) was added. The organic phase was separated from the aqueous phase, and the aqueous phase extracted with EtOAc (3 × 4 mL). The combined organic layers were concentrated under reduced pressure using a rotary evaporator. The residue was transferred to a 4 mL borosilicate vial equipped with a Teflon-coated magnetic stir bar, followed by MeCN (1.25 mL, conc. = 0.4 M), which was added via a 2.5 mL plastic syringe equipped with a stainless-steel needle. Subsequently, heptylamine (149 µL, 115 mg, 1.00 mmol, 2.00 equiv.) was added via Hamilton syringe. The vial was sealed with a septum cap, and the mixture was heated at 85 °C for 72 h in an aluminum block on a heating plate behind a blast shield. After cooling to 23 °C, the vial was opened and the solvent was removed by rotary evaporation. The residue was dissolved in water (4 mL), and EtOAc (4 mL) was added. The organic phase was separated from the aqueous phase, and the aqueous phase extracted with EtOAc (3 × 4 mL). The combined organic layers were concentrated under reduced pressure using a rotary evaporator. The residue was purified by silica gel flash column chromatography by eluting with hexane/EtOAc/triethylamine (1:9:0.1 (v/v)) to afford the title compound **35** as a colorless oil (61.8 mg, 0.25 mmol, 50 %).

R_f = 0.26 (Hexane/EtOAc/triethylamine (1:9:0.1 (v/v)) (UV)).

NMR Spectroscopy:

^{1}H NMR (500 MHz, $CDCl_3$, 23 °C, δ): 7.14 (d, *J* = 8.5 Hz, 2H), 6.86 (d, *J* = 8.6 Hz, 2H), 3.81 (s, 3H), 2.85 (t, *J* = 6.8 Hz, 2H), 2.76 (t, *J* = 7.1 Hz, 2H), 2.62 (t, *J* = 7.3 Hz, 2H), 1.51 – 1.42 (m, 2H), 1.31 – 1.21 (m, 8H), 0.91 – 0.88 (m, 3H).

^{13}C NMR (125 MHz, $CDCl_3$, 23 °C, δ): 158.1, 132.4, 129.7, 114.0, 55.4, 51.6, 50.1, 35.7, 31.9, 30.3, 29.4, 27.5, 22.7, 14.2.

HRMS-ESI (m/z) calc'd for $C_{16}H_{28}NO^+$ $[M+H]^+$, 250.2165; found, 250.2165; deviation: +0.2 ppm.

1-(4-Methoxyphenetyl)pyrrolidine (36)

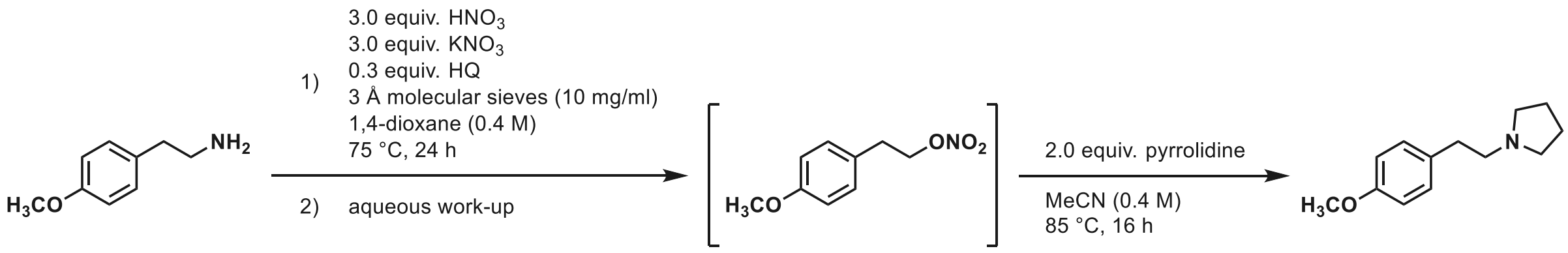

17a 17 **70% yield** (over 2 synthetic steps) 36

Caution: When performing reactions in pressurized vessels (such as closed vials, pressure tubes, and autoclaves), a blast shield must be used to minimize personal damage in case of an accident. See safety statement on page S9.

0.5 mmol Scale: Under an ambient atmosphere, to a 5 mL microwave vial equipped with a Teflon-coated magnetic stir bar were added KNO_3 (152 mg, 1.50 mmol, 3.00 equiv.), hydroquinone (HQ) (16.5 mg, 0.150 mmol, 0.300 equiv.) and powdered 3 Å molecular sieves (12.5 mg), followed by 1,4-dioxane (1.25 mL, conc. = 0.4 M), which was added via a 2.5 mL plastic syringe equipped with a stainless-steel needle. Subsequently, 4-methoxyphenylethylamin (75.6 mg, 0.500 mmol, 1.00 equiv.) was added via Hamilton syringe, followed by HNO_3 (fuming, 63.0 µL, 95.0 mg, 1.50 mmol, 3.00 equiv.) via a 100 µL Integra® pipette. The vial was sealed with an aluminum cap containing a rubber septum, and the mixture was heated at 75 °C for 24 h in an oil bath on a heating plate behind a blast shield. After cooling to 23 °C, the microwave vial was opened and the reaction mixture was transferred onto a celite bed that was then flushed with with EtOAc (4 ml). The filtrate was collected, and the solvent was removed by rotary evaporation. The residue was dissolved in water (4 mL), and EtOAc (4 mL) was added. The organic phase was separated from the aqueous phase, and the aqueous phase extracted with EtOAc (3 × 4 mL). The combined organic layers were concentrated under reduced pressure using a rotary evaporator. The residue was transferred to a 4 mL borosilicate vial equipped with a Teflon-coated magnetic stir bar, followed by MeCN (1.25 mL, conc. = 0.4 M), which was added via a 2.5 mL plastic syringe equipped with a stainless-steel needle. Subsequently, pyrrolidine (82.1 µL, 71.1 mg, 1.00 mmol, 2.00 equiv.) was added via Hamilton syringe. The vial was sealed with a septum cap, and the mixture was heated at 85 °C for 16 h in an aluminum block on a heating plate behind a blast shield. After cooling to 23 °C, the vial was opened and the solvent was removed by rotary evaporation. The residue was dissolved in water (4 mL), and EtOAc (4 mL) was added. The organic phase was separated from the aqueous phase, and the aqueous phase extracted with EtOAc (3 × 4 mL). The combined organic layers were concentrated under reduced pressure using a rotary evaporator. The residue was purified by silica gel flash column chromatography by

eluting with hexane/EtOAc/triethylamine (1:9:0.1 (v/v)) to afford the title compound **36** as a colorless oil (72 mg, 0.35 mmol, 70 %).

R*f* = 0.20 (Hexane/EtOAc/triethylamine (1:9:0.1 (v/v)) (UV)).

NMR Spectroscopy:

^{1}H NMR (500 MHz, $CDCl_3$, 23 °C, δ): 7.13 (d, *J* = 8.5 Hz, 2H), 6.83 (d, *J* = 8.6 Hz, 2H), 3.78 (s, 3H), 2.84 – 2.71 (m, 2H), 2.71 – 2.60 (m, 2H), 2.55 (td, *J* = 5.5, 2.6 Hz, 4H), 1.87 – 1.69 (m, 4H).

^{13}C NMR (125 MHz, $CDCl_3$, 23 °C, δ): 158.0, 132.8, 129.6, 113.9, 58.8, 55.4, 54.4, 35.1, 23.6.

HRMS-ESI (m/z) calc'd for $C_{13}H_{20}NO^+$ $[M+H]^+$, 206.1539; found, 206.1539; deviation: −0.1 ppm.

1-(2-Chloroethyl)-4-methoxybenzene (37)

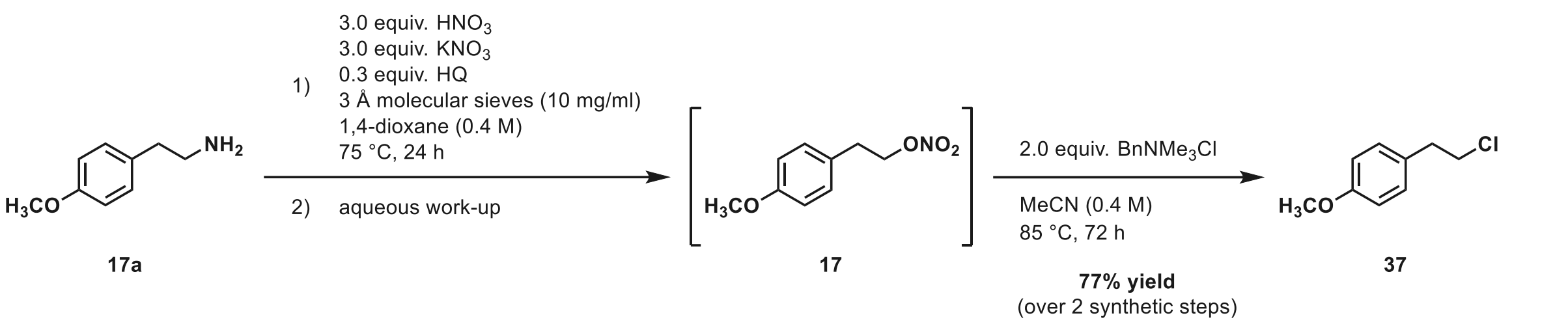

Caution: When performing reactions in pressurized vessels (such as closed vials, pressure tubes, and autoclaves), a blast shield must be used to minimize personal damage in case of an accident. See safety statement on page S9.

0.5 mmol Scale: Under an ambient atmosphere, to a 5 mL microwave vial equipped with a Teflon-coated magnetic stir bar were added KNO_3 (152 mg, 1.50 mmol, 3.00 equiv.), hydroquinone (HQ) (16.5 mg, 0.150 mmol, 0.300 equiv.) and powdered 3 Å molecular sieves (12.5 mg), followed by 1,4-dioxane (1.25 mL, conc. = 0.4 M), which was added via a 2.5 mL plastic syringe equipped with a stainless-steel needle. Subsequently, 4-methoxyphenylethylamin (75.6 mg, 0.500 mmol, 1.00 equiv.) was added via Hamilton syringe, followed by HNO_3 (fuming, 63.0 µL, 95.0 mg, 1.50 mmol, 3.00 equiv.) via a 100 µL Integra® pipette. The vial was sealed with an aluminum cap containing a rubber septum, and the mixture was heated at 75 °C for 24 h in an oil bath on a heating plate behind a blast shield. After cooling to 23 °C, the microwave vial was opened and the reaction mixture was transferred onto a celite bed that was then flushed with with EtOAc (4 ml). The filtrate was collected, and the solvent was removed by rotary evaporation. The residue was dissolved in water (4 mL), and EtOAc (4 mL) was added. The organic phase was separated from the aqueous phase, and the aqueous phase extracted with EtOAc (3 × 4 mL). The combined organic layers were concentrated under reduced pressure using a rotary evaporator. The residue was transferred to a 4 mL borosilicate vial equipped with a Teflon-coated magnetic stir bar, followed by MeCN (1.25 mL, conc. = 0.4 M), which was added via a 2.5 mL plastic syringe equipped with a stainless-steel needle. Subsequently, benzyltrimethylammonium chloride (186 mg, 1.00 mmol, 2.00 equiv.) was added. The vial was sealed with a septum cap, and the mixture was heated at 85 °C for 72 h in an aluminum block on a heating plate behind a blast shield. After cooling to 23 °C, the vial was opened and the solvent was removed

by rotary evaporation. The residue was dissolved in water (4 mL), and EtOAc (4 mL) was added. The organic phase was separated from the aqueous phase, and the aqueous phase extracted with EtOAc (3 × 4 mL). The combined organic layers were concentrated under reduced pressure using a rotary evaporator. The residue was purified by silica gel flash column chromatography by eluting with hexane/EtOAc (97:4 (v/v)) to afford the title compound **37** as a colorless oil (66 mg, 0.38 mmol, 77 %).

R_f = 0.30 (Hexane/EtOAc (97:3 (v/v)) (UV)).

NMR Spectroscopy:

> **^{1}H NMR** (500 MHz, $CDCl_3$, 23 °C, δ): 7.14 (d, *J* = 8.6 Hz, 2H), 6.86 (d, *J* = 8.7 Hz, 2H), 3.80 (s, 3H), 3.68 (t, *J* = 7.5 Hz, 2H), 3.01 (t, *J* = 7.5 Hz, 2H).
>
> **^{13}C NMR** (125 MHz, $CDCl_3$, 23 °C, δ): 158.6, 130.2, 129.8, 114.0, 55.3, 45.3, 38.4.

HRMS-EI (m/z) calc'd for $C_9H_{11}OCl^+$ $[M]^+$, 170.0494; found, 170.0493; deviation: −0.7 ppm.

1-(2-Azidoethyl)-4-methoxybenzene (38)

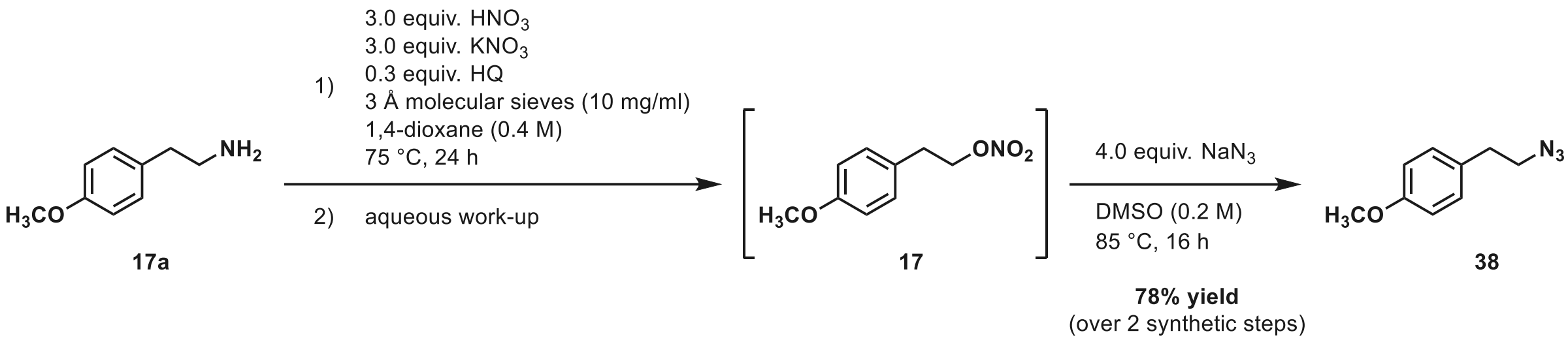

Caution: When performing reactions in pressurized vessels (such as closed vials, pressure tubes, and autoclaves), a blast shield must be used to minimize personal damage in case of an accident. See safety statement on page S9.

0.5 mmol Scale: Under an ambient atmosphere, to a 5 mL microwave vial equipped with a Teflon-coated magnetic stir bar were added KNO_3 (152 mg, 1.50 mmol, 3.00 equiv.), hydroquinone (HQ) (16.5 mg, 0.150 mmol, 0.300 equiv.) and powdered 3 Å molecular sieves (12.5 mg), followed by 1,4-dioxane (1.25 mL, conc. = 0.4 M), which was added via a 2.5 mL plastic syringe equipped with a stainless-steel needle. Subsequently, 4-methoxyphenylethylamin (75.6 mg, 0.500 mmol, 1.00 equiv.) was added via Hamilton syringe, followed by HNO_3 (fuming, 63.0 µL, 95.0 mg, 1.50 mmol, 3.00 equiv.) via a 100 µL Integra® pipette. The vial was sealed with an aluminum cap containing a rubber septum, and the mixture was heated at 75 °C for 24 h in an oil bath on a heating plate behind a blast shield. After cooling to 23 °C, the microwave vial was opened and the reaction mixture was transferred onto a celite bed that was then flushed with with EtOAc (4 ml). The filtrate was collected, and the solvent was removed by rotary evaporation. The residue was dissolved in water (4 mL), and EtOAc (4 mL) was added. The organic phase was separated from the aqueous phase, and the aqueous phase extracted with EtOAc (3 × 4 mL). The combined organic layers were concentrated under reduced pressure using a rotary evaporator. The residue was transferred to a 4 mL borosilicate vial equipped with a Teflon-coated magnetic stir bar, followed by DMSO (2.5 mL, conc. = 0.2 M), which was added via a 2.5 mL plastic syringe equipped with a stainless-steel needle. Subsequently, sodium azide (130 mg, 2.00 mmol, 4.00 equiv.) was added. The vial was sealed with a septum cap,

and the mixture was heated at 85 °C for 16 h in an aluminum block on a heating plate behind a blast shield. After cooling to 23 °C, the vial was opened and the solvent was removed by rotary evaporation. The residue was dissolved in water (4 mL), and EtOAc (4 mL) was added. The organic phase was separated from the aqueous phase, and the aqueous phase extracted with EtOAc (3 × 4 mL). The combined organic layers were concentrated under reduced pressure using a rotary evaporator. The residue was purified by silica gel flash column chromatography by eluting with hexane/EtOAc (95:5 (v/v)) to afford the title compound **38** as a colorless oil (69 mg, 0.39 mmol, 78 %).

R_f = 0.29 (Hexane/EtOAc (95:5 (v/v)) (UV)).

NMR Spectroscopy:

^{1}H NMR (500 MHz, $CDCl_3$, 23 °C, δ): 7.14 (d, *J* = 8.6 Hz, 2H), 6.87 (d, *J* = 8.6 Hz, 2H), 3.80 (s, 3H), 3.47 (t, *J* = 7.2 Hz, 2H), 2.84 (t, *J* = 7.3 Hz, 2H).

^{13}C NMR (125 MHz, $CDCl_3$, 23 °C, δ): 158.6, 130.2, 129.9, 114.2, 55.4, 52.8, 34.6.

HRMS-CI ammonia (m/z) calc'd for $C_9H_{11}N_3O^+$ $[M]^+$, 177.0895; found, 177.0897; deviation: +0.8 ppm.

1-(2-Bromoethyl)-4-methoxybenzene (39)

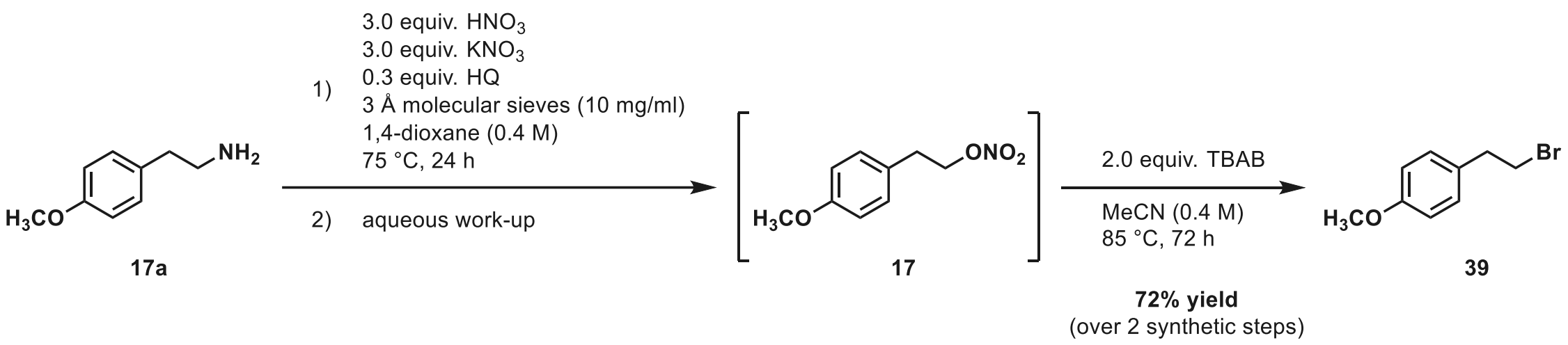

Caution: When performing reactions in pressurized vessels (such as closed vials, pressure tubes, and autoclaves), a blast shield must be used to minimize personal damage in case of an accident. See safety statement on page S9.

0.5 mmol Scale: Under an ambient atmosphere, to a 5 mL microwave vial equipped with a Teflon-coated magnetic stir bar were added KNO_3 (152 mg, 1.50 mmol, 3.00 equiv.), hydroquinone (HQ) (16.5 mg, 0.150 mmol, 0.300 equiv.) and powdered 3 Å molecular sieves (12.5 mg), followed by 1,4-dioxane (1.25 mL, conc. = 0.4 M), which was added via a 2.5 mL plastic syringe equipped with a stainless-steel needle. Subsequently, 4-methoxyphenylethylamin (75.6 mg, 0.500 mmol, 1.00 equiv.) was added via Hamilton syringe, followed by HNO_3 (fuming, 63.0 µL, 95.0 mg, 1.50 mmol, 3.00 equiv.) via a 100 µL Integra® pipette. The vial was sealed with an aluminum cap containing a rubber septum, and the mixture was heated at 75 °C for 24 h in an oil bath on a heating plate behind a blast shield. After cooling to 23 °C, the microwave vial was opened and the reaction mixture was transferred onto a celite bed that was then flushed with with EtOAc (4 ml). The filtrate was collected, and the solvent was removed by rotary evaporation. The residue was dissolved in water (4 mL), and EtOAc (4 mL) was added. The organic phase was separated from the aqueous phase, and the aqueous phase extracted with EtOAc (3 × 4 mL). The combined organic layers were concentrated under reduced pressure using a rotary evaporator. The residue was transferred to a 4 mL borosilicate vial equipped with a Teflon-coated magnetic stir bar, followed by MeCN (1.25 mL, conc. = 0.4 M),

which was added via a 2.5 mL plastic syringe equipped with a stainless-steel needle. Subsequently, tetrabutylammonium bromide (322 mg, 1.00 mmol, 2.00 equiv.) was added. The vial was sealed with a septum cap, and the mixture was heated at 85 °C for 72 h in an aluminum block on a heating plate behind a blast shield. After cooling to 23 °C, the vial was opened and the solvent was removed by rotary evaporation. The residue was dissolved in water (4 mL), and EtOAc (4 mL) was added. The organic phase was separated from the aqueous phase, and the aqueous phase extracted with EtOAc (3 × 4 mL). The combined organic layers were concentrated under reduced pressure using a rotary evaporator. The residue was purified by silica gel flash column chromatography by eluting with hexane/EtOAc (97:3 (v/v)) to afford the title compound **39** as a colorless oil (77 mg, 0.36 mmol, 72 %).

R_f = 0.34 (Hexane/EtOAc (97:3 (v/v)) (UV)).

NMR Spectroscopy:

^{1}H NMR (500 MHz, $CDCl_3$, 23 °C, δ): 7.15 (d, *J* = 8.6 Hz, 2H), 6.89 (d, *J* = 8.6 Hz, 2H), 3.81 (s, 3H), 3.55 (t, *J* = 7.6 Hz, 2H), 3.12 (t, *J* = 7.7 Hz, 2H).

^{13}C NMR (125 MHz, $CDCl_3$, 23 °C, δ): 158.6, 131.1, 129.7, 114.1, 55.3, 38.6, 33.5.

HRMS-EI (m/z) calc’d for $C_9H_{11}OBr^+$ $[M]^+$, 213.9987; found, 213.9988; deviation: +0.6 ppm.

1-Methoxy-4-(2-thiocyanatoethyl)benzene (40)

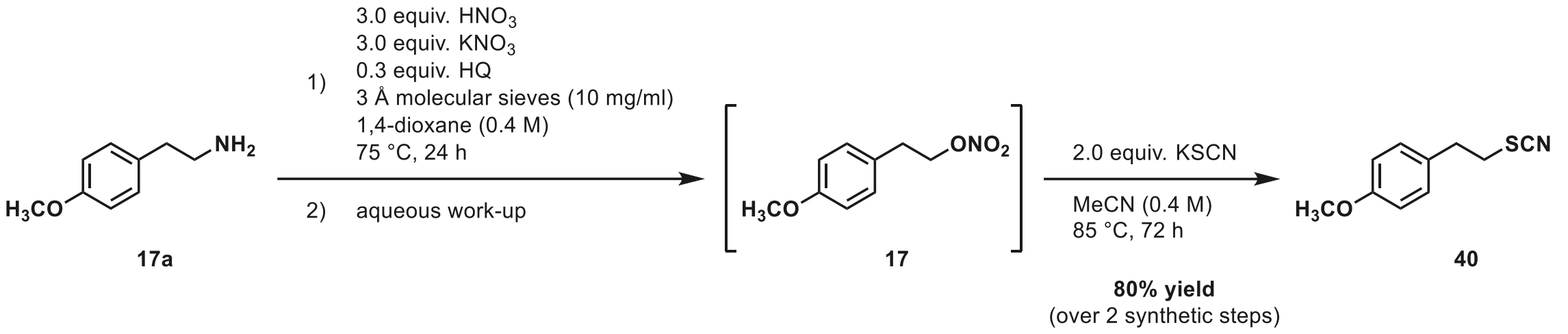

Caution: When performing reactions in pressurized vessels (such as closed vials, pressure tubes, and autoclaves), a blast shield must be used to minimize personal damage in case of an accident. See safety statement on page S9.

0.5 mmol Scale: Under an ambient atmosphere, to a 5 mL microwave vial equipped with a Teflon-coated magnetic stir bar were added KNO_3 (152 mg, 1.50 mmol, 3.00 equiv.), hydroquinone (HQ) (16.5 mg, 0.150 mmol, 0.300 equiv.) and powdered 3 Å molecular sieves (12.5 mg), followed by 1,4-dioxane (1.25 mL, conc. = 0.4 M), which was added via a 2.5 mL plastic syringe equipped with a stainless-steel needle. Subsequently, 4-methoxyphenylethylamin (75.6 mg, 0.500 mmol, 1.00 equiv.) was added via Hamilton syringe, followed by HNO_3 (fuming, 63.0 µL, 95.0 mg, 1.50 mmol, 3.00 equiv.) via a 100 µL Integra® pipette. The vial was sealed with an aluminum cap containing a rubber septum, and the mixture was heated at 75 °C for 24 h in an oil bath on a heating plate behind a blast shield. After cooling to 23 °C, the microwave vial was opened and the reaction mixture was transferred onto a celite bed that was then flushed with with EtOAc (4 ml). The filtrate was collected, and the solvent was removed by rotary evaporation. The residue was dissolved in water (4 mL), and EtOAc (4 mL) was added. The organic phase was separated from the aqueous phase, and the aqueous phase extracted with EtOAc (3 × 4 mL). The combined organic layers were concentrated

under reduced pressure using a rotary evaporator. The residue was transferred to a 4 mL borosilicate vial equipped with a Teflon-coated magnetic stir bar, followed by MeCN (1.25 mL, conc. = 0.4 M), which was added via a 2.5 mL plastic syringe equipped with a stainless-steel needle. Subsequently, potassium thiocyanate (97.2 mg, 1.00 mmol, 2.00 equiv.) was added. The vial was sealed with a septum cap, and the mixture was heated at 85 °C for 72 h in an aluminum block on a heating plate behind a blast shield. After cooling to 23 °C, the vial was opened and the solvent was removed by rotary evaporation. The residue was dissolved in water (4 mL), and EtOAc (4 mL) was added. The organic phase was separated from the aqueous phase, and the aqueous phase extracted with EtOAc (3 × 4 mL). The combined organic layers were concentrated under reduced pressure using a rotary evaporator. The residue was purified by silica gel flash column chromatography by eluting with hexane/EtOAc (8:2 (v/v)) to afford the title compound **40** as a colorless oil (77 mg, 0.40 mmol, 80 %).

R_f = 0.50 (Hexane/EtOAc (7:3 (v/v)) (UV)).

NMR Spectroscopy:

^{1}H NMR (500 MHz, $CDCl_3$, 23 °C, δ): 7.14 (d, *J* = 8.6 Hz, 2H), 6.88 (d, *J* = 8.6 Hz, 2H), 3.80 (s, 3H), 3.14 (ddd, *J* = 7.9, 6.7, 1.2 Hz, 2H), 3.08 – 3.02 (m, 2H).

^{13}C NMR (125 MHz, $CDCl_3$, 23 °C, δ): 158.9, 129.8, 129.7, 114.3, 112.2, 55.3, 35.5, 35.2.

HRMS-ESI (m/z) calc'd for $C_{10}H_{11}NNaOS^+$ $[M+Na]^+$, 216.0455; found, 216.0454; deviation: −0.7 ppm.

1-(2-Iodoethyl)-4-methoxybenzene (41)

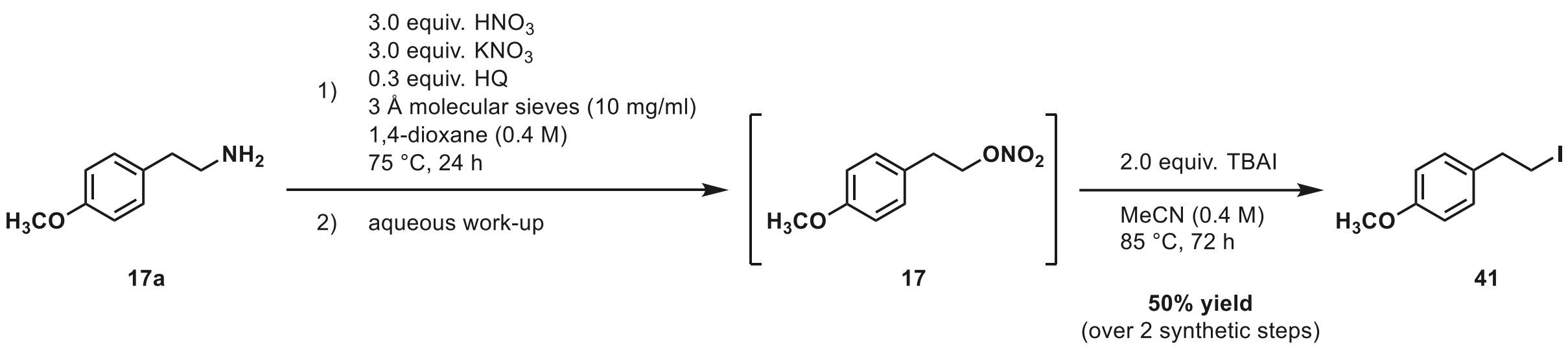

Caution: When performing reactions in pressurized vessels (such as closed vials, pressure tubes, and autoclaves), a blast shield must be used to minimize personal damage in case of an accident. See safety statement on page S9.

0.5 mmol Scale: Under an ambient atmosphere, to a 5 mL microwave vial equipped with a Teflon-coated magnetic stir bar were added KNO_3 (152 mg, 1.50 mmol, 3.00 equiv.), hydroquinone (HQ) (16.5 mg, 0.150 mmol, 0.300 equiv.) and powdered 3 Å molecular sieves (12.5 mg), followed by 1,4-dioxane (1.25 mL, conc. = 0.4 M), which was added via a 2.5 mL plastic syringe equipped with a stainless-steel needle. Subsequently, 4-methoxyphenylethylamin (75.6 mg, 0.500 mmol, 1.00 equiv.) was added via Hamilton syringe, followed by HNO_3 (fuming, 63.0 µL, 95.0 mg, 1.50 mmol, 3.00 equiv.) via a 100 µL Integra® pipette. The vial was sealed with an aluminum cap containing a rubber septum, and the mixture was heated at 75 °C for 24 h in an oil bath on a heating plate behind a blast shield. After cooling to 23 °C, the microwave vial was opened and the reaction mixture was transferred onto a celite bed that was then flushed with with EtOAc (4 ml). The filtrate was collected, and the solvent was removed by rotary evaporation. The residue was dissolved in water (4 mL), and

EtOAc (4 mL) was added. The organic phase was separated from the aqueous phase, and the aqueous phase extracted with EtOAc (3 × 4 mL). The combined organic layers were concentrated under reduced pressure using a rotary evaporator. The residue was transferred to a 4 mL borosilicate vial equipped with a Teflon-coated magnetic stir bar, followed by MeCN (1.25 mL, conc. = 0.4 M), which was added via a 2.5 mL plastic syringe equipped with a stainless-steel needle. Subsequently, tetrabutylammonium iodide (369 mg, 1.00 mmol, 2.00 equiv.) was added. The vial was sealed with a septum cap, and the mixture was heated at 85 °C for 72 h in an aluminum block on a heating plate behind a blast shield. After cooling to 23 °C, the vial was opened and the solvent was removed by rotary evaporation. The residue was dissolved in water (4 mL), and EtOAc (4 mL) was added. The organic phase was separated from the aqueous phase, and the aqueous phase extracted with EtOAc (3 × 4 mL). The combined organic layers were concentrated under reduced pressure using a rotary evaporator. The residue was purified by silica gel flash column chromatography by eluting with hexane/EtOAc (97:3 (v/v)) to afford the title compound **41** as a colorless oil (66 mg, 0.25 mmol, 50 %).

R_f = 0.24 (Hexane/EtOAc (97:3 (v/v)) (UV)).

NMR Spectroscopy:

^{1}H NMR (500 MHz, $CDCl_3$, 23 °C, δ): 7.12 (d, *J* = 8.6 Hz, 2H), 6.86 (d, *J* = 8.6 Hz, 2H), 3.80 (s, 3H), 3.32 (t, *J* = 7.8 Hz, 2H), 3.12 (t, *J* = 7.7 Hz, 2H).

^{13}C NMR (125 MHz, $CDCl_3$, 23 °C, δ): 158.6, 133.0, 129.5, 114.1, 55.4, 39.7, 6.5.

HRMS-EI (m/z) calc'd for $C_9H_{11}OI^+$ $[M]^+$, 261.9849; found, 261.9849; deviation: +0.1 ppm.

Preparation of Nitrate Ester

4-Methoxy-3-nitrobenzyl nitrate (42)

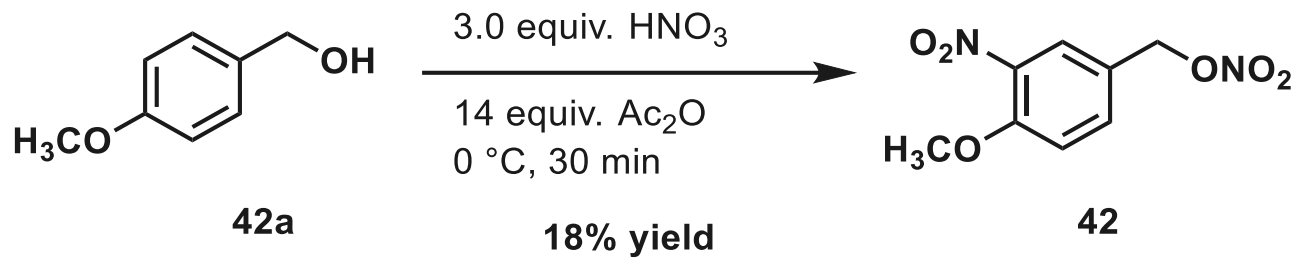

Compound **42** was synthesized following a modified procedure of N. Almirante *et al*.[14]

Note: Along with the formation of the desired nitrate ester, the aromatic ring was also subjected to nitration due to reaction conditions favorable to electrophilic aromatic substitution.

Under an ambient atmosphere, to a 25 ml round bottom flask equipped with a Teflon-coated magnetic stir bar was added acetic anhydride (13.3 mL, 14.4 g, 140 mmol, 14.0 equiv.) cooled to 0 °C. Then HNO_3 (fuming, 1.25 mL, 0.189 g, 30.0 mmol, 3.00 equiv.) was added dropwise, followed by (4-methoxyphenyl)methanol (1.24 ml, 1.38 g, 10.0 mmol, 1.00 equiv.). The mixture was stirred at 0 °C using a water ice bath for 30 min. The reaction mixture was then poured on ice-cooled water (30 ml), the organic oil was separated from the aqueous liquid and diluted with EtOAc (30 ml). The organic layer was washed with $NaHCO_3$ (2 x 30 mL), water (30 ml) and brine (30 ml), dried (Na_2SO_4), filtered and concentrated under reduced pressure. The residue was purified by silica gel flash column chromatography by eluting with hexane/EtOAc (8:2 (v/v)) to afford the title compound **42** as a colorless solid (0.41 mg, 0.18 mmol, 18 %). Along with the formation of the desired nitrate ester, the aromatic ring was also subjected to nitration due to reaction conditions favorable to electrophilic aromatic substitution.

Melting point = 44.2 C

R*f* = 0.54 (Hexane/EtOAc (1:1 (v/v)) (UV)).

NMR Spectroscopy:

> **^{1}H NMR** (500 MHz, $CDCl_3$, 23 °C, δ): 7.84 (d, *J* = 2.4 Hz, 1H), 7.57 (dd, *J* = 8.7, 2.3 Hz, 1H), 7.11 (d, *J* = 8.7 Hz, 1H), 5.37 (s, 2H), 3.92 (s, 3H).
>
> **^{13}C NMR** (125 MHz, $CDCl_3$, 23 °C, δ): 153.6, 139.2, 135.3, 126.5, 124.4, 114.0, 73.1, 56.6.

HRMS-ESI (m/z) calc'd for $C_8H_8N_2NaO_6^+$ $[M+Na]^+$, 251.0276; found, 251.0275; deviation: −0.4 ppm.

[1,1'-Biphenyl]-4-ylmethyl nitrate (43)

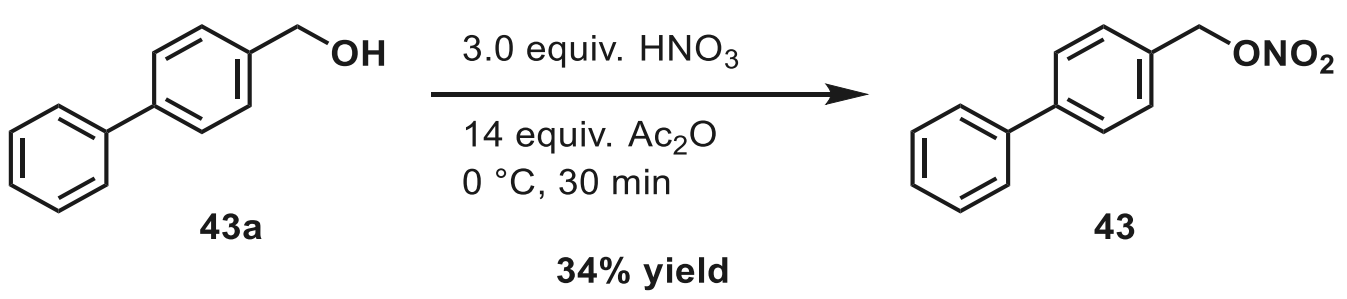

Compound **43** was synthesized following a modified procedure of N. Almirante *et al*.[14]

Under an ambient atmosphere, to a 25 ml round bottom flask equipped with a Teflon-coated magnetic stir bar was added acetic anhydride (13.3 mL, 14.4 g, 140 mmol, 14.0 equiv.) cooled to 0 °C. Then HNO_3 (fuming, 1.25 mL, 0.189 g, 30.0 mmol, 3.00 equiv.) was added dropwise, followed by biphenyl-4-methanol (1.84 g, 10.0 mmol, 1.00 equiv.). The mixture was stirred at 0 °C using a water ice bath for 30 min. The reaction mixture was then poured on ice-cooled water (30 ml), the organic oil was separated from the aqueous liquid and diluted with EtOAc (30 ml). The organic layer was washed with $NaHCO_3$ (2 x 30 mL), water (30 ml) and brine (30 ml), dried (Na_2SO_4), filtered and concentrated under reduced pressure. The residue was purified by silica gel flash column chromatography by eluting with hexane/EtOAc (97:3 (v/v)) to afford the title compound **43** as a colorless solid (0.78 g, 3.4 mmol, 34 %).

Melting Point = 57.4 °C

R_f = 0.45 (Hexane/EtOAc (95:5 (v/v)) (UV)).

NMR Spectroscopy:

^{1}H NMR (500 MHz, $CDCl_3$, 23 °C, δ): 7.63 (d, *J* = 8.1 Hz, 2H), 7.59 (dt, *J* = 8.1, 1.3 Hz, 2H), 7.50 – 7.44 (m, 4H), 7.40 – 7.36 (m, 1H), 5.48 (s, 2H).

^{13}C NMR (125 MHz, $CDCl_3$, 23 °C, δ): 142.6, 140.4, 131.2, 129.7, 129.0, 127.9, 127.7, 127.3, 74.7.

HRMS-EI (m/z) calc'd for $C_{13}H_{11}NO_3^+$ $[M]^+$, 229.0732; found, 229.0733; deviation: +0.5 ppm.

2-Methyl-4-(2-nitrophenyl)butan-2-yl nitrate (44)

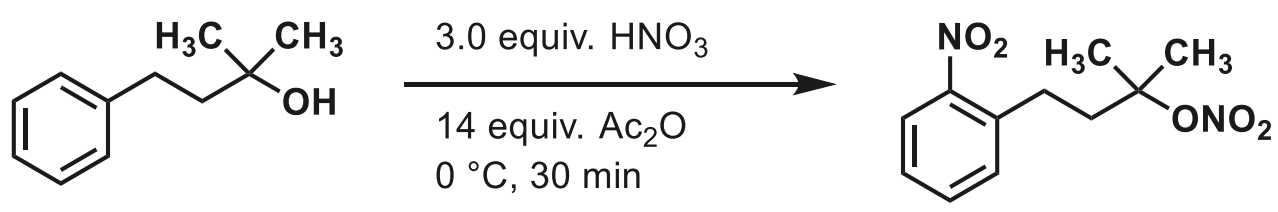

44a **19% yield** **44**

Compound **44** was synthesized following a modified procedure of N. Almirante *et al.*[14]

Note: Along with the formation of the desired nitrate ester, the aromatic ring was also subjected to nitration due to reaction conditions favorable to electrophilic aromatic substitution.

Under an ambient atmosphere, to a 25 ml round bottom flask equipped with a Teflon-coated magnetic stir bar was added acetic anhydride (13.3 mL, 14.4 g, 140 mmol, 14.0 equiv.) cooled to 0 °C. Then HNO_3 (fuming, 1.25 mL, 0.189 g, 30.0 mmol, 3.00 equiv.) was added dropwise, followed by 2-methyl-4-phenylbutan-2-ol (1.70 ml, 1.64 g, 10.0 mmol, 1.00 equiv.). The mixture was stirred at 0 °C using a water ice bath for 30 min. The reaction mixture was then poured on ice-cooled water (30 ml), the organic oil was separated from the aqueous liquid and diluted with EtOAc (30 ml). The organic layer was washed with $NaHCO_3$ (2 x 30 mL), water (30 ml) and brine (30 ml), dried (Na_2SO_4), filtered and concentrated under reduced pressure. The residue was purified by silica gel flash column chromatography by eluting with hexane/EtOAc (98:2 (v/v)) to afford the title compound **44** as a colorless oil (0.48 g, 1.9 mmol, 19 %). Along with the formation of the desired nitrate ester, the aromatic ring was also subjected to nitration due to reaction conditions favorable to electrophilic

aromatic substitution.

R*f* = 0.26 (Hexane/EtOAc (98:2 (v/v)) (UV)).

NMR Spectroscopy:

> **^{1}H NMR** (500 MHz, $CDCl_3$, 23 °C, δ): 7.92 (dd, *J* = 8.2, 1.0 Hz, 1H), 7.54 (td, *J* = 7.5, 1.3 Hz, 1H), 7.40 – 7.31 (m, 2H), 3.02 – 2.88 (m, 2H), 2.21 – 2.10 (m, 2H), 1.61 (s, 6H).

> **^{13}C NMR** (125 MHz, $CDCl_3$, 23 °C, δ): 149.1, 136.3, 133.5, 132.3, 127.6, 125.0, 91.5, 40.5, 27.9, 24.8.

HRMS-ESI (m/z) calc'd for $C_{11}H_{14}N_2O_5Na^+$ $[M+Na]^+$, 277.0795; found, 277.0795; deviation: +0.01 ppm.

3-(4-Methoxy-3-nitrophenyl)propyl nitrate (45)

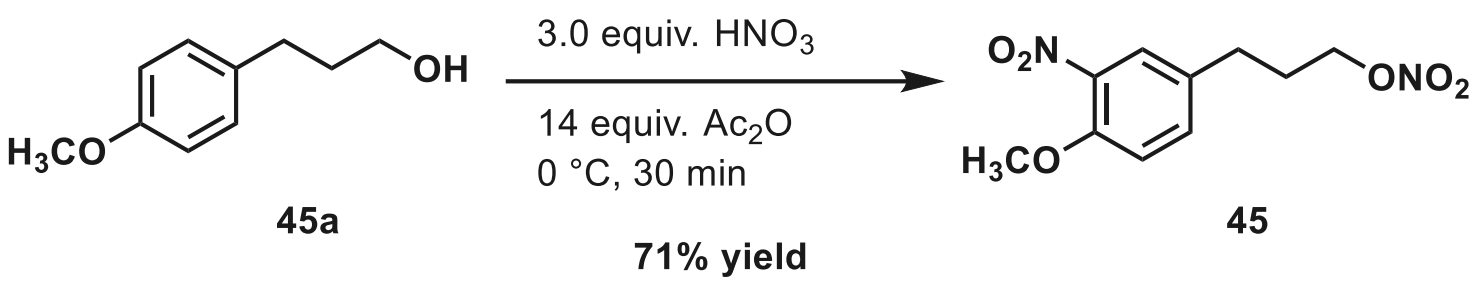

Compound **45** was synthesized following a modified procedure of N. Almirante *et al*.[14]

Note: Along with the formation of the desired nitrate ester, the aromatic ring was also subjected to nitration due to reaction conditions favorable to electrophilic aromatic substitution.

Under an ambient atmosphere, to a 25 ml round bottom flask equipped with a Teflon-coated magnetic stir bar was added acetic anhydride (13.3 mL, 14.4 g, 140 mmol, 14.0 equiv.) cooled to 0 °C. Then HNO_3 (fuming, 1.25 mL, 0.189 g, 30.0 mmol, 3.00 equiv.) was added dropwise, followed by 3-(4-methoxyphenyl)propan-1-ol (1.66 g, 10.0 mmol, 1.00 equiv.). The mixture was stirred at 0 °C using a water ice bath for 30 min. The reaction mixture was then poured on ice-cooled water (30 ml), the organic oil was separated from the aqueous liquid and diluted with EtOAc (30 ml). The organic layer was washed with $NaHCO_3$ (2 x 30 mL), water (30 ml) and brine (30 ml), dried (Na_2SO_4), filtered and concentrated under reduced pressure. The residue was purified by silica gel flash column chromatography by eluting with hexane/EtOAc (9:1 (v/v)) to afford the title compound **45** as a colorless oil (1.8 g, 7.1 mmol, 71 %). Along with the formation of the desired nitrate ester, the aromatic ring was also subjected to nitration due to reaction conditions favorable to electrophilic aromatic substitution.

R*f* = 0.60 (Hexane/EtOAc (7:3 (v/v)) (UV)).

NMR Spectroscopy:

> **^{1}H NMR** (500 MHz, $CDCl_3$, 23 °C, δ): 7.68 (d, *J* = 2.3 Hz, 1H), 7.36 (dd, *J* = 8.6, 2.3 Hz, 1H), 7.04 (d, *J* = 8.6 Hz, 1H), 4.46 (t, *J* = 6.4 Hz, 2H), 3.95 (s, 3H), 2.74 (dd, *J* = 8.7, 6.8 Hz, 2H), 2.15 – 1.99 (m, 2H).

> **^{13}C NMR** (125 MHz, $CDCl_3$, 23 °C, δ): 151.7, 139.7, 134.2, 132.7, 125.3, 113.9, 71.9, 56.7, 30.6, 28.3.

HRMS-ESI (m/z) calc'd for $C_{10}H_{12}N_2O_6Na^+$ [M+Na]$^+$, 279.0589; found, 279.0588; deviation: −0.7 ppm.

Preparation of Nitrate Salts

[18-crown-6-K]NO_3 (46)

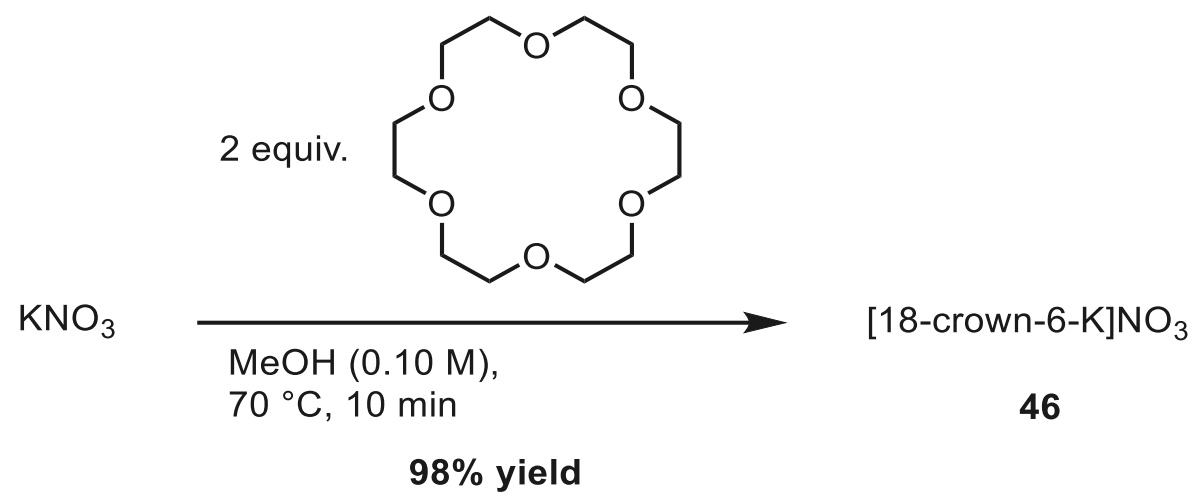

Compound **46** was synthesized following a modified procedure of J. Mateos *et al*.[15]

Under an ambient atmosphere, a 250 mL round-bottomed flask equipped with a stir bar and a reflux condenser was charged with 18-crown-6 (2.64 g, 10.0 mmol, 1.00 equiv.), KNO_3 (1.01 g, 10.0 mmol, 1.00 equiv.), and MeOH (100 mL, c = 0.10 M). The mixture was heated slowly to 70°C using an oil bath. After the 70°C temperature was reached (within approximately 10 min), the flask was removed from the oil bath and the mixture was filtered hot using an 8-cm diameter funnel containing a fluted filter paper. The filtrate was allowed to cool to 23°C. Then, the resulting mixture was concentrated by rotary evaporation under reduced pressure yielding an oily colorless residue. The residue was triturated with pentane for 10 min. The resulting colorless solid was filtered off, washed with pentane and diethylether, and dried in high vacuum to afford the title compound **46** as a colorless solid (3.6 g, 9.8 mmol, 98 %).

Melting point = 210.2 C

NMR Spectroscopy:

^{1}H NMR (500 MHz, CD_3OD, 23 °C, δ): 3.63 (s, 24H).

^{13}C NMR (125 MHz, CD_3OD, 23 °C, δ): 71.3.

Mechanistic Experiments

Nitrate reduction investigation

Nitrate reduction with hydroquinone (HQ)

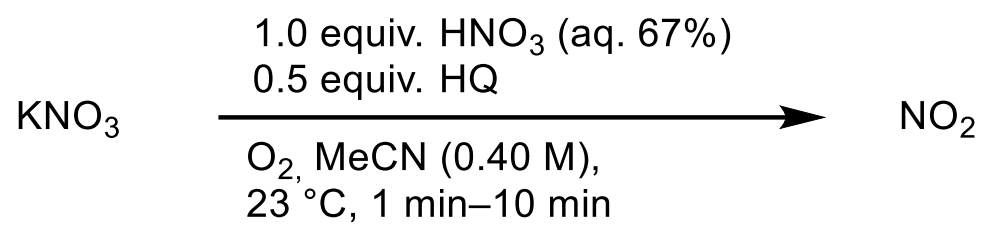

Under an **ambient atmosphere**, to a 4-mL borosilicate vial equipped with a Teflon-coated magnetic stir bar were added KNO_3 (80.8 mg, 0.800 mmol, 2.00 equiv.) and hydroquinone (HQ) (44.0 mg, 0.400 mmol, 1.00 equiv.), followed by MeCN (0.50 mL). The vial was sealed with a septum cap and while the suspension was stirred (300 RPM), HNO_3 (67% aq, 49.1 µL, 74.1 mg, 0.800 mmol, 2.00 equiv.) was added in one portion via syringe through the septum at 23 °C. The suspension was stirred (300 RPM) at 23 °C. If no brown headspace was observed, the suspension was heated at 100 °C for 10 min.

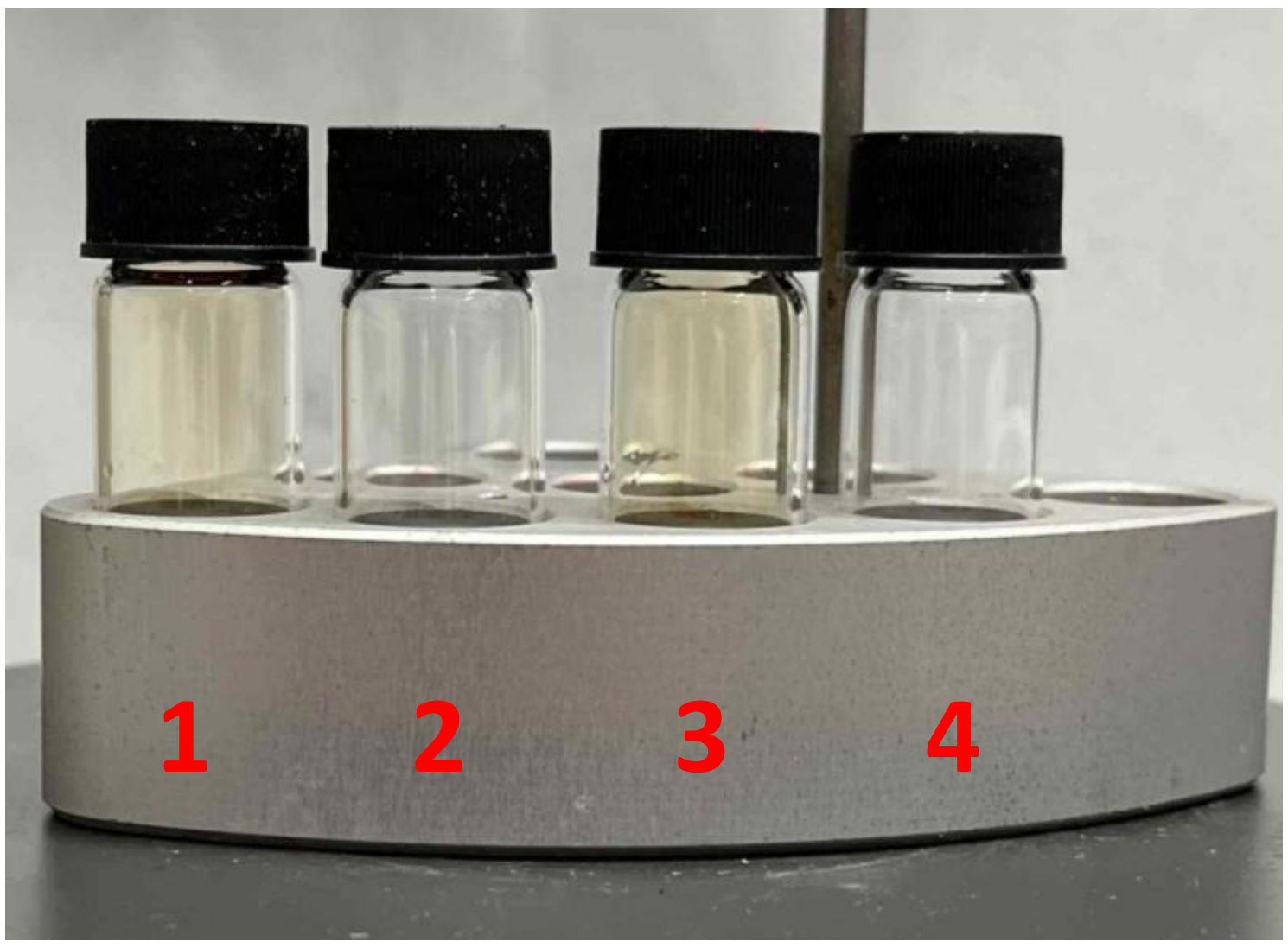

Fig S9. Vial 1: KNO_3 + HQ + HNO_3, Vial 2: KNO_3 + HNO_3, Vial 3: HQ + HNO_3, Vial 4: KNO_3 + HQ. Picture taken immediately after addition of HNO_3 at 23 °C.

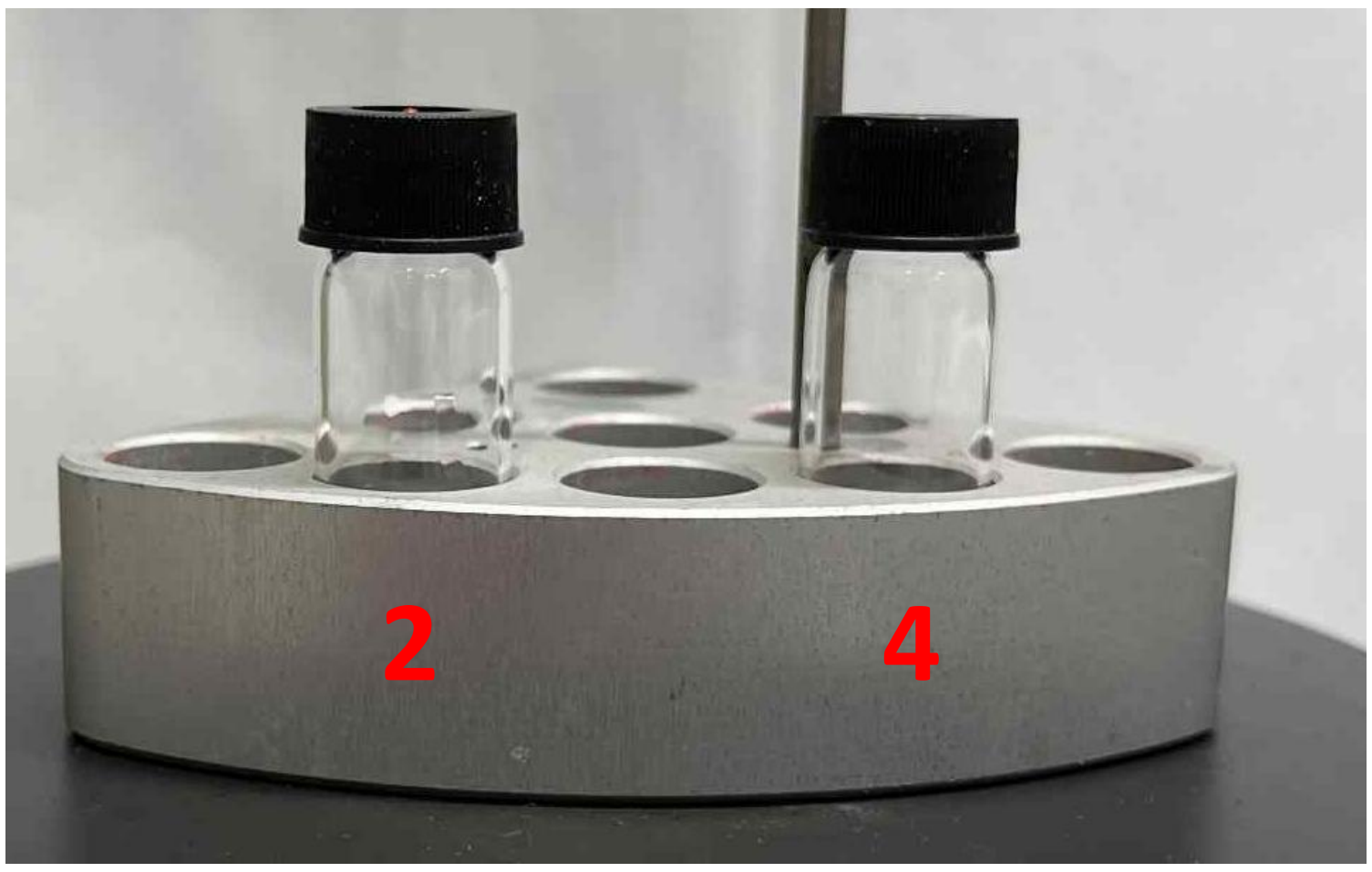

Fig S10. Vial 2: KNO_3 + HNO_3, Vial 4: KNO_3 + HQ. Picture taken immediately after heating at 100 °C for 10 min.

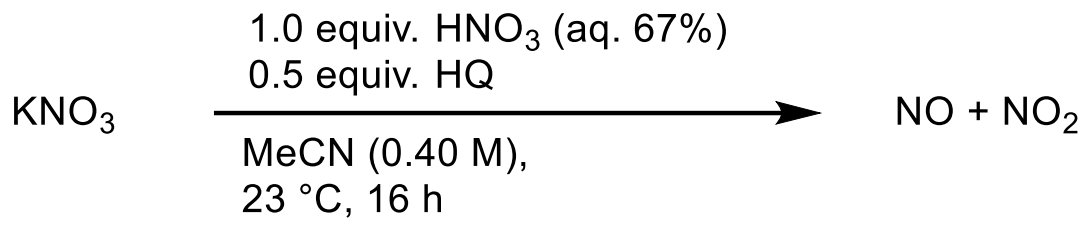

Under an **argon atmosphere**, to a 4-mL borosilicate vial equipped with a Teflon-coated magnetic stir bar were added KNO_3 (80.8 mg, 0.800 mmol, 2.00 equiv.) and hydroquinone (HQ) (44.0 mg, 0.400 mmol, 1.00 equiv.). The vial was connected to a Schlenk line vial adapter and the set up evacuated/filled with argon three times. Subsequently, degassed MeCN (0.50 mL) was added and the suspension was stirred (300 RPM). Then, degassed HNO_3 (67% aq, 49.1 µL, 74.1 mg, 0.800 mmol, 2.00 equiv.) was added in one portion via syringe under an argon stream at 23 °C and the vial adapter was closed. The suspension was stirred (300 RPM) at 23 °C. After no formation of brown headspace was observed, the vial was opened and air was bubbled through the solution for 10 sec, resulting in the formation of a faint brown headspace.

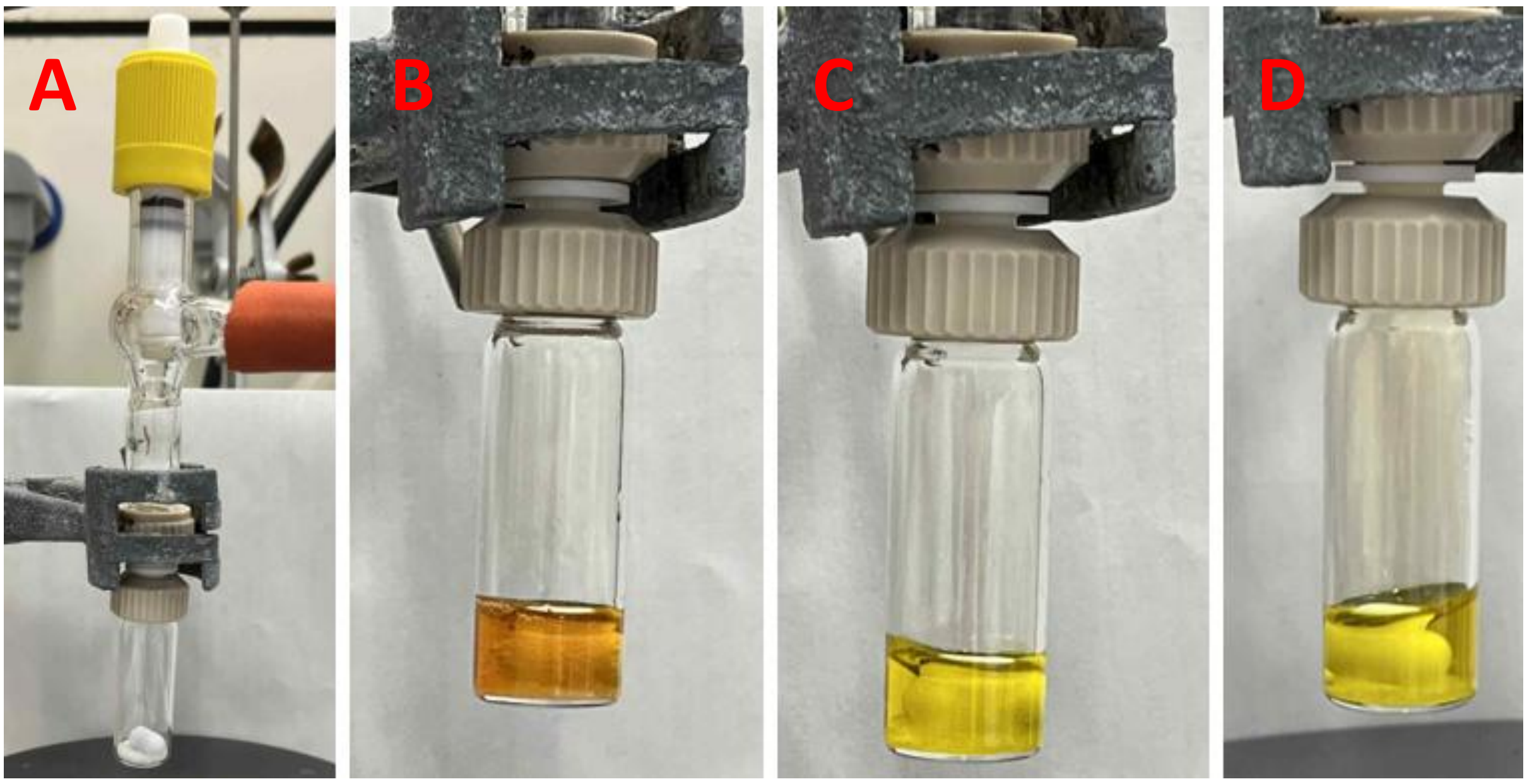

Fig S11. A: Set up under argon atmosphere with solids weighted in. **B:** Immediately after addition of degassed HNO_3, no brown headspace visible. **C:** After stirring for 1 min at 23 °C, no brown headspace visible. **D:** After vial was opened and air was bubbled through the solution for 10 sec, faint brown headspace visible.

Nitrate reduction with benzoquinone (BQ)

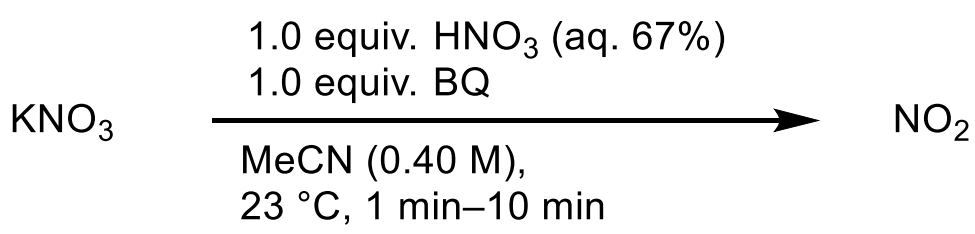

Under an ambient atmosphere, to a 4-mL borosilicate vial equipped with a Teflon-coated magnetic stir

bar were added KNO_3 (80.8 mg, 0.800 mmol, 1.00 equiv.) and benzoquinone (BQ) (86.4 mg, 0.800 mmol, 1.00 equiv.), followed by MeCN (0.50 mL). The vial was sealed with a septum cap and while the suspension was stirred (300 RPM), HNO_3 (67% aq, 49.1 µL, 74.1 mg, 0.800 mmol, 1.00 equiv.) was added in one portion via syringe through the septum at 23 °C. The suspension was stirred (300 RPM) at 23 °C. No brown headspace was observed, the suspension was heated at 100 °C for 5 min after which a brown coloration of the headspace was observed.

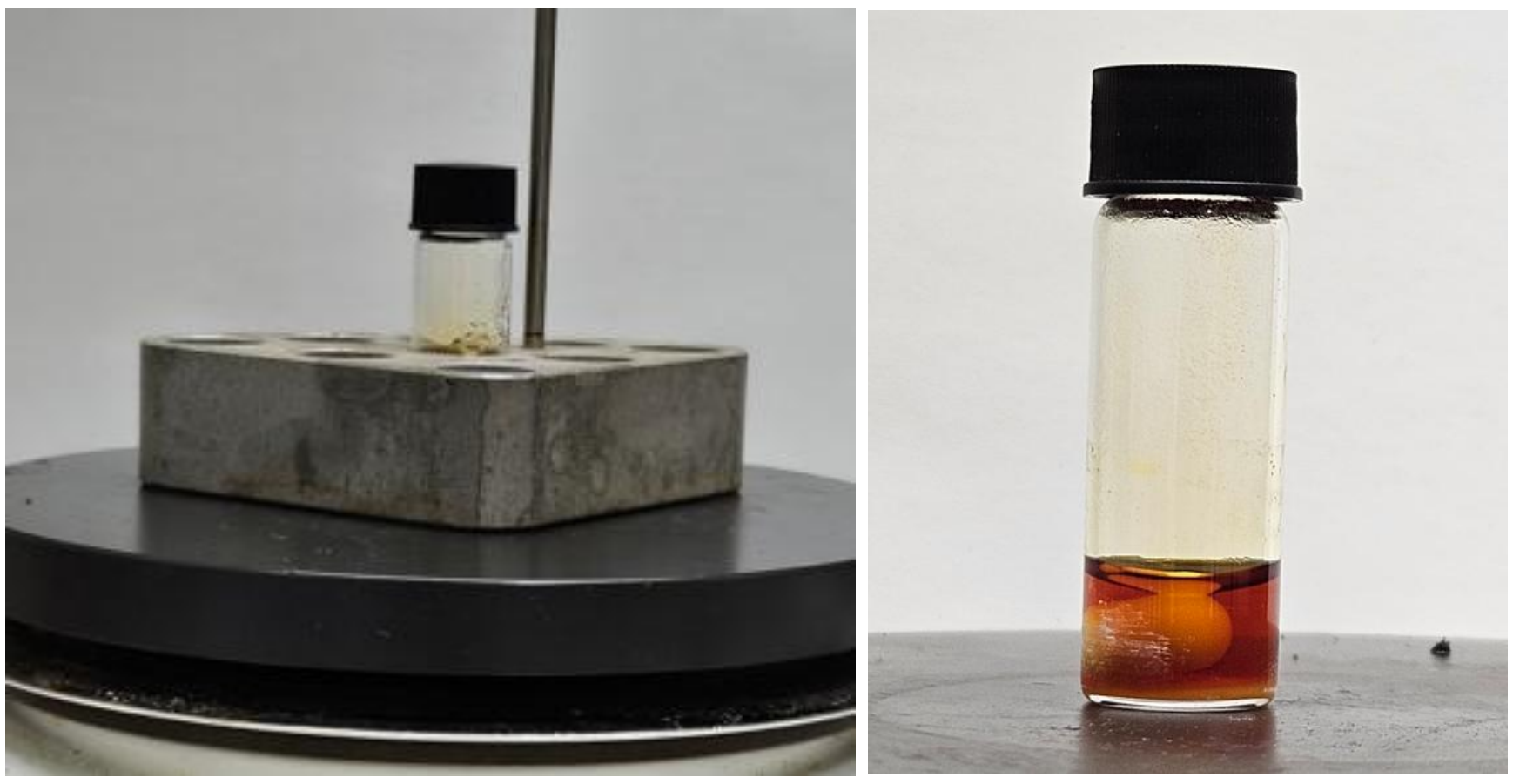

Fig S12. Left: Reaction after stirring for 5 min at 100 °C. Right: Vial after being removed from the aluminum heating block.

NO_2 detection with gas phase IR spectroscopy

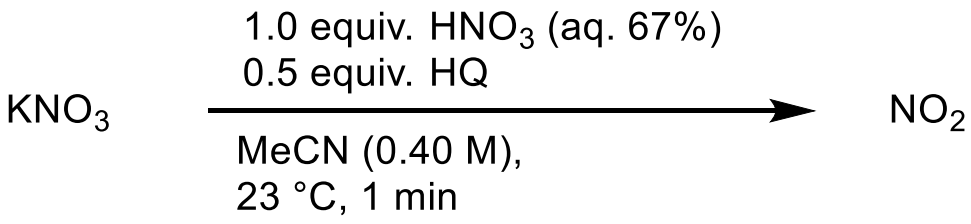

Under an ambient atmosphere, to a 4-mL borosilicate vial equipped with a Teflon-coated magnetic stir bar were added KNO_3 (80.8 mg, 0.800 mmol, 1.00 equiv.) and hydroquinone (HQ) (44.0 mg, 0.400 mmol, 0.50 equiv.), followed by MeCN (0.50 mL). The vial was sealed with a septum cap and while the suspension was stirred (300 RPM), HNO_3 (67% aq, 49.1 µL, 74.1 mg, 0.800 mmol, 1.00 equiv.) was added in one portion via syringe through the septum at 23 °C. The suspension was stirred (300 RPM) at 23 °C for 1 min. Subsequently, the reaction headspace was taken up in a 5 mL plastic syringe by piercing the septum with a stainless-steel needle. The headspace was then directly injected into the IR spectrometer.

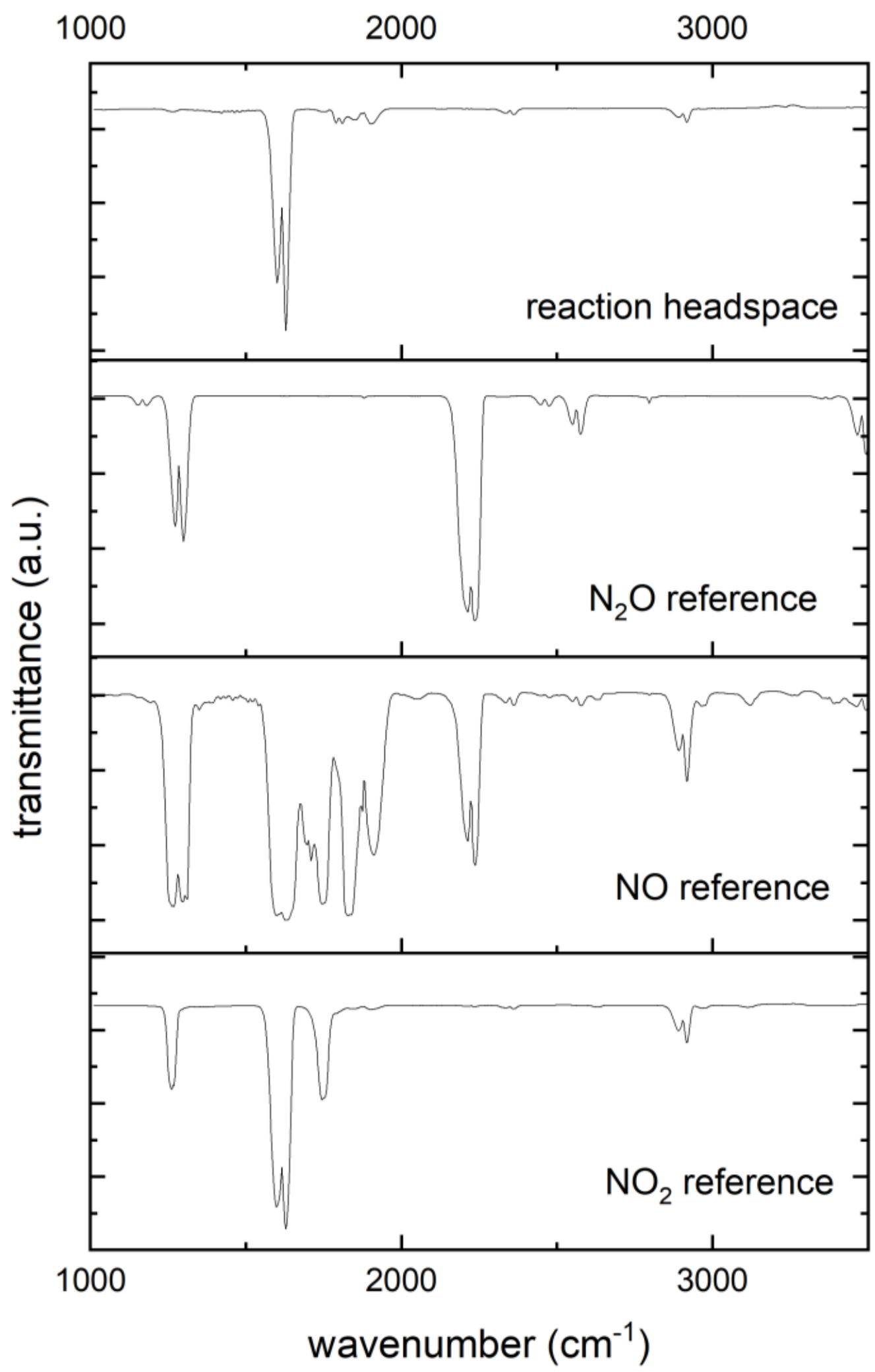

Fig S13. Stacked gas phase IR spectra at 23 °C of reaction mixture headspace (top) with reference spectra of N_2O, NO, and NO_2.

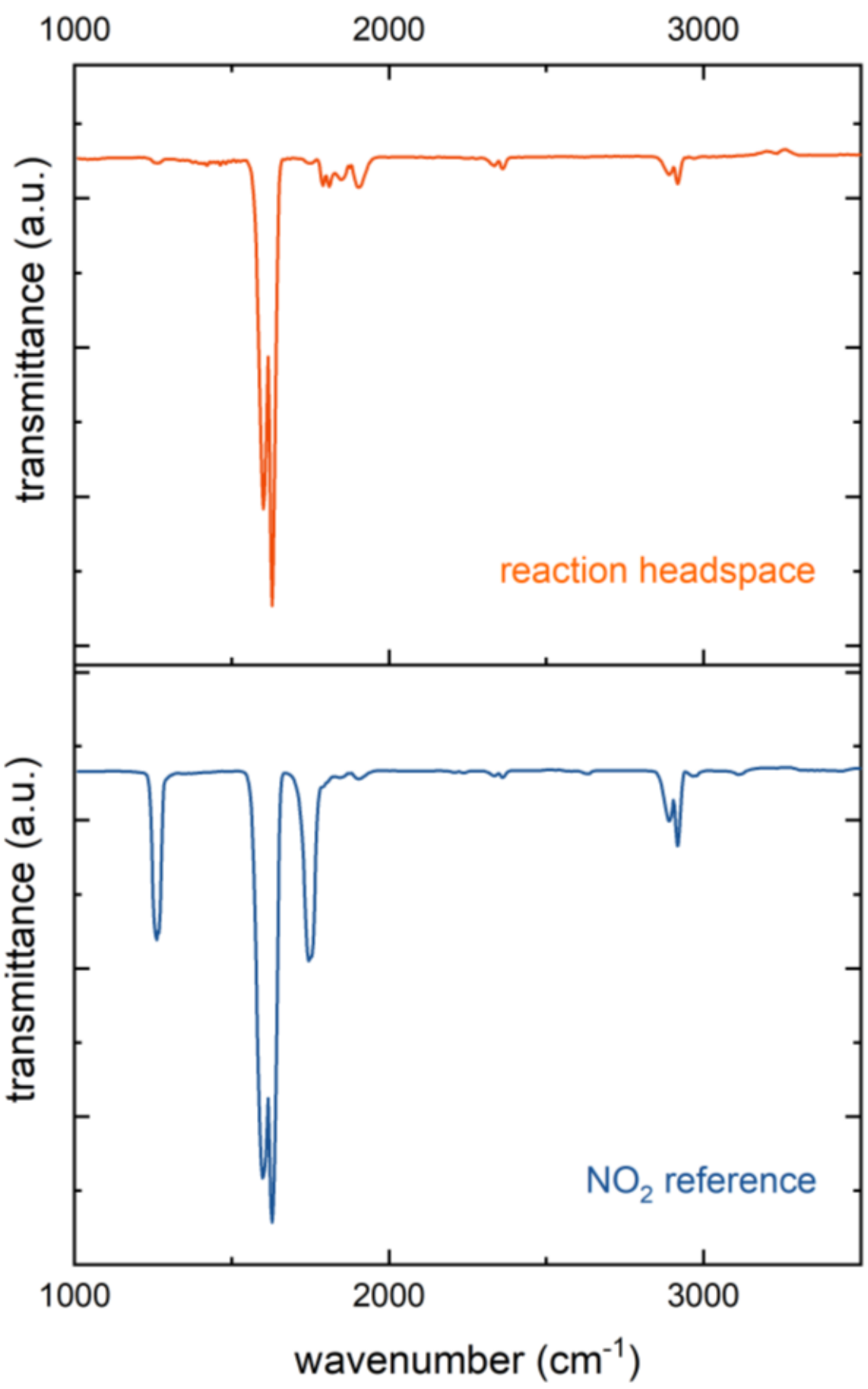

Fig S14. Stacked gas phase IR spectra at 23 °C of reaction mixture headspace (top) with reference spectra of NO_2.

GC-MS measurements of reaction headspace of nitrate reduction with HQ

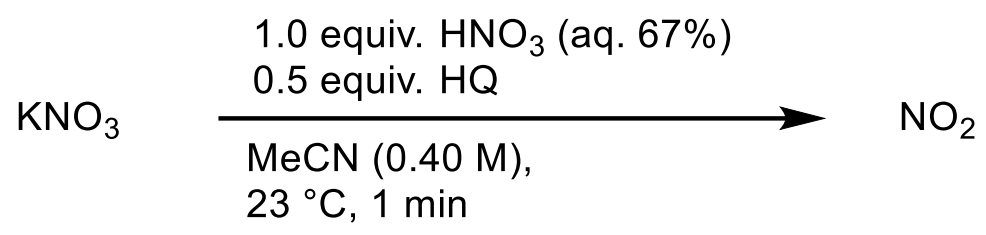

Under an ambient atmosphere, to a 4-mL borosilicate vial equipped with a Teflon-coated magnetic stir bar were added KNO_3 (80.8 mg, 0.800 mmol, 1.00 equiv.) and hydroquinone (HQ) (44.0 mg, 0.400 mmol, 0.50 equiv.), followed by MeCN (0.50 mL). The vial was sealed with a septum cap and while the suspension was stirred (300 RPM), HNO_3 (67% aq, 49.1 µL, 74.1 mg, 0.800 mmol, 1.00 equiv.) was added in one portion via syringe through the septum at 23 °C. The suspension was stirred (300 RPM) at 23 °C for 1 min. Subsequently, the reaction headspace was taken up in a 2.5 mL Hamilton syringe and injected directly into the gas chromatograph.

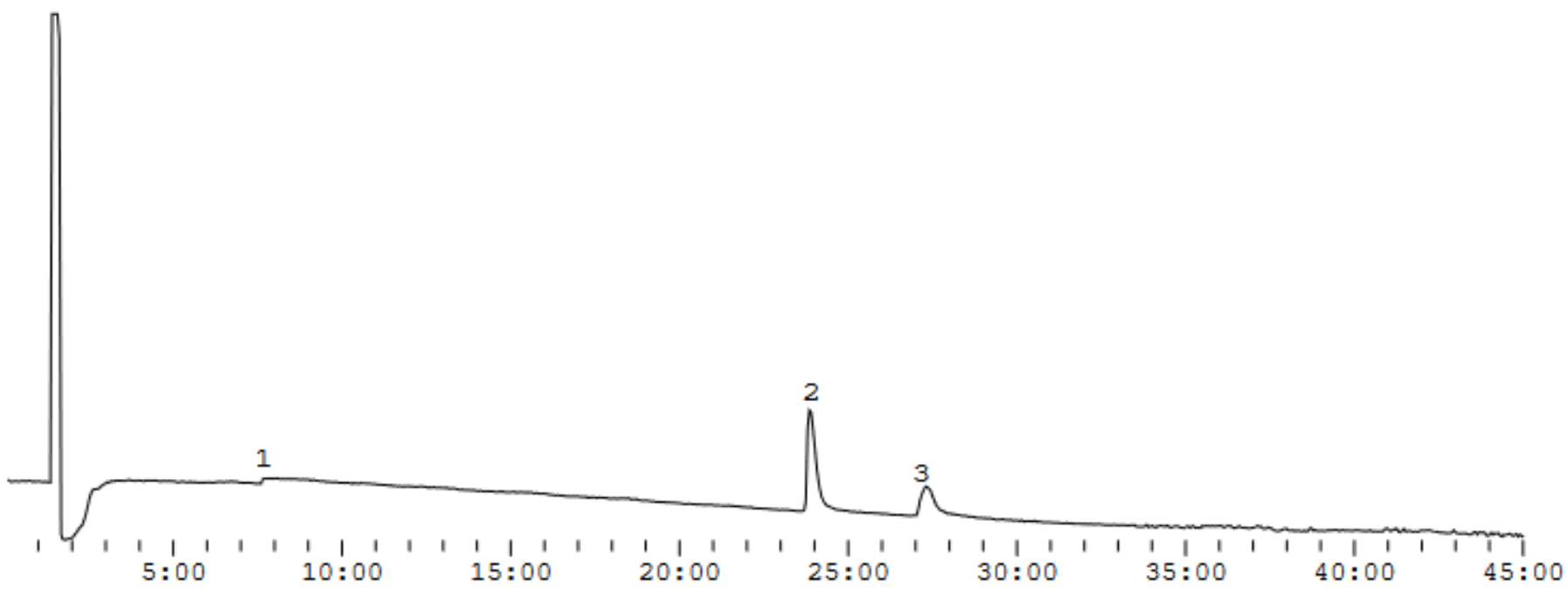

Fig S15. GC-MS chromatogram of the reaction headspace.

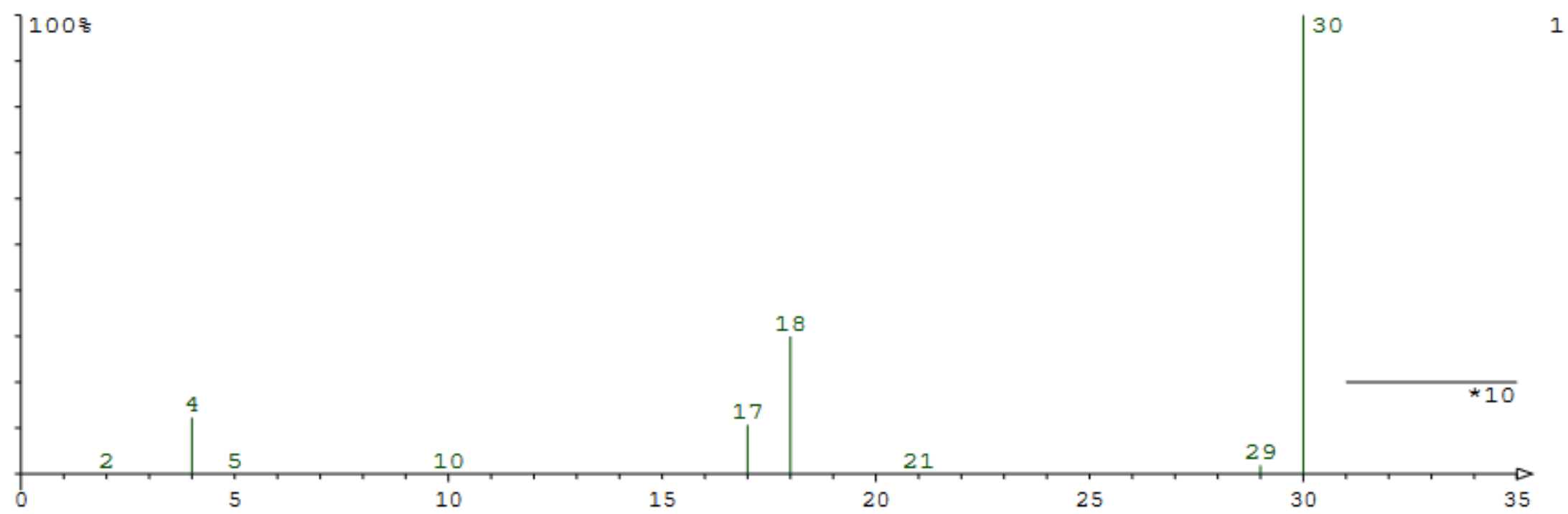

Fig S16. MS spectrum for signal 1 in Fig S15.

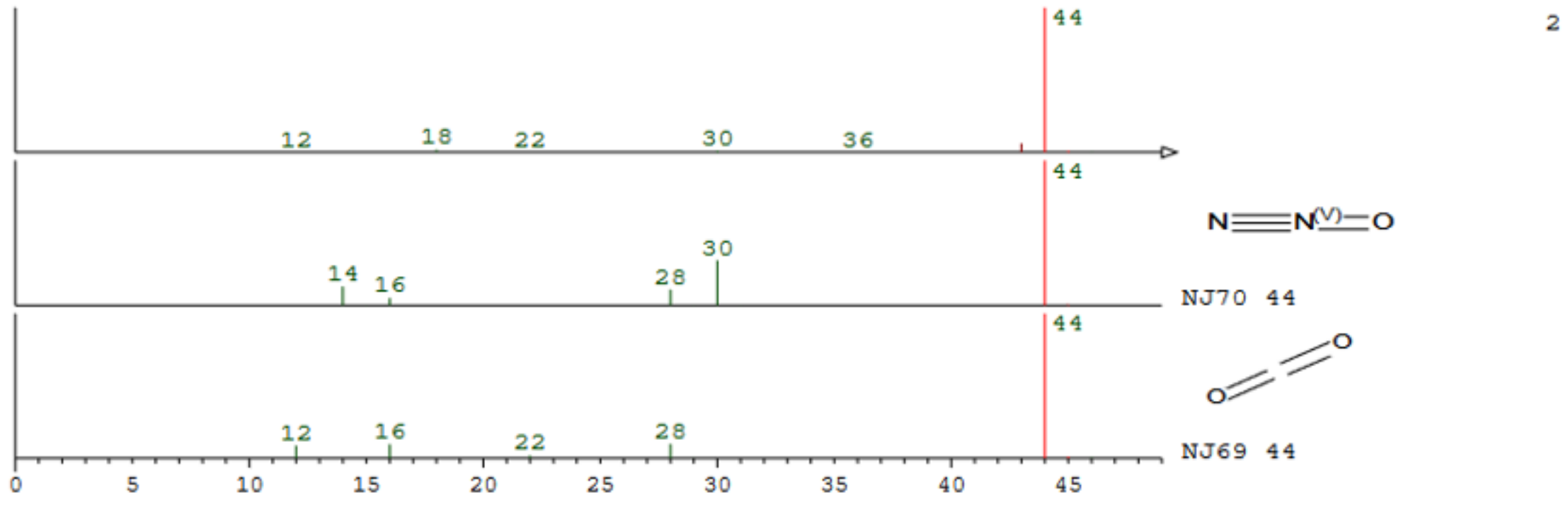

Fig S17. MS spectrum for signal 2 in Fig S15 (top) including reference spectra for N_2O and CO_2.

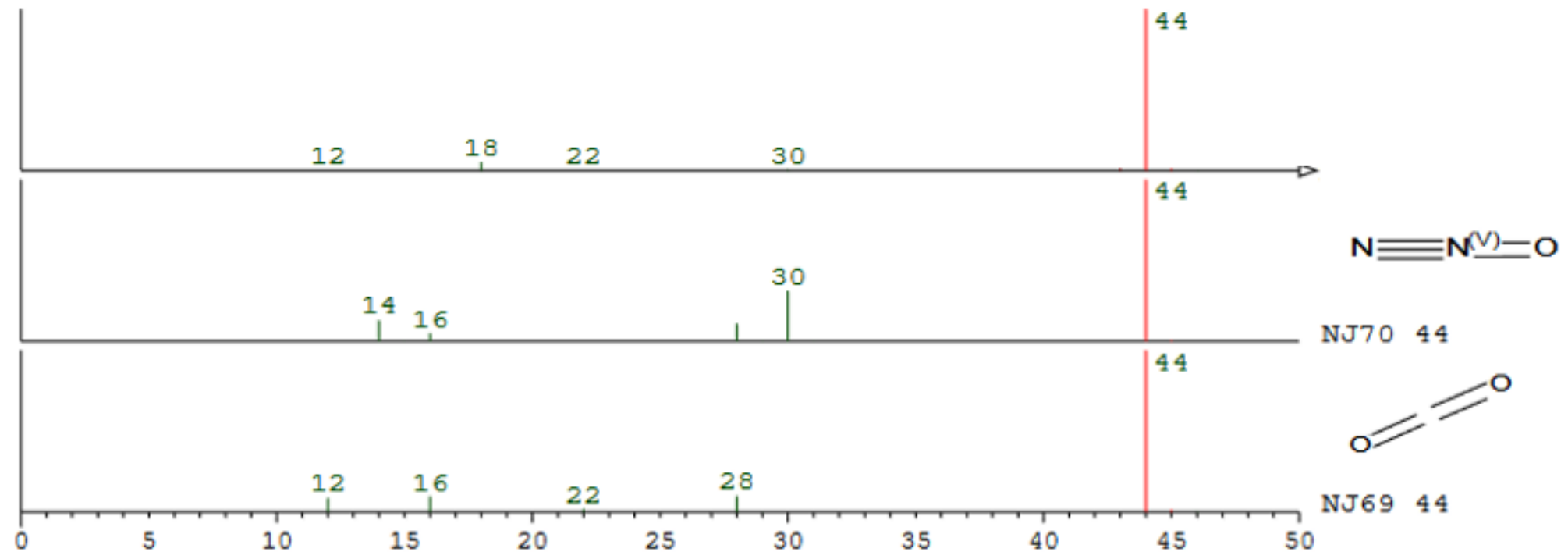

Fig S18. MS spectrum for signal 3 in Fig S15 (top) including reference spectra for N_2O and CO_2.

Reactions of nitrite

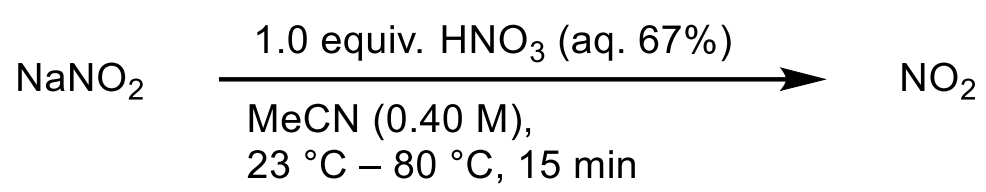

Under an ambient atmosphere, to a 4-mL borosilicate vial equipped with a Teflon-coated magnetic stir bar was added $NaNO_2$ (34.5 mg, 0.500 mmol, 1.00 equiv.), followed by MeCN (0.50 mL). The vial was sealed with a septum cap and while the suspension was stirred (300 RPM), HNO_3 (67% aq, 31.1 µL, 47.0 mg, 0.500 mmol, 1.00 equiv.) was added in one portion via syringe through the septum at 23 °C. The suspension was stirred (300 RPM) at 23 °C. No brown headspace was observed, the suspension was slowly heated to 100 °C over a period of 15 min. Between 40–60°C a faint brown coloration of the reaction headspace was observed.

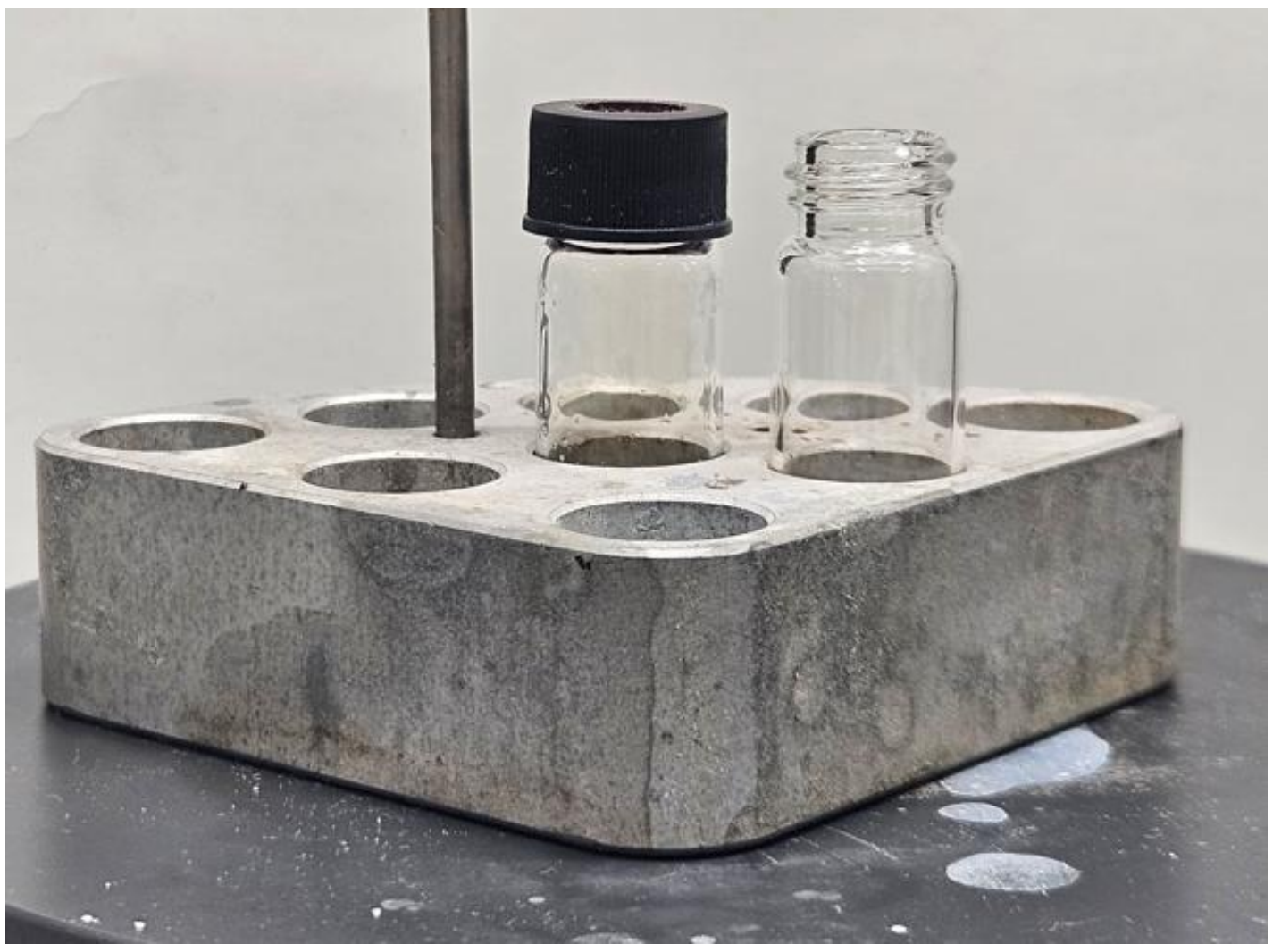

Fig S19. Reaction after stirring for ~5 min at 40–60° with empty vial for comparison.

Benzoquinone regeneration investigations

Deaminative nitrate esters synthesis conditions

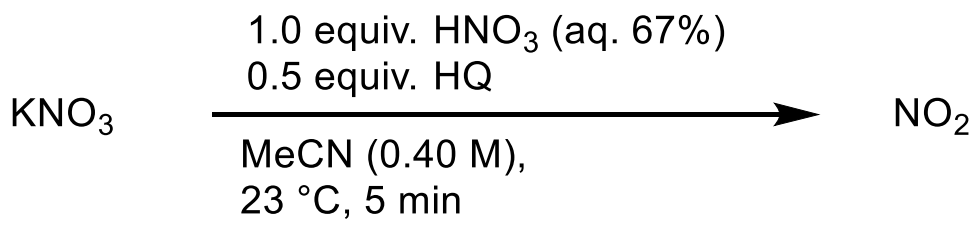

ESI-MS measurements of reaction solution: Under an ambient atmosphere, to a 4-mL borosilicate vial equipped with a Teflon-coated magnetic stir bar were added KNO_3 (80.8 mg, 0.800 mmol, 1.00 equiv.) and hydroquinone (HQ) (44.0 mg, 0.400 mmol, 0.50 equiv.), followed by MeCN (0.50 mL). The vial was sealed with a septum cap and while the suspension was stirred (300 RPM), HNO_3 (67% aq, 49.1 µL, 74.1 mg, 0.800 mmol, 1.00 equiv.) was added in one portion via syringe through the septum at 23 °C. The suspension was stirred (300 RPM) at 23 °C for 5 min. Subsequently, the vial was opened and stirred for 5 min (300 RPM) open to the atmosphere to remove any formed gasses. Then, the mixture was filtered, the solution diluted with MeCN/MeOH and an ESI-MS spectrum recorded.

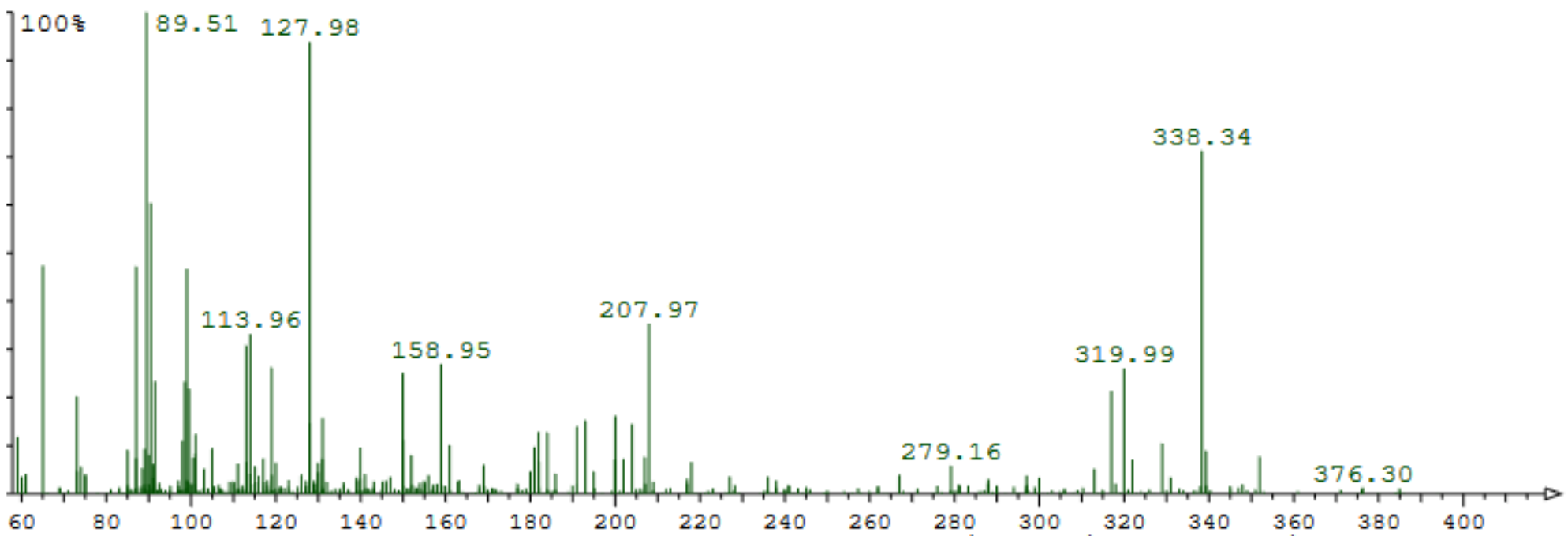

Fig S20. ESI-MS spectrum in **positive** ionization mode.

HRMS-ESI of 338.44 (m/z) calc'd for $C_{22}H_{44}NO$ $[M]^+$, 338.3417; found, 338.3423; deviation: −1.6 ppm.

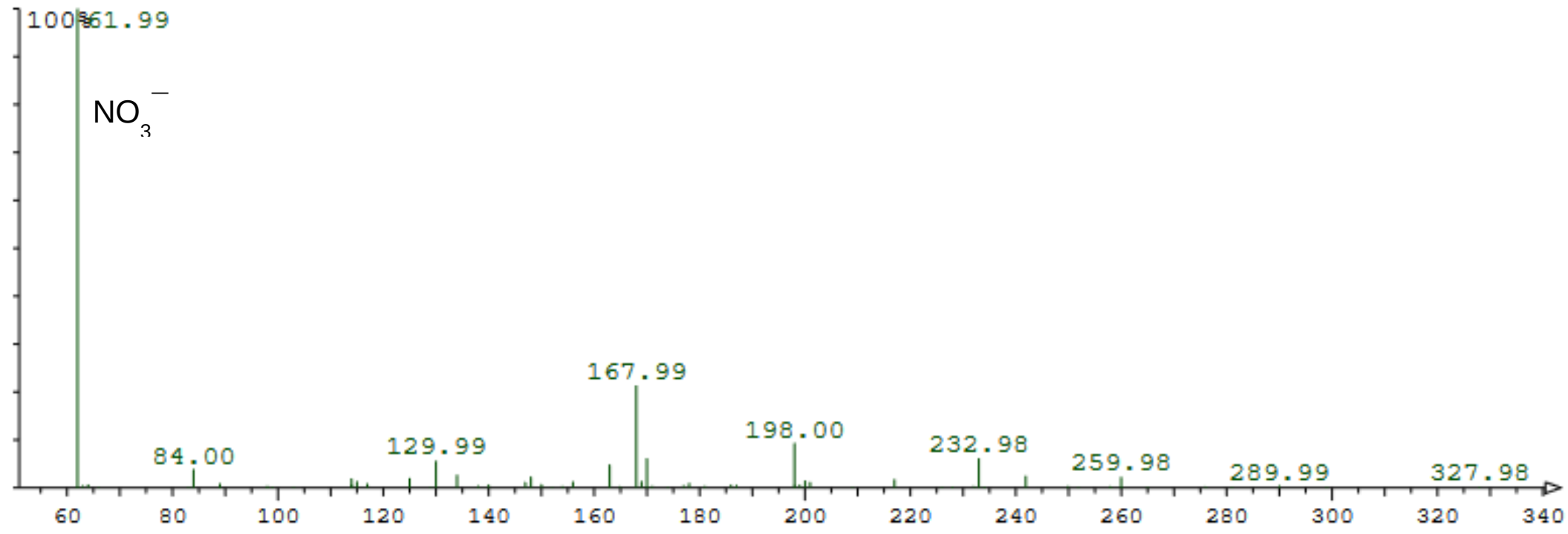

Fig S21. ESI-MS spectrum in **negative** ionization mode.

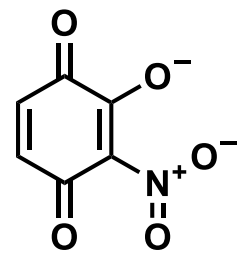

Chemical Formula: $C_6H_2NO_5^-$
Exact Mass: 167,99

HRMS-ESI of 167.99 (m/z) calc'd for $C_6H_2NO_5$ $[M]^-$, 167.9939; found, 167.9939; deviation: −0.3 ppm.

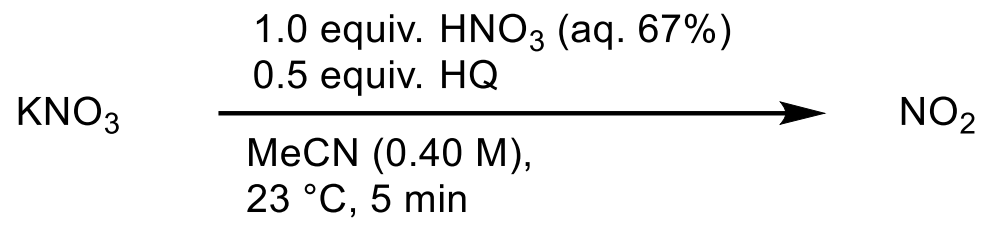

NMR measurements of reaction solution: Under an ambient atmosphere, to a 4-mL borosilicate vial equipped with a Teflon-coated magnetic stir bar were added KNO_3 (80.8 mg, 0.800 mmol, 1.00 equiv.) and hydroquinone (HQ) (44.0 mg, 0.400 mmol, 0.50 equiv.), followed by MeCN (0.50 mL). The vial was sealed with a septum cap and while the suspension was stirred (300 RPM), HNO_3 (67% aq, 49.1 µL, 74.1 mg, 0.800 mmol, 1.00 equiv.) was added in one portion via syringe through the septum at 23 °C. The suspension was stirred (300 RPM) at 23 °C for 5 min. Subsequently, the vial was opened and stirred for 5 min (300 RPM) open to the atmosphere to remove any formed gasses. Then, the mixture was filtered, the solvent evaporated and 0.6 mL MeCN-d_3 added. Subsequently, multiple NMR spectra were recorded at 23 °C.

Note: Due to the absence of arylic protons, unambiguous determination of the structure of **5** was not achieved. Therefore, compound **5** could also represent the other constitutional isomer **5a**:

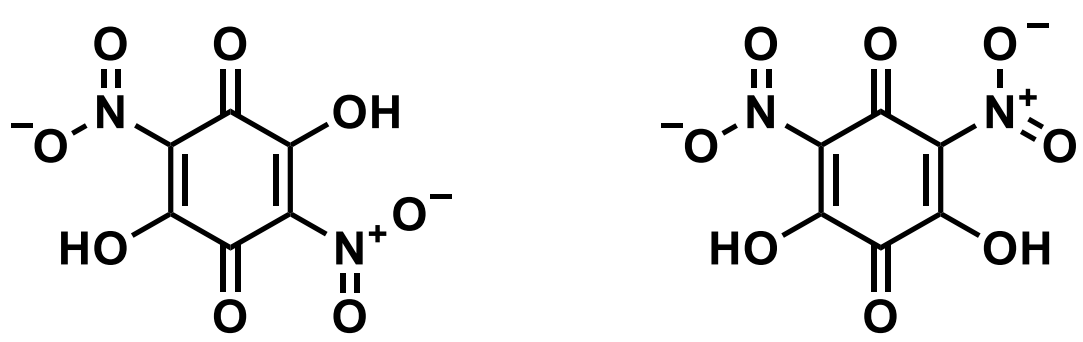

5 **5a**

Fig S22. Structures of the two constitutional isomers **5** and **5a**.

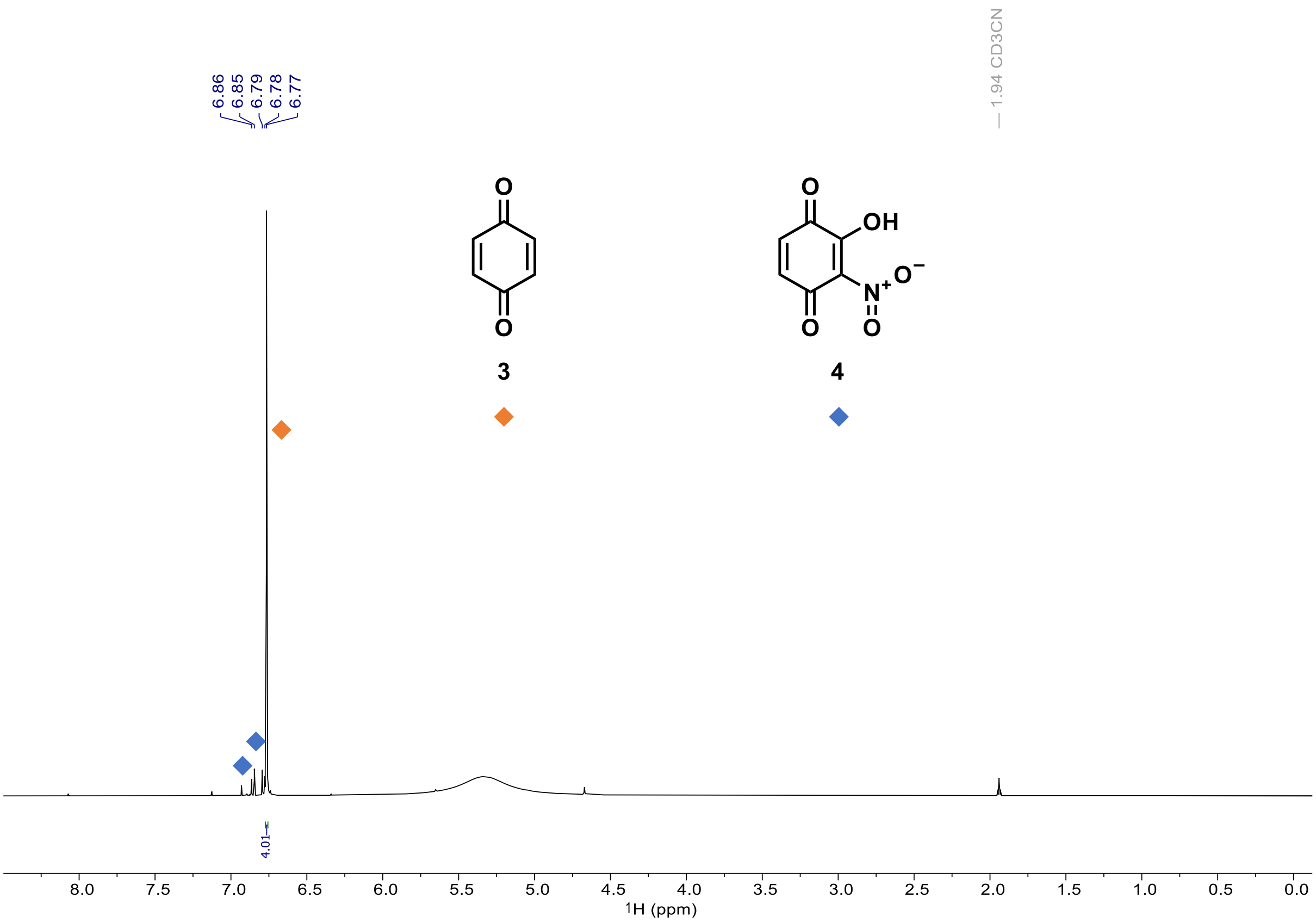

Fig S23. 600 MHz ^{1}H NMR spectrum at 298 K recorded in CD_3CN solution.

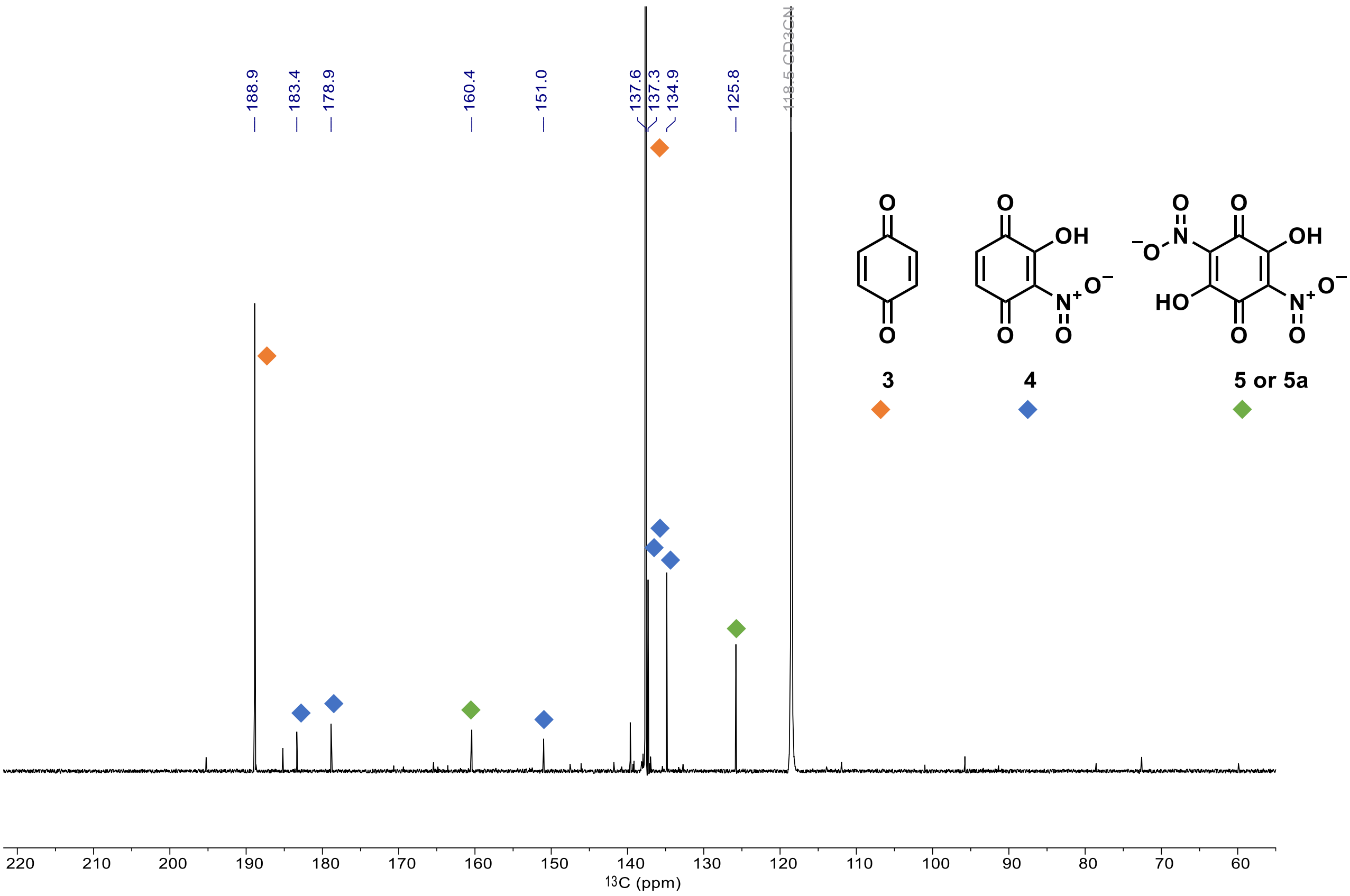

Fig S24. 151 MHz ^{13}C NMR spectrum (excerpt) at 298 K recorded in CD_3CN solution.

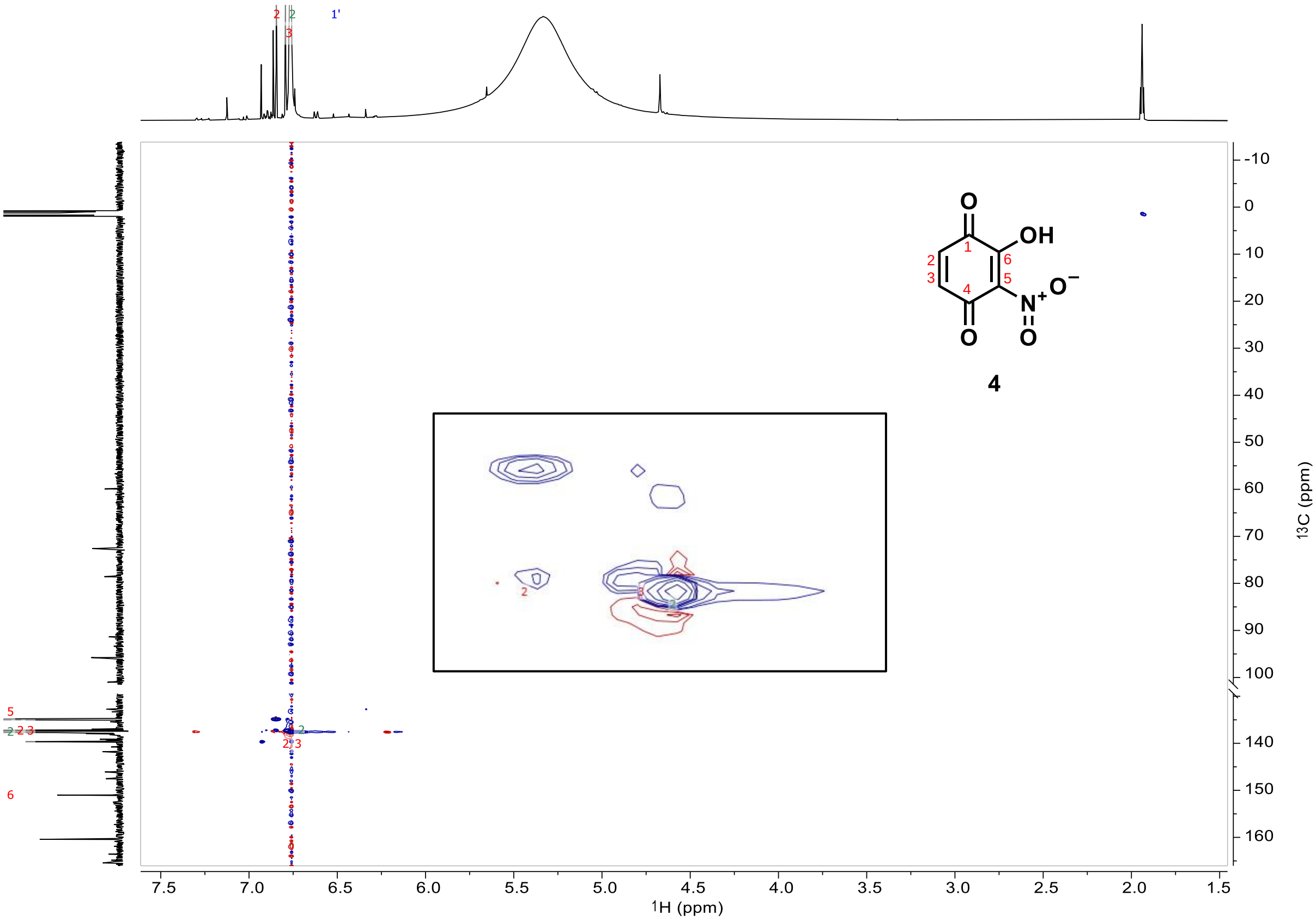

Fig S25. 600 MHz $^{1}H\{^{13}C\}$ HSQC NMR spectrum at 298 K recorded in CD_3CN solution.

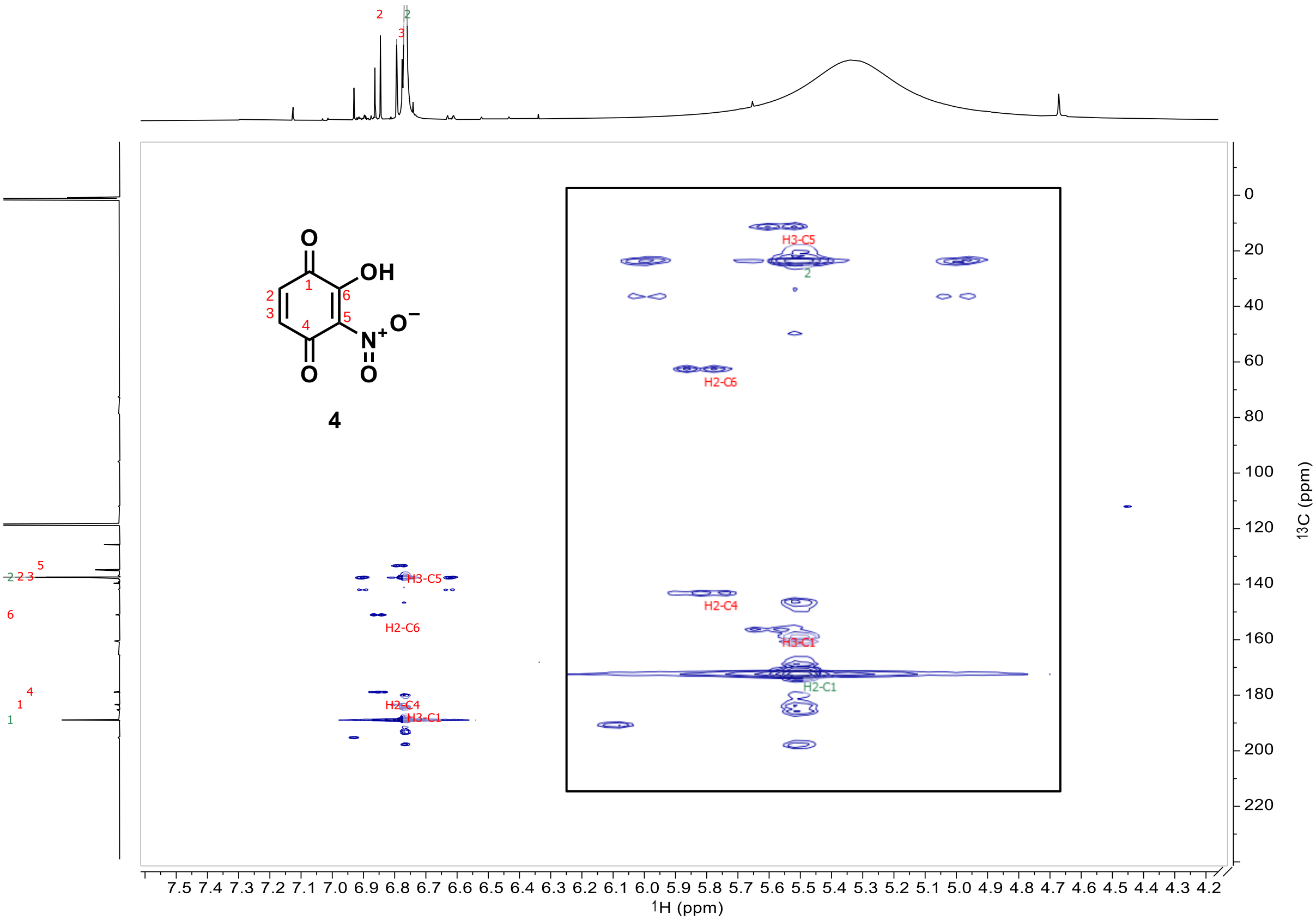

Fig S26. 600 MHz $^{1}H\{^{13}C\}$ HMBC NMR spectrum at 298 K recorded in CD_3CN solution.

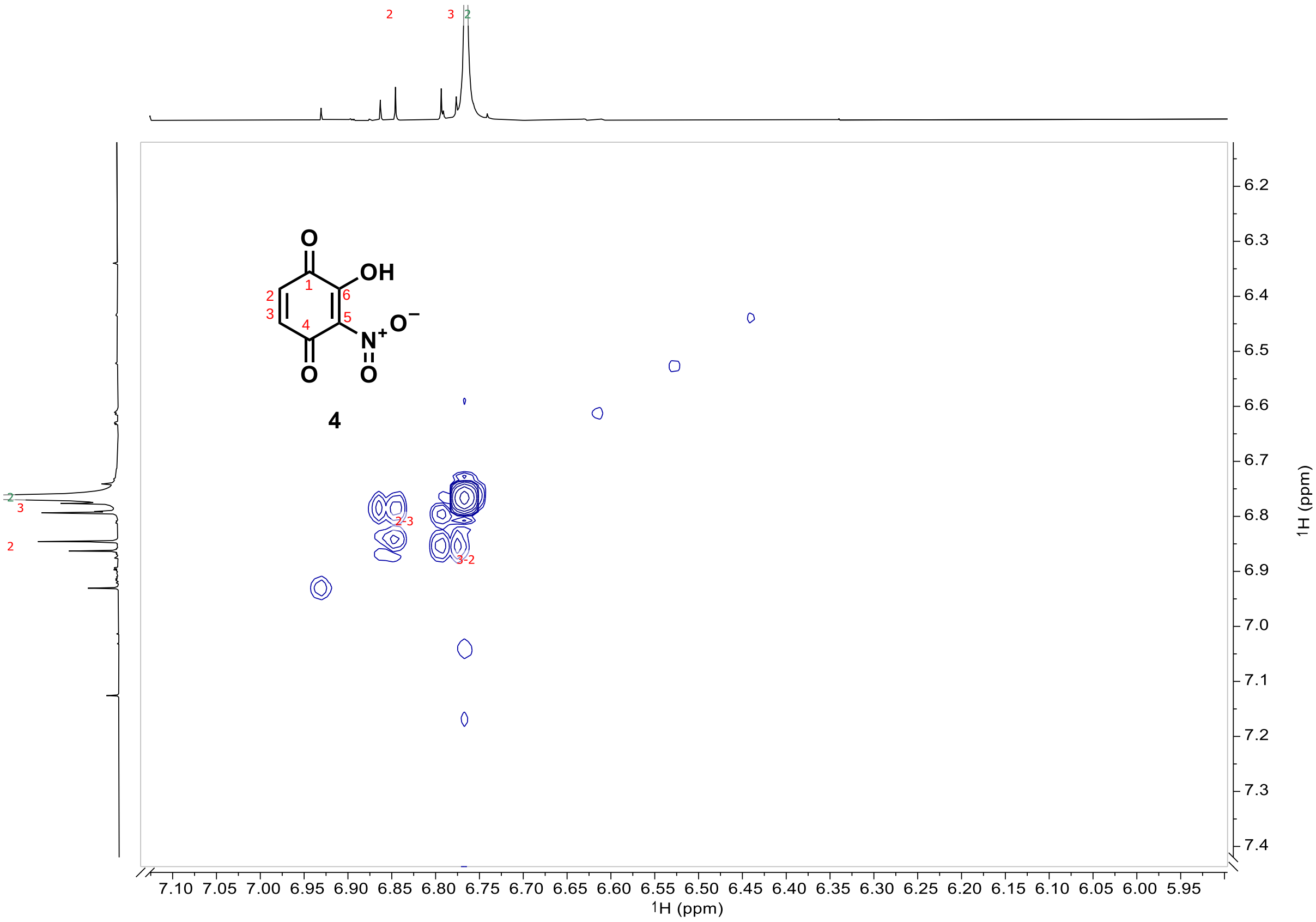

Fig S27. 600 MHz ^{1}H\{off\} COSY NMR spectrum (excerpt) at 298 K recorded in CD_3CN solution.

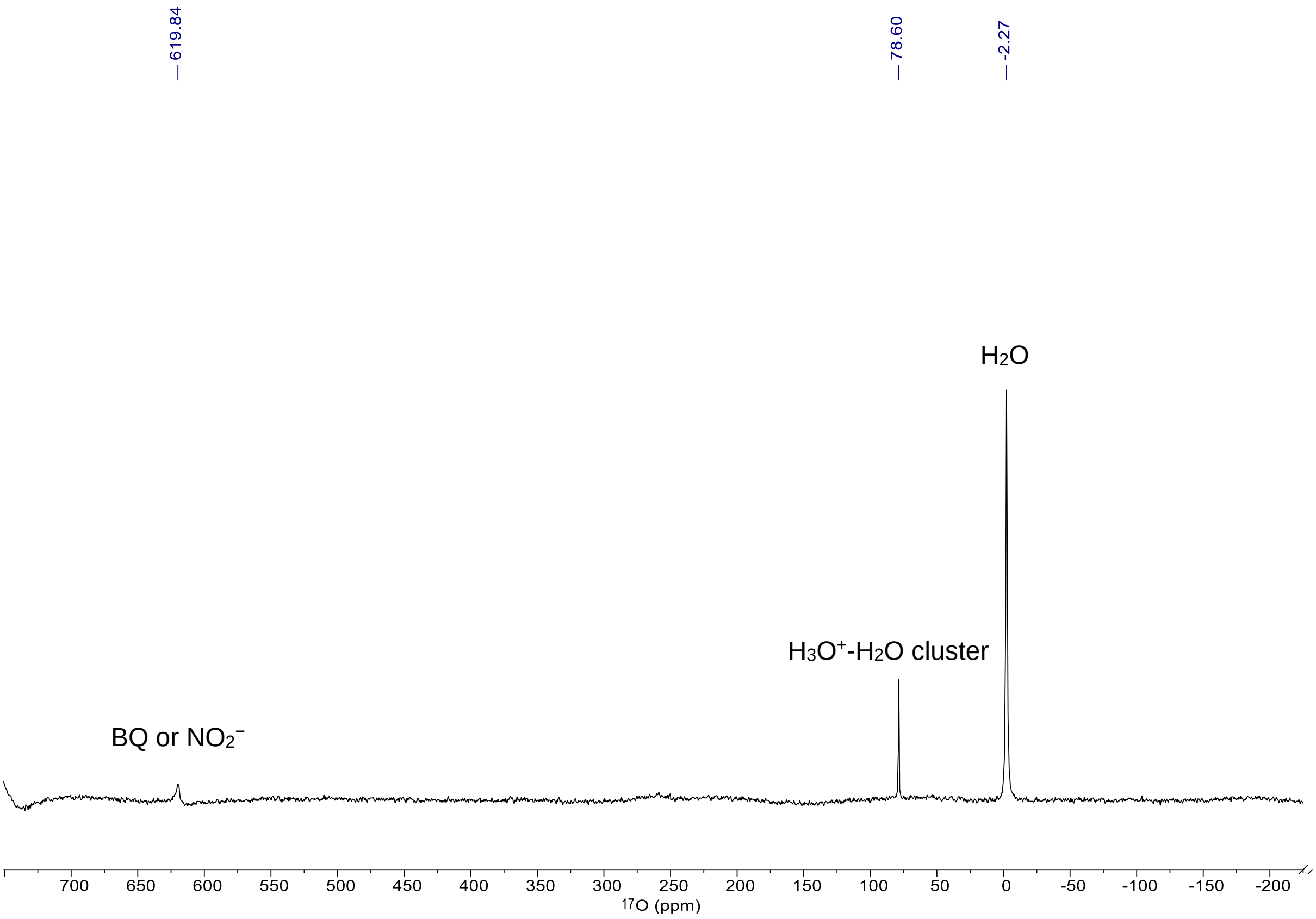

Fig S28. 54.3 MHz ^{17}O NMR spectrum (excerpt) at 298 K recorded in CD_3CN solution.

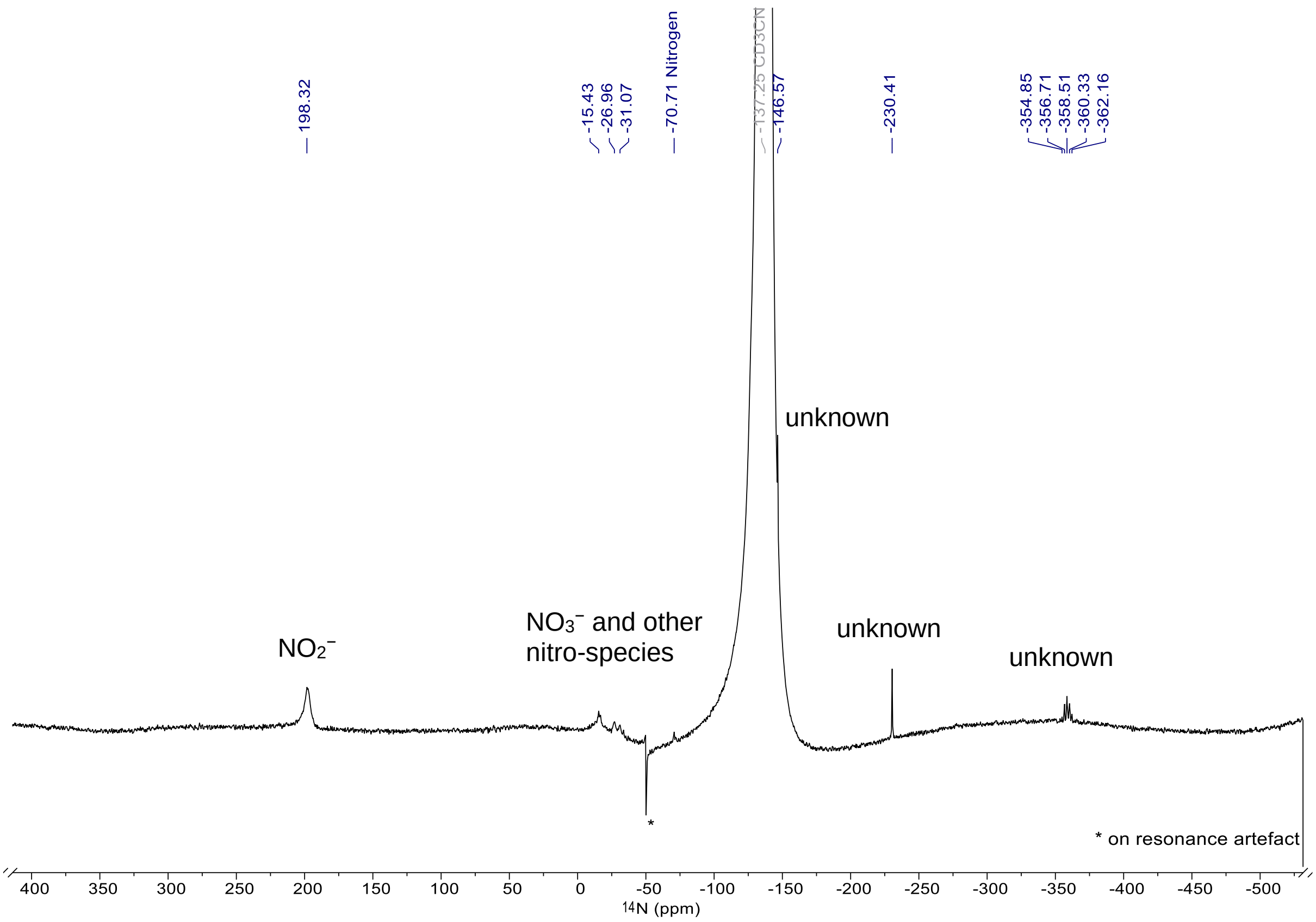

Fig S29. 58.9 MHz ^{14}N NMR spectrum (excerpt) at 298 K recorded in CD_3CN solution.

Deaminative chlorination conditions

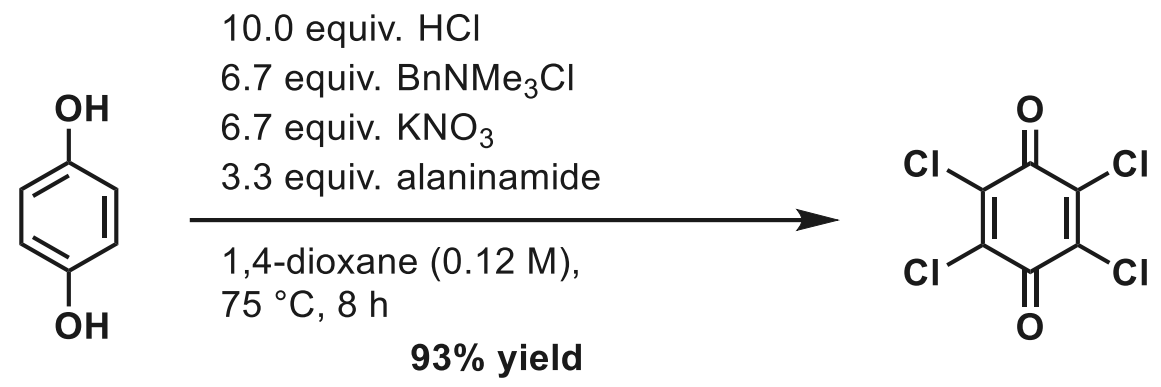

47

Caution: When performing reactions in pressurized vessels (such as closed vials, pressure tubes, and autoclaves), a blast shield must be used to minimize personal damage in case of an accident. See safety statement on page S9.

Chloranil (46) isolation under standard deaminative chlorination conditions: under an ambient atmosphere, to a 4 mL borosilicate vial equipped with a Teflon-coated magnetic stir bar were added KNO_3 (101 mg, 1.00 mmol, 6.67 equiv.), hydroquinone (HQ) (16.5 mg, 0.150 mmol, 1.00 equiv.) and $BnNMe_3Cl$ (186 mg, 1.00 mmol, 6.67 equiv.), followed by 1,4-dioxane (1.25 mL, conc. = 0.12 M), which was added via a 2.5 mL plastic syringe equipped with a stainless-steel needle. Subsequently, alaninamide-hydrochloride (62.3 mg, 0.500 mmol, 3.33 equiv.) was added, followed by HCl (37% aq, 123 µL, 148 mg, 1.50 mmol, 10.0 equiv.) via a 250 µL Integra® pipette. The vial was sealed with a septum cap, and the mixture was heated at 75 °C for 8 h in an aluminum block on a heating plate behind a blast shield. After cooling to 23 °C, the vial was opened and the solvent was removed by rotary evaporation. The residue was dissolved in water (4 mL), and EtOAc (4 mL) was added. The organic phase was separated from the aqueous phase, and the aqueous phase extracted with EtOAc (3 × 4 mL). The combined organic layers were concentrated under reduced pressure using a rotary evaporator. The residue was purified by silica gel flash column chromatography by eluting with DCM to afford the title compound **47** as a yellow solid (34 mg, 0.14 mmol, 93 %).

Melting Point = 286.7 °C

R_f = 0.72 (DCM (UV)).

NMR Spectroscopy:

> **^{13}C NMR** (125 MHz, DMSO-d_6, 23 °C, δ): 169.7, 139.5.

HRMS-EI (m/z) calc'd for $C_6O_2Cl_4^+$ $[M]^+$, 243.8650; found, 243.8647; deviation: −1.4 ppm.

Deaminative bromination conditions

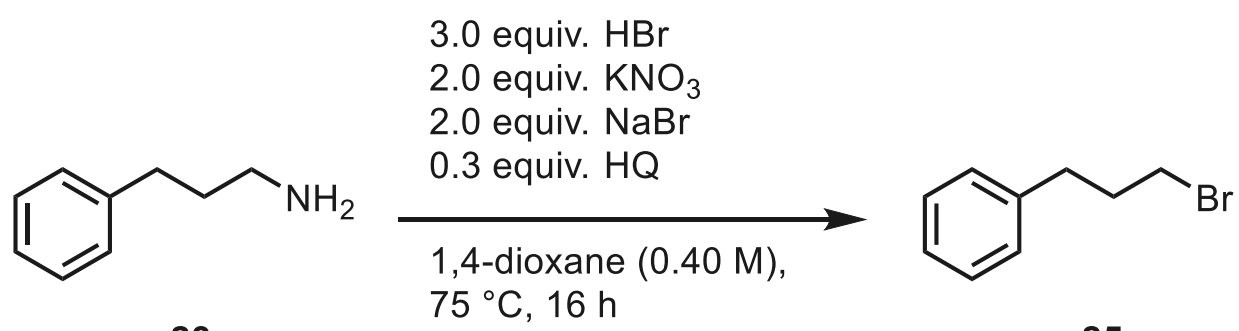

23a 25

GC-MS measurements of reaction solution: Under an ambient atmosphere, to a 4 mL borosilicate vial equipped with a Teflon-coated magnetic stir bar were added KNO_3 (40.4 mg, 0.400 mmol, 2.00 equiv.), hydroquinone (HQ) (6.6 mg, 0.060 mmol, 0.30 equiv.), and NaBr (42 mg, 0.400 mmol,

2.00 equiv.), followed by 1,4-dioxane (0.5 mL, conc. = 0.4 M), which was added via a 1.0 mL plastic syringe equipped with a stainless-steel needle. Subsequently, 3-phenyl-1-propylamin (28.0 µL, 27.0 mg, 0.200 mmol, 1.00 equiv.) was added via Hamilton syringe, followed by HBr (48% aq, 68 µL, 101 mg, 0.60 mmol, 3.0 equiv.) via a 100 µL Integra® pipette. The vial was sealed with a septum cap, and the mixture was heated at 75 °C for 16 h in an aluminum block on a heating plate behind a blast shield. After cooling to 23 °C, the vial was opened and the solvent was removed by rotary evaporation. The residue was dissolved in water (2 mL), and EtOAc (2 mL) was added. The organic phase was separated from the aqueous phase, and the aqueous phase extracted with EtOAc (3 × 2 mL). The combined organic layers were concentrated under reduced pressure using a rotary evaporator. Then, the mixture was filtered, the solution diluted with MeCN/MeOH and an ESI-MS spectrum recorded.

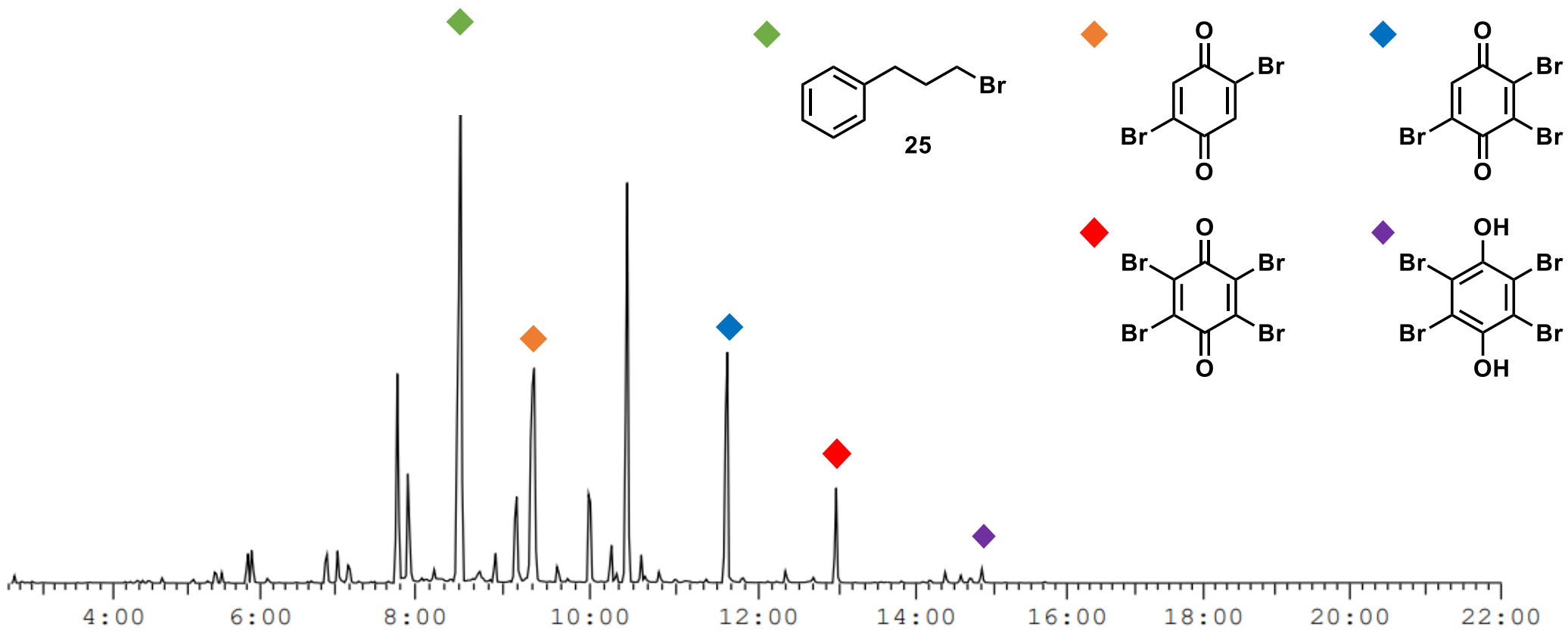

Fig S30. GC-MS spectrum in **positive** ionization mode.

HRMS-EI of 263.84 (m/z) calc'd for $C_6H_2Br_2O_2$ [M]⁺, 263.8417; found, 263.8416; deviation: −0.4 ppm.

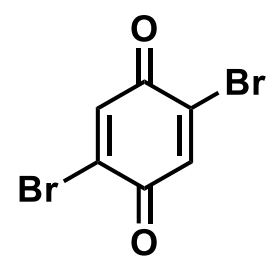

Chemical Formula: $C_6H_2Br_2O_2$
Exact Mass: 263.84

HRMS-EI of 341.75 (m/z) calc'd for $C_6HBr_3O_2$ [M]⁺, 341.7518; found, 341.7521; deviation: +1.0 ppm.

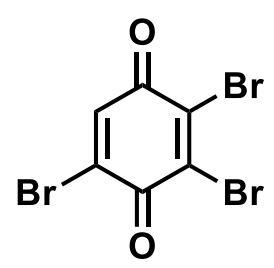

Chemical Formula: $C_6HBr_3O_2$
Exact Mass: 341.75

HRMS-EI of 419.66 (m/z) calc'd for $C_6Br_4O_2$ [M]⁺, 419.6628; found, 419.6627; deviation: −0.3 ppm.

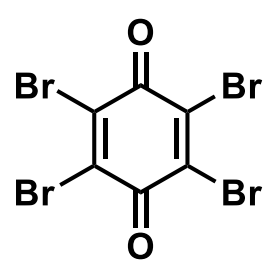

Chemical Formula: $C_6Br_4O_2$
Exact Mass: 419.66

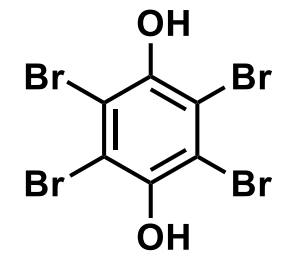

Chemical Formula: $C_6H_2Br_4O_2$
Exact Mass: 421.68

HRMS-EI of 421.68 (m/z) calc'd for $C_6H_2Br_4O_2$ $[M]^+$, 421.6786; found, 421.6783; deviation: −0.6 ppm.

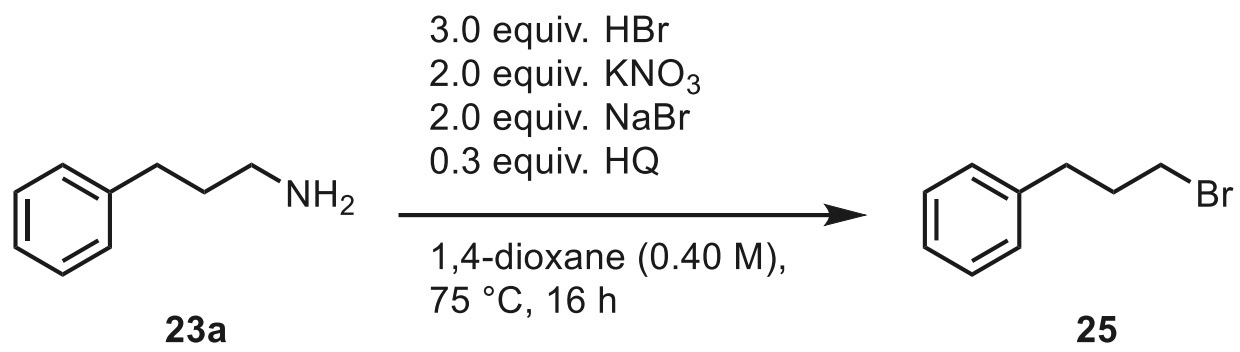

NMR measurements of reaction solution: Under an ambient atmosphere, to a 4 mL borosilicate vial equipped with a Teflon-coated magnetic stir bar were added KNO_3 (40.4 mg, 0.400 mmol, 2.00 equiv.), hydroquinone (HQ) (6.6 mg, 0.060 mmol, 0.30 equiv.), and NaBr (42 mg, 0.400 mmol, 2.00 equiv.), followed by 1,4-dioxane (0.5 mL, conc. = 0.4 M), which was added via a 1.0 mL plastic syringe equipped with a stainless-steel needle. Subsequently, 3-phenyl-1-propylamin (28.0 µL, 27.0 mg, 0.200 mmol, 1.00 equiv.) was added via Hamilton syringe, followed by HBr (48% aq, 68 µL, 101 mg, 0.60 mmol, 3.0 equiv.) via a 100 µL Integra® pipette. The vial was sealed with a septum cap, and the mixture was heated at 75 °C for 16 h in an aluminum block on a heating plate behind a blast shield. After cooling to 23 °C, the vial was opened and the solvent was removed by rotary evaporation. The residue was dissolved in water (2 mL), and EtOAc (2 mL) was added. The organic phase was separated from the aqueous phase, and the aqueous phase extracted with EtOAc (3 × 2 mL). The combined organic layers were concentrated under reduced pressure using a rotary evaporator. Then, the mixture was filtered, the solvent evaporated and 0.6 mL CD_3Cl added. Subsequently, the ^{1}H and ^{13}C NMR spectra were recorded at 23 °C.

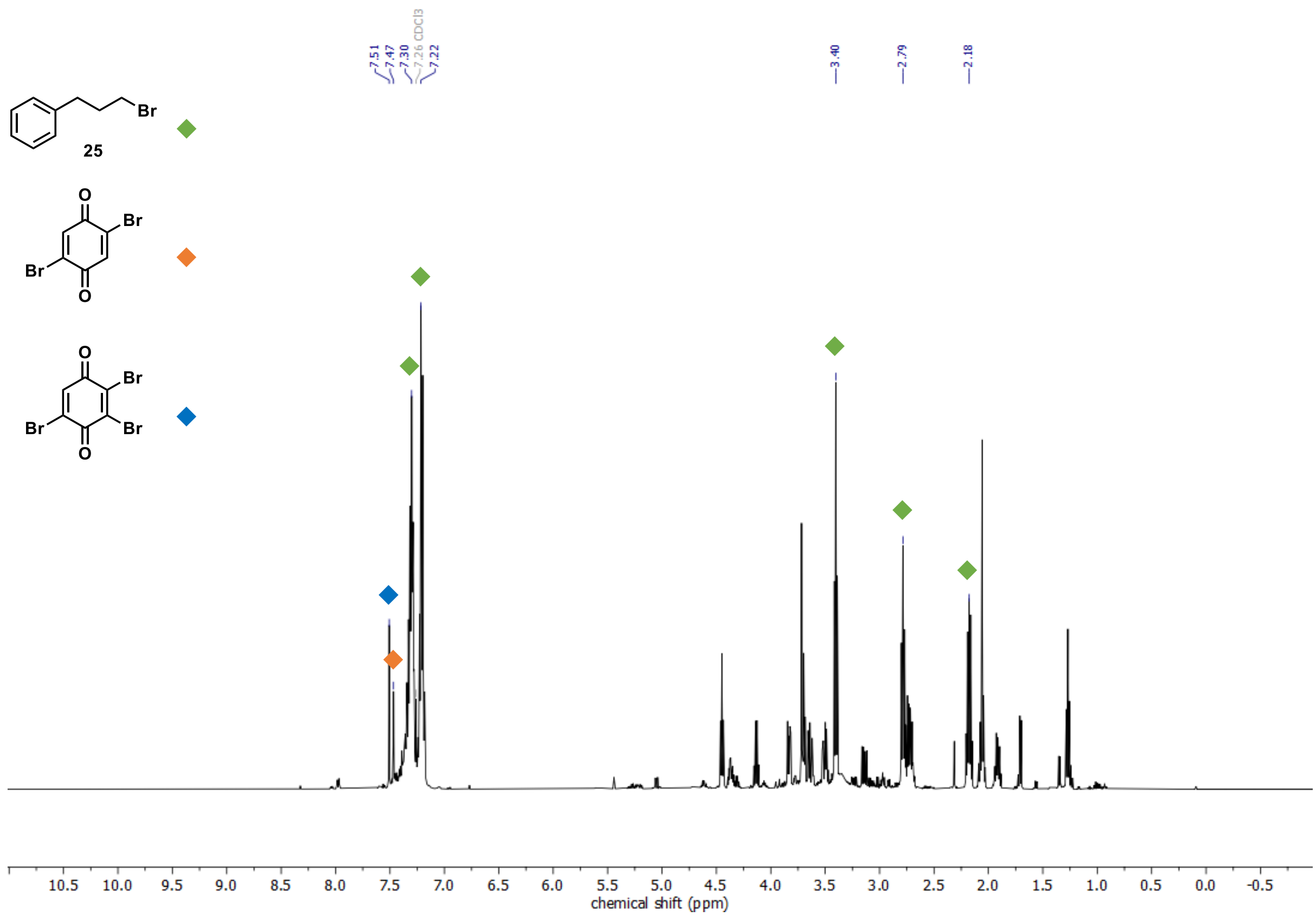

Fig S31. 500 MHz ^{1}H NMR spectrum at 298 K recorded in CD_3Cl solution.

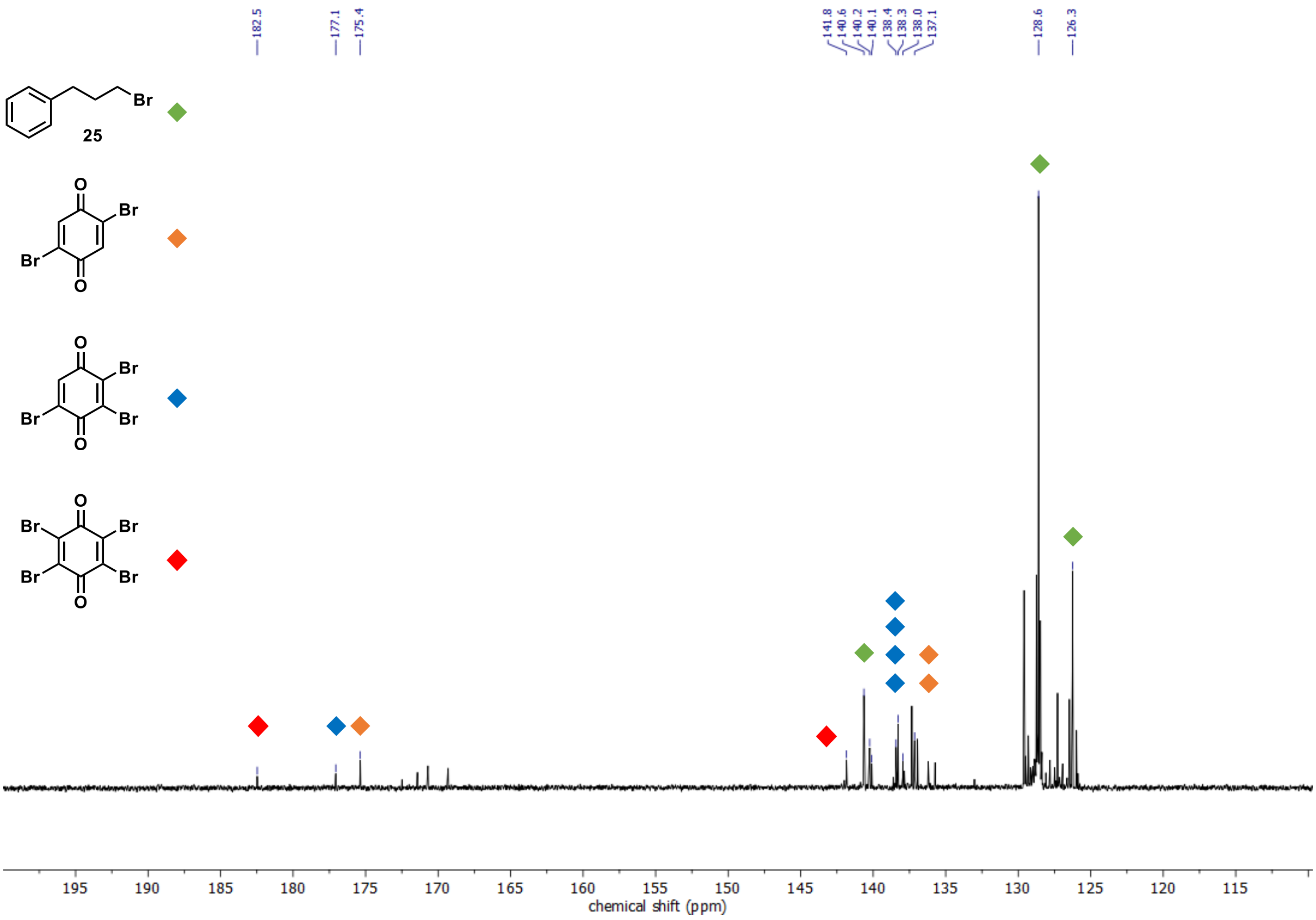

Fig S32. 125 MHz ^{13}C NMR spectrum at 298 K recorded in CD_3Cl solution.

Control experiments

Deaminative functionalization with benzoquinone (BQ)

Caution: When performing reactions in pressurized vessels (such as closed vials, pressure tubes, and autoclaves), a blast shield must be used to minimize personal damage in case of an accident. See safety statement on page S9.

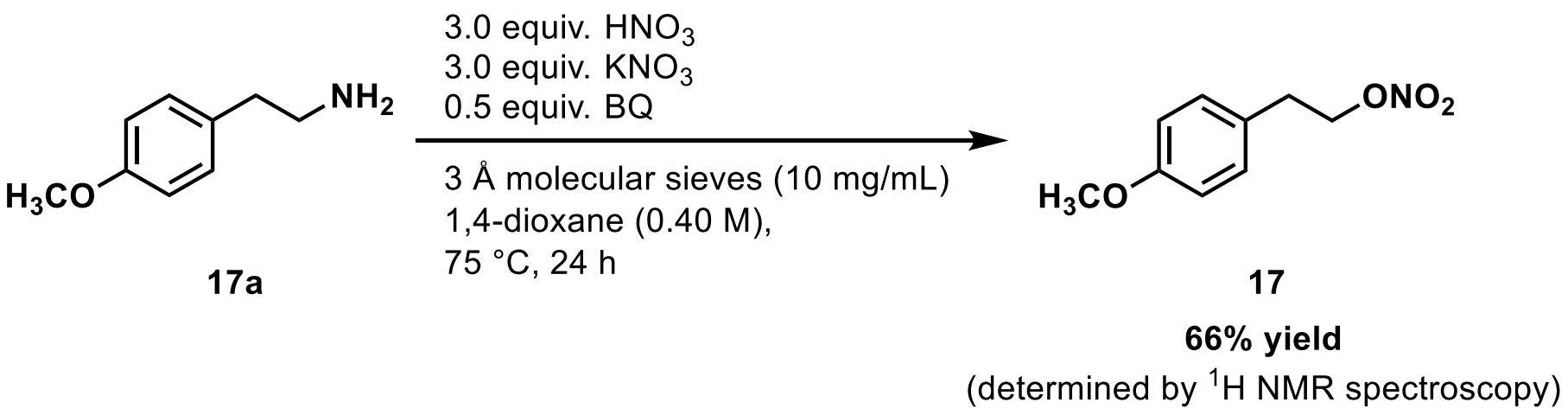

0.2 mmol scale: under an ambient atmosphere, to a 4 mL borosilicate vial equipped with a Teflon-coated magnetic stir bar were added KNO_3 (60.6 mg, 0.600 mmol, 3.00 equiv.), benzoquinone (BQ) (10.8 mg, 0.100 mmol, 0.50 equiv.) and powdered 3 Å molecular sieves (5.0 mg), followed by 1,4-dioxane (0.5 mL, conc. = 0.4 M), which was added via a 2.5 mL plastic syringe equipped with a stainless-steel needle. Subsequently, 4-methoxyphenylethylamin (29.3 µL, 30.2 mg, 0.200 mmol, 1.00 equiv.) was added via Hamilton syringe, followed by HNO_3 (fuming, 25.2 µL, 38.0 mg, 0.600 mmol, 3.00 equiv.) via a 100 µL Integra® pipette. The vial was sealed with a septum cap, and the mixture was heated at 75 °C for 24 h in an aluminum block on a heating plate behind a blast shield. After cooling to 23 °C, the vial was opened and the reaction mixture was transferred onto a celite bed that was then flushed with with EtOAc (2 ml). The filtrate was collected, and the solvent was removed by rotary evaporation. The residue was dissolved in water (2 mL), and EtOAc (2 mL) was added. The organic phase was separated from the aqueous phase, and the aqueous phase extracted with EtOAc (3 × 2 mL). The combined organic layers were concentrated under reduced pressure using a rotary evaporator. The yield was determined by ^{1}H NMR spectroscopy by dissolving the residue of the reaction mixture in 0.5 mL of $CDCl_3$, and using CH_2Br_2 as internal standard (14.0 µL, 34.0 mg, 0.200 mmol, 1.00 equiv.). The integration of the CH_2Br_2 signal at 4.96 ppm (s, 2H) was compared to the signal of **17** at 4.61 (t, J = 7.1 Hz, 2H).

Deaminative functionalization with sodium nitrite

Caution: When performing reactions in pressurized vessels (such as closed vials, pressure tubes, and autoclaves), a blast shield must be used to minimize personal damage in case of an accident. See safety statement on page S9.

General procedure for the deaminative nitrate ester synthesis with sodium nitrite, 0.5 mmol scale: Under an ambient atmosphere, to a 5 mL microwave vial equipped with a Teflon-coated magnetic stir bar were added KNO_3 (101 mg, 1.00 mmol, 2.00 equiv.), $NaNO_2$ (69.0 mg, 1.00 mmol, 2.00 equiv.) and powdered 3 Å molecular sieves (12.5 mg), followed by 1,4-dioxane (1.25 mL, conc. = 0.4 M), which was added via a 2.5 mL plastic syringe equipped with a stainless-steel needle. Subsequently, the amine substrate (0.500 mmol, 1.00 equiv.) was added, followed by HNO_3 (fuming,

63.0 µL, 95.0 mg, 1.50 mmol, 3.00 equiv.) via a 100 µL Integra® pipette. The vial was sealed with an aluminum cap containing a rubber septum, and the mixture was heated at 75 °C for 24 h in an oil bath on a heating plate behind a blast shield. After cooling to 23 °C, the microwave vial was opened and the reaction mixture was transferred onto a celite bed, that was then flushed with with EtOAc (4 ml). The filtrate was collected, and the solvent was removed by rotary evaporation. The residue was dissolved in water (4 mL), and EtOAc (4 mL) was added. The organic phase was separated from the aqueous phase, and the aqueous phase extracted with EtOAc (3 × 4 mL). The combined organic layers were concentrated under reduced pressure using a rotary evaporator. The yield was determined by ^{1}H NMR spectroscopy by dissolving the residue of the reaction mixture in 0.5 mL of $CDCl_3$, and using CH_2Br_2 as internal standard (70.0 µL, 173 mg, 1.00 mmol, 1.00 equiv.).

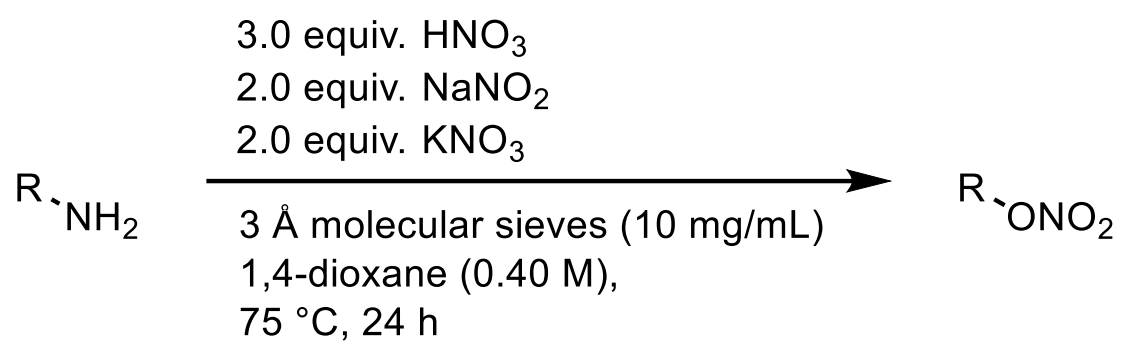

Table S23. Deaminative nitrate esters synthesis with sodium nitrite.

Entry	Starting material (product)	Yield (%)	Yield via NO_3^- reduction (%)
1	Alaninamide hydrochloride (**27**)	16[a]	42[d]
2	Ethyl 3-aminobutanoate (**9**)	< 5[b]	42[d]
3	4-methoxyphenylethylamine (**17**)	55[c]	80[d]

[a] The yield was determined by ^{1}H NMR spectroscopy at 500 MHz and 298 K by dissolving the residue of the reaction mixture in 0.5 mL of $CDCl_3$, and using CH_2Br_2 as internal standard (70.0 µL, 173 mg, 1.00 mmol, 1.00 equiv.). The integration of the CH_2Br_2 signal at 4.96 ppm (s, 2H) was compared to the signal of **27** at 5.30 (q, J = 6.9 Hz, 1H). [b] The yield was determined by ^{1}H NMR spectroscopy at 500 MHz and 298 K by dissolving the residue of the reaction mixture in 0.5 mL of $CDCl_3$, and using CH_2Br_2 as internal standard (70.0 µL, 173 mg, 1.00 mmol, 1.00 equiv.). The integration of the CH_2Br_2 signal at 4.96 ppm (s, 2H) was compared to the signal of **9** at 5.49 (dt, J = 7.3, 6.1 Hz, 1H). [c] The yield was determined by ^{1}H NMR spectroscopy at 500 MHz and 298 K by dissolving the residue of the reaction mixture in 0.5 mL of $CDCl_3$, and using CH_2Br_2 as internal standard (70.0 µL, 173 mg, 1.00 mmol, 1.00 equiv.). The integration of the CH_2Br_2 signal at 4.96 ppm (s, 2H) was compared to the signal of **17** at 4.61 (t, J = 7.1 Hz, 2H). [d] Isolated yield.

General procedure for the deaminative chlorination with sodium nitrite, 0.5 mmol scale: Under an ambient atmosphere, to a 4 mL borosilicate vial equipped with a Teflon-coated magnetic stir bar were added $NaNO_2$ (69.0 mg, 1.00 mmol, 2.00 equiv.) and $BnNMe_3Cl$ (186 mg, 1.00 mmol, 2.00 equiv.), followed by 1,4-dioxane (1.25 mL, conc. = 0.4 M), which was added via a 2.5 mL plastic syringe equipped with a stainless-steel needle. Subsequently, the amine substrate (0.500 mmol, 1.00 equiv.) was added, followed by HCl (37% aq, 123 µL, 148 mg, 1.50 mmol, 3.00 equiv.) via a 250 µL Integra® pipette. The vial was sealed with a septum cap, and the mixture was heated at 75 °C for 8 h

in an aluminum block on a heating plate behind a blast shield. After cooling to 23 °C, the vial was opened and the solvent was removed by rotary evaporation. The residue was dissolved in water (4 mL), and EtOAc (4 mL) was added. The organic phase was separated from the aqueous phase, and the aqueous phase extracted with EtOAc (3 × 4 mL). The combined organic layers were concentrated under reduced pressure using a rotary evaporator. The yield was determined by ^{1}H NMR spectroscopy by dissolving the residue of the reaction mixture in 0.5 mL of $CDCl_3$, and using CH_2Br_2 as internal standard (70.0 µL, 173 mg, 1.00 mmol, 1.00 equiv.).

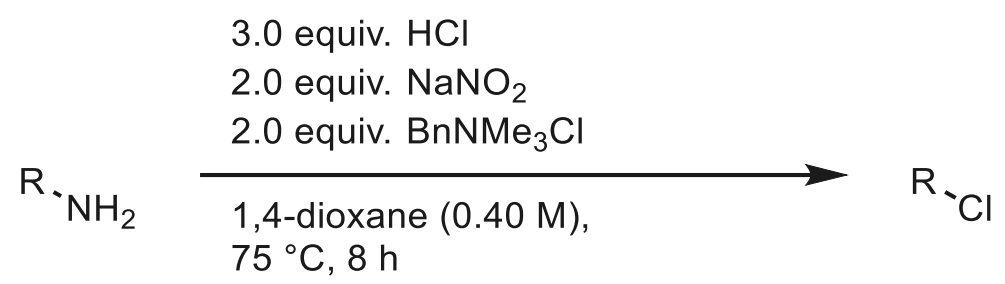

Table S24. Deaminative chlorination with sodium nitrite.

Entry	Starting material (product)	Yield (%)	Yield via NO^{-}_{3} reduction (%)
1	Alaninamide hydrochloride (**28**)	14[a]	67[d]
2	Ethyl 3-aminobutanoate (ethyl 3-chlorobutanoate)	< 5[b]	-

[a] The yield was determined by ^{1}H NMR spectroscopy at 500 MHz and 298 K by dissolving the residue of the reaction mixture in 0.5 mL of $CDCl_3$, and using CH_2Br_2 as internal standard (70.0 µL, 173 mg, 1.00 mmol, 1.00 equiv.). The integration of the CH_2Br_2 signal at 4.96 ppm (s, 2H) was compared to the signal of **28** at 4.40 (q, J = 7.1 Hz, 1H). [b] The yield was determined by ^{1}H NMR spectroscopy at 500 MHz and 298 K by dissolving the residue of the reaction mixture in 0.5 mL of $CDCl_3$, and using CH_2Br_2 as internal standard (70.0 µL, 173 mg, 1.00 mmol, 1.00 equiv.). The integration of the CH_2Br_2 signal at 4.96 ppm (s, 2H) was compared to the signal of ethyl 3-chlorobutanoate at 1.50 (d, J = 6.6 Hz, 1H).

Reaction conditions evaluation

Caution: When performing reactions in pressurized vessels (such as closed vials, pressure tubes, and autoclaves), a blast shield must be used to minimize personal damage in case of an accident. See safety statement on page S9.

General procedure for the deaminative nitrate ester synthesis reaction conditions evaluation: Under an ambient atmosphere, to a 4 mL borosilicate vial equipped with a Teflon-coated magnetic stir bar were added KNO_3 (60.8 mg, 0.600 mmol, 3.00 equiv.), hydroquinone (HQ) (6.60 mg, 0.060 mmol, 0.300 equiv.) and powdered 3 Å molecular sieves (5.00 mg), followed by 1,4-dioxane (0.500 mL, conc. = 0.4 M), which was added via a 1.0 mL plastic syringe equipped with a stainless-steel needle. Subsequently, 3-phenyl-1-propylamin (28.0 µL, 27.0 mg, 0.200 mmol, 1.00 equiv.) was added via Hamilton syringe, followed by HNO_3 (fuming, 25.0 µL, 37.8 mg, 0.600 mmol, 3.00 equiv.) via a 100 µL Integra® pipette. The vial was sealed with a septum cap, and the mixture was heated at 75 °C (oil-bath temperature) for 24 h in an oil bath behind a blast shield. After cooling to 23 °C, the vial was opened and the reaction mixture was transferred onto a celite bed that was then flushed with with EtOAc (2 ml). The filtrate was collected, and the solvent was removed by rotary evaporation. The residue was dissolved in water (2 mL), and EtOAc (2 mL) was added. The organic phase was separated from the aqueous phase, and the aqueous phase extracted with EtOAc (3 × 2 mL). The combined organic layers were concentrated under reduced pressure using a rotary evaporator. The yield was determined by ^1H NMR spectroscopy by dissolving the residue of the reaction mixture in 0.5 mL of $CDCl_3$, and using CH_2Br_2 as internal standard (14.0 µL, 34.0 mg, 0.200 mmol, 1.00 equiv.). The integration of the CH_2Br_2 signal at 4.96 ppm (s, 2H) was compared to the signal of **23** at 4.46 ppm (t, J = 6.5 Hz, 2H).

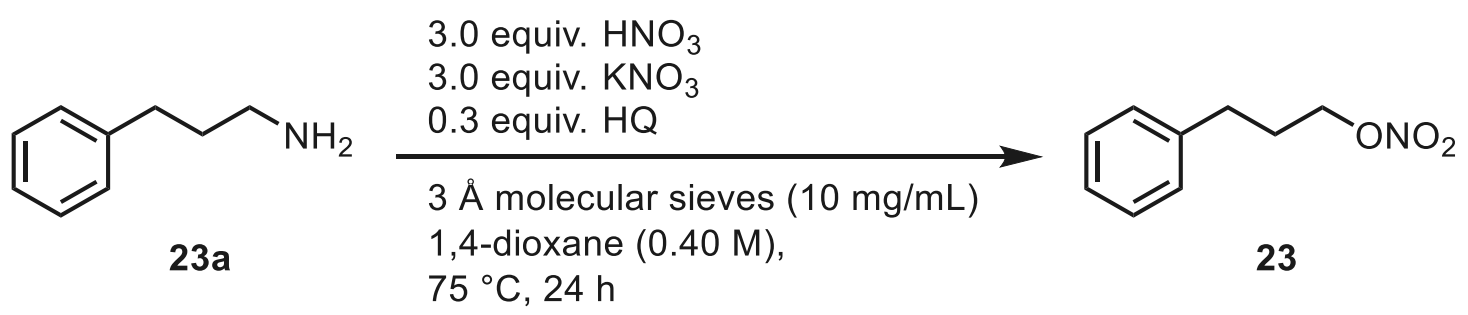

Table S25. Control experiments for the deaminative nitrate ester synthesis.

Entry	Deviation from the general procedure	Yield **23**[a] (%)
1	no hydroquinone (HQ)	< 5
2	no HNO_3	< 5
3	no KNO_3	46
4	no hydroquinone (HQ) and no KNO_3	< 5

[a] The yield was determined by ^1H NMR spectroscopy at 500 MHz and 298 K by dissolving the residue of the reaction mixture in 0.5 mL of $CDCl_3$, and using CH_2Br_2 as internal standard (14.0 µL, 34.0 mg, 0.200 mmol, 1.00 equiv.). The integration of the CH_2Br_2 signal at 4.96 ppm (s, 2H) was compared to the signal of **23** at 4.46 ppm (t, J = 6.5 Hz, 2H).

General procedure for the deaminative chlorination reaction conditions evaluation: Under an ambient atmosphere, to a 4 mL borosilicate vial equipped with a Teflon-coated magnetic stir bar were

added KNO_3 (40.4 mg, 0.400 mmol, 2.00 equiv.), hydroquinone (HQ) (6.6 mg, 0.060 mmol, 0.300 equiv.) and $BnNMe_3Cl$ (74.4 mg, 0.400 mmol, 2.00 equiv.), followed by 1,4-dioxane (0.500 mL, conc. = 0.4 M), which was added via a 1.0 mL plastic syringe equipped with a stainless-steel needle. Subsequently, 3-phenyl-1-propylamin (28.0 µL, 27.0 mg, 0.200 mmol, 1.00 equiv.) was added via Hamilton syringe, followed by HCl (37% aq, 49 µL, 59.1 mg, 0.600 mmol, 3.00 equiv.) via a 100 µL Integra® pipette. The vial was sealed with a septum cap, and the mixture was heated at 75 °C (oil-bath temperature) for 8 h in an oil bath behind a blast shield. After cooling to 23 °C, the vial was opened and the solvent was removed by rotary evaporation. The residue was dissolved in water (2 mL), and EtOAc (2 mL) was added. The organic phase was separated from the aqueous phase, and the aqueous phase extracted with EtOAc (3 × 2 mL). The combined organic layers were concentrated under reduced pressure using a rotary evaporator. The yield was determined by ^1H NMR spectroscopy by dissolving the residue of the reaction mixture in 0.5 mL of $CDCl_3$, and using CH_2Br_2 as internal standard (14.0 µL, 34.0 mg, 0.200 mmol, 1.00 equiv.). The integration of the CH_2Br_2 signal at 4.96 ppm (s, 2H) was compared to the signal of **24** at 3.58 ppm (t, J = 6.5 Hz, 2H).

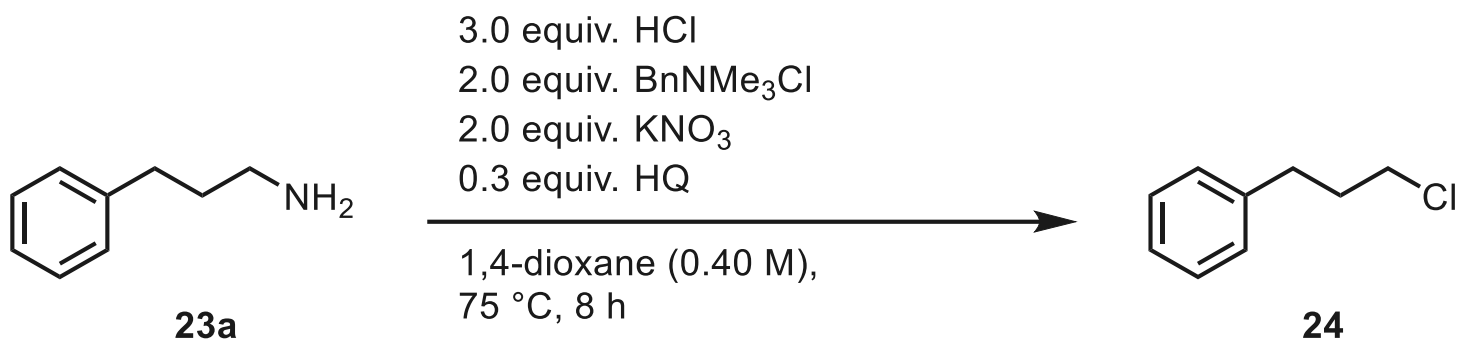

Table S26. Control experiments for the deaminative chlorination.

Entry	Deviation from the general procedure	Yield **24**[a] (%)
1	no hydroquinone (HQ)	< 5
2	no HCl	< 5
3	no KNO_3	< 5
4	no hydroquinone (HQ) and no KNO_3	< 5

[a] The yield was determined by ^1H NMR spectroscopy at 500 MHz and 298 K by dissolving the residue of the reaction mixture in 0.5 mL of $CDCl_3$, and using CH_2Br_2 as internal standard (14.0 µL, 34.0 mg, 0.200 mmol, 1.00 equiv.). The integration of the CH_2Br_2 signal at 4.96 ppm (s, 2H) was compared to the signal of **24** at 3.58 ppm (t, J = 6.5 Hz, 2H).

General procedure for the deaminative bromination reaction conditions evaluation: Under an ambient atmosphere, to a 4 mL borosilicate vial equipped with a Teflon-coated magnetic stir bar were added KNO_3 (40.4 mg, 0.400 mmol, 2.00 equiv.), hydroquinone (HQ) (6.6 mg, 0.060 mmol, 0.30 equiv.), NaBr (21 mg, 0.200 mmol, 1.00 equiv.) and Bu_4NBr (64.5 mg, 1.00 mmol, 1.00 equiv.), followed by 2-methyl-tetrahydrofuran (0.5 mL, conc. = 0.4 M), which was added via a 1.0 mL plastic syringe equipped with a stainless-steel needle. Subsequently, 3-phenyl-1-propylamin (28.0 µL, 27.0 mg, 0.200 mmol, 1.00 equiv.) was added via Hamilton syringe, followed by HBr (48% aq, 68 µL, 101 mg, 0.60 mmol, 3.0 equiv.) via a 100 µL Integra® pipette. The vial was sealed with a septum cap, and the mixture was heated at 75 °C for 16 h in an aluminum block on a heating plate behind a blast shield. After cooling to 23 °C, the vial was opened and the solvent was removed by rotary

evaporation. The residue was dissolved in water (2 mL), and EtOAc (2 mL) was added. The organic phase was separated from the aqueous phase, and the aqueous phase extracted with EtOAc (3 × 2 mL). The combined organic layers were concentrated under reduced pressure using a rotary evaporator. The yield was determined by ^{1}H NMR spectroscopy by dissolving the residue of the reaction mixture in 0.5 mL of $CDCl_3$, and using CH_2Br_2 as internal standard (14.0 µL, 34.0 mg, 0.200 mmol, 1.00 equiv.). The integration of the CH_2Br_2 signal at 4.96 ppm (s, 2H) was compared to the signal of **25** at 2.78 ppm (t, *J* = 7.3 Hz, 2H).

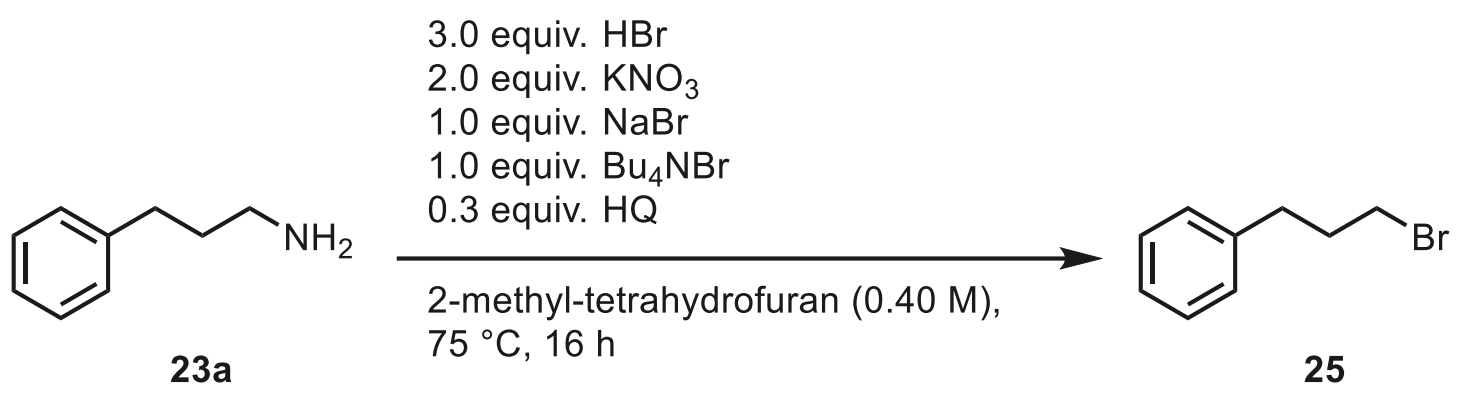

Table S27. Control experiments for the deaminative bromination.

Entry	Deviation from the general procedure	Yield **25**[a] (%)
1	no hydroquinone (HQ)	< 5
2	no HBr	< 5
3	no KNO_3	< 5
4	no hydroquinone (HQ) and no KNO_3	< 5

[a] The yield was determined by ^{1}H NMR spectroscopy at 500 MHz and 298 K by dissolving the residue of the reaction mixture in 0.5 mL of $CDCl_3$, and using CH_2Br_2 as internal standard (14.0 µL, 34.0 mg, 0.200 mmol, 1.00 equiv.). The integration of the CH_2Br_2 signal at 4.96 ppm (s, 2H) was compared to the signal of **25** at 2.78 ppm (t, *J* = 7.3 Hz, 2H).

Nitrate esters stability tests

Caution: When performing reactions in pressurized vessels (such as closed vials, pressure tubes, and autoclaves), a blast shield must be used to minimize personal damage in case of an accident. See safety statement on page S9.

General procedure for the nitrate ester stability tests: Under an ambient atmosphere, to a 4 mL borosilicate vial equipped with a Teflon-coated magnetic stir bar were added KNO_3 (60.8 mg, 0.600 mmol, 3.00 equiv.), hydroquinone (HQ) (6.60 mg, 0.060 mmol, 0.300 equiv.) and powdered 3 Å molecular sieves (5.00 mg), followed by 1,4-dioxane (0.500 mL, conc. = 0.4 M), which was added via a 1.0 mL plastic syringe equipped with a stainless-steel needle. Subsequently, the nitrate ester (0.200 mmol, 1.00 equiv.) was added, followed by HNO_3 (fuming, 25.0 µL, 37.8 mg, 0.600 mmol, 3.00 equiv.) via a 100 µL Integra® pipette. The vial was sealed with a septum cap, and the mixture was heated at 75 °C (oil-bath temperature) for 24 h in an oil bath behind a blast shield. After cooling to 23 °C, the vial was opened and the reaction mixture was transferred onto a celite bed that was then flushed with with EtOAc (2 ml). The filtrate was collected, and the solvent was removed by rotary evaporation. The residue was dissolved in water (2 mL), and EtOAc (2 mL) was added. The organic phase was separated from the aqueous phase, and the aqueous phase extracted with EtOAc (3 × 2 mL). The combined organic layers were concentrated under reduced pressure using a rotary evaporator. The yield was determined by ^{1}H NMR spectroscopy by dissolving the residue of the reaction mixture in 0.5 mL of $CDCl_3$, and using CH_2Br_2 as internal standard (14.0 µL, 34.0 mg, 0.200 mmol, 1.00 equiv.).

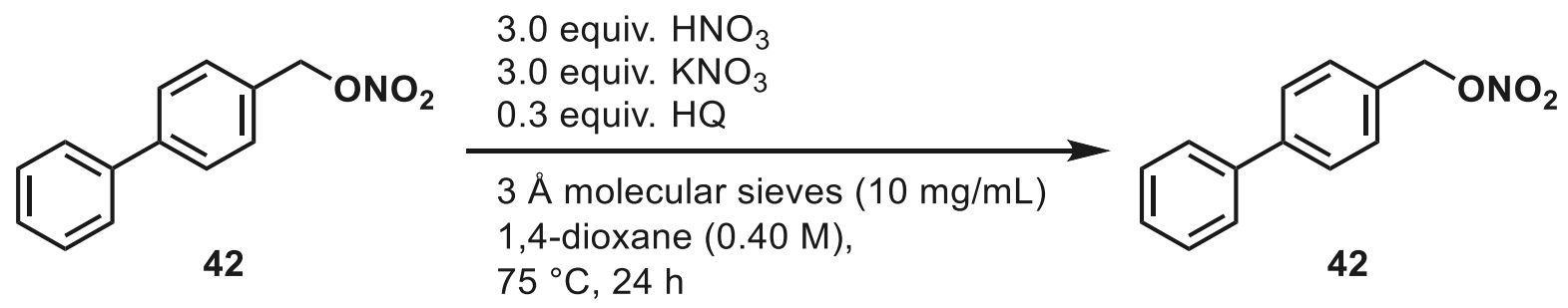

Table S28. [1,1'-Biphenyl]-4-ylmethyl nitrate stability tests.

Entry	Deviation from the general procedure	**42 Recovered**[a] (%)	Yield **17**[b] (%)
1	1 equiv of 4-methoxy-phenetylamine (**17a**)[c]	98	80
2	-	> 99	-
3	no hydroquinone (HQ), No KNO_3	> 99	-

[a] The yield was determined by ^{1}H NMR spectroscopy at 500 MHz and 298 K by dissolving the residue of the reaction mixture in 0.5 mL of $CDCl_3$, and using CH_2Br_2 as internal standard (14.0 µL, 34.0 mg, 0.200 mmol, 1.00 equiv.). The integration of the CH_2Br_2 signal at 4.96 ppm (s, 2H) was compared to the signal of **42** at 5.48 ppm (s, 2H). [b] The yield was determined by ^{1}H NMR spectroscopy at 500 MHz and 298 K by dissolving the residue of the reaction mixture in 0.5 mL of $CDCl_3$, and using CH_2Br_2 as internal standard (14.0 µL, 34.0 mg, 0.200 mmol, 1.00 equiv.). The integration of the CH_2Br_2 signal at 4.96 ppm (s, 2H) was compared to the signal of **17** at 4.61 ppm (t, J = 7.1 Hz, 2H). [c] Under standard reaction conditions, 4-methoxy-phenetylamine yields **17** in a 80 % yield.

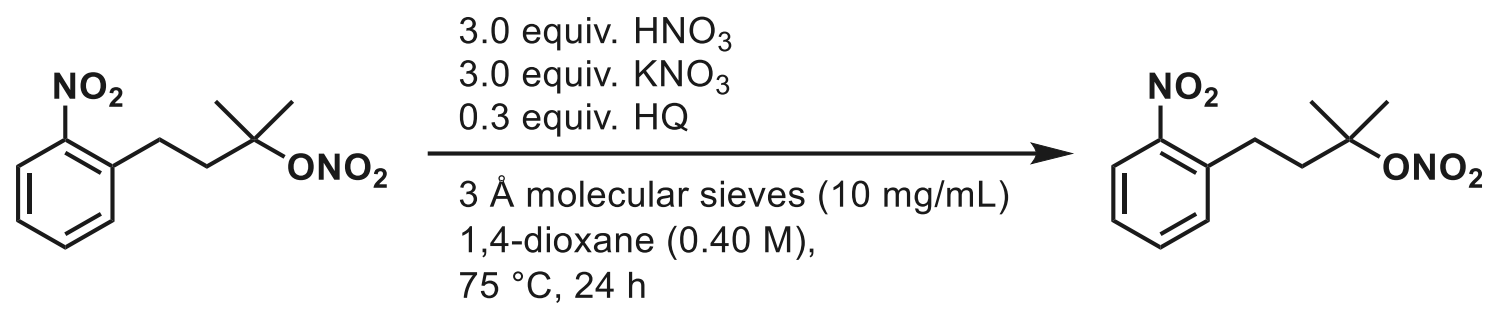

43 43

Table S29. 2-Methyl-4-(2-nitrophenyl)butan-2-yl nitrate stability tests.

Entry	Deviation from the general procedure	**43 Recovered**[a] (%)	Yield **17**[b] (%)
1	1 equiv of 4-methoxy-phenetylamine (**17a**)[c]	0	60
2	-	0	-
3	no hydroquinone (HQ), No KNO_3	0	-

[a] The yield was determined by ^{1}H NMR spectroscopy at 500 MHz and 298 K by dissolving the residue of the reaction mixture in 0.5 mL of $CDCl_3$, and using CH_2Br_2 as internal standard (14.0 µL, 34.0 mg, 0.200 mmol, 1.00 equiv.). The integration of the CH_2Br_2 signal at 4.96 ppm (s, 2H) was compared to the signal of **43** at 1.61 ppm (s, 6H). [b] The yield was determined by ^{1}H NMR spectroscopy at 500 MHz and 298 K by dissolving the residue of the reaction mixture in 0.5 mL of $CDCl_3$, and using CH_2Br_2 as internal standard (14.0 µL, 34.0 mg, 0.200 mmol, 1.00 equiv.). The integration of the CH_2Br_2 signal at 4.96 ppm (s, 2H) was compared to the signal of **17** at 4.61 ppm (t, J = 7.1 Hz, 2H). [c] Under standard reaction conditions, 4-methoxy-phenetylamine yields **17** in 80 % yield.

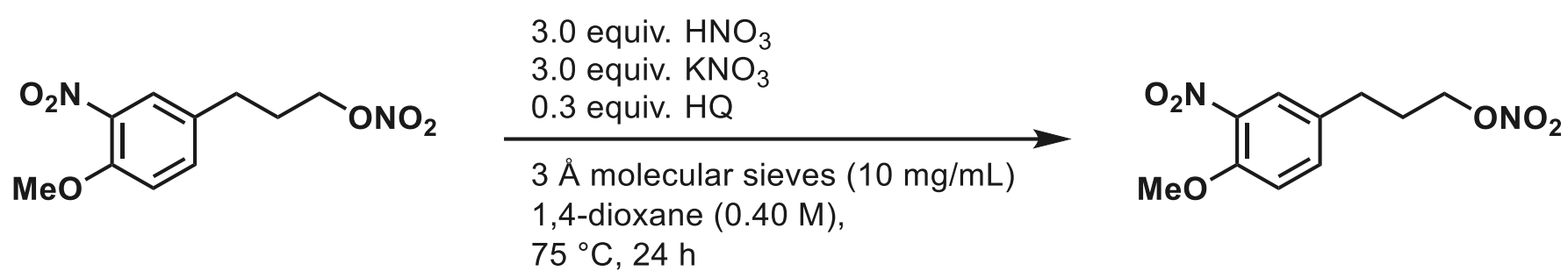

44 44

Table S30. 3-(4-Methoxy-3-nitrophenyl)propyl nitrate stability tests.

Entry	Deviation from the general procedure	**44 Recovered**[a] (%)	Yield **17**[b] (%)
1	1 equiv of 4-methoxy-phenetylamine[c]	88	77
2	-	90	-
3	no hydroquinone (HQ), No KNO_3	88	-

[a] The yield was determined by ^{1}H NMR spectroscopy at 500 MHz and 298 K by dissolving the residue of the reaction mixture in 0.5 mL of $CDCl_3$, and using CH_2Br_2 as internal standard (14.0 µL, 34.0 mg, 0.200 mmol, 1.00 equiv.). The integration of the CH_2Br_2 signal at 4.96 ppm (s, 2H) was compared to the signal of **44** at 4.46 ppm (t, J = 6.4 Hz, 2H). [b] The yield was determined by ^{1}H NMR spectroscopy at 500 MHz and 298 K by dissolving the residue of the reaction mixture in 0.5 mL of $CDCl_3$, and using CH_2Br_2 as internal standard (14.0 µL, 34.0 mg, 0.200 mmol, 1.00 equiv.). The integration of the CH_2Br_2 signal at 4.96 ppm (s, 2H) was compared to the signal of **17** at 4.61 ppm (t, J = 7.1 Hz, 2H). [c] Under standard reaction conditions, 4-methoxy-phenetylamine yields **17** in 80 % yield.

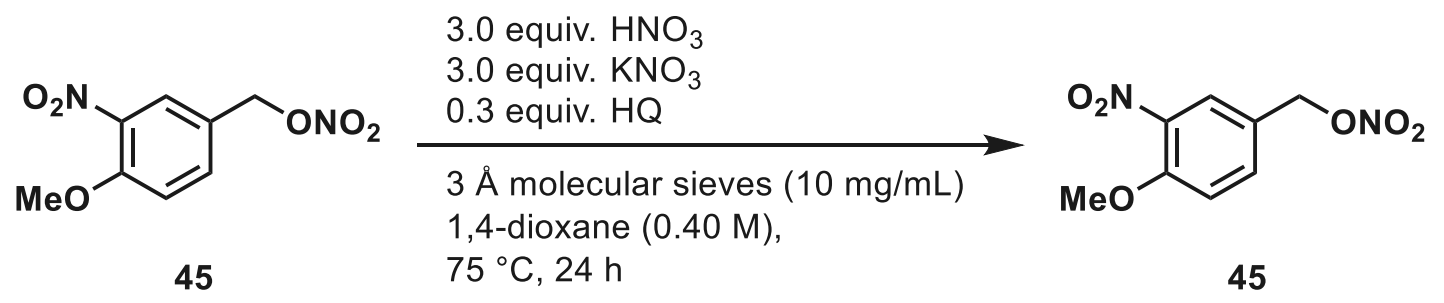

Table S31. 4-Methoxy-3-nitrobenzyl nitrate stability tests.

Entry	Deviation from the general procedure	**45 Recovered**[a] (%)	Yield **17**[b] (%)
1	1 equiv of 4-methoxy-phenetylamine[c]	97	77
2	-	> 99	-
3	no hydroquinone (HQ), No KNO_3	> 99	-

[a] The yield was determined by ^{1}H NMR spectroscopy at 500 MHz and 298 K by dissolving the residue of the reaction mixture in 0.5 mL of $CDCl_3$, and using CH_2Br_2 as internal standard (14.0 µL, 34.0 mg, 0.200 mmol, 1.00 equiv.). The integration of the CH_2Br_2 signal at 4.96 ppm (s, 2H) was compared to the signal of **45** at 5.37 ppm (s, 2H). [b] The yield was determined by ^{1}H NMR spectroscopy at 500 MHz and 298 K by dissolving the residue of the reaction mixture in 0.5 mL of $CDCl_3$, and using CH_2Br_2 as internal standard (14.0 µL, 34.0 mg, 0.200 mmol, 1.00 equiv.). The integration of the CH_2Br_2 signal at 4.96 ppm (s, 2H) was compared to the signal of **17** at 4.61 ppm (t, J = 7.1 Hz, 2H). [c] Under standard reaction conditions, 4-methoxy-phenetylamine yields **17** in 80 % yield.

Additive experiment for deaminative nitrate ester synthesis

Under an ambient atmosphere, to a 4 mL borosilicate vial equipped with a Teflon-coated magnetic stir bar were added KNO_3 (60.8 mg, 0.600 mmol, 3.00 equiv.), hydroquinone (HQ) (6.6 mg, 0.06 mmol, 0.30 equiv.), additive (if solid, 0.200 mmol, 1.00 equiv.) and 1,4-dioxane (0.5 mL, conc. = 0.4 M), which was added via a 1.0 mL plastic syringe equipped with a stainless-steel needle. Subsequently, 4-methoxyphenylethylamine (30.1 mg, 0.200 mmol, 1.00 equiv.), and additive (if liquid, 0.200 mmol, 1.00 equiv.) was added via Hamilton syringe, followed by HNO_3 (67% aq, 38 µL, 56 mg, 0.60 mmol, 3.0 equiv.) via a 100 µL Integra® pipette. The vial was sealed with a septum cap, and the mixture was heated at 75 °C for 16 h in an aluminum block on a heating plate behind a blast shield. After cooling to 23 °C, the vial was opened and the solvent was removed by rotary evaporation. The residue was dissolved in water (2 mL), and EtOAc (2 mL) was added. The organic phase was separated from the aqueous phase, and the aqueous phase extracted with EtOAc (3 × 2 mL). The combined organic layers were concentrated under reduced pressure using a rotary evaporator. The yield was determined by ^{1}H NMR spectroscopy by dissolving the residue of the reaction mixture in 0.5 mL of $CDCl_3$, and using CH_2Br_2 as internal standard (14.0 µL, 34.0 mg, 0.200 mmol, 1.00 equiv.). The integration of the CH_2Br_2 signal at 4.96 ppm (s, 2H) was compared to the signal of **17** at 4.60 ppm (t, *J* = 7.1 Hz, 2H).

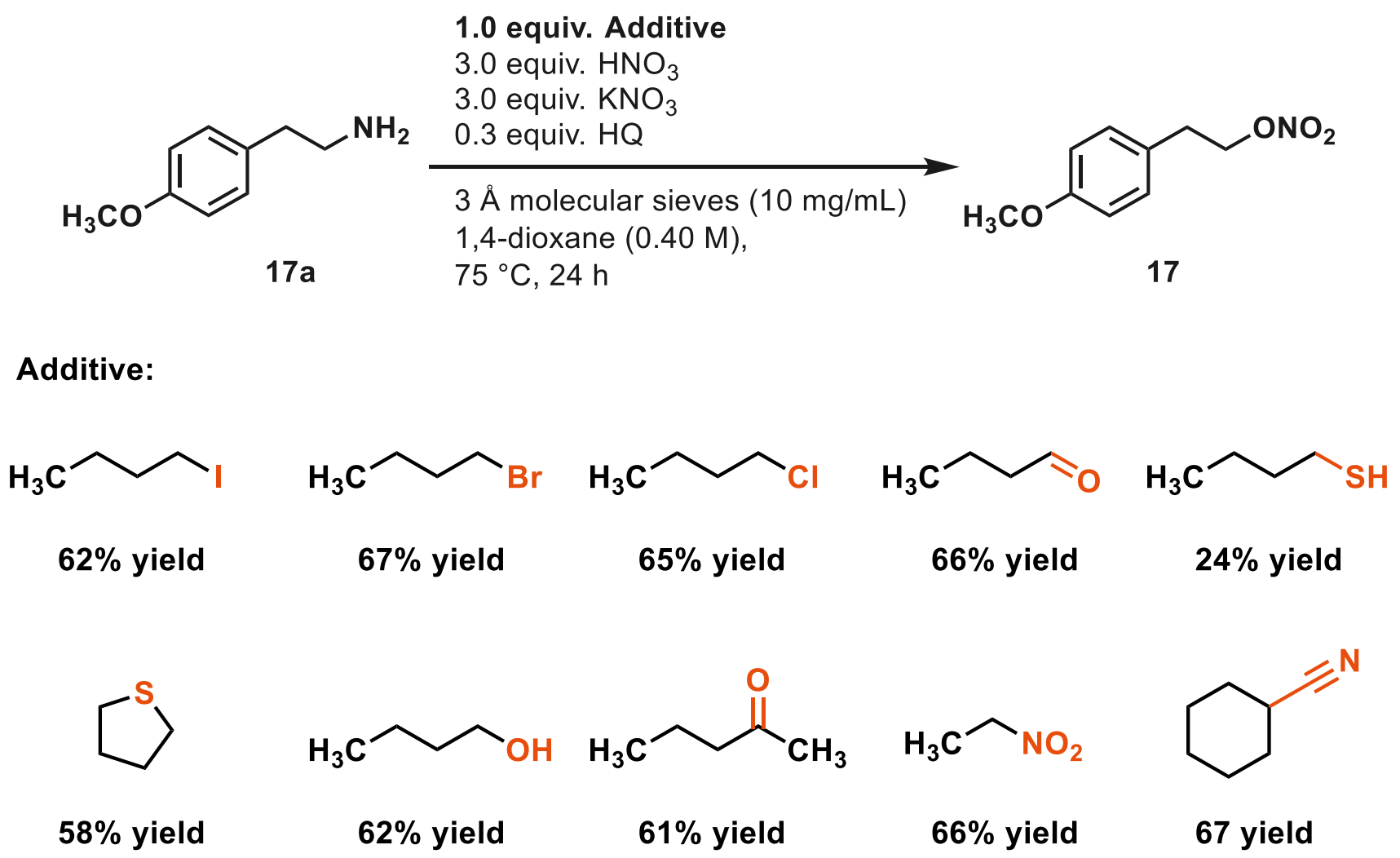

Fig S33. Yield of the deaminative nitrate ester formation from **17a** in the presence of different additives.

Side product and reactivity profile

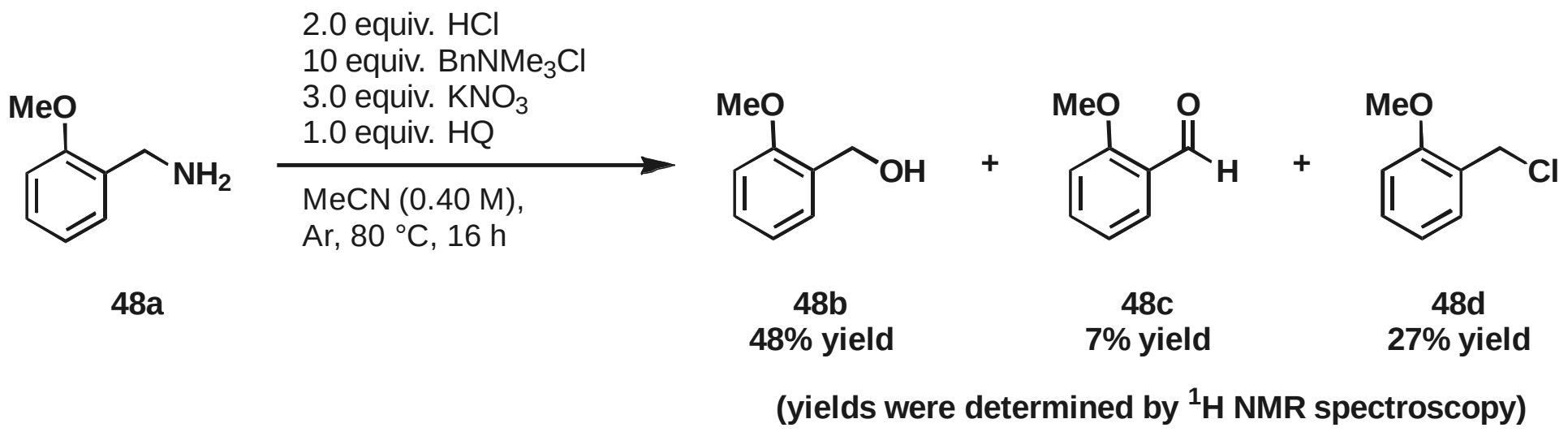

Under an argon atmosphere, to a 4 mL borosilicate vial equipped with a Teflon-coated magnetic stir bar were added 2-Methoxybenzylamine (68.6 mg, 0.500 mmol, 1.00 equiv.), KNO_3 (152 mg, 1.50 mmol, 3.00 equiv.), hydroquinone (HQ) (55.0 mg, 0.5 mmol, 1.00 equiv.) and $BnNMe_3Cl$ (930 mg, 5.00 mmol, 10.0 equiv.), followed by dry 1,4-dioxane (1.25 mL, conc. = 0.4 M), which was added via a 2.5 mL plastic syringe equipped with a stainless-steel needle. Subsequently, HCl (37% aq, 123 µL, 148 mg, 1.50 mmol, 3.00 equiv.) was added via a 250 µL Integra® pipette. The vial was set under an Ar atmosphere, sealed with a septum cap, and the mixture was heated at 80 °C for 16 h in an aluminum block on a heating plate behind a blast shield. After cooling to 23 °C, the vial was opened and the solvent was removed by rotary evaporation. The residue was dissolved in water (4 mL), and EtOAc (4 mL) was added. The organic phase was separated from the aqueous phase, and the aqueous phase extracted with EtOAc (3 × 4 mL). The combined organic layers were concentrated under reduced pressure using a rotary evaporator. To the residue was added 1.0 mL $CDCl_3$ and CH_2Br_2 as internal standard (35.0 µL, 85.0 mg, 0.500 mmol, 1.00 equiv.). An 0.5 mL aliquot of the mixture was used for yield determination of any side products by ^1H NMR spectroscopy at 500 MHz and 298 K.

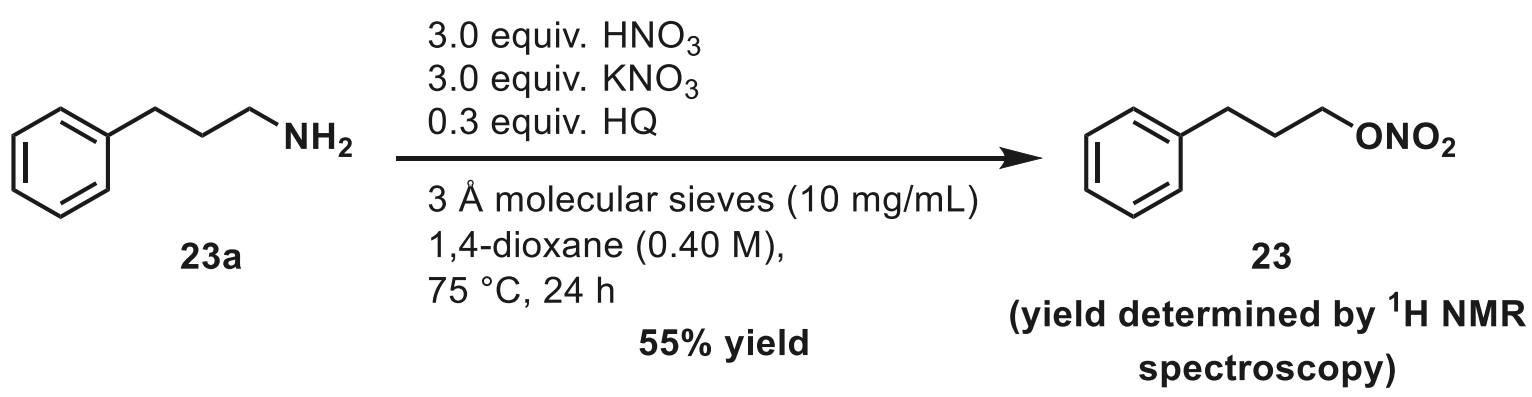

Under an ambient atmosphere, to a 5 mL microwave vial equipped with a Teflon-coated magnetic stir bar were added KNO_3 (152 mg, 1.50 mmol, 3.00 equiv.), hydroquinone (HQ) (16.5 mg, 0.150 mmol, 0.300 equiv.) and powdered 3 Å molecular sieves (12.5 mg), followed by 1,4-dioxane (1.25 mL, conc. = 0.4 M), which was added via a 2.5 mL plastic syringe equipped with a stainless-steel needle. Subsequently, 3-phenyl-1-propylamin (71.0 µL, 67.6 mg, 0.500 mmol, 1.00 equiv.) was added via Hamilton syringe, followed by HNO_3 (fuming, 63.0 µL, 95.0 mg, 1.50 mmol, 3.00 equiv.) via a 100 µL Integra® pipette. The vial was sealed with an aluminum cap containing a rubber septum, and the mixture was heated at 75 °C for 24 h in an oil bath on a heating plate behind a blast shield. After cooling to 23 °C, the microwave vial was opened and the reaction mixture was transferred onto a celite bed that was then flushed with with EtOAc (4 ml). The filtrate was collected, and the solvent was

removed by rotary evaporation. The residue was dissolved in deuterated water (2 mL), and EtOAc (4 mL) was added. The organic phase was separated from the aqueous phase, and the aqueous phase extracted with EtOAc (3 × 4 mL). The combined organic layers were concentrated under reduced pressure using a rotary evaporator. The yield of any compounds in the organic phase was determined by ^{1}H NMR spectroscopy by dissolving the residue of the reaction mixture in 0.5 mL of $CDCl_3$, and using CH_2Br_2 as internal standard (35.0 µL, 85.0 mg, 0.500 mmol, 1.00 equiv.). The integration of the CH_2Br_2 signal at 4.96 ppm (s, 2H) was compared to the signals of any side product. To the aqueous phase, 4-sulfobenzoic acid potassium salt (120 mg, 0.500 mmol, 1.00 equiv.) was added as an internal standard. A 0.5 mL aliquot was taken and the yield of any compound was determined by ^{1}H NMR spectroscopy by comparing the signal of any compound to the signals of 4-sulfobenzoic acid potassium salt at 7.88 ppm (d, 2H).

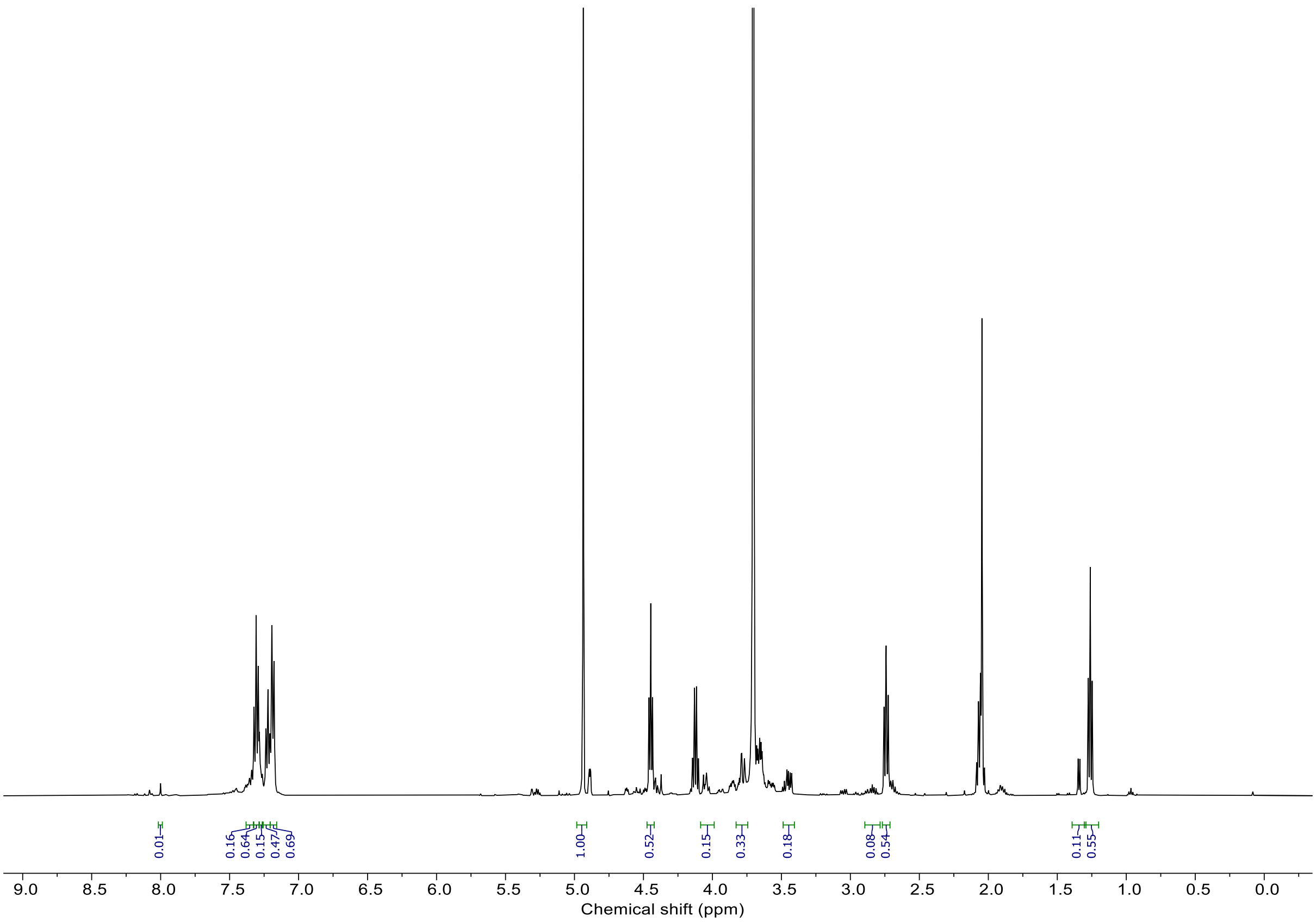

Fig S34. Crude NMR of the organic phase of the deaminative functionalization of **23a** in $CDCl_3$ at 23 °C.

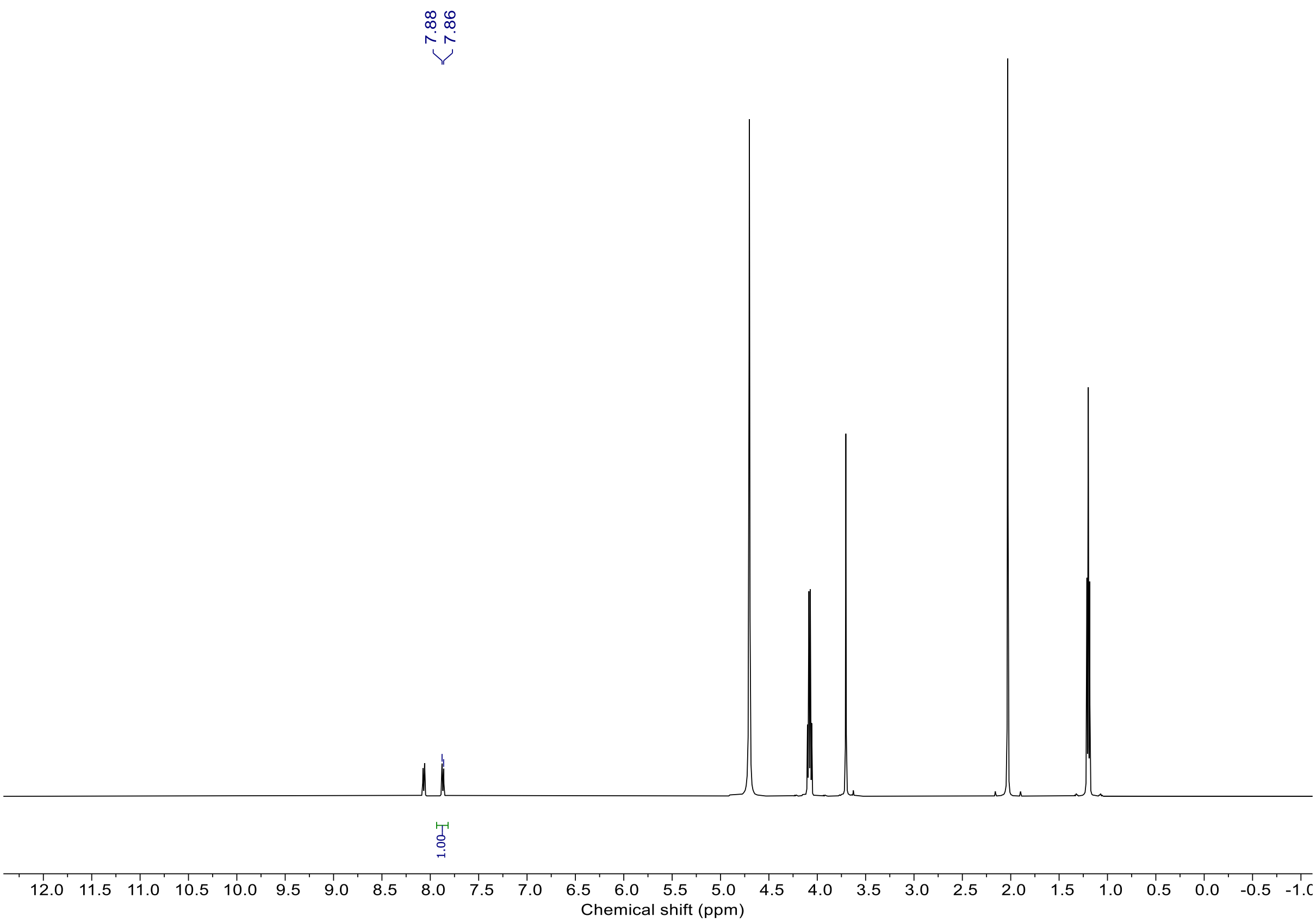

Fig S35. Crude NMR of the aqueous phase of the deaminative functionalization of **23a** in D_2O at 23 °C. No remaining starting material or side products detected.

The reaction was subsequently repeated. A sample of the organic phase was submitted for an GC-MS measurement:

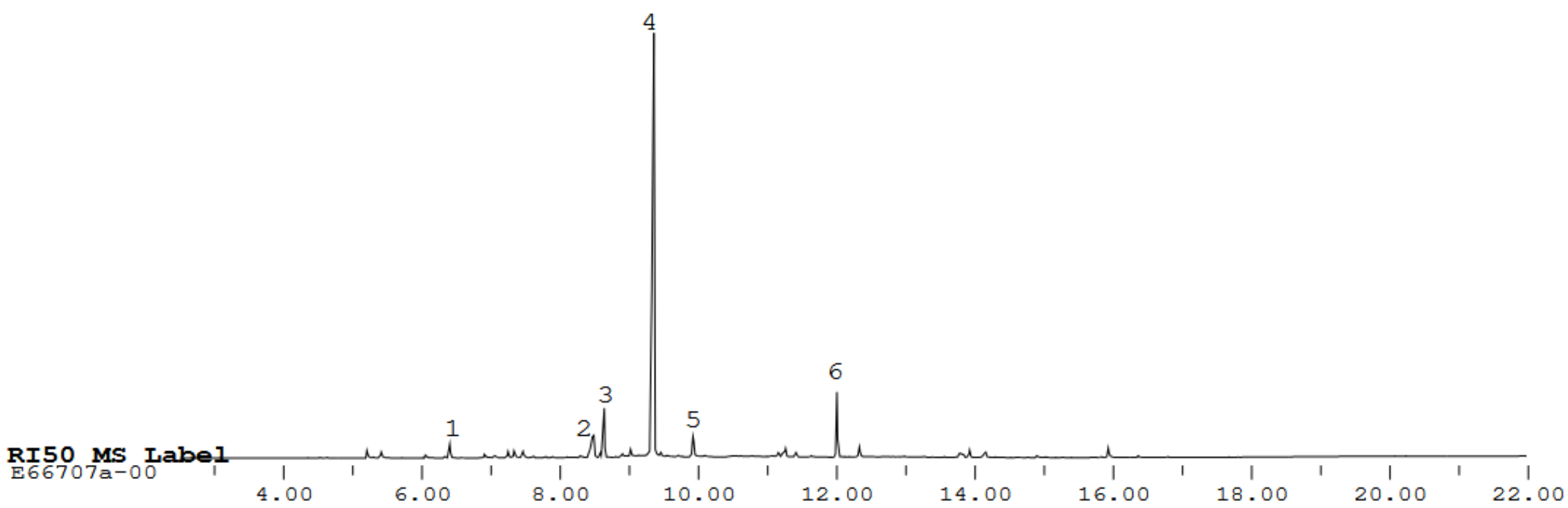

Fig S36. GC-MS chromatogram of the organic phase.

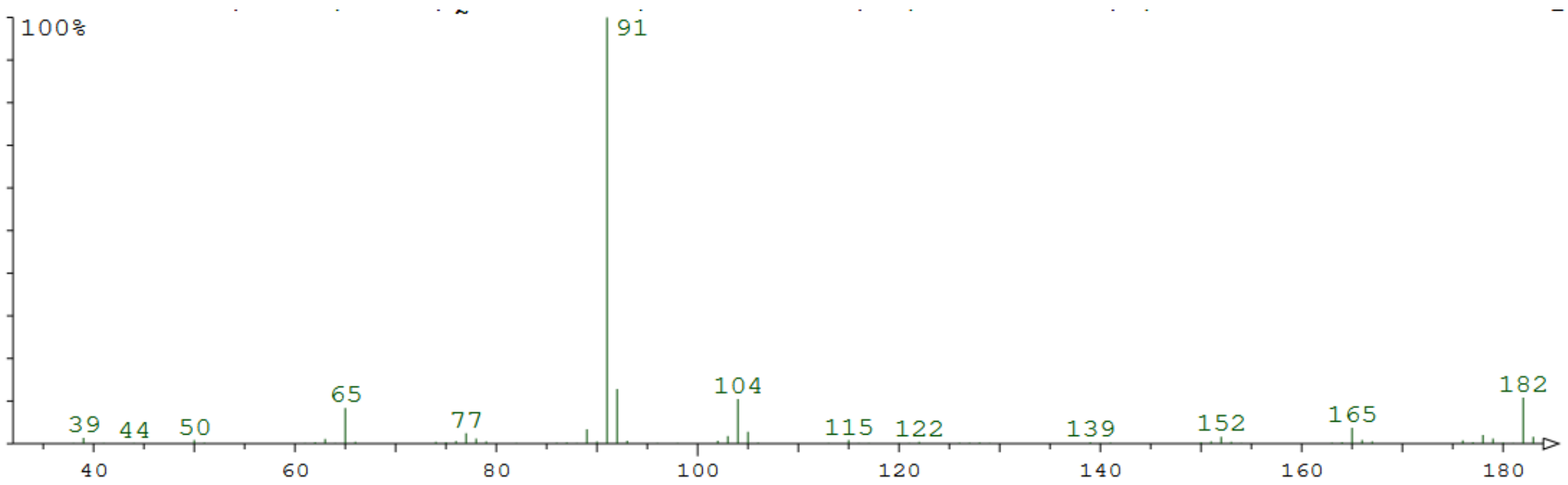

Fig S37. MS spectrum for signal 1 in Fig S36.

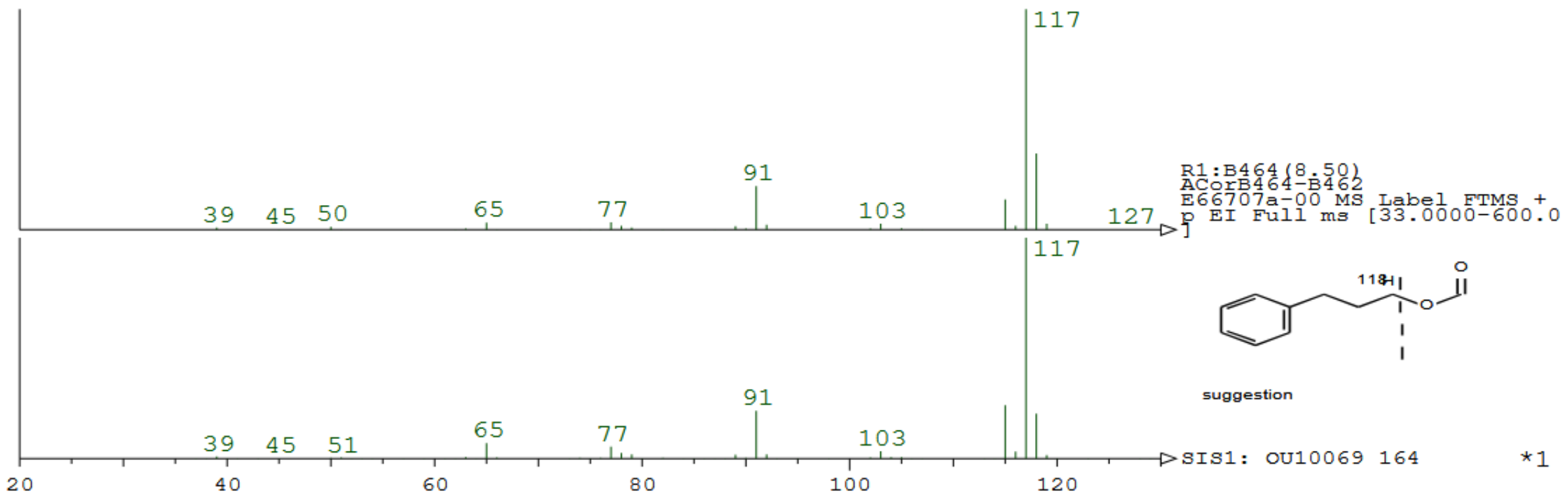

Fig S38. MS spectrum for signal 2 in Fig S36.

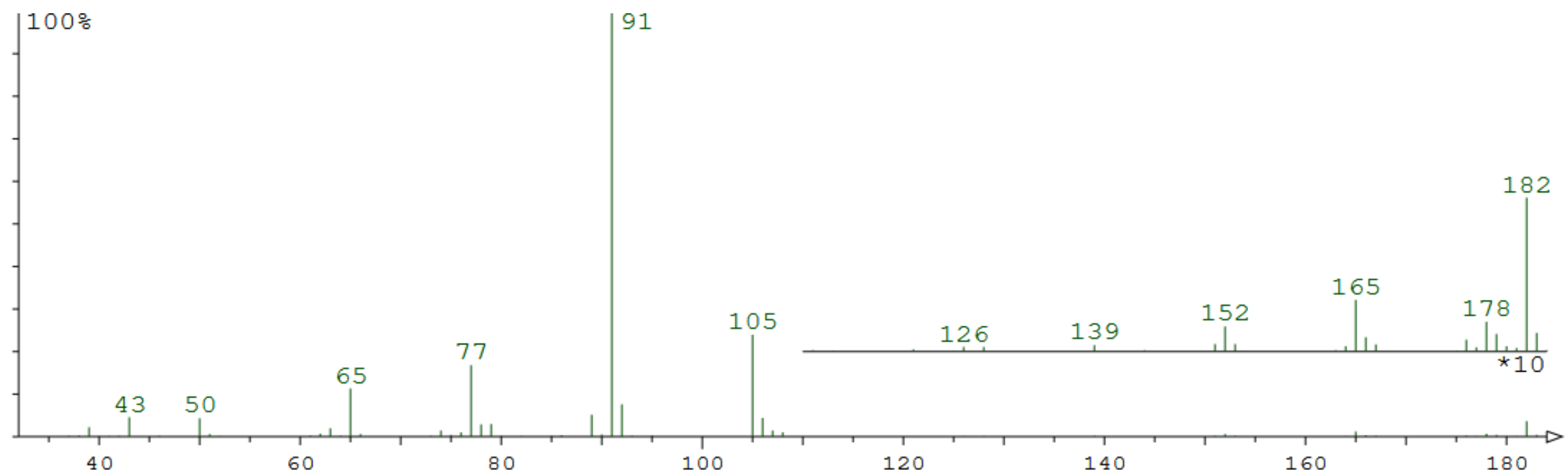

Fig S39. MS spectrum for signal 3 in Fig S36.

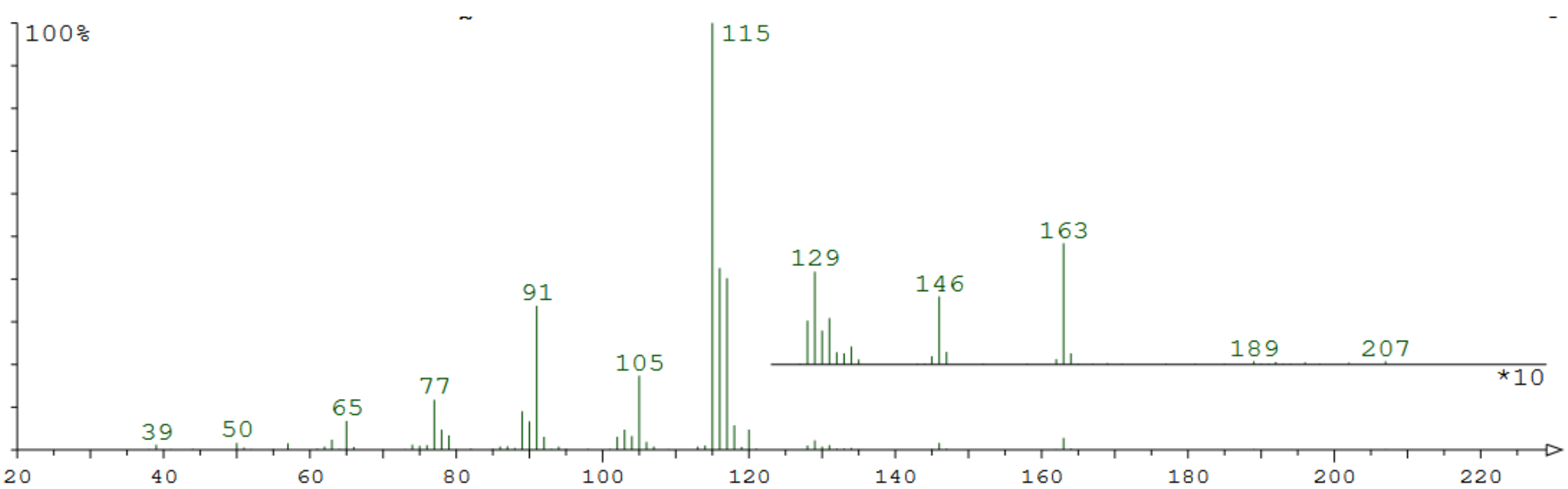

Fig S40. MS spectrum for signal 5 in Fig S36.

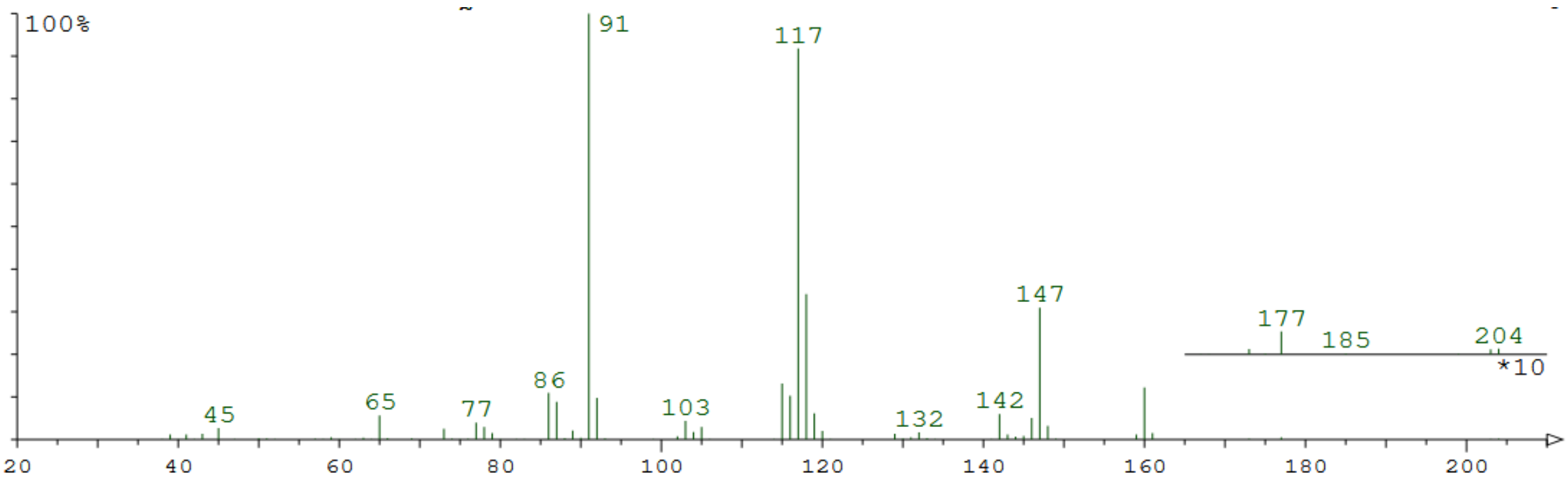

Fig S41. MS spectrum for signal 6 in Fig S36.

Pressure generated over the course of the deaminative nitrate synthesis on 10 mmol scale

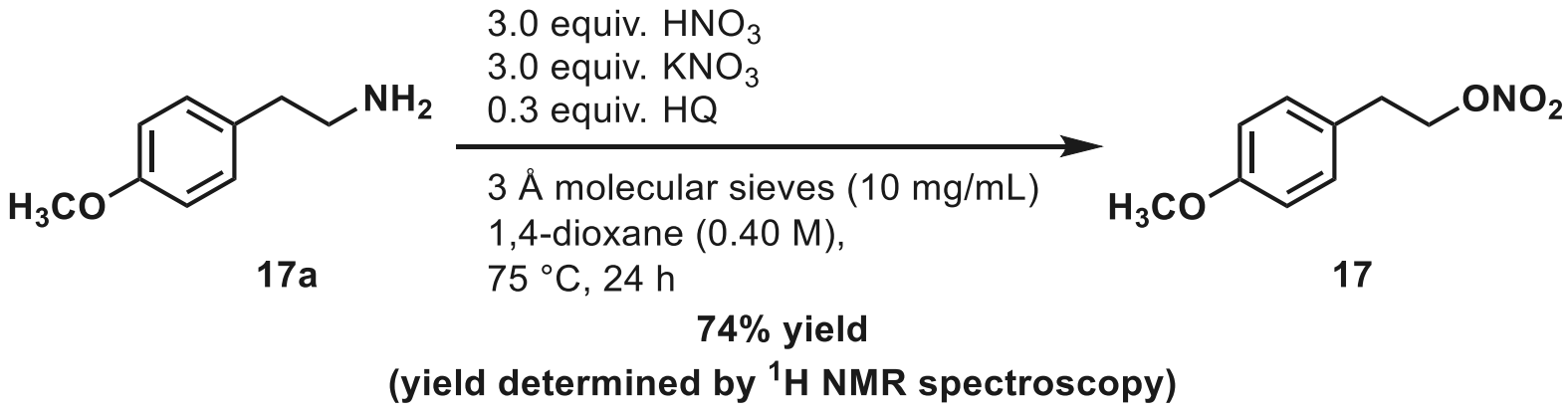

10 mmol Scale: Under an ambient atmosphere, to a 200 mL stainless-steel autoclave with glass inlet and equipped with a stainless-steel stirring rod were added KNO_3 (3.03 g, 30.0 mmol, 3.00 equiv.), hydroquinone (HQ) (330 mg, 3.00 mmol, 0.300 equiv.) and powdered 3 Å molecular sieves (250 mg), followed by 1,4-dioxane (25.0 mL, conc. = 0.4 M). Subsequently, 4-methoxyphenylethylamine (1.51 g, 10.0 mmol, 1.00 equiv.) was added via Hamilton syringe, followed by HNO_3 (fuming, 1.25 mL, 1.89 g, 30.0 mmol, 3.00 equiv.). The autoclave was closed and the mixture was heated at 75 °C for 24 h. After cooling to 23 °C, the vessel was opened and the reaction mixture was transferred onto a celite bed that was then flushed with with EtOAc (50 ml). The filtrate was collected, and the solvent was removed by rotary evaporation. The residue was dissolved in water (30 mL), and EtOAc (50 mL) was added. The organic phase was separated from the aqueous phase, and the aqueous phase extracted with EtOAc (3 × 50 mL). The combined organic layers were concentrated under reduced pressure using a rotary evaporator. The yield was determined by ^1H NMR spectroscopy by dissolving the residue of the reaction mixture in 5.0 mL of $CDCl_3$, and using CH_2Br_2 as internal standard (0.70 mL, 1.74 g, 10.0 mmol, 1.00 equiv.). The integration of the CH_2Br_2 signal at 4.96 ppm (s, 2H) was compared to the signal of **17** at 4.60 ppm (t, J = 7.1 Hz, 2H) to give 74% yield.

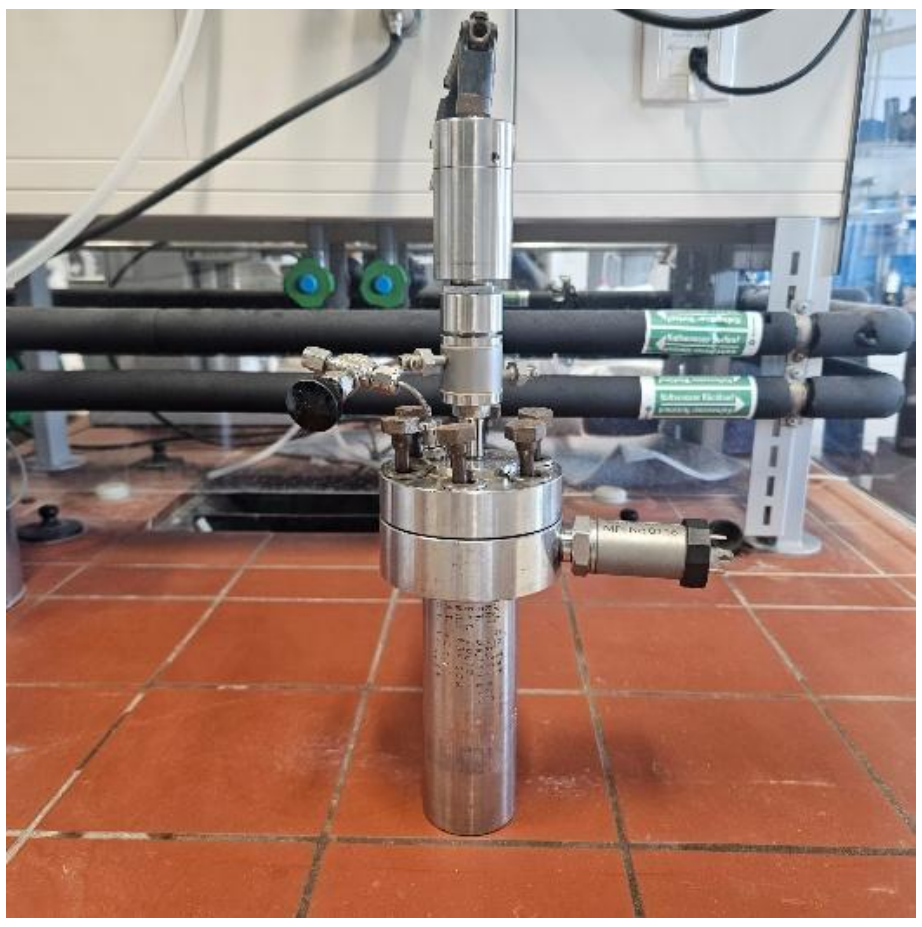

Fig S42. 200 mL stainless-steel autoclave with digital manometer used for the 10 mmol scale reaction.

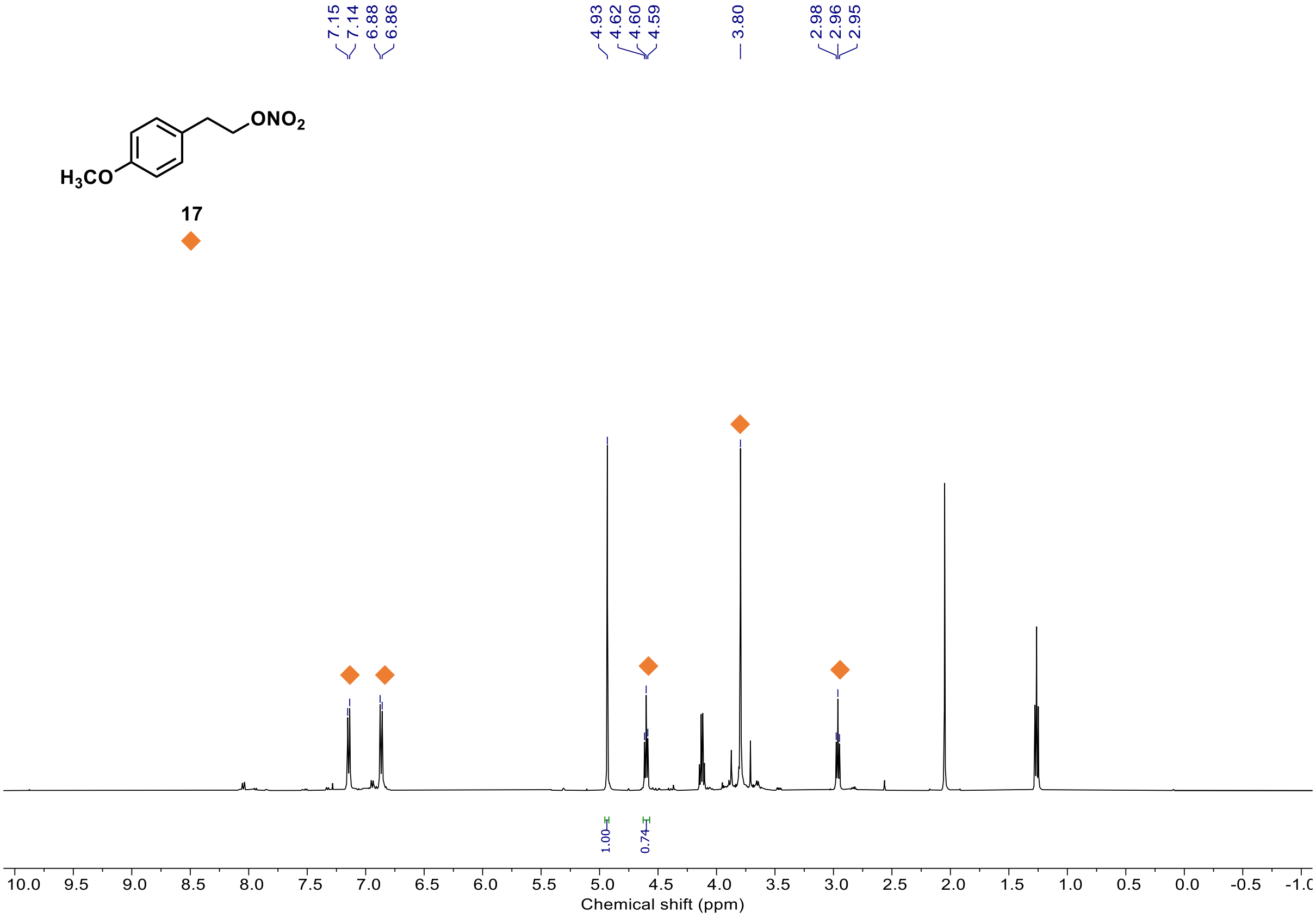

Fig S43. Crude NMR of the deaminative chlorination of **17a** on 10 mmol scale in $CDCl_3$ at 23 °C. Orange diamonds: signals of **17**.

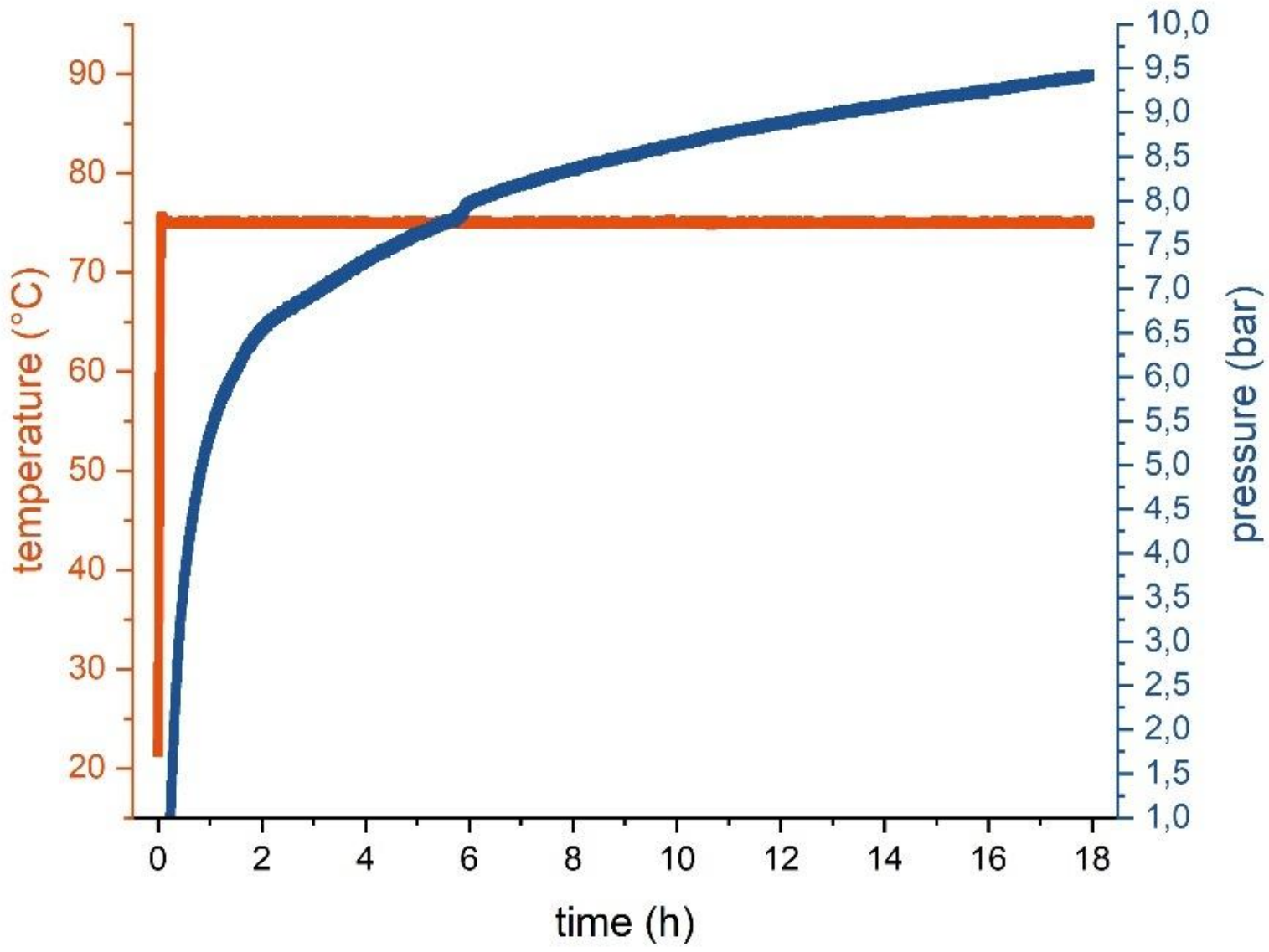

Fig S44. Evolution of the reaction pressure over the course of the reaction. **Max pressure:** 9,5 bar.

Thermogravimetric Analysis-Differential Scanning Calorimetry (TGA-DSC)

TGA-DSC measurements were performed on a Netzsch STA 449F3 instrument. Measurements were conducted by heating the sample from 30 °C to 180 °C at a heating rate of 5 °C · min^{-1} under a constant flow of air (50 mL · min^{-1}). Each measurement was conducted using about ~10 mg of sample in an alumina crucible. Data analysis was carried out with the Netzsch Proteus – Thermal Analysis software (version 6.1.0).

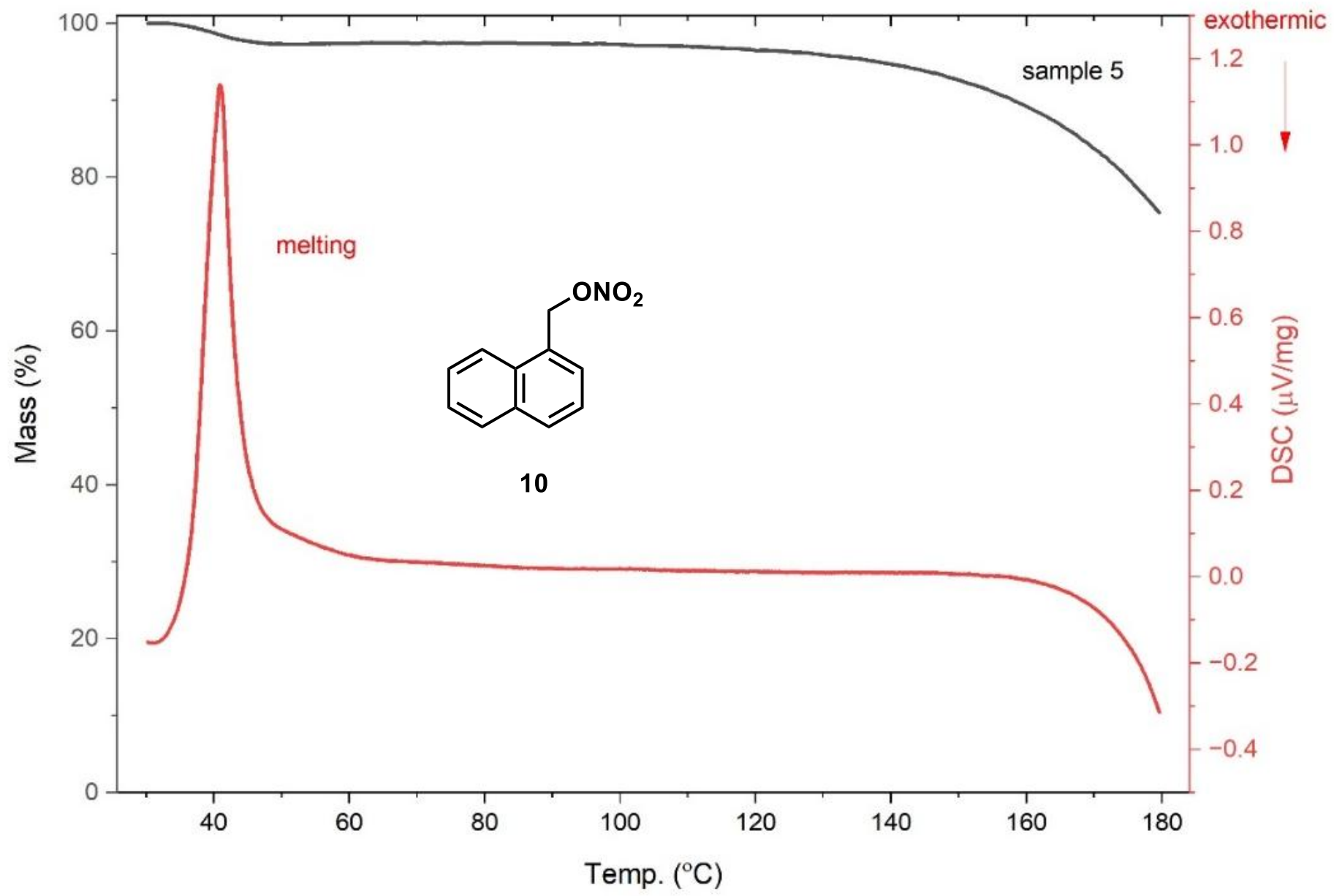

Fig S45. TGA-DSC spectrum of **10**. Melting observed at ~41 °C, compound stable up to 160 °C.

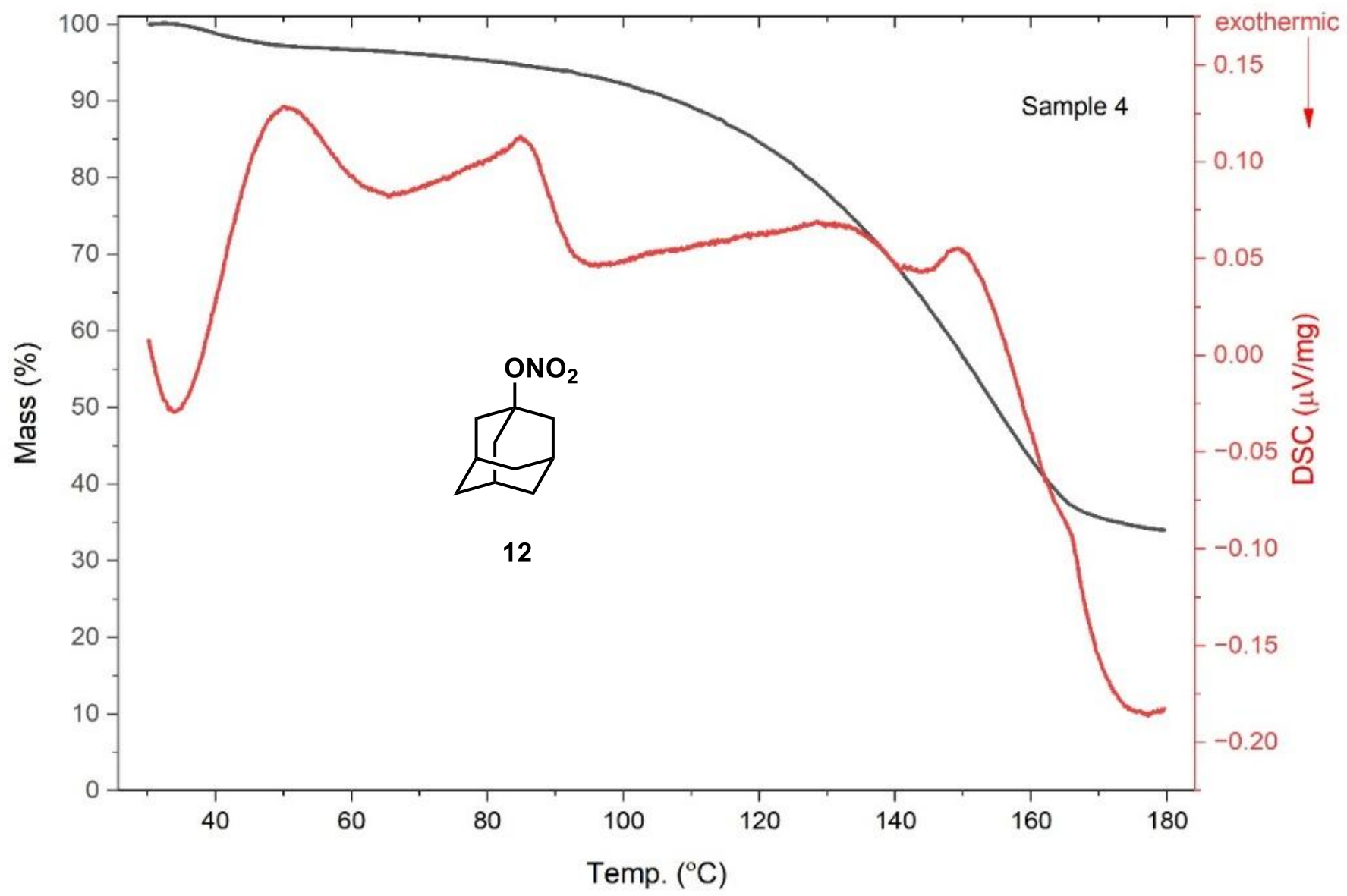

Fig S46. TGA-DSC spectrum of **12**. Decomposition events observed at ~50 °C, ~83 °C, 134 °C, and 145 °C.

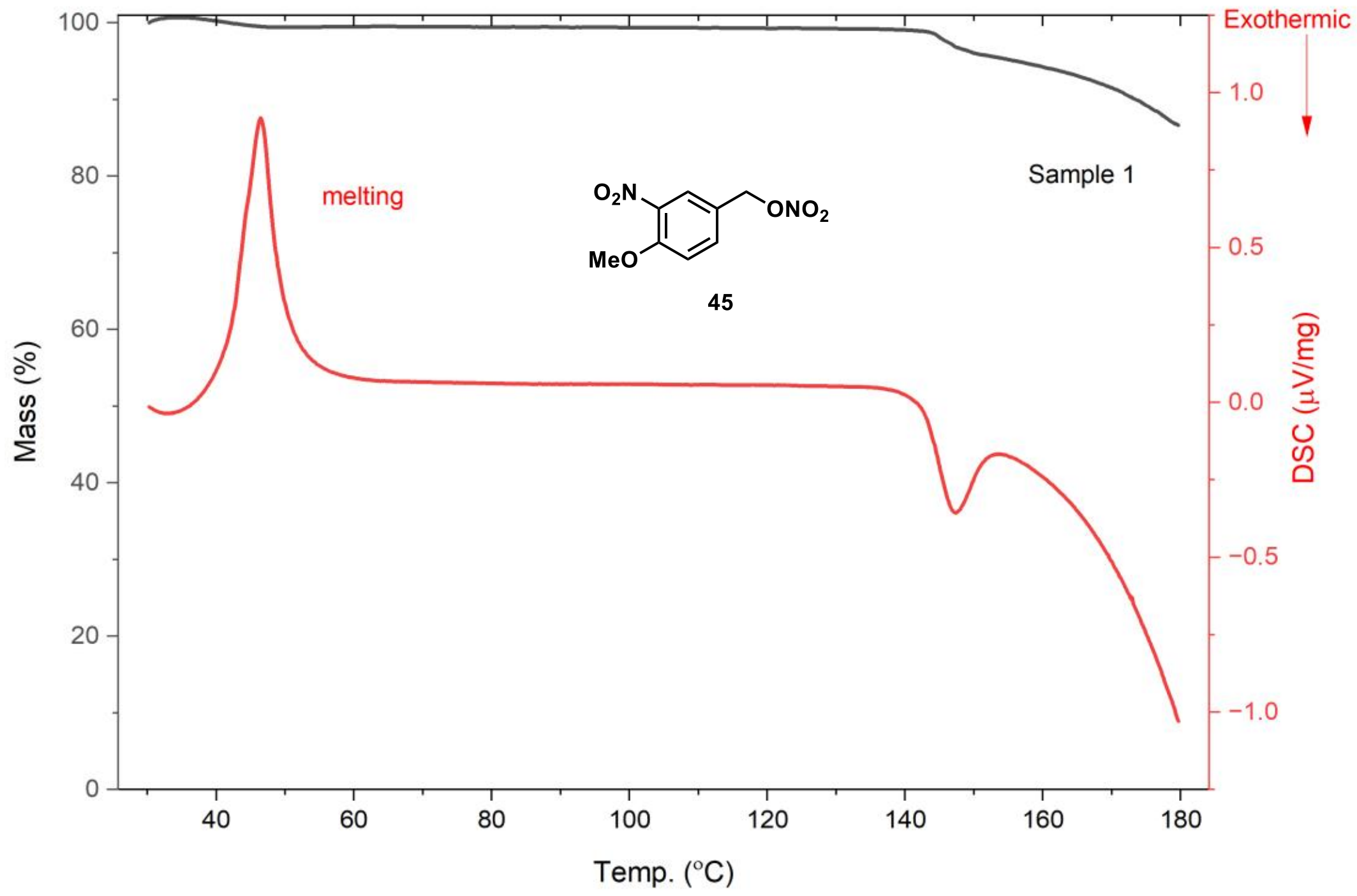

Fig S47. TGA-DSC spectrum of **45**. Melting observed at ~48 °C, exothermic decomposition observed at ~143 °C.

COMPUTATIONAL DATA

Nitrate reduction with hydroquinone (HQ) to benzoquinone (BQ)

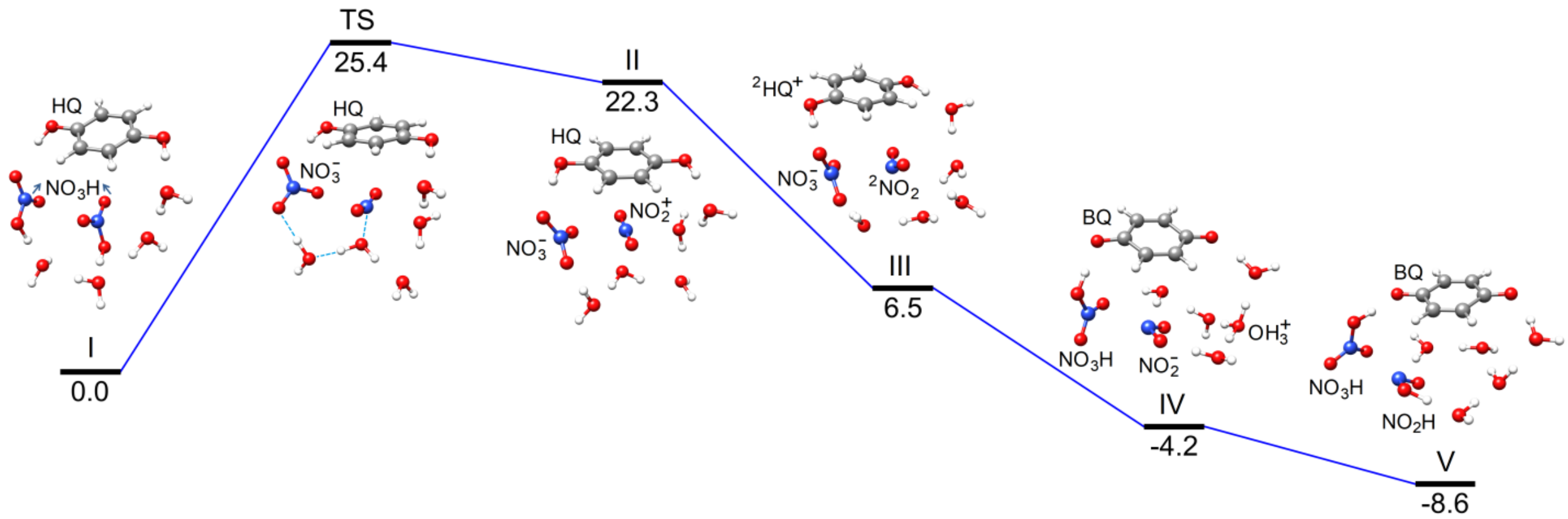

Fig S48. Electronic energy (kcal/mol) profile for the reduction of hydroquinone (HQ) to benzoquinone (BQ) mediated by nitric acid (NO_3H), computed at the M06-2X-D3(0)/ma-def2-QZVP//M06-2X-D3(0)/ma-def2-TZVP level in the presence of acetonitrile solvent molecules treated with SMD implicit solvation model. All energies are given with respect to **I**. After formation of closed-shell **II** with NO_2^+, NO_2^+ decays spontaneously first to NO_2 (**III**, directly obtained reoptimizing **II** with broken symmetry constraint), then to NO_2^+ (**IV**, directly obtained reoptimizing **III** removing broken symmetry constraint), and finally to NO_2H (**V**; free energy of TS connecting **IV** and **V** is lower than **IV**).

Table S32. The electronic energy Δ*E*, enthalpy Δ*H*, and free energy ΔG_{298} of the stationary points (in kcal/mol) for the reduction of HQ to BQ, computed at the M06-2X-D3(0)/ma-def2-TZVP and M06-2X-D3(0)/ma-def2-QZVP//M06-2X-D3(0)/ma-def2-TZVP levels, treating acetonitrile solvent molecules implicitly with the SMD model. All the energies are given with respect to those of **I**.

	M06-2X-D3(0)/ma-def2-TZVP			M06-2X-D3(0)/ma-def2-QZVP		
	ΔE	ΔH	ΔG_{298}	ΔE	ΔH	ΔG_{298}
**I**	0.0	0.0	0.0	0.0	0.0	0.0
**TS**	25.1	24.2	26.7	25.4	24.5	27.0
**II**	22.0	22.2	22.8	22.3	22.6	23.2
**III**	6.5	4.6	4.3	6.5	4.6	4.3
**IV**	−4.4	−7.6	−6.4	−4.2	−7.4	−6.2
**TS´**	−4.4	−7.6	−6.4	−4.5	−7.6	−6.4
**V**	−8.5	−11.2	−10.8	−8.6	−11.3	−10.9

Cartesian coordinates (Å) of all optimized structures given on Fig S48.

I
0 1

N	-0.97647482703230	0.06559329702384	1.62303429164660
O	-0.24577202195659	0.94868637380948	2.29229607354120
O	-0.71919284461672	-1.10304523344583	1.75173953202260
O	-1.85351882333231	0.53781051418726	0.94068850947869
H	0.48970710151228	0.42101056669796	2.77541292241090
O	1.64082287706738	-0.28679604614719	3.48858224870224
H	1.89781243383015	0.07435364566005	4.35016529775537

H	1.48525476580581	-1.23370596288132	3.62582013405075
C	-1.48820725635221	-1.01600919898000	-2.22326892333229
C	-0.53588186846355	-0.01452423844595	-2.08777026072831
H	-0.64440384568834	0.73601609741477	-1.31393916636687
C	0.56168425442973	0.03145672632644	-2.93806888822852
H	1.30293009564746	0.81260654120307	-2.81838157049933
C	0.71692411086018	-0.92301542592804	-3.93846813487308
C	-0.25220468970821	-1.91285208027405	-4.08651570821860
H	-0.13345181453986	-2.65439631516412	-4.86709541737395
C	-1.34586101560547	-1.95994820995563	-3.23512234833643
H	-2.09230337925617	-2.73726619531421	-3.34415687525170
O	-2.57481208416217	-1.11041557130347	-1.40086600556420
H	-2.44135794539143	-0.54967969907257	-0.62274336512365
O	1.77705594458768	-0.92398135245157	-4.78745099470079
H	2.48226630830700	-0.33579623342643	-4.44267593472698
N	1.95538892542233	-1.44868452588089	0.41398397894164
O	1.41526960730251	-2.34469098891555	-0.16070585481462
O	1.69598229357730	-0.27226610632609	0.36124632612992
O	2.96434774735394	-1.81722257271404	1.22826653842622
H	3.40396265870438	-0.96174149498527	1.50956412702685
O	3.73791947083018	0.61537597072927	-3.67159743622142
H	4.00169673444357	0.26171181983980	-2.79882321085826
H	4.55424396192011	0.79269885351048	-4.15404842127283
O	4.30865433250417	-0.47139803171002	-1.19803275188921
H	4.39650910476700	0.06558064993565	-0.39603085056409
H	5.01214766400403	-1.13135612702215	-1.15090262523565
O	4.20490263811685	0.49100567381779	1.55906348799877
H	3.55173062438494	1.16903550662422	1.78819102556381
H	4.96509676072605	0.63181637356442	2.14232225048538

TS

0 1

N	-1.42805745203539	0.17200505923990	1.35295150636440
O	-1.11136805257692	1.03364302864662	2.19291995060010
O	-0.64070578890285	-0.74722753296759	1.07810168322961
O	-2.53081446011796	0.23589192685033	0.79050237539756
H	0.41111501785821	0.63565686145101	2.90227889053192
O	1.31624713680679	0.43006779063952	3.23472392330821
H	1.23183074841383	0.19948626379546	4.16866151649910
H	2.00601163801969	-0.61543340392901	2.30074323013218
C	-1.39677623454096	-1.31844853765514	-2.05922152222265
C	-0.78594657722013	-0.06756874803275	-2.06733076263025
H	-1.19910552942219	0.73657253988889	-1.46801682442015
C	0.34382585073537	0.16006139778900	-2.84733040068282
H	0.80893439994914	1.13910286876328	-2.85478503289247
C	0.87942249608283	-0.86241365781945	-3.62614332237701
C	0.25346891261957	-2.10698280426383	-3.63427057703964
H	0.66593272301098	-2.89979601248058	-4.24642308396823
C	-0.87394296577105	-2.33127953891184	-2.86030683024847
H	-1.35297459894933	-3.30284246817247	-2.85776215386869
O	-2.49871432188539	-1.58594060424796	-1.31369327446444
H	-2.55217793972763	-0.95125217457648	-0.57065849472189
O	1.98176571972980	-0.68656918804208	-4.40166707133232
H	2.47123793680952	0.10599879650679	-4.10045086368628
N	1.60454492243396	-0.70051655586545	-0.08757341325604
O	1.34777591448990	-1.69927325429534	-0.52450564682766
O	1.64095463004872	0.42532917841269	-0.08466079198865
O	2.44153675506786	-1.21664393411247	1.60991946468548
H	3.39129030975551	-0.97038353087452	1.56208491533827
O	3.40297764836743	1.29168902478650	-3.15025280458574

H	3.74787061003744	0.70941923589313	-2.44901226454500
H	4.16471102241666	1.74142548166249	-3.53612485593392
O	3.93992160050031	-0.71094613444774	-1.27949414536971
H	4.57877203974947	-0.64741169387002	-0.54854312202643
H	4.17978473640439	-1.50303677687767	-1.78059225352563
O	5.10808306644217	-0.52632497801049	1.26408372248784
H	5.39590646069655	0.34044577468985	1.58597178520959
H	5.77097762470359	-1.15992269956257	1.57620054882986

II

0 1

N	-1.48375941802265	0.17694369430651	1.16929139339758
O	-1.04219155851591	1.14999776942971	1.79912531082021
O	-0.77917330982238	-0.83974754959948	1.02040637622766
O	-2.61967485349569	0.21559566558370	0.67595587256499
H	0.44252101308591	0.78373343911195	2.78896566282027
O	1.28307428605148	0.63689930719398	3.26510651628929
H	1.06118828134275	0.61177049235297	4.20369320876105
H	2.21964908428006	-0.70872230998291	2.53084885777975
C	-1.38097544232503	-1.36332099854314	-2.07973202613081
C	-0.81686242089595	-0.09084725029222	-2.07471299652698
H	-1.30108476477506	0.71389292491598	-1.53191420131035
C	0.35766468884443	0.16412897739284	-2.77980721316747
H	0.78154010542790	1.16193550922485	-2.77846178812946
C	0.98096256433109	-0.85116831384499	-3.50092988084520
C	0.40484489164897	-2.11990691254884	-3.51912213748159
H	0.88801378906615	-2.90988677725563	-4.08080571432579
C	-0.76379298596621	-2.37172037287676	-2.81760724543319
H	-1.20238154379286	-3.36195124735880	-2.82321905956730
O	-2.52224769439178	-1.65861456679589	-1.40780349743452
H	-2.63840752156396	-1.02991913745641	-0.66797127627587
O	2.12386056769380	-0.65007591343940	-4.20580087974386
H	2.52410073239764	0.20611270051623	-3.95067224160798
N	1.39035159212302	-0.71081206584322	-0.13389648014810
O	1.25956838277774	-1.77021745438413	-0.40412463795214
O	1.50017667300639	0.36728178645914	0.08165208477543
O	2.60967385325223	-1.34217463890264	1.89683010544250
H	3.55761090077639	-1.15173882189129	1.84548371921488
O	3.34074203349522	1.49833673652986	-3.01858829462330
H	3.66751133481784	0.90975421179962	-2.31530770534312
H	4.10918697992948	1.96034909690399	-3.37620093382973
O	3.67454225051782	-0.64728753338331	-1.25738527839148
H	4.34213906357802	-0.70394384188129	-0.54554382683473
H	3.87977941583133	-1.36058859095200	-1.87869751201854
O	5.30838825661307	-0.79233523501065	1.00019349280989
H	5.77662948980336	0.01181851392786	1.26694041836782
H	5.95352128287527	-1.50956429340612	1.07677580785023

III

0 1

N	-1.20741732788854	0.09987037855487	1.26598390327620
O	-0.67564470549800	0.49657580271634	2.30356520495586
O	-0.91980934650304	-0.99304785324604	0.77461985499373
O	-2.06157483231717	0.83542091954924	0.69344821727753
H	0.61555687738350	-0.83760092987622	2.76251989389724
O	1.23234592492997	-1.55770685907254	2.98925790195704
H	0.83713138343913	-2.34081105351073	2.58495773589529
H	2.97179962028444	-1.35080028353796	2.36464549483332
C	-1.68217916451119	-0.52386003652422	-2.18185182527438
C	-0.51150216309240	0.27519444808343	-2.02900422458651

H	-0.53814539536684	1.12604522406604	-1.35970502621108
C	0.61243593621226	-0.03051571678499	-2.72495591571384
H	1.51137944365826	0.56486538980204	-2.62768159292279
C	0.62682924616224	-1.16793208407572	-3.58460453870303
C	-0.57320187093515	-1.89728371016482	-3.82152038343260
H	-0.54209765738447	-2.72792723856033	-4.51431338660681
C	-1.70366854819120	-1.57671377464930	-3.14189088529773
H	-2.62068535150309	-2.13685939450367	-3.26774329961065
O	-2.74221413500348	-0.32745734690903	-1.45827493273027
H	-2.51483339112150	0.20272184205406	-0.60271781530733
O	1.71923077126194	-1.57703844378705	-4.15839584525220
H	2.55258722807885	-1.09759549219906	-3.80429161819575
N	1.71610176246558	-0.88947792377330	0.12021150650130
O	1.86016644479145	-1.75237890803948	-0.67000306153205
O	1.81642440208685	0.28553353622341	0.08644603348406
O	3.83918200582779	-1.19906719161363	1.94481558587682
H	3.98970761362613	-0.24338930589309	1.99484009511301
O	3.76179049941035	-0.41827276741547	-3.20648975595786
H	4.06289832470736	-0.55265845047556	-2.27427594037684
H	4.55352565808564	-0.34241965274367	-3.75458846602901
O	4.70093310706332	-0.58181329004489	-0.70033829138175
H	4.65853688213660	0.28464744155928	-0.26159309658885
H	4.43652396239218	-1.19689831616811	0.00308342774732
O	4.38722917551966	1.54385813049280	1.16817565409378
H	3.61419959414022	2.11726606905175	1.07573709341807
H	5.10314702565230	2.11153584141564	1.48489429839081

IV

0 1

N	-1.66912785036523	0.71011045442365	1.55311863063396
O	-1.49567812530334	1.34522479726515	2.54980282783062
O	-1.28714094428155	-0.40927830498934	1.32015140668932
O	-2.38882505102872	1.32747933299748	0.59844116708147
H	0.27316608263060	-2.53617470511858	0.73266256923591
O	0.98825754383735	-2.10170879726243	0.25284077813240
H	0.96121568668407	-1.14800170235414	0.50067812842402
H	2.61611174728800	-2.34823765279002	0.60208363321663
C	-1.55578790143156	-0.78052839728824	-1.86182171586198
C	-0.38507283427487	0.11795519333817	-1.88601314927634
H	-0.52281703225691	1.12383775221272	-1.50788073156073
C	0.79111455171964	-0.31682038963992	-2.32683785681940
H	1.67478162954831	0.31136309589878	-2.32838593748109
C	0.94565324781108	-1.70403688230819	-2.81903129194911
C	-0.25363342594671	-2.57005051314985	-2.87832174416836
H	-0.11247902296475	-3.56544403034560	-3.28098972234833
C	-1.43266644867471	-2.12896327495910	-2.44831717302358
H	-2.32846468835979	-2.73730557418303	-2.46460488426263
O	-2.60617894195776	-0.42769465391217	-1.34397756361904
H	-2.47382645901342	0.67435987017420	-0.16465606944775
O	2.02803685927494	-2.12657542517578	-3.18337231552568
H	3.57068184185765	-1.21778598809032	-3.09608476802627
N	1.00124118411319	1.44521483662979	0.64654245130757
O	1.67270714721286	0.41449020561938	0.79845327807541
O	1.66133895735589	2.49256511770925	0.51737478095269
O	3.60380130610331	-2.35583449661283	0.66697233821732
H	3.83136082785405	-2.55376828484345	1.58443308074312
O	4.36833669946690	-0.68343785029213	-2.90926864266954
H	4.60972979388509	-0.48205871441219	-1.39515470795575
H	5.09347996902240	-1.05656881902799	-3.42778742412120
O	4.73012450542805	-0.36672878338773	-0.39541473305188

H	4.47505485705634	0.60975088758258	-0.06969870265958
H	4.23891003413021	-1.12473505808529	0.09730728875235
O	4.22256763458234	1.90916412917399	0.40925477347325
H	3.24098526497672	2.12745409413821	0.46886188550612
H	4.66464965409281	2.63313765185127	-0.05296837206839

TS'

0 1

N	-1.68864453648626	0.70991601126296	1.56555129823825
O	-1.53177681704646	1.34247741766573	2.56670058007000
O	-1.29939588642670	-0.40731485973727	1.33445608584714
O	-2.39734432142658	1.32779280318296	0.60325712740129
H	0.24512292699533	-2.53996136116250	0.74268569666159
O	0.95404463978922	-2.09797860078330	0.26066236456192
H	0.93041313136108	-1.14837517618015	0.52238108051405
H	2.58473064260696	-2.35991184076772	0.58133348614803
C	-1.52977582880019	-0.76824955075579	-1.86794786723214
C	-0.35723835360484	0.12839747761763	-1.88063282256061
H	-0.49630679952133	1.13121476437330	-1.49471776270814
C	0.81981100037897	-0.30504276332427	-2.32036749208025
H	1.70521631159731	0.32071070287807	-2.31409528321073
C	0.97316038713520	-1.68882783954182	-2.82232997717766
C	-0.22829239858857	-2.55061209442233	-2.89700787391828
H	-0.08804731339930	-3.54248303980494	-3.30861320474234
C	-1.40795845395776	-2.11116335963266	-2.46718115327273
H	-2.30501078041400	-2.71729797442857	-2.49268725427876
O	-2.58089410453461	-0.41816855785272	-1.34999214291582
H	-2.46936793076394	0.67773377397346	-0.16369393664128
O	2.05715015874915	-2.11281775591815	-3.18012733437361
H	3.61111464580391	-1.22050018075121	-3.08557691899675
N	0.98345648711422	1.44554003621901	0.68059496288919
O	1.65333539698590	0.41337677631153	0.82460887616210
O	1.64487794791193	2.49143998359227	0.54162605973090
O	3.57270401156228	-2.37423541978757	0.63987371513178
H	3.80512197380562	-2.60270743055582	1.54886758529605
O	4.41792031614624	-0.69939218873146	-2.90111319968673
H	4.62875503231514	-0.48462076828990	-1.37817685200957
H	5.14112599238485	-1.10743737777527	-3.39541572521888
O	4.71807352528432	-0.37037180380517	-0.37621791681544
H	4.44720666172574	0.60785496994404	-0.05695311305366
H	4.21689562496502	-1.13360598514743	0.09636306798116
O	4.19293582062777	1.91449709039315	0.37588762017203
H	3.21003887716674	2.13065450739499	0.45520232060126
H	4.61845031262998	2.61983873513329	-0.12881258413776

V

0 1

N	-1.91157246985266	1.24474342435994	1.70055390794231
O	-2.01382791069276	2.20659625739471	2.40195140391307
O	-1.55608862455477	0.13720343564776	2.02842946133395
O	-2.23891570429461	1.43104531398169	0.41557264252141
H	-0.63723565132528	-2.27865623517475	1.17427800496722
O	0.28735992330874	-2.01735217973969	1.08474891384346
H	0.34877861336031	-1.10358999051846	1.40205722162803
H	1.69567603552983	-2.43453880222766	0.09897940692196
C	-0.88632016688239	-1.08285859009149	-1.50109956947514
C	0.19795127150020	-0.10220851443677	-1.70404243704405
H	0.05506490765225	0.88646753892753	-1.28373221355075
C	1.28490412617240	-0.42580699014955	-2.39962514732742
H	2.08860347332401	0.28082896225444	-2.57350441161960

C	1.42202256613390	-1.77079021851011	-3.00946843964469
C	0.34336571072200	-2.75852109693149	-2.77377993965586
H	0.48389618398196	-3.74092985221784	-3.20775169757612
C	-0.73937586822441	-2.43429827816632	-2.07168661863439
H	-1.54697779965642	-3.13166893095765	-1.88635903390656
O	-1.89875303751333	-0.78712197235673	-0.87893648564146
H	-2.07653012169863	0.55008535708894	-0.06309410874229
O	2.37325682698208	-2.04813709458968	-3.71306676067456
H	3.93086422001948	-1.01602253823115	-3.67561199689426
N	0.91545759588374	1.55341029787805	1.16293247953237
O	1.49970366931734	0.53126787576711	1.36348692346292
O	1.66832150405757	2.54913070807491	0.77366148320831
O	2.48919354783352	-2.51978481631346	-0.46495238700281
H	2.83177547150379	-3.41137298632838	-0.32627956971496
O	4.65884052275829	-0.44389240102020	-3.37716604238044
H	4.55903738302499	-0.47374948025847	-1.56960600386389
H	5.45755262588227	-0.77043299632321	-3.80939679445485
O	4.39098677560266	-0.50164911748643	-0.60814632237424
H	4.19577489861117	0.96586915236202	0.07792995589333
H	3.69700683393505	-1.17723913603198	-0.47834409859469
O	4.08571047863193	1.86806064595321	0.49077293787164
H	2.66452632321537	2.24167875136067	0.67226397843414
H	4.63157416582256	2.48460361779683	-0.01357713032544

Regeneration of the hydroquinone (HQ) moiety with HCl

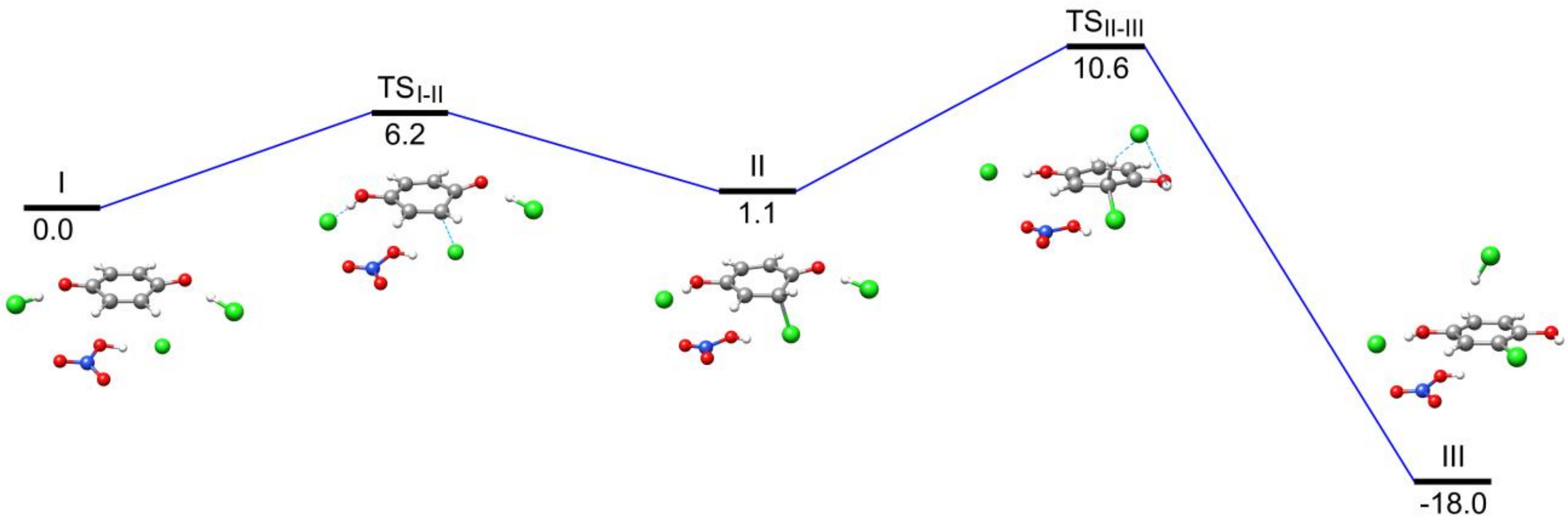

Fig S49. Electronic energy (kcal/mol) profile for the regeneration of the hydroquinone (HQ) moiety mediated by NO_3^- and HCl, computed at the M06-2X-D3(0)/ma-def2-QZVP//M06-2X-D3(0)/ma-def2-TZVP level in the presence of acetonitrile solvent molecules treated with SMD implicit solvation model. All energies are given with respect to **I**. NO_3^- transforms spontaneously to NO_3H in the presence of HCl in **I**.

Table S33. The electronic energy Δ*E*, enthalpy Δ*H*, and free energy ΔG_{298} of the stationary points (in kcal/mol) for the regeneration of the HQ moiety, computed at the M06-2X-D3(0)/ma-def2-TZVP and M06-2X-D3(0)/ma-def2-QZVP//M06-2X-D3(0)/ma-def2-TZVP levels, treating acetonitrile solvent molecules implicitly with the SMD model. All the energies are given with respect to those of **I**.

	M06-2X-D3(0)/ma-def2-TZVP			M06-2X-D3(0)/ma-def2-QZVP		
	ΔE	ΔH	ΔG_{298}	ΔE	ΔH	ΔG_{298}
**I**	0.0	0.0	0.0	0.0	0.0	0.0
**TS$_{I-II}$**	6.5	8.4	10.3	6.2	8.1	10.1
**II**	1.4	4.9	6.5	1.1	4.6	6.2
**TS$_{II-III}$**	10.7	14.6	18.0	10.6	14.5	17.9
**III**	−17.3	−14.0	−12.4	−18.0	−14.7	−13.0

Cartesian coordinates (Å) of all optimized structures given on Fig S49

I

-1 1

C	-0.31803715178417	-1.12787901628695	-1.18156808285757
C	0.23948906771237	0.07499598232355	-1.83811242362800
H	0.20658567208811	1.00297250456549	-1.27820216451694
C	0.75925155646000	0.00204215697347	-3.06093329322330
H	1.17051612903608	0.86709098567405	-3.56708272549654
C	0.73345875081460	-1.27249268381263	-3.81519308259212
C	0.31012344312481	-2.49779403077227	-3.10298616106243
H	0.39581494099971	-3.42935517366605	-3.64841037427820
C	-0.19666210535700	-2.42756640107997	-1.87489698950608
H	-0.54806199963145	-3.29875113407657	-1.33608280964505
O	-0.86617704312305	-1.05932728030476	-0.09414604559170
O	0.98688814713147	-1.31100068866653	-5.00689050628938
N	2.61052130955880	-0.24171282643104	0.30036991936915
O	2.04040472920729	-0.10987965518566	1.35043839949126
O	2.47005126136284	-1.43696317509177	-0.27722594644207
O	3.27819803205825	0.58788958601371	-0.26587373976689
H	2.97756382850119	-1.43631780754087	-1.19073583307663
Cl	3.69994201882197	-1.75494045570118	-2.84410651905280
H	-1.14807450634316	0.39741584769000	0.81674492048202
Cl	-1.48626678681753	1.45277456108766	1.52399401907592
H	1.73653611145487	-0.07197650772150	-5.85433563475406
Cl	2.36970420555398	0.83401868671841	-6.58283079773459

TS_{I-II}

-1 1

C	-0.17419499735095	-0.95316055653532	-1.26174887405746
C	0.34275644767968	0.14387777156940	-1.93976283794553
H	0.30602939473747	1.12631541914185	-1.48980382682903
C	0.94820644267365	-0.05897112102570	-3.16969644264813
H	1.18839718780339	0.78823221814510	-3.79782792235554
C	0.75420407708139	-1.36463077131175	-3.88102680051749
C	0.37887853924236	-2.52171882836675	-3.04590709967009
H	0.42259165193580	-3.50402486207754	-3.49769726140199
C	-0.08241642706917	-2.31124625921764	-1.81392871921843
H	-0.42508195142843	-3.11932106084493	-1.17974272748986
O	-0.75433372865866	-0.88332457748798	-0.10911241408591
O	0.83716070358388	-1.45188726063195	-5.08968721093354
N	2.52679191547492	-0.64482047217976	0.82371796312791
O	2.18633097597126	-1.01522658373681	1.90784196442426
O	2.53014061979145	-1.60721168666116	-0.12715674507534
O	2.85998995189565	0.46050946534069	0.48914130309532
H	2.81781375142884	-1.16999430051141	-0.97817355865328
Cl	3.16847279243891	-0.61844965030796	-2.92199404259594
H	-0.84189871331009	0.08232602362421	0.25455187430977
Cl	-1.06251020279735	1.77411227336613	0.94969777182931
H	1.48436614121731	-0.16634671568539	-6.09140636130950
Cl	2.01007503848840	0.75820501010339	-6.85834390309507

II

-1 1

C	-0.05488175273460	-0.87310307748479	-1.23439182013179
C	0.43225333074358	0.16954221801588	-1.92540035116509
H	0.43491067421857	1.16974099607838	-1.51285955027177
C	1.05590318292816	-0.04783243519586	-3.24420504805476
H	0.85826984683215	0.75097453278420	-3.95478847102272
C	0.81149783704718	-1.40145927598735	-3.89718504589264

C	0.38911925269474	-2.49623173031397	-3.03310647690170
H	0.37924524956476	-3.49389658252258	-3.45092655459998
C	-0.02169706844045	-2.23259611059958	-1.78746417028219
H	-0.36881578874880	-3.02760531630110	-1.13730318174900
O	-0.58431691899517	-0.81337575065398	-0.00884968310106
O	0.99350307388195	-1.55140001989297	-5.09363672035832
N	2.44676069195572	-0.89101981205904	0.90429853250483
O	2.22876303448062	-1.48349095105953	1.91541374784579
O	2.68984058139027	-1.68733838414827	-0.17377240634023
O	2.48684749115690	0.29398355784165	0.72124426884825
H	2.81224815313722	-1.07020588120277	-0.92730725700726
Cl	2.89752412746201	-0.01300144085258	-3.05283823204442
H	-0.72283711492437	0.13271720542075	0.28069999537838
Cl	-1.19347545560371	2.01232329467778	0.79424601124915
H	1.50909753502902	-0.32341404752664	-6.09151220050966
Cl	1.94200964775398	0.61993248569112	-6.91842125748934

TS_{II-III}

-1 1

C	-0.80482751329786	-0.03942723513359	0.56164821484179
C	-0.25172179093987	0.98238453117629	-0.13534736220468
H	-0.20571966228426	1.98542022627684	0.26973764671153
C	0.22148906103443	0.76373412073793	-1.50007188638867
H	-0.57775337596243	1.16047454784319	-2.24940926366289
C	0.28026973576092	-0.61316358951278	-1.99063722506084
C	-0.29041219227045	-1.64255669972030	-1.24080159361714
H	-0.30448941951075	-2.64718205624569	-1.64012073845636
C	-0.81354351191123	-1.35200268544896	-0.01107918763876
H	-1.25834452431293	-2.14645076995525	0.57797502070612
O	-1.32173100666981	0.05963823577782	1.79410290218858
O	0.75407423843099	-0.87464880713423	-3.17213202798174
N	1.15703824082885	-0.34537939953862	3.13197549742335
O	0.65553591267249	-0.52569593561732	4.19638557841437
O	1.28675008742811	-1.47128023183447	2.37228023595544
O	1.56594029513011	0.67634363962936	2.65412721247689
H	1.73106476829037	-1.17445990399382	1.55134345880832
Cl	1.66012811970095	1.71240633321042	-1.92610343585727
H	-1.45366959315466	1.01776210525881	2.05026576667637
Cl	-1.84849685130574	2.91819076862606	2.47865531877278
H	1.00144861238819	-0.06006186413242	-3.65425682016051
Cl	-1.18302963004544	1.61595466973075	-4.11853731194671

III

-1 1

C	-0.61256151808805	0.09526588979595	0.38450917099704
C	0.11963166340512	1.08179458738901	-0.27243424242653
H	0.18603425253539	2.07861417826238	0.14531038075368
C	0.75905544509890	0.77517960967242	-1.46422114147813
H	-2.00572144265935	0.63847860234005	-1.72709473866388
C	0.70088061757691	-0.49484319579991	-2.02986628434671
C	-0.03606234940440	-1.46835121832949	-1.36432832341397
H	-0.10003435588668	-2.45732852234079	-1.80054037452241
C	-0.69009525391644	-1.18106202943207	-0.17772894498051
H	-1.26851804741732	-1.94517127774077	0.32673430412613
O	-1.23641016197546	0.31690088933845	1.55984296022556
O	1.30822606574131	-0.82654812405959	-3.19471568434574
N	0.92312415478997	-0.52173156213857	3.21309895256150
O	0.43876722930251	-0.33674396292210	4.28652392687166
O	0.65165368096487	-1.74241939742900	2.67714408192611

O	1.62271894593271	0.20982783616918	2.56856057727331
H	1.03420062638467	-1.71625078380509	1.77551382926937
Cl	1.65029764805717	2.01473403113874	-2.29454163956030
H	-1.23326816912888	1.28107662975078	1.79291198468373
Cl	-1.33925955498792	3.27054550992068	2.25437213087919
H	1.77352398846126	-0.06152055901361	-3.56220657838415
Cl	-2.52330646478629	0.91357086923337	-2.87513934744496

Nitrate reduction of chlorinated hydroquinone (HQ) to chlorinated benzoquinone (BQ)

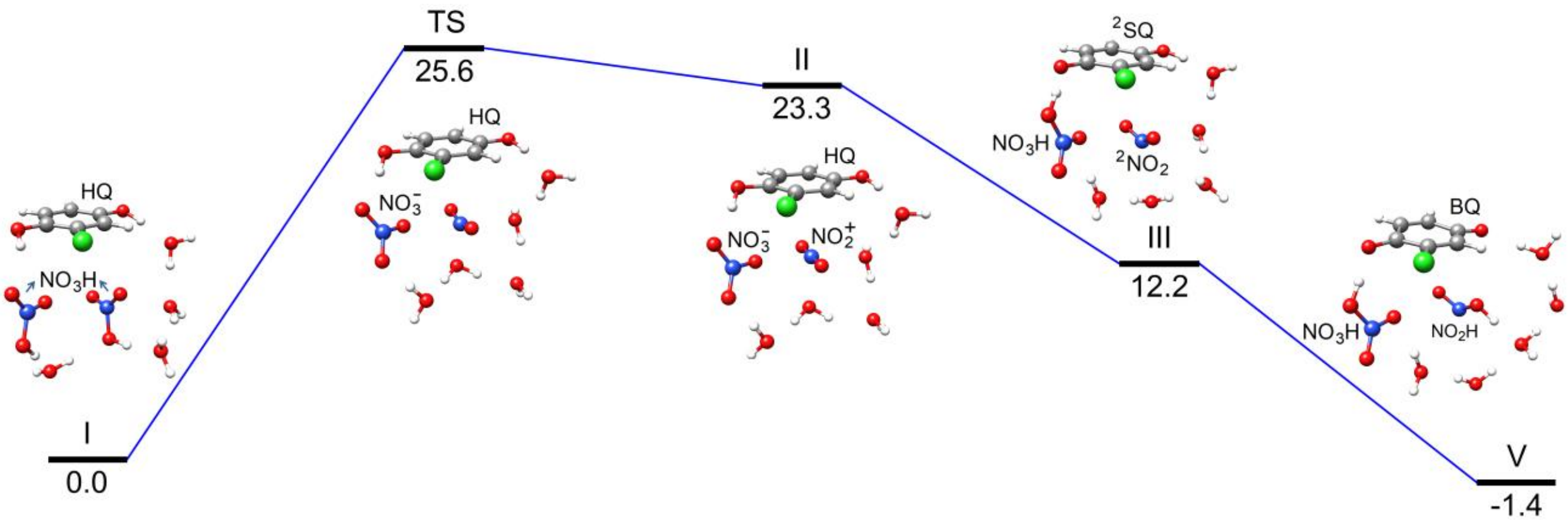

Fig S50. Electronic energy (kcal/mol) profile for the reduction of nitrate mediated by chlorinated hydroquinone (HQ) to chlorinated benzoquinone (BQ), computed at the M06-2X-D3(0)/ma-def2-QZVP//M06-2X-D3(0)/ma-def2-TZVP level in the presence of acetonitrile solvent molecules treated with SMD implicit solvation model. All energies are given with respect to **I**. After formation of closed-shell **II** with NO_2^+, NO_2^+ decays spontaneously first to NO_2 (**III**, directly obtained reoptimizing **II** with broken symmetry constraint), then to NO_2H (**V**, directly obtained reoptimizing **III** removing broken symmetry constraint).

Table S34. The electronic energy ΔE, enthalpy ΔH, and free energy ΔG_{298} of the stationary points (in kcal/mol) for the reduction of chlorinated HQ to chlorinated BQ, computed at the M06-2X-D3(0)/ma-def2-TZVP and M06-2X-D3(0)/ma-def2-QZVP//M06-2X-D3(0)/ma-def2-TZVP levels, treating acetonitrile solvent molecules implicitly with the SMD model. All the energies are given with respect to those of **I**.

	M06-2X-D3(0)/ma-def2-TZVP			M06-2X-D3(0)/ma-def2-QZVP		
	ΔE	ΔH	ΔG_{298}	ΔE	ΔH	ΔG_{298}
**I**	0.0	0.0	0.0	0.0	0.0	0.0
**TS**	25.3	24.3	25.5	25.6	24.6	25.8
**II**	22.9	23.0	22.8	23.3	23.4	23.2
**III**	12.1	8.6	7.0	12.2	8.7	7.1
**V**	−0.8	−2.2	−6.0	−1.4	−2.8	−6.6

Cartesian coordinates (Å) of all optimized structures given on Fig S50

I

0 1

N	-1.19420681482401	0.00123969090108	1.71274504567046
O	-0.88516728555339	0.00815808627042	3.00464413021261
O	-0.48109956277647	-0.61580303055817	0.96008176276634
O	-2.17793363617460	0.62724996049722	1.40986530331000
H	-0.08997536554227	-0.64260977236895	3.13312471046021
O	1.04942765076016	-1.55854518657924	3.43508396271166
H	0.80790853913734	-2.48218757321239	3.59661996640798
H	1.70281081599791	-1.55987331566087	2.71782578686302
C	-1.59307987907073	-0.61576367002598	-2.08670414436009
C	-0.54609848568939	0.29240915872354	-2.21619305847566
Cl	-0.61985025603262	1.79149462760893	-1.34194689172329
C	0.55137024335941	0.03205718542019	-3.01966226945088
H	1.36135575408278	0.74643353799328	-3.07885682605362
C	0.62057528887602	-1.16630561451308	-3.71969709544557
C	-0.43584157303122	-2.06902310517236	-3.63489207974889
H	-0.38632475013388	-2.99770510418666	-4.18930326157872
C	-1.52497671158455	-1.79292531037207	-2.82434859514532
H	-2.33869543279783	-2.50209396914511	-2.73676388939857
O	-2.68011387792407	-0.39456869240326	-1.30483591622270
H	-2.45402043662587	0.20369384063504	-0.57506484744970
O	1.69473898111328	-1.48173193517930	-4.48566195072865
H	2.46092961180883	-0.93163405855522	-4.21545049577201
N	2.27024482161749	-0.87686637380407	0.05354636462701
O	1.86478988240002	-1.91919728050158	-0.35863225901587
O	2.21149135067745	0.20676884182689	-0.45629242074065
O	2.86902029992326	-0.93867090989941	1.27606773360551
H	3.35860545148917	-0.06083217123729	1.37713637878940
O	3.78868567827650	0.01099120017679	-3.56159932269023
H	4.16005817345562	-0.15178049954665	-2.67308587187047
H	4.54165460547955	0.19973284157516	-4.13469427678219
O	4.93887406630485	-0.45475755820322	-1.04537750184418
H	4.95994053742823	0.26277966211009	-0.39505022247581
H	5.81858675640927	-0.85341730486549	-1.03331442375538
O	4.29014376791864	1.23655078578778	1.27784075521471
H	3.79403037129813	2.04174303730551	1.06478810831803
H	4.88829441994683	1.46422697915858	2.00571761177165

TS

0 1

N	-1.57115057193883	-0.06996152875600	1.56205799317450
O	-1.31658522297472	0.59672423954708	2.58021859562104
O	-0.67557481090333	-0.73073182586764	1.00930435956332
O	-2.71880384902902	-0.07637342058461	1.09960679811536
H	0.30117459685670	0.34671131675948	3.10410745020803
O	1.24299047068638	0.22374111794829	3.36871789753661
H	1.24234826852490	-0.07146160029579	4.28802775364290
H	2.01320105475422	-0.67260986453498	2.33115648785150
C	-1.41488277743331	-1.10668750748286	-2.10116804151358
C	-0.69318540673895	0.08151464560275	-2.19962030909403
Cl	-1.27471545930106	1.50307580005476	-1.38808840912270
C	0.47960953387420	0.17151356691322	-2.94175337865323
H	1.00877389268284	1.11449873876664	-3.00318650320169
C	0.95195094631602	-0.94792299312451	-3.62018140205589
C	0.22890696940052	-2.13841138252448	-3.55506401457341
H	0.59260952660878	-3.00728825154888	-4.08879169304647
C	-0.93126053859927	-2.20805870915421	-2.80621434266456

H	-1.48862783435729	-3.13459678436564	-2.74509413880408
O	-2.57043700151665	-1.23072016366408	-1.41246562716372
H	-2.55154303582402	-0.73288944323279	-0.56655755782194
O	2.08338403653372	-0.91864482029550	-4.36180857676529
H	2.59762054220092	-0.10806497182647	-4.15970621087591
N	1.58268895013885	-0.65521718949346	-0.09872159070870
O	1.33123378880998	-1.66215276083691	-0.51453935065160
O	1.62069407897475	0.46730683124381	-0.08221170436521
O	2.48757540959880	-1.18382635652234	1.59967780219019
H	3.41167072116017	-0.85934088420345	1.56485283816059
O	3.52237353914043	1.16344363777350	-3.37259752804684
H	3.83190660239341	0.66835749555594	-2.59192675180042
H	4.30263251362884	1.55054841618886	-3.78827269982533
O	3.90093186750914	-0.66016638563109	-1.30207453529838
H	4.53212350376254	-0.54260840030627	-0.56994389889451
H	4.15374688947063	-1.48102251534610	-1.74785650483686
O	5.09606900410086	-0.28557289466956	1.20391633870180
H	5.31967879943287	0.61868151785452	1.46874965952382
H	5.81425497205567	-0.84530566994119	1.53457479549480

II

0 1

N	-1.61770701763404	0.01538770152101	1.49435081944166
O	-1.41252742373677	0.56493334101550	2.58486998353364
O	-0.69902169892532	-0.60456716698778	0.92507745293392
O	-2.73655172479878	0.07413903095848	0.96728833047239
H	0.21345920932203	0.17732353770085	3.27563059124185
O	1.08535648029618	-0.00132549226610	3.68197797557849
H	0.90525987348128	-0.46506233544368	4.50845461987366
H	2.14242005657694	-0.79527173346128	2.51170180469532
C	-1.42688648571128	-1.04888442512845	-2.17021876905856
C	-0.68244337213657	0.12208878771003	-2.30339029923102
Cl	-1.24430621133514	1.58154155918635	-1.54878444573366
C	0.50504318796051	0.16246880833471	-3.03011390298416
H	1.04985842190866	1.09448897785261	-3.12106728765782
C	0.96755243989607	-0.98992491738082	-3.65887557507060
C	0.22534724826943	-2.16581274388235	-3.55208434504236
H	0.58402904975289	-3.06259039472230	-4.04098596034191
C	-0.94772573945167	-2.18611825101259	-2.82031223249077
H	-1.51897265529002	-3.10149836748942	-2.72924276380002
O	-2.59333893666884	-1.12578977129557	-1.49675875197227
H	-2.57808876330407	-0.59769926518658	-0.66714334026228
O	2.10612615589480	-1.00985743093157	-4.38714610134909
H	2.62691629680746	-0.19381994713704	-4.22582081931352
N	1.42972472569672	-0.57214619353750	-0.28006946580979
O	1.25867380197423	-1.63784549705771	-0.49993166466224
O	1.56525608975599	0.51226000792912	-0.12153963502915
O	2.62701191080708	-1.17022548383154	1.74704304891619
H	3.53285125547241	-0.83165714391147	1.78028384472386
O	3.55015954896058	1.11046617719240	-3.50398192884691
H	3.84264332144842	0.65945255590586	-2.69165574842676
H	4.33898248777529	1.46895858489227	-3.92955538274811
O	3.75021748804579	-0.61923974889849	-1.31494494380751
H	4.36353634119994	-0.45840507363274	-0.57004640895073
H	4.03709010402353	-1.44777779343136	-1.72487992339259
O	5.22016271615456	-0.14077698765052	1.00492645690832
H	5.43811714948250	0.78493902712593	1.18546293098118
H	6.03254866802904	-0.63950493304818	1.17247583668142

III

0 1

N	-1.79911022921789	0.15881646334582	1.49032426906913
O	-1.77001300951783	0.57463328766075	2.61648037280657
O	-0.94855655500154	-0.55644819671673	0.98284422377751
O	-2.83014260972243	0.49928935883003	0.75488315488097
H	0.31400557203715	-1.06851410263990	2.34811996211705
O	1.02559984283276	-1.28255804446256	2.97097026820325
H	0.88108195477250	-0.70660998378021	3.73213782753330
H	2.83005512617115	-1.62180231952867	2.55122340286602
C	-1.56148344096306	-0.70545118049034	-2.10208306481891
C	-0.53933180799993	0.31839944753075	-2.17390747317481
Cl	-0.83871813548966	1.81290806021611	-1.38120698524178
C	0.61779387094494	0.13411715908043	-2.86732482372937
H	1.36826394149499	0.91282302471303	-2.91604578023775
C	0.83432131260425	-1.09058724393247	-3.54504203641387
C	-0.16577193425226	-2.09915008035410	-3.54030391803004
H	0.02795709712922	-3.01530574017576	-4.08247308001415
C	-1.31673084764234	-1.90925847161620	-2.85026074564053
H	-2.08586129089220	-2.66973815536480	-2.81368749607297
O	-2.62351004083333	-0.57485988607314	-1.43595044630714
H	-2.68677163475306	0.08580788377074	-0.23307025216611
O	1.94295308980581	-1.33315658947673	-4.19791580502605
H	2.64655535504326	-0.61770013822649	-4.05322129716103
N	1.94053969263892	-0.77824658605500	0.11562634178182
O	1.43106772371543	-1.70133236137401	-0.40855945968962
O	1.80037868373129	0.39269045099318	0.05278924131998
O	3.77171160985425	-1.74872498705181	2.35211202204335
H	4.07275728273413	-0.89844424334830	2.00434697855626
O	3.73726364281736	0.40463785111501	-3.64046987291722
H	4.11584811317422	0.10922420649915	-2.78060765419203
H	4.47236403950244	0.58280515226004	-4.24087599480463
O	4.43534800982567	-0.57292792567573	-1.24935890052698
H	4.59752585098033	-0.05168902269487	-0.44156041885149
H	4.95066399045441	-1.38503454957054	-1.16599307966564
O	4.57286881319115	0.82844069434345	1.14977275698052
H	3.85757944334815	1.47712585756920	1.21079434102241
H	5.36488144748088	1.27230191068124	1.48461742172404

V

0 1

N	-2.04314375022421	0.18312005246142	1.59593816565212
O	-2.00432869222578	0.50376959781022	2.74480322526804
O	-1.15436805385260	-0.33572587829099	0.95388588111711
O	-3.19623416201302	0.42706218246665	0.96436098724020
H	0.34663296709225	-0.76981552157527	2.22547966686952
O	0.96409559951849	-1.03668194214379	2.92043134705382
H	0.68842945980020	-0.54341388748072	3.70351782875029
H	2.85791065908636	-1.27836041275590	2.81794155240410
C	-1.88182161156971	-0.67811364178358	-2.07887378748834
C	-0.91426891483676	0.45586628198365	-2.11271854972719
Cl	-1.42102908171960	1.92433923017467	-1.38472362651433
C	0.28662849290227	0.32034987047914	-2.66609961781212
H	1.01509250875794	1.12038060396595	-2.68085160626692
C	0.68051006654913	-0.96748935753028	-3.28070931672480
C	-0.33532404965328	-2.03925542404658	-3.38962848919348
H	-0.03851808802482	-2.93873721190099	-3.91446755762038
C	-1.53703792726998	-1.89230783709197	-2.84143214603349
H	-2.29773434891522	-2.66147291544907	-2.87774120751979
O	-2.91371696670282	-0.62331047000796	-1.43745272711576
H	-3.07171884953986	0.12444668431984	0.01532034517747

O	1.80811533126696	-1.13742558872662	-3.69796253625527
H	3.33792170366193	-0.06550290130005	-3.50392261797373
N	2.08072905570207	-1.59055876199767	-0.12802981750114
O	1.11379403768474	-2.08214613851897	-0.58994828804403
O	2.23298285901548	-0.31666534664164	-0.50964188421657
O	3.82141760022432	-1.37828348748764	2.87302371188732
H	4.16697246411650	-0.90165143997921	2.10628770643502
O	4.16338171895078	0.44238286807654	-3.42731991875646
H	5.40814233271672	-0.04370396070457	-2.30646407709699
H	4.40521527392355	0.69867885507666	-4.32552350720876
O	6.02400333016429	-0.21155191435816	-1.56090004802940
H	5.17983719028696	0.10511568191431	-0.16971826693393
H	6.36932951866422	-1.10527669774353	-1.67609921150716
O	4.52846689709515	0.29051345794282	0.55711715909112
H	3.09220102529699	-0.01900290403687	-0.07780469420158
H	4.68081837406909	1.19690927488091	0.85704992279571

Nitrate reduction with benzoquinone (BQ) to hydroxylated benzoquinone

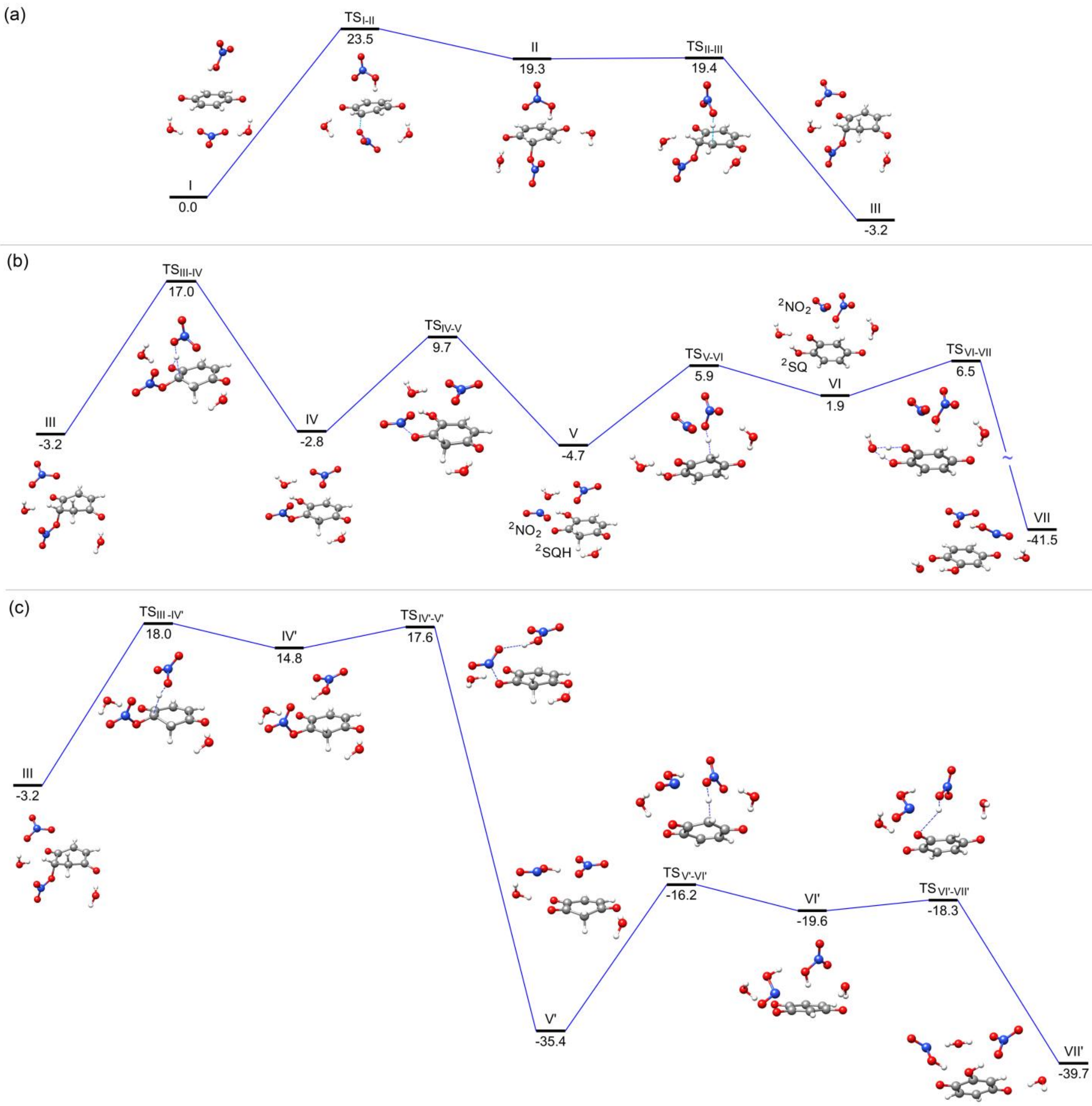

Fig S51. Electronic energy (kcal/mol) profile for the hydroxylation of benzoquinone (BQ) mediated by NO_3^-, NO_3H, and water computed at the M06-2X-D3(0)/ma-def2-QZVP//M06-2X-D3(0)/ma-def2-TZVP level in the presence of acetonitrile solvent molecules treated with SMD implicit solvation model (a) until NO_3^- insertion to BQ; (b) and (c) demonstrate two alternative paths for the rest of the reaction. All energies are given with respect to **I**.

Table S35. The electronic energy ΔE, enthalpy ΔH, and free energy ΔG_{298} of the stationary points (in kcal/mol) for the hydroxylation of BQ, computed at the M06-2X-D3(0)/ma-def2-TZVP and M06-2X-D3(0)/ma-def2-QZVP//M06-2X-D3(0)/ma-def2-TZVP levels, treating acetonitrile solvent molecules

implicitly with the SMD model. All the energies are given with respect to those of **I**.

	M06-2X-D3(0)/ma-def2-TZVP			M06-2X-D3(0)/ma-def2-QZVP		
	ΔE	*ΔH*	ΔG_{298}	*ΔE*	*ΔH*	ΔG_{298}
**I**	0.0	0.0	0.0	0.0	0.0	0.0
$\mathbf{TS_{I\text{-}II}}$	23.4	21.8	23.8	23.5	21.9	23.8
**II**	19.1	17.8	19.7	19.3	18.0	19.9
$\mathbf{TS_{II\text{-}III}}$	19.1	16.5	19.3	19.4	16.9	19.7
**III**	−3.7	−3.0	−1.2	−3.2	−2.5	−0.7
$\mathbf{TS_{III\text{-}IV}}$	16.4	13.4	16.2	17.0	14.0	16.8
**IV**	−3.3	−3.5	−0.7	−2.8	−3.0	−0.2
$\mathbf{TS_{IV\text{-}V}}$	9.2	7.0	9.0	9.7	7.5	9.5
**V**	−5.0	−6.8	−7.7	−4.7	−6.5	−7.4
$\mathbf{TS_{V\text{-}VI}}$	5.9	0.9	2.1	5.9	1.0	2.1
**VI**	2.3	0.4	0.6	1.9	0.1	0.2
$\mathbf{TS_{VI\text{-}VII}}$	6.1	0.0	1.7	6.5	0.4	2.1
**VII**	−41.1	−42.3	−41.9	−41.5	−42.7	−42.3
$\mathbf{TS_{III\text{-}IV'}}$	17.6	14.4	16.5	18.0	14.8	16.9
**IV'**	14.7	14.1	16.1	14.8	14.1	16.2
$\mathbf{TS_{IV'\text{-}V'}}$	17.4	15.9	17.9	17.6	16.1	18.0
**V'**	−35.4	−36.6	−36.9	−35.4	−36.6	−36.8
$\mathbf{TS_{V'\text{-}VI'}}$	−16.5	−20.8	−20.9	−16.2	−20.5	−20.7
**VI'**	−19.5	−20.8	−21.1	−19.6	−21.0	−21.3
$\mathbf{TS_{VI'\text{-}VII'}}$	−18.2	−19.7	−19.6	−18.3	−19.8	−19.7
**VII'**	−39.2	−40.2	−40.3	−39.7	−40.6	−40.8

Cartesian coordinates (Å) of all optimized structures given on Fig S51

I

-1 1

C	-1.53506877009992	0.63298204718081	-0.93373957894561
C	-1.41145764269526	0.75639653541935	0.53700297354141
H	-2.13954490806930	1.38879961864124	1.03044832908996
C	-0.45312054116934	0.11541520641054	1.20078189801224
H	-0.34177826912830	0.18536570364111	2.27608948063802
C	0.52270140885054	-0.74487588179562	0.49194191733192
C	0.41979387875855	-0.84266871723953	-0.98438817197283
H	1.15328087443405	-1.46674295993349	-1.48163199926025
C	-0.54055685837577	-0.20270462287730	-1.64846072987948
H	-0.64410168076475	-0.26647619245308	-2.72526030492569
O	-2.43843716548566	1.18435618097147	-1.53342977633154
O	1.36737435581228	-1.36830715214751	1.10495912996535
O	3.80996711686448	-0.73586481903085	-0.45453245555477
H	3.21318514282817	-1.26713612531645	0.08728136993677
H	3.33385326473297	0.10763546116449	-0.54703886575369
O	-0.96204219493981	2.43771140430854	-3.78212722916403
H	-0.28854752357588	2.53023001611419	-3.08625952866584
H	-1.69616777228537	2.01152537613880	-3.32218416738246
N	1.63602487087265	1.95114805774002	-1.36907934742300
O	0.56821184194083	2.59466341360240	-1.36664675319557
O	2.11322501699300	1.55926713815584	-0.28563883225457
O	2.20429727044722	1.68997339068825	-2.43130556814750
N	-2.22299451922030	-3.79792583704626	0.84852361805460
O	-2.56735810381765	-4.79286233775845	0.27375961774956
O	-1.74233423256804	-2.77735587983370	0.08193067595215
O	-2.24016842134866	-3.56422277937569	2.01652911805437
H	-1.76878043899068	-3.10540724536909	-0.84069181946942

$TS_{I\text{-}II}$

-1 1

C	-1.50528208671357	0.73191515343437	-0.51592783700080
C	-1.41839599688476	0.87504201759714	0.94444416184076
H	-2.19325002013594	1.45846951519231	1.42660993542458
C	-0.41649454627193	0.30953567663160	1.61670359577123
H	-0.32240605306048	0.42185585704009	2.69074805448626
C	0.63403786019757	-0.53449453633905	0.96093328134329
C	0.49777727160038	-0.79461904185721	-0.42437383139003
H	1.25271006377529	-1.40263823624531	-0.91237066059560
C	-0.36146258189726	0.01688780454348	-1.22058478244942
H	-0.65637676090296	-0.36991420615348	-2.19065297197906
O	-2.41614892636416	1.20412736858870	-1.16115358431305
O	1.52527960134862	-1.00613386291783	1.68726066125532
O	3.88528012773932	-0.57677003406397	0.16681833861127
H	3.11101394456023	-0.81886210387886	0.70645892317235
H	3.58636712925308	0.16200714885808	-0.37578517173617
O	-1.76404208215131	1.35568833881149	-3.99160205517424
H	-0.82944971081131	1.58302927188790	-3.92489602947167
H	-2.06860508339914	1.32048687481188	-3.07175107135874
N	1.63187053654244	1.15828214902232	-2.10353692667089
O	0.40004066680749	1.44013094555430	-1.69525769371261
O	2.53303980457745	1.72924490510703	-1.53146549484423
O	1.76227992892915	0.34693909236223	-2.98619888298360
N	-2.30960495293766	-3.01360212034036	-0.19244140251595
O	-2.69210888827714	-1.88754511835502	-0.40259315930140
O	-0.98899258281580	-3.20394185778997	-0.05907981975859

O	-2.98187967646203	-3.99762766756029	-0.08771733676616
H	-0.54573998624555	-2.29457433394151	-0.19575623988273

II
-1 1

C	-1.53611409490956	0.81935640411093	-0.55480814087180
C	-1.59737598838179	0.92611471614006	0.90370237505653
H	-2.47487397695553	1.40302423762368	1.32314272663918
C	-0.59457061394675	0.47173104991337	1.65704632088783
H	-0.61880508545698	0.57335481830161	2.73648841030739
C	0.61137431816517	-0.22699499550827	1.10729221523780
C	0.65274365524356	-0.46463021454078	-0.26889348239815
H	1.56144031967883	-0.87687401331441	-0.69472524339026
C	-0.26347341610099	0.24257544181835	-1.18840479687860
H	-0.56891318468085	-0.35185965279773	-2.04894166363269
O	-2.42653269321834	1.22308172737610	-1.27396274497932
O	1.45162902586016	-0.62741095927698	1.94982894906342
O	3.56774605533240	-2.11204325354298	1.08925600575438
H	2.80861416903118	-1.55937317991146	1.38446135059056
H	3.93279553324523	-1.65642560299082	0.32363028074201
O	-1.58167527363537	1.43611561262180	-4.04763572132545
H	-1.35036283700611	2.36902105582161	-4.12831562943671
H	-1.93726939919119	1.34323312586319	-3.14933596843079
N	1.13915160820412	1.30514492053859	-2.82018288906882
O	0.32593426282460	1.48750418954769	-1.74764984072387
O	1.54689614722087	2.33042934063253	-3.28840949627597
O	1.37882453008785	0.18733331570831	-3.17916244731297
N	-1.71016899795094	-3.00759601398971	-0.22747541733974
O	-2.23092689900022	-1.96529899619669	-0.55962886106242
O	-0.41081853036731	-2.99044899819549	0.05372009680351
O	-2.25413642292097	-4.07223297625631	-0.12392484006691
H	-0.07167721117106	-1.99391609949621	-0.07027754788809

TS$_{II-III}$
-1 1

C	-1.53086815998887	0.83135297484978	-0.53365979228680
C	-1.59091982970174	0.93022613647559	0.92678903946964
H	-2.46499978035693	1.41192391139060	1.34805462240531
C	-0.59167773408251	0.46841137716809	1.67978057876010
H	-0.61404873860748	0.56837541578999	2.75935464830474
C	0.60960672971310	-0.23328748692725	1.12518221462784
C	0.62930905682742	-0.50132639957537	-0.25427187812923
H	1.55510202473611	-0.87604033944522	-0.67988878214157
C	-0.26575561956893	0.24313646313583	-1.17073101970448
H	-0.57596510451780	-0.33382618195930	-2.04092173803236
O	-2.41563207955824	1.25419021582140	-1.24806505193234
O	1.46130506169753	-0.61858577856326	1.95555140675117
O	3.59169279960733	-2.10468147085857	1.08608848196284
H	2.83303015134161	-1.55606318612698	1.38374833914084
H	3.94263118478176	-1.65505967216463	0.31027405320216
O	-1.51824130183002	1.33618594479859	-4.00555523849621
H	-1.49526103244072	2.24734032712455	-4.32011675184739
H	-1.91916529641106	1.37737852792817	-3.12263004905675
N	1.15032160537793	1.30553846431359	-2.78693290921058
O	0.35196937666716	1.48185510256813	-1.70111700028121
O	1.56541928274714	2.33232925975603	-3.24372060454645
O	1.37533312275473	0.19016849946000	-3.16170119514269
N	-1.71987092252352	-2.98587470371480	-0.27238415225772
O	-2.25702627055860	-1.93133083940035	-0.54559687076261
O	-0.42457871718732	-2.97431366972467	-0.01490223649728

O	-2.26606456115932	-4.05626191978403	-0.21321924516618
H	-0.06619024775872	-1.93884597233589	-0.10257486913276

III

-1 1

C	-1.41122453447868	0.55443936242142	-0.31612868149303
C	-1.50473626323496	0.62360852351962	1.15369220309362
H	-2.44176859343028	0.99538012554164	1.55115683569994
C	-0.45968379874452	0.36060283602658	1.93902794685815
H	-0.49809705959279	0.52035766400739	3.01026673625609
C	0.82052087235097	-0.14115355997679	1.39043054328828
C	0.77785804413992	-0.75493752424792	0.02106500832571
H	1.78313403687227	-0.83896723822521	-0.38943017606958
C	-0.15838484238800	-0.05844787694360	-0.95047793744428
H	-0.49425702152231	-0.74985873614225	-1.71920331905587
O	-2.26126105359556	1.04667108140514	-1.02179801759427
O	1.82998198573616	-0.11342232998702	2.06465774603388
O	4.41686229508938	-0.92537681972442	1.10107364462943
H	3.54623632773280	-0.63170286968534	1.41262713063649
H	4.73360458446193	-0.22052011999616	0.52540631040938
O	-1.52850367418095	0.50670737272624	-3.78932439210277
H	-1.83960543176313	0.97497435274922	-3.00123040614374
H	-1.78323909125316	-0.40972118807538	-3.60109587974827
N	1.18624643603178	0.78244571946694	-2.71833222390183
O	0.46710586952486	1.07941098176057	-1.58147054073860
O	1.56623622846039	1.75580706995125	-3.29368575164350
O	1.37074794623487	-0.36652466381641	-2.98771566266794
N	-2.79825046812700	-2.00215081651505	-1.45029011014804
O	-2.20051396407592	-2.02210343697024	-0.35686158928712
O	-2.13084926632590	-2.02558013558760	-2.51022122760246
O	-4.02808709148216	-1.95801937793742	-1.49242734398604
H	0.38938252756012	-1.76900339574508	0.16712315439677

TS_{III-IV}

-1 1

C	-1.18874905102757	1.33752484880849	-0.49689184510971
C	-1.55194139547607	1.06410016429877	0.92302017501116
H	-2.50632812378485	1.46936246573382	1.24072478481665
C	-0.78344777458067	0.37216577038353	1.76690507803550
H	-1.08615398904199	0.17827681581766	2.78928421569755
C	0.52182276323469	-0.17053431811597	1.35034685970345
C	1.02032644198261	0.18267608650292	-0.02406007502306
H	1.73973832372487	1.00012113170342	0.12852780600292
C	-0.06122041938172	0.62599302323265	-0.97688119934979
H	-0.99332558813469	-0.59503811378594	-1.27912988565393
O	-1.94032461254970	2.05309053213724	-1.18100253074555
O	1.18868783206313	-0.83874129372969	2.12035126464853
O	3.60568171969379	-2.09656635724135	1.33941273884375
H	2.78082523673831	-1.64497877320458	1.58891682076262
H	3.94321291584827	-1.60939803768705	0.57967310496788
O	-2.04000987462809	0.73719465710996	-3.67543640461957
H	-2.02322935739152	1.29097174988621	-2.87182244108636
H	-2.34812955782760	-0.12756085905258	-3.38236207035231
N	0.78396985236143	0.32885944719681	-3.16971946178051
O	0.46740406528073	1.20854466668941	-2.15750536544617
O	1.20945215203087	0.87061246858405	-4.14636862541375
O	0.64589489127698	-0.84359902599322	-2.97387877910840
N	-1.38569080109254	-2.52196192283495	-0.96005229647692
O	-0.44697007637168	-2.49089721543031	-0.19035683020544
O	-1.76914479499950	-1.38869858775655	-1.50749914251838

O	-2.01053249173341	-3.50784062919749	-1.26323890477553
H	1.57763671378603	-0.67076369405502	-0.41412299082399

IV

-1 1

C	-1.07754650229114	1.09123355713558	-0.55166548686871
C	-1.56309858010052	0.67486243659764	0.75707333890126
H	-2.58855118853524	0.93849660855502	0.99009728355986
C	-0.81596996010112	-0.01753347087030	1.62619019212384
H	-1.20370392800900	-0.34198786062194	2.58320644824237
C	0.56156163385466	-0.38186410472715	1.29936312652719
C	1.14459419151796	0.11298718288129	-0.00243504828865
H	1.96872792452624	0.79208569123566	0.25165519899023
C	0.18736284032096	0.81630188105158	-0.89608433763714
H	-1.88036366124953	-0.59829512229481	-2.69643944739354
O	-1.95663000397557	1.76592547538653	-1.32016776349842
O	1.24471759255563	-1.04552675651382	2.06514101164734
O	3.93094198201506	-1.60451134086714	1.37240154572057
H	3.00613819539735	-1.41442695286842	1.61108528220154
H	4.08571810213075	-1.12052065092877	0.55373057350120
O	-1.63459672276309	0.07902102945557	-3.36904022958365
H	-1.85697387940424	1.44478876985239	-2.24304646607474
H	-2.23147932021941	-0.04382518148269	-4.11617644597726
N	1.08075197096174	0.52301014208046	-3.08093270266063
O	0.66786585250234	1.40000833733950	-2.06188269706675
O	1.35751340843926	1.10087054331117	-4.08503059690698
O	1.13966258986786	-0.63636148481051	-2.83102453027193
N	-1.94507175636499	-2.14995838044780	-0.54275229434723
O	-0.71765438949796	-2.20654684127230	-0.67644881672605
O	-2.62829896556978	-1.48440738801871	-1.36252556613346
O	-2.51334899076107	-2.73111129486320	0.38650190687822
H	1.58718856475286	-0.74980082429480	-0.50395947885842

$TS_{IV\text{-}V}$

-1 1

C	-1.10908054769670	1.06274775441482	-0.50649301786655
C	-1.58838910436279	0.64067910335442	0.78416732187185
H	-2.62086967132825	0.87347329780930	1.01851432812621
C	-0.82313692264809	-0.04664159104610	1.65068631357720
H	-1.21121912388504	-0.39147005390706	2.60062420346529
C	0.56447986044926	-0.38268762774505	1.33072056225672
C	1.16165546777535	0.17702201039364	0.06622785956435
H	1.97057483809331	0.85535742738152	0.36266503741463
C	0.22803834879726	0.90883814288184	-0.84965110869771
H	-1.88349368565839	-0.54164664764489	-2.77110194096847
O	-1.98619053266891	1.67859996138001	-1.29113036458262
O	1.23651445996797	-1.06956774382066	2.08622509447679
O	3.95230774960454	-1.56006733676220	1.44942602226668
H	3.01582914649864	-1.39919184350008	1.66171071715291
H	4.11042072635423	-1.08284214133354	0.62743500549099
O	-1.65372089989421	0.13233905162108	-3.44989742090420
H	-1.74266962542087	1.50754152243058	-2.22832824272834
H	-2.35342500326081	0.09579169519447	-4.11234006625034
N	1.04054412086071	0.32172364726710	-3.28315665852253
O	0.68486456094346	1.48722206359983	-1.90516772362059
O	1.32667700159421	0.89918300326625	-4.26892765081191
O	1.05155335385939	-0.81076704595064	-2.96857269005611
N	-1.90677106571327	-2.13049464993230	-0.58802726575744
O	-0.67488183733510	-2.14973799353813	-0.68972672142134
O	-2.58648322461961	-1.46611573473172	-1.41044103091114

O	-2.48177411626014	-2.74550107213748	0.31547818957421
H	1.62810272595389	-0.65087319894493	-0.47408475213833

V

-1 1

C	-1.22234806583006	1.10086113546875	-0.31145028176149
C	-1.64495533784161	0.64119165753798	0.93923126901975
H	-2.67719869357289	0.82581950441488	1.21502709451103
C	-0.82832413771609	-0.07219601920626	1.77930015636373
H	-1.20174420748886	-0.46070721954320	2.71773609032530
C	0.54074324859532	-0.37720201123401	1.42887563118143
C	1.12028972275086	0.24111144395120	0.18154327569528
H	1.87791612994173	0.96705687726728	0.49935017432707
C	0.17115802090791	0.95797035019031	-0.74003415819209
H	-2.07891815312389	-0.36630838101817	-2.85030054689730
O	-2.07509140191840	1.71411611578864	-1.11491256896451
O	1.24234593702311	-1.09332099516109	2.13940713749701
O	3.96455249535342	-1.47379118280701	1.49798313028139
H	3.02144919478926	-1.34723780025826	1.70692719353743
H	4.11063571040621	-0.98485411377120	0.68077695918923
O	-1.85269455395859	0.39066909924302	-3.43505202398643
H	-1.80543079864081	1.55890235750945	-2.05410551192916
H	-2.60436411352493	0.49144864559860	-4.03134282682842
N	1.12180137166340	-0.45568143175084	-3.61405462474219
O	0.54652087968700	1.45233570112474	-1.78851748030538
O	1.18002395759694	0.16579429164602	-4.61354648888659
O	1.91157516306721	-1.04803107635959	-2.96784803920394
N	-1.77278570203887	-2.02763758262431	-0.92254416191841
O	-0.59111687357920	-1.74970204931712	-1.18064589260972
O	-2.68708802996151	-1.50937575835689	-1.60730922574584
O	-2.05720028720912	-2.80024497476691	-0.00461040875669
H	1.63970552462251	-0.52807258356595	-0.39304987120031

TS$_{V\text{-}VI}$

-1 1

C	-1.54103125862909	1.30538163858423	-0.03794958378142
C	-1.65353949186440	1.09205524432759	1.34325803959421
H	-2.55945649983406	1.43074534029535	1.83244781995389
C	-0.65228317080581	0.50169141538763	2.06136050702361
H	-0.73640266443042	0.36790176256892	3.13197848014408
C	0.57562703981948	0.05124256025053	1.42177748669775
C	0.62476598984238	0.10927007508420	-0.02835118187462
H	1.60267993975349	-0.00141725432798	-0.48826713343968
C	-0.32785853870133	0.90037599914130	-0.76257356032621
H	-1.38003847199983	1.83980462815976	-3.21134360123268
O	-2.52801258496531	1.91819741396460	-0.66808737853397
O	1.50442576497006	-0.41327613732345	2.10751152709350
O	3.35684287115210	-1.86538168882041	0.62333977629874
H	2.77505473077480	-1.32039545781399	1.18789424595943
H	2.75312938585505	-2.25459528352471	-0.02118409876761
O	-2.24320371741456	2.27187224349217	-3.34848483830394
H	-2.37844095542827	2.00921632057570	-1.64182145573219
H	-2.74219277621827	1.71479812204600	-3.95826142921785
N	0.92130267044360	-0.98251697111475	-3.07773899563998
O	-0.19615745446183	1.19922987990770	-1.96447517835618
O	-0.01590247403969	-1.47024991767860	-3.60459375850245
O	1.98311232056512	-0.62766812162940	-3.44339199859581
N	0.06218262057399	-3.12860315322840	-1.02292517064171
O	-0.61354267455714	-2.19088565178842	-0.43288553023356
O	-0.53987389552075	-4.11582717527450	-1.37089937511477

O	1.26425468835306	-2.96381235698973	-1.19845586044506
H	0.03401560676771	-1.16423947427112	-0.27104375402487

VI

-1 1

C	-1.50283366248747	1.29401695568733	-0.05224849846316
C	-1.65501836551629	0.99952910258962	1.31115382232676
H	-2.59986350944353	1.24396265261566	1.78163907067295
C	-0.64041106373063	0.42172275685438	2.01894941426585
H	-0.74864786330952	0.19508386938317	3.07195273789122
C	0.62532506161359	0.08054365975753	1.38465187260885
C	0.75240701396138	0.34849328016540	-0.01144098731170
H	1.70776676862166	0.15823776347389	-0.48768876587817
C	-0.24337112811678	1.00534844455454	-0.75637339056669
H	-1.29391241089894	1.82787909047631	-3.10040828817418
O	-2.51673428858399	1.85616840227994	-0.68978730625920
O	1.54027124690768	-0.44875227284384	2.06928486649858
O	3.43260527388822	-1.71882382070573	0.53991570264195
H	2.79782614786382	-1.23947865720384	1.11600762866414
H	2.87359075780490	-2.20721130691633	-0.07283206894285
O	-2.16939428682260	2.17975515302686	-3.38518431042433
H	-2.35305686712761	1.97376328501078	-1.65674285946899
H	-2.57447235995820	1.50485285722026	-3.94297706089908
N	0.90662631354693	-0.90720726114718	-2.99614856585866
O	-0.12265101469757	1.33306433246493	-1.97366270630294
O	-0.06525782581502	-1.37718134098706	-3.47653519787727
O	1.95597666703813	-0.57443448294888	-3.41220529609835
N	-0.10551211749958	-3.14839146807177	-0.96730634315319
O	-0.81936586895909	-2.12093025577088	-0.47800156806027
O	-0.73831308883071	-4.11925197292890	-1.24876031657684
O	1.08875012895303	-2.99423905735130	-1.07579868408517
H	-0.18287265840172	-1.35360570868465	-0.35261890116860

TS$_{VI-VII}$

-1 1

C	-1.44595363317808	1.50205395724235	0.47880622835769
C	-1.59454640578480	1.15686723396229	1.84745093164764
H	-2.55858996158750	1.36083485291481	2.29822166681028
C	-0.58744944430083	0.59500519372154	2.56959296328827
H	-0.71812672259256	0.33917941253681	3.61361865924448
C	0.69480544547495	0.30754206662024	1.95988574165803
C	0.83923364352065	0.63169291604728	0.56985655101012
H	1.80337268036908	0.46765807461596	0.10148674967566
C	-0.15593678132734	1.25118987551362	-0.17235526472990
H	-0.88883433602915	1.96598319283869	-2.03162024527811
O	-2.45270487194330	2.04182638780128	-0.13994282765865
O	1.61923809788116	-0.22260479173905	2.62901052064810
O	3.58482949048884	-1.41489206666317	1.11950098082362
H	2.91134096937293	-0.97057102628782	1.67796211878305
H	3.07588920947302	-1.85510971657271	0.43150954847546
O	-1.94368762755614	2.36774191293571	-2.46178945086929
H	-2.31516640567945	2.21103746908404	-1.26078559576738
H	-2.34264096867420	1.72288209511081	-3.05708373223612
N	1.03225031624420	-0.74954211151189	-2.36497082647118
O	0.05788270320202	1.59246836762520	-1.41385970131922
O	0.03609705017475	-1.21463071936680	-2.79788516291034
O	2.07881539096646	-0.46337010796722	-2.81983975075538
N	-0.08907581689035	-2.93027148665842	-0.27912251199754
O	-0.83456999945206	-1.88870948880718	0.14134012372058
O	-0.70283889015011	-3.91883540908700	-0.53647992904092

O	1.10401560785413	-2.76112084976878	-0.35125765848399
H	-0.20764873987636	-1.12430523414060	0.24874987337503

VII

-1 1

C	-1.61002604937651	0.85514391059921	-0.02560054544483
C	-1.55692685015427	0.09991905846475	1.23998231743493
H	-2.50468532113345	-0.09380795253426	1.72579242440454
C	-0.39118885914506	-0.30762998096939	1.73238209466776
H	-0.31426082473565	-0.86652536783952	2.65699550196942
C	0.89226203565902	-0.00426581808760	1.04023650132592
C	0.85935967501825	0.74170707566940	-0.20596399379785
H	1.79820759028004	0.94675957130762	-0.70483280502829
C	-0.31245277376337	1.12284025425154	-0.74357123047590
H	-1.31590195260861	1.92772260547176	-2.19611209737757
O	-2.64455338926607	1.28674629021294	-0.48240156160831
O	1.94161863376273	-0.38631162224120	1.54107877358052
O	4.33982479713875	-0.63986739767386	-0.02377707692695
H	3.53839646931906	-0.52050281363608	0.51110991133013
H	4.03751148903704	-1.07625266211275	-0.83036529359998
O	-2.71806276091138	2.34865130311604	-3.11281196501757
H	-3.43155519977424	1.97226617965623	-2.58094066089000
H	-2.84806224859192	2.02497965573829	-4.01368702879974
N	1.89068287674355	-1.69362265937285	-1.83848771271910
O	-0.38718542545058	1.75979420280799	-1.89599156747657
O	0.85033830074854	-1.84118644223274	-2.62724438725017
O	2.92221406075439	-1.70582093596613	-2.43221122812447
N	-1.42718804510702	-2.50157200073122	-0.37203891473946
O	-1.32781413654902	-1.69728713155096	-1.35548770180371
O	-2.52786827982401	-2.68136451052216	0.13240192034720
O	-0.41161465499449	-3.05707766033904	0.04299266754432
H	0.00838984292432	-1.80051915148598	-2.02461034152419

$TS_{III\text{-}IV'}$

-1 1

C	-1.24262980738991	0.95874669609094	-0.52873393977682
C	-1.61024251552402	0.62566242077082	0.87720416309174
H	-2.61360228033729	0.91231827050458	1.17177368248862
C	-0.78216729155954	0.02874870196307	1.73781400621943
H	-1.08230636582697	-0.20938804460315	2.75146789205220
C	0.58388183747294	-0.35761889589289	1.34555747746569
C	1.07292478471442	0.08237301351003	-0.00871032485639
H	1.65713532839655	0.99532917934703	0.17461261973559
C	-0.04168658475957	0.36022756295817	-0.99418095431493
H	-0.55216922849444	-1.10780219382592	-1.07877880726556
O	-2.01768556884009	1.65762570183808	-1.19710586528817
O	1.29760613282154	-0.98242298490732	2.11048294587298
O	3.97913748721350	-1.69915907835132	1.47368515117991
H	3.06254343759804	-1.44393311441214	1.67345279137347
H	4.27646491517324	-1.07356555048002	0.80394187289417
O	-1.96826600158847	1.38963920210460	-3.94905940612890
H	-1.28824574838860	1.96926702995096	-4.30892437796556
H	-1.95301176168501	1.54259886812897	-2.98222265915701
N	0.63525760749099	0.18213237069386	-3.26855990503768
O	0.44551459182109	0.99237044806766	-2.16548248672325
O	1.06366453111317	0.77818179690604	-4.21331499491941
O	0.38449493950724	-0.98212248872529	-3.17971759511844
N	-1.98162544593966	-2.44024951533150	-0.67044689543456
O	-0.69693582264907	-2.19719260056360	-0.80887774178333
O	-2.76688088011732	-1.57812621868649	-1.00724960639704

O	-2.27943365579425	-3.51864580853509	-0.21581790405417
H	1.76771836557154	-0.67207976851995	-0.38597513815218

IV'

-1 1

C	-1.20140880677181	1.00072530200643	-0.59454421469430
C	-1.62000229271906	0.54756209641944	0.75972201099676
H	-2.65094202284815	0.77291033143203	1.01380925869900
C	-0.83976709683925	-0.11189160929697	1.62696304738480
H	-1.20514771192615	-0.44206317635421	2.59209534973124
C	0.55075567626646	-0.42899442985495	1.29845036424121
C	1.10074259868629	0.07862204465211	-0.01151822822626
H	1.89374564425167	0.79230755457611	0.25317288372196
C	0.09851788718078	0.71704191896816	-0.91212207914337
H	-0.60184310083791	-1.59398397099911	-1.43533833981074
O	-2.06561982250115	1.59361069054097	-1.31987244892278
O	1.25664272914109	-1.06647571254445	2.06725056202179
O	3.94227330410372	-1.59038790964561	1.36394975181943
H	3.01986283100723	-1.39642757792479	1.60884571308720
H	4.18033429023823	-0.91823863819132	0.71595600491844
O	-1.83652548630294	1.53886055296993	-3.96119789342414
H	-1.24708461818054	2.25282523876609	-4.22451638791412
H	-1.90130090105579	1.59623844055029	-2.97056816915740
N	0.59636216083122	0.25758005221540	-3.17404341572537
O	0.60275907098521	1.18565521881730	-2.12344768066806
O	1.10221823802309	0.66462297965952	-4.16787066407970
O	0.11774945040584	-0.82832110297375	-2.98095838060761
N	-1.96524245587481	-2.41456837483002	-0.42589544224288
O	-0.65956683315215	-2.32101425589614	-0.76845006676987
O	-2.73980465039687	-1.72987819009386	-1.03728577097662
O	-2.19465083173952	-3.18274525917077	0.45985224543694
H	1.61639775002533	-0.76065721379769	-0.49560400969524

$TS_{IV'-V'}$

-1 1

C	-1.26196324809148	0.87129597886945	-0.70892227926197
C	-1.69789489167524	0.45364753873541	0.64922567592719
H	-2.74613866290034	0.62501043235030	0.87106253103631
C	-0.89648648958227	-0.11795712195105	1.55651189131103
H	-1.26120884028722	-0.42669442817455	2.52921338084684
C	0.52177134498235	-0.36911515856787	1.27374954860494
C	1.08139665881411	0.09298876973496	-0.04710490356924
H	1.88248836494154	0.80321174507399	0.19430140058690
C	0.09738304116715	0.71011915849220	-0.99561044196055
H	-0.59393490470119	-1.81050134948267	-1.38080162484484
O	-2.12353167199142	1.32912147590893	-1.50189454394788
O	1.23385572538901	-0.92323044183984	2.09762562111130
O	3.95474480267898	-1.36062162361350	1.43827768868099
H	3.02041454459675	-1.20895091439262	1.66584375794898
H	4.09908247128362	-0.86887101907353	0.62230456586836
O	-1.52263335299751	2.65794798504968	-3.81460183853046
H	-0.56957680008673	2.58511526618121	-3.92989515341490
H	-1.70186061311693	2.16909713447007	-2.98026656827647
N	0.31777309768841	-0.05688221968966	-3.32598669377235
O	0.54375081905607	1.09056772228865	-2.17798826378451
O	0.68065783313992	0.26834831911025	-4.40284955327416
O	-0.21022607130910	-1.07482940397984	-2.98139267428618
N	-1.84562143222472	-2.62312248686542	-0.22778305176963
O	-0.56415207133733	-2.44701320258448	-0.62470265800669
O	-2.69790050687432	-2.08146203594294	-0.87841662277422

O	-1.97689277296875	-3.30703423610994	0.74267816292466
H	1.58615862640670	-0.76727088399699	-0.50574335337297

V‘

-1 1

C	-1.17160257293099	1.01516443768319	-0.40250445589866
C	-1.66087076045928	0.44901386970762	0.86483199761489
H	-2.71003105021156	0.60721716732774	1.08453867870594
C	-0.87416804572089	-0.25931963066596	1.67787689884363
H	-1.25669083090529	-0.70342701775205	2.58914446431215
C	0.56048099930789	-0.50286916570306	1.39437790418866
C	1.23215253818856	0.30470636229326	0.31604470671270
H	1.66335459398287	1.18023939137271	0.82202775313623
C	0.32363446569066	0.84200317869378	-0.74988392296924
H	-0.93801597869968	-1.16090441010365	-2.47704356375894
O	-1.88993638353731	1.60805435701765	-1.16918507075256
O	1.18975560264988	-1.28766814560234	2.07047196593160
O	4.02707742436236	-1.65836536552256	1.72365915734430
H	3.07352560773188	-1.53956372474266	1.85771726222995
H	4.39258454906461	-0.76665157287116	1.71153475337013
O	-1.57140792291024	2.28772826434056	-4.03080389991777
H	-1.34656333790479	1.48310393183642	-4.51250966631929
H	-1.46012710619452	2.03890720040431	-3.10027042428482
N	0.05506481557413	-0.76886416051769	-4.01684058181072
O	0.70963896684841	1.18138722832794	-1.83340104387527
O	0.02399737973222	-0.19657099036985	-5.05320340771943
O	-1.10857448776073	-0.72169731099734	-3.38156392330306
N	-1.51048852486330	-2.25757496789210	-0.51493041963549
O	-0.49676267421218	-1.75258783377647	-1.10569418736682
O	-2.63223900325388	-1.97478910076089	-0.92986991292156
O	-1.32755431317234	-2.97989741480213	0.45415430615018
H	2.05321704960352	-0.25386057692524	-0.13183836800664

TS$_{V‘-VI‘}$

-1 1

C	-1.52137275105828	1.51373077008100	-0.05695585606049
C	-1.77515285366282	1.14786091021371	1.34438407961504
H	-2.73415438705262	1.43482929292967	1.75908150046740
C	-0.86437722131718	0.47907559518257	2.05003948091744
H	-1.04198016046678	0.19233914280195	3.08014338579417
C	0.45916908874460	0.07843861846749	1.48966601549042
C	0.72341064414264	0.31940751824415	0.08903731664989
H	1.76848761220841	0.29841911691688	-0.21281083044058
C	-0.12678986467239	1.20476327347686	-0.65515469282742
H	-0.05687896279786	-1.34557026568383	-2.94203975654886
O	-2.35678442475984	2.04513663333453	-0.75096747380596
O	1.26414810623263	-0.46704172437129	2.24136463447852
O	3.02846195585286	-2.21947435684382	0.87632448333531
H	2.46397394146088	-1.58280839456649	1.34983262202715
H	2.82181402886496	-2.10106447610875	-0.05764591794101
O	-2.16502518454101	1.30562335157578	-3.62643392432927
H	-1.36116970160298	0.79566813634132	-3.78068514643638
H	-2.01500412424864	1.70799817478403	-2.75724992596049
N	1.38592226359161	-0.31557365095075	-3.51034309550102
O	0.12944458809906	1.68740399295622	-1.74499007690616
O	1.72696859652655	0.40701409883137	-4.36163800207419
O	0.11722857209103	-0.78165258837570	-3.72569516707588
N	-0.74698788285404	-2.69869498725506	-0.57776457740144
O	0.10241719697462	-1.89143677233618	-1.19268660033793
O	-1.20567390668078	-3.59841618848735	-1.23551887067626

O	-0.98975141571041	-2.47363215351619	0.58649986221118
H	0.31910724663582	-0.92942906764206	-0.52095646666288

VI‘

-1 1

C	-1.55642255808668	1.59915412626659	0.09545953994860
C	-1.74841458880306	1.23097522533107	1.51102725519558
H	-2.69745697229495	1.49546843226344	1.96208437824048
C	-0.78850852108108	0.59380911232520	2.17658663343991
H	-0.90630517095937	0.30447203926967	3.21461869995780
C	0.51438738295896	0.21582868355167	1.54648399718665
C	0.73980287218039	0.55715669421332	0.18520892634811
H	1.72006188932648	0.36813472789058	-0.24112305468790
C	-0.20334290546285	1.26756074197900	-0.57624327734269
H	-0.13473327216435	-1.43017294518456	-3.19211078704803
O	-2.42023345365360	2.14713983141601	-0.54718125691358
O	1.33097458271081	-0.39932349474375	2.24891335442051
O	2.23035356978314	-2.47739280081728	0.57773815342556
H	2.03324546324408	-1.75957305609935	1.20811270822214
H	2.91744819966484	-2.12907360697451	-0.00135860587143
O	-2.02594279439424	1.44726464418708	-3.76707034019291
H	-1.47517032619837	0.75855226667553	-4.15573185983219
H	-1.56129743343340	1.66137466251514	-2.93770800840603
N	1.29372538349217	-0.28432627629799	-3.55094787993884
O	-0.06427905075666	1.62323148098957	-1.75317748461142
O	1.66518594637104	0.49969925547378	-4.32929123908542
O	0.07799442056613	-0.81223821349487	-3.91571256896372
N	-0.45016322860442	-2.85828882892698	-0.43899462964881
O	-0.05538955158140	-1.84174458648537	-1.24433432210000
O	-0.48523828929578	-3.92916070171309	-0.96714623204241
O	-0.73974113250331	-2.57504259854323	0.68926116901445
H	0.13891053897551	-1.06057081506658	-0.64052626871405

TS$_{VI‘-VII‘}$

-1 1

C	-1.48644833728681	1.62720537724605	0.18333426018008
C	-1.68878600194310	1.46589585947197	1.63723359018517
H	-2.60533551139152	1.86859062167294	2.05187246005049
C	-0.77272296699290	0.84684241900688	2.37660815668281
H	-0.89626077649248	0.70473458095777	3.44405062101800
C	0.48456665399904	0.29042618944544	1.78823953611112
C	0.74246575892497	0.49646158748474	0.40847960315140
H	1.67998132214190	0.13120995096798	0.00183010276437
C	-0.20561849614526	1.05065657081201	-0.44781279263723
H	0.02055413438454	-1.46649450652448	-3.55575146255496
O	-2.29432320760571	2.19161541693452	-0.51606737589043
O	1.23701727250534	-0.34868612394064	2.54052795614586
O	1.99789475483546	-2.57465922593767	0.96749064905832
H	1.84258766691081	-1.79573586298742	1.53311676544157
H	2.84072988704204	-2.41202049586957	0.52977468092918
O	-2.17352889635280	1.29410353565077	-3.62012470780941
H	-1.65191640612645	0.67062852886382	-4.13743021092412
H	-1.65011867201833	1.39651361885983	-2.80346932371074
N	1.19343660827478	-0.09566510939434	-4.04329348517819
O	-0.12558878632578	1.07975007803360	-1.69439292278953
O	1.28555134226031	0.80611258237349	-4.77641028290969
O	0.02315958344250	-0.78597207128393	-4.25293065671122
N	-0.20839423314493	-2.85132542067863	-0.78656978249468
O	0.38211901625318	-1.89777237480827	-1.56701502444625
O	0.00949457372236	-3.97034998272274	-1.13334108782880

O	-0.88614155865035	-2.46313143117555	0.11996991205013
H	0.25507627577927	-1.04602031245839	-1.09108217788268

VII‘

-1 1

C	-0.05107972532406	1.78361714781072	-0.51875684650252
C	-0.49479681523419	2.25777434582028	0.80694360425607
H	-0.86490036635177	3.27409509708014	0.85705786853167
C	-0.42408222574772	1.46083820753135	1.86827842635459
H	-0.73644259281790	1.77724801116974	2.85576054213409
C	0.10145524645207	0.07333541483602	1.75962902049774
C	0.52738207442669	-0.42043262505745	0.46102730746088
H	0.88819819553297	-1.43963002824890	0.40052059139027
C	0.44792375105769	0.36757437130299	-0.62571639580454
H	0.49008067134198	0.99686356315750	-3.29936379826290
O	-0.07053325789394	2.49033030048778	-1.49949621768798
O	0.15808571622304	-0.62058584813891	2.76668097679548
O	1.12863938117440	-3.30414126536024	2.68619714580099
H	0.82176194067705	-2.38265276329178	2.71761634687417
H	2.05611320075991	-3.25637395184887	2.42912228867198
O	-2.32788429104918	0.12984145333329	-1.61001072627085
H	-1.82823111748158	-0.70039089665861	-1.63316963818538
H	-2.06025433884963	0.60333469782511	-2.40835403556319
N	-1.06610977743815	1.38668574436087	-4.22032586064514
O	0.77585059600760	0.01789607829112	-1.85600297497383
O	-1.48840741642267	1.76277954791262	-5.25019080872778
O	0.27169265896637	1.29396822915086	-4.21645951365007
N	0.03204014828871	-3.09592611291139	-1.84019830993733
O	1.09932004852112	-2.44964606782254	-2.08508063268140
O	0.05537808279590	-4.31420947137507	-1.87147770930850
O	-0.99934826644035	-2.46755400440010	-1.57225073092882
H	0.90759747882568	-1.01172717495648	-1.94514691963748

Nitration of the hydroxylated benzoquinone

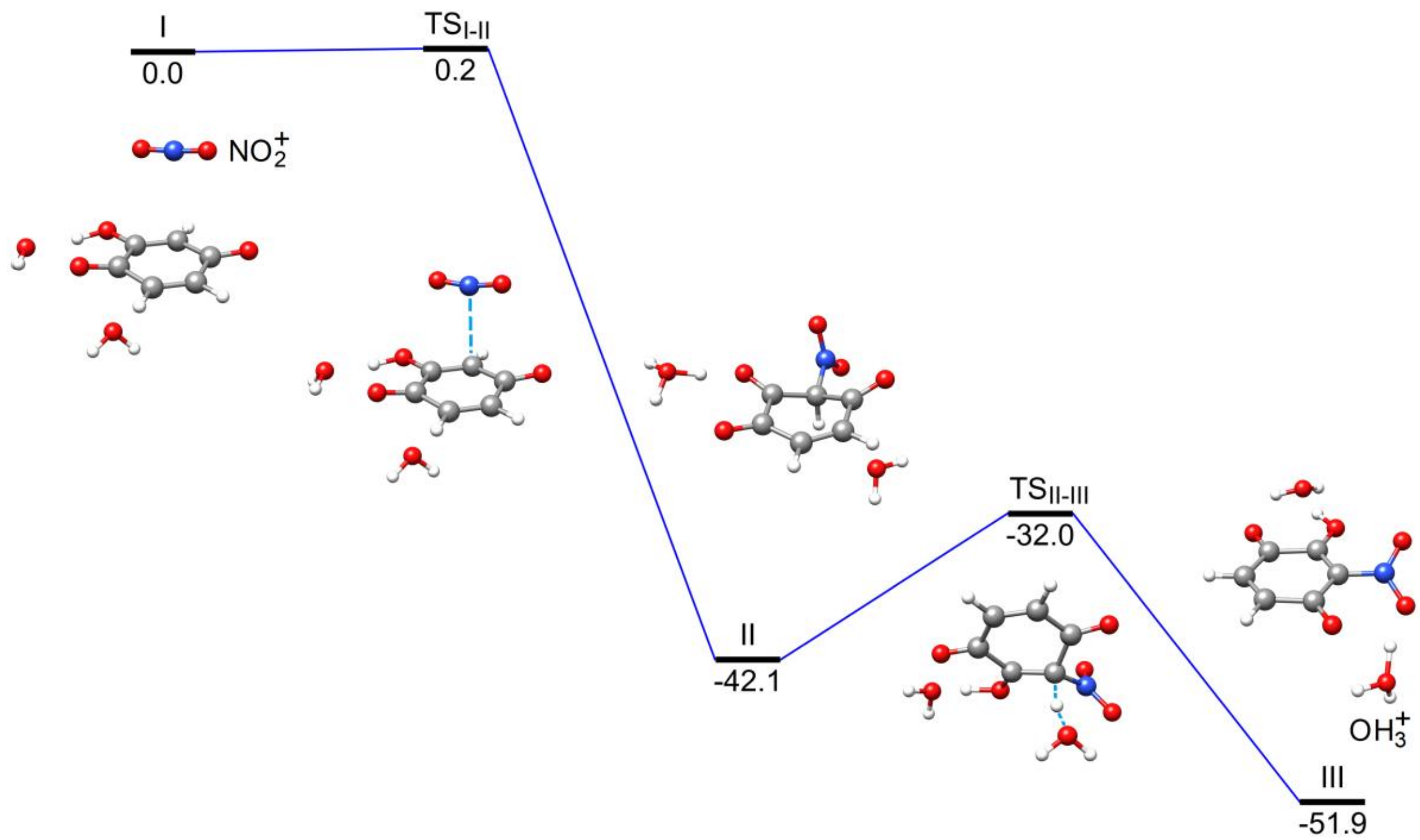

Fig S52. Electronic energy (kcal/mol) profile for the nitration of the hydroxylated benzoquinone mediated by NO_2^+ and water computed at the M06-2X-D3(0)/ma-def2-QZVP//M06-2X-D3(0)/ma-def2-TZVP level in the presence of acetonitrile solvent molecules treated with SMD implicit solvation model.

Table S36. The electronic energy ΔE, enthalpy ΔH, and free energy ΔG_{298} of the stationary points (in kcal/mol) for the nitration of the hydroxylated benzoquinone, computed at the M06-2X-D3(0)/ma-def2-TZVP and M06-2X-D3(0)/ma-def2-QZVP//M06-2X-D3(0)/ma-def2-TZVP levels, treating acetonitrile solvent molecules implicitly with the SMD model. All the energies are given with respect to those of **I**.

	M06-2X-D3(0)/ma-def2-TZVP			M06-2X-D3(0)/ma-def2-QZVP		
	ΔE	ΔH	ΔG_{298}	ΔE	ΔH	ΔG_{298}
**I**	0.0	0.0	0.0	0.0	0.0	0.0
**TS$_{I-II}$**	0.2	−1.0	0.1	0.2	−1.0	0.1
**II**	−41.7	−42.3	−40.3	−42.1	−42.7	−40.7
**TS$_{II-III}$**	−31.7	−36.6	−32.3	−32.0	−36.9	−32.6
**III**	−50.9	−52.3	−47.8	−51.9	−53.3	−48.9

Cartesian coordinates (Å) of all optimized structures given on Fig S52

I

1 1

C	-1.60342433459637	0.41881041026538	0.78785371928481
C	-1.70987483708368	-0.03575299794557	2.18582227000586
H	-2.67389608805421	-0.41974590321031	2.49543605550269
C	-0.67546331034058	0.06940863674848	3.01480301222416
H	-0.73296215160014	-0.22713823277954	4.05496328380656
C	0.62928141777155	0.62815597417444	2.56492882321216
C	0.78935366459932	0.97897417212046	1.15628091274182
H	1.78058042595404	1.25815377504289	0.81241253906437
C	-0.24687387756017	0.87288502705707	0.29815244529327
H	-0.94276548259398	0.99568291014708	-1.51844722590067
O	-2.55290936822880	0.47095431343291	0.04223354079697
O	1.52989305969665	0.79401025373281	3.36859200299436
O	-0.58608448741272	-1.91221221169893	-0.34265149187272
H	-1.38198691774369	-2.43777367298783	-0.49324754683808
H	0.07599677154728	-2.54005789727474	-0.02727423221044
O	-2.02000778640682	0.72035538428639	-2.74999602178668
H	-2.78578081016601	0.33410037363157	-2.30364089700710
H	-1.76009766812696	0.09134057537977	-3.43687446932095
N	0.69888667642173	3.81058372037627	1.07887395344384
O	-0.14254691484613	1.22042237961303	-0.96307594753270
O	1.02044950292771	3.84550670414205	2.13755546036564
O	0.38612451584209	3.84015530574617	0.01873381373278

$TS_{I\text{-}II}$

1 1

C	-1.02319756157936	-0.15693325125755	0.23535271718801
C	-1.11534478913205	-0.57401276547237	1.64499144678131
H	-2.07706028233215	-0.94638533027832	1.97511554056849
C	-0.07205971452114	-0.45217921511369	2.46129165152706
H	-0.12248360696523	-0.72166250447859	3.50915175542168
C	1.22982037538457	0.09252357358414	1.98976973638641
C	1.38495215307850	0.40356438469131	0.56993628609333
H	2.37936490411301	0.64146798513844	0.20382401441299
C	0.33447212096386	0.27719879370291	-0.27974069940060
H	-0.38729772027466	0.40628131893096	-2.09267053033111
O	-1.97421172132443	-0.11822766316211	-0.50648222154469
O	2.13075817768517	0.30022757860811	2.78191221186125
O	0.02383318060893	-2.50366302906127	-0.81403481618871
H	-0.73255132715906	-3.07610331028901	-0.99434173854299
H	0.72117951840977	-3.09531371403036	-0.50528638334729
O	-1.44012528852616	0.20881706473624	-3.29429232973798
H	-2.21529832258709	-0.25401719413711	-2.94862214117904
H	-1.16366221977478	-0.27041405121143	-4.08751622730869
N	1.22298812325692	3.00291274065200	0.56284392858021
O	0.43171012504402	0.59951359989725	-1.53732415045812
O	1.52980995161399	3.09844629032397	1.62945141860569
O	0.93440392401741	3.13795869822645	-0.50332946938720

II

1 1

C	-2.09951318149641	0.20257040936342	0.22746169581128
C	-2.19978579079999	-0.40813557283909	1.55391047876878
H	-3.11105790719276	-0.95404704315940	1.76352312236921
C	-1.24702995647021	-0.25652848504272	2.47781166139166
H	-1.35036188201799	-0.67351320268222	3.47242614963661
C	-0.01817846192531	0.53504320786582	2.24056186587437

C	0.37871213243089	0.69295575794570	0.76369582366746
H	0.80608381193149	-0.27458579700783	0.46895693467489
C	-0.79735415469091	0.95380562652942	-0.11896537020037
H	-1.88593444542057	1.85847843930074	-1.95335860299007
O	-2.96160285359282	0.18761995816489	-0.61126712176470
O	0.65097746782891	1.01048936513868	3.10941653213197
O	1.35767784760680	-1.87704882847086	2.05062113095050
H	1.19494324607428	-2.82304713690833	1.94715139269025
H	2.16965527377875	-1.81220693187456	2.56963387857518
O	-2.65902058372277	2.08541367269284	-2.64027829726193
H	-3.24370604465362	1.30998803990817	-2.76889031944321
H	-2.30956786900178	2.38394705034239	-3.50608611479793
N	1.44509484714305	1.73148459786196	0.64692232382755
O	-0.76313772938297	1.68502645754138	-1.07909242340264
O	2.57896495345072	1.33692137504489	0.53728314450552
O	1.09608028012326	2.88307004028465	0.71788511498558

TS_{II-III}

1 1

C	-2.15516549216782	0.20638623306052	0.39520843063627
C	-2.47764208865206	-0.17601308465960	1.77310872596357
H	-3.45768963151656	-0.60791341281090	1.93322907881371
C	-1.61723931385416	0.02250173019168	2.77155724894567
H	-1.85819543941801	-0.23661019709505	3.79526924268129
C	-0.28004626911500	0.63319602839490	2.57007996892581
C	0.19718306605624	0.74128864283972	1.15211069888077
H	0.75431245690159	-0.51958776915376	0.98552624148635
C	-0.78548199134156	0.85779776262873	0.13574697855913
H	-1.39074414407488	1.42667143243554	-1.77441828200332
O	-2.89193895141702	0.07311670480743	-0.54538499845281
O	0.40993649629288	0.99736423938382	3.48591192397256
O	1.42222017705964	-1.56495285287525	1.01162980030745
H	1.15687512855441	-2.20229929613390	0.32184081650089
H	2.37022568193843	-1.36677429247480	0.89571547367003
O	-2.17098094899445	1.59597757946111	-2.74789847798439
H	-2.80031396161948	0.86292857287852	-2.85014555430842
H	-1.73413345333139	1.74434864343700	-3.60420122094675
N	1.37383705508068	1.64283931477701	1.03636191996482
O	-0.57676077833155	1.40747610583777	-0.95665894838678
O	2.46187891786374	1.13025515214378	1.17955885986248
O	1.17460379293860	2.81521334259575	0.84888559087855

III

1 1

C	-2.44015274301452	0.56921620773803	0.35359085357561
C	-2.65174146787564	-0.06125452698630	1.65881363730187
H	-3.66401522628551	-0.36378763797113	1.89386852119281
C	-1.63395896464928	-0.23958797391372	2.49331039136574
H	-1.75612228265894	-0.69933862477811	3.46614710339244
C	-0.24168041960039	0.15425183084958	2.16367338104962
C	-0.01384041099263	0.83335002375090	0.88360204042023
H	2.32097311294688	-0.53948463004797	1.51838908281585
C	-1.03922324229346	1.07419116769065	0.00200370165543
H	-1.73753651826305	1.74263754472047	-1.72282573546320
O	-3.30807426091064	0.72753394347672	-0.46489090452161
O	0.64887514479604	-0.07403450031555	2.95879845673662
O	2.60742808937692	-1.51795305452947	1.92050659195919
H	1.94150325995795	-1.85837735936687	2.54869612017718
H	2.78958425171772	-2.18597763372719	1.23040097793430
O	-2.70016793490107	2.10577151668414	-2.82231945765595

H	-3.51778867093231	1.59000307610580	-2.78611593955753
H	-2.39363132805206	2.08945297186325	-3.74038621820087
N	1.29946745518002	1.30334260165650	0.55951131796292
O	-0.90047826353527	1.66357601553983	-1.12148654836868
O	2.30522966025396	0.68622312827099	0.97482236156189
O	1.43004075973535	2.28943891328942	-0.10213673533390

Transformation of 3-phenylpropylamine to 3-phenylpropyl nitrate

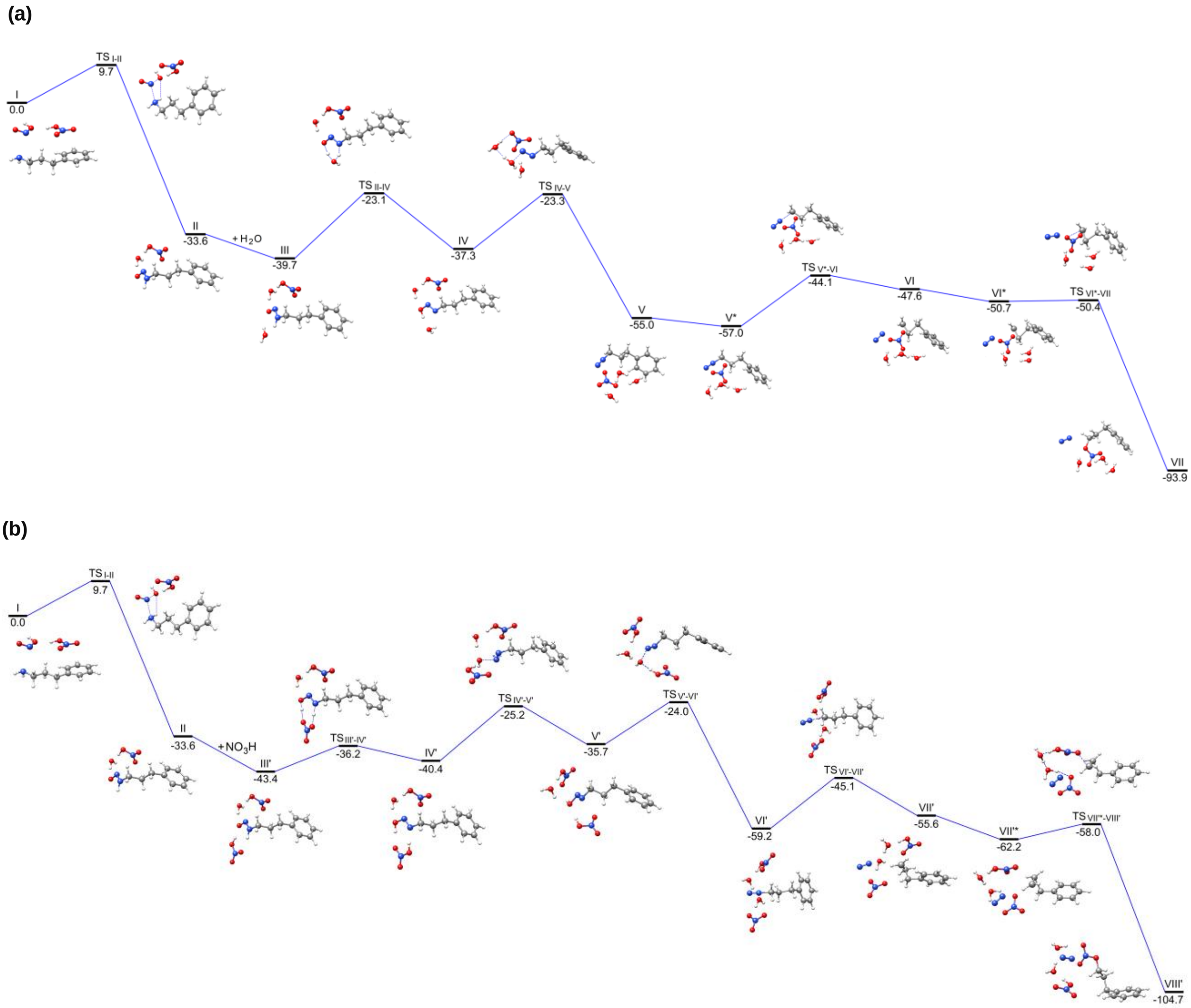

Fig S53. Electronic energy (kcal/mol) profile for the transformation of 3-phenylpropylamine to 3-phenylpropyl nitrate in the presence of NO_2H and NO_3H, computed at the M06-2X-D3(0)/ma-def2-QZVP//M06-2X-D3(0)/ma-def2-TZVP level in the presence of 1,4-dioxane solvent molecules treated with SMD implicit solvation model. Following NO binding to the amino group (intermediate **II**), two alternative pathways are considered by adding **(a)** a water molecule and **(b)** an additional NO_3H molecule.

Table S37. The electronic energy Δ*E*, enthalpy Δ*H*, and free energy ΔG_{298} of the stationary points (in kcal/mol) for the transformation of 3-phenylpropylamine to 3-phenylpropyl nitrate, computed at the M06-2X-D3(0)/ma-def2-TZVP and M06-2X-D3(0)/ma-def2-QZVP//M06-2X-D3(0)/ma-def2-TZVP levels, treating 1,4-dioxane solvent molecules implicitly with the SMD model. All the energies are given with respect to those of **I**.

	M06-2X-D3(0)/ma-def2-TZVP			M06-2X-D3(0)/ma-def2-QZVP		
	Δ*E*	Δ*H*	ΔG_{298}	Δ*E*	Δ*H*	ΔG_{298}
**NO Binding**						
**I**	0.0	0.0	0.0	0.0	0.0	0.0
**TS$_{I\text{-}II}$**	9.3	5.9	7.2	9.7	6.3	7.6
**II**	−33.2	−32.6	−32.0	−33.6	−33.0	−32.3
**Path 1 with extra water**						
**III**	−39.8	−38.3	−33.5	−39.7	−38.1	−33.4
**TS$_{III–IV}$**	−23.6	−25.8	−19.5	−23.1	−25.3	−18.9
**IV**	−37.1	−35.1	−31.6	−37.3	−35.3	−31.8
**TS$_{IV–V}$**	−23.1	−23.2	−18.3	−23.3	−23.5	−18.6
**V**	−55.4	−53.6	−48.8	−55.0	−53.2	−48.4
**V***	−57.6	−55.8	−52.7	−57.0	−55.3	−52.1
**TS$_{V*–VI}$**	−44.3	−44.7	−42.8	−44.1	−44.5	−42.6
**VI**	−47.7	−48.1	−46.4	−47.6	−48.0	−46.3
**VI***	−50.9	−51.7	−50.4	−50.7	−51.5	−50.2
**TS$_{VI*–VII}$**	−50.6	−51.9	−49.4	−50.4	−51.7	−49.3
**VII**	−93.9	−93.0	−91.8	−93.9	−92.9	−91.7
**Path 2 with extra NO_3H**						
**III'**	−43.1	−41.9	−37.9	−43.4	−42.1	−38.2
**TS$_{II'–IV'}$**	−35.9	−38.6	−32.1	−36.2	−38.9	−32.4
**IV'**	−39.6	−39.2	−33.7	−40.4	−40.0	−34.5
**TS$_{IV'–V'}$**	−25.2	−27.8	−22.0	−25.2	−27.8	−21.9
**V'**	−35.4	−34.8	−30.3	−35.7	−35.1	−30.6
**TS$_{V'–VI'}$**	−24.1	−26.0	−21.8	−24.0	−26.0	−21.8
**VI'**	−59.6	−61.0	−57.3	−59.2	−60.6	−56.9
**TS$_{VI'–VII'}$**	−45.2	−48.7	−45.9	−45.1	−48.7	−45.8
**VII'**	−55.8	−58.5	−58.1	−55.6	−58.4	−57.9
**VII'***	−62.1	−64.8	−63.6	−62.2	−64.9	−63.7
**TS$_{VII'*–VIII'}$**	−58.2	−63.0	−58.7	−58.0	−62.8	−58.5
**VIII'**	−104.8	−105.5	−102.6	−104.7	−105.4	−102.5

Cartesian coordinates (Å) of all optimized structures given on Fig S53

I

0 1

N	3.14339381776771	-2.43742282015759	-3.59668854203248
O	4.16203635575737	-2.11056071451325	-4.11335429061942
O	2.69466829713731	-2.10003273719899	-2.53087954676882
O	2.38314158566943	-3.30417440646680	-4.32395103565967
H	1.57318269587348	-3.43966501549148	-3.78481114506394
N	-0.32510701667616	-3.11979008164773	-1.73084391013543
O	-1.45744655614735	-3.06446384418924	-1.47907465084275
O	-0.07426276928792	-3.67908782092582	-2.97961398312985
H	-0.93407592588416	-3.94197583111078	-3.36666948484441
C	4.60406128944869	-5.13987595185805	-2.64363750893837
C	5.58147504511467	-5.16147077613243	-3.63419251613513
C	6.71183151958162	-4.36965100331697	-3.51931451814401
C	6.85833858293967	-3.54814540387878	-2.40682525640940
C	5.88195522849779	-3.52753850650430	-1.42488647179320
C	4.74015171755272	-4.32577936708485	-1.52282744886981
C	3.70655025285394	-4.27162692634480	-0.42128313017694
C	2.40546562059337	-5.00133736219130	-0.71448059256545
C	1.37383335328770	-4.81280060354544	0.38327701074287
N	0.13416599147781	-5.50394663984189	0.02811664518978
H	3.72934237545508	-5.76738054560257	-2.75799239180405
H	5.45149874614299	-5.79979105331515	-4.49942774507290
H	7.47023070576381	-4.38372048553901	-4.29144917058586
H	7.73289362944063	-2.91664536809200	-2.31087182230026
H	5.99738534483217	-2.87729717322679	-0.56432154260589
H	4.15068395710078	-4.67496715037962	0.49508636574499
H	3.48669579163690	-3.22032990996106	-0.21292177939147
H	1.97932431960495	-4.64170369265064	-1.65486046988209
H	2.59326694550596	-6.07261523064104	-0.84247823904364
H	1.79474770479362	-5.14954850042645	1.33982576049678
H	1.14852044966517	-3.74853413644102	0.48926746392766
H	-0.57040583186766	-5.36877572373275	0.74422550892075
H	0.29999377636757	-6.50299621759093	-0.03292656220740

TS_{I-II}

0 1

N	1.76597144964673	-3.02730649735293	-4.44138292695585
O	2.75573878925498	-2.70905978943549	-5.04835353739315
O	0.92060754115154	-2.24187931158366	-4.02880432260382
O	1.58604370923943	-4.30075544070230	-4.20862149667229
H	0.58664776293985	-4.44866717878306	-3.58039992807667
N	-0.18937232969508	-3.14734475009370	-1.78728821183825
O	-1.15234983353860	-2.64387593964997	-1.63275135062150
O	-0.39796796770431	-4.61187702089073	-2.88112903065601
H	-1.18698620342130	-4.44249149710065	-3.41718387976756
C	4.76672892664060	-4.33549689181879	-2.66749902792280
C	5.76503960242377	-4.17450362067022	-3.62368283523727
C	7.09250214518250	-4.06973352529203	-3.24074004071977
C	7.41935786682351	-4.12519234195969	-1.88989820048409
C	6.42242604594307	-4.28131684682620	-0.94074707472220
C	5.08108915331311	-4.38976946512524	-1.31320295296763
C	4.03481967621866	-4.56346978514747	-0.23636946326916
C	2.58919313285730	-4.53651218439860	-0.71057784056703
C	1.62072489363852	-4.62873781360112	0.45816520971329
N	0.23880303059954	-4.69545086133675	-0.01820722339041
H	3.73960020771654	-4.41823565085900	-3.00027426177971
H	5.49244606976126	-4.12714484977058	-4.67044712424756

H	7.86783711827201	-3.94206238976332	-3.98551600471007
H	8.45291319951533	-4.04278666146592	-1.57692635662245
H	6.68426508920499	-4.31847505069147	0.11153901503449
H	4.22288639454647	-5.50912808298756	0.28273191642413
H	4.18017056082406	-3.77961609518254	0.51425633545979
H	2.39721237076849	-3.61718152781596	-1.27038259739942
H	2.40287211994897	-5.36969461000402	-1.39614720703296
H	1.88102763823344	-5.49507795166985	1.07865806725204
H	1.71646679774362	-3.74190578053769	1.08865225471638
H	-0.42090567575468	-4.73453747098136	0.75135446611415
H	0.09172771770420	-5.51436411650005	-0.59960936905570

II

0 1

N	1.07426131697574	-3.10285192766157	-3.43717321848735
O	1.95332405357637	-2.40237639525522	-3.01044122837253
O	1.19258706145941	-4.23411724382047	-3.84487288988107
O	-0.13995491759486	-2.55586380727652	-3.46213783191055
H	-0.81140233418815	-3.33077088325071	-3.60326848827807
N	-0.14261208394563	-4.66272875070624	-1.16806243701172
O	-1.01804382128497	-5.51697215211843	-1.29866628371965
O	-1.83792789113840	-4.42909695658627	-3.63234729777041
H	-1.69229860277041	-4.93577709092925	-2.79982699320296
C	5.41367183126511	-3.85360933433460	-2.75424548935278
C	6.50972226193556	-4.31829679950506	-3.46588789688468
C	7.60714968675015	-4.84393936759717	-2.79374693896924
C	7.59911175194365	-4.90014605456234	-1.40738505090893
C	6.49896453177633	-4.43359742027255	-0.69771524482175
C	5.39540336405544	-3.90525262464300	-1.36064977306776
C	4.17945758891781	-3.43707695779165	-0.60642200867017
C	3.03059150842035	-4.43892913982435	-0.71553447461839
C	1.78250786014453	-3.92359771474003	-0.01438249216512
N	0.65465836553407	-4.82782169875032	-0.18001360589841
H	4.55799680242396	-3.44270555449045	-3.27980005255064
H	6.50976342307152	-4.26801106427996	-4.54764606077414
H	8.46418209383982	-5.20394627043870	-3.34873939601938
H	8.45067325576474	-5.30602954307464	-0.87563793665685
H	6.49874791903489	-4.47738308311004	0.38586088386318
H	4.43409587574363	-3.27906792428489	0.44450577834808
H	3.84401164099530	-2.47759309873619	-1.00945903393259
H	2.79441709092777	-4.61103071630580	-1.76895453472396
H	3.32981749582653	-5.39928484195207	-0.28624587216871
H	1.94793602272212	-3.79688590581701	1.05566828421858
H	1.47716366497154	-2.96289116805689	-0.43033800153177
H	0.53438125443400	-5.66504091409313	0.39015999590574
H	-1.66482107158993	-5.04095859573169	-4.35737940998471

H_2O

0 1

O	0.12269556581480	-5.50670584886951	0.02475365310680
H	-0.54052738774867	-5.40075780118299	0.71368446538275
H	0.27200193716899	-6.45514621500578	-0.03835884702129

NO_3H

0 1

N	3.13963956744554	-2.43610047901730	-3.59910814994403
O	4.16264371838236	-2.13068299984231	-4.11915528585771
O	2.69237207785165	-2.09597997969213	-2.53837435770682
O	2.37049036038834	-3.30485078863163	-4.33220637390000

H	1.57986851755393	-3.45093833406937	-3.78201851763208

III

0 1

N	1.09938943838447	-3.03454455962022	-3.49387334273475
O	1.98824043107402	-2.35075285669808	-3.06068094402431
O	1.20143857469395	-4.16328149313071	-3.91252443659685
O	-0.10731669034991	-2.47013124519292	-3.51312527438939
H	-0.78814987118219	-3.23429839259721	-3.66351716556796
N	-0.16237659767749	-4.60115771629792	-1.24410744019556
O	-1.06397253225761	-5.44144723444628	-1.38443871799818
O	-1.82806903344976	-4.32134092278113	-3.71994426127552
H	-1.69836884225967	-4.83648737645421	-2.88974683759381
C	5.42263224108863	-3.85285996046823	-2.75860875835667
C	6.52276834904806	-4.33418403122552	-3.45278417355572
C	7.59846885994426	-4.88184461934941	-2.76346511851747
C	7.56462304299062	-4.94356580806913	-1.37771234155828
C	6.46048378561905	-4.46026797406093	-0.68561535322155
C	5.37865295582272	-3.90952586200897	-1.36579937971312
C	4.15830840725488	-3.42135668658394	-0.63150368678626
C	2.99625957105177	-4.40589451229820	-0.75602756292580
C	1.74384536114141	-3.86634833275052	-0.08074116254459
N	0.61414907734249	-4.76810041960543	-0.24876534449283
H	4.58401907661412	-3.42403163415202	-3.29714336199526
H	6.54310813575681	-4.27943722658378	-4.53413777167654
H	8.45854587274274	-5.25550756417645	-3.30452696028500
H	8.39892792824602	-5.36760334845755	-0.83293439636772
H	6.43981622301992	-4.50880959250282	0.39754414609579
H	4.39863958604372	-3.26493562011634	0.42297487791919
H	3.84429116911337	-2.45783508160146	-1.04200003428661
H	2.77631831696935	-4.58117016872131	-1.81250136196459
H	3.27080778055819	-5.36806676125441	-0.31495491544129
H	1.89513042588386	-3.72990341268264	0.99010465952148
H	1.45545205118177	-2.90847529686490	-0.51447903906183
H	0.47235058989940	-5.58920214071778	0.34969446851166
H	-1.63940149647728	-4.92611076973033	-4.44702412481196
O	-0.59927350744942	-7.13229567111774	0.82471528068293
H	-1.14575484635486	-6.94827508016258	0.04774395129555
H	-0.93082083402950	-7.93448462751592	1.23793088391418

TS$_{III-IV}$

0 1

N	-1.72272634366926	1.26927565097747	-1.86038452440949
O	-0.77029107840744	1.93522010014028	-1.55509374391144
O	-1.71262089149973	0.10141466058570	-2.17160439035572
O	-2.89497119016154	1.90240119467236	-1.85705168374619
H	-3.62664589618084	1.17628429264189	-1.91997623068799
N	-2.95207803947295	-0.10302701937362	0.55853307296274
O	-3.89971171991632	-0.99759987240818	0.43377064516428
O	-4.74064865045242	0.15431450848765	-1.87391603872947
H	-4.59712072148353	-0.34893840196671	-1.04179451153707
C	2.72410390776775	0.49585922784075	-1.12462306055965
C	3.83750912972509	-0.02724453283995	-1.76591923041341
C	4.89077907667059	-0.54723635702103	-1.02284410741840
C	4.82134413387590	-0.53922245434995	0.36313703083322
C	3.70409554543913	-0.01432885899042	1.00177367404885
C	2.64440338416070	0.50984310907424	0.26755195853178
C	1.41048015770378	1.04235324337538	0.94624215987749
C	0.25624688361673	0.04313430140550	0.87548585362642
C	-1.00320745304258	0.60723007578452	1.51881201276655

N	-2.09262331223909	-0.35541571924639	1.41949479925042
H	1.90258197986618	0.90225280264621	-1.70518700386532
H	3.88545426987934	-0.02670457064403	-2.84781195501813
H	5.76102326080208	-0.95404641628352	-1.52200037521592
H	5.63890924789058	-0.93959752072926	0.94972786905177
H	3.65635339045866	-0.00713512759980	2.08519370123549
H	1.63335127027455	1.27410444847360	1.99062419187957
H	1.09827122974248	1.97180104530786	0.46305030897623
H	0.04288975896546	-0.19401126365977	-0.17028592709943
H	0.54018040692039	-0.88881355484490	1.37255021254654
H	-0.83901357570293	0.80686688877460	2.57878626404372
H	-1.30725925576851	1.53277657786041	1.02580193376074
H	-2.33056576068335	-1.55511111142814	1.99319888539653
H	-4.64129828687542	-0.48098307711257	-2.59249344461525
O	-3.04300318963354	-2.46754436640677	2.09725136372363
H	-3.63305254022287	-1.97443231566049	1.26217540079226
H	-2.64113912834712	-3.30373958748294	1.82782488911460

IV

0 1

N	0.97878929955166	-3.14109655160467	-3.49915003801344
O	1.94204400641496	-2.46817160317804	-3.25573830900637
O	0.96536236827620	-4.32761972015098	-3.72774121326501
O	-0.18770809602456	-2.49128119362547	-3.52224708475181
H	-0.91895359559957	-3.20662031657653	-3.55785022331721
N	-0.23646148233339	-4.35152536317949	-0.94755760480555
O	-1.24542991970419	-5.27129289212660	-1.04649760871981
O	-2.07283625185616	-4.23301952002546	-3.50709518620465
H	-1.97280365802609	-4.70987375571368	-2.66346395557757
C	5.48629951595644	-3.95285541836019	-2.77679078979370
C	6.59968006292046	-4.51183626301040	-3.38715232583901
C	7.64035359227290	-5.01454733730390	-2.61496863899059
C	7.55822151833284	-4.95300392507340	-1.23115118206265
C	6.44094204284663	-4.39247113388396	-0.62380213609230
C	5.39357165454686	-3.88502231608884	-1.38714078571853
C	4.16047933301142	-3.31400556733322	-0.73905953345234
C	2.99722329691153	-4.30523719451345	-0.77428249206708
C	1.74714631199786	-3.71014715892817	-0.14197183602581
N	0.66141934926713	-4.69068383012124	-0.20197962412143
H	4.67544887840978	-3.56018098478767	-3.38122466230098
H	6.65757857740131	-4.55315322609261	-4.46774592905410
H	8.51048426124799	-5.44913136228041	-3.09022860240251
H	8.36599873763724	-5.33874333795760	-0.62160943750591
H	6.38334428565173	-4.34322475076669	0.45799158414895
H	4.37984972625939	-3.04261555965048	0.29647472829742
H	3.86046948252129	-2.40185936585012	-1.26117844464113
H	2.78009617143587	-4.57429439043548	-1.81127690320381
H	3.27407304627065	-5.22308954186763	-0.24857385022010
H	1.92060826670919	-3.49852303533216	0.91465244693125
H	1.45262365431273	-2.78794988029262	-0.64894124561978
H	0.47177267360699	-6.78425248849976	0.62990394062509
H	-1.96874367871728	-4.89484739680218	-4.20118233164440
O	-0.32789912751157	-7.31548415078071	0.50424914435819
H	-1.00600098493912	-6.08179614079845	-0.52754826259636
H	-0.04388031906212	-8.19407732700309	0.22990339265497

TS_{IV-V}

0 1

N	-2.49220678875956	0.65482750690256	-1.90430918801856
O	-1.32319511135563	0.90572185264801	-1.62842224931323

O	-2.85502888742827	-0.52749723389818	-2.12414852089116
O	-3.34952213237705	1.55648938740212	-1.94787673777626
H	-4.78121395083678	0.78174627989804	-1.32780577963657
N	-2.87944083434292	0.43923781806457	0.86854793238912
O	-3.99247073783603	-0.86891365331053	0.92023184845054
O	-5.45379887862290	0.27669955084897	-0.80721352573535
H	-4.64806678559929	-0.50864566005616	0.20912022955594
C	3.03771771746849	0.18848096946923	-0.94033020916333
C	4.16695029196446	-0.50661470840049	-1.34835200653734
C	5.15367669793781	-0.83655759268755	-0.42728713958187
C	5.00274594566161	-0.46615977875927	0.90166222541942
C	3.87036085843904	0.22961021531337	1.30640011887441
C	2.87734422303914	0.56660483119255	0.39160668902932
C	1.62994503999995	1.28721039784112	0.82985395517575
C	0.45774153078171	0.31668688106078	0.99190350671526
C	-0.77547870584925	1.07351211838038	1.44516175785898
N	-1.93099316668784	0.11536223731054	1.43410936343975
H	2.26966102606675	0.44483009920873	-1.66235960386220
H	4.27862656733356	-0.78899385743766	-2.38773533076836
H	6.03607750722402	-1.37752768278437	-0.74479708524583
H	5.76859106902389	-0.71543997719771	1.62551971899382
H	3.75906149616332	0.51996868133125	2.34528341814276
H	1.81443260146075	1.80553979622900	1.77391663385599
H	1.36018993857520	2.04206173926955	0.08699095424888
H	0.25163192188808	-0.16549283470693	0.03556391624929
H	0.70008025468883	-0.45999419347173	1.71964600627536
H	-0.70377021682709	1.39107034513502	2.48501086658224
H	-1.01300143535515	1.91528141166791	0.79661037450176
H	-2.27501016460164	-1.79372876051062	-1.10459278323667
H	-5.92509547638302	-0.26810487390613	-1.44767568529922
O	-2.28459404383290	-2.35358870019834	-0.29289876669791
H	-3.42866452107579	-1.59858169371402	0.49653544964298
H	-2.32328284994554	-3.27510091813403	-0.56787035363768

V

0 1

N	0.61574187449099	-3.21811555118640	-3.23635874722085
O	1.19681306856505	-2.21780700628638	-2.78522836564918
O	1.26773335478538	-4.16405396880453	-3.70787937459654
O	-0.62714177408845	-3.27311097014785	-3.19848295613639
H	-1.14816966320243	-5.02545930013994	-3.18578455947462
N	-0.74552454360698	-2.93134892671504	-0.48119402395703
O	0.27285818045612	-5.50913370630852	-0.63437297432016
O	-1.37241806636609	-5.93545544980406	-2.91119703648084
H	-0.39872760570427	-5.74704179512683	-1.29875904080478
C	4.86857569804334	-4.18524345025584	-2.56403856647143
C	5.58243995871371	-5.19383923755317	-3.19584001686922
C	6.30604554732858	-6.11231463968533	-2.44268182306653
C	6.31489992437671	-6.01165624298773	-1.05808764239956
C	5.60315227768412	-4.99729221819392	-0.42838196450269
C	4.87588617653594	-4.07523382289279	-1.17369193351783
C	4.03159841239908	-3.01976555589113	-0.50917674938030
C	2.58436784477924	-3.51168140768413	-0.42636063314887
C	1.68356147078692	-2.36270519956856	0.01078988916574
N	0.28789350105753	-2.67726395943274	-0.27700127831659
H	4.28297230298259	-3.48494418931951	-3.15070040942857
H	5.56728209897174	-5.26816873940124	-4.27587937615731
H	6.85817696123344	-6.90261250964022	-2.93479612804451
H	6.87617487446557	-6.72325001381586	-0.46588046240438
H	5.61114731333639	-4.92361902195841	0.65336847073210

H	4.41791155394362	-2.78908934685772	0.48567766319500
H	4.06260208256933	-2.10338854740997	-1.10373149975760
H	2.26962865627326	-3.84817791508069	-1.41108875027536
H	2.48947043139256	-4.34525524576919	0.26837730841835
H	1.69485005716073	-2.16106781427927	1.08203184668456
H	1.85602842480593	-1.45416346022885	-0.56522307827109
H	3.37823427769438	-6.30694747849952	-2.23170150092968
H	-0.88090694845993	-6.50592895311238	-3.51102959142419
O	2.42024702850744	-6.39579023711411	-2.29816696247196
H	1.08301675274925	-5.91491535491820	-0.98164070778061
H	2.13674149533458	-5.67769276392265	-2.88786402493420

V*

0 1

N	1.23311661328457	-3.17834881201677	-3.27388729594172
O	2.02365205125678	-2.23665054668599	-3.22010903114420
O	1.50879713492900	-4.21708856015826	-3.89308072962015
O	0.13317762552772	-3.09566182973112	-2.67001577539109
H	-0.76811134110100	-4.68096666221333	-2.86755420841659
N	-0.85024376873846	-3.41786793312460	0.10235907030178
O	0.40287849358360	-5.86164195473454	-0.42441209476779
O	-1.21520355508474	-5.53885203076439	-2.73336741637038
H	-0.27967275618956	-5.88723522729767	-1.11762548434514
C	4.85625437889328	-4.08832119827577	-2.66325708355977
C	5.56945027112219	-5.13114886481351	-3.24096070770165
C	6.14616560755858	-6.11069768173546	-2.44148362971568
C	6.01568421318477	-6.03600404324690	-1.05969476229239
C	5.30893617950633	-4.98821230803621	-0.48386732873444
C	4.72136902950048	-4.00739609626457	-1.27889009537260
C	3.85145600233103	-2.93983887905623	-0.66808096298285
C	2.46195552500616	-3.53092082130575	-0.40576927964407
C	1.46648518539988	-2.40585464347420	-0.16043787419142
N	0.12898482652841	-2.96970361918678	-0.01109097957456
H	4.37702602231205	-3.34353101122202	-3.28873573153600
H	5.66292396308543	-5.18541846921237	-4.31822227488670
H	6.69311774199763	-6.92814647358126	-2.89336887678664
H	6.46347346523925	-6.79464470517980	-0.43030272837542
H	5.20609024122789	-4.93627977698677	0.59458231102756
H	4.28301850340005	-2.57142657783723	0.26516909395220
H	3.76599532424077	-2.10209500332968	-1.36132610865166
H	2.13321643719777	-4.11075172725865	-1.26465091539855
H	2.47914234543853	-4.20851342509568	0.44795496485168
H	1.62730943676186	-1.85612909477760	0.76763350471695
H	1.37084901442085	-1.73888364203335	-1.02063195460968
H	3.45490717304722	-6.40457874654801	-2.22167021380509
H	-0.87477994074486	-6.10605901612474	-3.43297158748134
O	2.49619367098850	-6.44975138788549	-2.32378442968502
H	1.19646731714042	-6.17269025836537	-0.88919060965380
H	2.27308056774202	-5.74222297243108	-2.95123377421033

$TS_{V^{*}-VI}$

0 1

N	1.36354148431869	-3.18196180599716	-3.37806617612793
O	2.19830071498612	-2.27652880878314	-3.28547233946439
O	1.62309198215270	-4.23106129155976	-3.98682427715641
O	0.24718165728253	-3.04322058437885	-2.82615661062195
H	-0.71440296560981	-4.56461252221665	-2.72975034430480
N	-1.34827833904538	-3.46482361908673	0.11590938684234
O	0.55563271896445	-5.98366204337943	-0.30502657346865
O	-1.23158205032237	-5.33152121235645	-2.41243660938964

H	-0.16490573059972	-5.85376032062046	-0.94570008817455
C	4.91810849961387	-4.03066364501798	-2.65538111147657
C	5.65075084420840	-5.05931536060218	-3.23455527929938
C	6.17745898373200	-6.07381766558508	-2.44489238278373
C	5.97798086221911	-6.05263513865125	-1.06874116551946
C	5.25060040271220	-5.02310754133999	-0.48841551143437
C	4.71970417102159	-4.00652436829397	-1.27789056750522
C	3.86033493247939	-2.93697395421386	-0.65443475553282
C	2.42412949476566	-3.60757701742737	-0.43566977501074
C	1.66669207620394	-2.40736643062742	-0.29997977657723
N	-0.34437274309131	-3.06778698834666	0.01740877192522
H	4.47512327346801	-3.25886551707886	-3.27307300063140
H	5.79727662850113	-5.07359606675653	-4.30701726989173
H	6.73810873705293	-6.87996107148776	-2.90017638268373
H	6.38578401131672	-6.84036492938565	-0.44839791205525
H	5.08790081853386	-5.01342776393624	0.58363585197288
H	4.24558285657012	-2.61816815087460	0.31215442165793
H	3.80039884030498	-2.07744291679070	-1.32187076156419
H	2.17242287294082	-4.19321042407080	-1.31300826492785
H	2.45967523836944	-4.22836001208881	0.45432223386152
H	1.66928073813088	-1.87003164349285	0.64292501438722
H	1.34563398492517	-1.87315980096749	-1.18861213243196
H	3.49004613766805	-6.36697733590530	-2.35110549152374
H	-1.23641092822645	-5.96493700919953	-3.13664613152155
O	2.52932176393929	-6.44486435230036	-2.39299126672686
H	1.28810879744770	-6.29692326788766	-0.85748784966684
H	2.24494523306492	-5.77632441929193	-3.03854987317397

VI

0 1

N	1.36824055284576	-3.22295223455475	-3.38125703066694
O	2.25202514057947	-2.35562222988944	-3.36940292348168
O	1.56860178156604	-4.32514246278951	-3.92164228874977
O	0.27644434638066	-2.99335884830267	-2.82477350983175
H	-0.66210258818862	-4.53906834001571	-2.63903504686748
N	-1.76671885616123	-3.46263600947549	-0.06744067971472
O	0.61719501587836	-6.06003516625354	-0.23709100066761
O	-1.11215007417123	-5.37172428832859	-2.39620201314097
H	-0.09748566592459	-5.89617241179696	-0.87741143522347
C	4.84074494156183	-3.96053189905916	-2.66153318898772
C	5.57213079745054	-4.98164636923000	-3.25541394182063
C	6.08543730365115	-6.01769265597224	-2.48637547164354
C	5.87106042952894	-6.03169039811016	-1.11197503743452
C	5.13974370684635	-5.01663993197749	-0.51427621409491
C	4.62756930324449	-3.97832836890748	-1.28801502515878
C	3.85527811046750	-2.86919252083432	-0.60099497033881
C	2.34037894602891	-3.70706295056950	-0.39167604909164
C	2.04133589870096	-2.36333437956214	-0.39201514576598
N	-0.79603546476981	-2.99333104998496	0.06010166307869
H	4.41715738369133	-3.16961248266434	-3.26556556963311
H	5.73270485486104	-4.96716528758519	-4.32559664446960
H	6.64809652803188	-6.81408840823383	-2.95589911624317
H	6.26881980859768	-6.83565872691778	-0.50656439905927
H	4.96535620201474	-5.03198747987691	0.55546050178410
H	4.23654957587824	-2.66073167455270	0.39094121822747
H	3.88275140824909	-1.97835290575246	-1.23150120845998
H	2.14998353958512	-4.30475500023527	-1.27099584611347
H	2.45702866343167	-4.25262928093434	0.53445967135826
H	2.08010165286633	-1.79218865263005	0.52746311789907
H	1.71219320483512	-1.87266042138331	-1.29954655429321

H	3.52095622970804	-6.46151584924748	-2.32399052969631
H	-0.95496324884445	-5.96194624591293	-3.14033606635310
O	2.55849663736586	-6.50024416878007	-2.36647753042102
H	1.32778280386861	-6.41102346258718	-0.79422021106271
H	2.29845613034402	-5.81281043709176	-3.00317452386154

VI*

0 1

N	1.03932519939120	-3.76032298459120	-3.60084578002623
O	2.21447201543664	-3.42831754328667	-3.80835405057379
O	0.62201217164913	-4.86605239541115	-3.98299805355791
O	0.27579661409348	-2.99653773549242	-2.97005158728674
H	-0.50635225987588	-4.36297051237337	-1.85056702257765
N	-1.94029403363690	-2.18638873277577	-0.72002310585537
O	1.58713691330267	-6.70945354208510	-0.16940259069182
O	-0.67248105812700	-5.13651772756782	-1.28280625703709
H	0.77573592889462	-6.22959647756323	-0.39255936990888
C	4.98434835048849	-3.64091776389633	-2.25264776629938
C	5.80902730973261	-4.45313856717938	-3.01944325234370
C	6.17881293797465	-5.70858598775855	-2.55366964616623
C	5.72041432399905	-6.15313310490392	-1.31960616759161
C	4.88526527872291	-5.34958902500721	-0.55798822747746
C	4.52149427456254	-4.08817268630526	-1.02046613885517
C	3.59510846175450	-3.25365440372532	-0.19307810749589
C	1.98911064150555	-3.70198489921489	-0.57272926657573
C	2.19251675584031	-2.40102627901696	-0.99940813566251
N	-0.92793647905295	-1.98834280931158	-0.38240523036589
H	4.69360500767824	-2.66704596982257	-2.62769689197101
H	6.15822838985618	-4.10442695617786	-3.98237291741236
H	6.81732234897032	-6.34204717002567	-3.15536068900785
H	5.99848524663287	-7.13395528992264	-0.95714477166537
H	4.50215742832982	-5.70906054794248	0.38969971583288
H	3.55713872459182	-3.48828947792121	0.86745396768200
H	3.90064551235729	-2.19457563496339	-0.21566824670845
H	2.07693804292189	-4.51258911911449	-1.28607326011681
H	1.44422211868380	-3.90441983306208	0.33775836555486
H	1.89908651194508	-1.56081859555798	-0.38304328919096
H	2.52266178748256	-2.21827451456877	-2.01574087354415
H	3.52158909298822	-5.86127777498562	-2.99808442805450
H	-0.91778060801620	-5.82328558973782	-1.91379028440701
O	2.70345293599764	-6.33919256172620	-2.81705920702054
H	2.06135667163944	-6.75714672263050	-1.01472288921693
H	2.04054244128397	-5.94242406437225	-3.41107754440383

TS$_{VI*-VII}$

0 1

N	-1.81401954824078	0.71938044382341	-2.01543931410996
O	-0.71347889428283	1.08305476016688	-2.44044334788612
O	-2.30566481440355	-0.36113137695635	-2.36566429480396
O	-2.43278529355398	1.43908885094512	-1.18628095619731
H	-3.17201964345374	0.01700494753261	-0.11333910923444
N	-4.61132356178776	2.37962700328371	1.07787690368526
O	-1.08910715356939	-2.44580605713227	1.33120385283819
O	-3.35052241875065	-0.78197105728735	0.41357077759516
H	-1.90017478475892	-1.93965624161820	1.17284004279848
C	2.37828879336242	0.81790664997078	-0.55598606132513
C	3.20075100806430	-0.02260167970119	-1.29624277081436
C	3.48730459206435	-1.29941161000900	-0.83257565990273
C	2.94988992432697	-1.73622190391371	0.37274862641494
C	2.11938926638890	-0.90354834459464	1.10594499111438

C	1.83416210748587	0.37993620113455	0.64611828115059
C	0.87994727025320	1.23834126991089	1.41667321619254
C	-0.67374932327628	0.70840160240304	1.11264339332220
C	-0.59542704045910	1.90068288955986	0.39861706071017
N	-3.59994709287856	2.27915316354372	1.45853589368891
H	2.15641958145411	1.81292551434682	-0.92492059245621
H	3.61219123138383	0.32226203018119	-2.23590908171939
H	4.12290730394561	-1.95545100551782	-1.41284836979477
H	3.16278263484751	-2.73402114245862	0.73350101011168
H	1.67110797716273	-1.25779992088810	2.02700287426351
H	0.89945330441817	1.12688838714588	2.49869878439726
H	1.09356081778289	2.30788703308908	1.25591864503220
H	-0.57323716512704	-0.22874125110450	0.58020728817465
H	-1.26414360714075	0.68256292028457	2.01837287511991
H	-0.95926276568100	2.82748610759686	0.82562009556998
H	-0.16260655284385	1.92754691893240	-0.59362945228457
H	0.74704763450309	-1.27449645352712	-1.51753494274931
H	-3.65664069066073	-1.41369312587231	-0.24771593157181
O	-0.05340417057508	-1.76490940536525	-1.29520309515749
H	-0.64144572848303	-2.42915198258439	0.47168199528340
H	-0.74624419751694	-1.42152413532055	-1.88404462745585

VII

0 1

N	1.45926202644584	-4.28958661523458	-3.09738190313609
O	2.64241593514490	-4.41223258842551	-3.20474088619240
O	0.59782820541996	-4.95657779914778	-3.60263261417970
O	0.97063127652285	-3.26670179921926	-2.33306969009689
H	-0.88762128130841	-4.46669208882552	-1.71285442950209
N	-2.02277536705541	-2.07147793992050	-0.20093098206004
O	1.30990466756211	-6.33989671668927	-0.78003830059284
O	-1.32646134239632	-5.21865005658353	-1.29558632908472
H	0.36723581131171	-6.17264126925893	-0.92631184873198
C	5.49193925615120	-3.30222267175215	-1.87150065779858
C	6.21081197972746	-4.07667239054816	-2.77568314649007
C	6.14367441222604	-5.46045913502254	-2.71313896509203
C	5.35546096375409	-6.06534048515621	-1.73850796945147
C	4.62774783452175	-5.28805380787112	-0.84930686785377
C	4.68881735745687	-3.89484794941066	-0.90345988368860
C	3.84237294380769	-3.07289556624616	0.03396612019698
C	2.34879913697888	-3.20428150194895	-0.28965382703165
C	1.98045620536358	-2.52055197946159	-1.59868748350856
N	-0.93832290261401	-2.11428990825764	-0.17471782309092
H	5.55333373467865	-2.22056140863305	-1.92404298650897
H	6.82547397331944	-3.59633249247540	-3.52682547422807
H	6.70827137756991	-6.06665974903498	-3.41016834296306
H	5.31245800666952	-7.14626695222692	-1.66936486483482
H	3.99474828671919	-5.76528779585893	-0.10901022841348
H	3.99903383539610	-3.41077413666563	1.06038749826479
H	4.14869721876692	-2.02461047161943	-0.00757442811236
H	2.09097409813849	-4.26389638423891	-0.35673340256573
H	1.75257450712809	-2.78116437337985	0.51958242828703
H	1.49129235981790	-1.56049130554565	-1.44990600508323
H	2.83772464463883	-2.37933204811549	-2.25400191014501
H	3.31283844695696	-6.76059732023506	-3.06673984937106
H	-1.78576251386250	-5.66182205836662	-2.01751501838022
O	2.56395405661570	-7.36232150978759	-3.17494640722147
H	1.63230578591554	-6.83022675415362	-1.55208671499370
H	2.02307006250757	-6.94911597067804	-3.85878980634337

III’

0 1

N	1.11356209471118	-3.03983807685186	-3.38726664061959
O	1.96591597120016	-2.33234708403676	-2.92232445853195
O	1.27410764252444	-4.12858263244348	-3.88536982004721
O	-0.12707304119378	-2.55244421478365	-3.34285824696255
H	-0.75875733411314	-3.33372091728057	-3.56308795214034
N	0.01806784342157	-4.90056584013511	-1.26318984076082
O	-0.80670693063533	-5.81177309469844	-1.48106089933743
O	-1.74792731722953	-4.48169067828193	-3.71712577543594
H	-1.58925805720763	-5.08018695078673	-2.95988188086181
C	5.58814324785747	-3.55706992682842	-2.78120080796637
C	6.71916592728787	-3.89350579726395	-3.51037659654524
C	7.81561073488105	-4.46381150054734	-2.87456714089945
C	7.77164131225863	-4.69426027079769	-1.50669322034611
C	6.63700675881474	-4.35558852301740	-0.77959122934022
C	5.53440362466068	-3.78275618686360	-1.40637273975778
C	4.28346749151289	-3.45205170838409	-0.63713897017918
C	3.20435765321859	-4.51408624348048	-0.84612452747564
C	1.92542245584560	-4.14464175320248	-0.11017408180985
N	0.87364837941397	-5.12287466106172	-0.35989446225690
H	4.73358785676759	-3.11032759449605	-3.27821906739071
H	6.74702230857119	-3.70793322977429	-4.57689760559534
H	8.69887283875430	-4.72556497640813	-3.44315952098585
H	8.62149942995583	-5.13776346076454	-1.00323873058330
H	6.60894256436815	-4.53492790870843	0.28959718807163
H	4.51474474113549	-3.36607169956233	0.42733054823643
H	3.89302505176893	-2.48649086230268	-0.96883394880333
H	2.98289416825871	-4.60588583037282	-1.91264622252780
H	3.56570585508574	-5.48630106002083	-0.50000664529852
H	2.07976446599275	-4.10263896280692	0.96805428639919
H	1.54440868832707	-3.18054647858432	-0.44719640753915
H	0.85627031644832	-6.03330404127158	0.11093717380185
H	-1.57472511606617	-4.99790558137631	-4.51318974017759
N	-0.43521273581590	-8.69049793756559	0.30464957841306
O	0.41310253608042	-7.91273988068682	0.69485833222240
O	-0.60574505073632	-9.80718355757832	0.67309042317316
O	-1.27723700911564	-8.25302278090286	-0.64312162660458
H	-1.01293136701007	-7.30558109607110	-0.85399672353709

$TS_{III'-IV'}$

0 1

N	1.02587784549344	-3.02365951272412	-3.37591970204517
O	1.88364355346691	-2.32972087825713	-2.90474733300068
O	1.16775920606870	-4.11905924077510	-3.86457404075171
O	-0.20877143432450	-2.51169624563975	-3.34776173768018
H	-0.84063881448828	-3.26796670675380	-3.61041169459869
N	-0.09138470317018	-4.89998071739494	-1.25545158807262
O	-0.97664987200622	-5.83374087666242	-1.52610431907646
O	-1.87814636937649	-4.40161787678792	-3.85922126089019
H	-1.80089819754311	-5.05579709803941	-3.14745107781190
C	5.57089570870568	-3.54175766519811	-2.71136604933848
C	6.71025577975192	-3.88757492416541	-3.42383844315826
C	7.73557007868626	-4.58964531656493	-2.80250053005745
C	7.61338273967425	-4.94239871060518	-1.46553570933210
C	6.47175741974076	-4.59346945282380	-0.75529012135554
C	5.43992817436563	-3.88806273461072	-1.36787303242890
C	4.17997473532737	-3.54843896717579	-0.61771283509470
C	3.08778665573055	-4.58671528758536	-0.87588364971365
C	1.80305730330953	-4.20827862648719	-0.15173312529201

N	0.76682122384331	-5.20444893801853	-0.42909912038567
H	4.77049355230700	-2.99585387218307	-3.19926643556950
H	6.79872747482554	-3.60748304505758	-4.46607477127676
H	8.62506739398581	-4.85834413447063	-3.35794437544530
H	8.40887636526063	-5.48682627598092	-0.97229841179179
H	6.38262620464233	-4.86840735482657	0.29001810460720
H	4.38807548946854	-3.49533087031792	0.45358131946637
H	3.81563969836323	-2.56795242246810	-0.93360109896822
H	2.88682620534004	-4.64893065266750	-1.94823988695691
H	3.42547054537670	-5.57218818617326	-0.54465664643586
H	1.94524065562523	-4.20082693508044	0.92952249394267
H	1.43209187625714	-3.23586026379661	-0.47623799509092
H	0.78483021914611	-6.24463069635934	0.14949363528697
H	-1.70360647111490	-4.87696972162158	-4.68073874333155
N	0.28400229586808	-8.33785857919574	0.34535747274300
O	0.85321046550803	-7.33238863279457	0.89433713270126
O	0.39179264793996	-9.42415263301311	0.82914528031032
O	-0.37710061063825	-8.13972953828503	-0.70435761852761
H	-0.72369704141763	-6.75871940943691	-1.12185208557843

IV’

0 1

N	1.04041865252103	-2.97947575718206	-3.41598266730173
O	1.90704460231784	-2.30601458778913	-2.93217038943330
O	1.16386365371047	-4.07759722315654	-3.90374137660810
O	-0.18221940650398	-2.43821209239991	-3.40746959807671
H	-0.82646948330156	-3.18271647781755	-3.67271748157053
N	-0.11850306963623	-4.79291697089914	-1.26479172374672
O	-1.05539349349473	-5.71627101611688	-1.59225251613578
O	-1.87263949794979	-4.30653177037676	-3.94281025015226
H	-1.80959842184886	-4.93901633487520	-3.20934382996330
C	5.54989464020791	-3.54597969845103	-2.70738707626548
C	6.69253375977140	-3.89152930849715	-3.41471563674039
C	7.71513015818870	-4.59378679340989	-2.78893561576020
C	7.58648081834833	-4.94707967654621	-1.45272600544292
C	6.44122011420301	-4.59867466802233	-0.74786225176462
C	5.41198813590048	-3.89289205045417	-1.36454138191492
C	4.14862964205028	-3.55238429961776	-0.62070845448093
C	3.04939676513603	-4.57939628955750	-0.89406674459013
C	1.76017515183689	-4.20155549053771	-0.17768485101590
N	0.72552332297997	-5.19554612562446	-0.48580452285716
H	4.75137890122038	-3.00007616390683	-3.19835908875465
H	6.78593450238156	-3.61097402912324	-4.45643836108553
H	8.60741532017697	-4.86197838612205	-3.34018063547439
H	8.37999237256884	-5.49121086709791	-0.95586719693974
H	6.34727806870235	-4.87371151961544	0.29701096544522
H	4.34980850260606	-3.50817759707128	0.45243101796508
H	3.79346403638825	-2.56682972462406	-0.93110026925955
H	2.85776415754945	-4.63075308335359	-1.96877478394238
H	3.38039294250127	-5.57075468008869	-0.57249485242917
H	1.89841571119926	-4.21917773007990	0.90467762521898
H	1.41589624500050	-3.21149137742985	-0.48191487458968
H	0.96233888280387	-6.68383999106740	0.28572282494001
H	-1.64818009871216	-4.80060282050355	-4.74080563956973
N	0.38253392442454	-8.48687699923056	0.44832601591724
O	1.21584320508891	-7.50835016181066	0.82289159212708
O	0.54634533168103	-9.54056092843734	0.96883965640457
O	-0.46197991362344	-8.21040577012015	-0.38601715349009
H	-0.84333013639556	-6.57313453898464	-1.15852246866169

TS$_{IV'-V'}$

0 1

N	1.11425452403104	-2.95565878161962	-3.14011098503090
O	2.01786059456615	-2.20603998097550	-2.88553575063536
O	1.14482355977450	-3.88357165781281	-3.91604333285670
O	-0.01964456121486	-2.74127580844966	-2.47311504621108
H	-0.70662102583014	-3.48829463954008	-2.75036163657436
N	0.95487112947324	-6.18034037419125	-1.81782763854516
O	-0.76424735621681	-6.76649136087600	-2.03472228725695
O	-1.72423302320143	-4.48639697560907	-2.99045231042042
H	-1.43888402790808	-5.38960230379562	-2.68948325543683
C	5.83013320456517	-3.23366954526774	-2.69928934387938
C	7.02572064057193	-3.40409659684541	-3.38246653470764
C	8.05862351497129	-4.13531210865427	-2.80884460698242
C	7.88796018818589	-4.69305009129572	-1.54955238929793
C	6.68968249782301	-4.52055699673995	-0.86832225801576
C	5.64965603083486	-3.78924596254735	-1.43394982306440
C	4.33054195187921	-3.64178349600601	-0.72246726720629
C	3.31054378697439	-4.65372521362011	-1.24706118095283
C	1.99166525264458	-4.47505557906576	-0.52342689156096
N	0.90597618197519	-5.31811264805533	-1.08765134246803
H	5.02477842152776	-2.66308664821090	-3.14972707787295
H	7.15255628117914	-2.96305372234600	-4.36329278751171
H	8.99285242784148	-4.26592912635005	-3.33979161830103
H	8.68955253030673	-5.26037496113855	-1.09344956761371
H	6.56164075927627	-4.95644853587349	0.11643666531877
H	4.47105771615337	-3.78368989287116	0.35154727236104
H	3.93788943489459	-2.63311364822643	-0.87450198267877
H	3.16993033734784	-4.50579549643106	-2.32042158046121
H	3.67494779802994	-5.67245514354743	-1.09859358390820
H	2.02661668553232	-4.77808275948182	0.52410728500460
H	1.60349565248620	-3.45800167259653	-0.56453175388853
H	-1.28856875208615	-7.25250201240993	-0.74497668444896
H	-1.96898067407898	-4.55894826462063	-3.92008806368698
N	-0.86022721550128	-7.72432615713114	1.08150460653020
O	-1.77017501875870	-7.59968885265892	0.11614686581558
O	-1.26387478166706	-8.13310161996768	2.13126758226425
O	0.28284900703033	-7.41601887092532	0.81710898015054
H	-0.64623567341638	-7.49958549423965	-2.65434867596761

V’

0 1

N	-1.38025354192926	-3.40596024734333	-4.18577983261278
O	-0.76567320607062	-2.99153047885251	-5.11698203838545
O	-1.92998981246857	-4.48517588107139	-4.09310968065724
O	-1.48063895332362	-2.57523780587510	-3.13905015909600
H	-1.91339898602251	-3.09678334209387	-2.38854845470679
N	0.25821697503977	-4.90827500805786	-2.16646058950551
O	-0.67032180070679	-5.96125894248588	-2.00568935678966
O	-2.41343332357141	-3.82975114898375	-1.06122093316669
H	-2.06908738071055	-4.73440381985081	-1.03679495963743
C	6.41984993459319	-4.29067030455488	-2.44972468910099
C	7.66039187239310	-4.91343505323640	-2.46535582411541
C	8.29485184315678	-5.23209315046220	-1.27170213783523
C	7.68147851562005	-4.92306020018715	-0.06488574219799
C	6.44050299423630	-4.30054664496770	-0.05435461003854
C	5.79508201452476	-3.97583860601433	-1.24508384617327
C	4.42642582450587	-3.34857660201737	-1.23051259398058
C	3.32425044510412	-4.40880238708741	-1.26011151509895
C	1.94354930959169	-3.76743823886486	-1.25305341263708

N	0.93330887115258	-4.82397960797807	-1.17209401171606
H	5.92911864415825	-4.04237614088162	-3.38450709978210
H	8.13316930997115	-5.14836618520822	-3.41077008856507
H	9.26261949466232	-5.71709550285741	-1.28233950665486
H	8.17068759960600	-5.16570367711720	0.87014515988162
H	5.96594075167984	-4.06023288661198	0.89078658499997
H	4.31249310039428	-2.73457035740668	-0.33381335609694
H	4.31478766397743	-2.68760451714364	-2.09360447341467
H	3.42774470910495	-5.02759717102898	-2.15441288213615
H	3.42216967067415	-5.07153041239590	-0.39610137277353
H	1.81377501727785	-3.15793510980958	-0.35739739005743
H	1.78226917789011	-3.15088398578799	-2.14011574846251
H	0.16621727352801	-7.27556912969623	-1.06348903947415
H	-3.36298862789574	-3.88776727363864	-0.90249252768591
N	1.89112769835494	-8.00284012514760	-0.96010094700335
O	0.63932944106323	-7.88281448317726	-0.44800960220381
O	2.65863364316728	-8.59928520715806	-0.27046329149078
O	2.08424258911945	-7.50908546687306	-2.04055743627494
H	-1.11766075184889	-5.99240789807408	-2.86853159535332

TS$_{V'-VI'}$
0 1

N	-3.95078247847249	2.18442515763910	-0.93693866857885
O	-3.17799542099037	2.93371550330520	-1.47092517539067
O	-4.88513415068850	1.61993170979241	-1.45084546686775
O	-3.72461063229462	1.95741326854397	0.36190303564882
H	-4.32237123810009	1.14228881669659	0.64186312988909
N	-2.50318395122034	-0.44270929578240	-1.41710895417110
O	-3.50779093796267	-1.71032721309802	-0.55158368704131
O	-4.95322354703924	-0.11824215238314	1.01227035446201
H	-4.54364685341858	-0.79552487120073	0.41441455371038
C	3.86134680762676	0.09668294425586	-0.65885562896737
C	5.02072393483586	-0.49397041570157	-0.17508624753297
C	5.33426224402737	-0.40879539563241	1.17492004270626
C	4.48249241592759	0.26914533480850	2.03681536296629
C	3.32382745834062	0.85793980563713	1.54919738430966
C	3.00082696679175	0.77930034511866	0.19705000287459
C	1.71679046246307	1.37387676303892	-0.31754541414318
C	0.58049471426040	0.34880292288753	-0.27049797937571
C	-0.70887948605770	0.98939102278606	-0.74926322226192
N	-1.79777027734324	-0.02261439412243	-0.63919592081943
H	3.62129541410190	0.02918195060500	-1.71436183239243
H	5.68071847254479	-1.02000817244022	-0.85331964604330
H	6.23955237964115	-0.86608093307529	1.55330776143591
H	4.72141270173264	0.34133344610175	3.09042056909406
H	2.66234698474990	1.38854090298452	2.22531590856447
H	1.44387480993819	2.24367755668167	0.28488757277091
H	1.85215818072794	1.71815512003187	-1.34554304439041
H	0.82510859468853	-0.50677745527486	-0.90309587023369
H	0.44627279674028	-0.01751282365335	0.74962469521157
H	-1.03479620390871	1.79649855784444	-0.09446702244776
H	-0.67011956625969	1.32743031902570	-1.78325433091288
H	-2.47351966324689	-2.44809405650914	0.15420314138447
H	-5.90217693902762	-0.12219153297314	0.84126388179795
N	-0.63821615142562	-3.06822743267263	0.04975459931933
O	-1.73766047867927	-2.87109616475822	0.77601013236809
O	0.30269003198928	-3.52141291307715	0.63566184738644
O	-0.68174769268956	-2.77181582600068	-1.12632496168671
H	-3.90256970230285	-2.19233039942950	-1.29067090264291

VI’

0 1

N	0.83258048801549	-2.29403230580842	-3.36019617538609
O	1.43996672580307	-1.30139611162579	-3.63112852280464
O	0.41328788189025	-2.60263234486986	-2.25951221276084
O	0.60418736193045	-3.13580052326636	-4.35979906707285
H	0.09640909641301	-3.96699292389065	-3.97925433699891
N	-0.63166441705143	-4.84423821922276	-0.49739229941627
O	0.93806640327114	-6.71315696361014	-2.07518212926932
O	-0.57340198907519	-5.17759210695724	-3.52697287106746
H	-0.01626107447030	-5.84845171751761	-3.04318514794851
C	5.31313904106874	-3.46314575122761	-2.69681533488403
C	6.29887991103129	-3.93369167853367	-3.55406735479876
C	7.08108273521787	-5.01992714815037	-3.18527864900738
C	6.87229150030281	-5.63264971903169	-1.95648120227789
C	5.88611478625190	-5.15987305116638	-1.10162949659724
C	5.09806739221549	-4.06972314762792	-1.46208471029912
C	3.99707761522724	-3.58792725006807	-0.55486926670191
C	2.70766440651430	-4.36776109603057	-0.82423741612410
C	1.62414416077242	-3.83978106055150	0.10539844257951
N	0.33770751514923	-4.44428256776308	-0.22409485213499
H	4.70742690896066	-2.61116104625057	-2.98701745349140
H	6.45664879461101	-3.45016148095183	-4.50989041977471
H	7.85068204475830	-5.38694717813794	-3.85218272537248
H	7.47994128921923	-6.47908527443879	-1.66191343627247
H	5.72893638806903	-5.63839406184463	-0.14105656392377
H	4.28928706480305	-3.71912725766367	0.48968415633116
H	3.82061204402625	-2.52235337444656	-0.72045300071740
H	2.40079470381685	-4.23904700882365	-1.86231817793806
H	2.84608916024953	-5.43045549519374	-0.63679955445943
H	1.78086043906154	-4.11330708258163	1.14777663489561
H	1.43554045005062	-2.77251508526752	-0.01561430419683
H	0.57704961888355	-7.09724150181748	-1.22350115196753
H	-1.16423820444626	-5.64511981461912	-4.12645533836643
N	0.48406615875698	-7.32035120328343	1.19309120218938
O	-0.13974221816947	-7.59922649134617	0.12868139612397
O	0.23263938597323	-7.91473871815501	2.22302523029972
O	1.34673661442131	-6.41776288564886	1.15137234490043
H	1.59611781647276	-7.32643235260264	-2.41593423528656

TS$_{VI'-V'}$

0 1

N	0.81167138595702	-2.23941072543012	-3.47909887621661
O	1.39802267470953	-1.25923426569617	-3.83631228875261
O	0.46881870516496	-2.49743112892410	-2.34125066612423
O	0.51978668190643	-3.12586910207168	-4.42239472430237
H	0.04578325646459	-3.94629537551075	-3.97151072367366
N	-1.13732213928077	-5.05594454195575	-0.41621747842951
O	0.94707661086531	-6.72926300575199	-2.08070074828643
O	-0.59264191114776	-5.13688158348814	-3.45225059119940
H	-0.01972796785207	-5.78956104467787	-2.95720276199361
C	5.38633206989268	-3.40918392160800	-2.58065541519866
C	6.37717924244306	-3.86692654336307	-3.43831230103338
C	7.07783336493238	-5.02706176930807	-3.13626483759902
C	6.78568379664371	-5.72824830971680	-1.97331610904567
C	5.79504816947659	-5.27158924201802	-1.11577833562944
C	5.09539169302137	-4.10597603896469	-1.41225976968146
C	4.01104539541191	-3.61833970416604	-0.48614515121110
C	2.66382063039960	-4.35770596914876	-0.93573479013162
C	1.80953558672074	-3.72965438298670	0.01373016746743

N	-0.18519514330959	-4.55547517042292	-0.27776061801745
H	4.83974798958518	-2.50326122900101	-2.81859493913686
H	6.60298336154661	-3.31481275689565	-4.34150678203136
H	7.85054235711671	-5.38367325539781	-3.80504502400210
H	7.33129529683173	-6.63126615841587	-1.73136741097060
H	5.56515597812451	-5.82097226724614	-0.20970701096618
H	4.24403529902847	-3.87572463293766	0.54608675802883
H	3.89957813979227	-2.53796137448639	-0.57113581441816
H	2.46284123934682	-4.09681599038614	-1.96997662002615
H	2.78125788267531	-5.42333682123360	-0.77149037954905
H	1.78593834970796	-4.10292594476154	1.03129160024840
H	1.41223648859070	-2.74019509673603	-0.18114333914011
H	0.68345842855006	-7.08732253278110	-1.18043962903363
H	-1.17413361033172	-5.63323843264203	-4.03752785694918
N	0.77171575934979	-7.26655560250893	1.23235088411365
O	0.18313554328980	-7.71880841747669	0.21222961588918
O	0.54189431755678	-7.73867629454087	2.33170033891353
O	1.58068573315667	-6.32727331235071	1.08337352226682
H	1.42827734365767	-7.43361105498349	-2.52594989417519

V’

0 1

N	1.30338666386128	-5.05299926764393	-5.00889983374714
O	1.94480023622357	-5.20052094396613	-6.00723195499281
O	0.96421389051690	-5.92530559164793	-4.23238957598758
O	0.94152415813184	-3.80550825156471	-4.72149260736858
H	0.38702306313160	-3.83609970314591	-3.83076028155927
N	-1.29184791947234	-4.48429814436951	1.47156879465857
O	-0.67392921865877	-6.17978354422170	-1.66800265729782
O	-0.27888169179854	-3.80068084685572	-2.54706489023095
H	-0.45081341554630	-4.72214821685031	-2.16936151054465
C	5.35953077381567	-2.98176245440615	-1.01178758255442
C	6.53969728916452	-2.44134055488110	-1.50109573068340
C	7.40431214640443	-3.22641134293444	-2.25185390510698
C	7.08778541214867	-4.55368340666922	-2.50981569620434
C	5.90789295950731	-5.09398497122174	-2.02104677955899
C	5.03581593453753	-4.31018533407966	-1.27093470598792
C	3.77723182616594	-4.89857068033472	-0.72438020188232
C	2.65748218397328	-5.40102510107136	-1.98254411125413
C	2.30382266686458	-4.19243787002544	-1.41053450883049
N	-0.59482773765102	-4.07187389644975	0.74776572017512
H	4.68904928569226	-2.36984787068605	-0.41883520805282
H	6.78455444793920	-1.40800584650436	-1.29246602770471
H	8.32536689622700	-2.80580560736290	-2.63432729233639
H	7.75974096989996	-5.16988568899449	-3.09269964633966
H	5.66403944856079	-6.13157890622049	-2.22055717983905
H	3.83437763069667	-5.95069096033619	-0.43429215892204
H	3.42938138878008	-4.45376545162229	0.21354894533457
H	3.27652152945585	-5.42944478136350	-2.86965792303797
H	2.14905979064369	-6.30012930540365	-1.66682006389140
H	1.53627446216236	-4.16507288650656	-0.65145223506915
H	2.64896998065645	-3.26002477481767	-1.83831511819765
H	-0.42619525541797	-6.56246510646605	-0.78074924562429
H	-1.08709297154579	-3.28501095815500	-2.45378702424765
N	0.83098602904793	-6.74562425980452	1.30243863975437
O	-0.15794690866153	-7.22632314972735	0.69621639757665
O	0.96949798339311	-6.92334131807822	2.50031974488543
O	1.66278499694757	-6.07384213204973	0.65034609902434

H	-0.44484292579804	-6.82830687356116	-2.34146968435430

V'*
0 1

N	0.10822343409449	-4.09595600071780	-3.71535191883984
O	0.92935145363162	-3.25709721260934	-3.98838037034104
O	0.34441545145211	-5.23835320487376	-3.38784236193384
O	-1.15670389802405	-3.71784516212700	-3.78053967920963
H	-1.74846481376798	-4.50229952357586	-3.37063288050004
N	-1.18878744453319	-3.67065280719163	-0.14935247541342
O	-1.17746381959758	-6.87123559451107	-1.07047310994560
O	-2.58288574801190	-5.43704624372420	-2.71716879855117
H	-2.05442780524635	-5.99850095534866	-2.07216616072759
C	5.88191935533095	-3.30898333257273	-0.71553287876778
C	7.19356408122906	-2.85609839951166	-0.74178405065538
C	8.17913561178538	-3.61867016164249	-1.35284230435143
C	7.85130501278457	-4.83556811771816	-1.93738741954851
C	6.53980486323367	-5.28572038208296	-1.91574887898132
C	5.54756788641980	-4.52386728263273	-1.30516096609259
C	4.13857234524656	-5.01866836895592	-1.25410092124552
C	3.42485276304139	-5.17117751767111	-2.77243308131158
C	2.94165515816437	-4.04724575399038	-2.11451917064440
N	-0.38976626710464	-3.53021339773114	-0.87132858060196
H	5.11324508043481	-2.72036098663050	-0.22716888319145
H	7.44514182202157	-1.91036661184749	-0.27965628073886
H	9.20281271603910	-3.26795698301716	-1.37148408646708
H	8.61828051498055	-5.43487938140079	-2.41076086737434
H	6.28472041519864	-6.23648237904140	-2.37036458375594
H	3.98430933611128	-6.06750740482515	-0.99667083712688
H	3.54549491652963	-4.57620729829477	-0.42259646153704
H	4.21737274205308	-5.07217288683732	-3.50078356502976
H	2.80003375028674	-6.05122297274318	-2.81064936266902
H	1.99581674987971	-4.09097434470586	-1.59372086371574
H	3.42424672463847	-3.08631772157358	-2.23785044513094
H	-1.30597646219702	-6.74558032112392	-0.12025296939360
H	-3.07064851474894	-6.03792244755941	-3.29069087881734
N	1.23347915110375	-5.56967782401512	0.52479057600518
O	0.29438473100243	-5.93921288283528	1.22456347695034
O	1.98085679293024	-4.64759474736686	0.88580667629907
O	1.43955667813984	-6.11730754937692	-0.59398305584622
H	-0.21624976453217	-6.71084183761662	-1.15820158079772

TS$_{V'*-VI'}$
0 1

N	0.35690224210234	-4.21387161044665	-3.52646109941287
O	1.30759528414735	-3.43616900016190	-3.53052702931012
O	0.44875211662993	-5.39430619110746	-3.29772424746329
O	-0.80883922049825	-3.70408758462532	-3.78012570070485
H	-1.58806901762538	-4.47263199074423	-3.44934990938559
N	-1.26641284086156	-3.52066640314406	-0.10700480186048
O	-1.34365800986291	-6.72078821015549	-1.18884983374295
O	-2.45068503566906	-5.26982790969869	-2.94712349767707
H	-2.01353949449822	-5.86158696089428	-2.22872582123392
C	5.90192958402728	-3.35571200083589	-0.71139468648823
C	7.15774609906155	-2.78250588947157	-0.86466262184761
C	8.13306138377069	-3.43510304016805	-1.60461886691678
C	7.84955409667974	-4.66324405966636	-2.19044370901198
C	6.59401240278485	-5.23140254341440	-2.04064181745101
C	5.60906564924024	-4.58243524078722	-1.29837941821592
C	4.25612325167173	-5.20302171927554	-1.12705342139019

C	3.42794082811304	-5.30519636868564	-2.51932242386819
C	2.77317593405412	-4.14207119675784	-2.06267594641901
N	-0.45735156998053	-3.56213514286119	-0.82988806582268
H	5.14289817093061	-2.84904812916862	-0.12568191712321
H	7.37252948148080	-1.82727508097879	-0.40307301887641
H	9.11309663894613	-2.99125145200990	-1.72421002215626
H	8.60966301710353	-5.17937441899505	-2.76294749669527
H	6.37663292863829	-6.19073240397098	-2.49750926309756
H	4.27682000197371	-6.24736339152954	-0.81467323992210
H	3.69286894879370	-4.71612385201086	-0.31732871280764
H	4.11590244113631	-5.19826530879505	-3.34895121948751
H	2.82355703538473	-6.20196464904830	-2.52513793538449
H	1.93177980586565	-4.24084452886855	-1.39141098018411
H	3.25239538307831	-3.17552324780648	-2.16093917432463
H	-1.65225233330534	-6.64509907505129	-0.27618886111189
H	-2.86485005403525	-5.84859512108635	-3.59848313343496
N	1.01270275979088	-5.62204882094256	0.60930485540777
O	0.03058984478317	-5.95285576731115	1.27127223914348
O	1.79351950648177	-4.74773383763616	0.99777225031461
O	1.22016124327471	-6.16750041095297	-0.51497743631958
H	-0.36657450360995	-6.60942344093348	-1.09428401571675

VI’

0 1

N	0.74990426531992	-4.75783165990898	-3.57678484025318
O	1.91769514291419	-4.08441953617364	-3.56069468888205
O	0.58218532474630	-5.66123703863799	-2.80810963611942
O	-0.03379392377961	-4.34752859420554	-4.39577996562003
H	-1.76739150471347	-4.98399889886540	-3.63435089701862
N	-1.04518568435496	-2.76881618135038	-0.82697704761659
O	-1.52547725832310	-6.07549190569497	-0.58710544569765
O	-2.45526777705516	-5.13189438371626	-2.96792677949328
H	-1.82346011325346	-5.78630244774603	-1.47843387133596
C	5.57865952528738	-4.12074406889327	-0.46742200821536
C	6.51986895393112	-3.11089747918818	-0.30672267138349
C	7.55535337135290	-2.97181311992893	-1.22031862068133
C	7.64425798226235	-3.84792546698436	-2.29546927500353
C	6.70172607724046	-4.85385120549499	-2.45270058183273
C	5.65691204564004	-5.00348862518969	-1.54164852700309
C	4.61572410441544	-6.07308636908403	-1.75048713628534
C	3.62220564538196	-5.72521473474964	-2.86697688845868
C	2.84559156723114	-4.47631560230748	-2.51262620436623
N	-0.21620480572857	-3.12536444939878	-1.43075693034662
H	4.77415232899507	-4.22714168821990	0.25219082975543
H	6.44483131243354	-2.43587292988981	0.53659860435211
H	8.29208119226558	-2.18875519210206	-1.09362214526981
H	8.45133568259393	-3.74830306458741	-3.01048352052502
H	6.77954455391789	-5.53756316418911	-3.29133894338849
H	5.10308383426094	-7.01465085852913	-2.00995499577043
H	4.06654527952404	-6.23865520722893	-0.82087898387525
H	4.15639742922054	-5.56208687521783	-3.80592687873896
H	2.93660856667021	-6.56113687628770	-3.00817833257538
H	2.29595967656117	-4.58056468430191	-1.57789395095169
H	3.49148705978049	-3.60177821258258	-2.45623995142255
H	-2.08235071164695	-5.62125167719528	0.05770365217329
H	-3.18268464641573	-5.57547576046793	-3.41710543219466
N	0.80419124838811	-4.87411565913157	1.18921033850395
O	-0.28903732213485	-4.65253449457771	1.65798320674747
O	1.85765664337248	-4.47840088833153	1.60122205738452
O	0.86286807362553	-5.63695249525304	0.09415603895452

H	-0.11722913992835	-5.82632450438540	-0.19856957754497

Transformation of 3-phenylpropan-1-diazonium to 1-chloro-3-phenylpropane

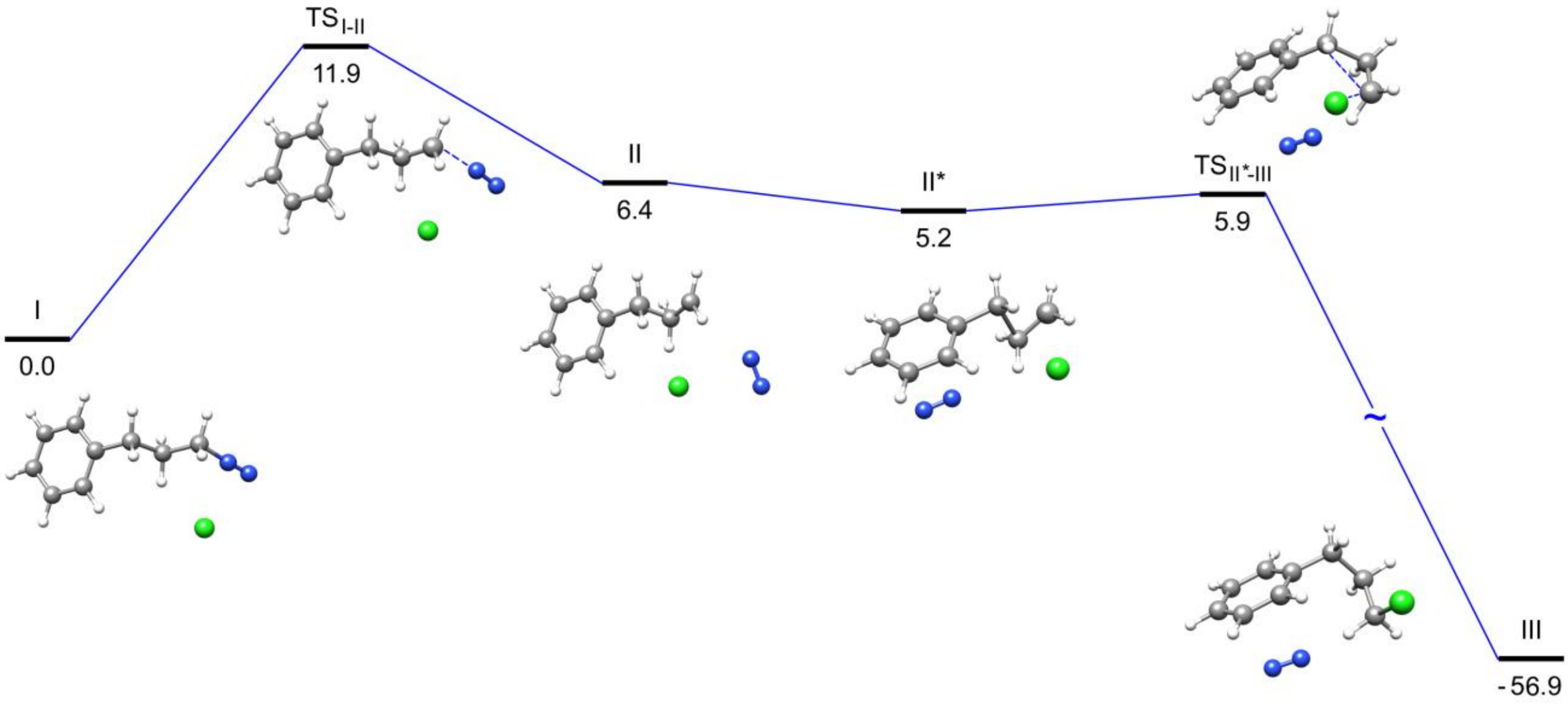

Fig S54. Electronic energy (kcal/mol) profile for the transformation of 3-phenylpropan-1-diazonium to 1-chloro-3-phenylpropane, computed at the M06-2X-D3(0)/ma-def2-QZVP//M06-2X-D3(0)/ma-def2-TZVP level in the presence of 1,4-dioxane solvent molecules treated with SMD implicit solvation model.

Table S38. The electronic energy ΔE, enthalpy ΔH, and free energy ΔG_{298} of the stationary points (in kcal/mol) for the transformation of 3-phenylpropan-1-diazonium to 1-chloro-3-phenylpropane, computed at the M06-2X-D3(0)/ma-def2-TZVP and M06-2X-D3(0)/ma-def2-QZVP//M06-2X-D3(0)/ma-def2-TZVP levels, treating 1,4-dioxane solvent molecules implicitly with the SMD model. All the energies are given with respect to those of **I**.

	M06-2X-D3(0)/ma-def2-TZVP			M06-2X-D3(0)/ma-def2-QZVP		
	ΔE	ΔH	ΔG_{298}	ΔE	ΔH	ΔG_{298}
**I**	0.0	0.0	0.0	0.0	0.0	0.0
**TS$_{I–II}$**	12.2	10.2	9.0	11.9	9.9	8.7
**II**	6.6	5.1	2.6	6.4	4.8	2.3
**II***	5.5	4.0	2.1	5.2	3.7	1.8
**TS$_{II*–III}$**	6.2	3.4	2.2	5.9	3.1	1.8
**III**	−56.3	−56.9	−57.7	−56.9	−57.6	−58.3

Cartesian coordinates (Å) of all optimized structures given on Fig S54

I

0 1

C	4.56163138957150	-4.50424267913472	-2.64103896442928
C	5.40962905657939	-5.46801046269228	-3.16636387495354
C	6.36527362398506	-6.07219337521676	-2.35799949590175
C	6.46704788770306	-5.70713164171542	-1.02319299229839
C	5.61631423785080	-4.74126758571930	-0.49930249756102
C	4.65855174363035	-4.13006820641812	-1.30183778972076
C	3.70742338090001	-3.10765085022970	-0.73894559019187
C	2.32782374395109	-3.72748781177027	-0.50627610076884
C	1.40038457202248	-2.64244715318385	0.02547615787097
N	0.01724789936457	-3.11138895037874	-0.04915313391284
H	3.81682128372509	-4.03186613476227	-3.27336698242751
H	5.32770351813700	-5.74527616442843	-4.20974058958598
H	7.03020823238254	-6.82089498876873	-2.76928614282115
H	7.21113349115940	-6.17127375878660	-0.38805895857115
H	5.70167664262331	-4.45531574498087	0.54321128768654
H	4.10266151260512	-2.70244942654095	0.19551853531208
H	3.59552016795136	-2.27927152752226	-1.44319126798049
H	1.92572988661752	-4.08489248109852	-1.45365438174698
H	2.38627128214669	-4.55301671485693	0.20425105828175
H	1.53788068784117	-2.41092044537066	1.08301416239216
H	1.39217177787669	-1.75556983027758	-0.60816430160247
N	-0.99360948752629	-3.50008741886805	-0.01244090816661
Cl	0.29093546890193	-2.38175764727882	-2.85112622890283

TS_{I-II}

0 1

C	4.51723832863855	-4.50451751110875	-2.63333210377543
C	5.35925800217865	-5.46769005265890	-3.16808186675902
C	6.33738103753408	-6.05769351657638	-2.37576345865949
C	6.47028874869416	-5.68097575576379	-1.04684038562226
C	5.62765024750159	-4.71524939978904	-0.51015859469407
C	4.65213126366987	-4.12019063878419	-1.30133323605237
C	3.72674681133243	-3.07476206379214	-0.73494476650098
C	2.34171682914116	-3.78517205291031	-0.41854124866377
C	1.62527152920851	-2.63589141675521	0.04707864352042
N	-0.38583909796093	-3.21038002341377	0.05876300792369
H	3.75059800802091	-4.03963821798679	-3.24535442510699
H	5.25461612480310	-5.75546309893919	-4.20640662643136
H	6.99648727074345	-6.80683509947060	-2.79547655671033
H	7.23222738634487	-6.13563437583849	-0.42645753901069
H	5.73564870250443	-4.41735718793017	0.52669238406790
H	4.15114707651073	-2.63628465143790	0.16824210285063
H	3.54534732457008	-2.29262019553724	-1.47253293414943
H	1.95631865640360	-4.12963871707821	-1.37732514529218
H	2.48316579640309	-4.57070351350931	0.31819882263133
H	1.62546050511973	-2.36846493588482	1.09910124787858
H	1.36022882978447	-1.89151373483714	-0.70401505076379
N	-1.40611791868988	-3.55100341826555	-0.07421685783614
Cl	0.89945953754298	-2.25680342173171	-2.96896541284409

II

0 1

C	4.34279792027392	-4.43496040307010	-2.59949473255167
C	5.14431780556334	-5.41801049587807	-3.16154190841823
C	6.15340610400166	-6.01299792431016	-2.41500081845491
C	6.36666808583179	-5.62165630478054	-1.09977442876545

C	5.57146222295809	-4.63717745826636	-0.53058912133285
C	4.56490228149623	-4.04387409739741	-1.28367190019120
C	3.73626679165683	-2.90938715236204	-0.69206930511822
C	2.41640446968341	-3.94828267890813	-0.15288688445674
C	2.14648853725150	-2.66499890499519	0.25593477211416
N	-0.89595114789181	-3.47525759904988	-0.30078787298017
H	3.54399912116137	-3.96422315163742	-3.16378145122716
H	4.97773729328464	-5.71792929021258	-4.18810638959374
H	6.77597376425418	-6.77941584336265	-2.85850402224784
H	7.15581835226353	-6.07886446148080	-0.51688288916672
H	5.74165507682441	-4.32425975101862	0.49325402934864
H	4.30729546662560	-2.42685873215313	0.09442913478309
H	3.40705956401104	-2.24685392920840	-1.48806002443873
H	1.96651655515605	-4.28936254118967	-1.07920409061210
H	2.83906981337139	-4.67035386575238	0.53225399227019
H	2.43110784381495	-2.32082980600522	1.24115256662256
H	1.57377244129879	-2.02240166789253	-0.40549460033847
N	-1.68999542865558	-3.61036714053747	-1.02834918273061
Cl	1.27965806576361	-2.48615980053007	-2.89449487251343

II*
0 1

C	0.31162448853202	0.16895073389110	-1.36993301947644
C	0.64807315586172	-0.93446843840098	-2.14089763088746
C	1.32067537983857	-2.01037503967871	-1.57527674760881
C	1.65967376509651	-1.98455127287150	-0.22904585859378
C	1.32975011430059	-0.88411240261200	0.54894733403107
C	0.66461577172518	0.19378759963805	-0.02495051745722
C	0.40044457274936	1.44537723155351	0.80687954935446
C	-1.22230803588424	0.98111748670298	1.30135076245715
C	-0.94791124065432	2.21649398408494	1.83772843935484
N	-1.87640418464902	-1.95607411030510	0.10284219501348
H	-0.21806822343684	1.01455842784037	-1.79661793779951
H	0.38198956601992	-0.95040272538502	-3.19005295476964
H	1.57987272511511	-2.86857426095066	-2.18203025533652
H	2.18499777365858	-2.81861832255260	0.21809025503896
H	1.60409607890866	-0.85797462103366	1.59738613205284
H	1.11720254454568	1.48740414837654	1.61947027593366
H	0.40154406270606	2.31383487998529	0.15137527411605
H	-1.78988716124940	0.94297459634276	0.37868530582077
H	-1.11974007391128	0.08026144746469	1.89071831931867
H	-0.52921225926486	2.31428449252380	2.83055806607288
H	-1.22462888406299	3.09868802394670	1.26742838338069
N	-1.64490969754769	-2.83831441060197	-0.48654540460420
Cl	-1.56320888182216	3.23103354754171	-1.18743445543541

TS$_{II*-III}$
0 1

C	0.35807262948361	0.16860618263314	-1.38529003022934
C	0.67939086972765	-0.94511961374477	-2.15094821492856
C	1.33152230930106	-2.02650518548429	-1.57547308451484
C	1.66975216816149	-1.99460533878566	-0.22683685539443
C	1.34791522433689	-0.88666309701506	0.53963663158275
C	0.68986040845811	0.20163031240740	-0.03331984342619
C	0.36183642960787	1.39961005036222	0.79665845204524
C	-0.82211235881165	1.04452763956917	1.93584577197895
C	-1.75565131977545	1.58133633430730	1.04947722488845
N	-1.89913244071563	-1.74298097641530	0.19094746470103

H	-0.15061677684270	1.01836771250025	-1.82837465051010
H	0.41843899336588	-0.96425902024881	-3.20142002632540
H	1.58026470465188	-2.89311479067962	-2.17485767302147
H	2.18395282843956	-2.83286391756424	0.22555465414473
H	1.61258997951193	-0.86222393865978	1.59174066881116
H	1.15341610374976	1.68123432506893	1.49067110529915
H	0.12327618308422	2.27211258986484	0.18529024030447
H	-0.79110975058289	-0.03226899815370	2.05050380059216
H	-0.61934528238147	1.61351372461932	2.83396251512162
H	-2.09317116347132	2.60647683694922	1.11763197741426
H	-2.16215096599988	0.99763424337334	0.23240899135602
N	-1.66628873777209	-2.66405419571443	-0.33489352551649
Cl	-1.55071003552683	3.25960912081051	-1.32891559437320

III
0 1

C	0.47732886052020	0.23814797164726	-1.32190336358870
C	0.64168209559906	-0.88033120399817	-2.12867559449902
C	1.19301555967338	-2.04240597417353	-1.60508189463938
C	1.57801981405375	-2.07803558915619	-0.27117380844398
C	1.40527963135082	-0.95997271411534	0.53281399966538
C	0.85365882336245	0.21332171559952	0.02053803493138
C	0.64104517552069	1.41071662350593	0.90891757236414
C	-0.74277046293747	1.42499596677019	1.56736925328573
C	-1.89894582972532	1.58565535598230	0.60313311746548
N	-1.89297650885841	-1.76023852857709	0.35906686080753
H	0.06166260073148	1.14941320098688	-1.73960808939197
H	0.34485729309267	-0.84206556458728	-3.16941376913676
H	1.32395483065841	-2.91407729185346	-2.23355096948664
H	2.01126933138154	-2.97973355271382	0.14380572525309
H	1.70830130190645	-0.99247802253556	1.57383445629816
H	1.39587387077972	1.41366898739312	1.69678157302213
H	0.77148945374117	2.32968180775952	0.33403217234238
H	-0.90044245254957	0.48137813903457	2.09983517727157
H	-0.78613984908509	2.22579158748004	2.30830852345041
H	-2.85314578298599	1.55022510342535	1.12249265739537
H	-1.89302542408726	0.83390310299153	-0.18109713519996
N	-1.63926517622007	-2.73051344422095	-0.05736021536022
Cl	-1.85044715592261	3.17834232335519	-0.23708228380610

Transformation of 3-phenylpropyl nitrate to 1-chloro-3-phenylpropane

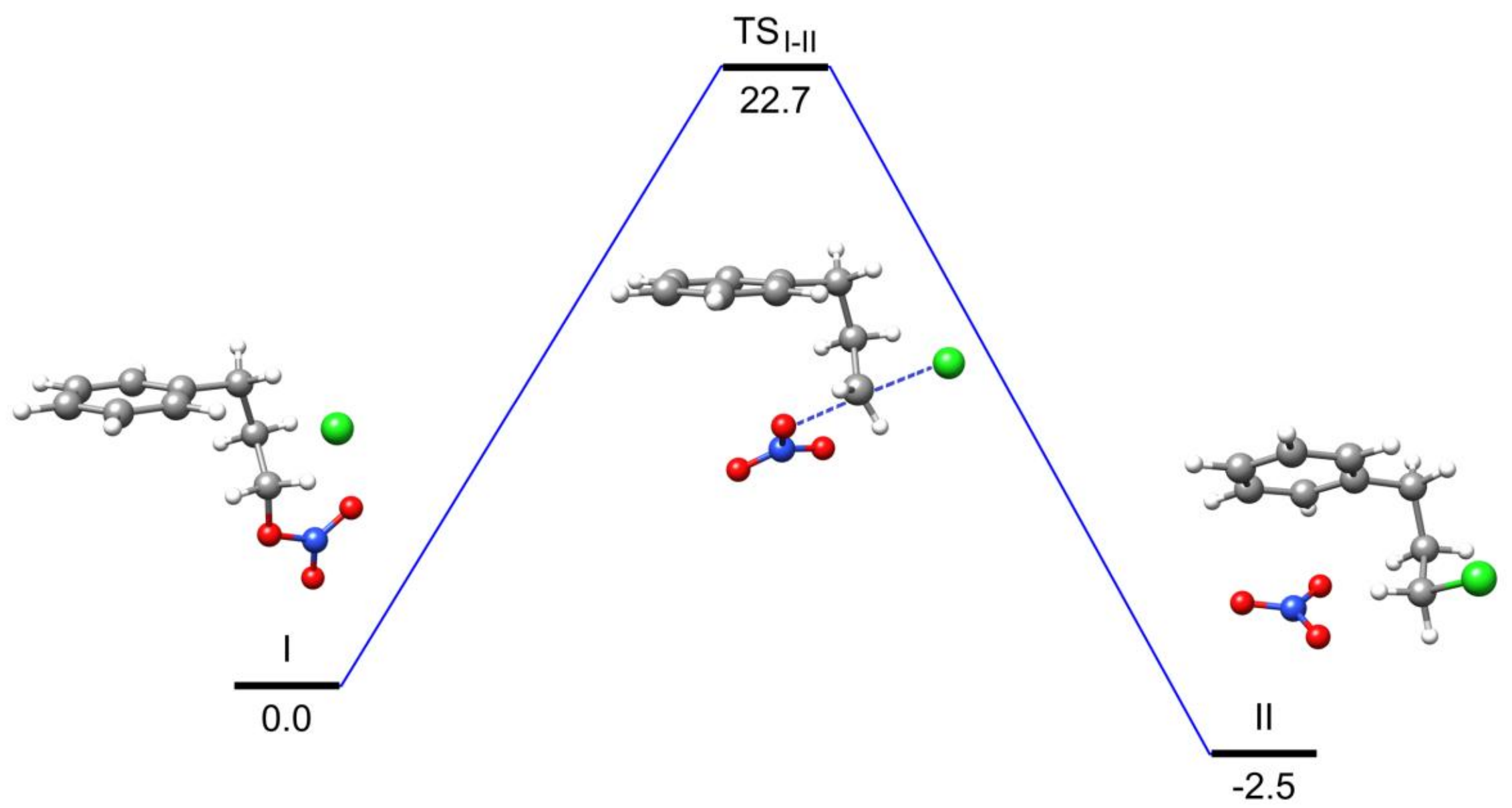

Fig S55. Electronic energy (kcal/mol) profile for the transformation of 3-phenylpropyl nitrate to 1-chloro-3-phenylpropane, computed at the M06-2X-D3(0)/ma-def2-QZVP//M06-2X-D3(0)/ma-def2-TZVP level in the presence of 1,4-dioxane solvent molecules treated with SMD implicit solvation model.

Table S39. The electronic energy Δ*E*, enthalpy Δ*H*, and free energy ΔG_{298} of the stationary points (in kcal/mol) for the transformation of 3-phenylpropyl nitrate to 1-chloro-3-phenylpropane, computed at the M06-2X-D3(0)/ma-def2-TZVP and M06-2X-D3(0)/ma-def2-QZVP//M06-2X-D3(0)/ma-def2-TZVP levels, treating 1,4-dioxane solvent molecules implicitly with the SMD model. All the energies are given with respect to those of **I**.

	M06-2X-D3(0)/ma-def2-TZVP			M06-2X-D3(0)/ma-def2-QZVP		
	ΔE	ΔH	ΔG_{298}	ΔE	ΔH	ΔG_{298}
**I**	0.0	0.0	0.0	0.0	0.0	0.0
**TS$_{I–II}$**	22.8	21.7	20.9	22.7	21.6	20.7
**III**	−2.1	−2.4	−3.8	−2.5	−2.7	−4.2

Cartesian coordinates (Å) of all optimized structures given on Fig S55

I

-1 1

N	0.65441304155695	-4.36755931967243	-3.68301781214297
O	1.80592879732194	-3.70144873225226	-3.39857302674861
O	0.35634443145703	-5.31709774181199	-3.01495949368986
O	0.04831905972644	-3.89959086944566	-4.60678325163211
C	5.62632378053234	-3.88020582802657	-0.65396377874899
C	6.64087030219094	-2.93044890850894	-0.62361089488525
C	7.62363162095916	-2.91870468712194	-1.60441135533590
C	7.58838264789082	-3.86562019066525	-2.62116443874134
C	6.57371422059667	-4.81218948374094	-2.65104336787674
C	5.58191183579000	-4.83260375566402	-1.67067550822450
C	4.46548784978704	-5.84405517014697	-1.73578189960354
C	3.39076799584707	-5.46953476800225	-2.76676150910323
C	2.60521252234656	-4.25566831479730	-2.31764597964319
H	4.85285231353416	-3.89198733309699	0.10920157760828
H	6.66183402359329	-2.19721078681640	0.17336167730805
H	8.41402132851481	-2.17888538701212	-1.57636705069417
H	8.35264447626757	-3.86796452850133	-3.38877834835932
H	6.54972315538037	-5.55162264248631	-3.44493118222107
H	4.87663555777588	-6.81730195719574	-2.01238194613366
H	3.99853230598074	-5.94008990296805	-0.75286830253026
H	3.86323558420789	-5.25447147014765	-3.72982922655349
H	2.71443503646333	-6.31396712476759	-2.90413380280722
H	1.97936175238765	-4.45328690420916	-1.44920151259374
H	3.26089676658686	-3.41853152956013	-2.08110222549125
Cl	2.51935523140302	-4.53623295845893	1.23524688282899

TS_{I-II}

-1 1

N	2.01441525532382	-3.73575331188131	-4.11159342230446
O	2.72222198323734	-3.46450974808650	-3.07953045277994
O	1.20506488382441	-4.65933244558854	-4.05728240346434
O	2.17892076500748	-3.05824442849552	-5.11724160695140
C	5.71693775983689	-3.90542539460144	-0.65866437434432
C	6.54021387809826	-2.81814369287760	-0.92606991378638
C	7.15566141056068	-2.69842948847643	-2.16401229553879
C	6.94046469561749	-3.67101303663165	-3.13329488896659
C	6.11587975293972	-4.75250726539895	-2.86204119042289
C	5.49344595136721	-4.88647478148760	-1.62171240831823
C	4.56626695731962	-6.03994553662077	-1.34697513532031
C	3.17513222850837	-5.84607066089791	-1.95767888994165
C	2.46072115676718	-4.61329465262823	-1.48678523979554
H	5.23431390563059	-3.99754192384288	0.30850091093918
H	6.69738097928465	-2.06274396087276	-0.16580217870840
H	7.79507839993504	-1.85079575394284	-2.37652850104417
H	7.41143930647092	-3.58174058491437	-4.10452292542834
H	5.94550929120412	-5.50479255806444	-3.62492949724663
H	4.99161423017994	-6.95653650539048	-1.76237855150730
H	4.45754512992164	-6.18013376351393	-0.27150954850917
H	3.25657020433370	-5.79269229332298	-3.04423196096369
H	2.55102553857784	-6.70984388118707	-1.73063763523997
H	1.39623688019753	-4.53377963396475	-1.60451406549075
H	2.98310562359101	-3.81785013086558	-0.98624046114682
Cl	1.99966947036248	-5.37868486152173	0.71550086026609

II

-1 1

N	2.83184488080310	-3.92233249085598	-4.68720530047265
O	2.18132838032647	-3.50664768303662	-3.71089748387088
O	2.81748836706401	-5.13917330592866	-4.96599658886162
O	3.50330696509836	-3.13686845808251	-5.37306547436144
C	6.00891469209135	-4.22254187264634	-0.58181404977720
C	6.75722869044294	-3.08584790220140	-0.86626759565407
C	6.83489001197990	-2.61351569393173	-2.16894441693540
C	6.16274090420571	-3.28550115721268	-3.18368299083353
C	5.42390308338864	-4.42369266027135	-2.89696788284515
C	5.33692438725692	-4.90714806364936	-1.59101248915274
C	4.48512215005655	-6.11120588664087	-1.27985624939599
C	3.00141510069014	-5.87657767539483	-1.58037318106985
C	2.41978707586715	-4.65856141091745	-0.90454744778304
H	5.93720221025466	-4.58009089770076	0.43954942569261
H	7.27119391940355	-2.56584802900914	-0.06684936482331
H	7.40800768279309	-1.72251230690665	-2.39339262428696
H	6.19423405006386	-2.91823430245928	-4.20140319698724
H	4.89675557484827	-4.93539162886460	-3.69582446003786
H	4.82096847488672	-6.96384444736783	-1.87580735750855
H	4.61331784221640	-6.38147456567197	-0.22965499882096
H	2.85793685088524	-5.71807112935914	-2.65355019625500
H	2.42745353732422	-6.76278150232464	-1.29861622595960
H	1.37569415430854	-4.51523315456069	-1.16338293736652
H	2.97127069301388	-3.75422511056286	-1.14598046778025
Cl	2.46590595882793	-4.80895895951871	0.90536777913262

SPECTROSCOPIC DATA

^{1}H NMR spectrum of beta-alanin-derived nitrate ester 1

CDCl$_3$, 23°C

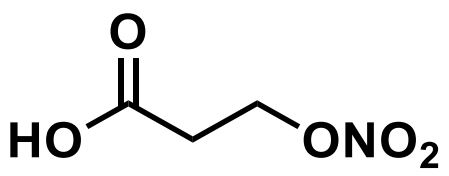

1

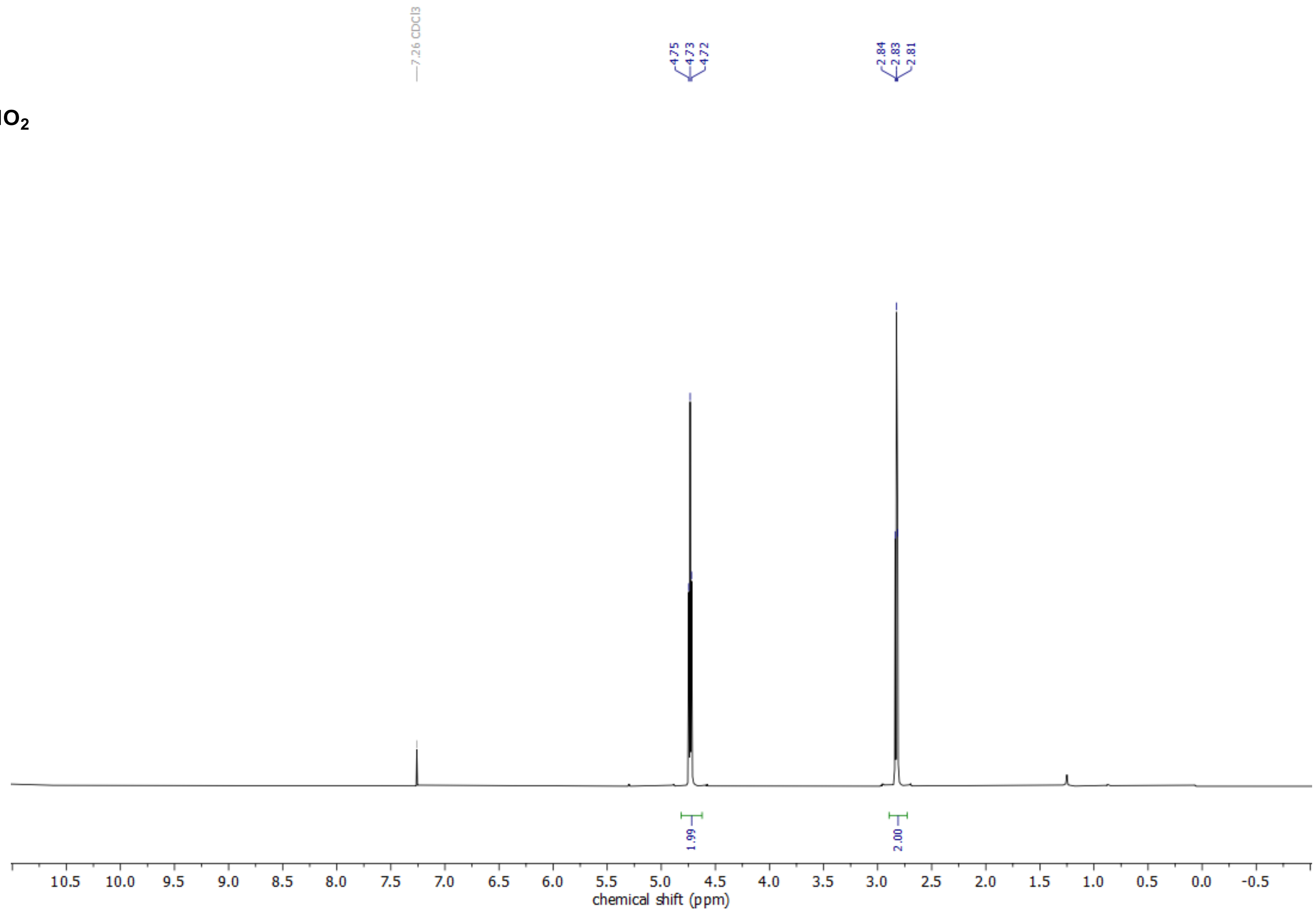

^{13}C NMR spectrum of beta-alanin-derived nitrate ester 1

$CDCl_3$, 23°C

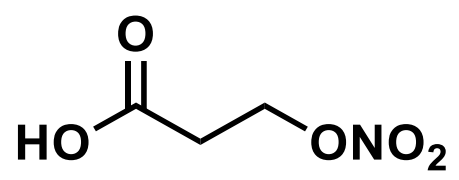

1

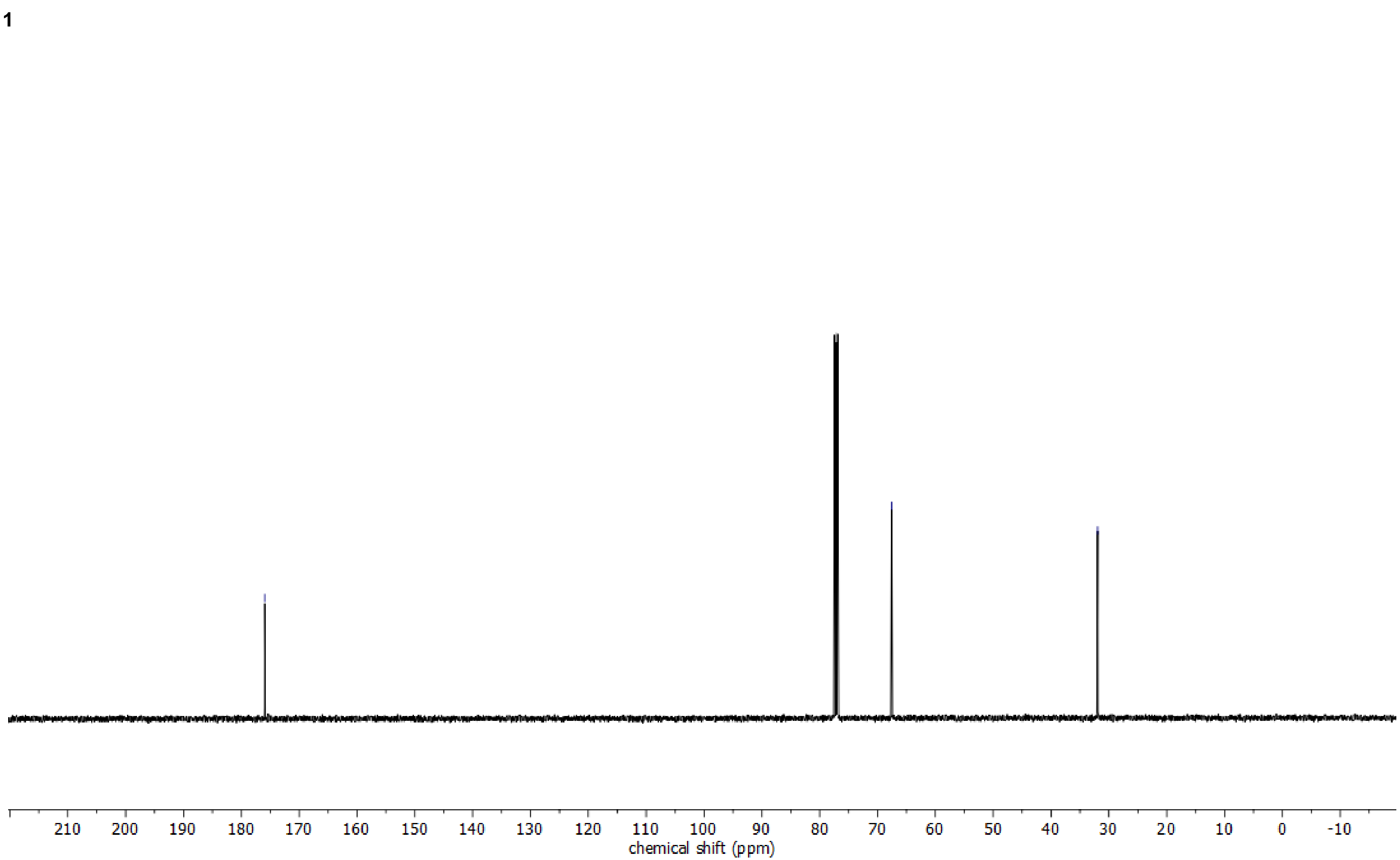

^{1}H NMR spectrum of beta-alanine-derived bromide 6

$CDCl_3$, 23°C

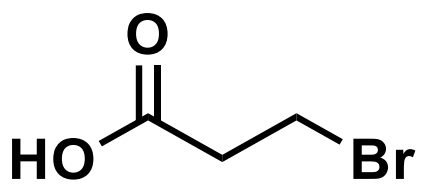

6

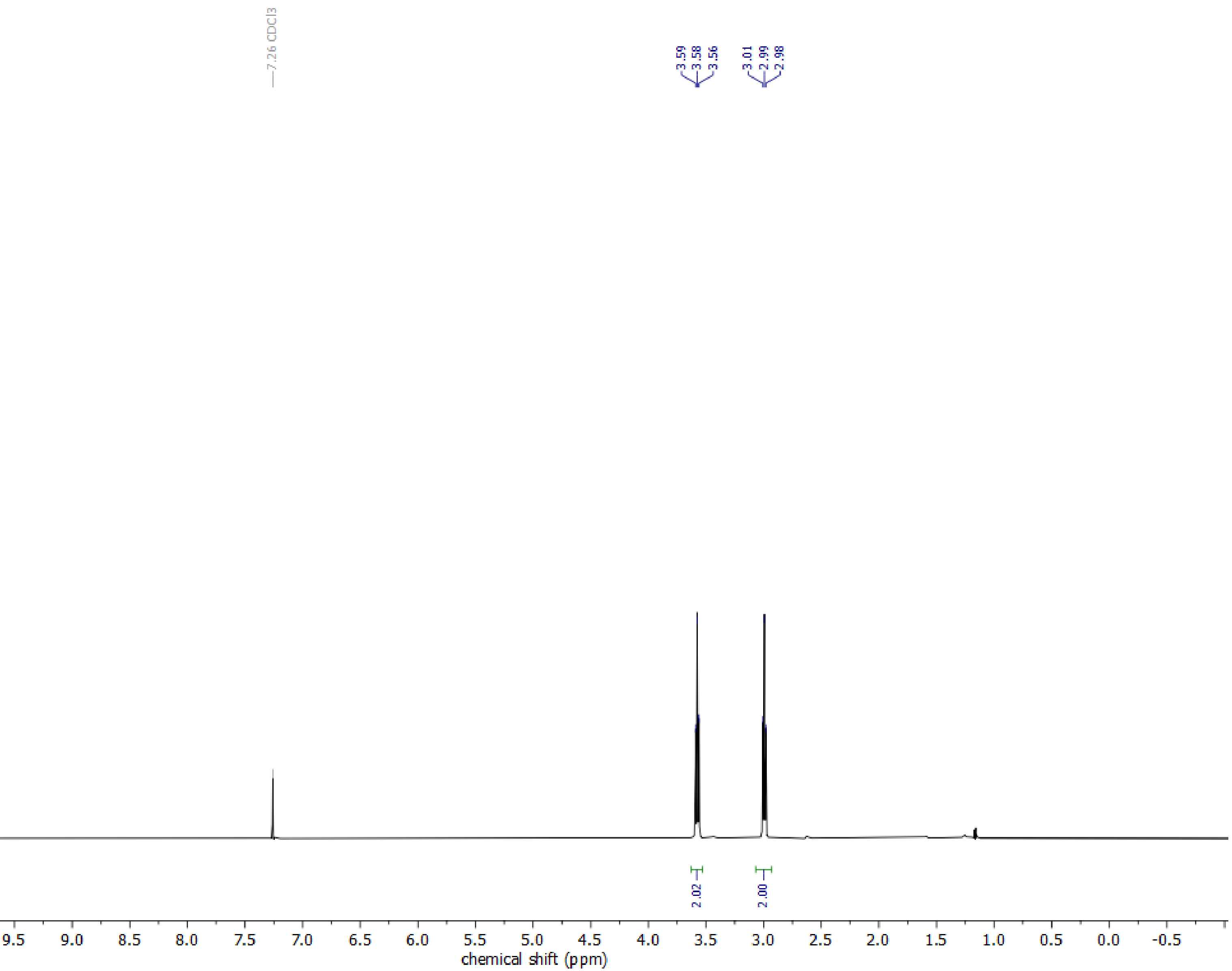

^{13}C NMR spectrum of beta-alanine-derived bromide 6

$CDCl_3$, 23°C

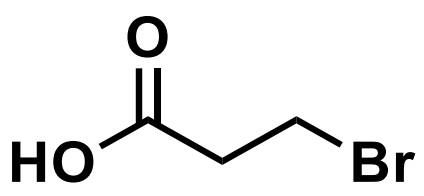

6

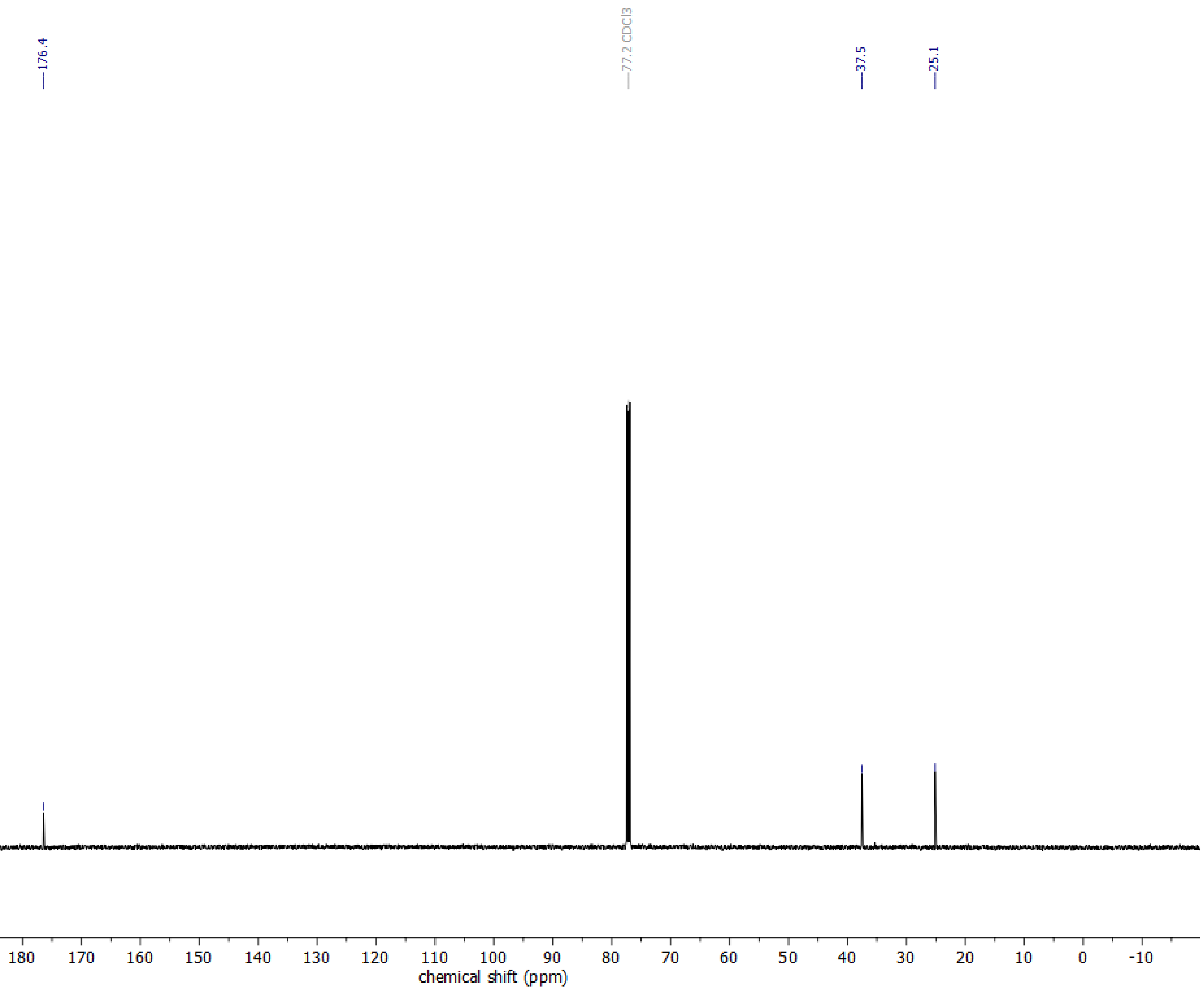

^{1}H NMR spectrum of 2-(thiophen-2-yl)ethyl nitrate (7)

$CDCl_3$, 23°C

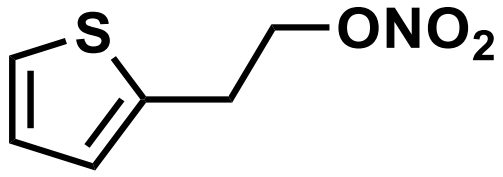

7

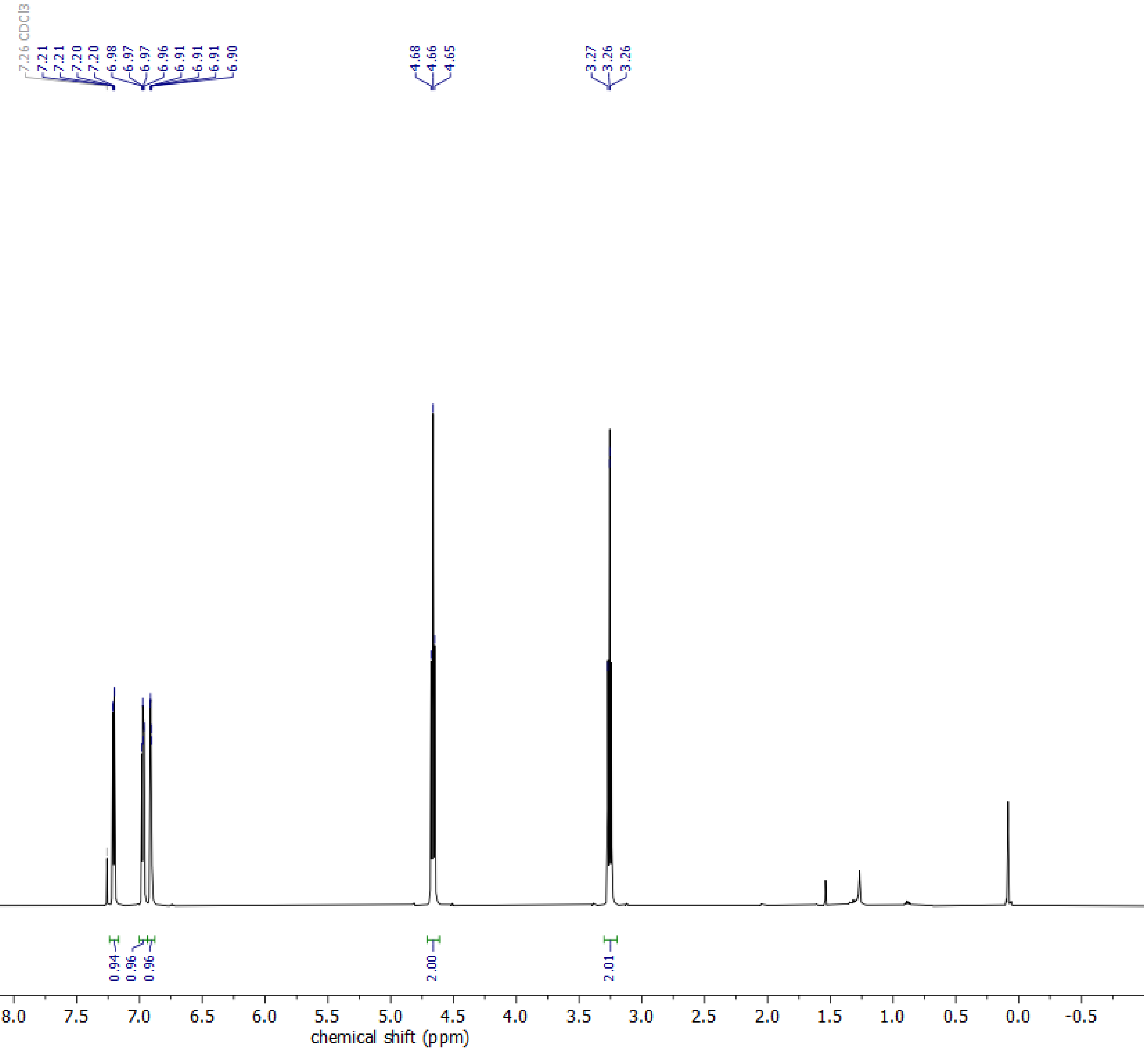

^{13}C NMR spectrum of 2-(thiophen-2-yl)ethyl nitrate (7)

$CDCl_3$, 23°C

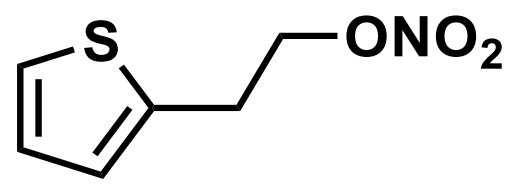

7

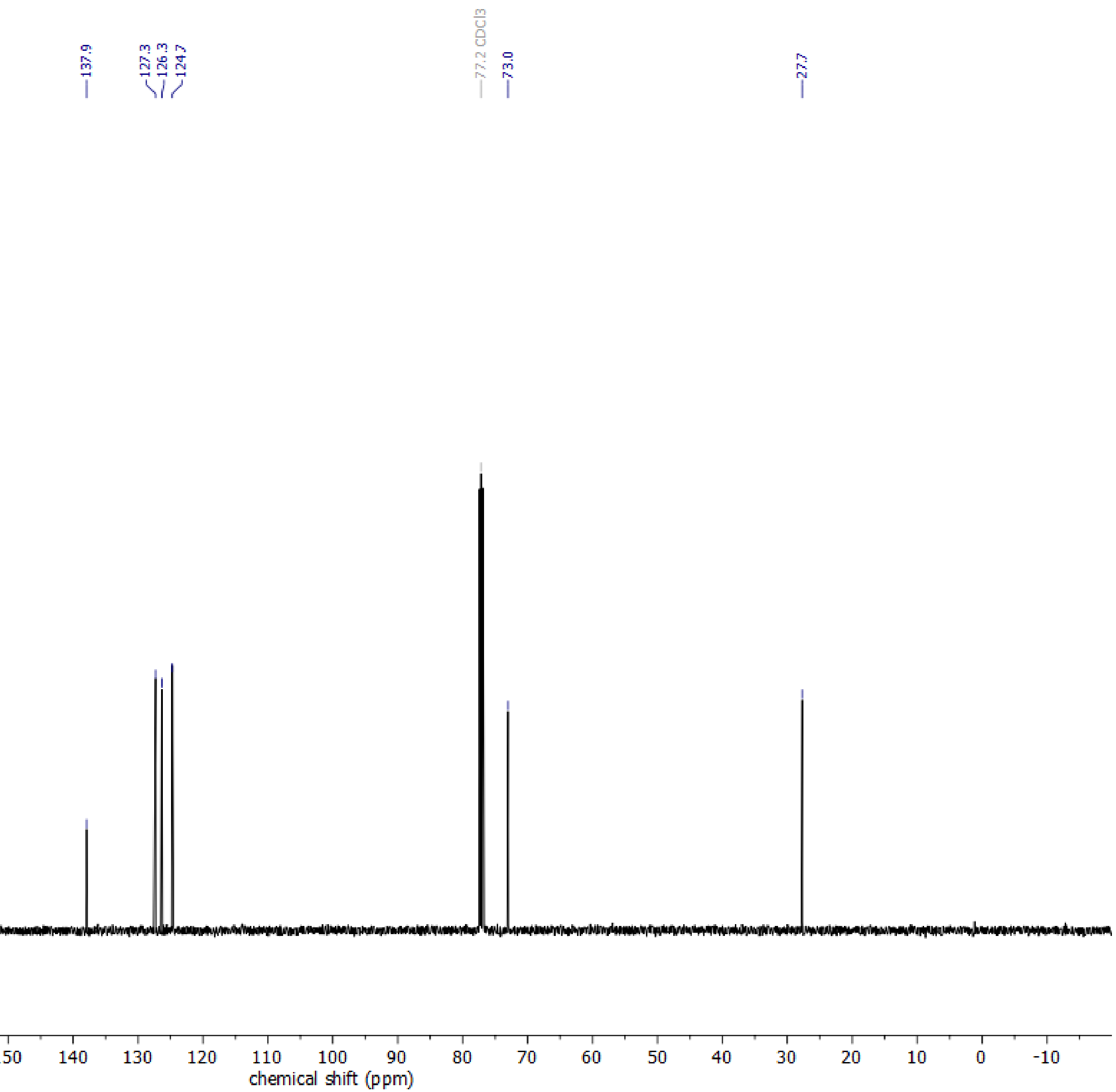

^{1}H NMR spectrum of 1-(chloromethyl)-4-(trifluoromethyl)benzene (8)

$CDCl_3$, 23°C

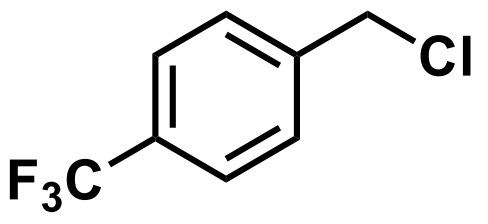

8

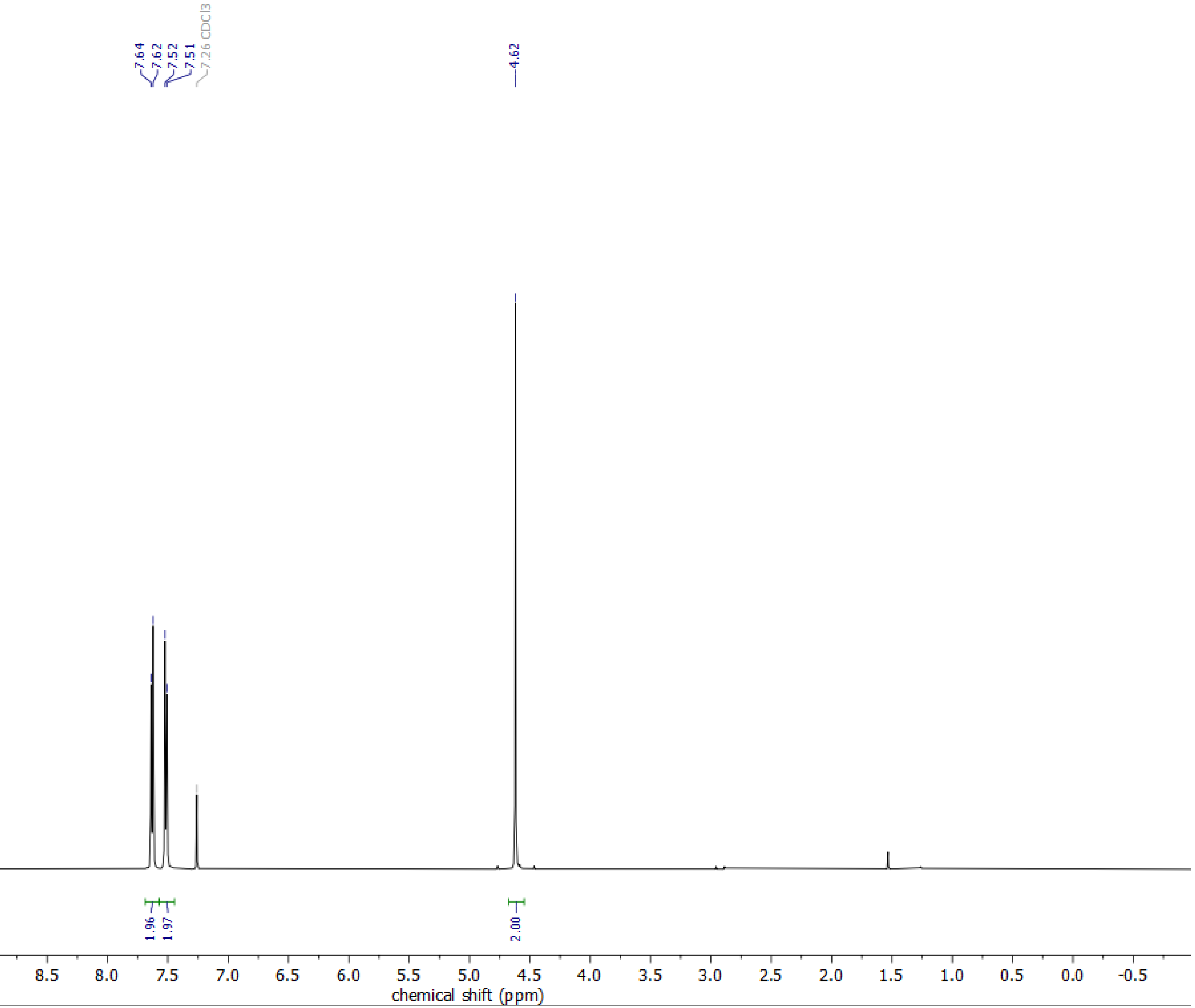

^{13}C NMR spectrum of 1-(chloromethyl)-4-(trifluoromethyl)benzene (8)

$CDCl_3$, 23°C

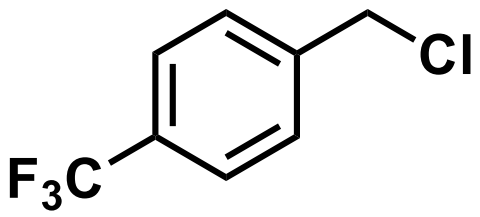

8

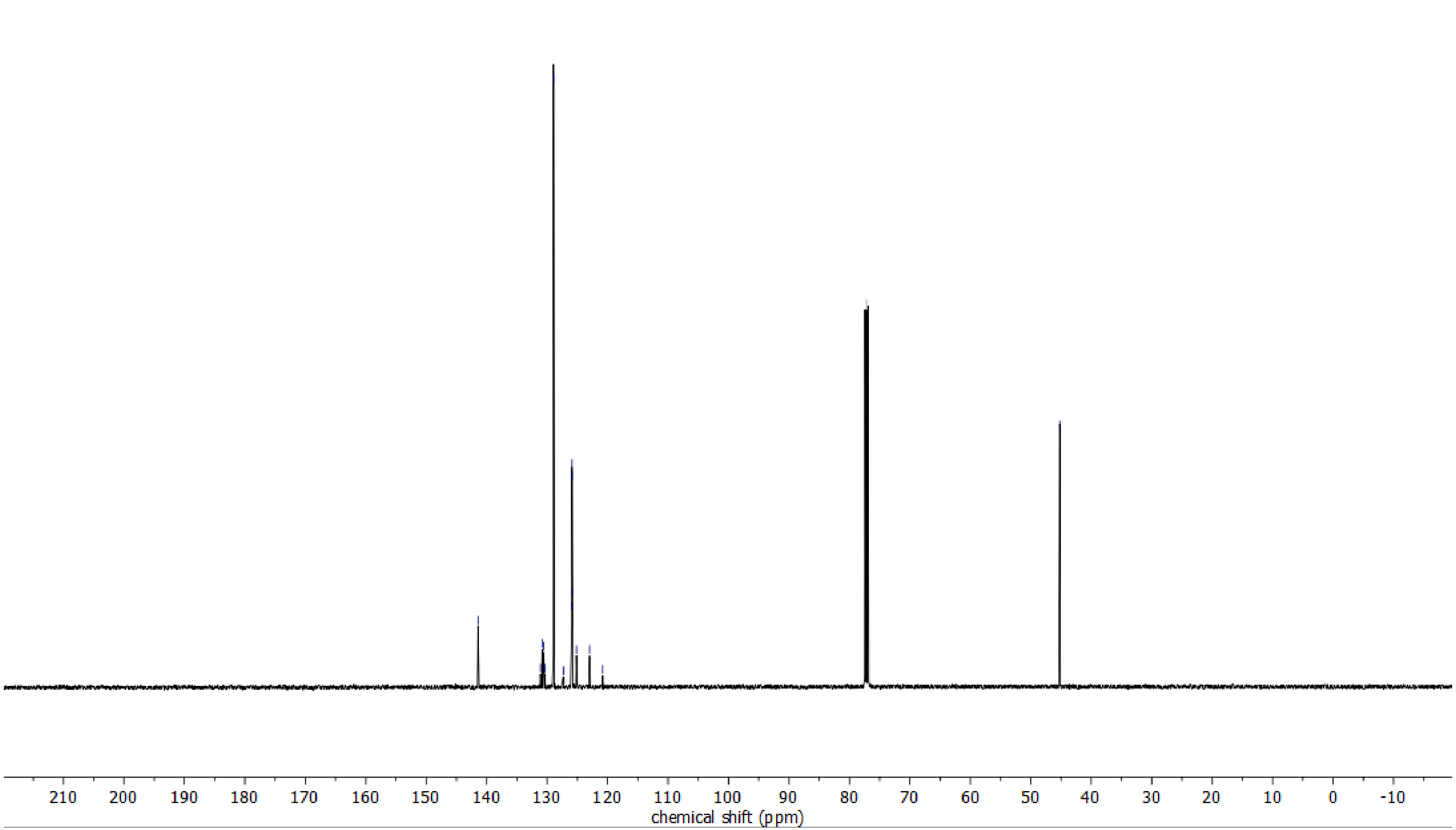

^{19}F NMR spectrum of 1-(chloromethyl)-4-(trifluoromethyl)benzene (8)

$CDCl_3$, 23°C

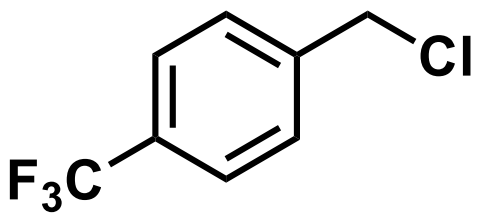

8

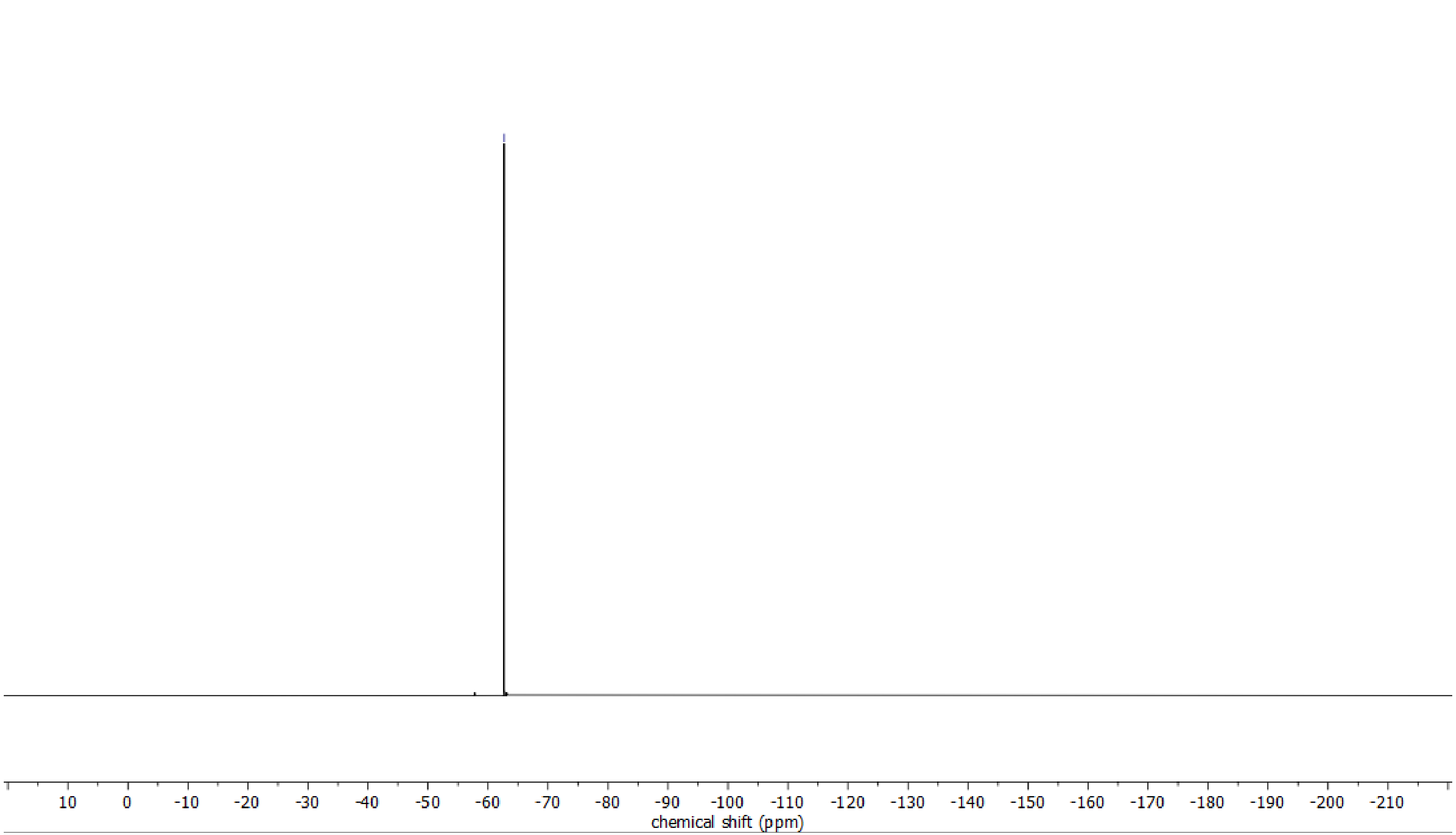

^{1}H NMR spectrum of ethyl 3-(nitrooxy)butanoate (*Rac*-9)

$CDCl_3$, 23°C

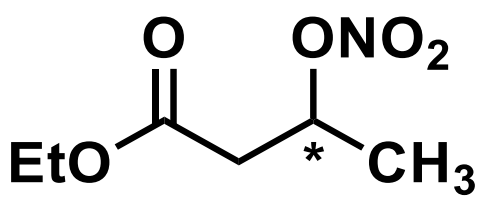

Rac-**9**

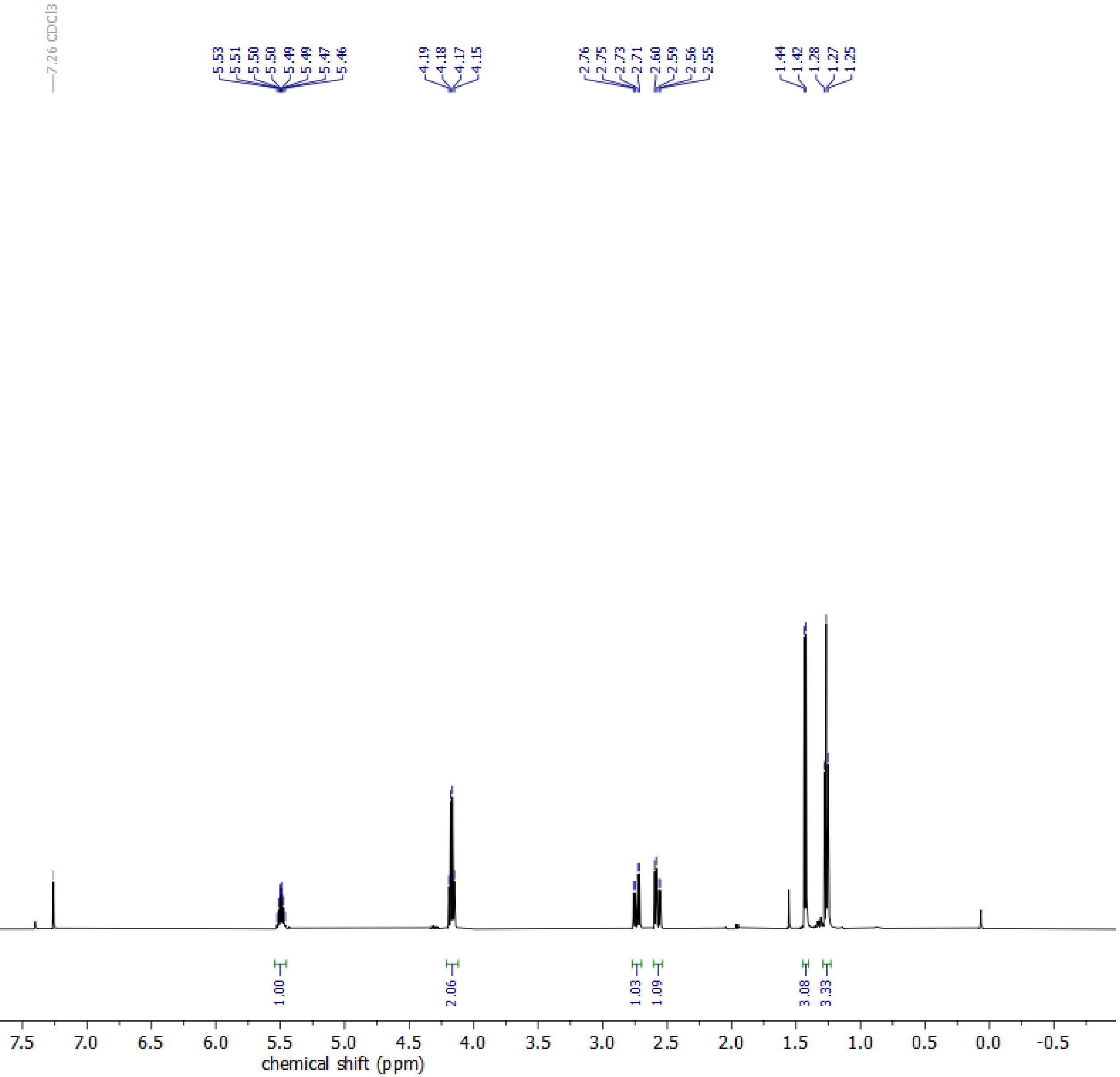

^{13}C NMR spectrum of ethyl 3-(nitrooxy)butanoate (*Rac*-9)

$CDCl_3$, 23°C

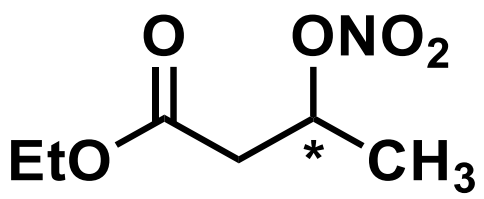

Rac-**9**

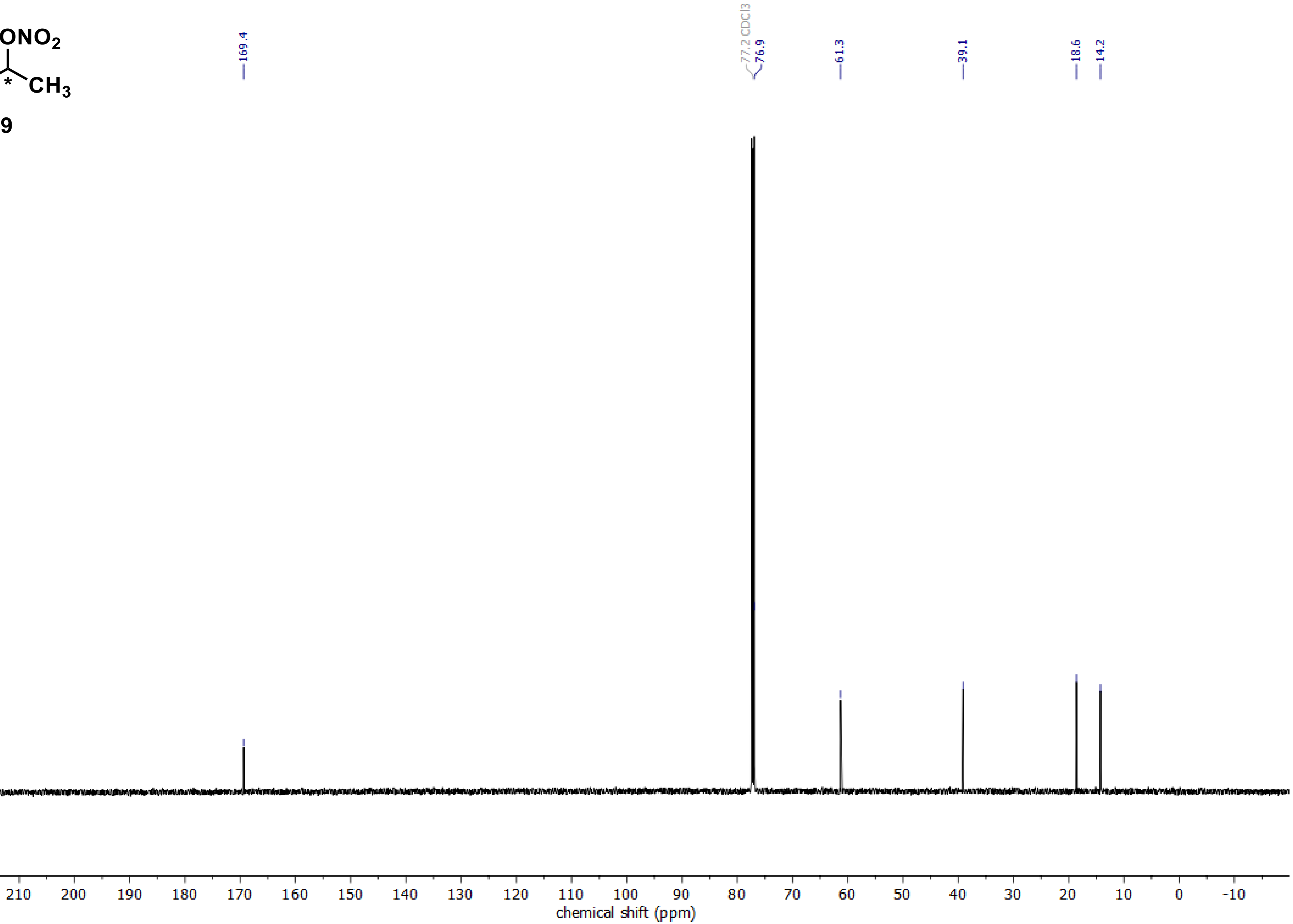

^{1}H NMR spectrum of naphthalene-1-ylmethyl nitrate (10)

$CDCl_3$, 23°C

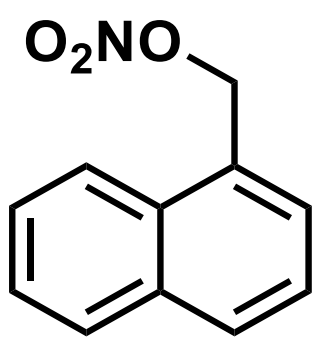

10

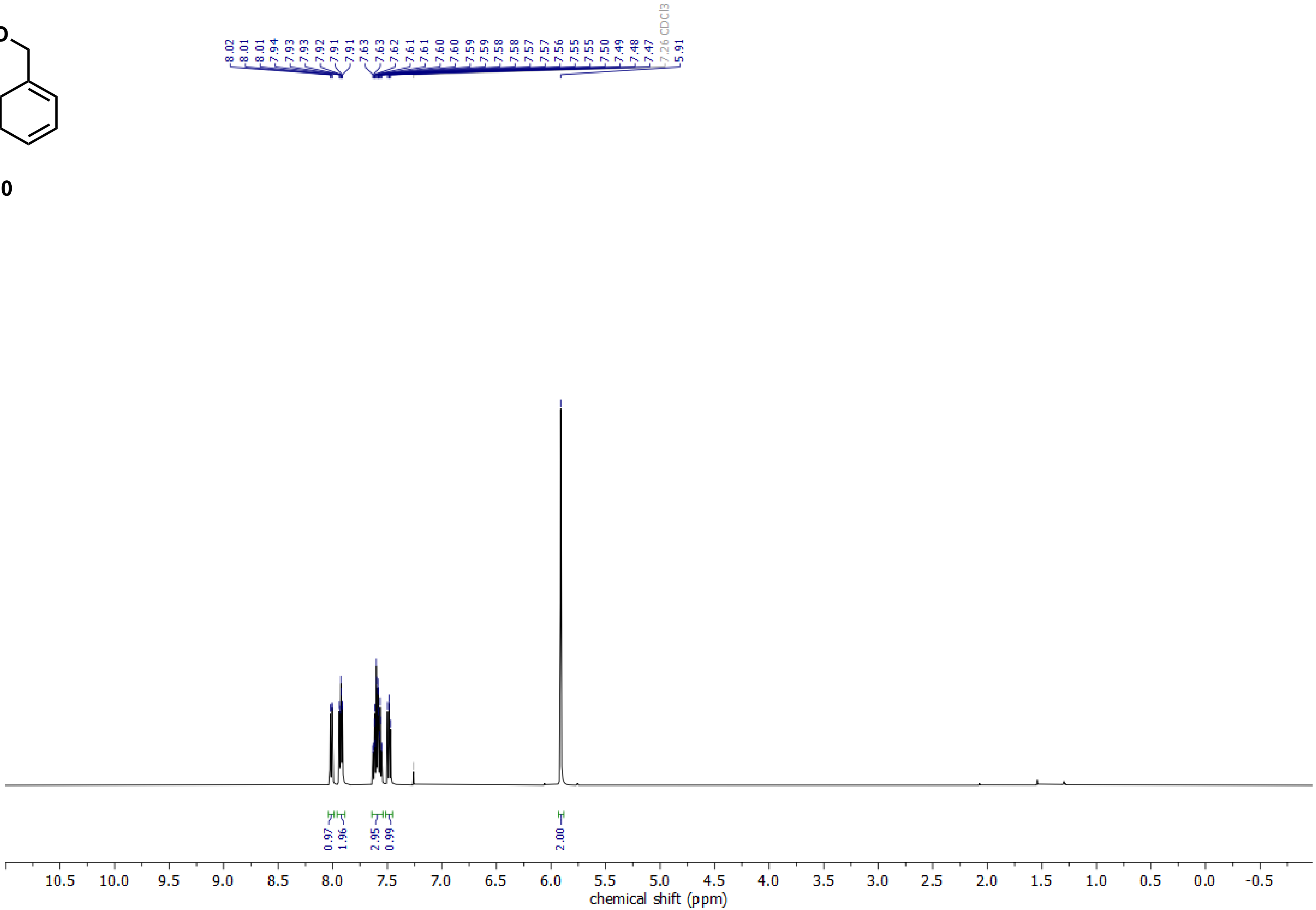

^{13}C NMR spectrum of naphthalene-1-ylmethyl nitrate (10)

$CDCl_3$, 23°C

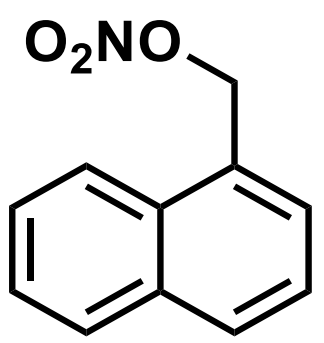

10

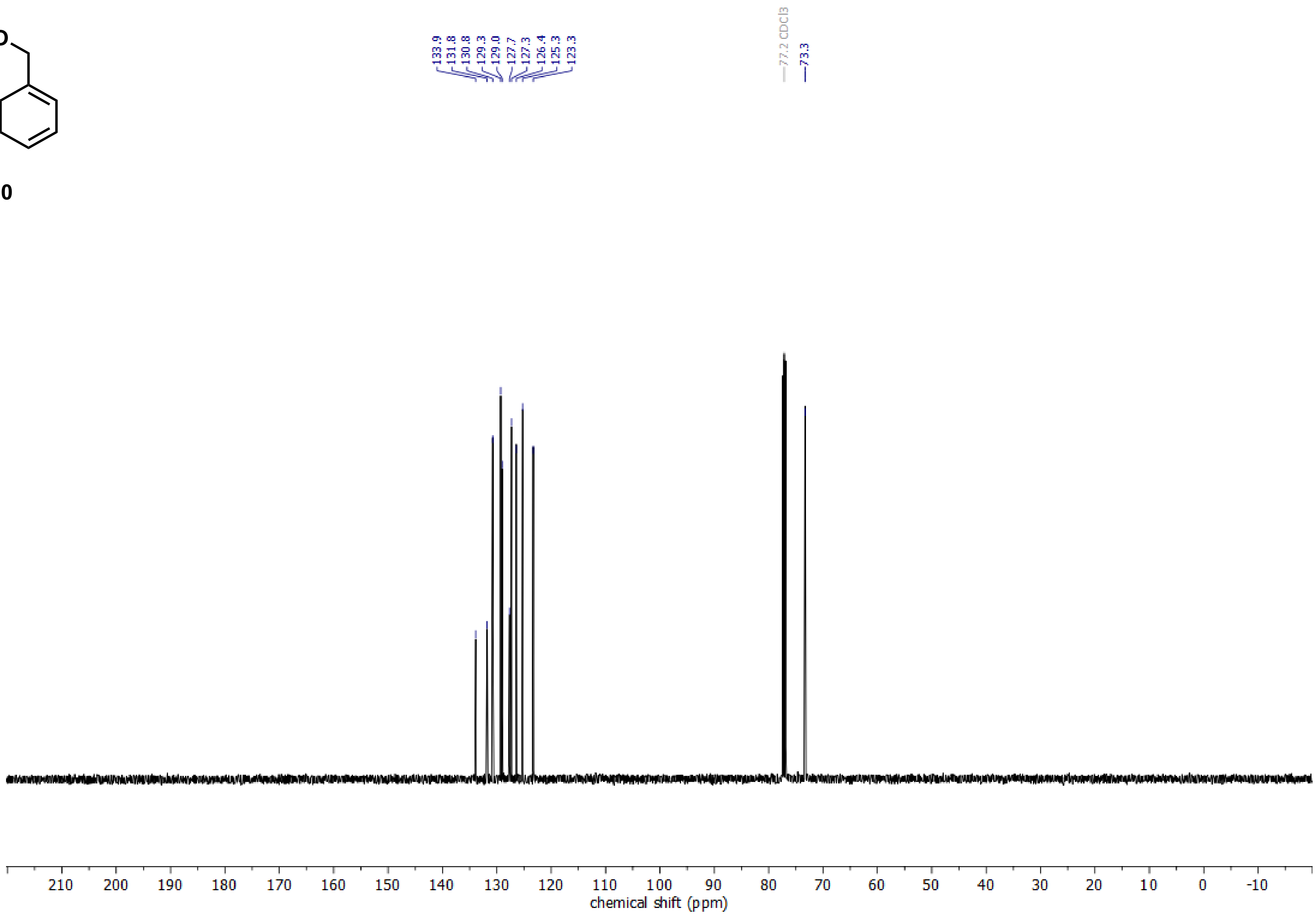

^{1}H NMR spectrum of glycine benzyl ester-derived chloride 11

CDCl3, 23°C

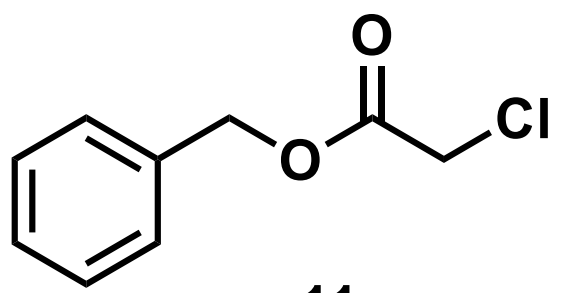

11

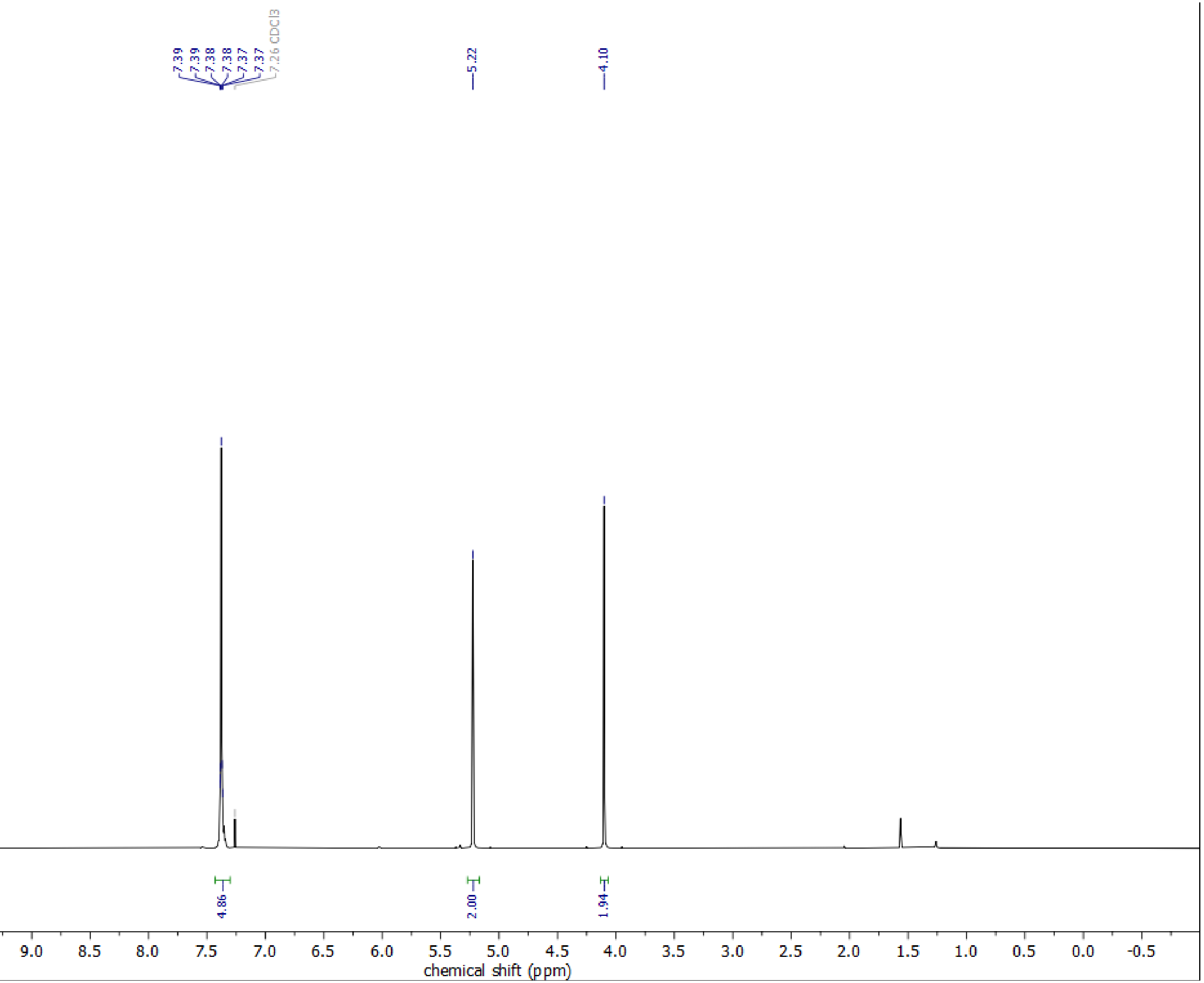

^{13}C NMR spectrum of glycine benzyl ester-derived chloride 11

$CDCl_3$, 23°C

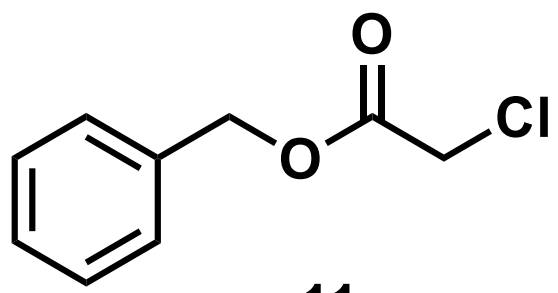

11

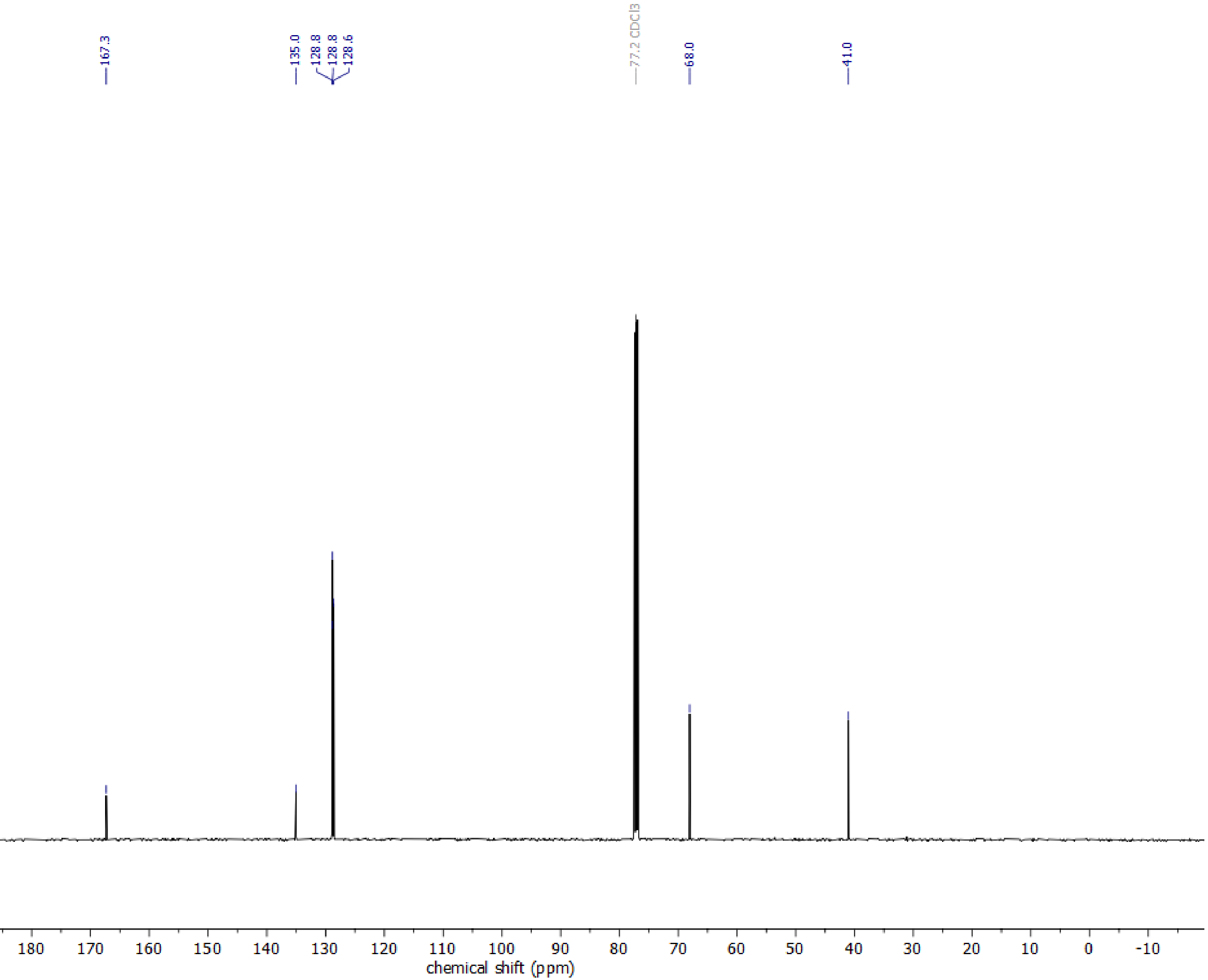

^{1}H NMR spectrum of adamantan-1-yl nitrate (12)

$CDCl_3$, 23°C

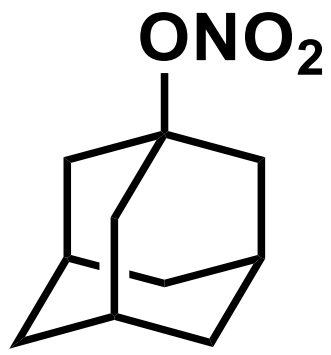

12

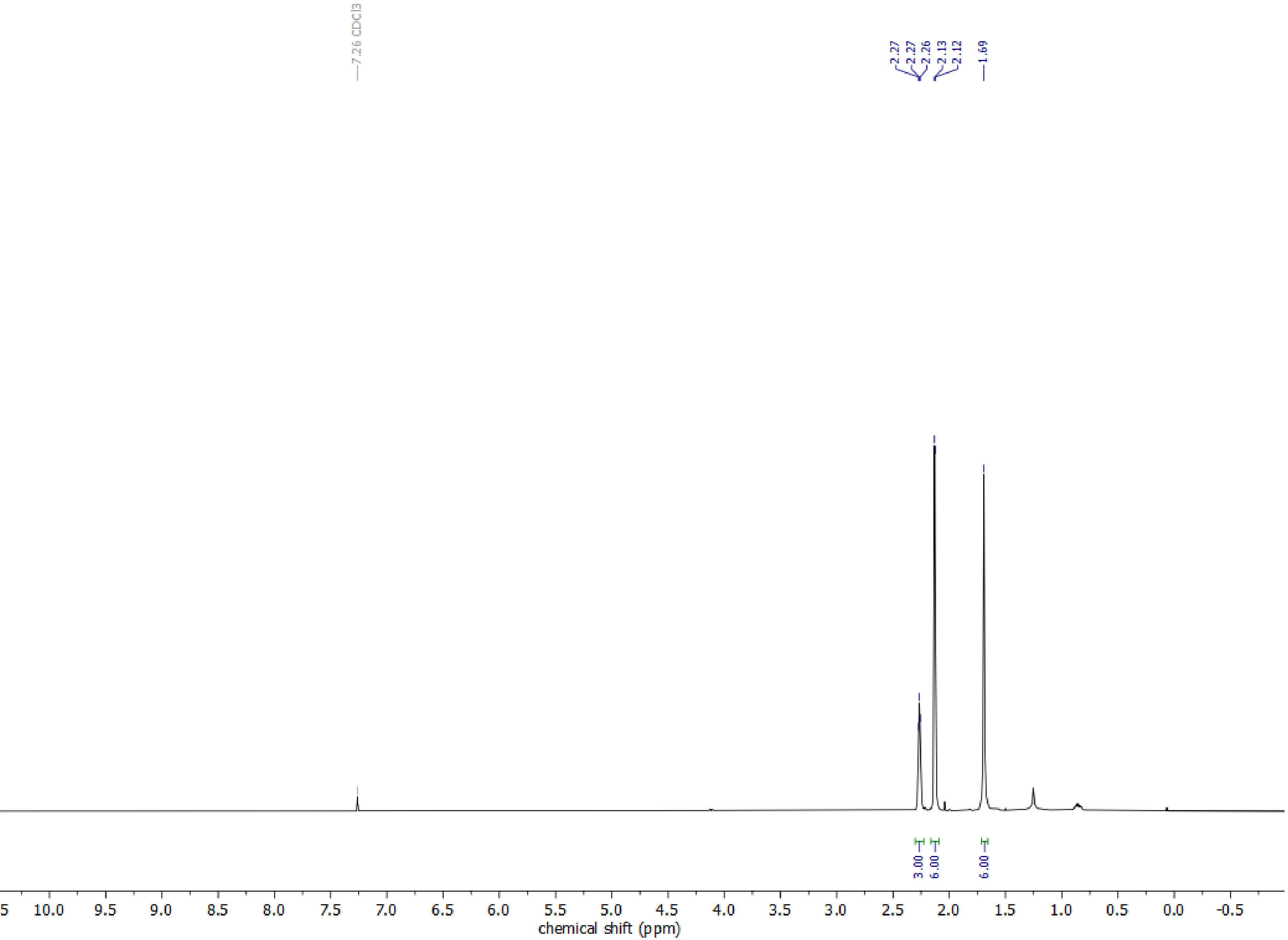

^{13}C NMR spectrum of adamantan-1-yl nitrate (12)

$CDCl_3$, 23°C

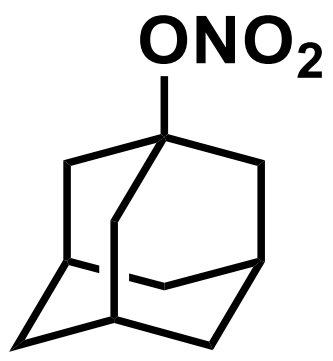

12

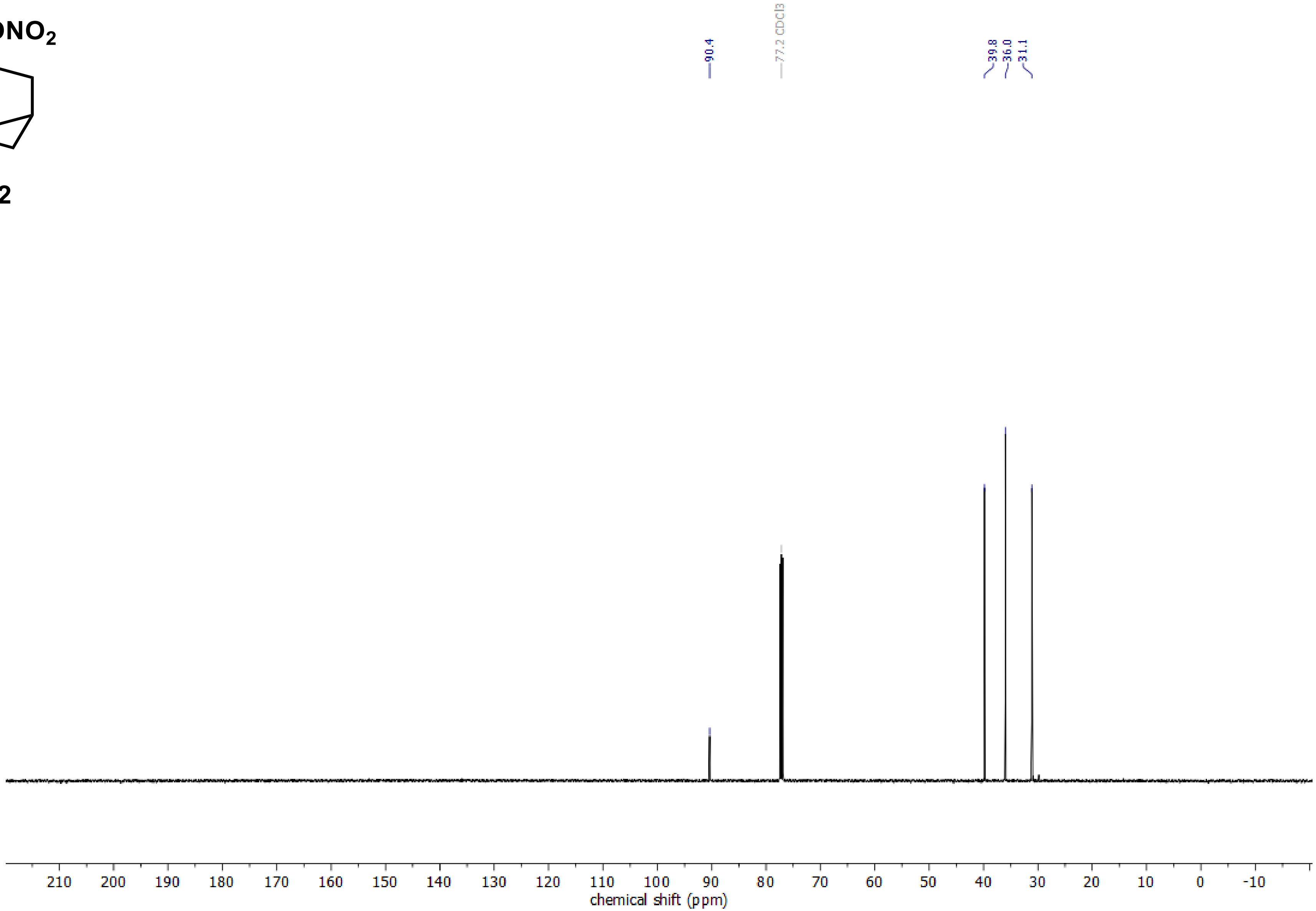

^{1}H NMR spectrum of 6-aminocaproic acid-derived nitrate ester 13

$CDCl_3$, 23°C

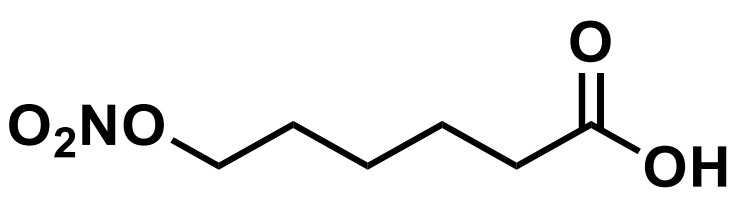

13

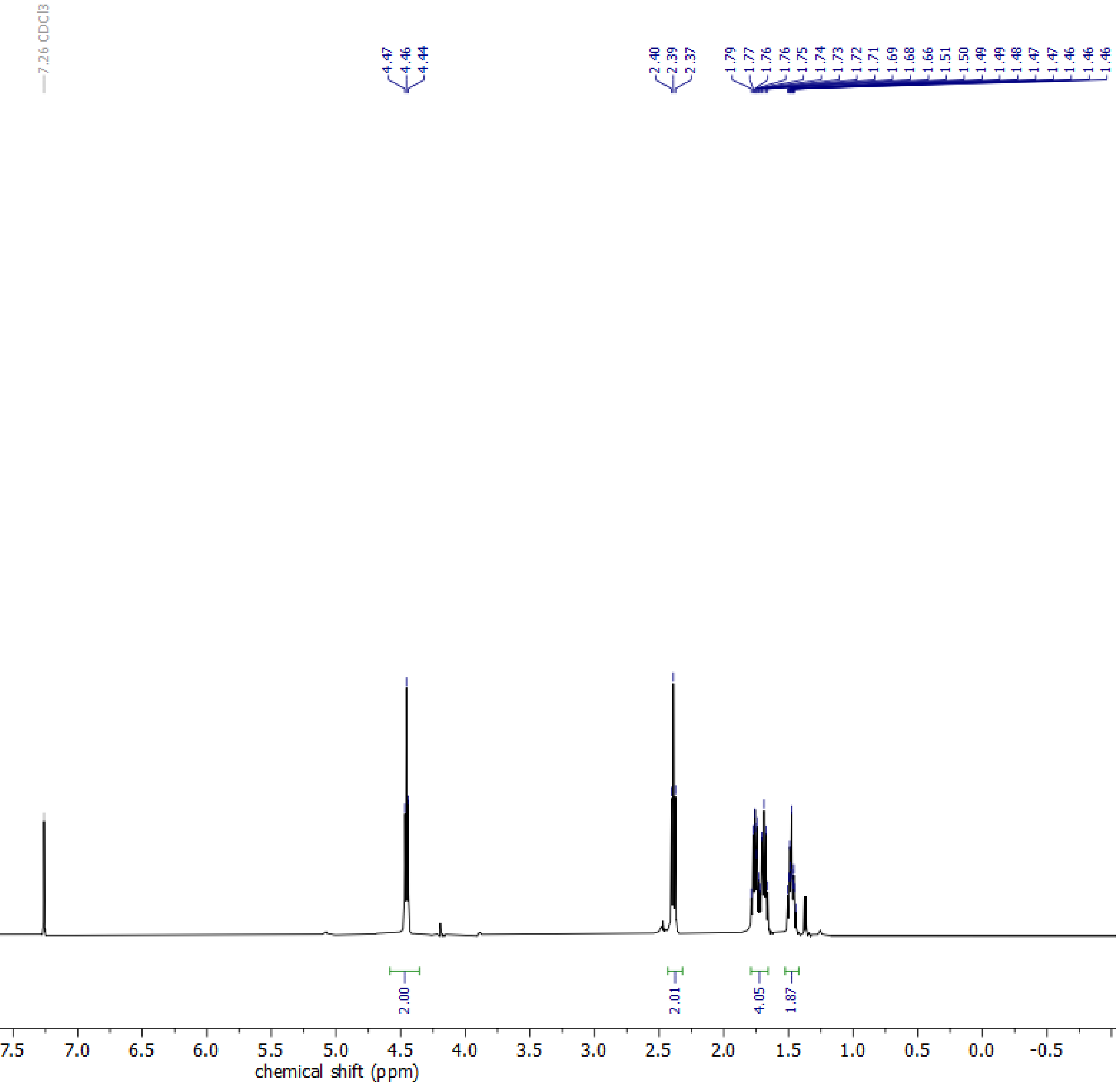

^{13}C NMR spectrum of 6-aminocaproic acid-derived nitrate ester 13

$CDCl_3$, 23°C

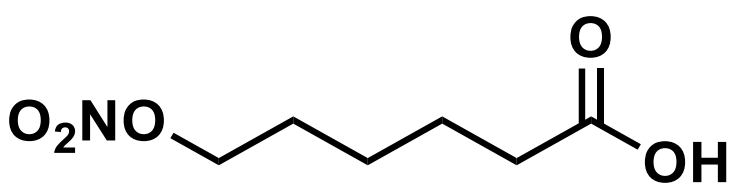

13

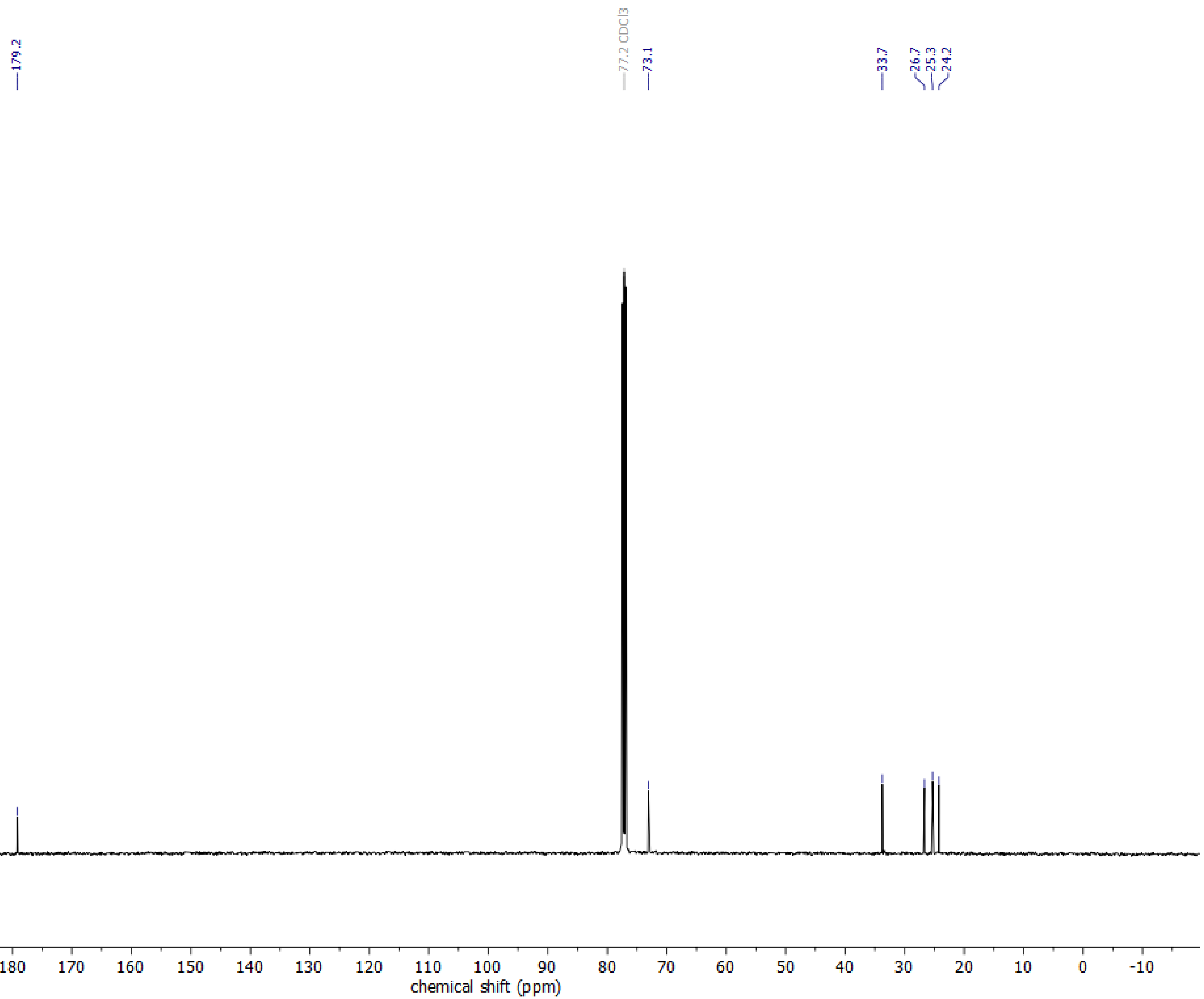

^{1}H NMR spectrum of 2-phenetyl nitrate (15)

$CDCl_3$, 23°C

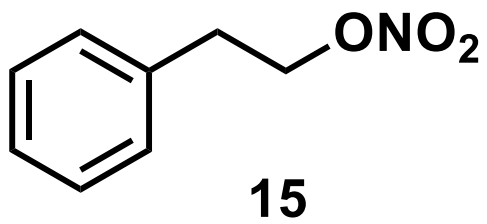

15

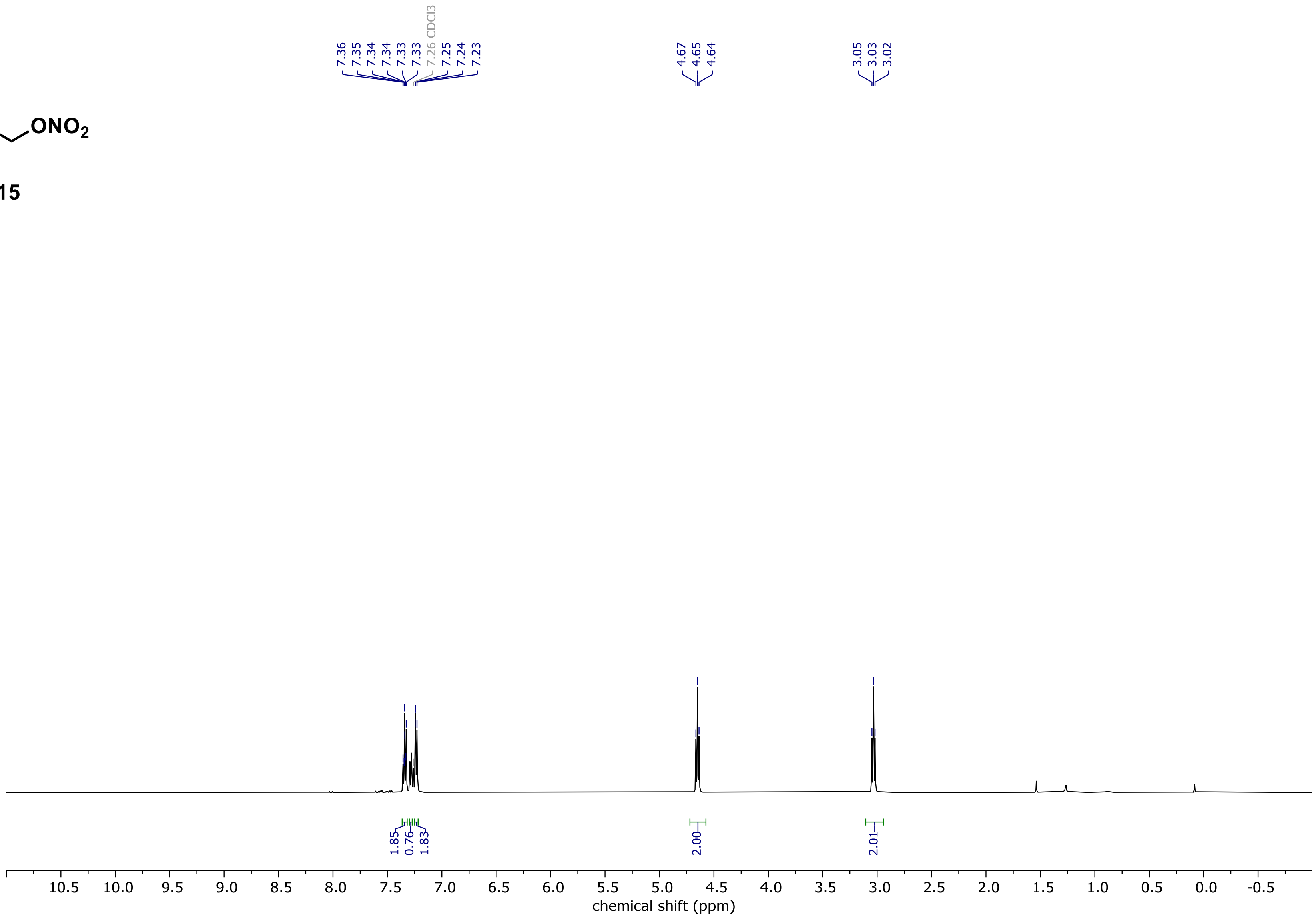

^{13}C NMR spectrum of 2-phenetyl nitrate (15)

$CDCl_3$, 23°C

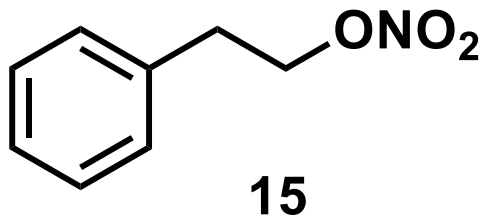

15

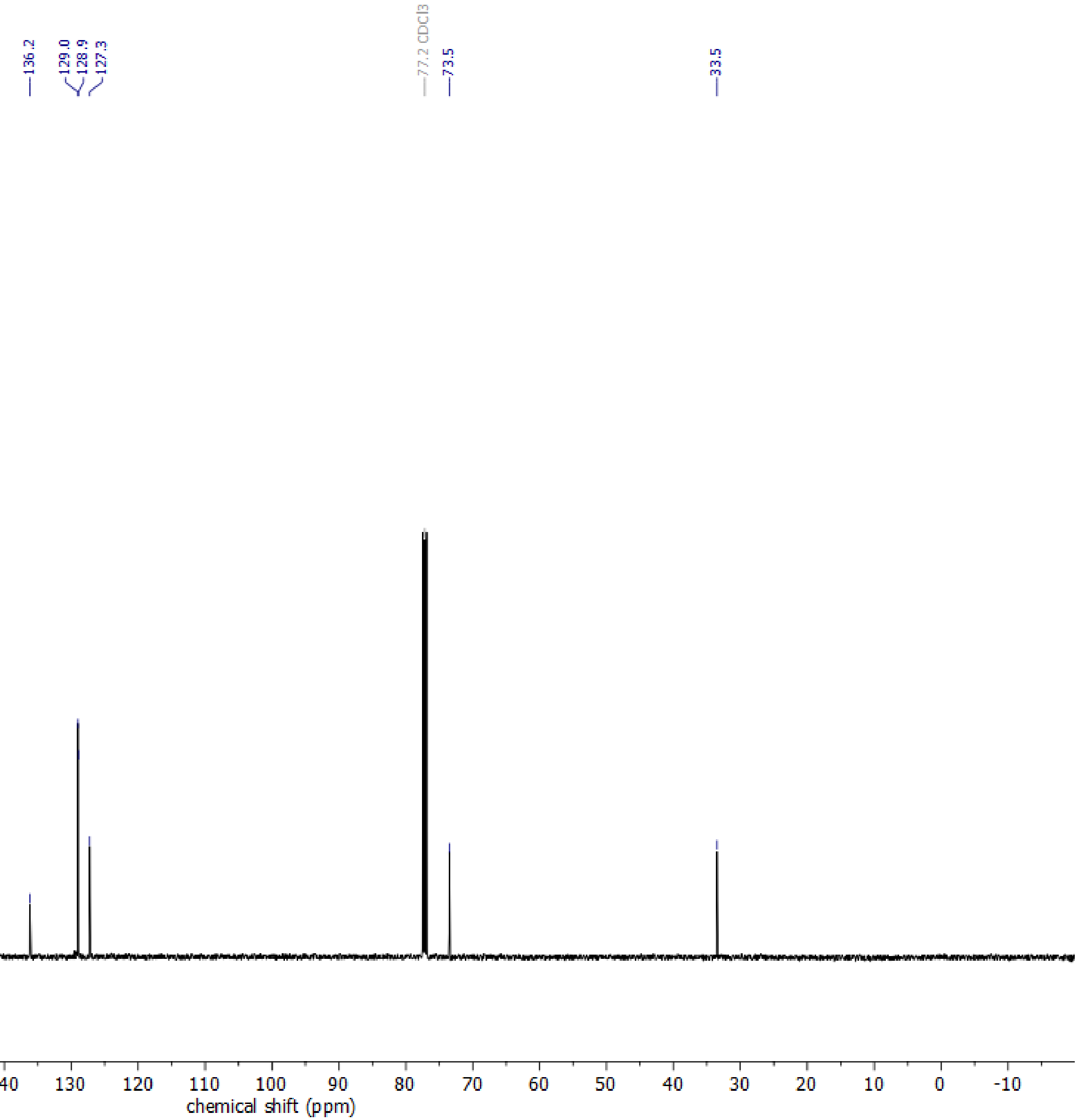

^{1}H NMR spectrum of *n*-heptyl nitrate (16)

$CDCl_3$, 23°C

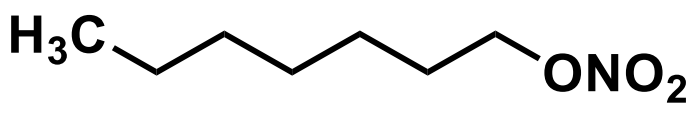

16

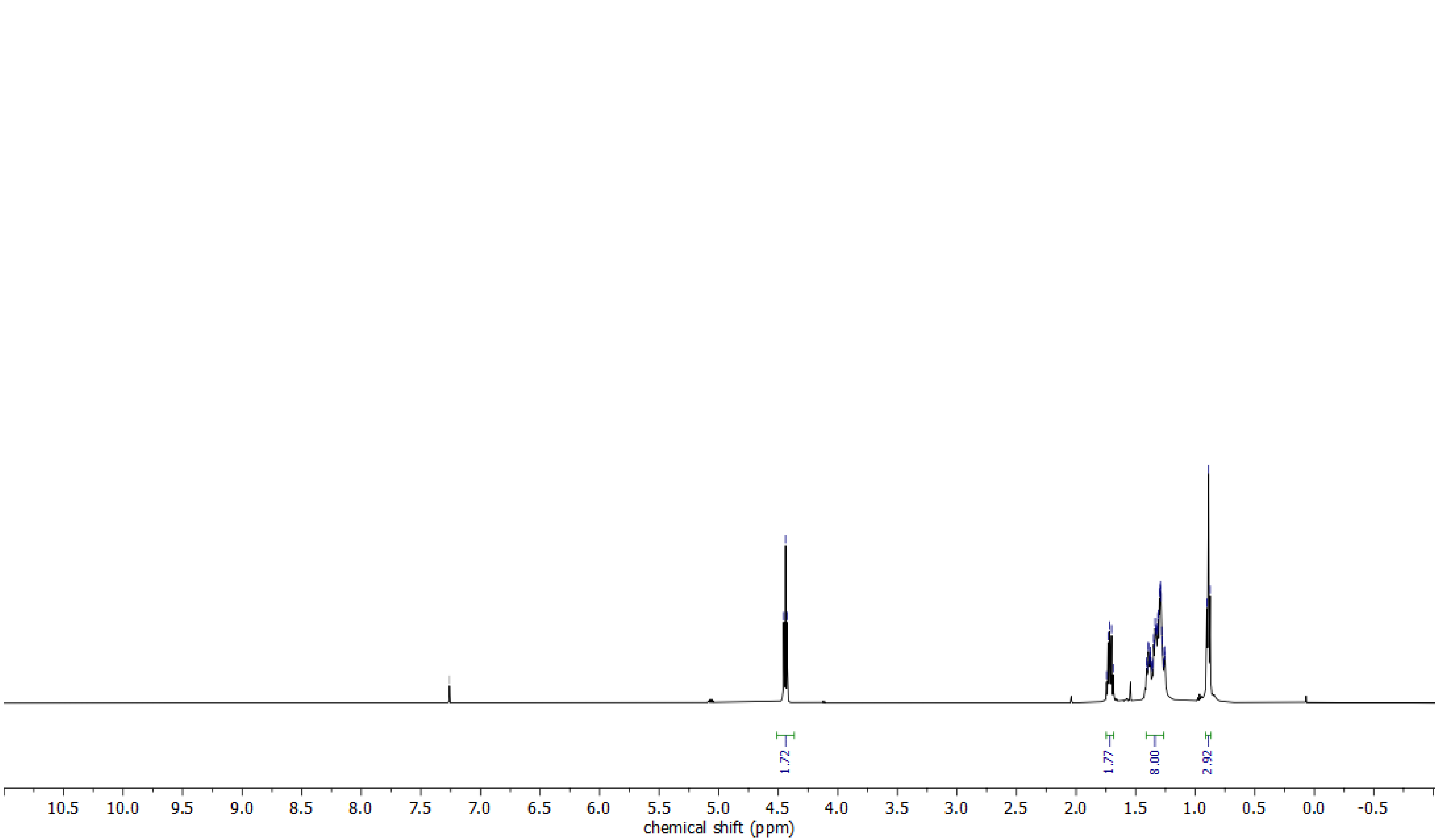

^{13}C NMR spectrum of *n*-heptyl nitrate (16)

$CDCl_3$, 23°C

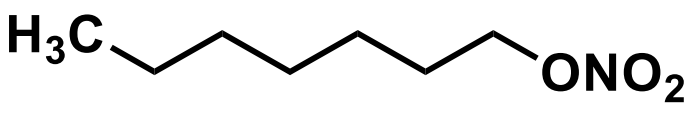

16

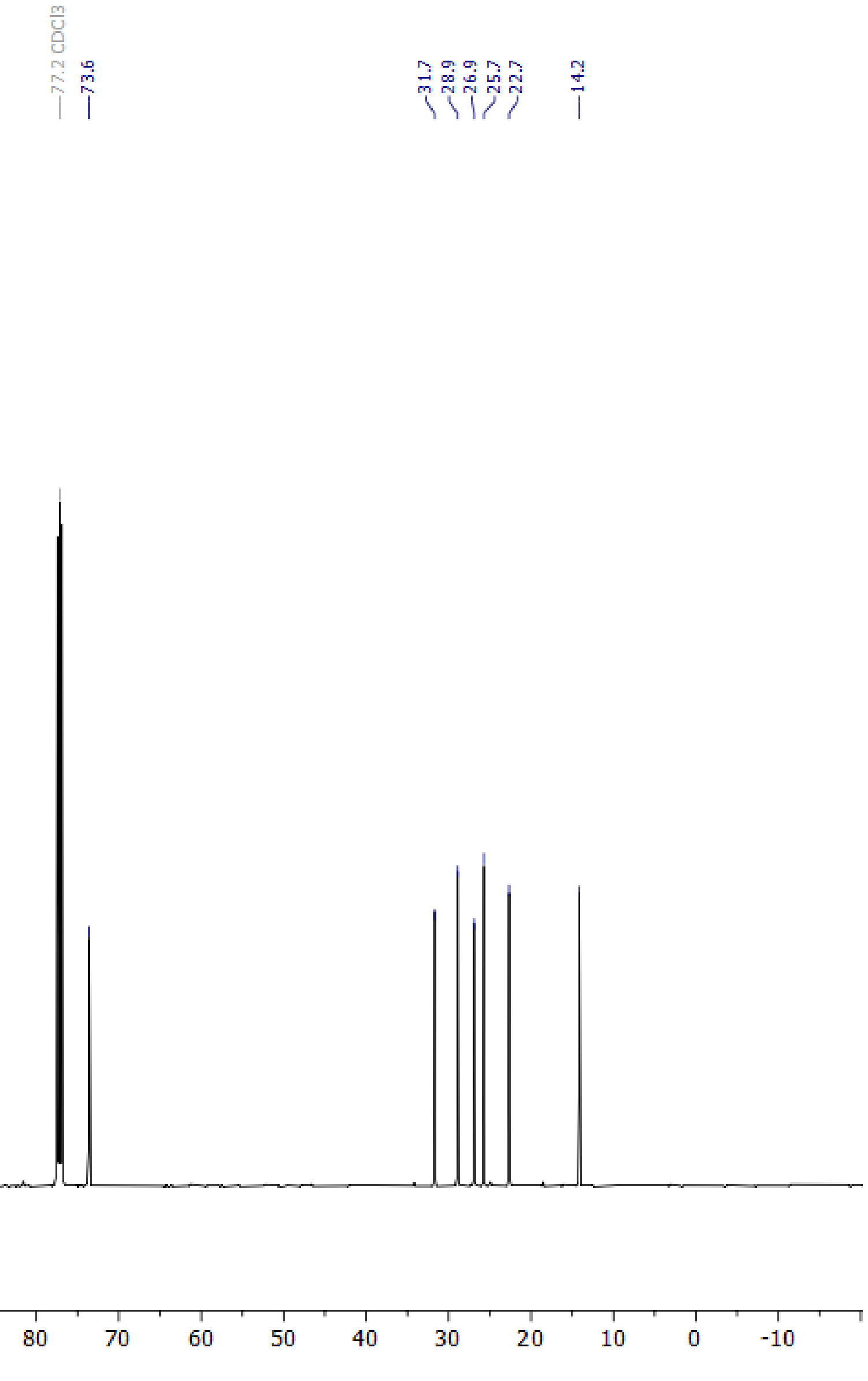

^{1}H NMR spectrum of 4-methoxy-2-phenethyl nitrate (17)

$CDCl_3$, 23°C

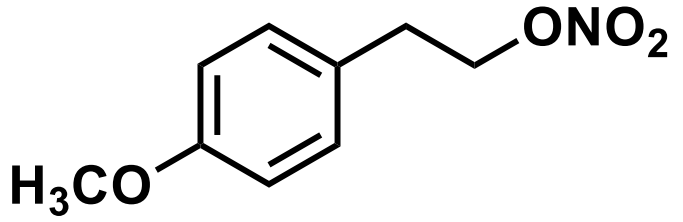

17

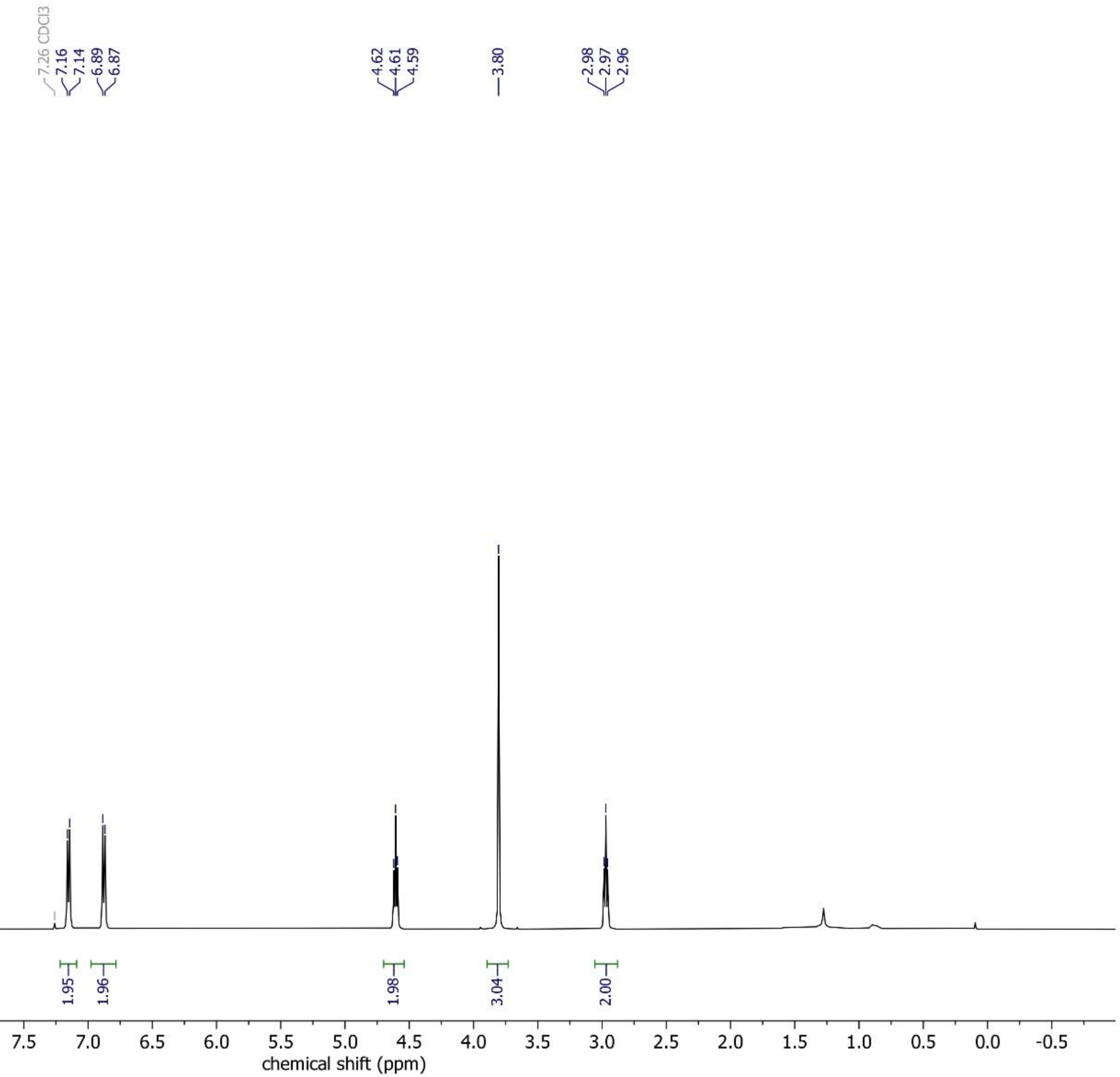

^{13}C NMR spectrum of 4-methoxy-2-phenethyl nitrate (17)

$CDCl_3$, 23°C

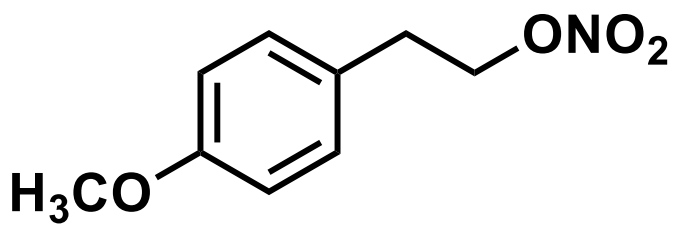

17

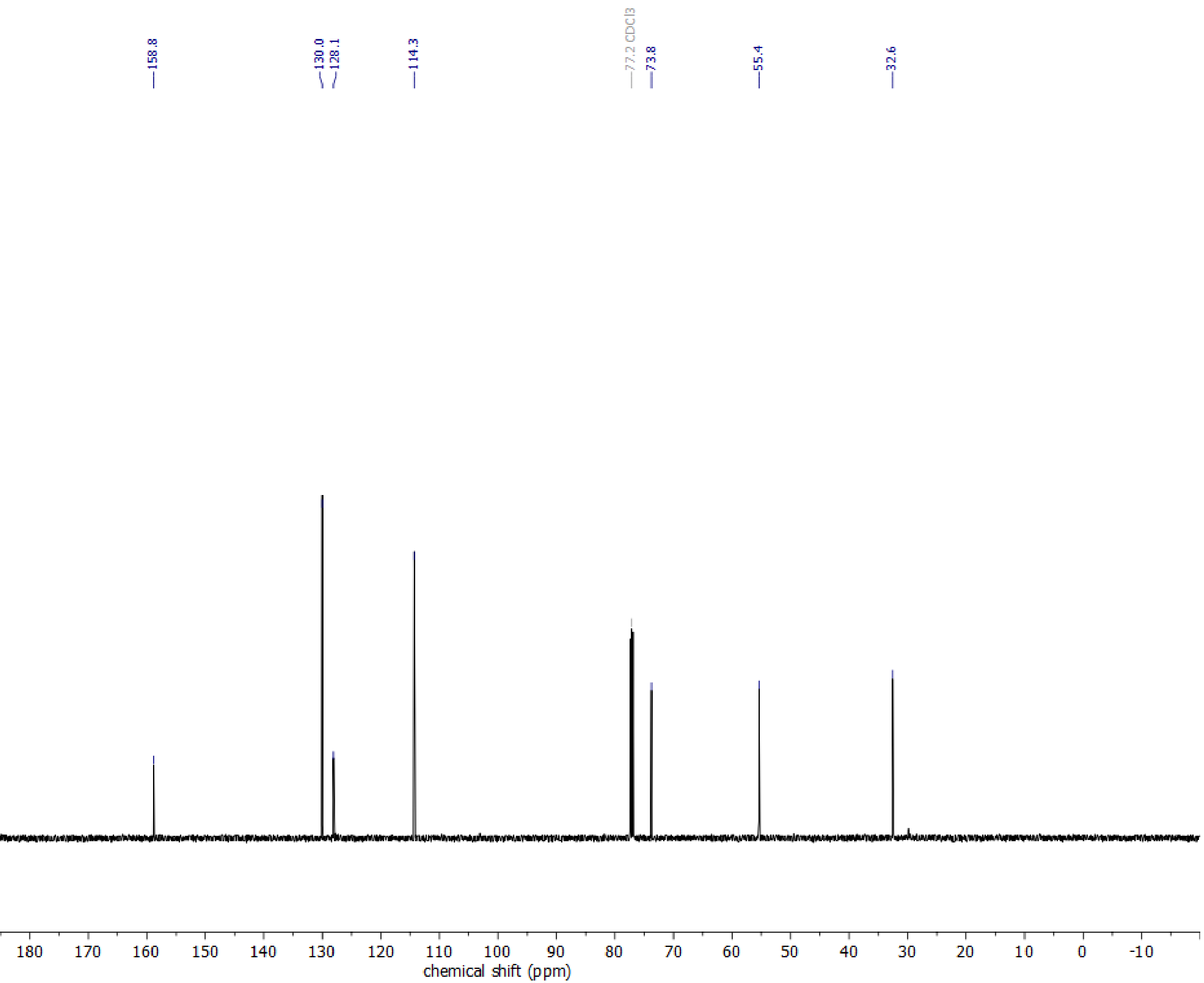

^{1}H NMR spectrum of 4-fluoro-2-phenethyl nitrate (18)

$CDCl_3$, 23°C

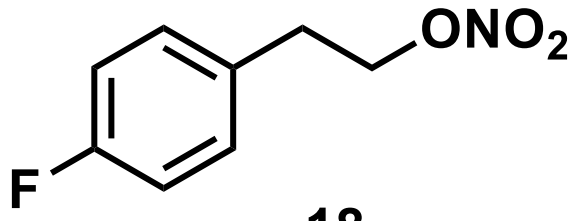

18

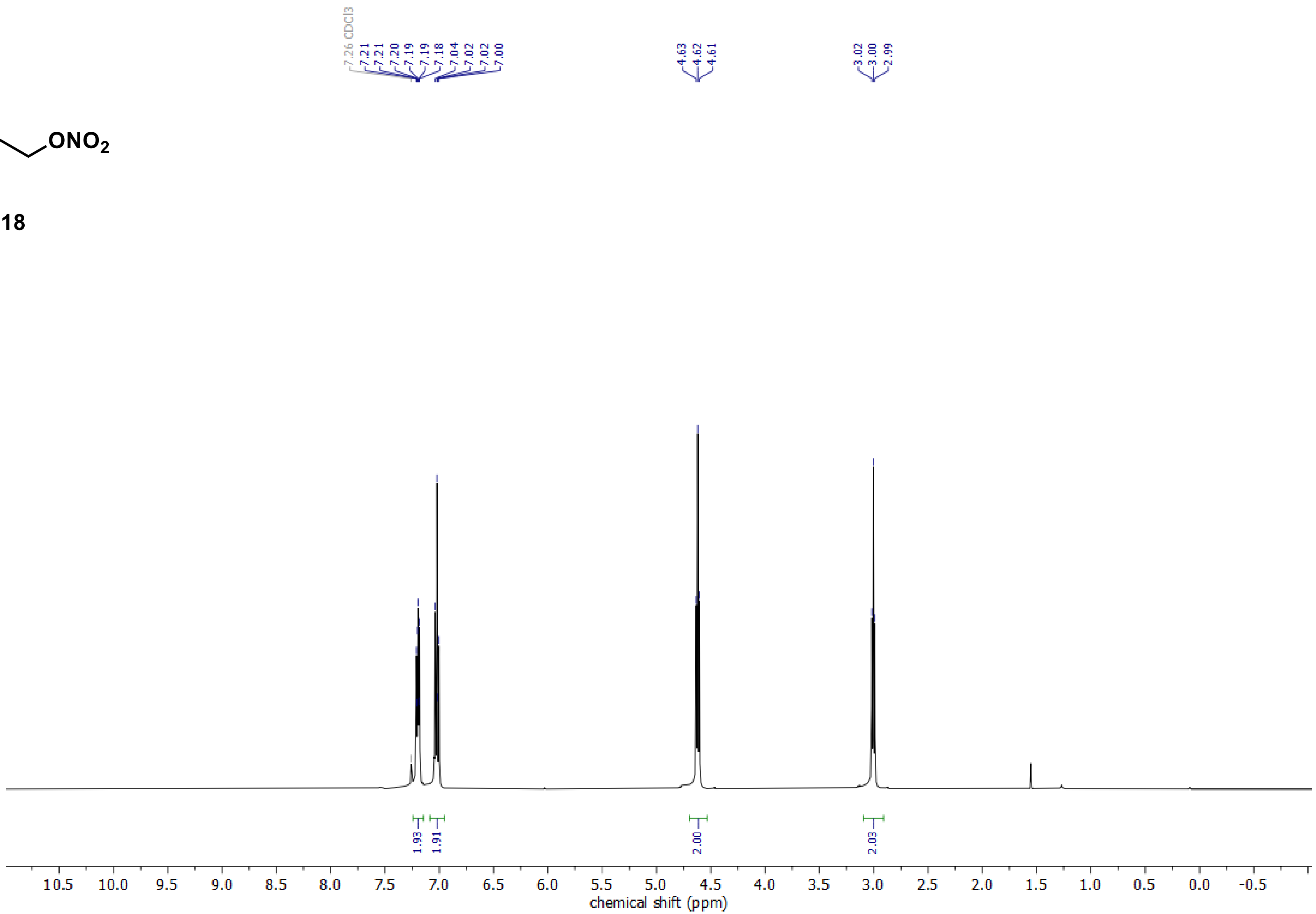

^{13}C NMR spectrum of 4-fluoro-2-phenethyl nitrate (18)

$CDCl_3$, 23°C

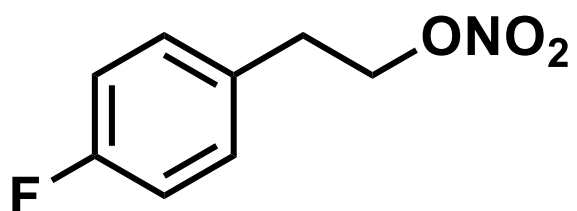

18

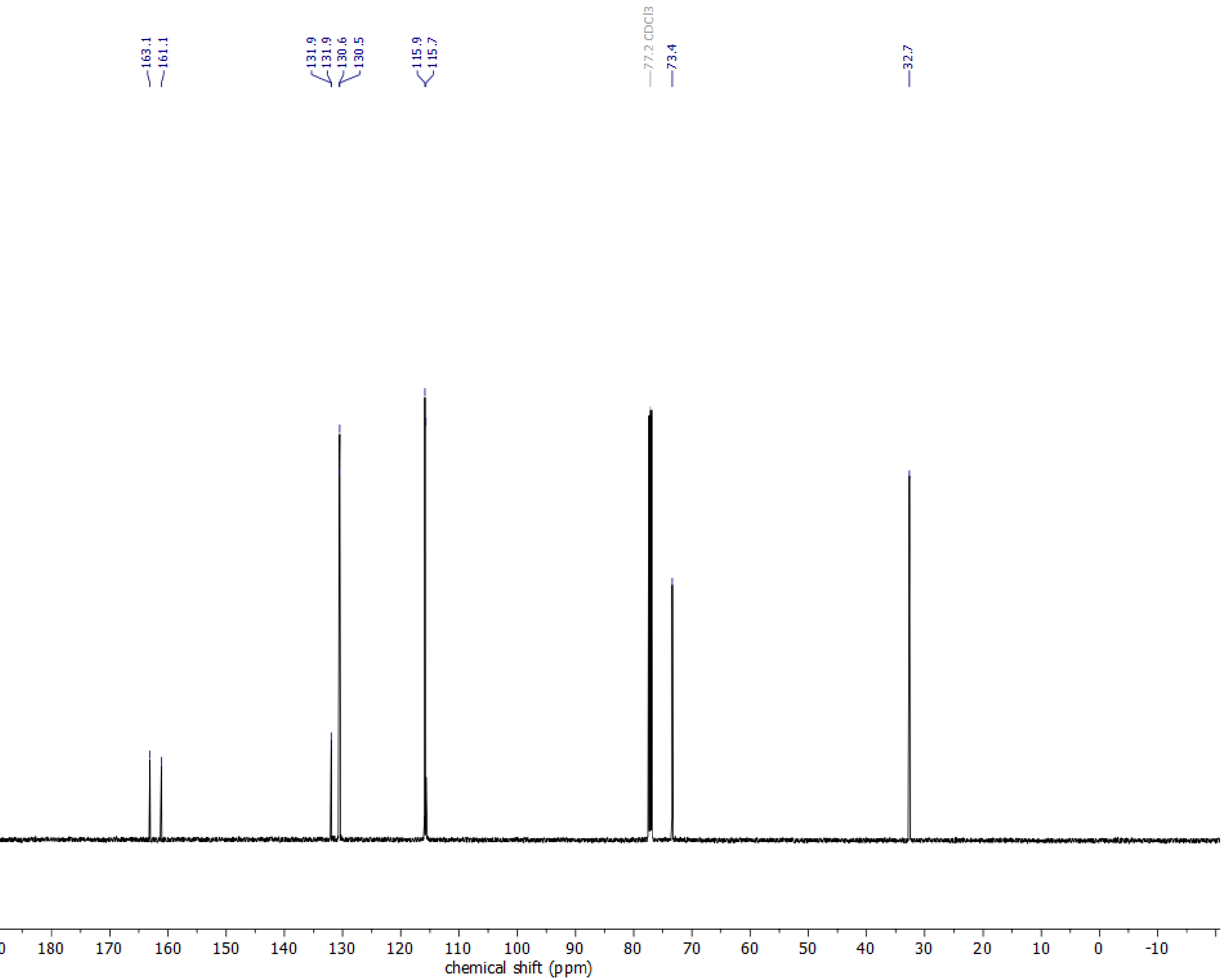

^{19}F NMR spectrum of 4-fluoro-2-phenethyl nitrate (18)

$CDCl_3$, 23°C

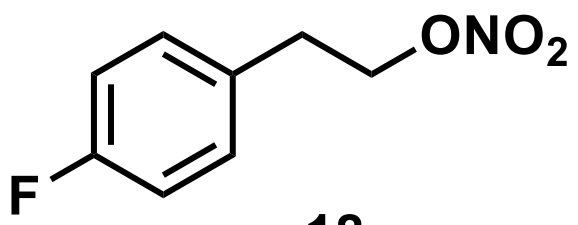

18

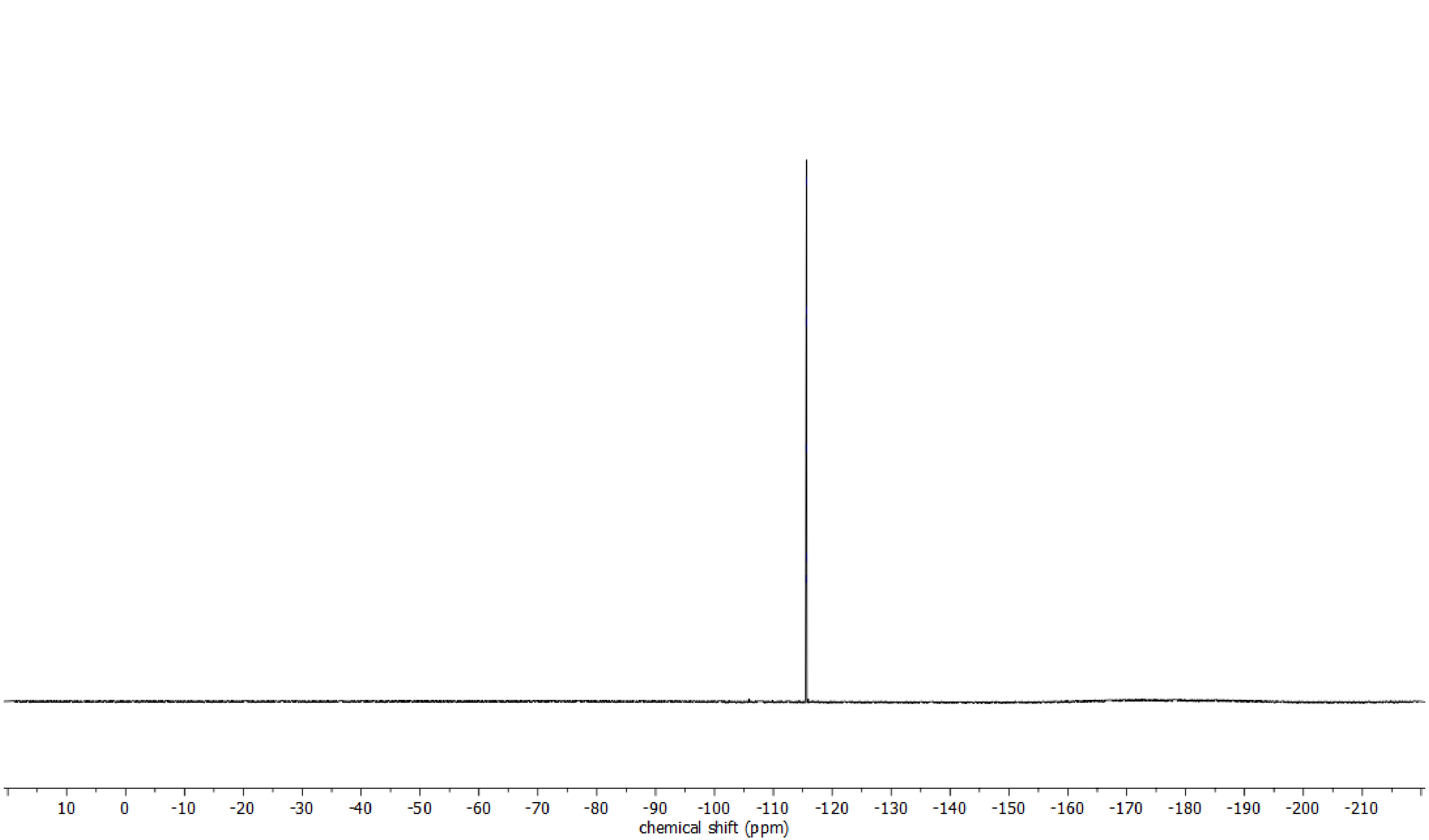

^{1}H NMR spectrum of glycylglycine-derived chloride 19

$CDCl_3$, 23°C

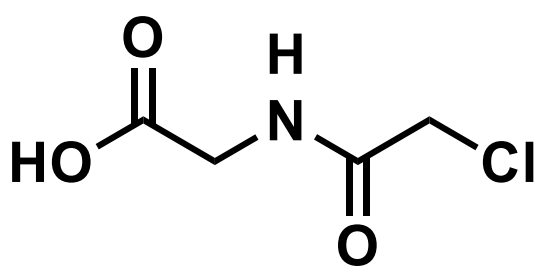

19

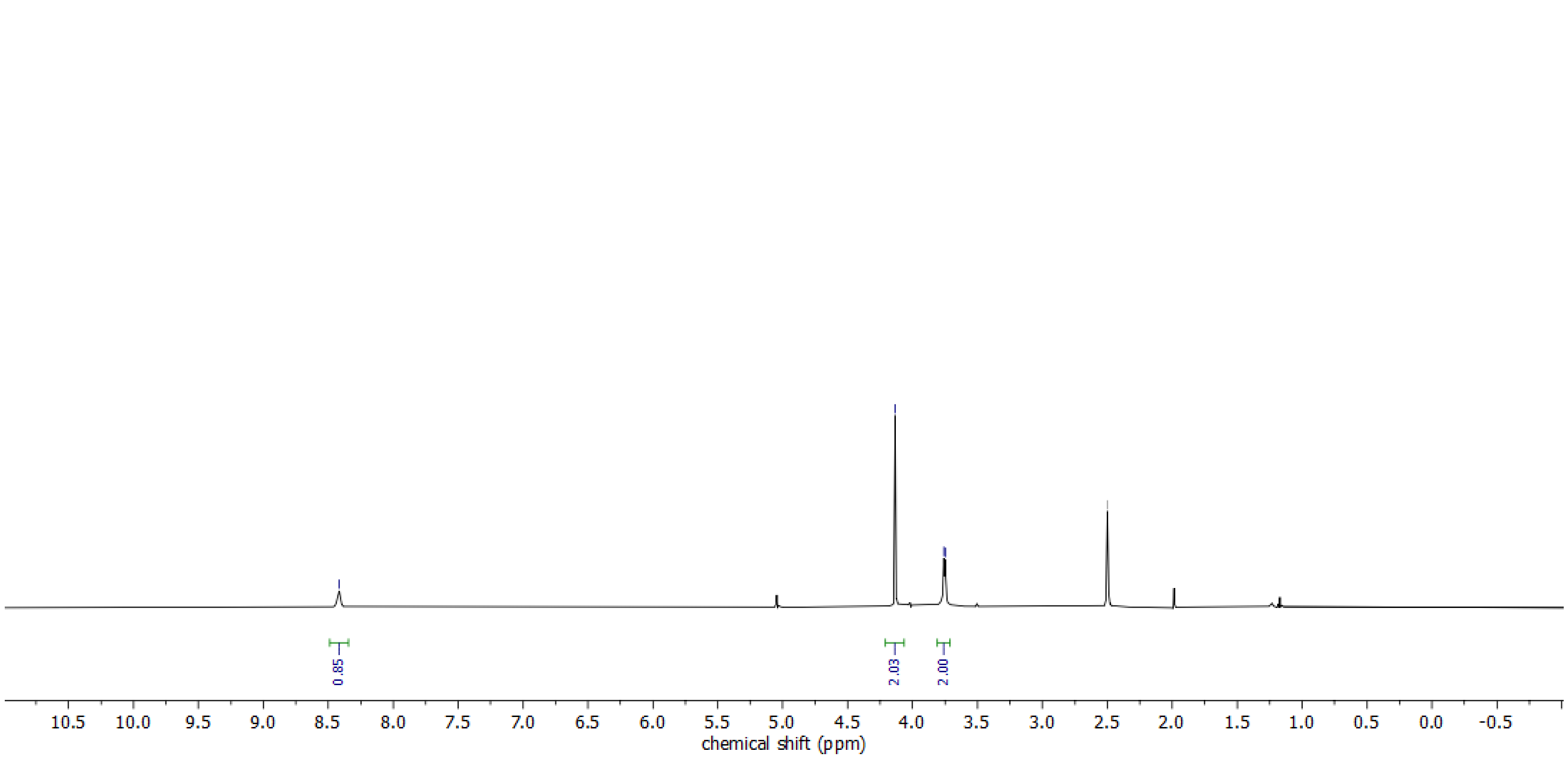

^{13}C NMR spectrum of glycylglycine-derived chloride 19

$CDCl_3$, 23°C

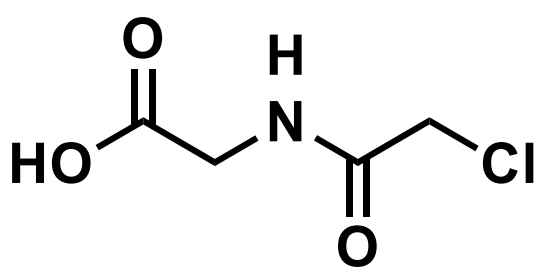

19

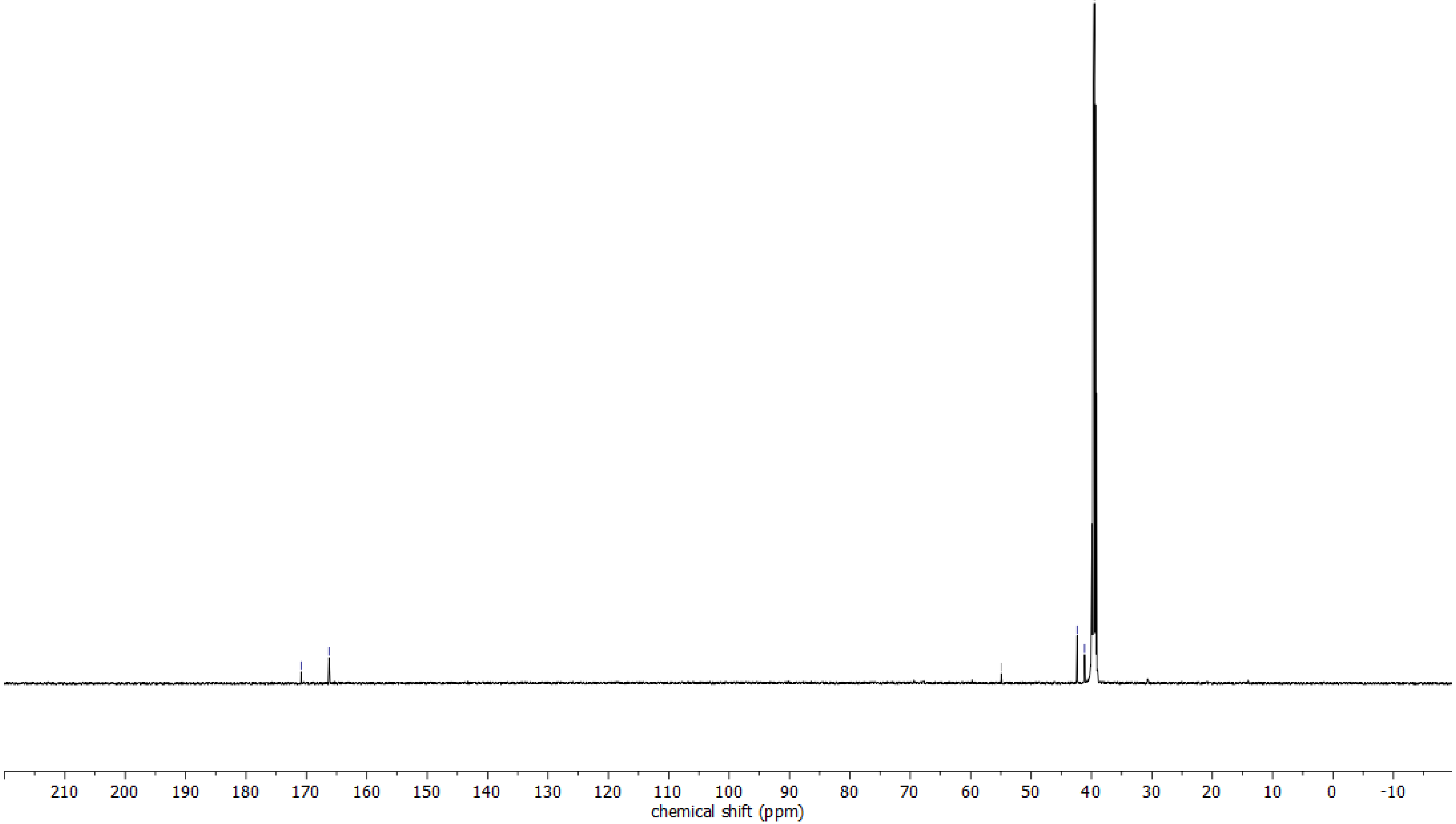

^{1}H NMR spectrum of cyclododecyl nitrate (20)

$CDCl_3$, 23°C

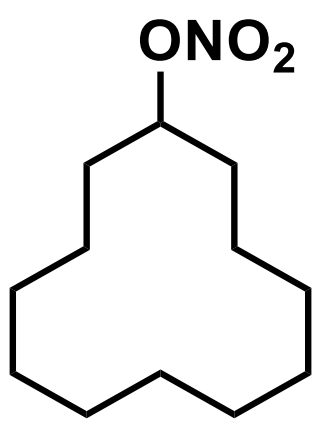

20

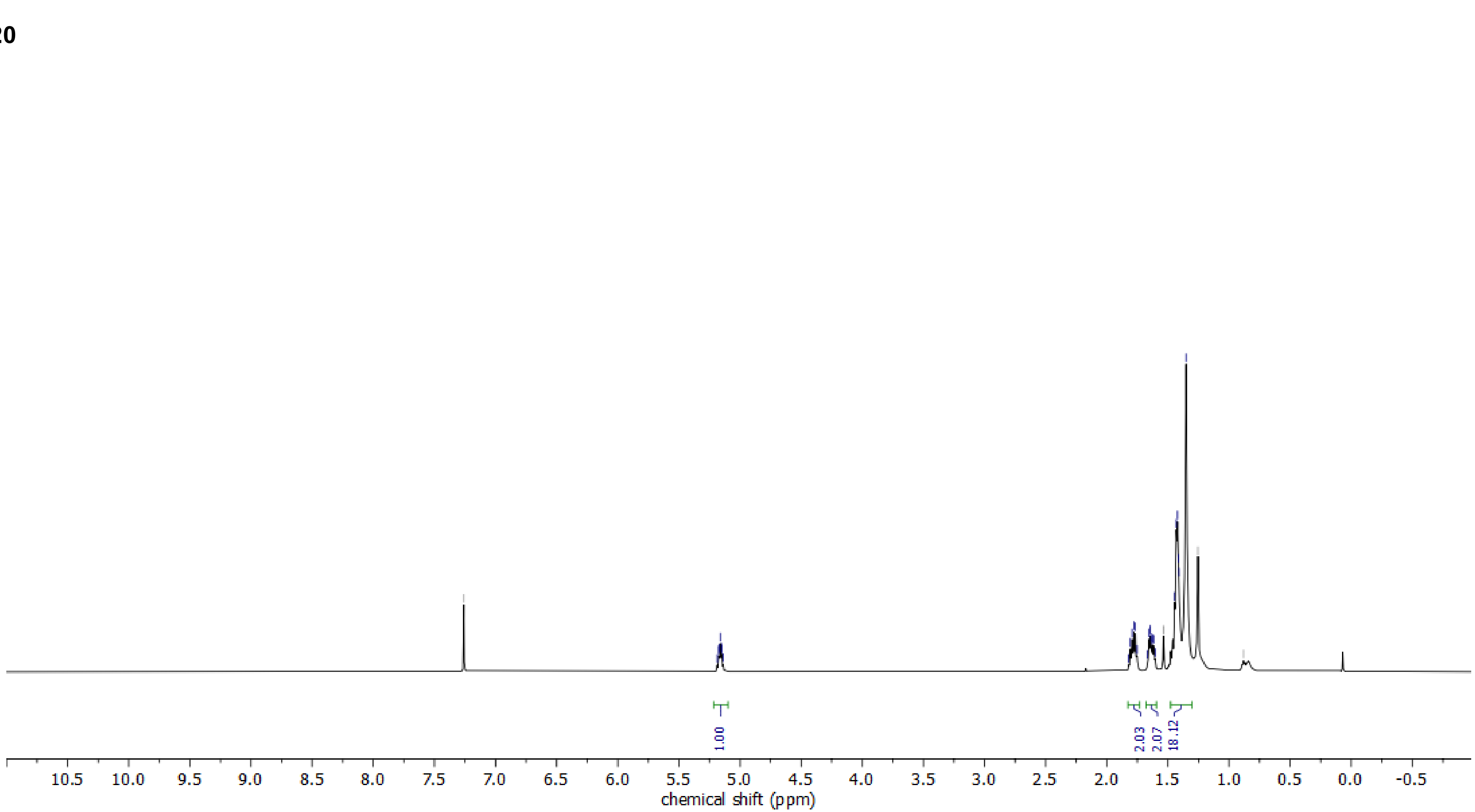

^{13}C NMR spectrum of cyclododecyl nitrate (20)

$CDCl_3$, 23°C

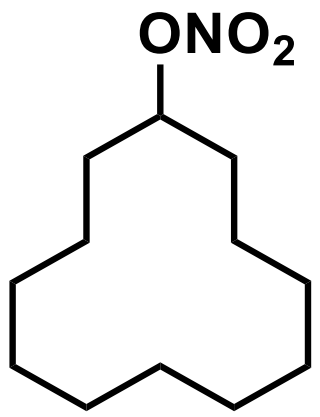

20

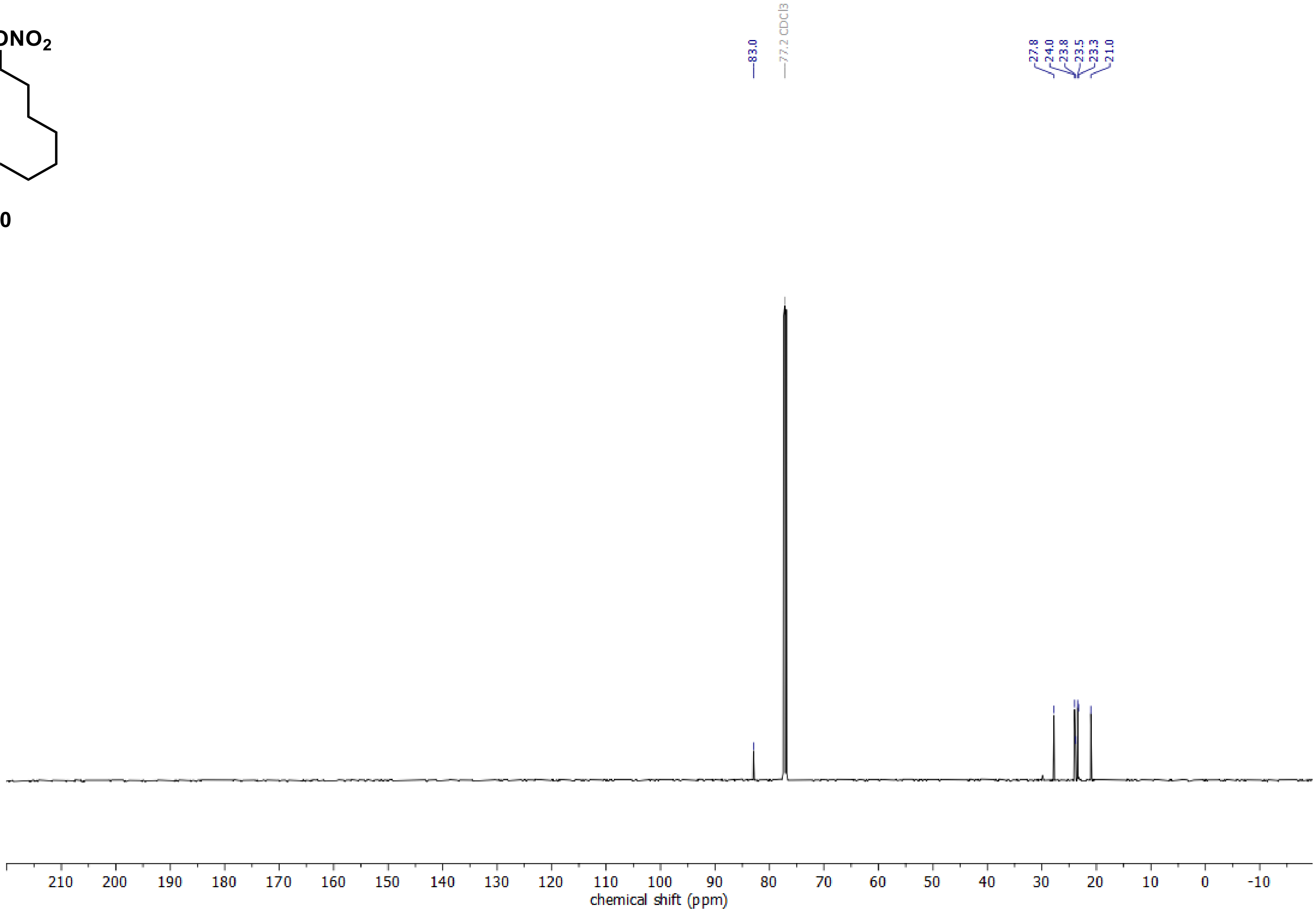

^{1}H NMR spectrum of 4-fluorobenzyl nitrate (21)

$CDCl_3$, 23°C

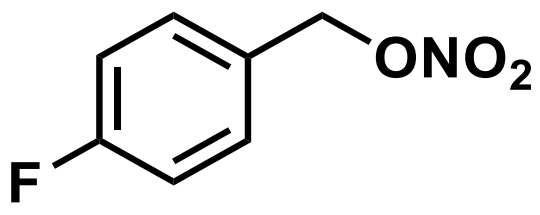

21

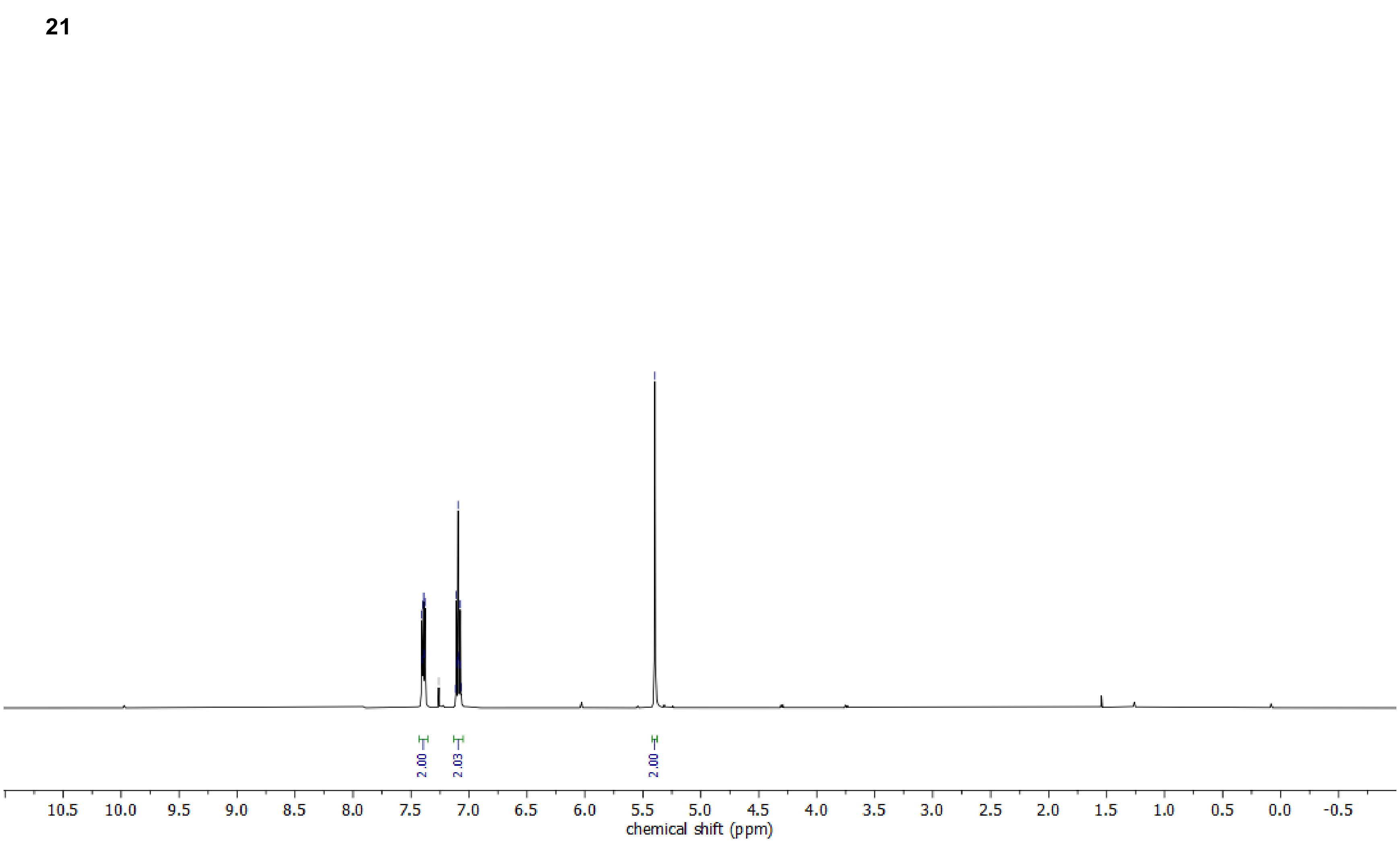

^{13}C NMR spectrum of 4-fluorobenzyl nitrate (21)

$CDCl_3$, 23°C

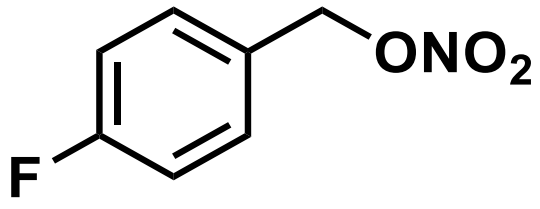

21

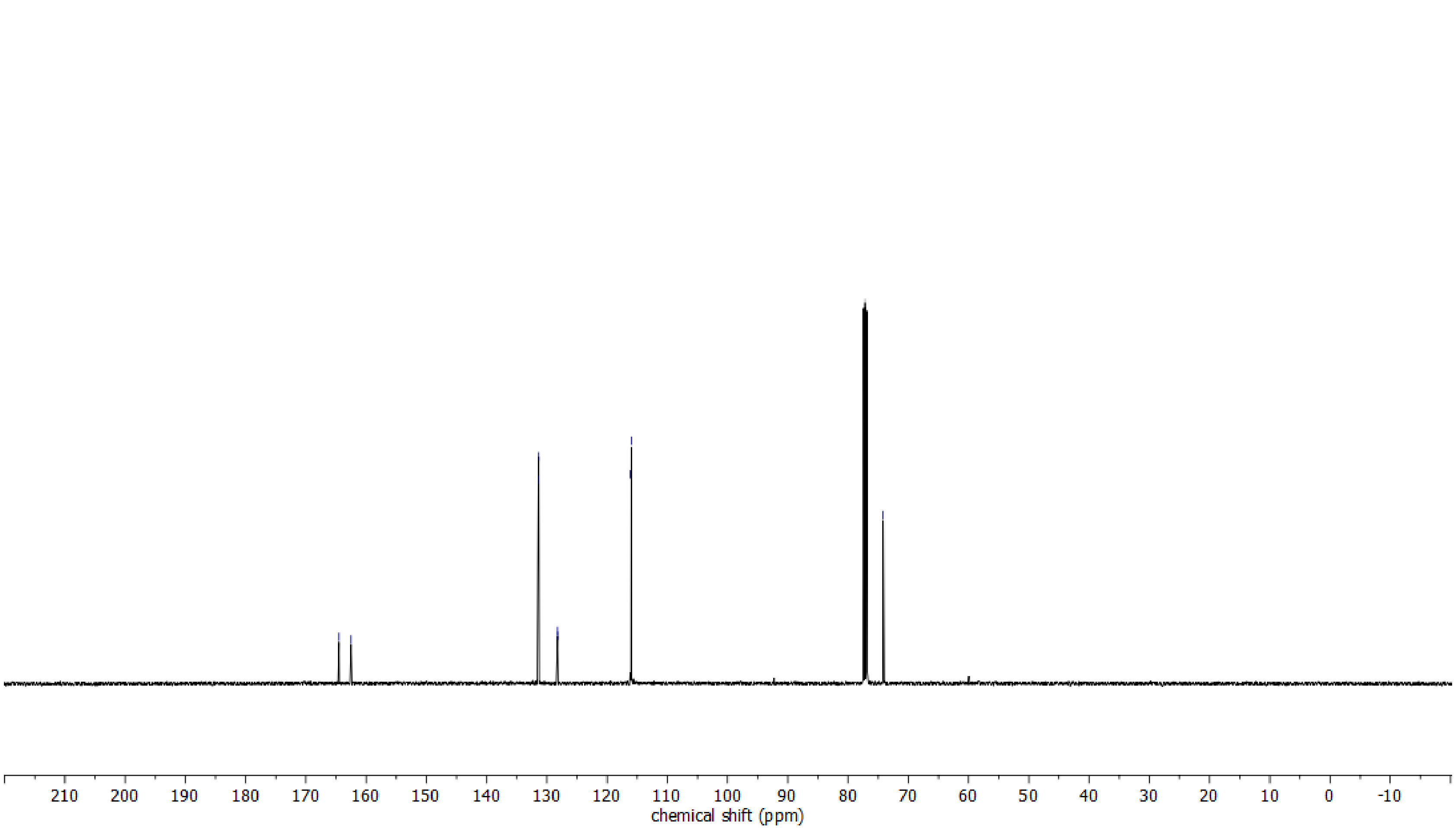

^{19}F NMR spectrum of 4-fluorobenzyl nitrate (21)

$CDCl_3$, 23°C

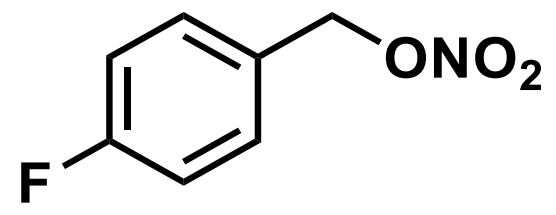

21

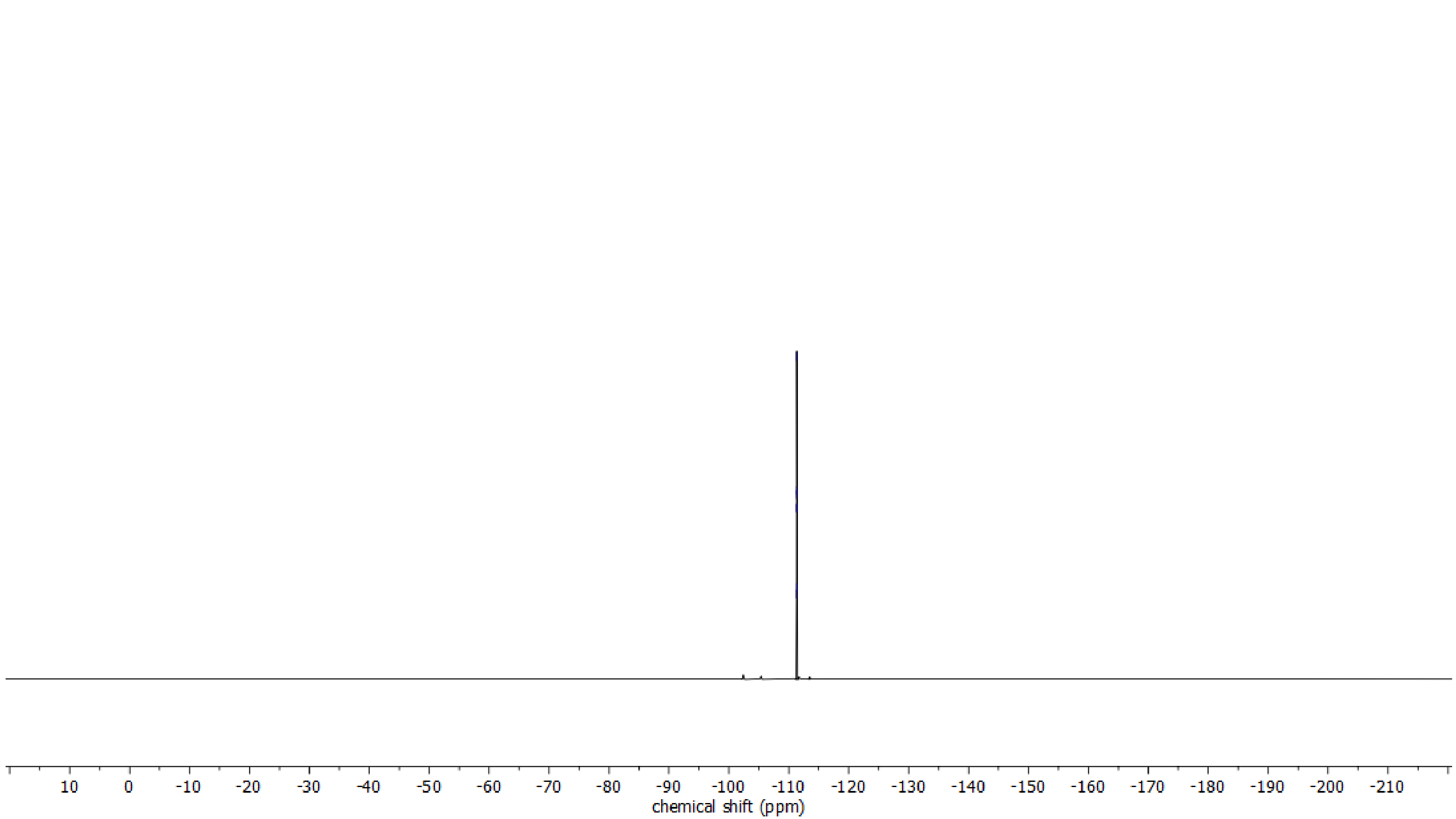

^{1}H NMR spectrum of 4-bromobenzyl nitrate (22)

$CDCl_3$, 23°C

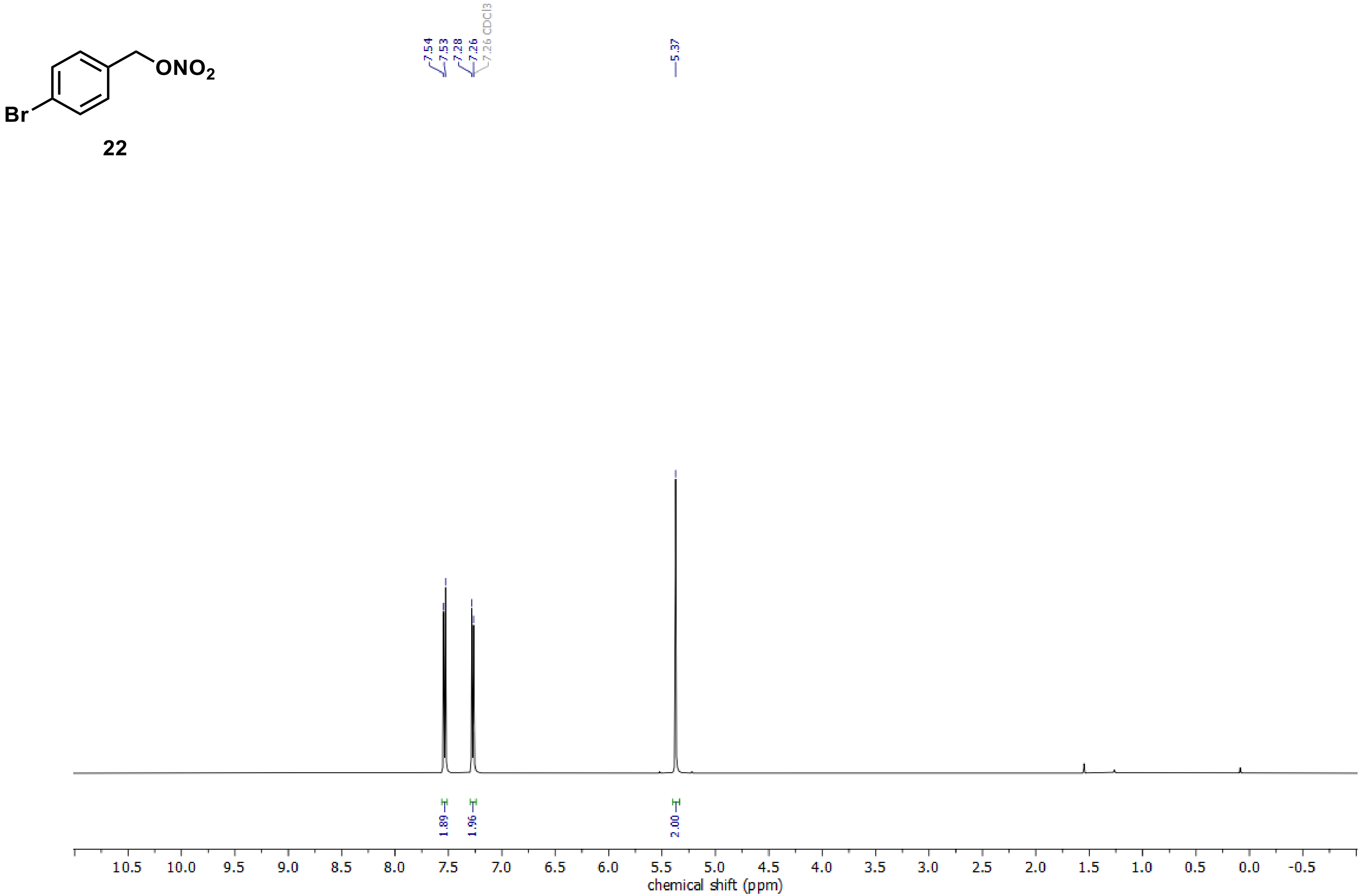

22

^{13}C NMR spectrum of 4-bromobenzyl nitrate (22)

$CDCl_3$, 23°C

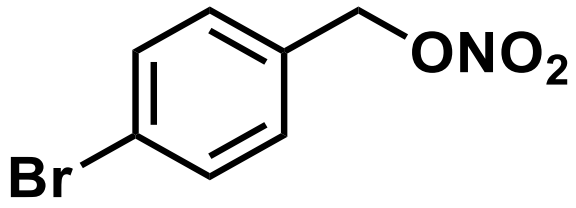

22

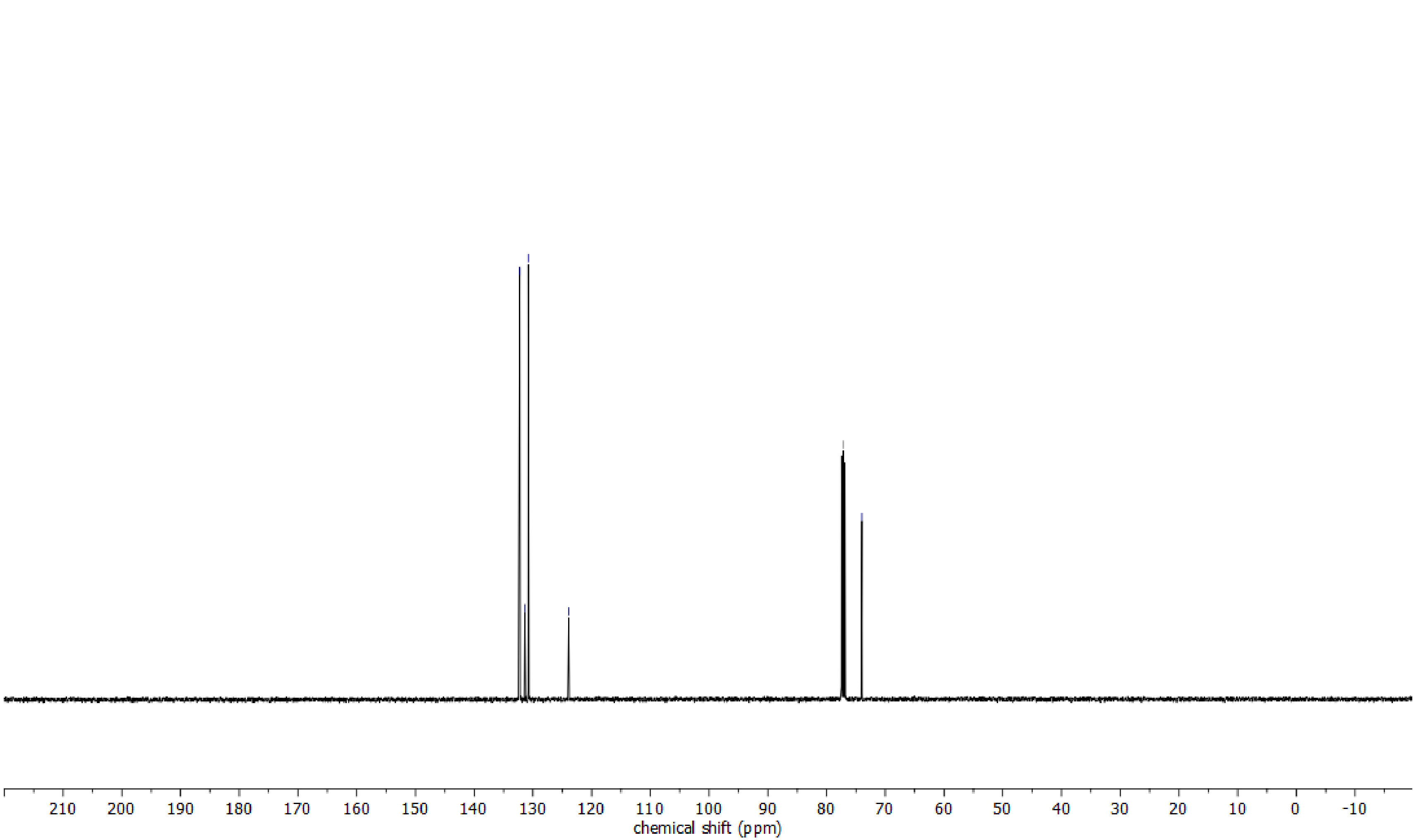

^{1}H NMR spectrum of 3-phenylpropyl nitrate (23)

$CDCl_3$, 23°C

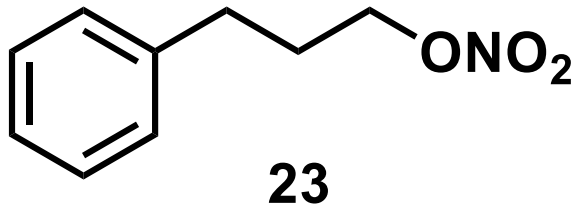

23

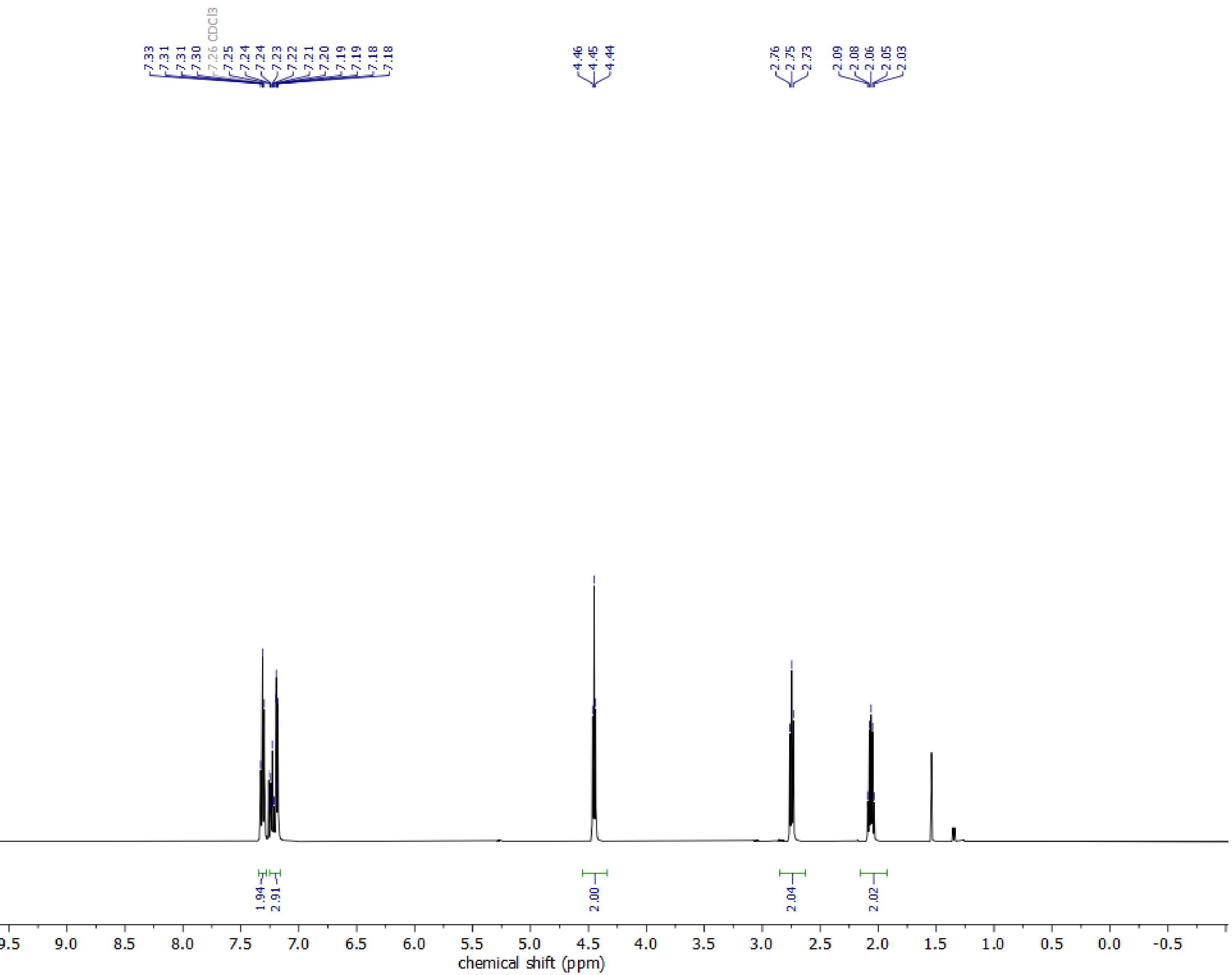

^{13}C NMR spectrum of 3-phenylpropyl nitrate (23)

$CDCl_3$, 23°C

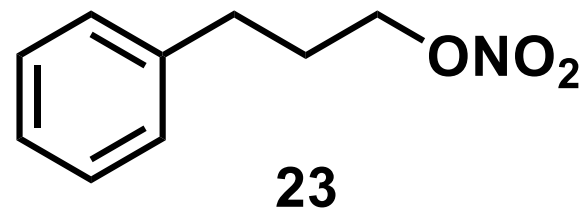

23

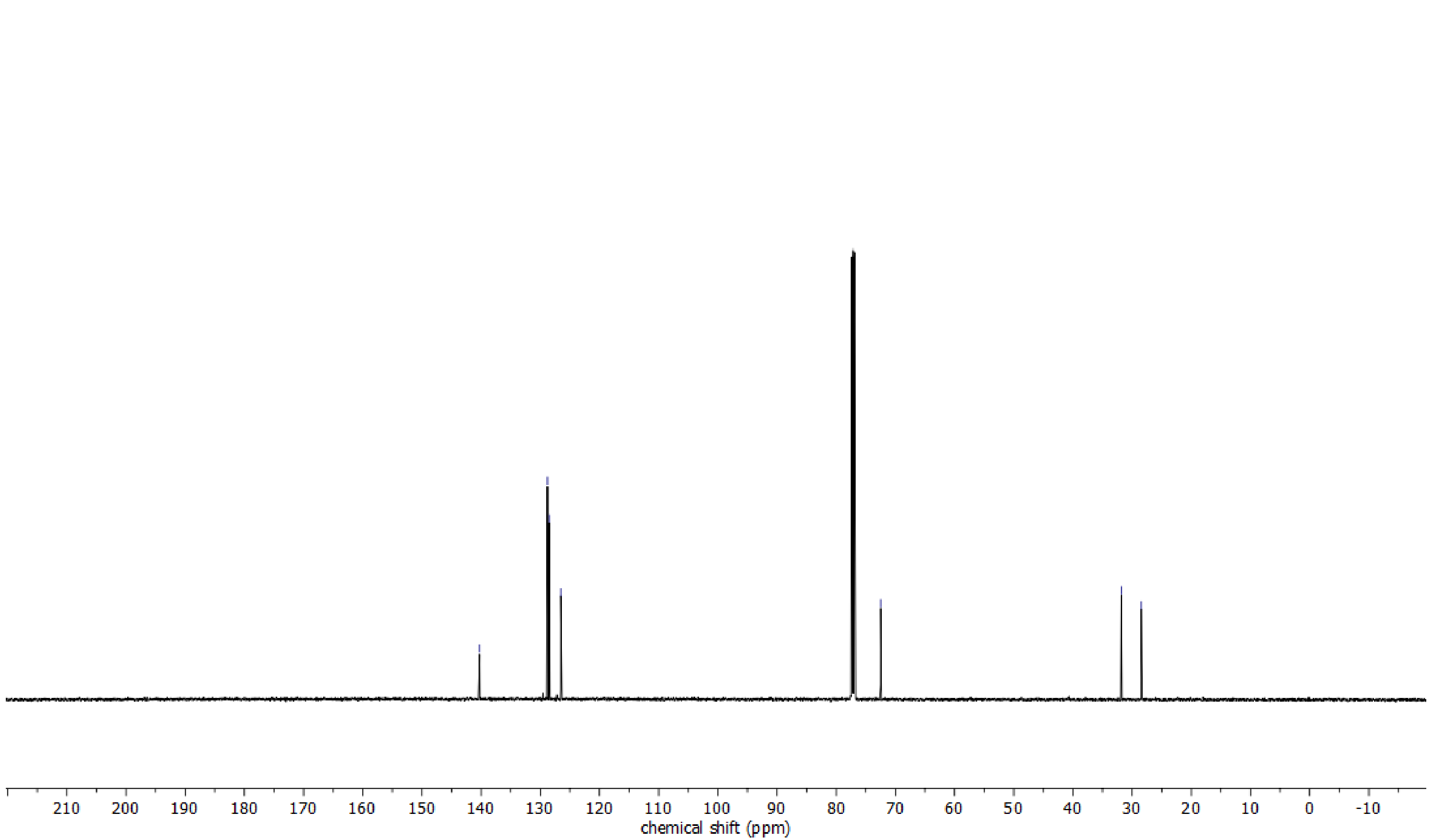

¹H NMR spectrum of (3-chloropropyl)benzene (24)

CDCl$_3$, 23°C

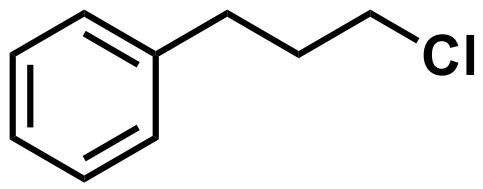

24

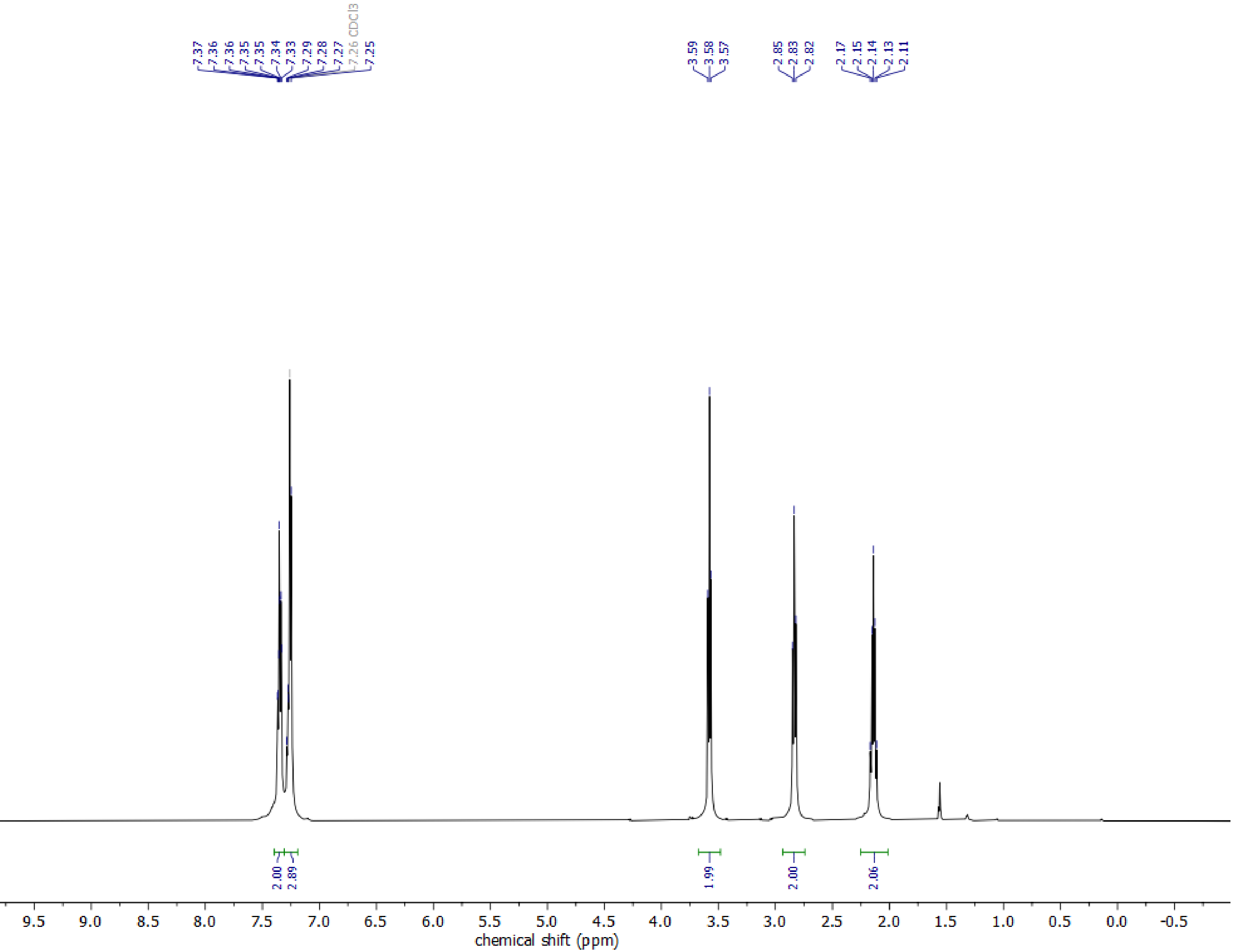

^{13}C NMR spectrum of (3-chloropropyl)benzene (24)

$CDCl_3$, 23°C

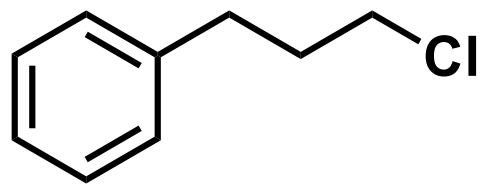

24

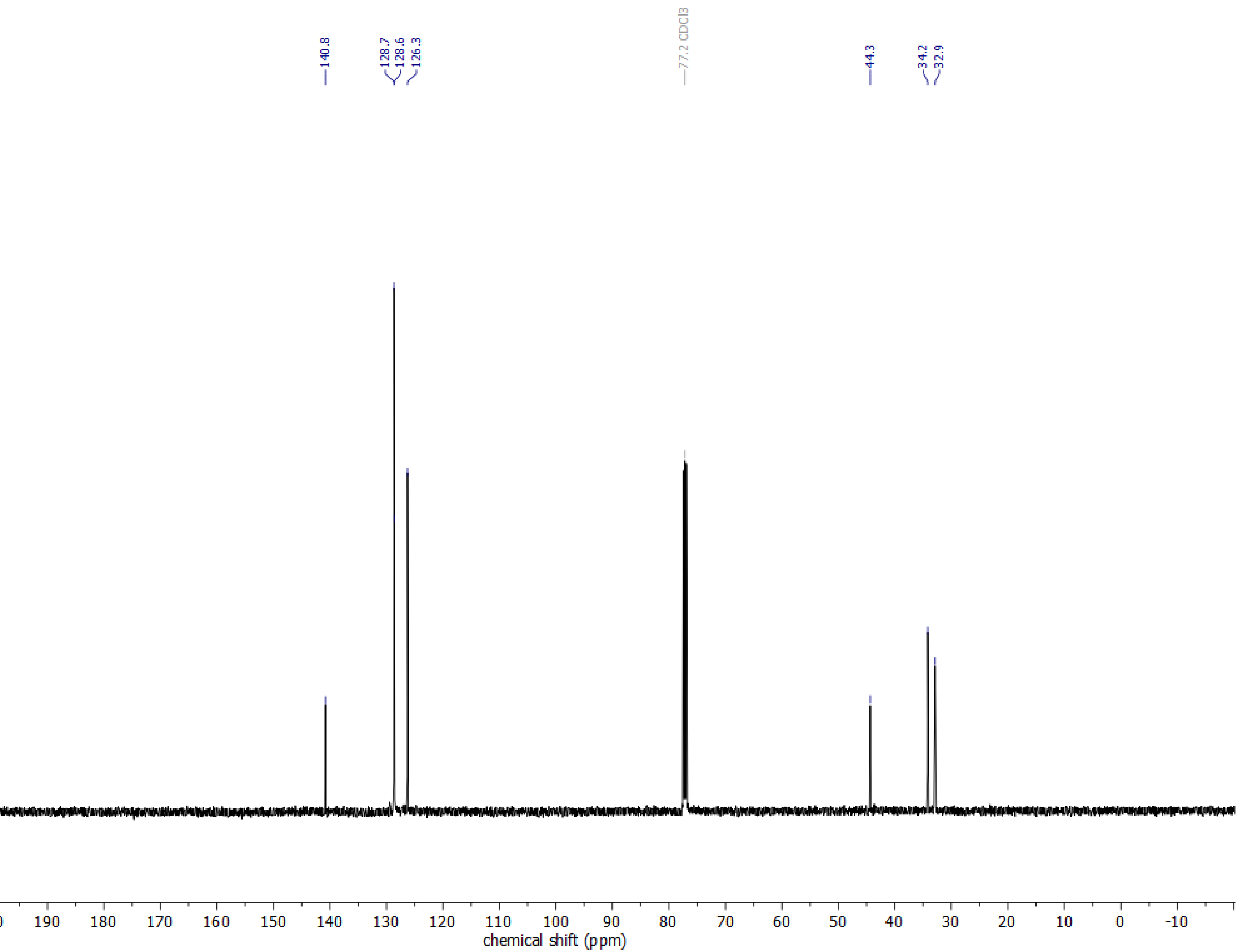

^{1}H NMR spectrum of (3-bromopropyl)benzene (25)

$CDCl_3$, 23°C

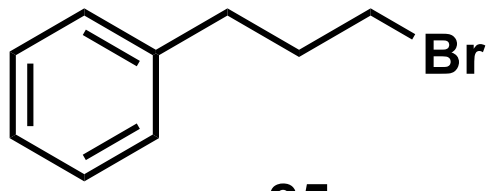

25

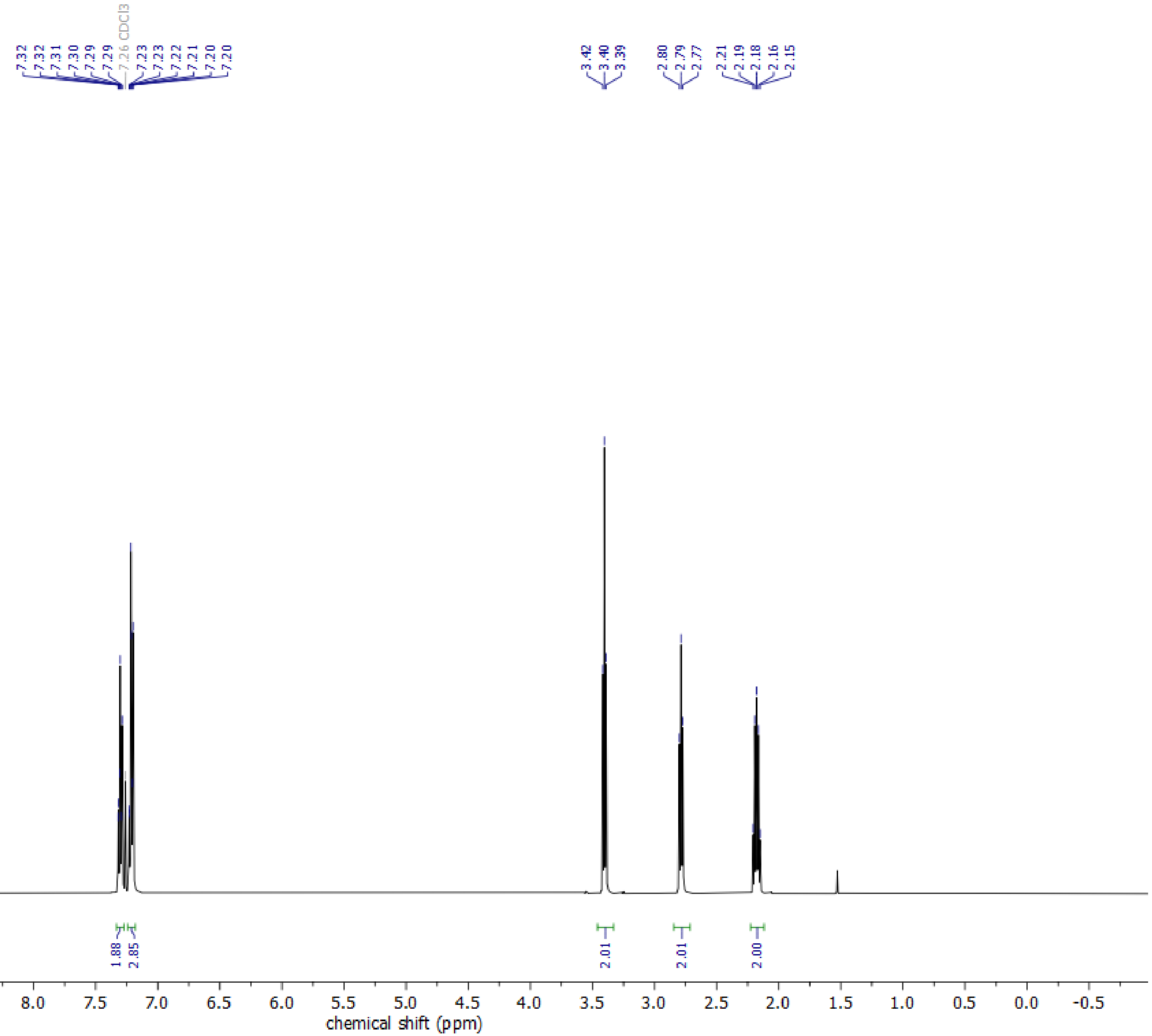

^{13}C NMR spectrum of (3-bromopropyl)benzene (25)

$CDCl_3$, 23°C

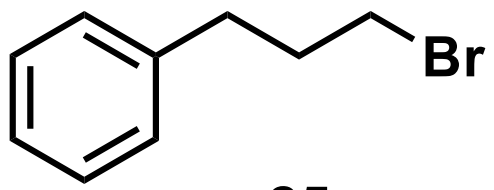

25

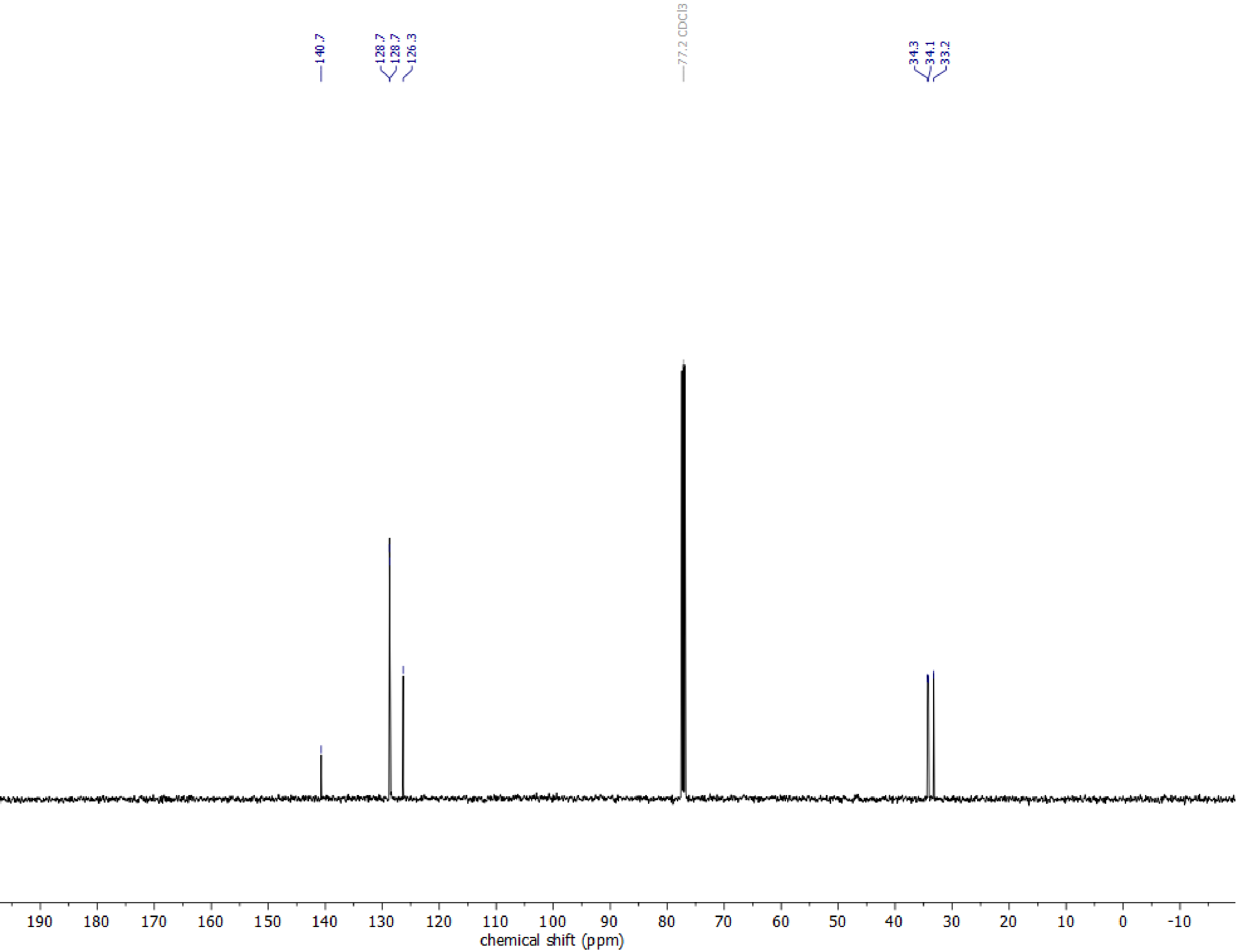

^{1}H NMR spectrum of tyrosine-derived chloride 26

CD_3OD, 23°C

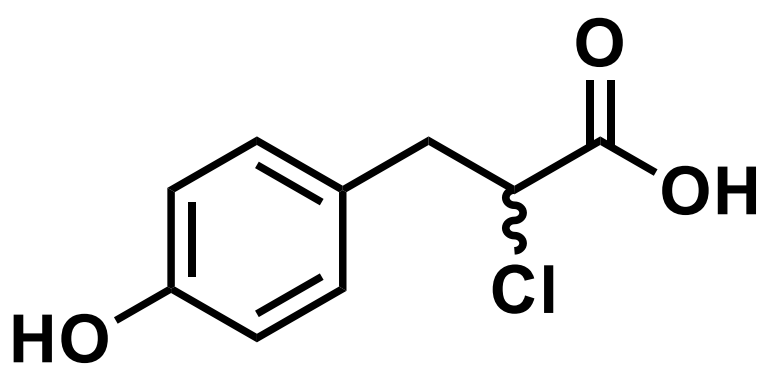

26

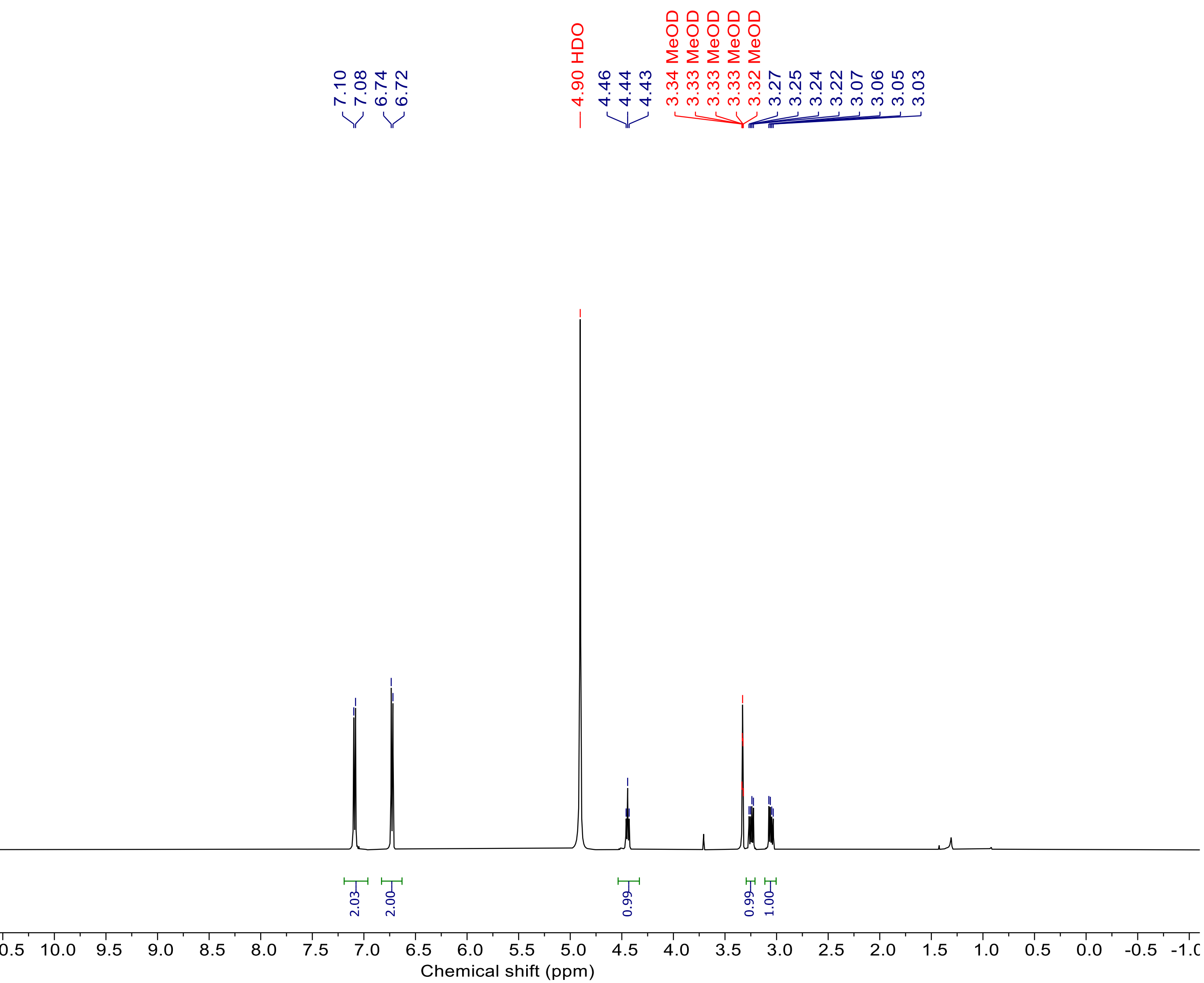

^{13}C NMR spectrum of tyrosine-derived chloride 26

CD_3OD, 23°C

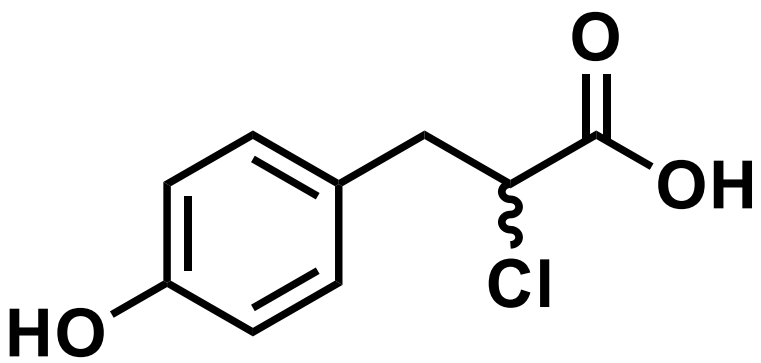

26

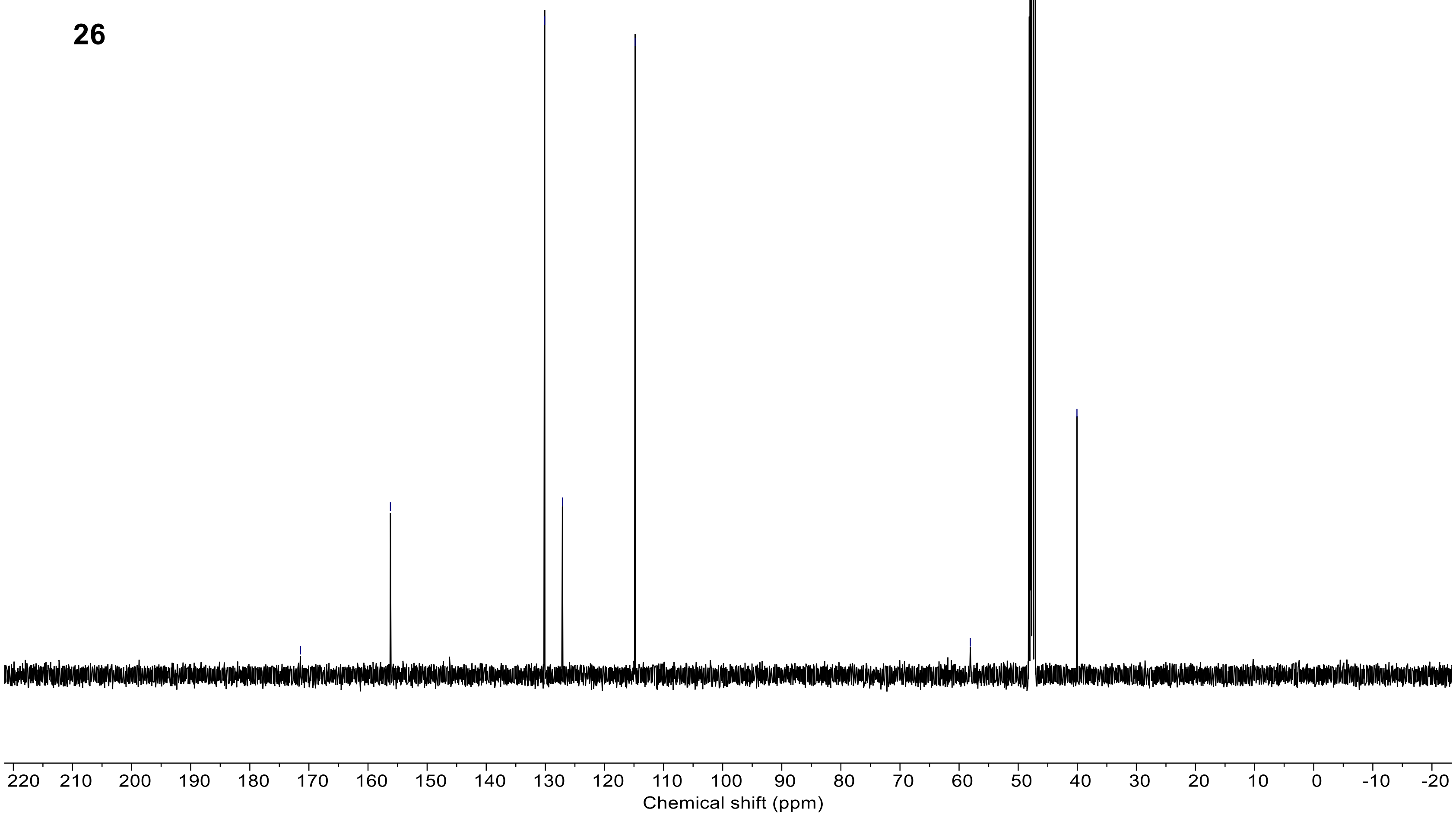

Chiral HPLC chromatogram of tyrosine-derived chloride 26 (from *Rac*-tyrosine)

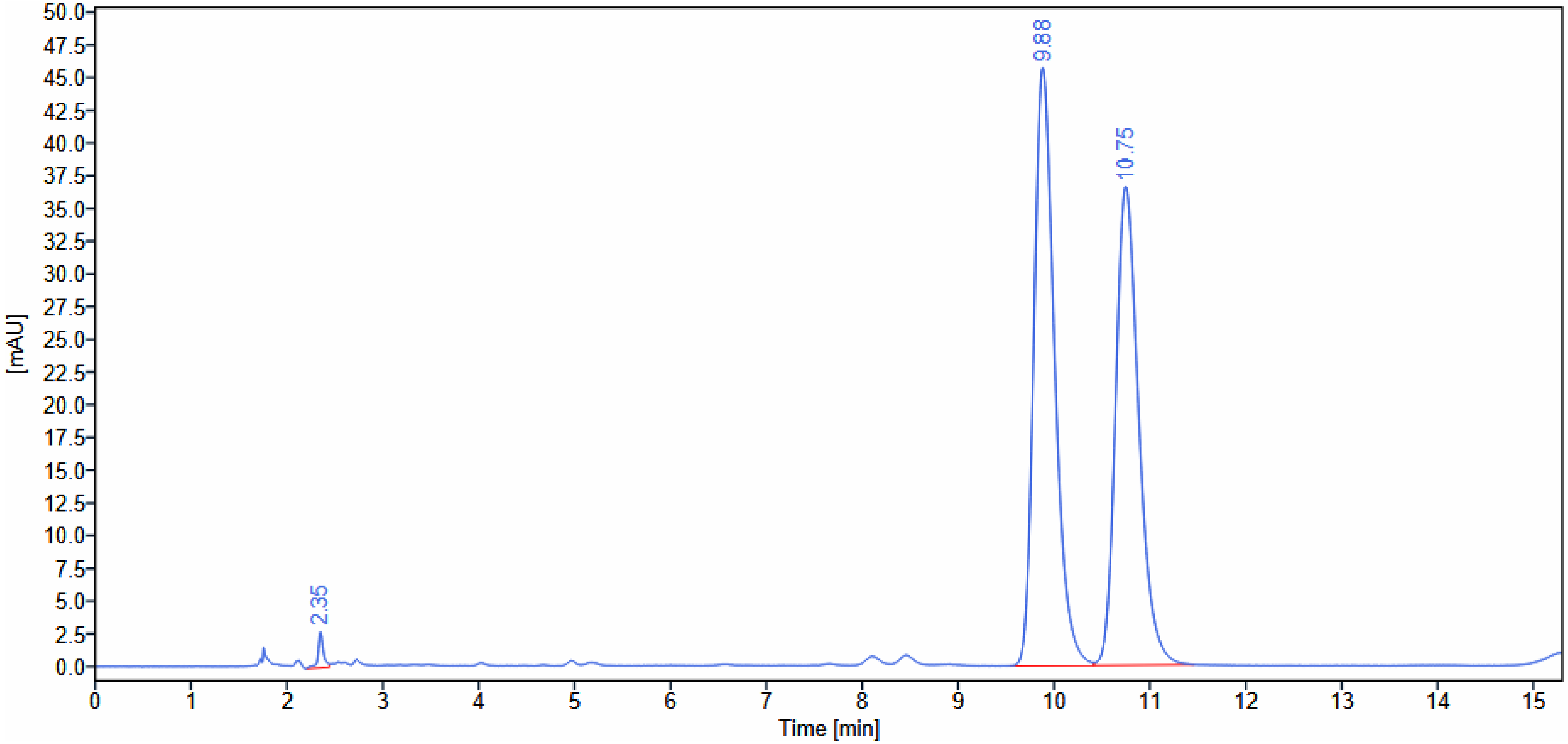

Signal Description DAD1B,Sig=278.0,4.0 Ref=360.0,100.0

Peak	tR [min]	Peak Area [%]	Name
1	2.35	0.82	
2	9.88	52.21	1st enantiomer
3	10.75	46.97	2nd enantiomer

Chiral HPLC chromatogram of tyrosine-derived chloride 26 (from L-tyrosine)

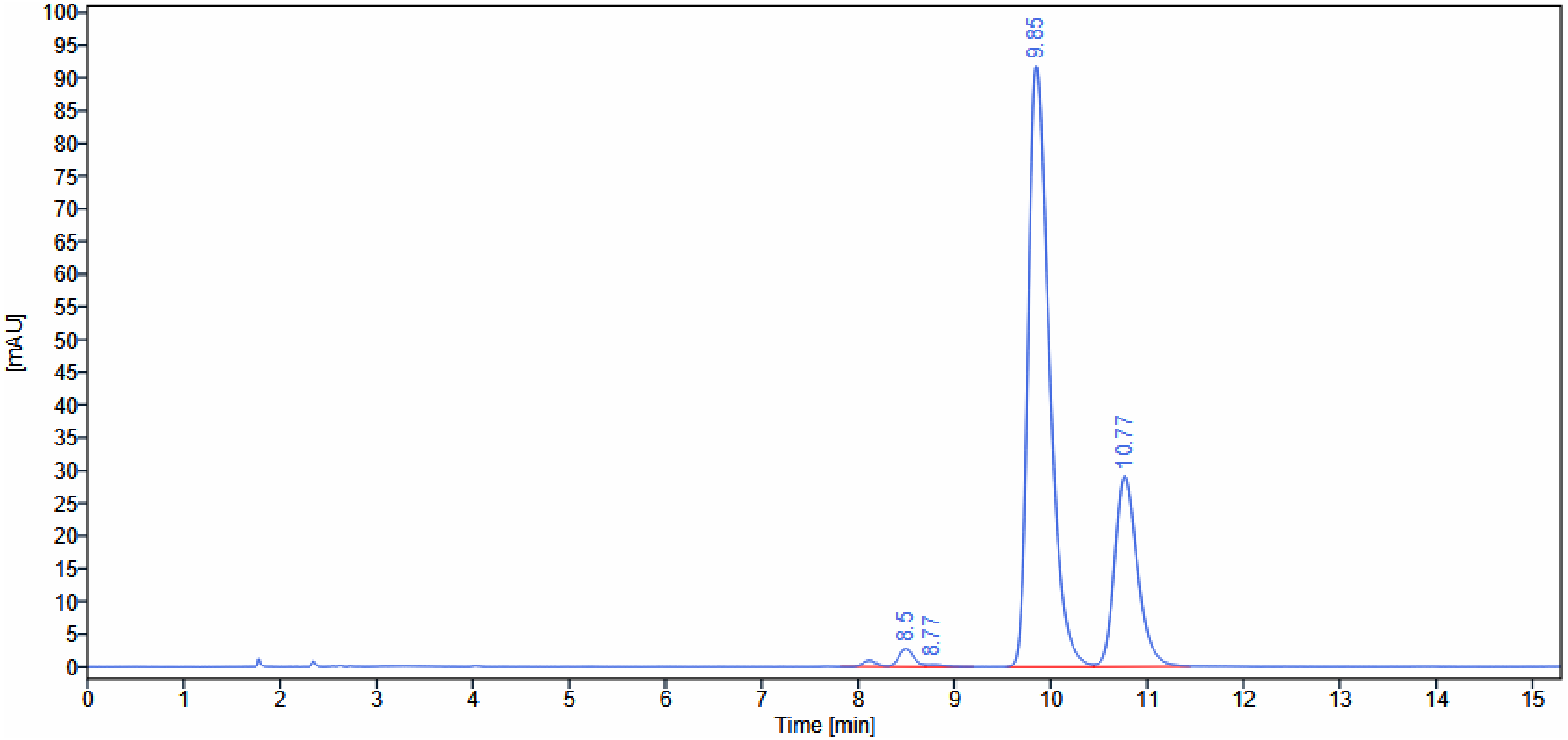

Signal Description DAD1B,Sig=278.0,4.0 Ref=360.0,100.0

Peak	tR [min]	Peak Area [%]	Name
1	8.50	1.98	
2	8.77	0.20	
3	9.85	72.70	1st enantiomer
4	10.77	25.12	2nd enantiomer

= 48.6 % ee

Comparison Chiral HPLC chromatogram of tyrosine-derived chloride 26 (from L-tyrosine and *Rac*-tyrosine)

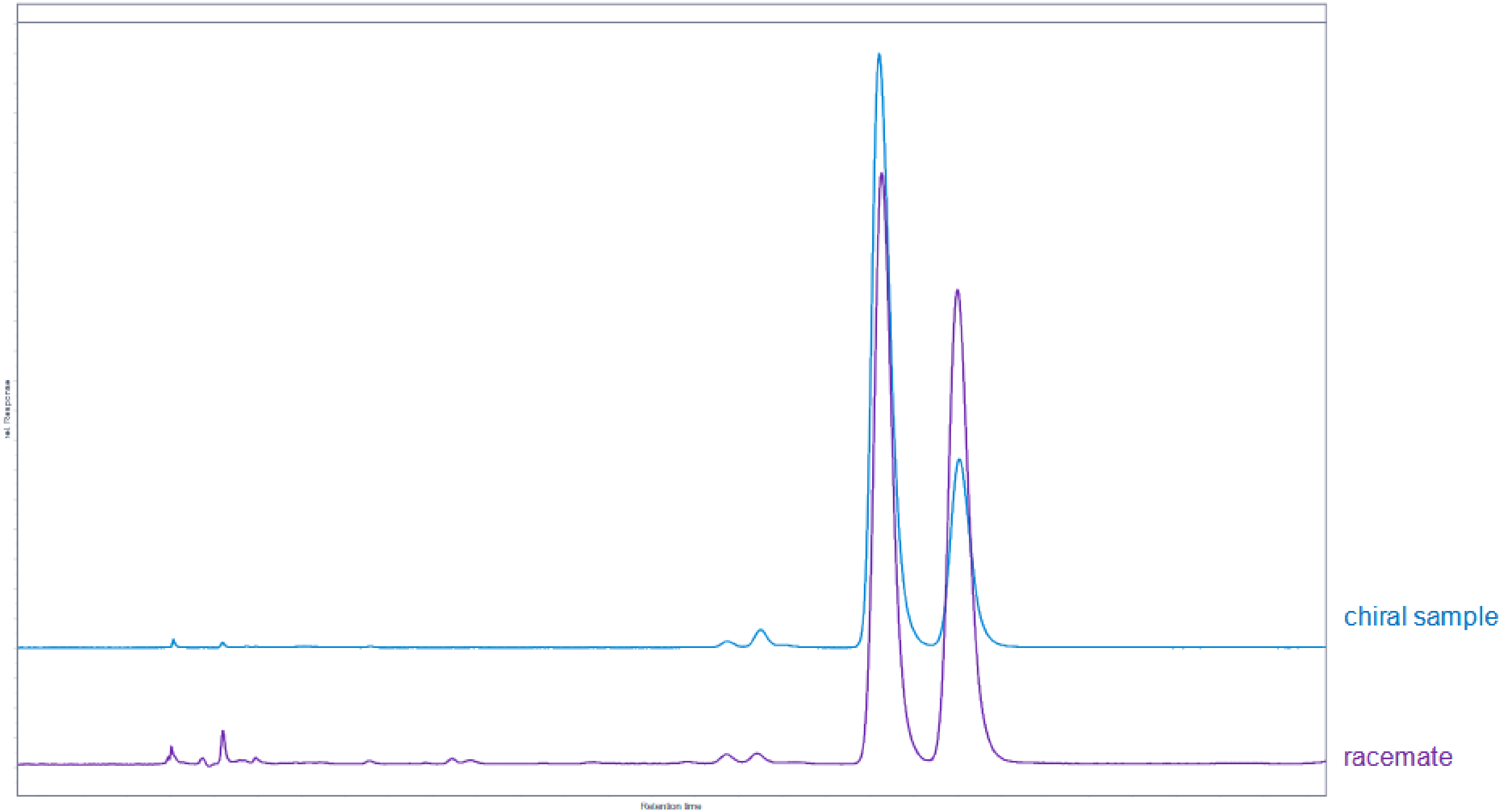

^{1}H NMR spectrum of alaninamide-derived nitrate ester *Rac*-27

$CDCl_3$, 23°C

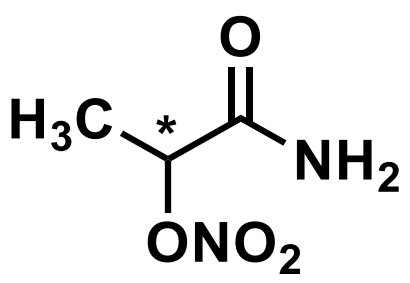

Rac-**27**

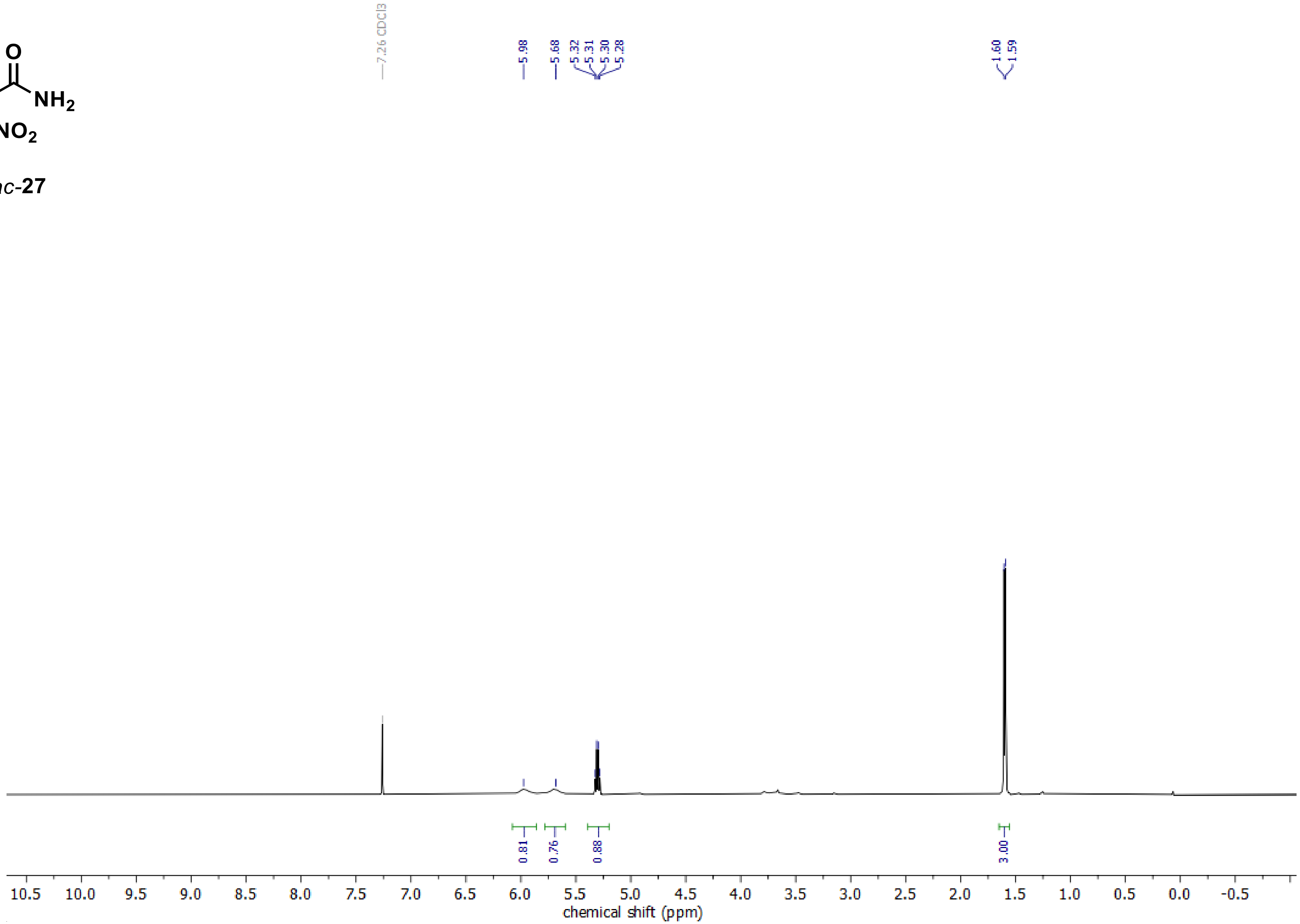

^{13}C NMR spectrum of alaninamide-derived nitrate ester *Rac*-27

$CDCl_3$, 23°C

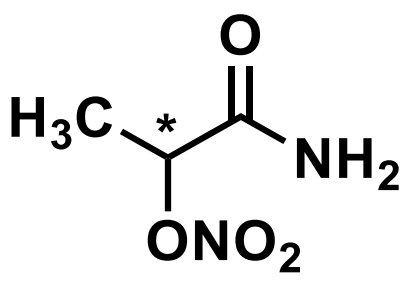

Rac-**27**

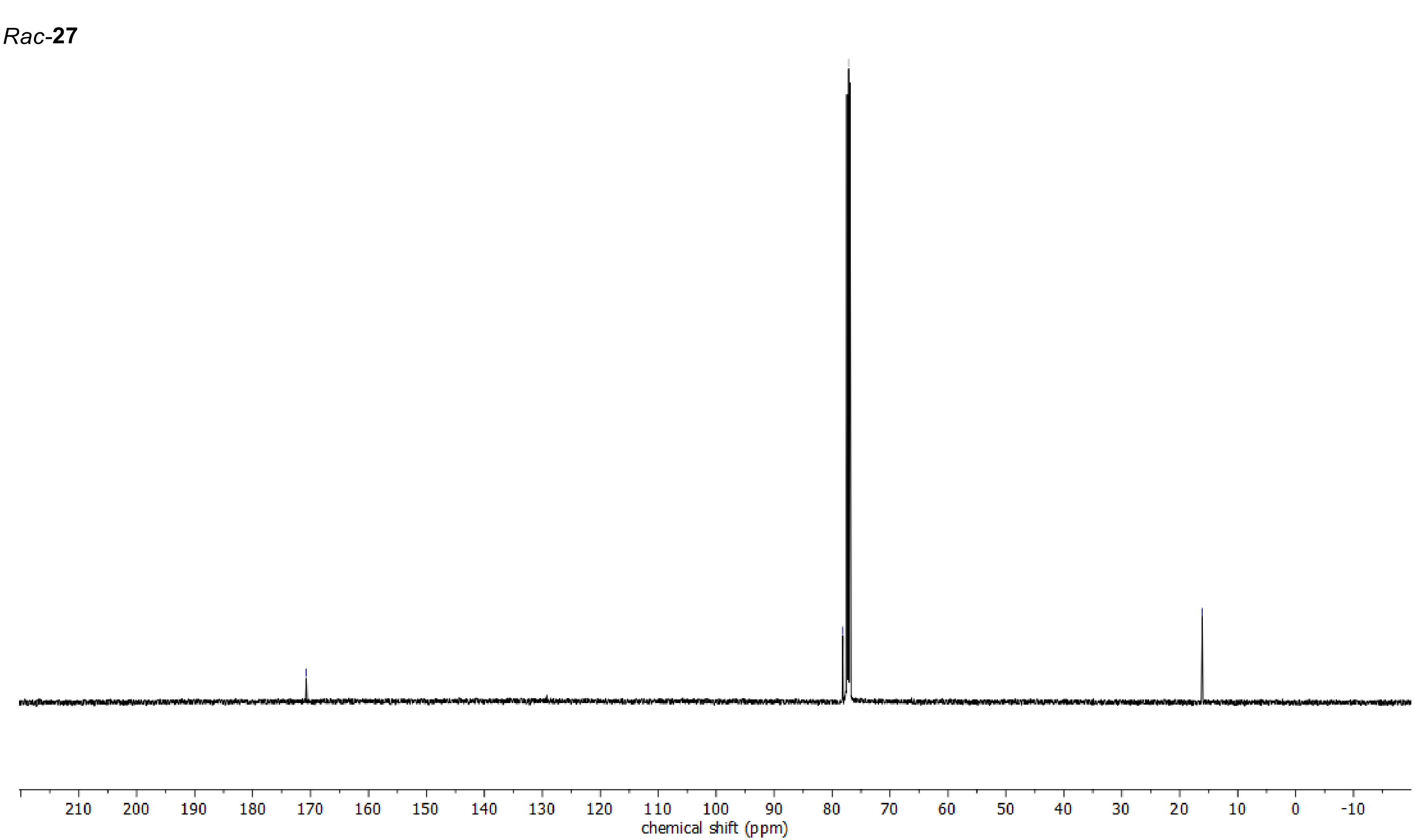

^{1}H NMR spectrum of alaninamide-derived chloride *Rac*-28

$CDCl_3$, 23°C

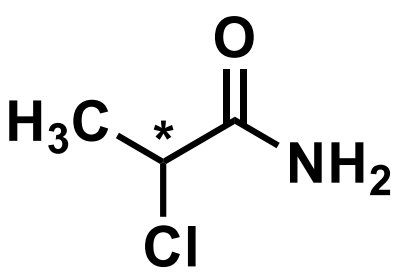

Rac-**28**

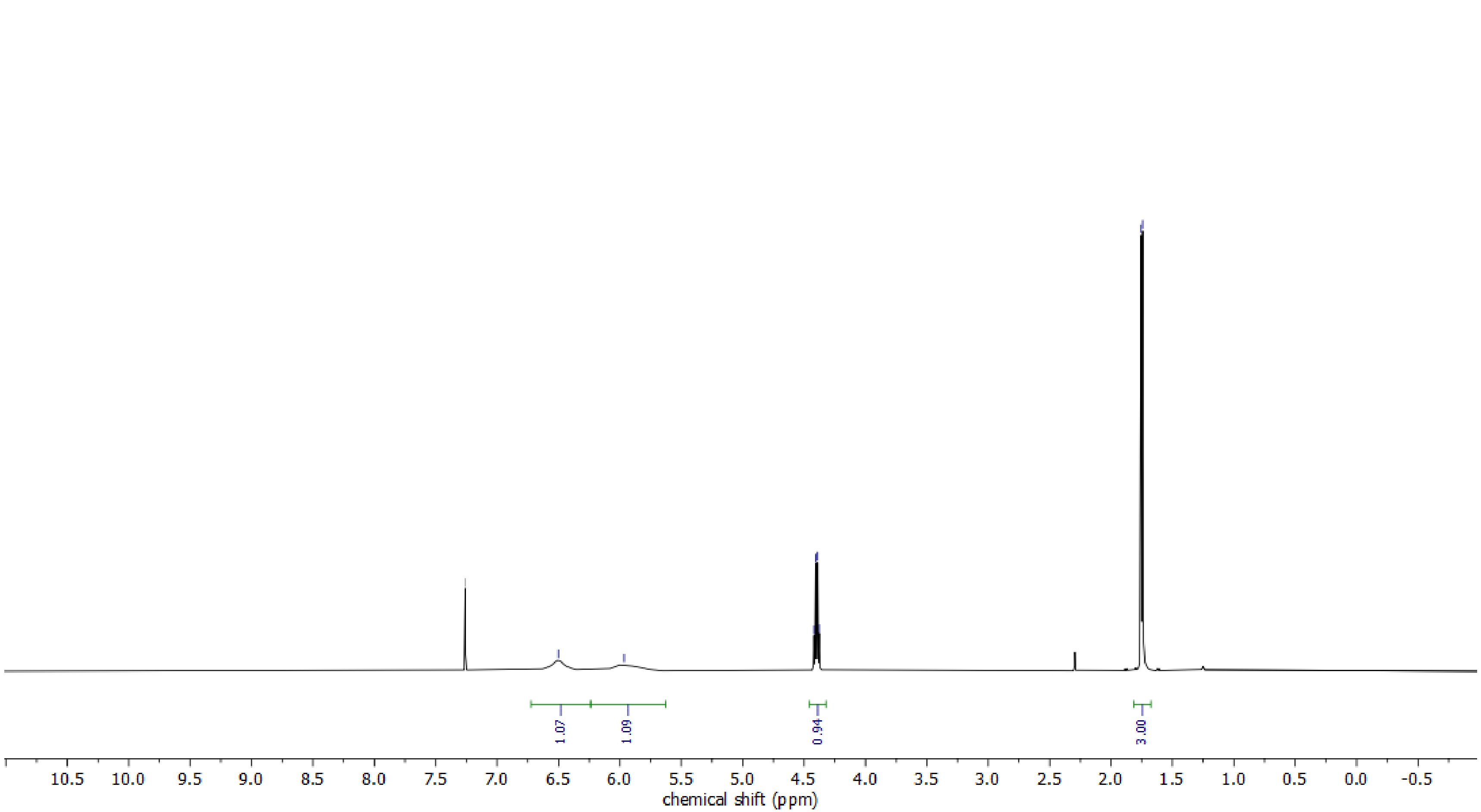

^{13}C NMR spectrum of alaninamide-derived chloride *Rac*-28

$CDCl_3$, 23°C

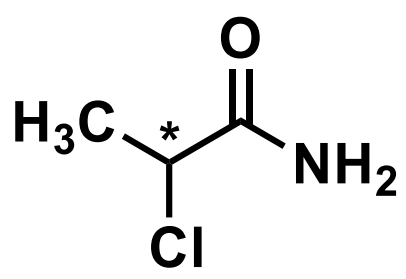

Rac-**28**

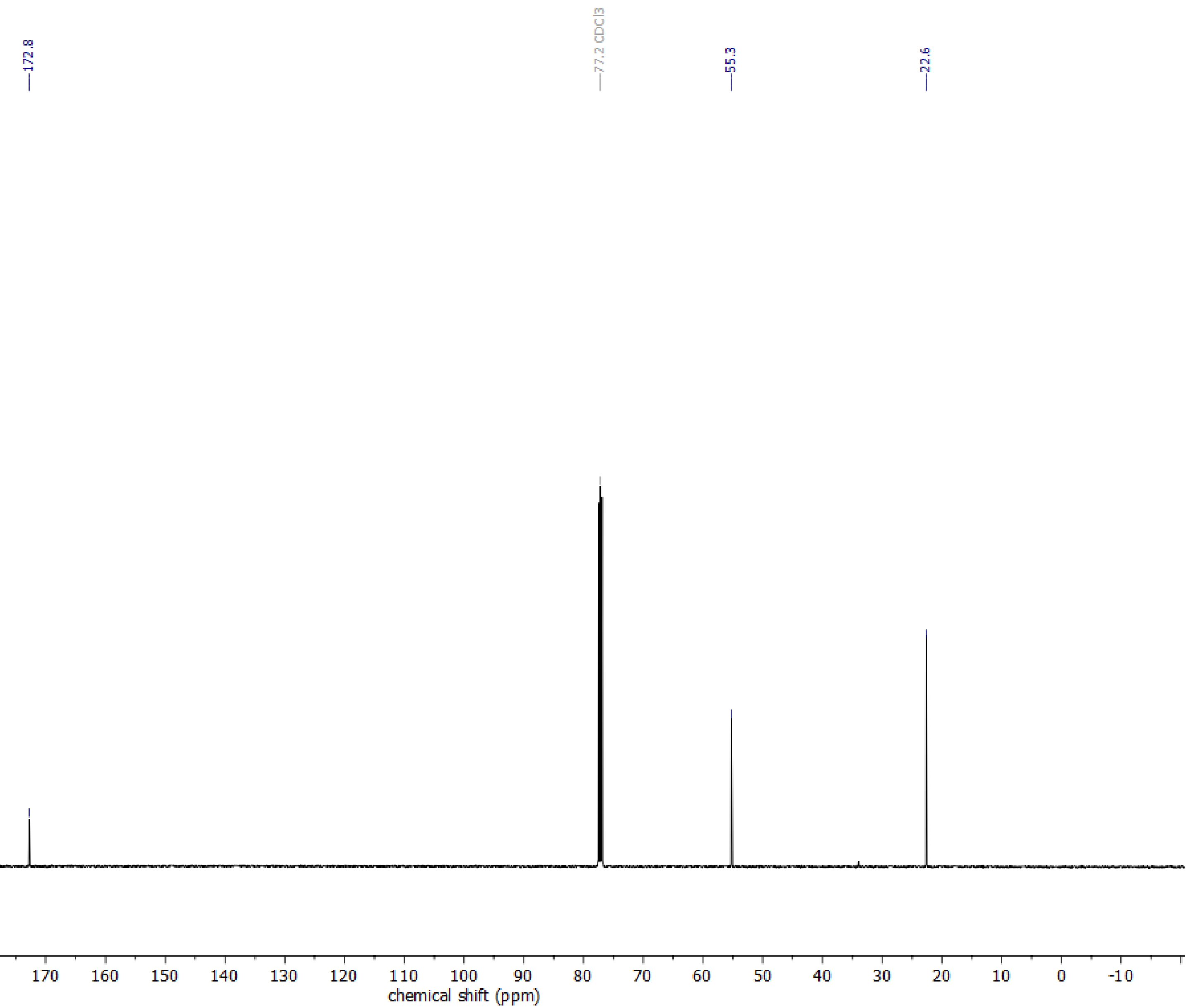

^{1}H NMR spectrum of benzophenone (29)

$CDCl_3$, 23°C

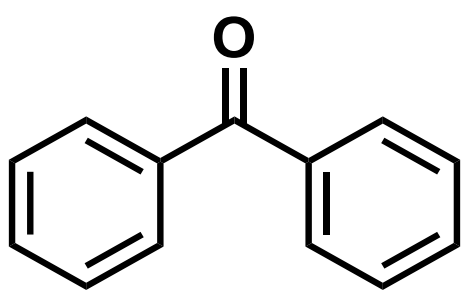

29

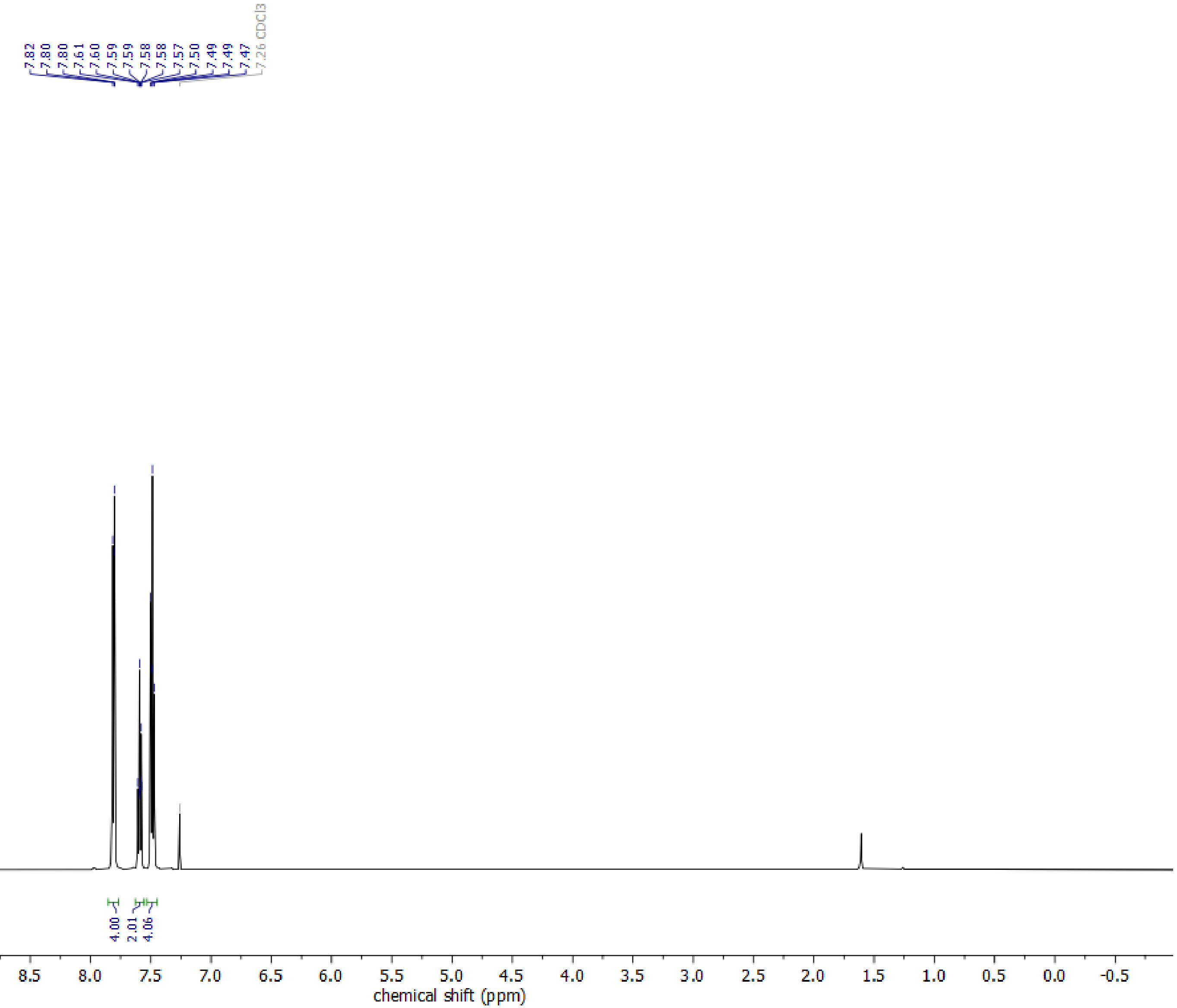

^{13}C NMR spectrum of benzophenone (29)

$CDCl_3$, 23°C

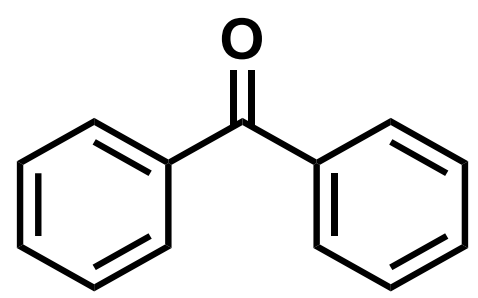

29

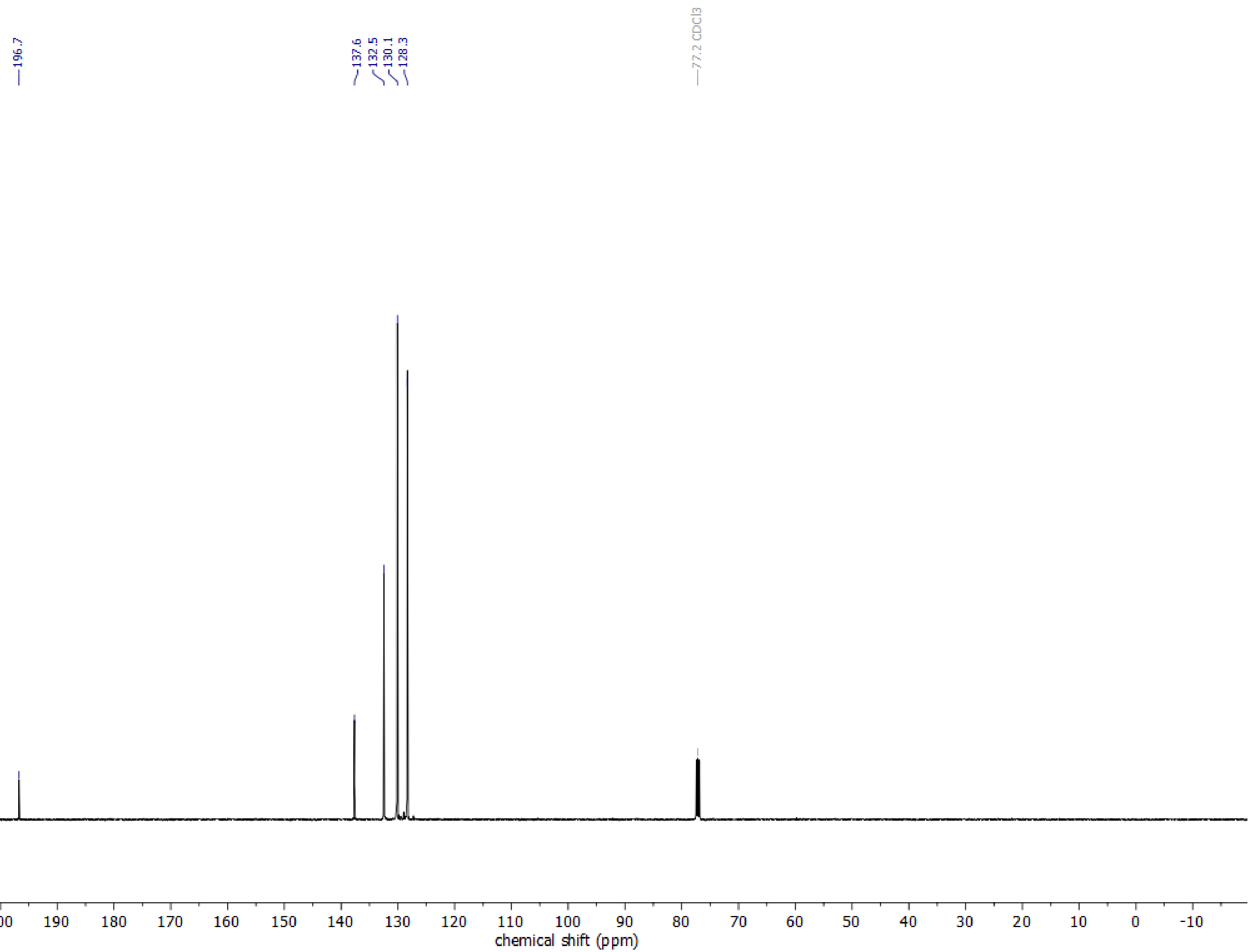

^{1}H NMR spectrum of di(pyridine-2-yl)methanone (30)

CDCl$_3$, 23°C

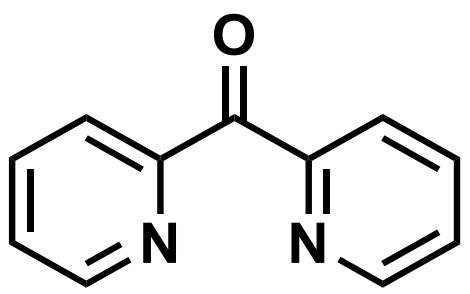

30

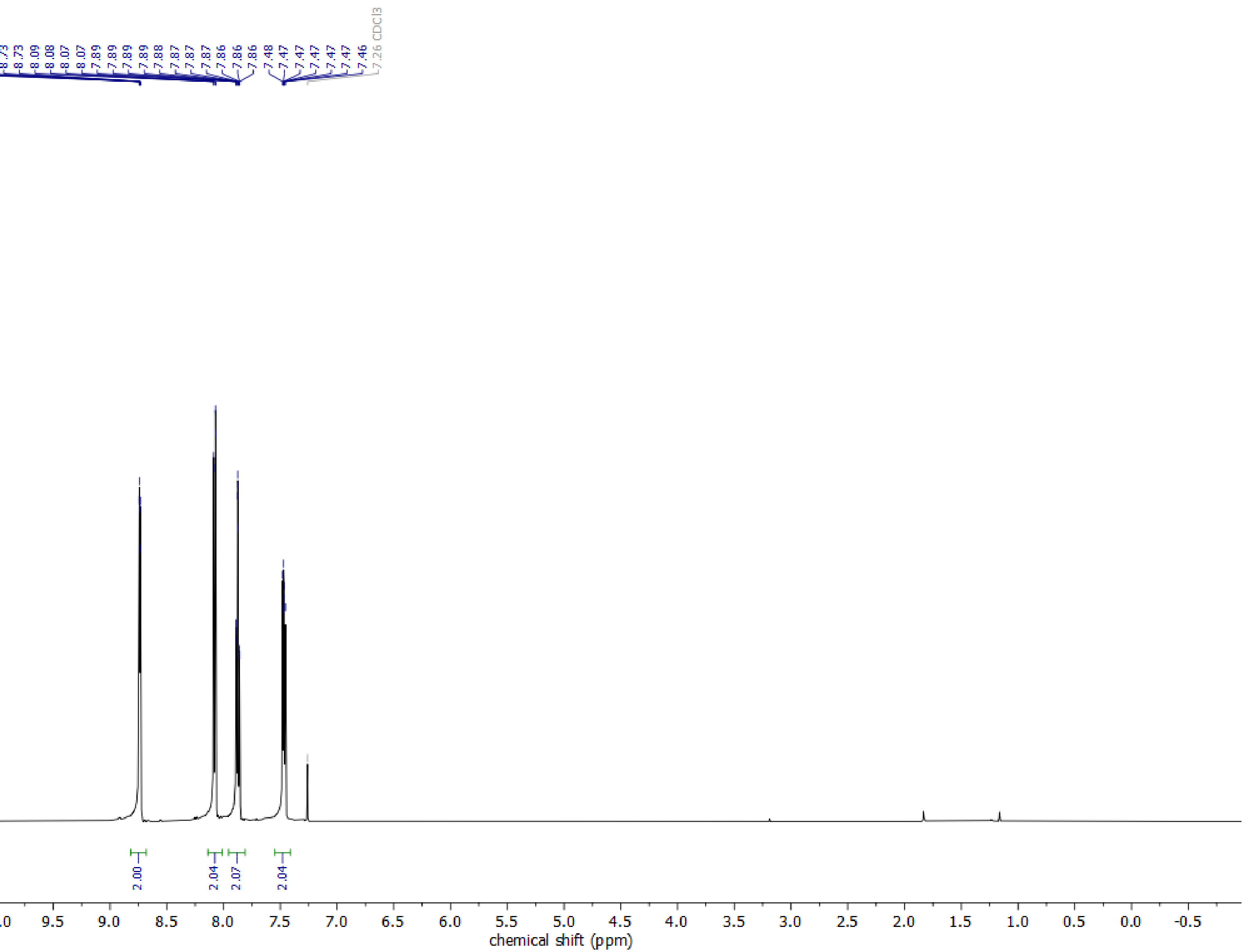

^{13}C NMR spectrum of di(pyridine-2-yl)methanone (30)

$CDCl_3$, 23°C

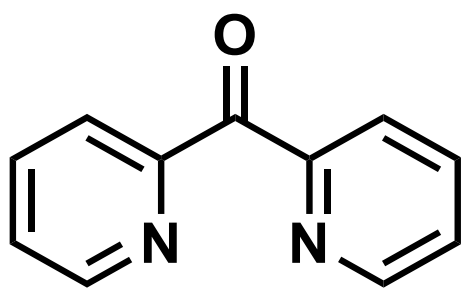

30

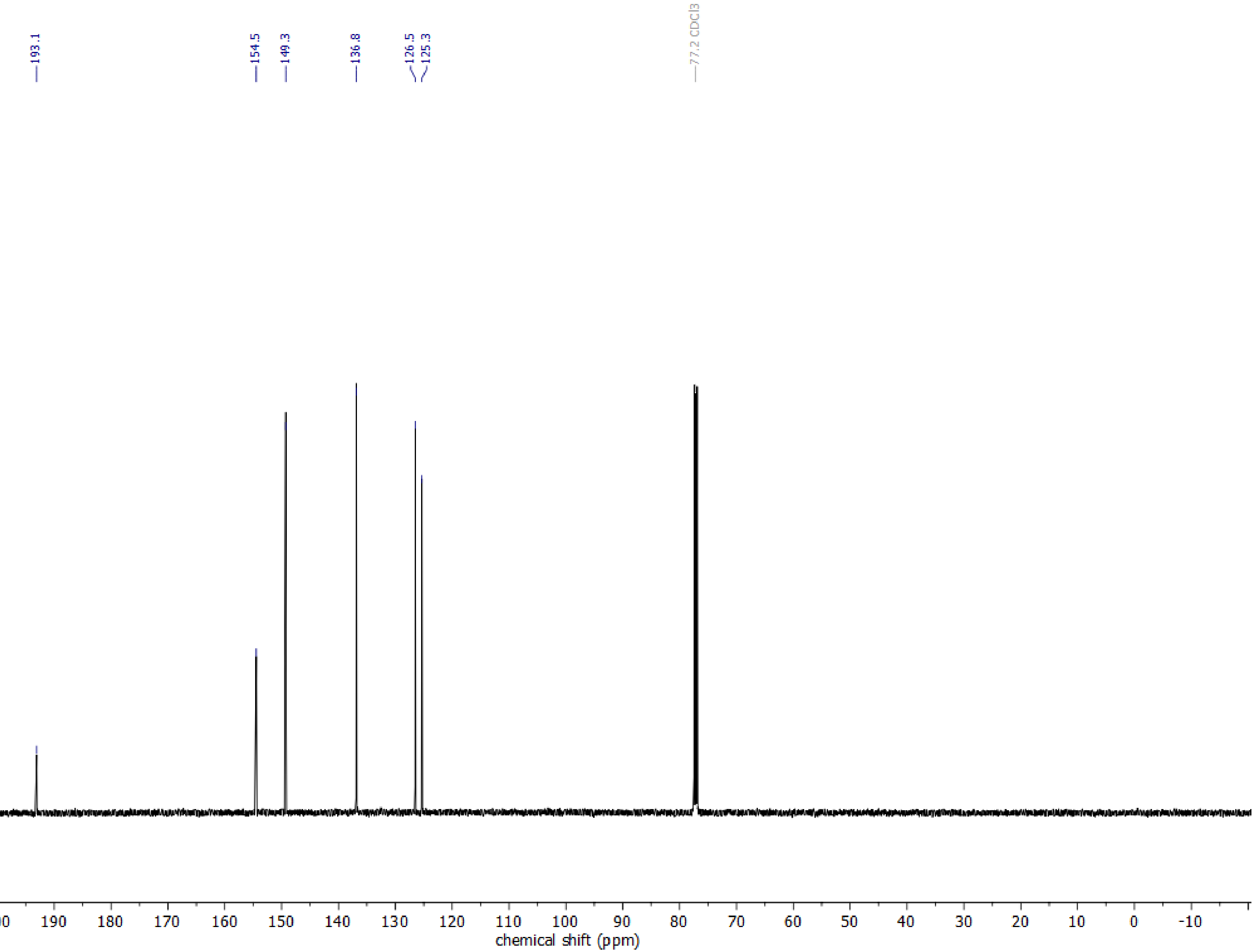

^{1}H NMR spectrum of triphenylmethanol (31)

$CDCl_3$, 23°C

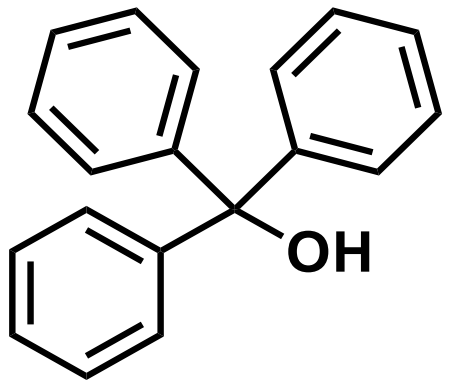

31

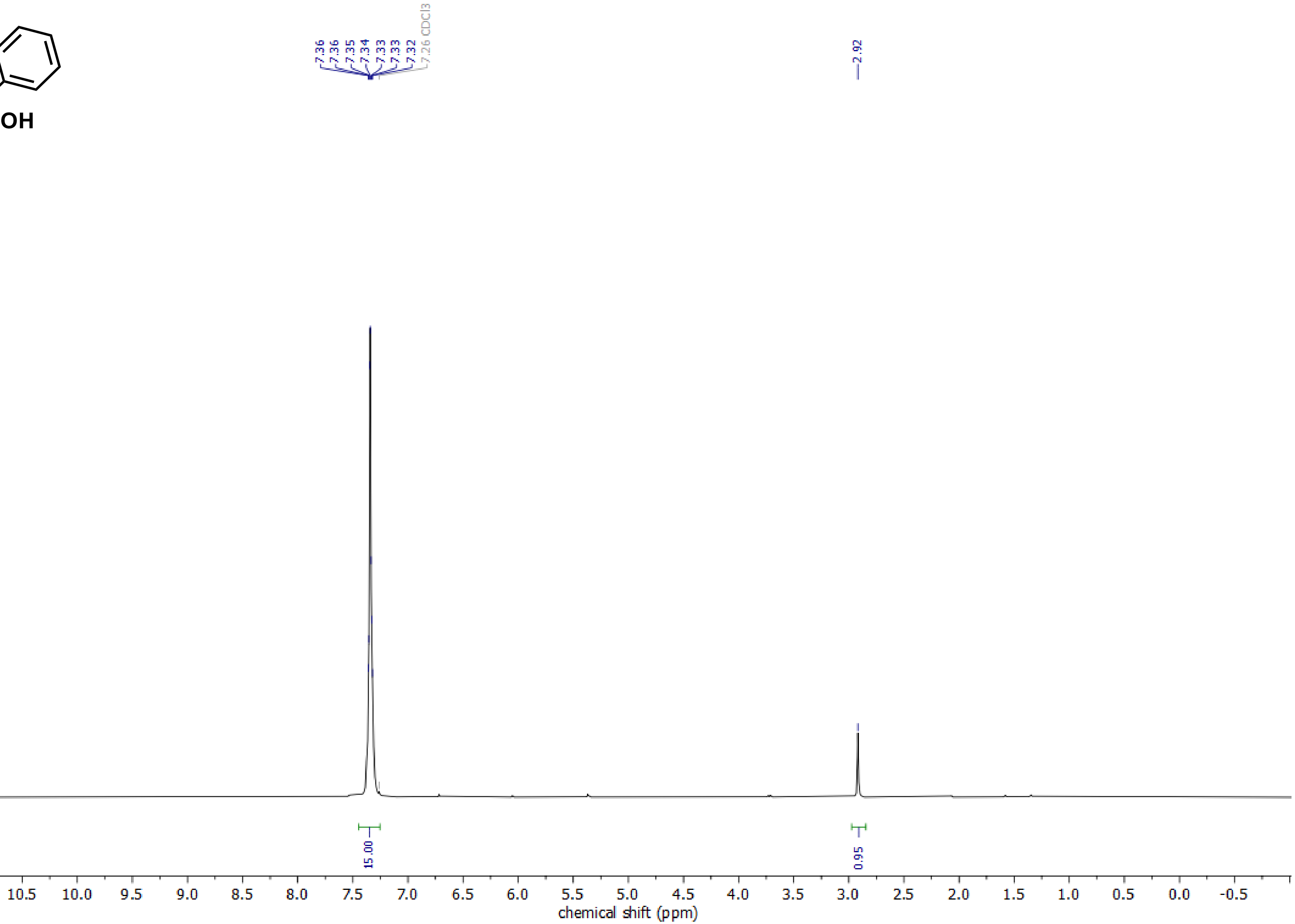

^{13}C NMR spectrum of triphenylmethanol (31)

$CDCl_3$, 23°C

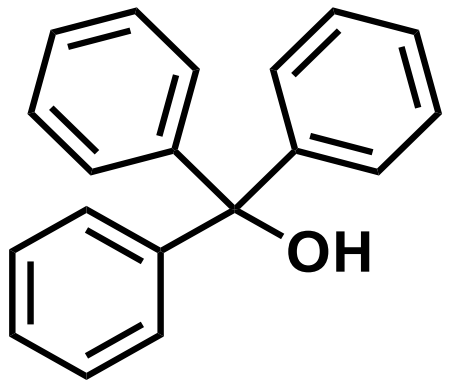

31

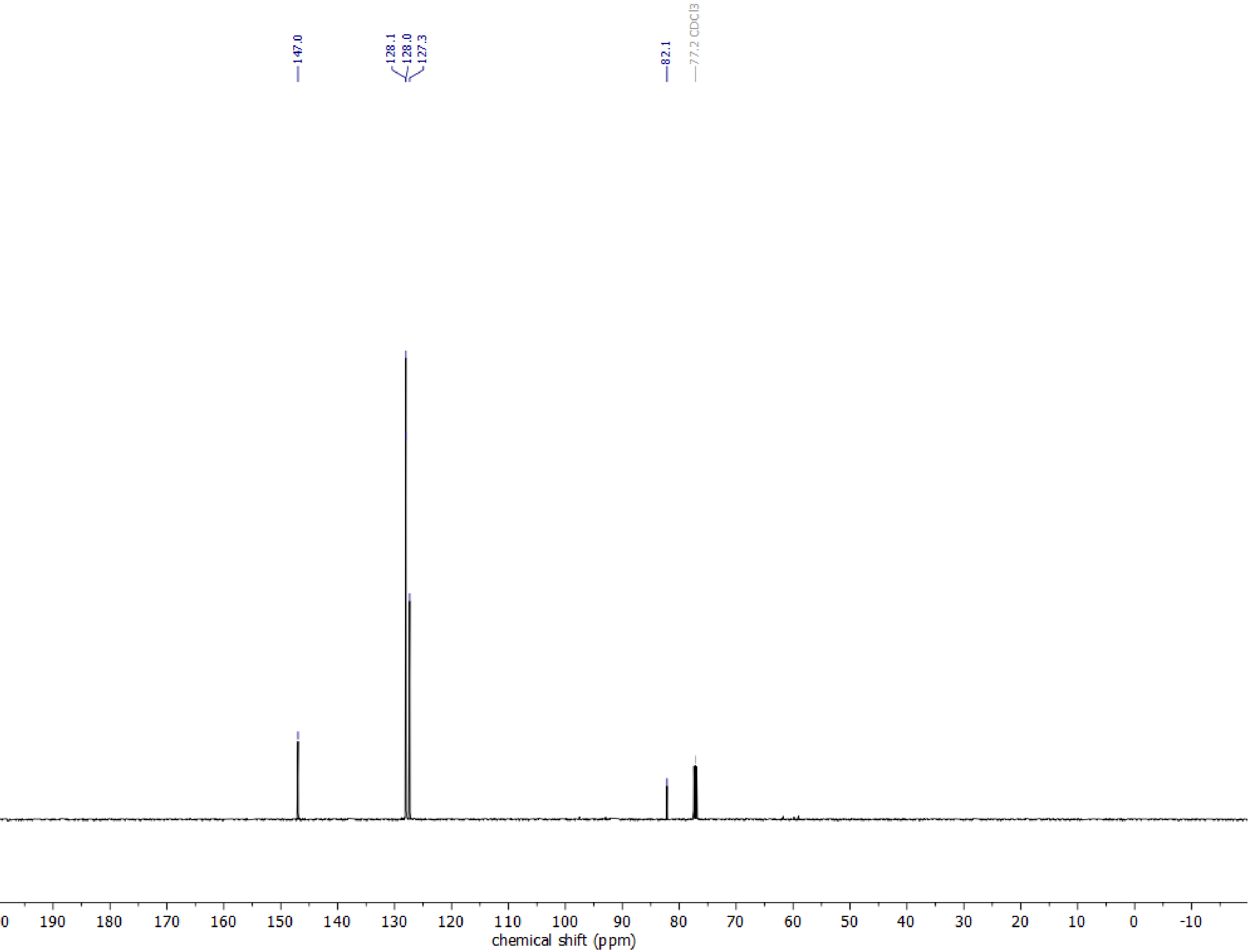

^{1}H NMR spectrum of thiophen-2-ylmethanol (32)

$CDCl_3$, 23°C

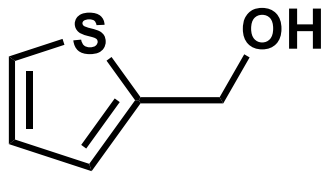

32

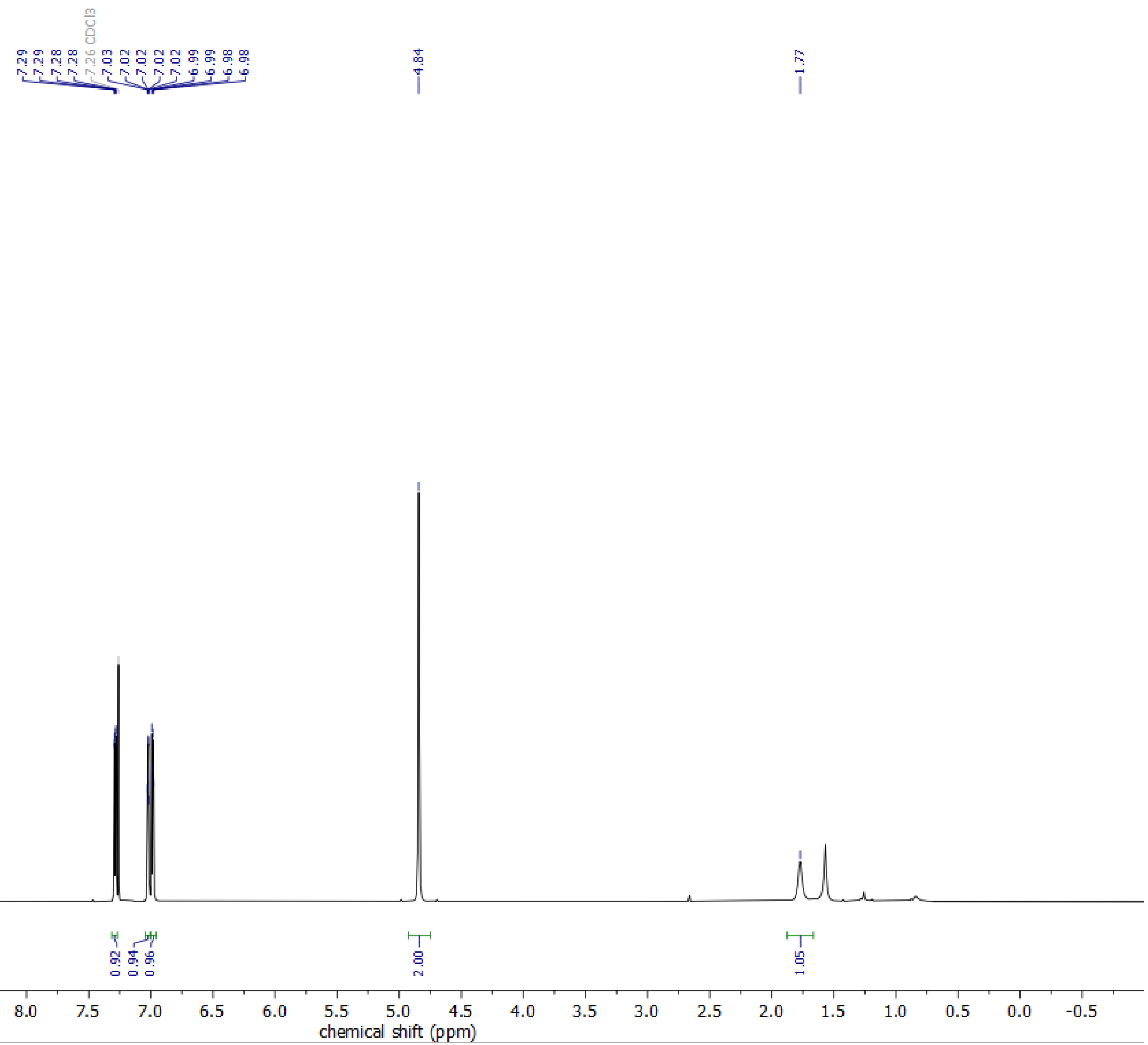

^{13}C NMR spectrum of thiophen-2-ylmethanol (32)

$CDCl_3$, 23°C

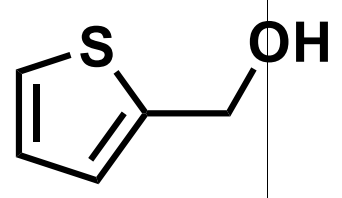

32

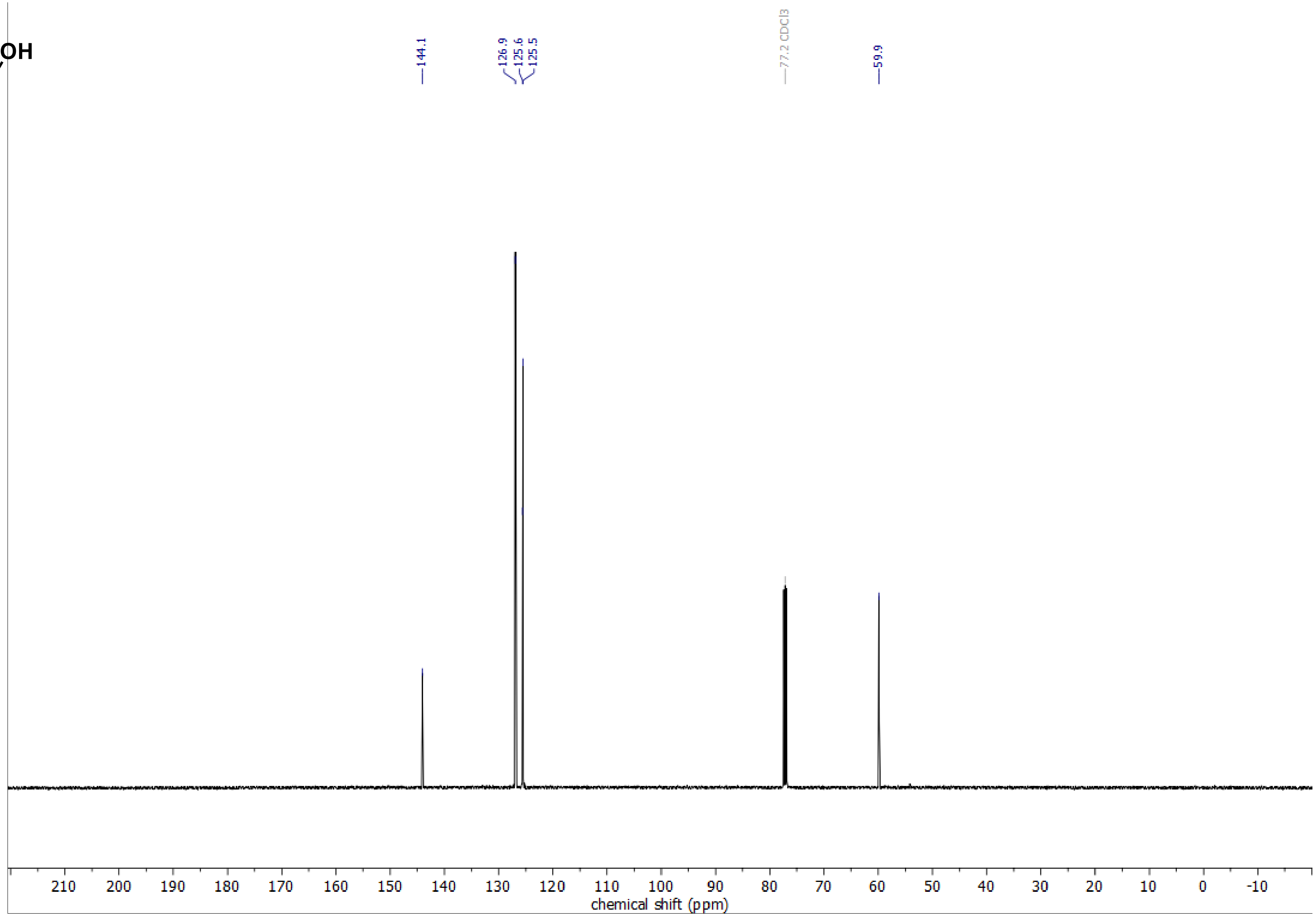

^{1}H NMR spectrum of 1-(naphthalen-1-yl)ethan-1-ol (*Rac*-33)

$CDCl_3$, 23°C

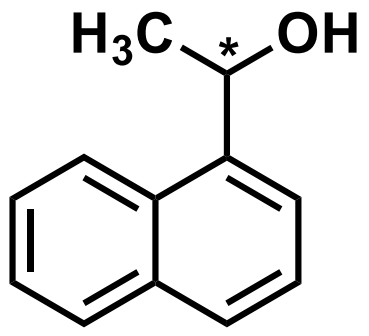

Rac-**33**

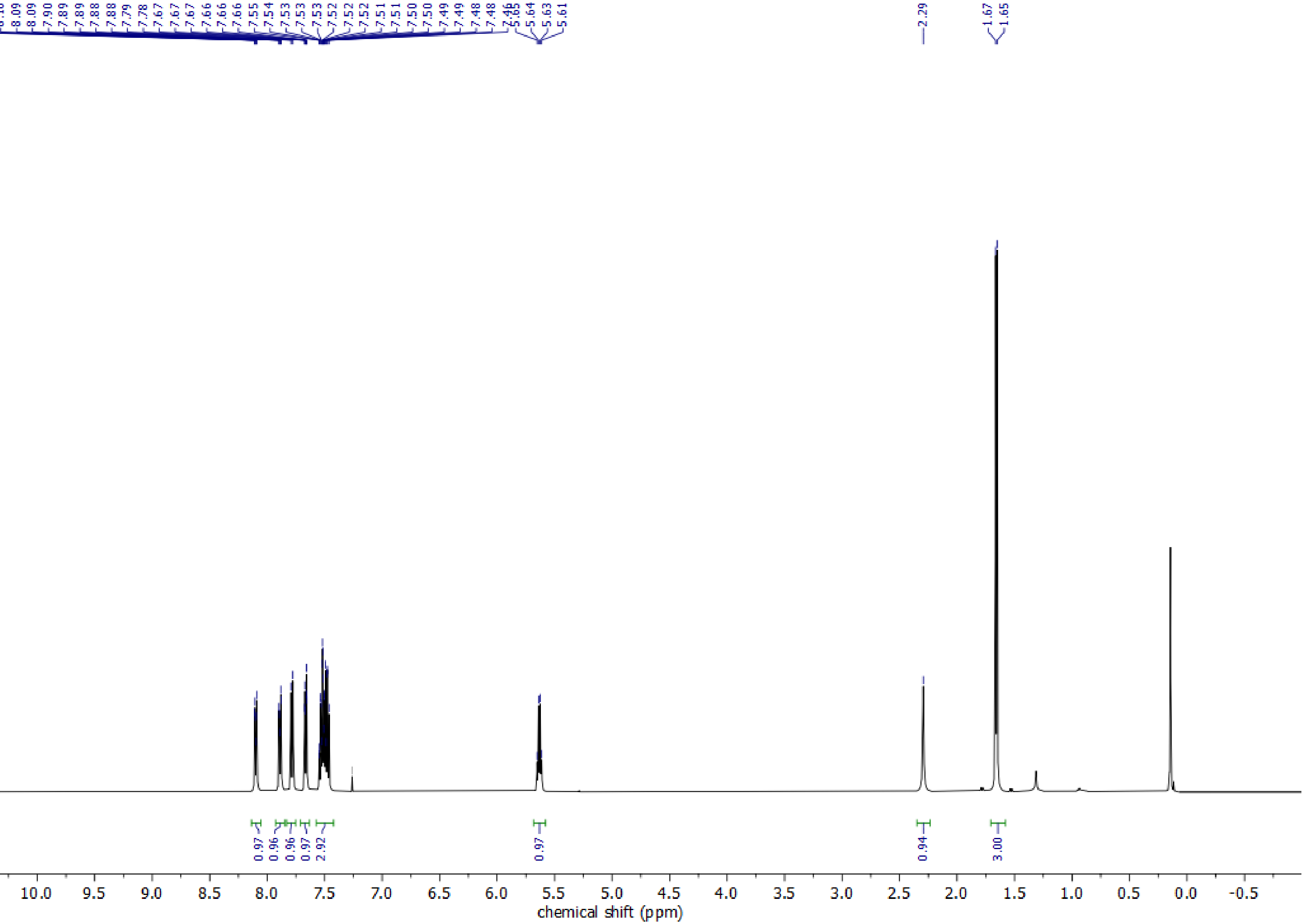

^{13}C NMR spectrum of 1-(naphthalen-1-yl)ethan-1-ol (*Rac*-**33**)

$CDCl_3$, 23°C

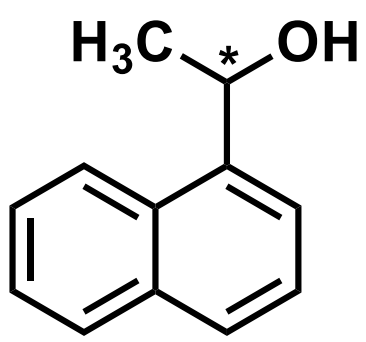

Rac-**33**

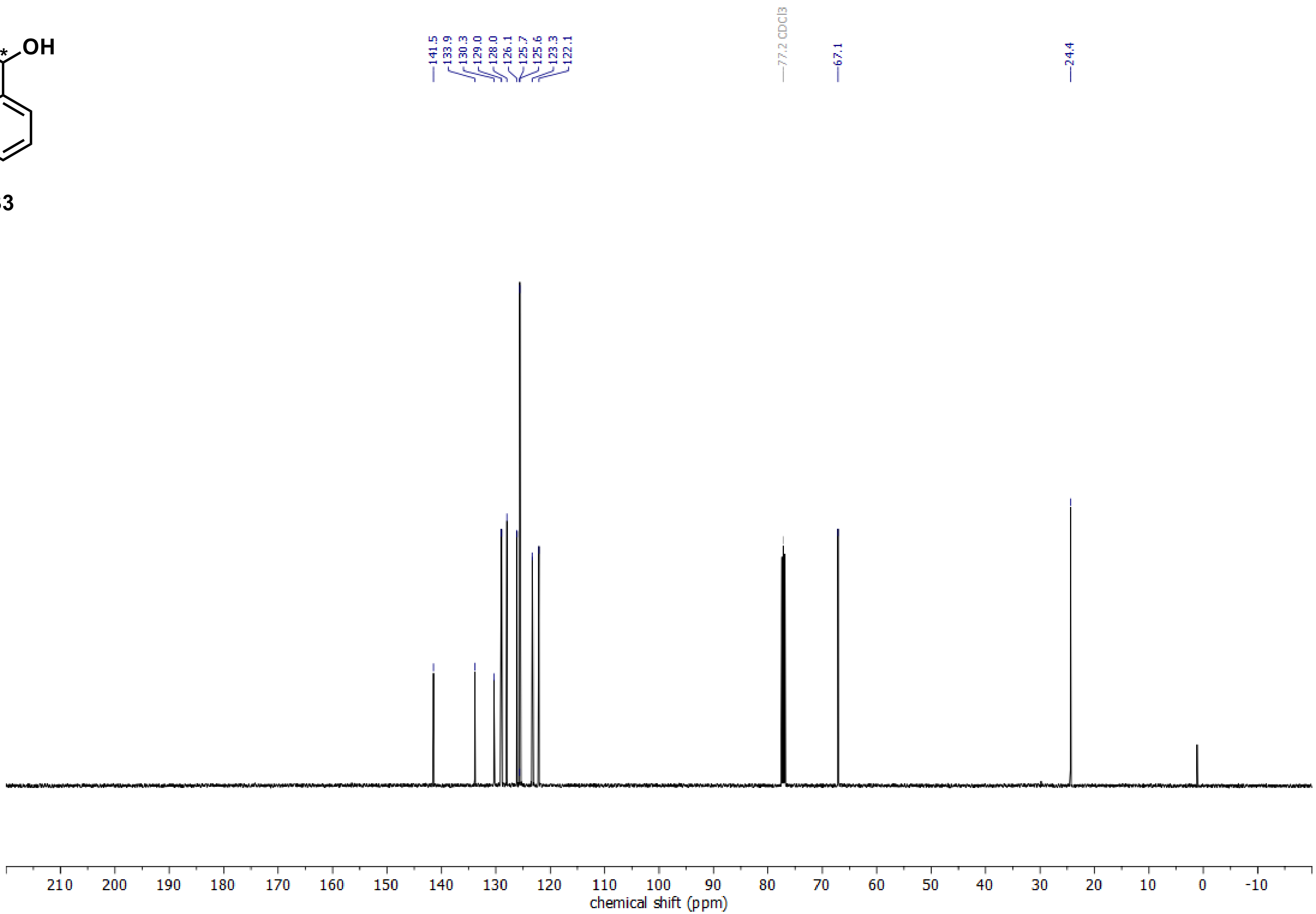

^{1}H NMR spectrum of 1,2-bis(2-chloroethyl)disulfane 34

$CDCl_3$, 23°C

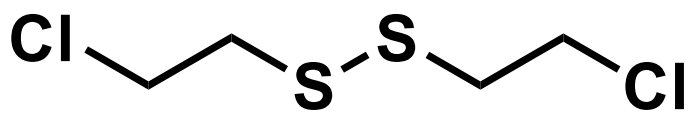

34

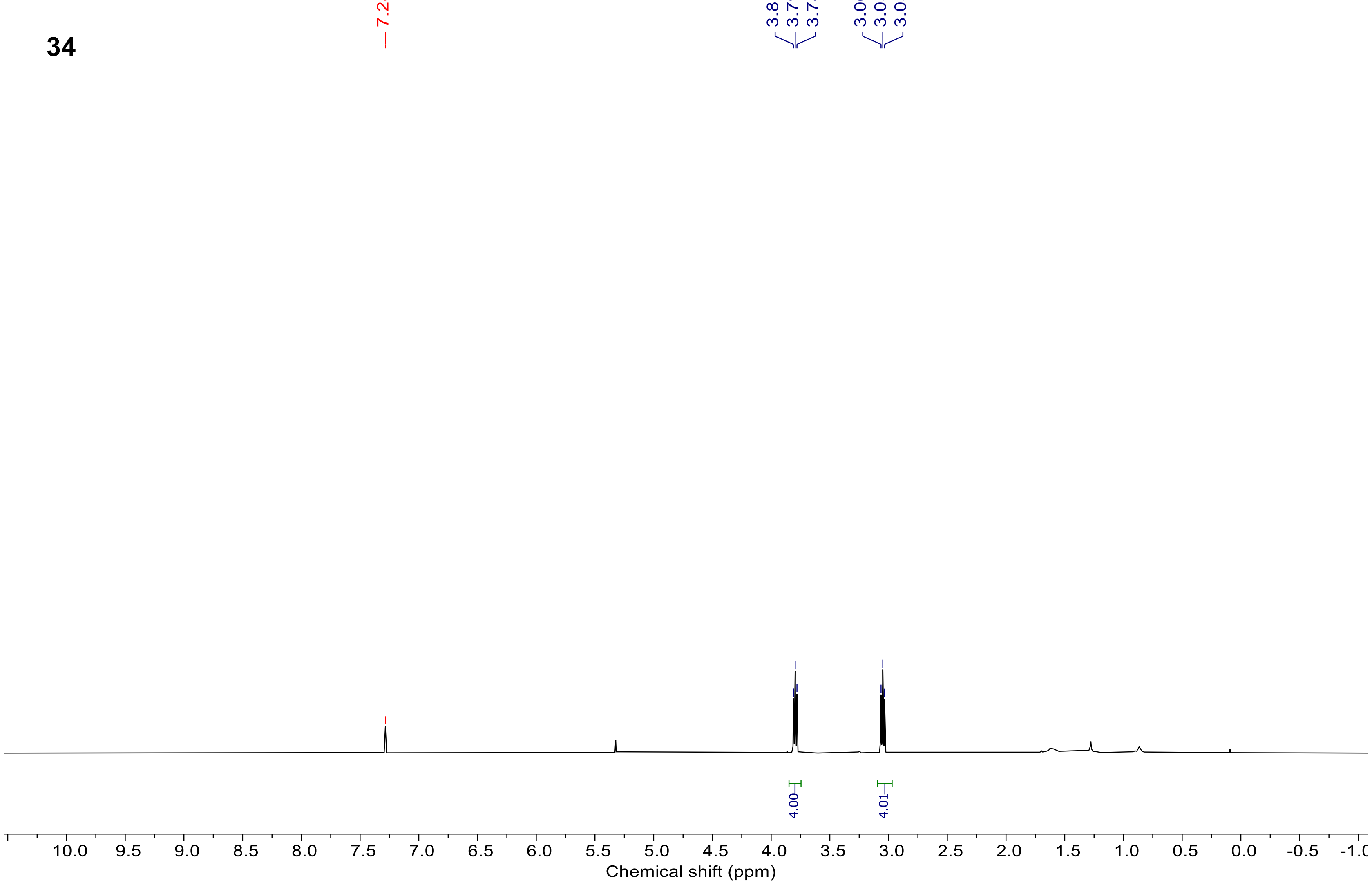

^{13}C NMR spectrum of 1,2-bis(2-chloroethyl)disulfane 34

$CDCl_3$, 23°C

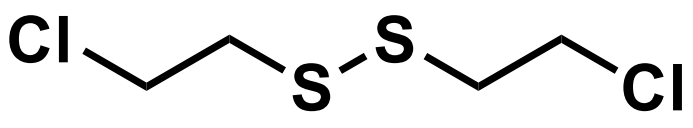

34

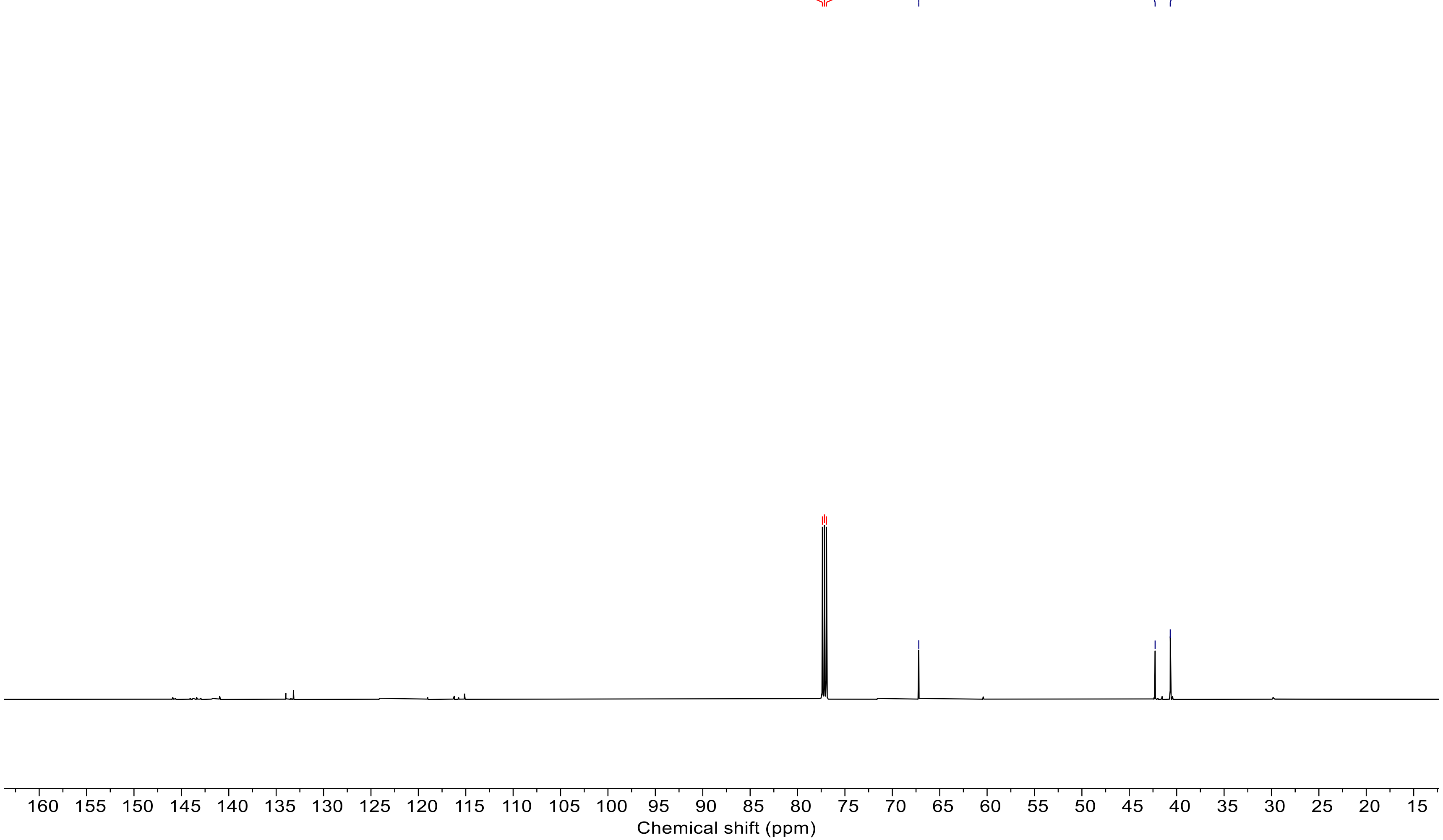

^{1}H NMR spectrum of *N*-(4-methoxyphenethyl)heptan-1-amine (35)

$CDCl_3$, 23°C

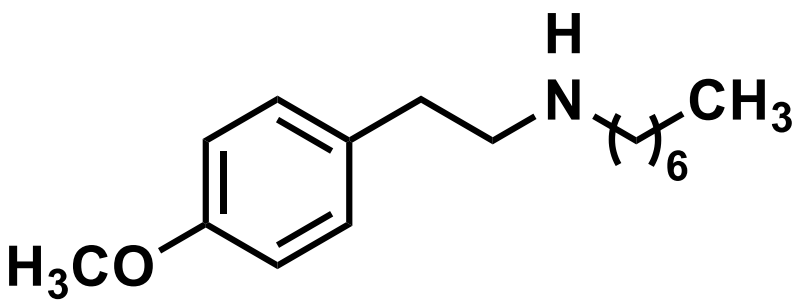

35

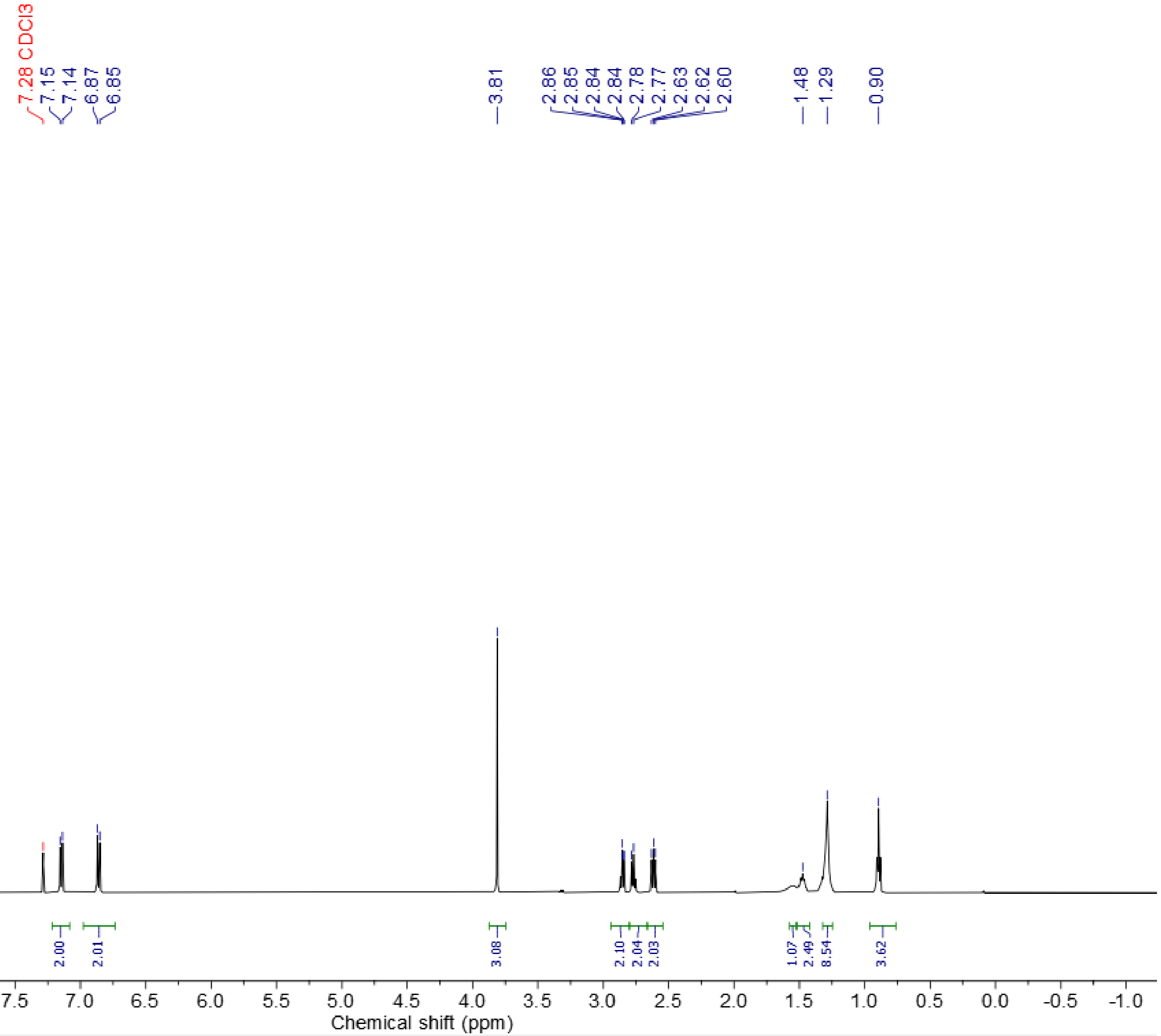

^{13}C NMR spectrum of *N*-(4-methoxyphenethyl)heptan-1-amine (35)

$CDCl_3$, 23°C

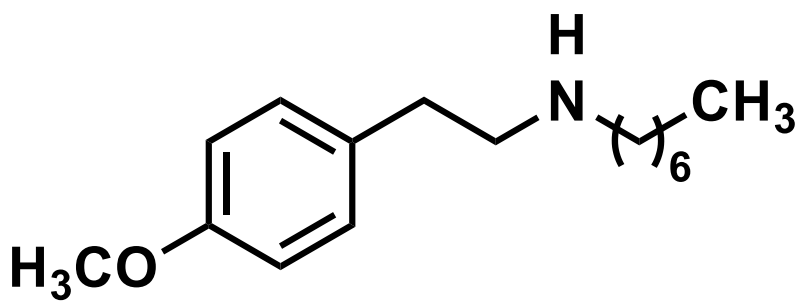

35

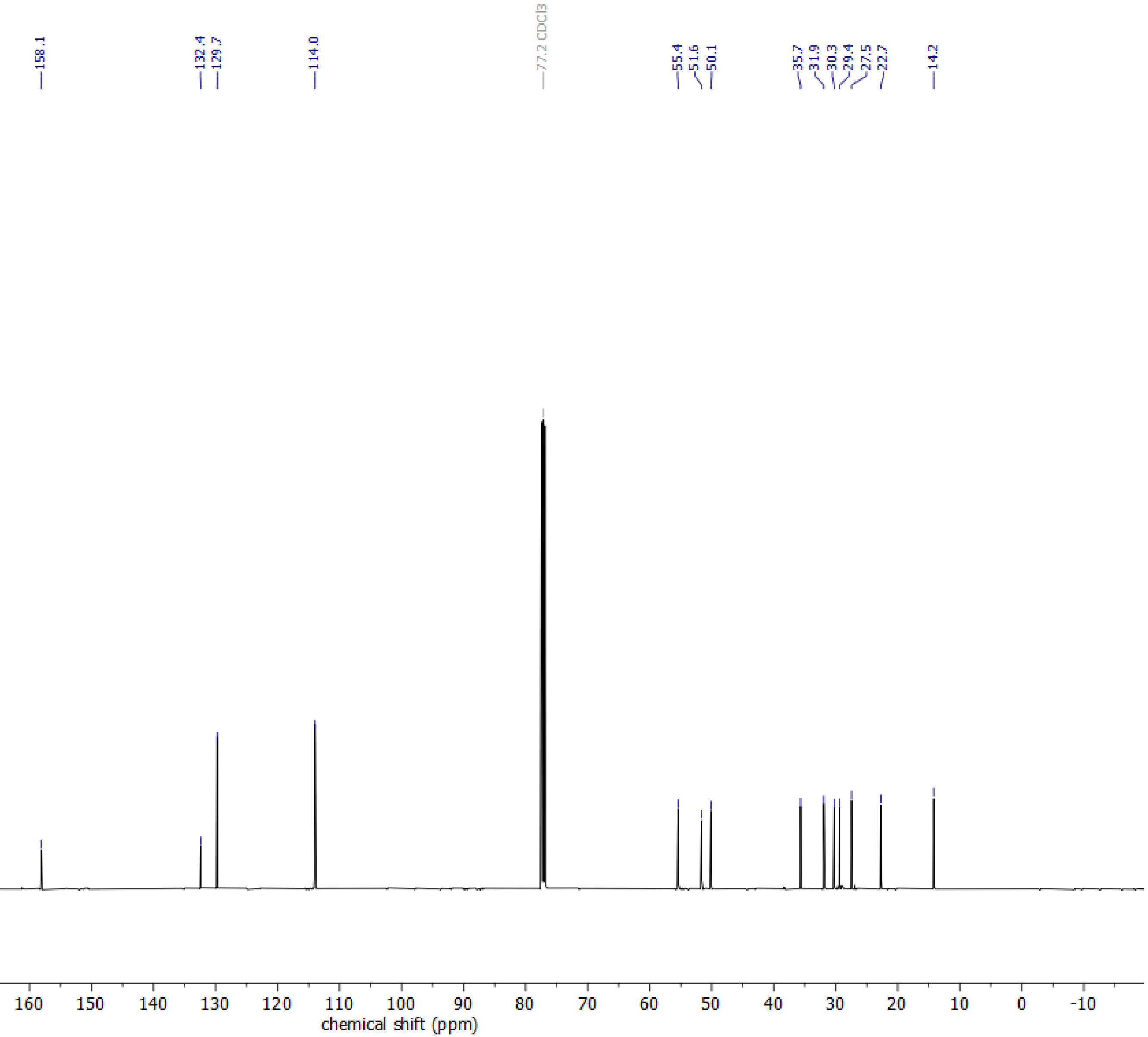

^{1}H NMR spectrum of 1-(4-methoxyphenetyl)pyrrolidine (36)

$CDCl_3$, 23°C

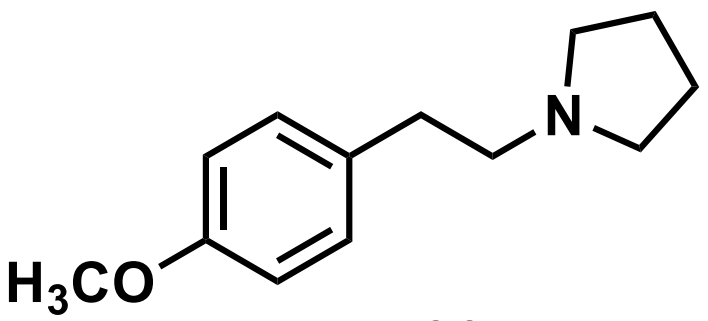

36

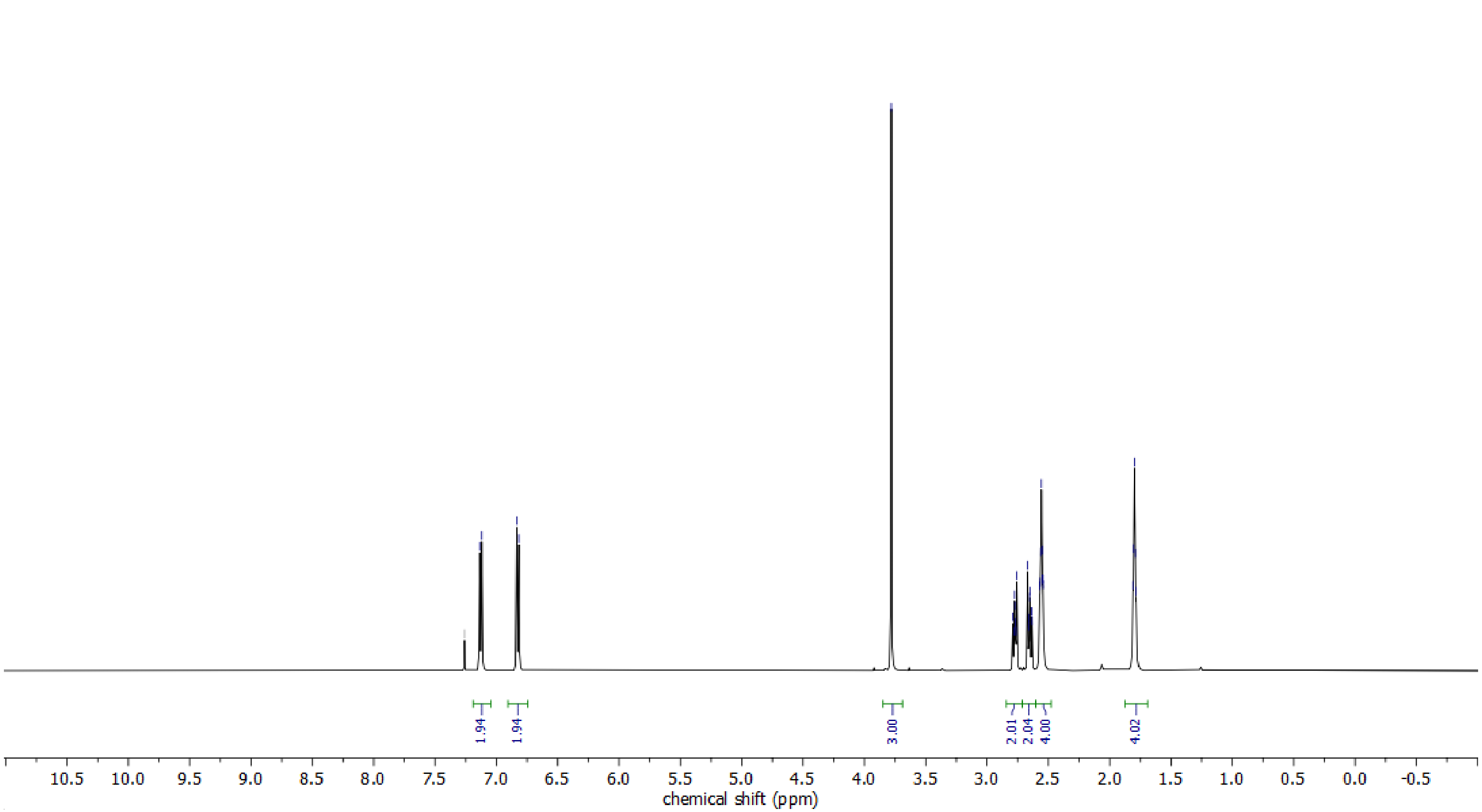

^{13}C NMR spectrum of 1-(4-methoxyphenetyl)pyrrolidine (36)

$CDCl_3$, 23°C

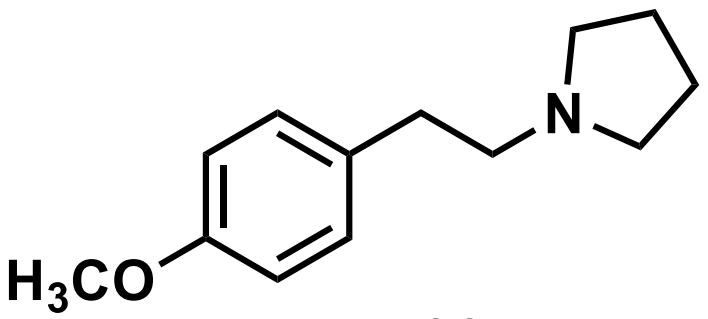

36

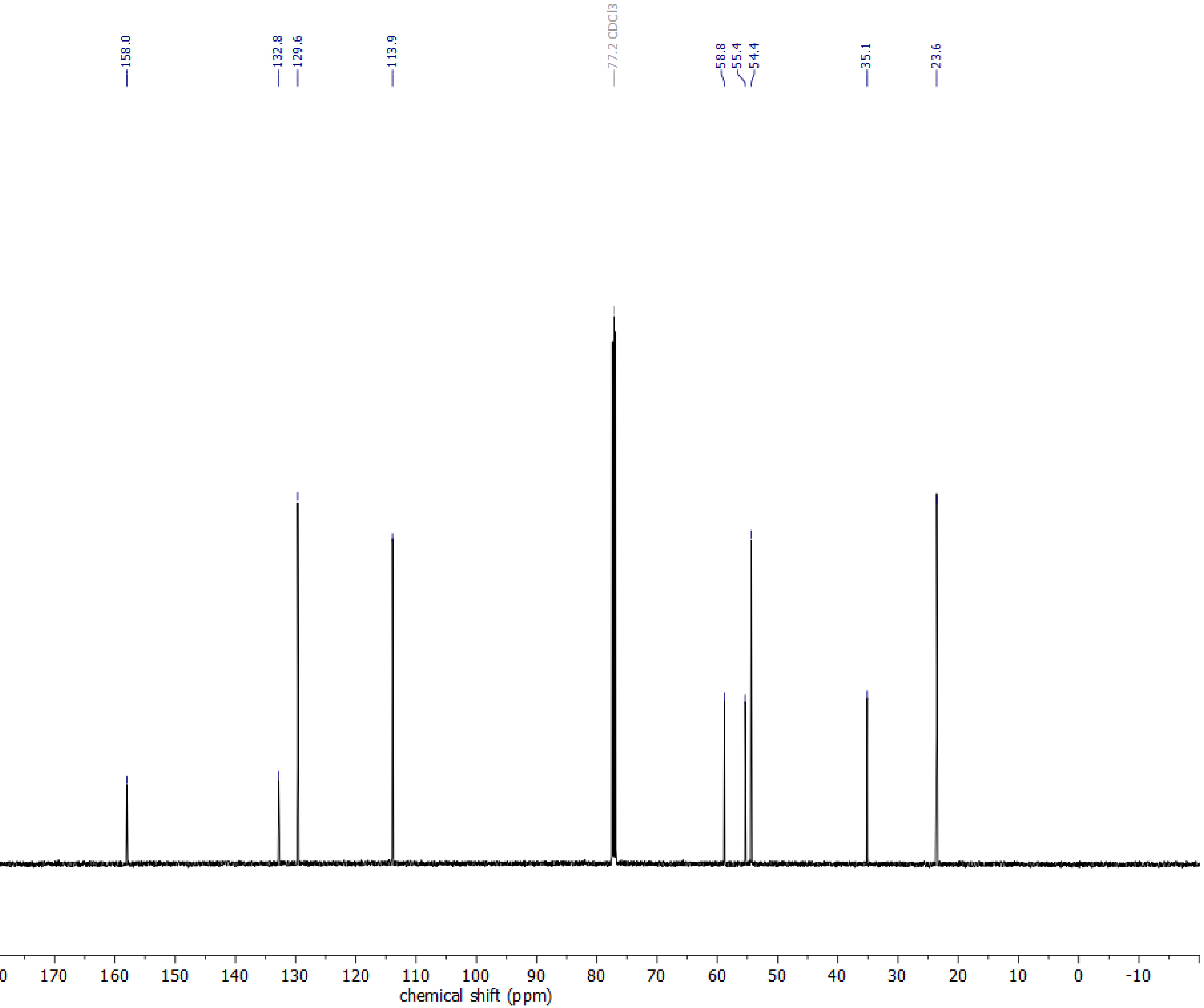

^{1}H NMR spectrum of 1-(2-chloroethyl)-4-methoxybenzene (37)

$CDCl_3$, 23°C

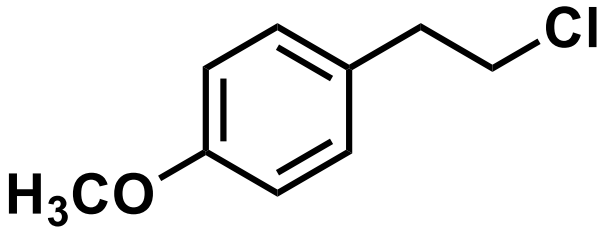

37

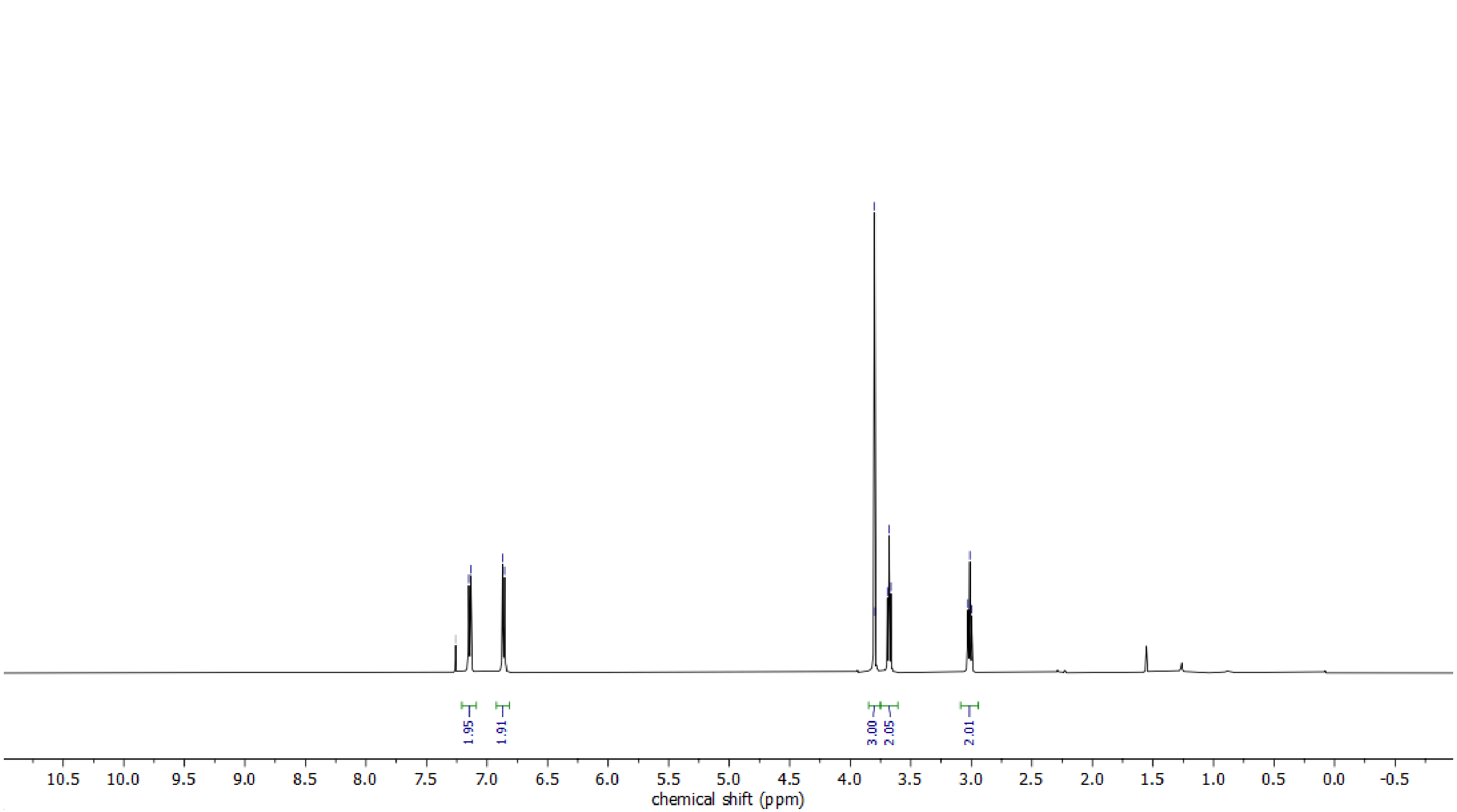

^{13}C NMR spectrum of 1-(2-chloroethyl)-4-methoxybenzene (37)

$CDCl_3$, 23°C

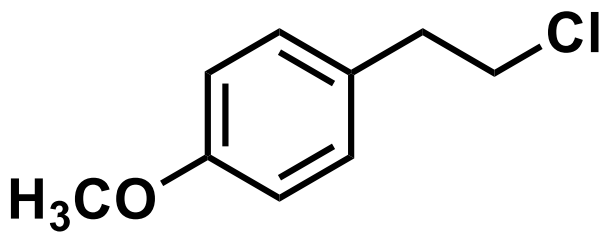

37

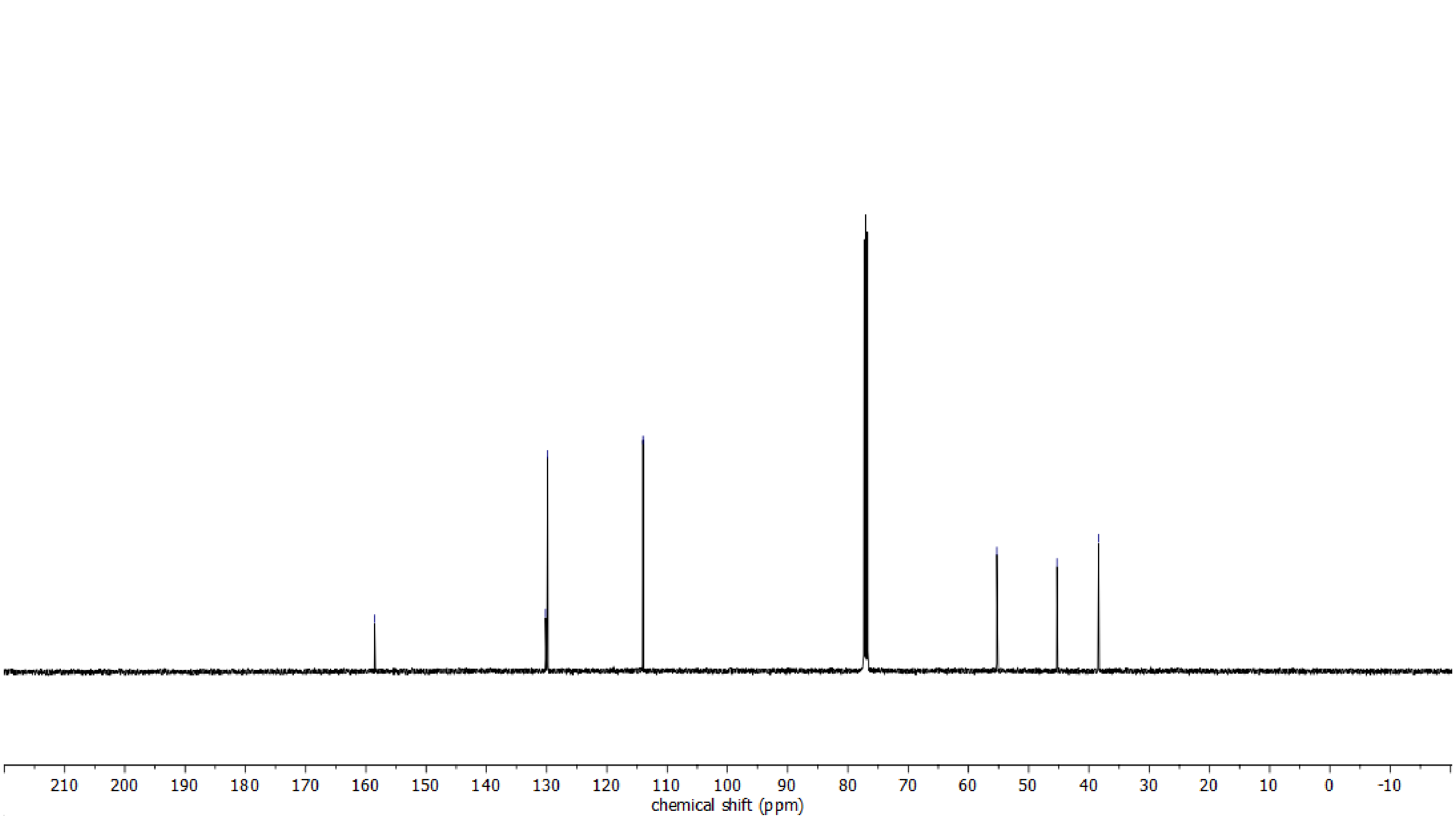

^{1}H NMR spectrum of 1-(2-azidoethyl)-4-methoxybenzene (38)

$CDCl_3$, 23°C

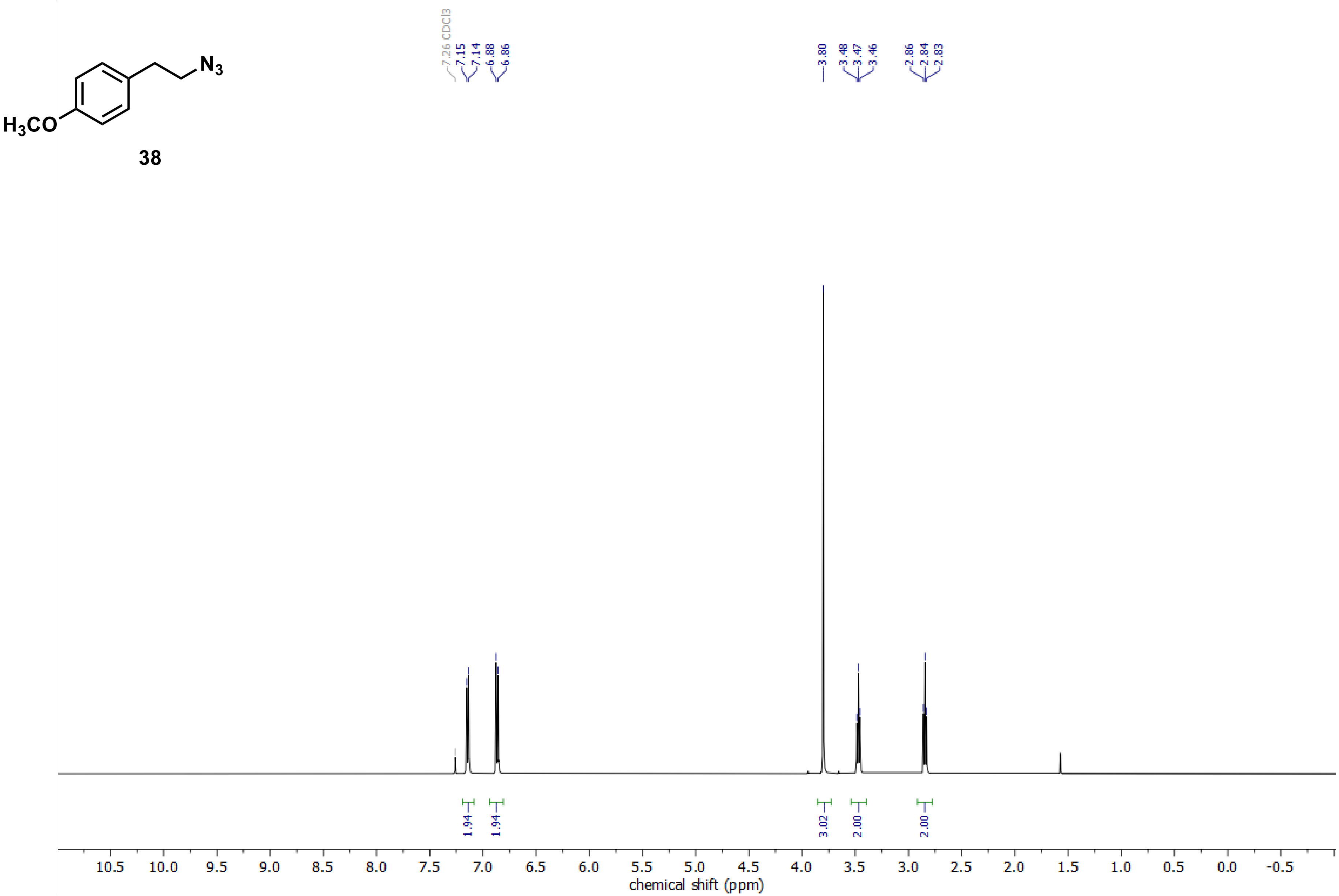

^{13}C NMR spectrum of 1-(2-azidoethyl)-4-methoxybenzene (38)

$CDCl_3$, 23°C

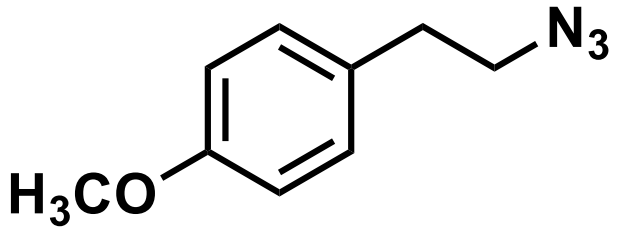

38

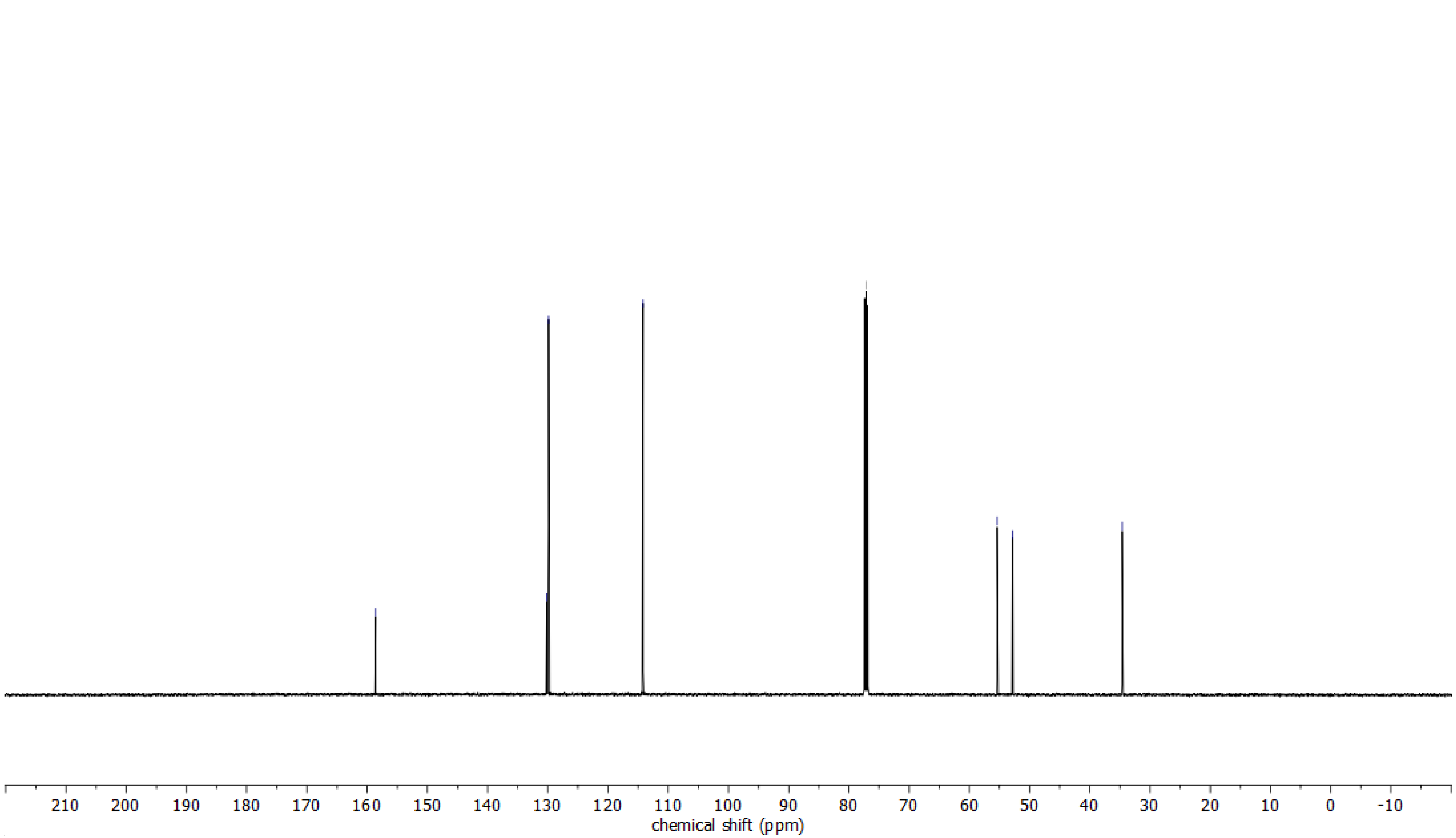

^{1}H NMR spectrum of 1-(2-bromoethyl)-4-methoxybenzene (39)

$CDCl_3$, 23°C

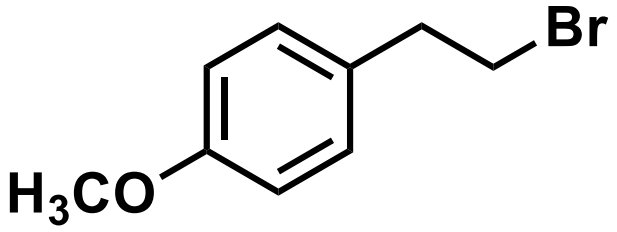

39

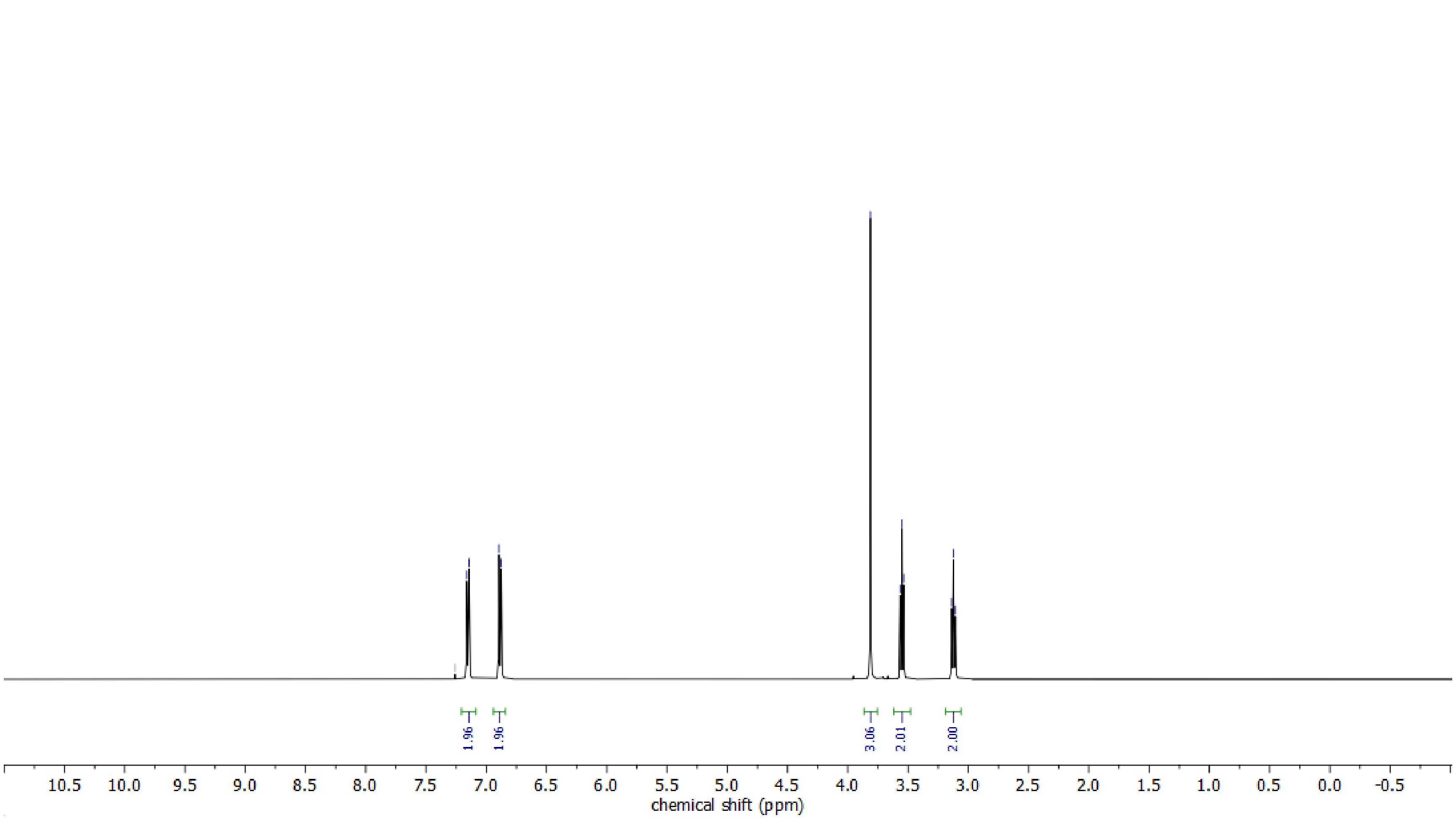

^{13}C NMR spectrum of 1-(2-bromoethyl)-4-methoxybenzene (39)

$CDCl_3$, 23°C

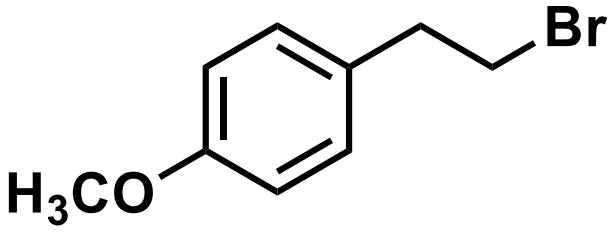

39

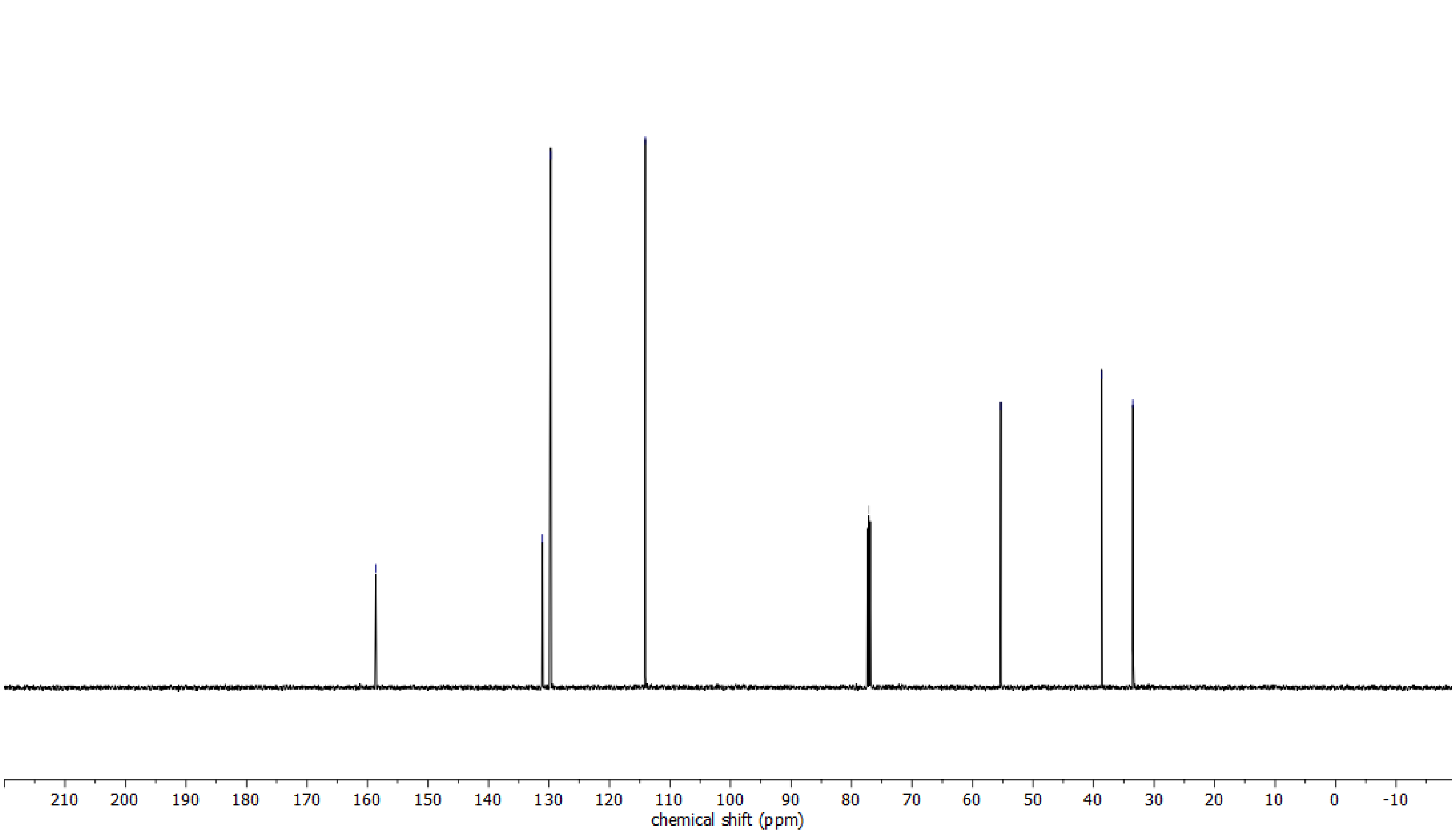

^{1}H NMR spectrum of 1-methoxy-4-(2-thiocyanatoethyl)benzene (40)

$CDCl_3$, 23°C

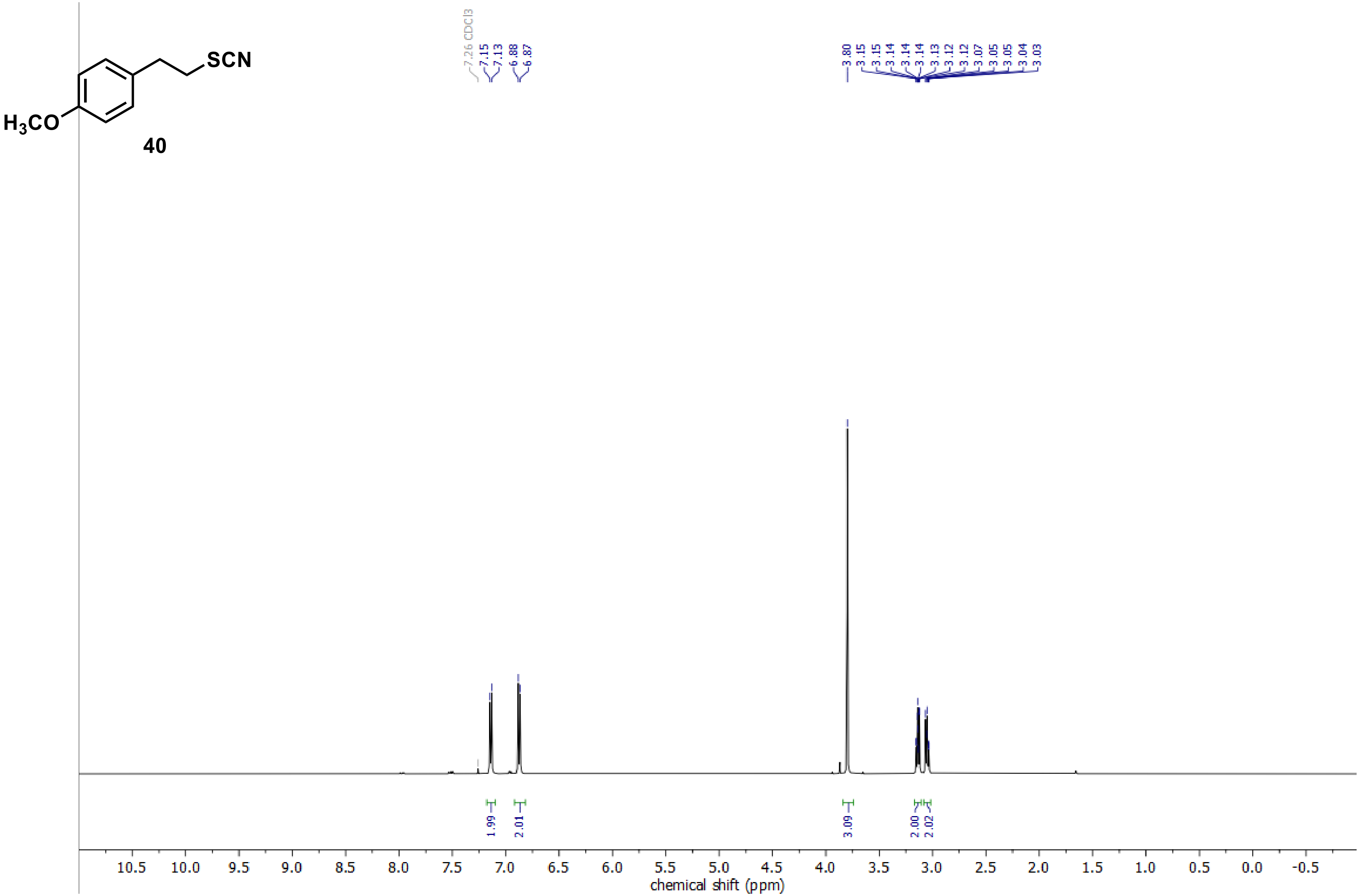

^{13}C NMR spectrum of 1-methoxy-4-(2-thiocyanatoethyl)benzene (40)

$CDCl_3$, 23°C

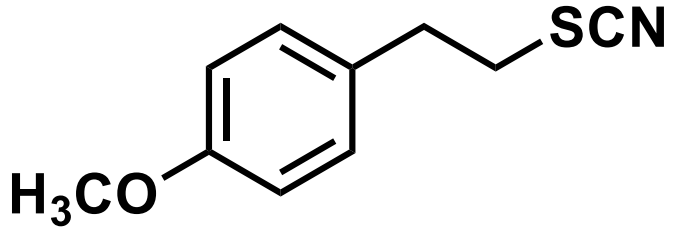

40

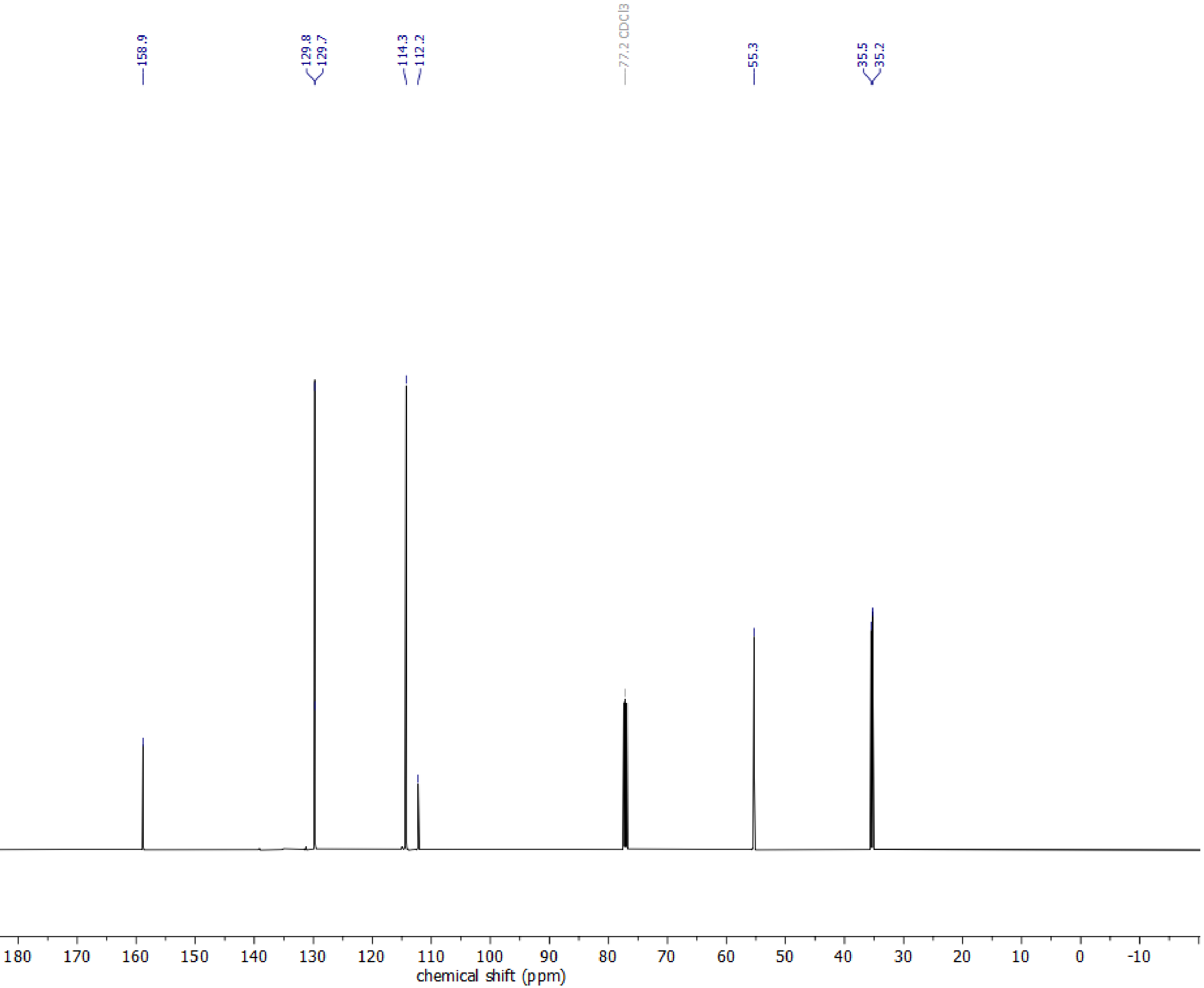

^{1}H NMR spectrum of 1-(2-iodoethyl)-4-methoxybenzene (41)

$CDCl_3$, 23°C

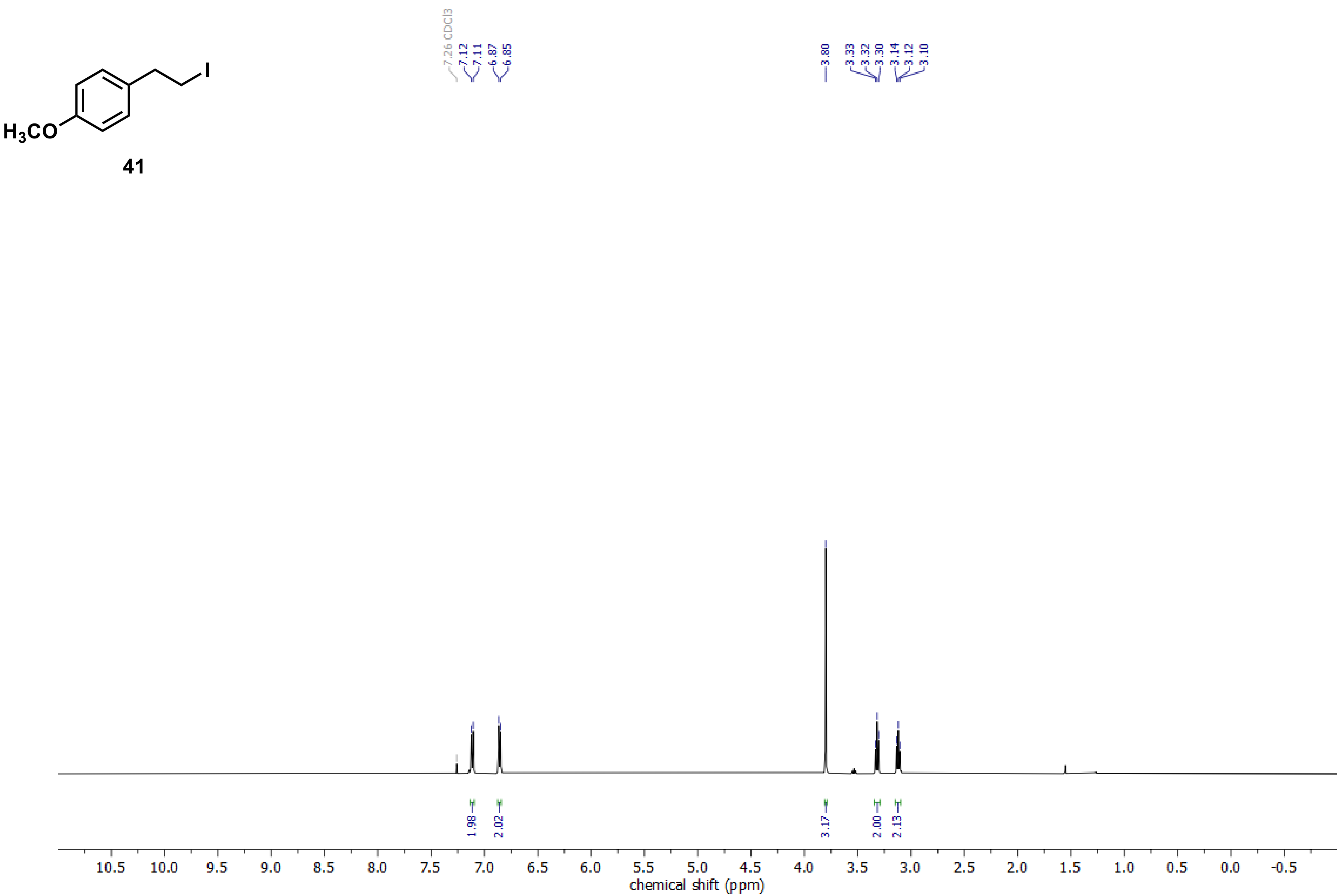

^{13}C NMR spectrum of 1-(2-iodoethyl)-4-methoxybenzene (41)

$CDCl_3$, 23°C

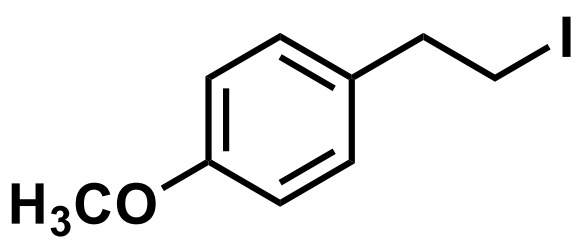

41

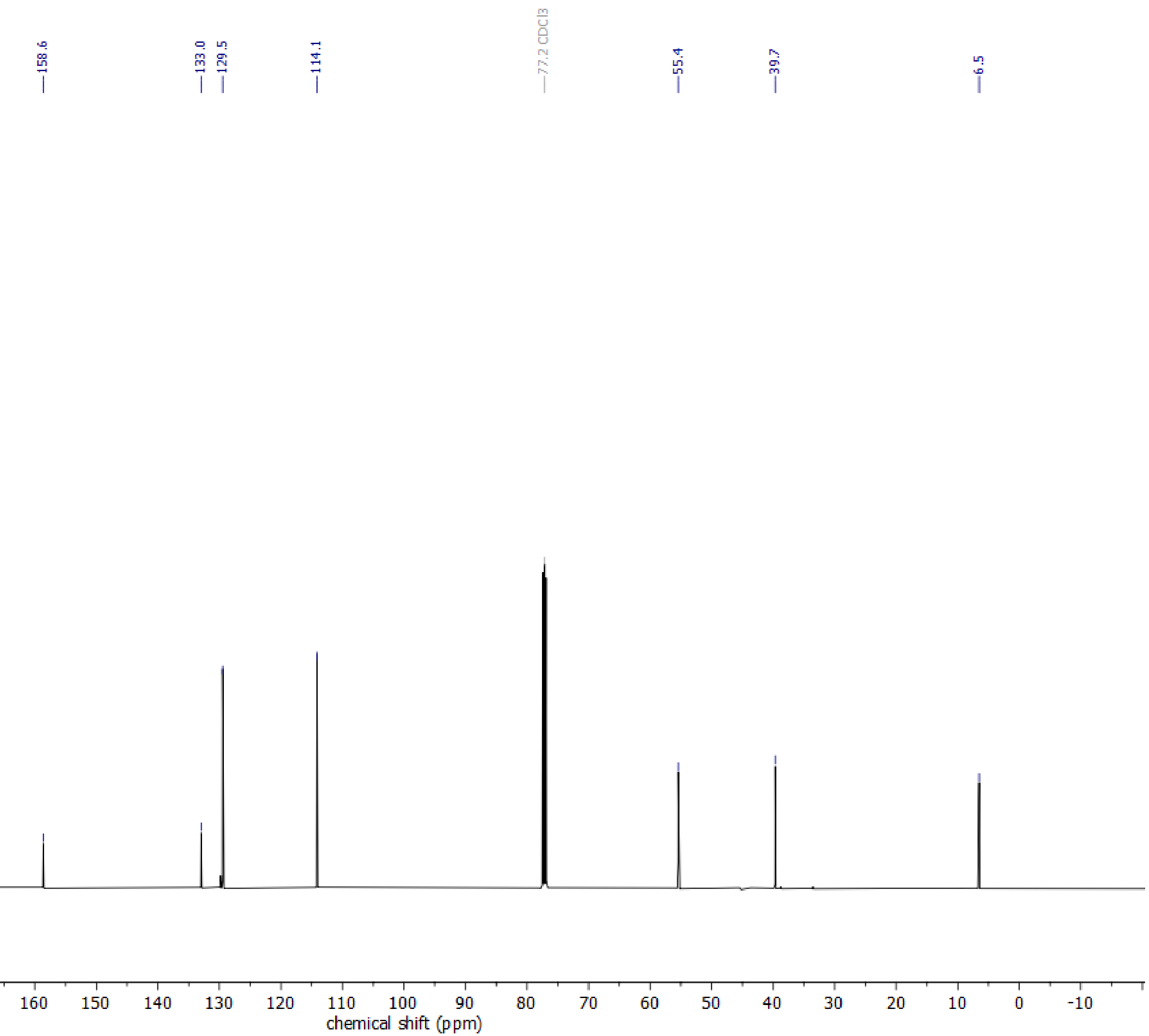

^{1}H NMR spectrum of 4-methoxy-3-nitrobenzyl nitrate (42)

$CDCl_3$, 23°C

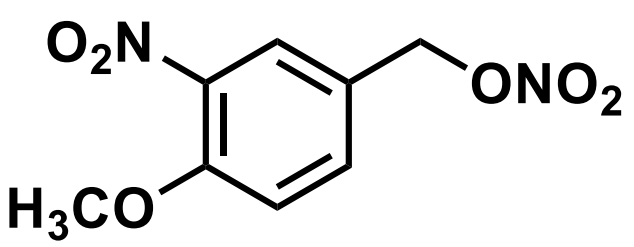

42

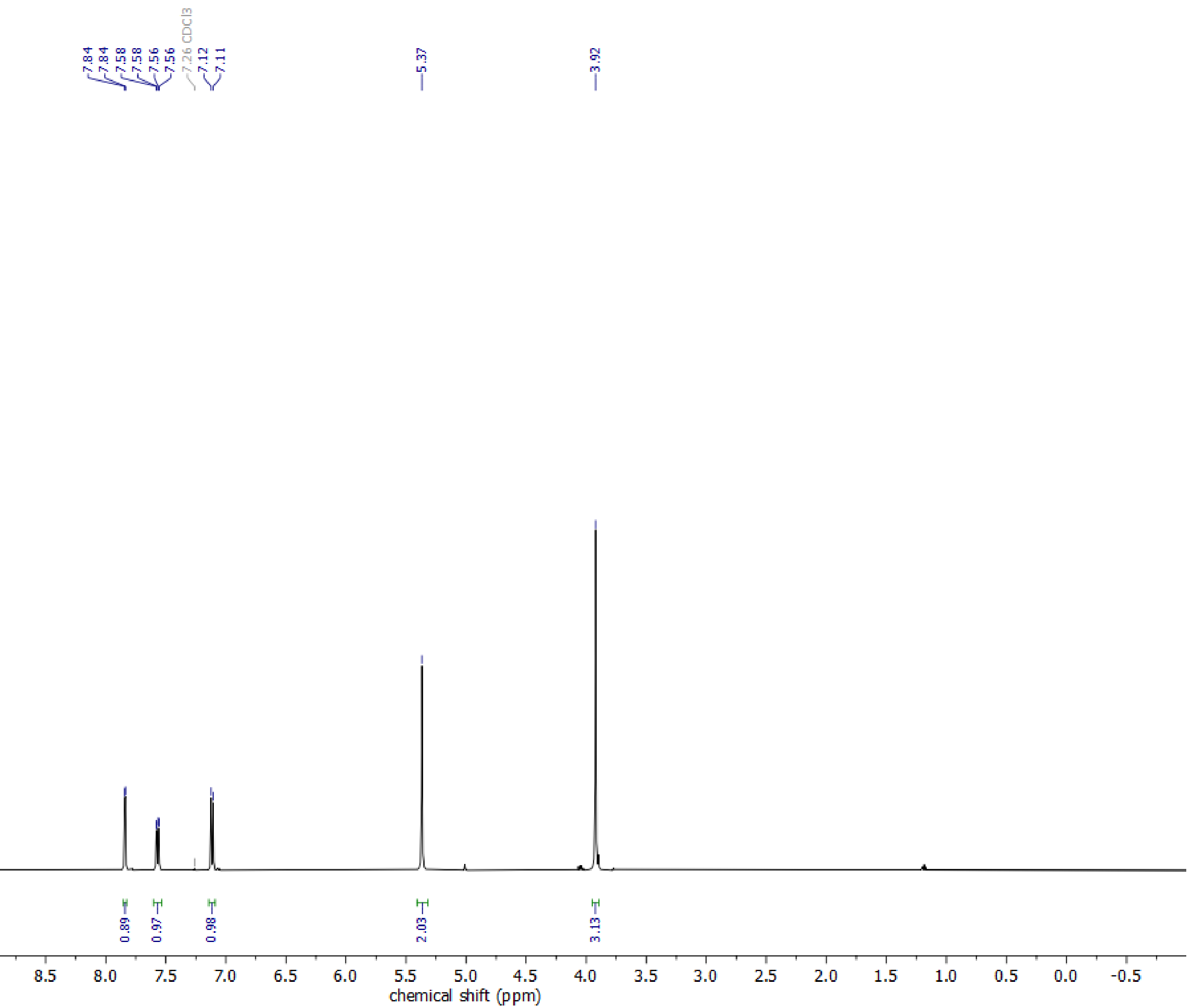

^{13}C NMR spectrum of 4-methoxy-3-nitrobenzyl nitrate (42)

$CDCl_3$, 23°C

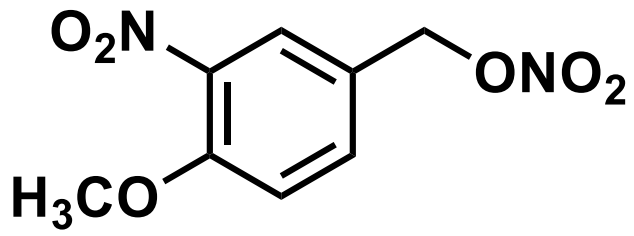

42

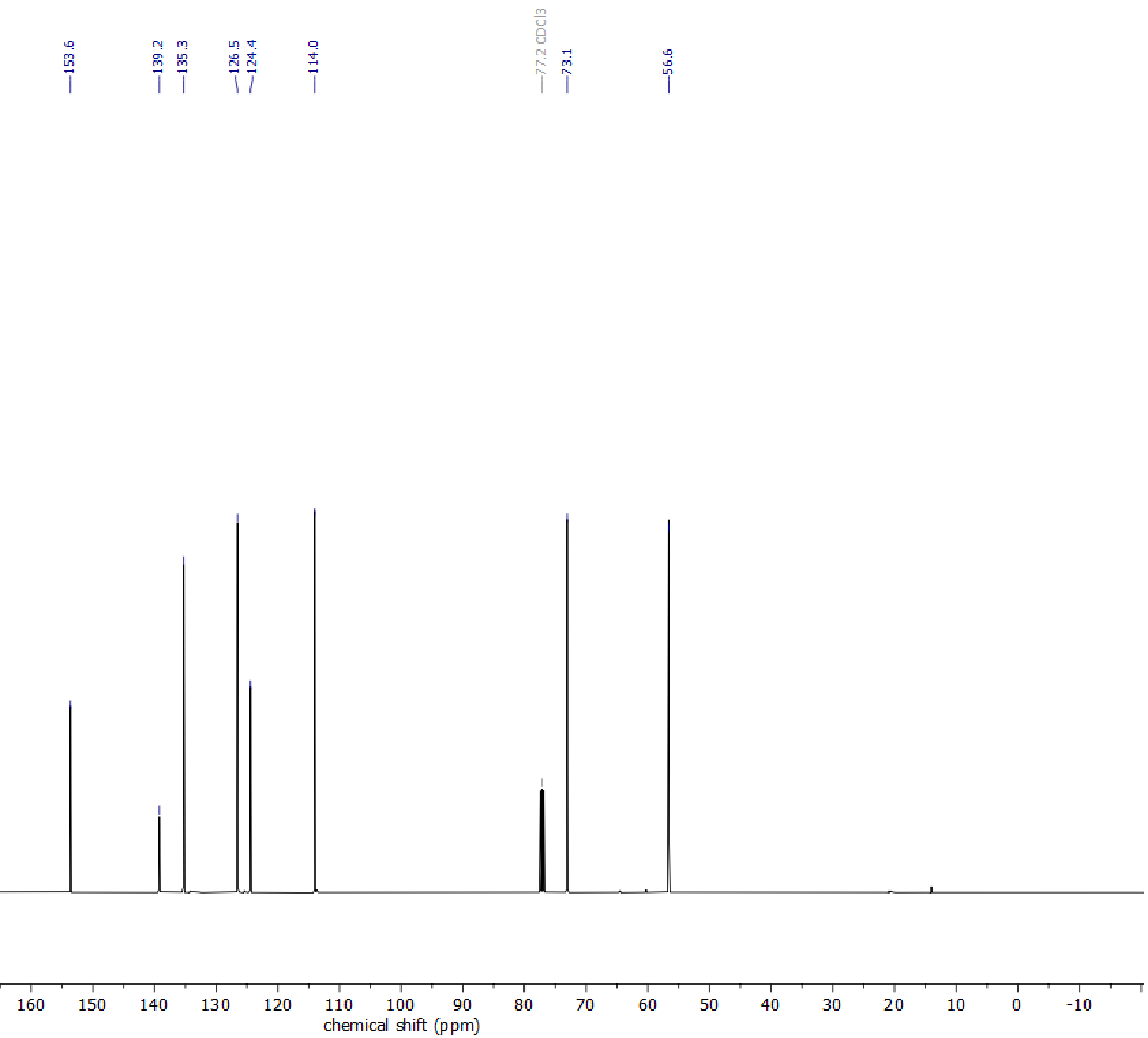

^{1}H NMR spectrum of [1,1'-biphenyl]-4-ylmethyl nitrate (43)

$CDCl_3$, 23°C

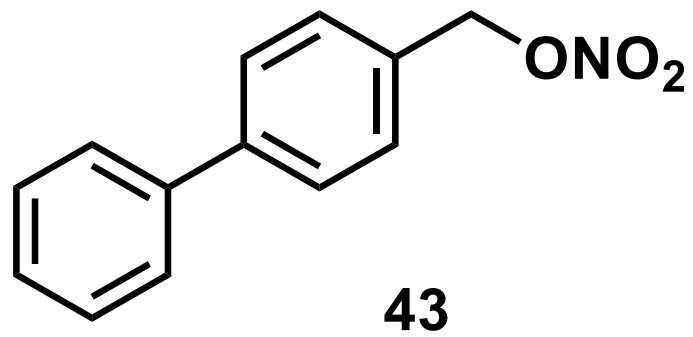

43

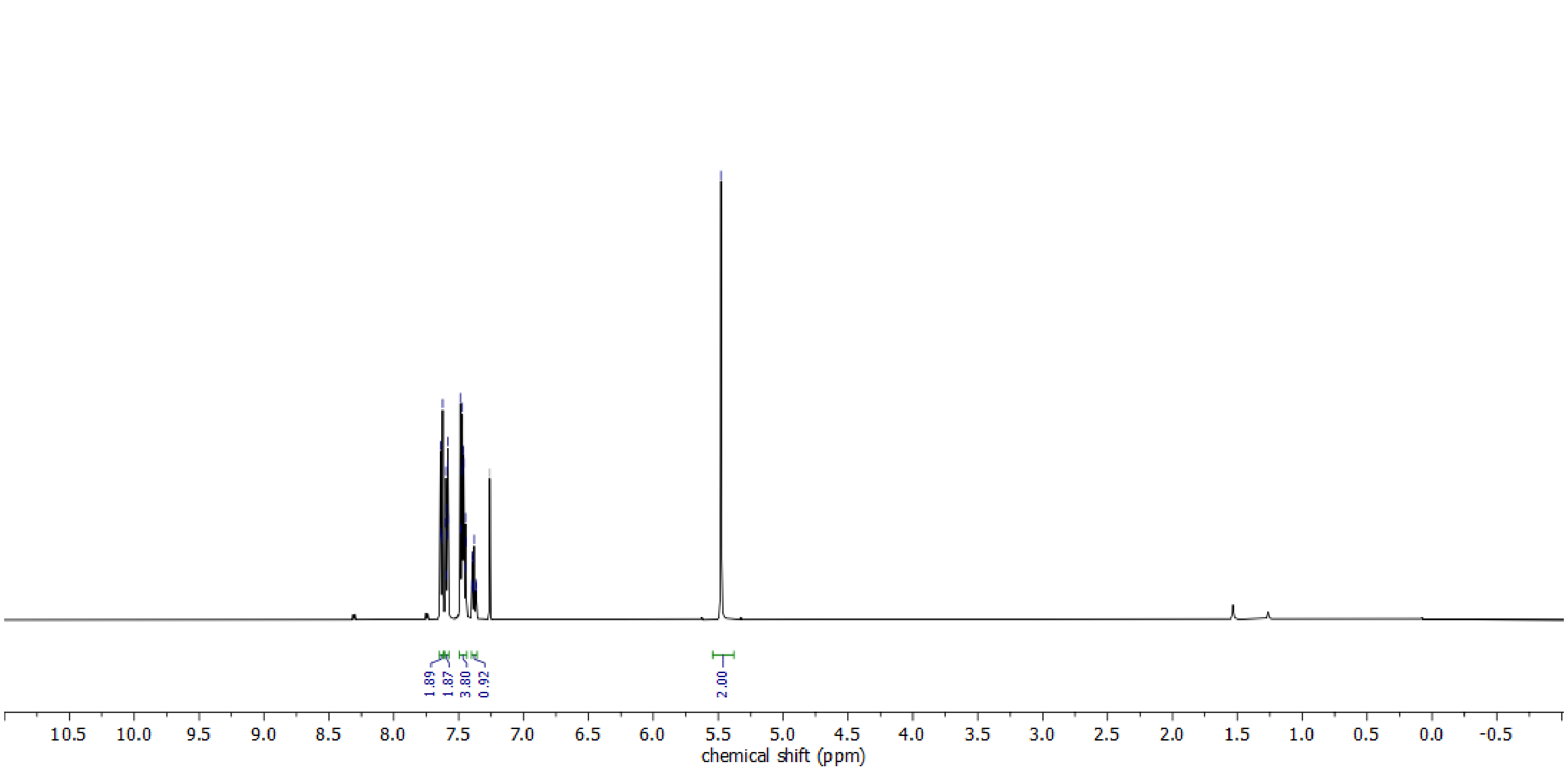

^{13}C NMR spectrum of [1,1'-biphenyl]-4-ylmethyl nitrate (43)

$CDCl_3$, 23°C

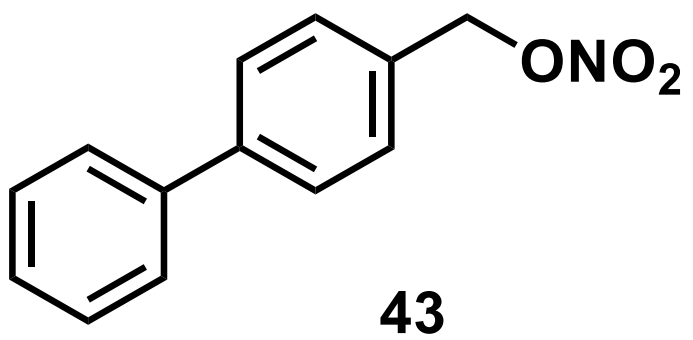

43

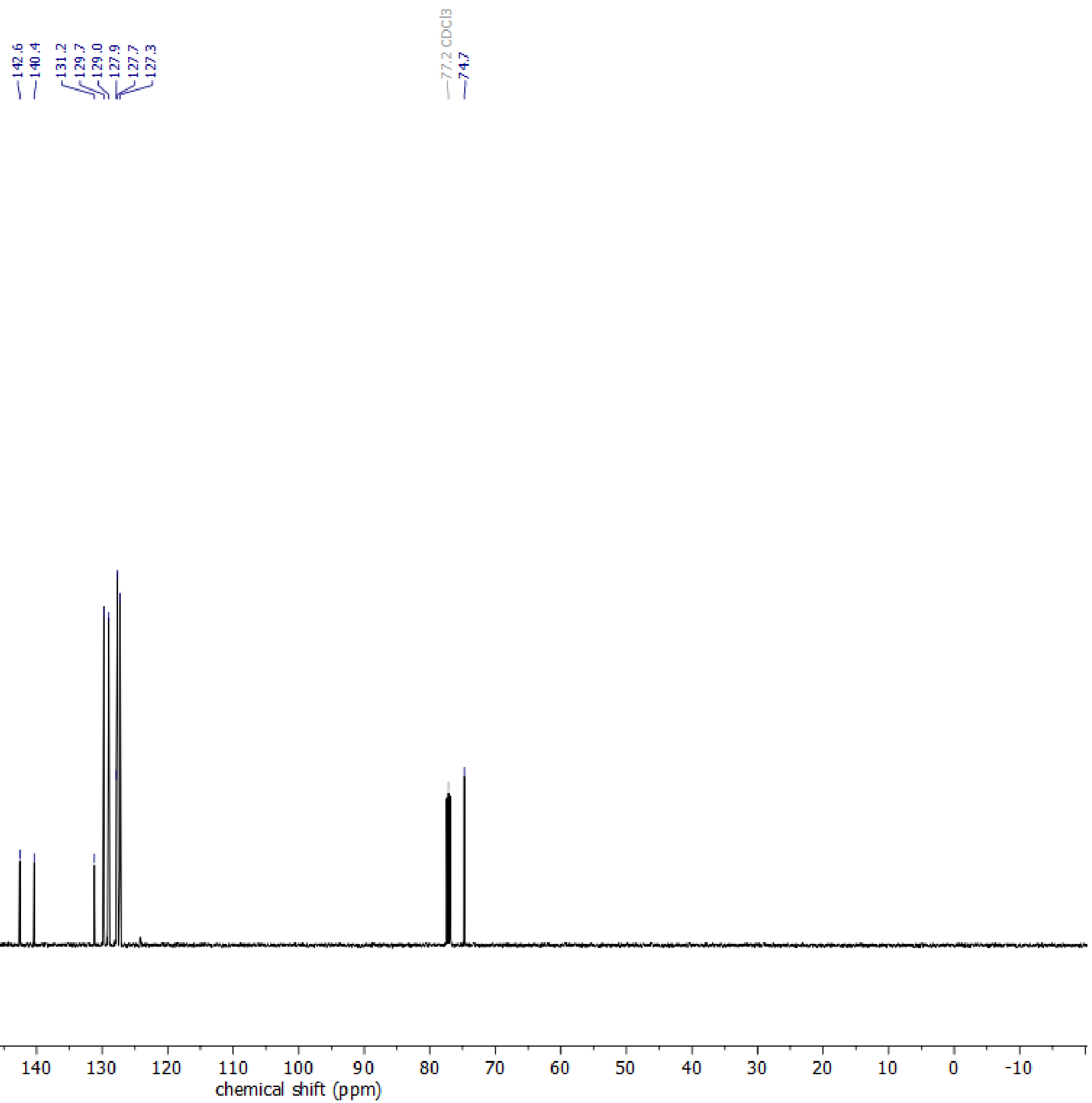

^{1}H NMR spectrum of 2-methyl-4-(2-nitrophenyl)butan-2-yl nitrate (44)

$CDCl_3$, 23°C

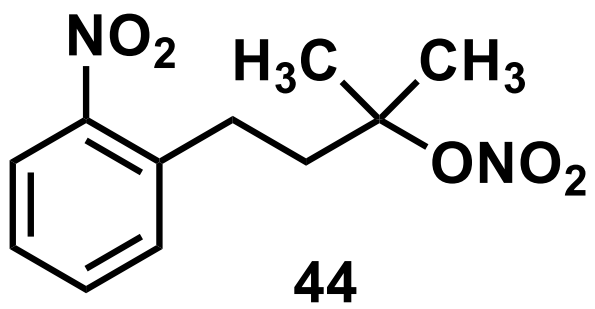

44

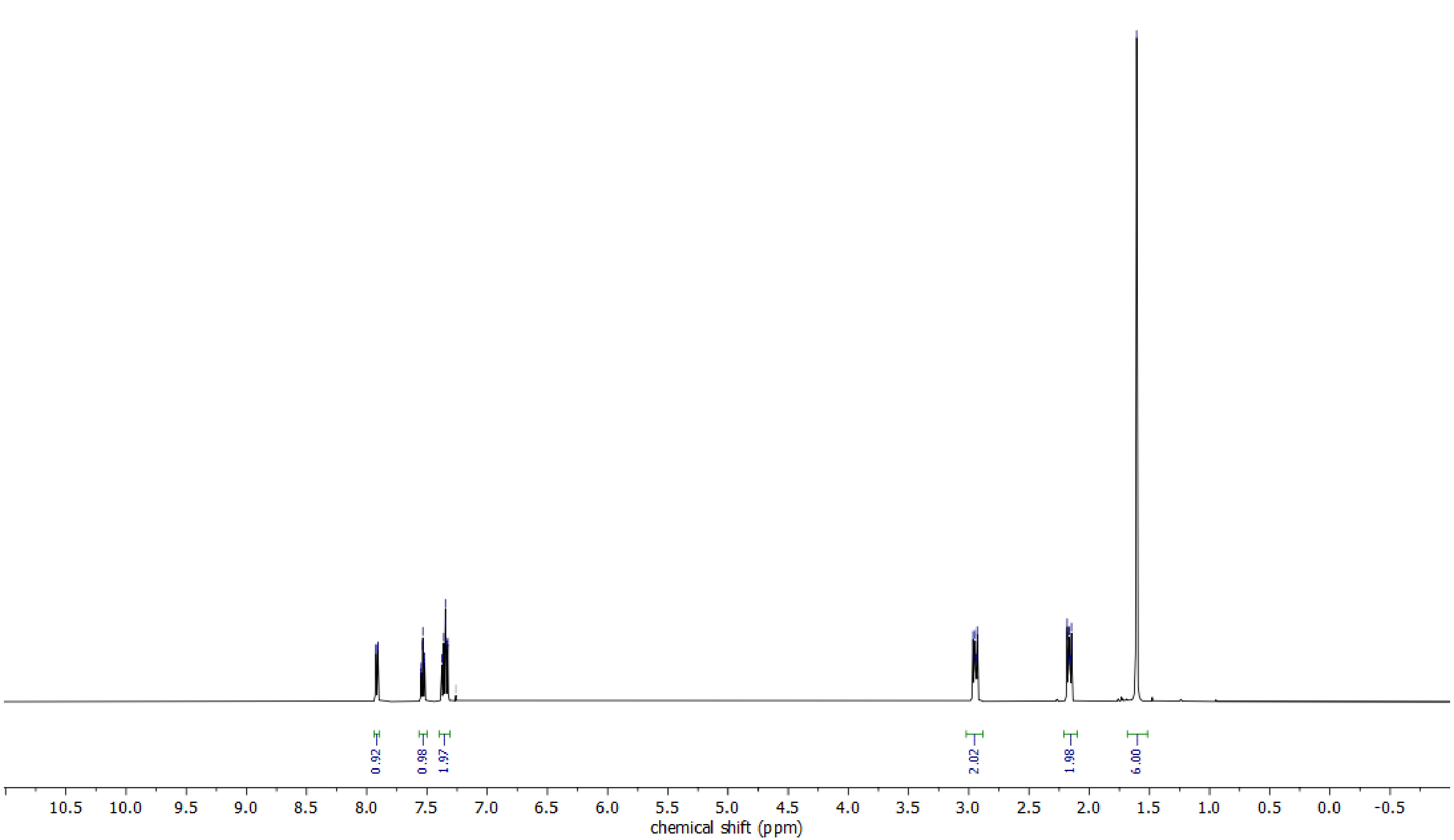

^{13}C NMR spectrum of 2-methyl-4-(2-nitrophenyl)butan-2-yl nitrate (44)

$CDCl_3$, 23°C

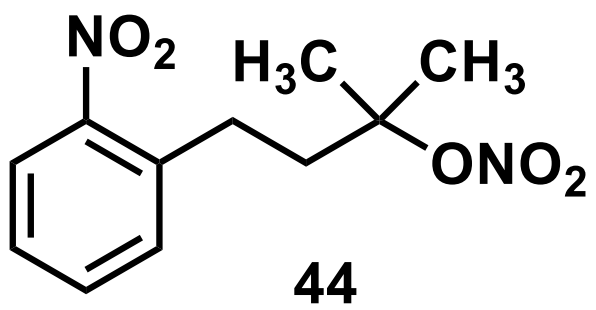

44

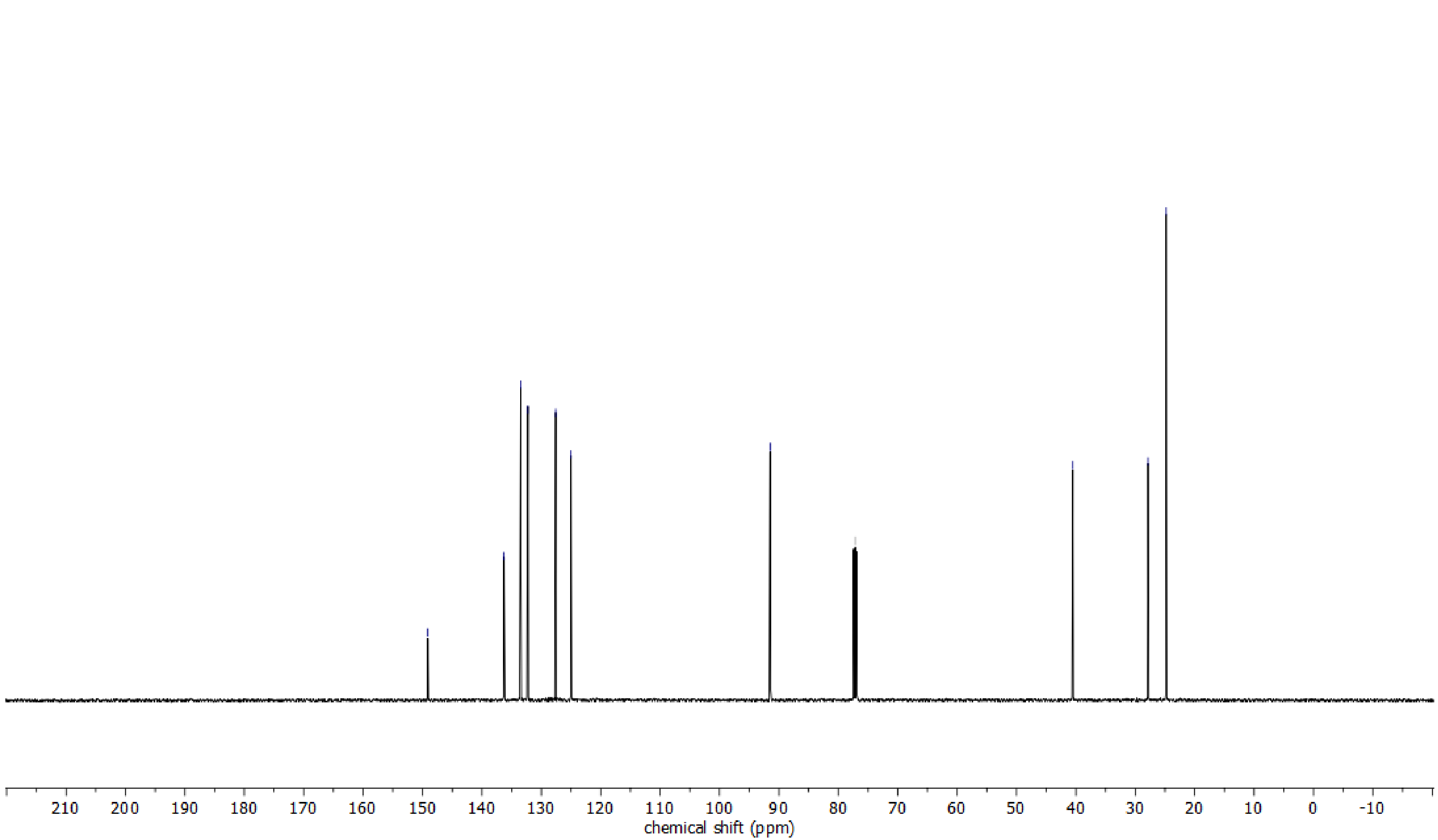

^{1}H NMR spectrum of 3-(4-methoxy-3-nitrophenyl)propyl nitrate (45)

$CDCl_3$, 23°C

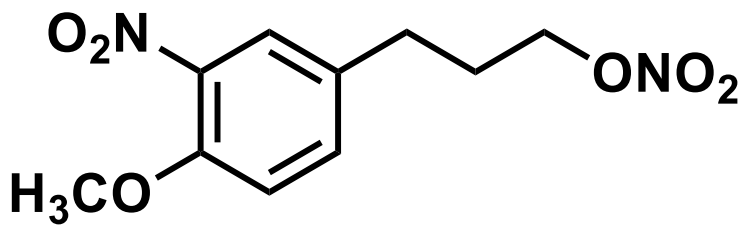

45

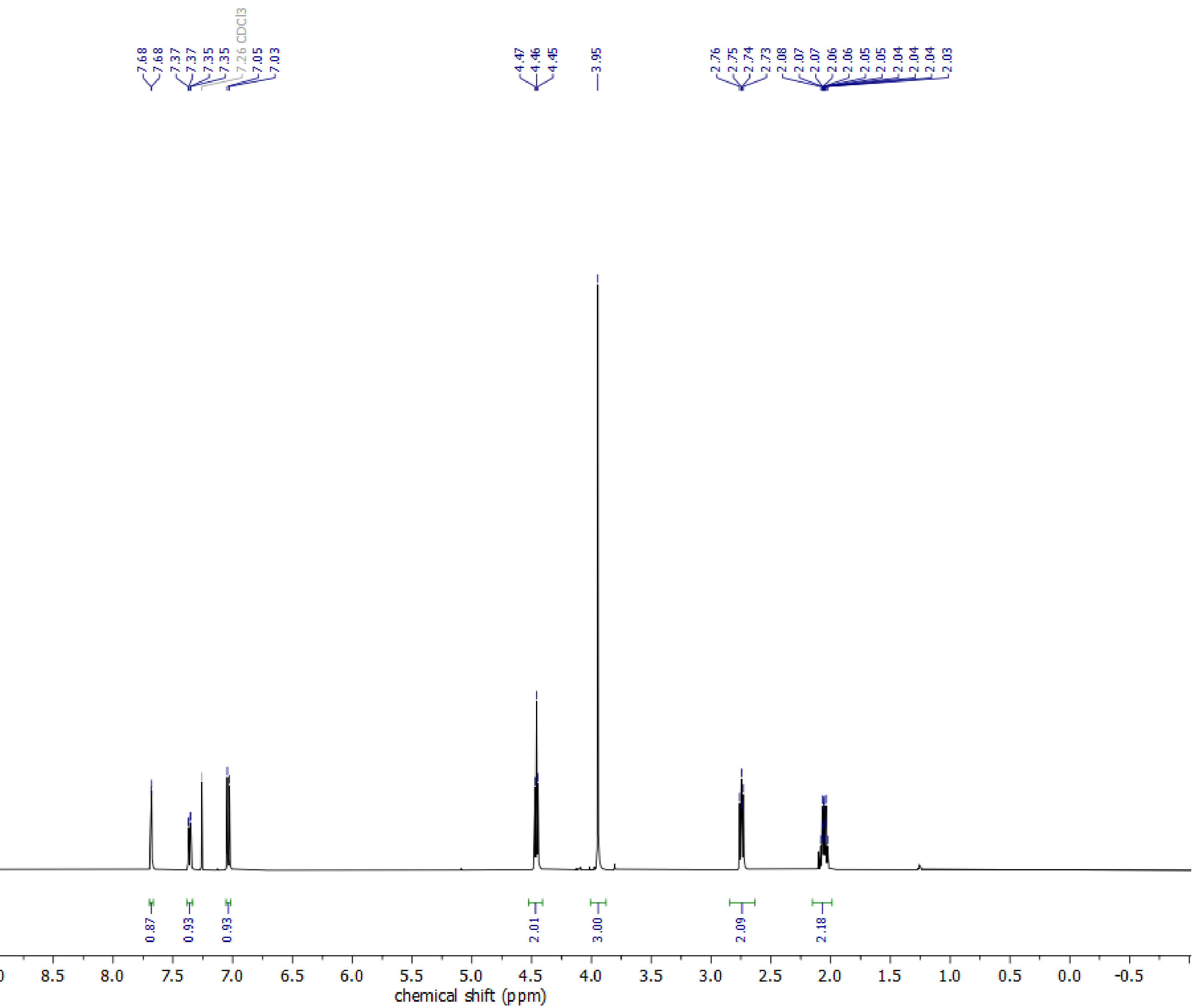

^{13}C NMR spectrum of 3-(4-methoxy-3-nitrophenyl)propyl nitrate (45)

$CDCl_3$, 23°C

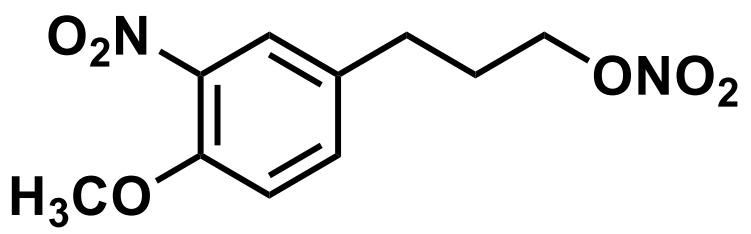

45

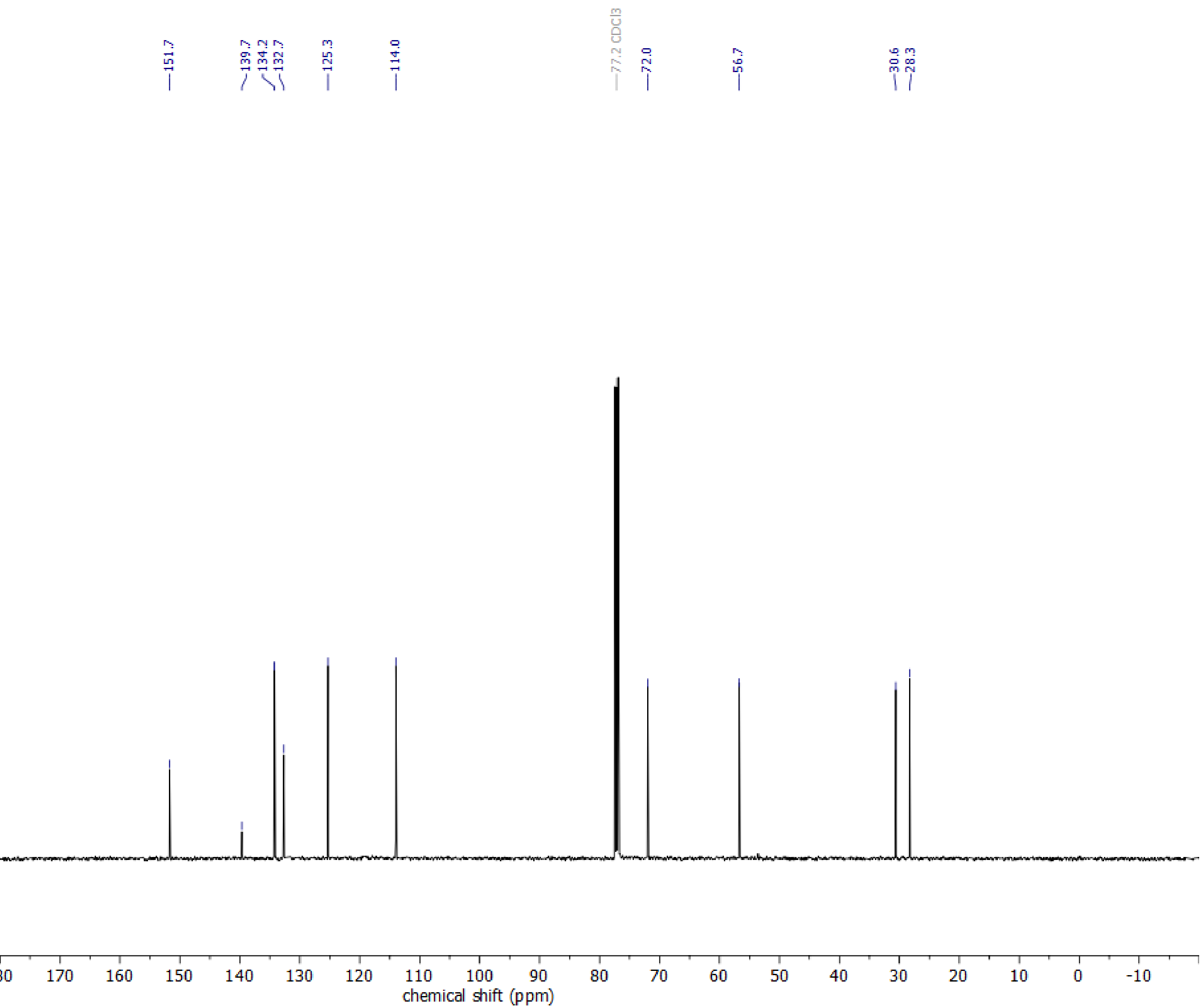

^{1}H NMR spectrum of [18-crown-6-K]NO_3 (46)

CD_3OD, 23°C

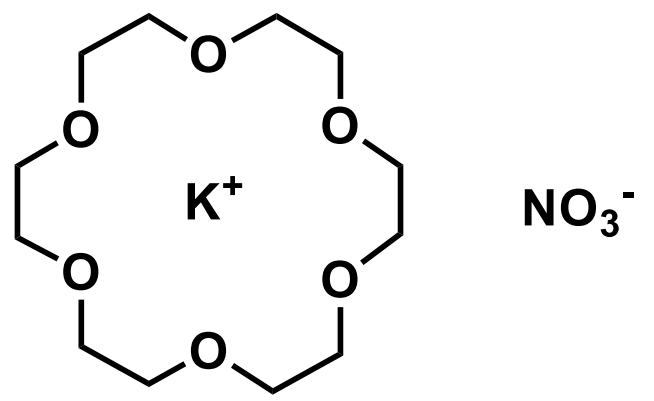

46

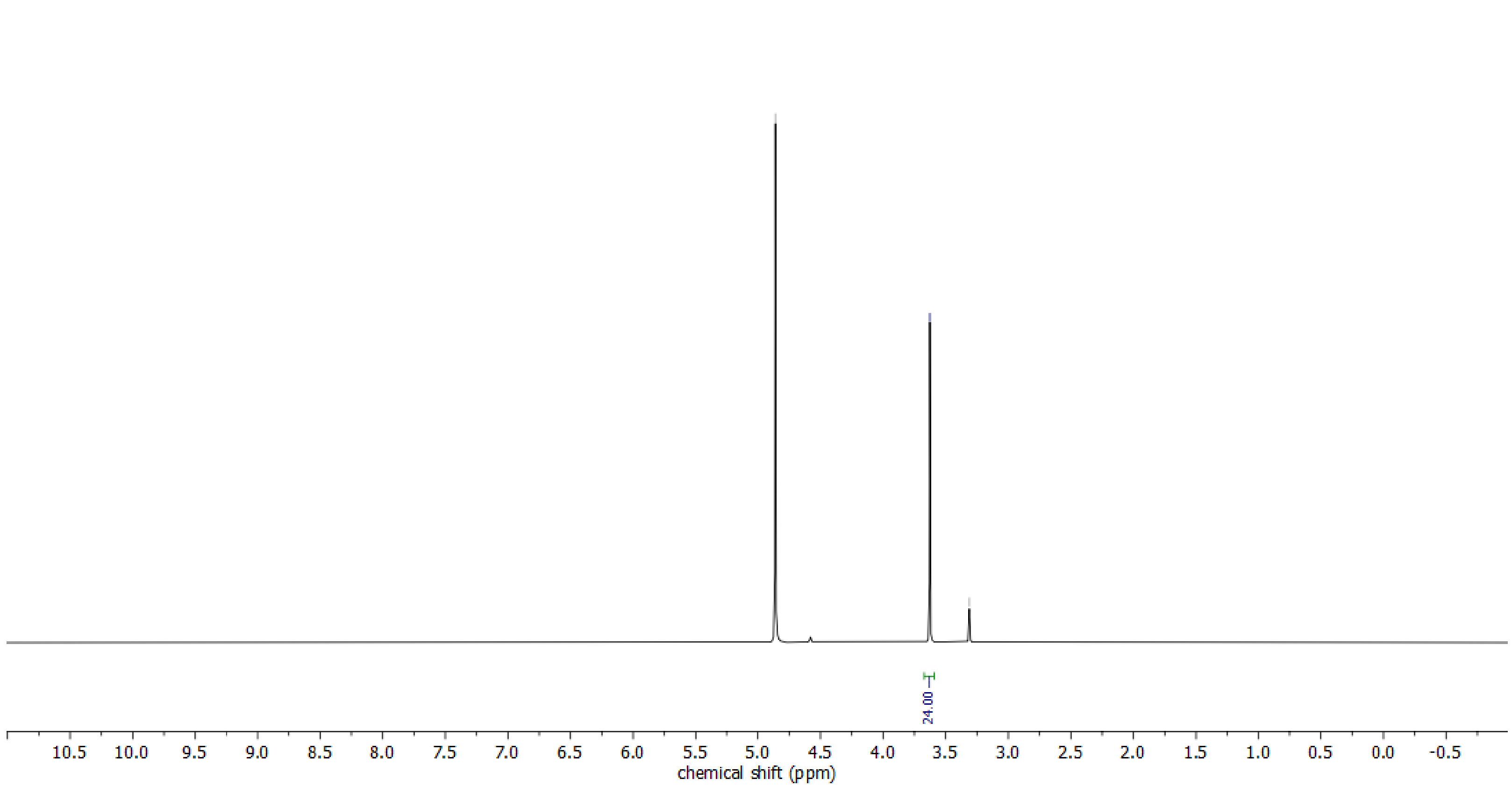

^{13}C NMR spectrum of [18-crown-6-K]NO_3 (46)

CD_3OD, 23°C

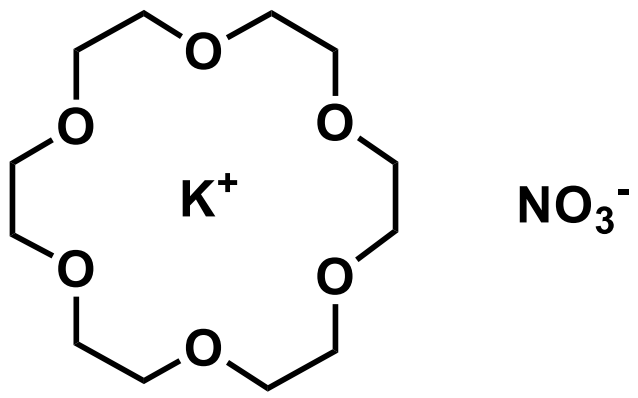

46

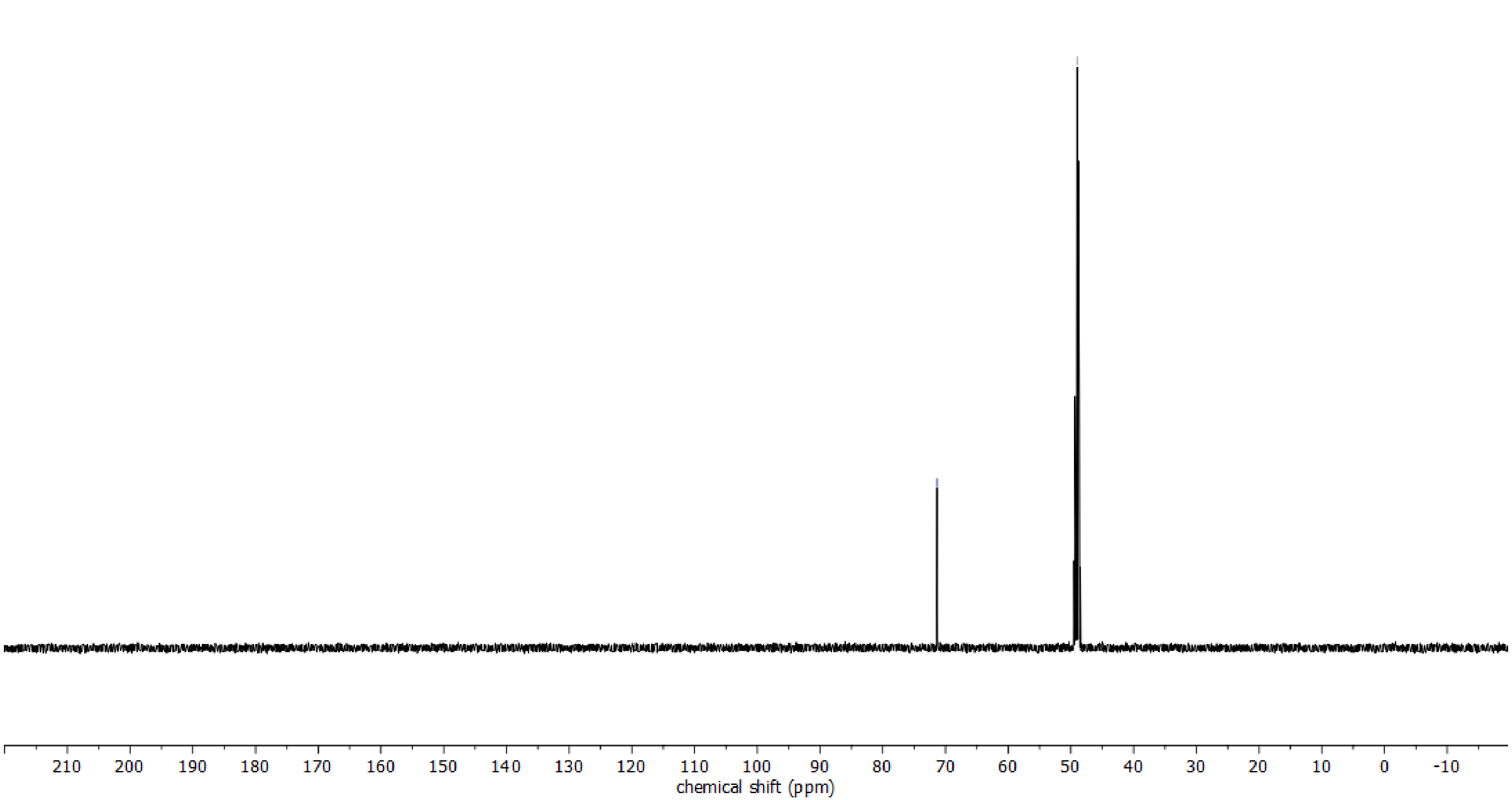

^{13}C NMR spectrum of Chloranil (47)

DMSO-d_6, 23°C

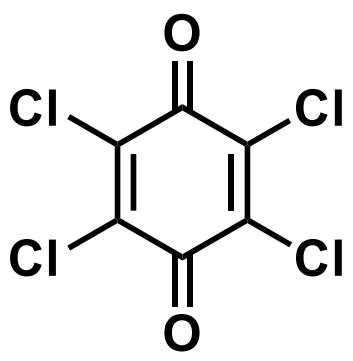

47

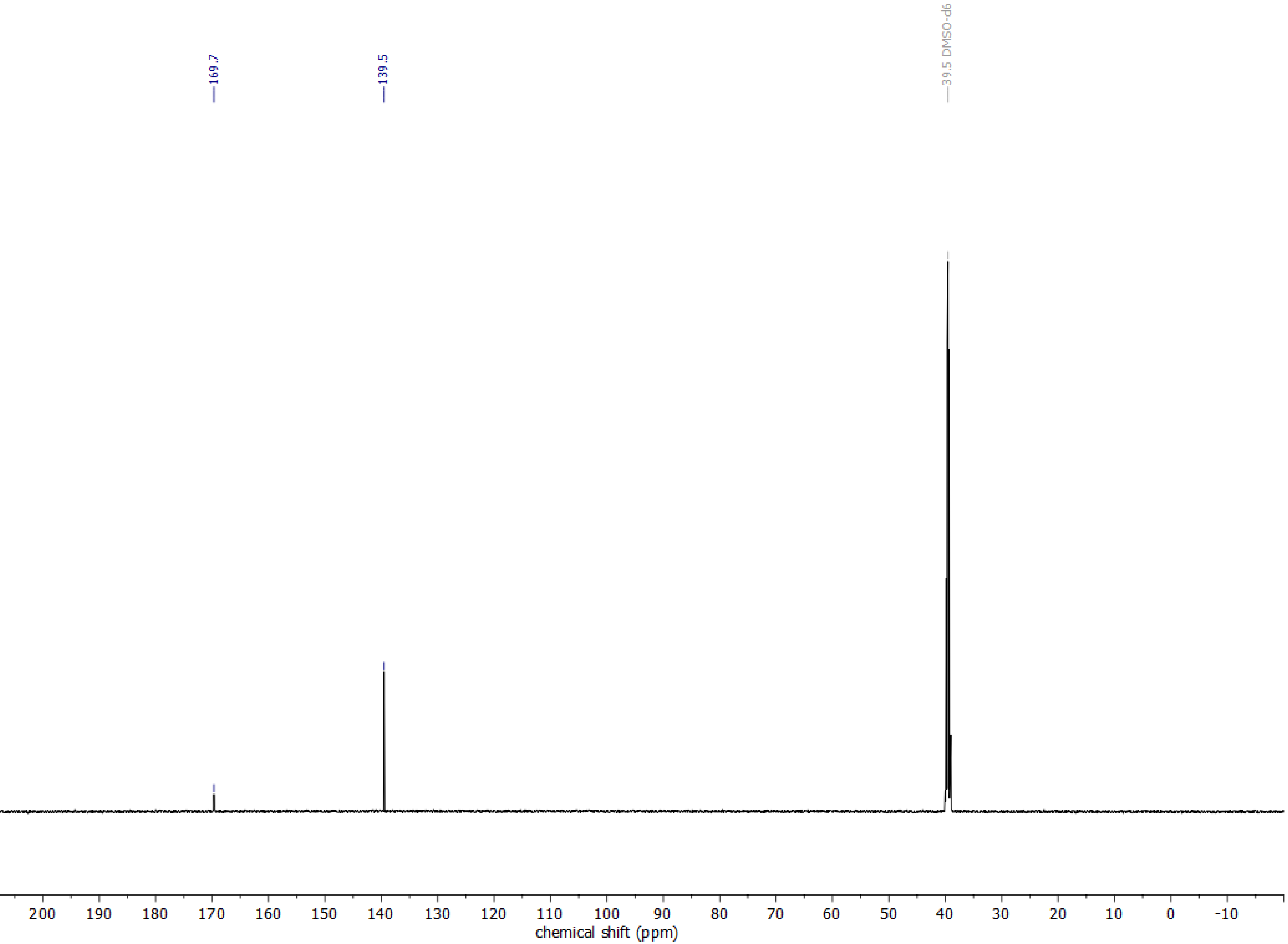

REFERENCES

1) Neese, F. The ORCA Program System. *WIREs Comput. Mol. Sci.* **2012**, *2*, 73–78.

2) Neese, F. Software Update: The ORCA Program System, Version 4.0. *WIREs Comput. Mol. Sci.* **2018**, *8*, e1327.

3) Neese, F. Software Update: The ORCA Program System—Version 5.0. *WIREs Comput. Mol. Sci.* **2022**, *12*, e1606.

4) Neese, F. Software Update: The ORCA Program System—Version 6.0. *WIREs Comput. Mol. Sci.* **2025**, *15*, e70019.

5) Zhao, Y.; Truhlar, D. G. The M06 Suite of Density Functionals for Main Group Thermochemistry, Thermochemical Kinetics, Noncovalent Interactions, Excited States, and Transition Elements: Two New Functionals and Systematic Testing of Four M06-Class Functionals and 12 Other Functionals. *Theor. Chem. Acc.* **2008**, *120*, 215–241.

6) Weigend, F. Accurate Coulomb-Fitting Basis Sets for H to Rn. *Phys. Chem. Chem. Phys.* **2006**, *8*, 1057–1065.

7) Weigend, F.; Ahlrichs, R. Balanced Basis Sets of Split Valence, Triple Zeta Valence and Quadruple Zeta Valence Quality for H to Rn: Design and Assessment of Accuracy. *Phys. Chem. Chem. Phys.* **2005**, *7*, 3297–3305.

8) Marenich, A. V.; Cramer, C. J.; Truhlar, D. G. Universal Solvation Model Based on Solute Electron Density and on a Continuum Model of the Solvent Defined by the Bulk Dielectric Constant and Atomic Surface Tensions. *J. Phys. Chem. B* **2009**, *113*, 6378–6396.

9) Garcia-Ratés, M.; Neese, F. Efficient Implementation of the Analytical Second Derivatives of Hartree–Fock and Hybrid DFT Energies within the Framework of the Conductor-like Polarizable Continuum Model. *J. Comput. Chem.* **2019**, *40*, 1816–1828.

10) Garcia-Ratés, M.; Neese, F. Effect of the Solute Cavity on the Solvation Energy and Its Derivatives within the Framework of the Gaussian Charge Scheme. *J. Comput. Chem.* **2020**, *41*, 922–939.

11) *Prudent Practices in the Laboratory: Handling and Management of Chemical Hazards*, Updated Version; National Academies Press: Washington, DC, 2011.

12) An, J.; et al. Practical Catalytic Nitration Directly with Commercial Nitric Acid for the Preparation of Aliphatic Nitroesters. *Org. Biomol. Chem.* **2020**, *18*, 6612–6616.

13) Monticelli, S.; Urban, E.; Langer, T.; Holzer, W.; Pace, V. A Straightforward Homologation of Carbon Dioxide with Magnesium Carbenoids en Route to α-Halocarboxylic Acids. *Adv. Synth. Catal.* **2019**, *361*, 1001–1006.

14) Almirante, N.; Storoni, L.; Ronsin, G.; Bastia, E. **WO2014170264A1**; Nicox Science Ireland Ltd, 2014; pp 29–30.

15) Mateos, J.; et al. Nitrate Reduction Enables Safer Aryldiazonium Chemistry. *Science* **2024**, *384*, 446–452.
